# Supplementary material for: A split-GAL4 driver line resource for Drosophila neuron types
Source: eLife. 2025 Jan 24;13:RP98405. doi: 10.7554/eLife.98405 (PMC11759409; doi:10.7554/eLife.98405)
Supplement: Supplementary file 1. — Images are averaged color depth MIPs from rescreened and raw collection SS screen and polarity neuron channel images (Otsuna et al., 2018). Images were inverted, overlayed on a 2D outline of JRC2018, and composited. Depth color scale for inverted color depth MIPs is on first page, running from yellow on the anterior brain (or ventral VNC) to blue on the posterior brain (or dorsal VNC). [file elife-98405-supp1.pdf]

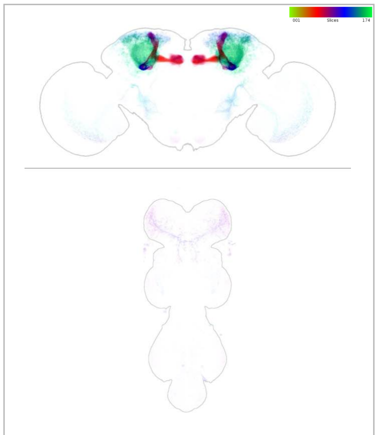

MB005B

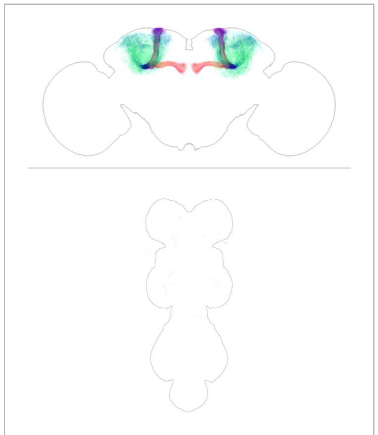

MB008B

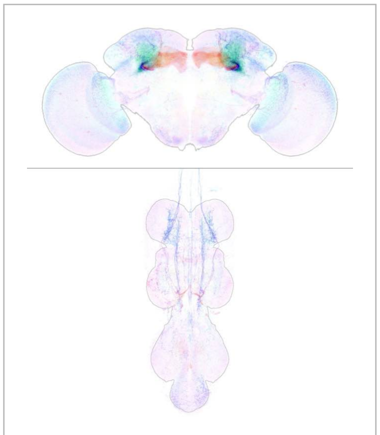

MB009B

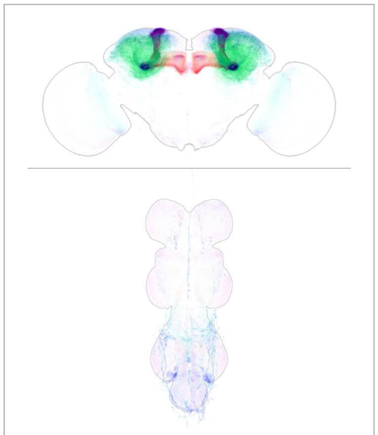

MB010B

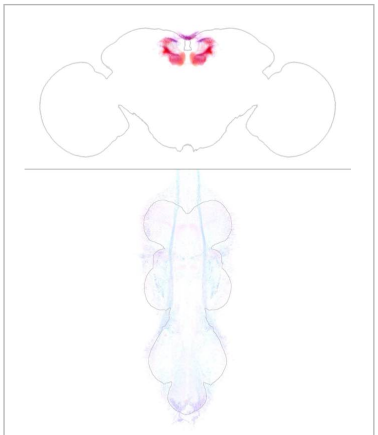

MB011B

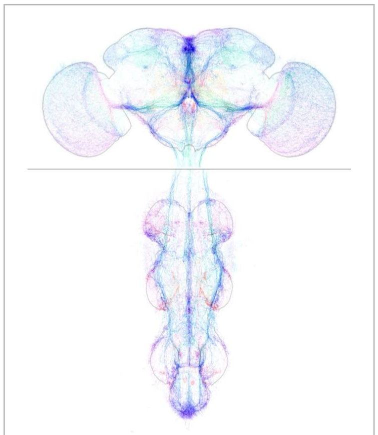

MB013B

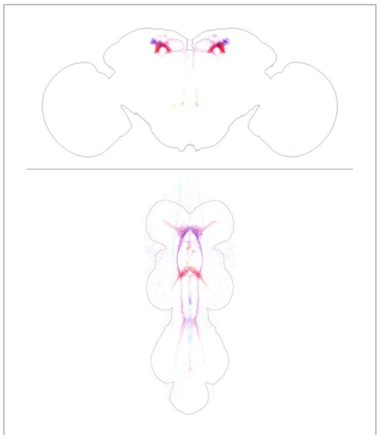

MB018B

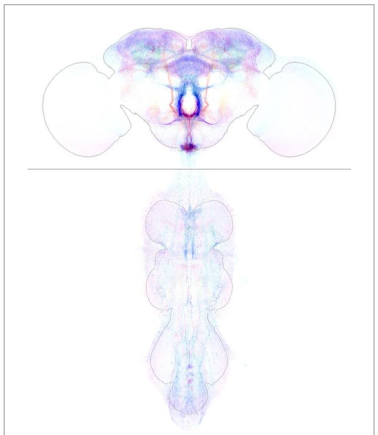

MB022B

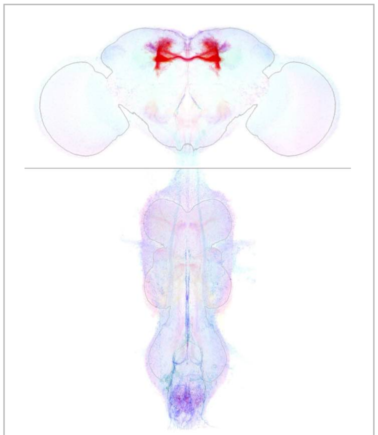

MB025B

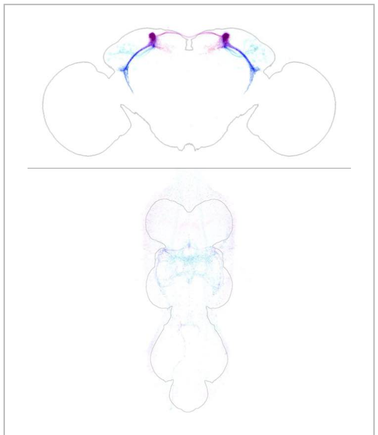

MB027B

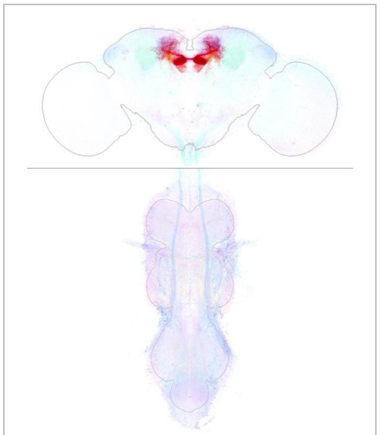

MB032B

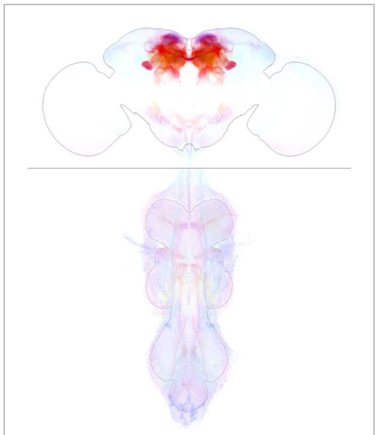

MB042B

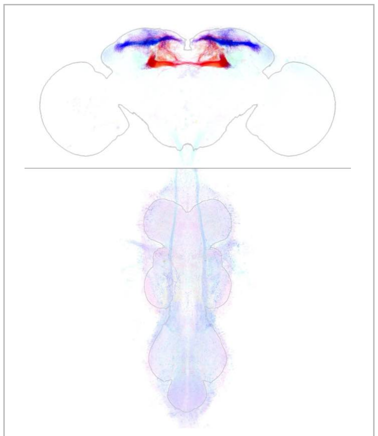

MB043B

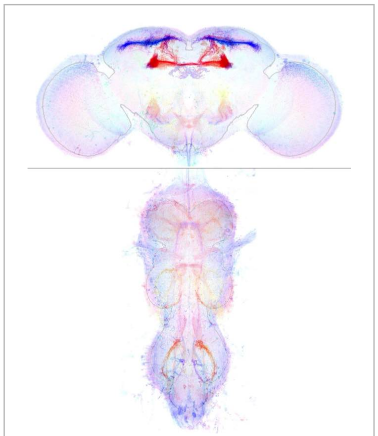

MB043C

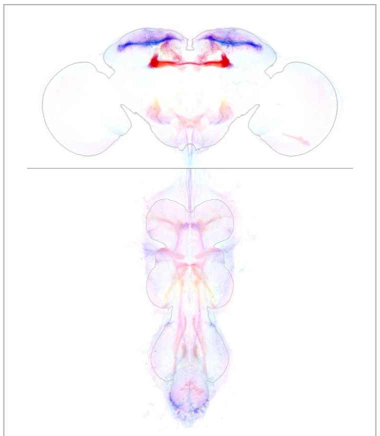

MB045B

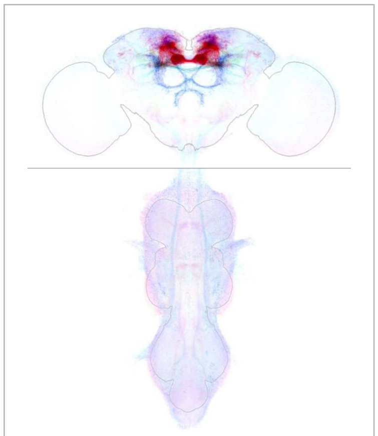

MB047B

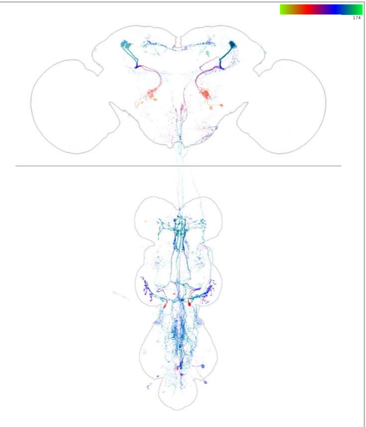
MB048B

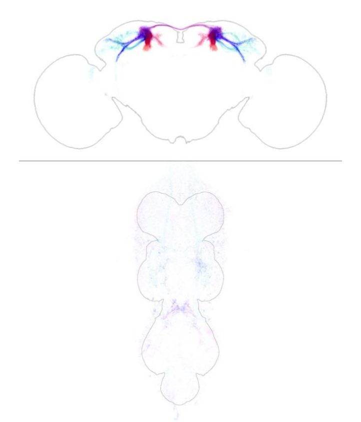
MB050B

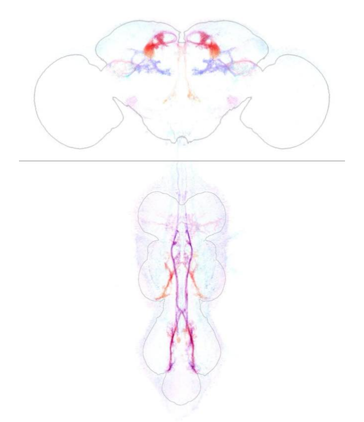
MB051B

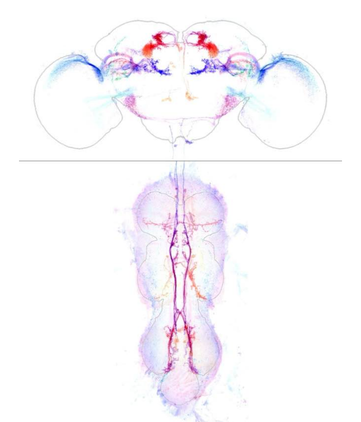
MB051C

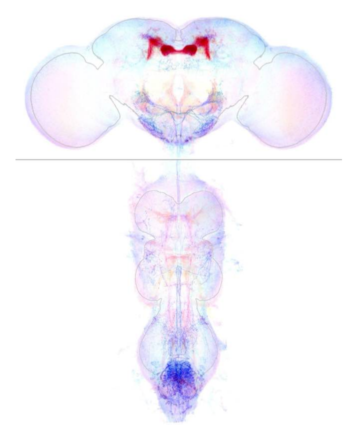
MB056B

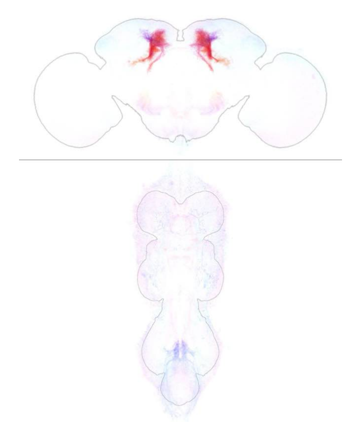
MB057B

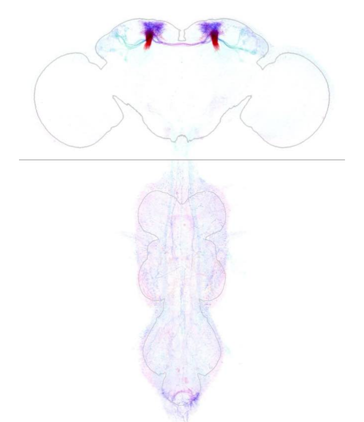
MB058B

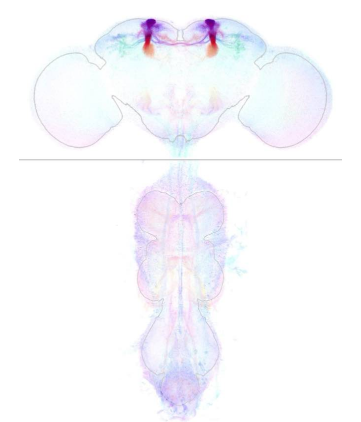
MB060B

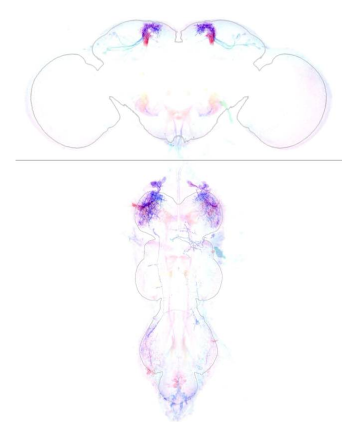
MB062B

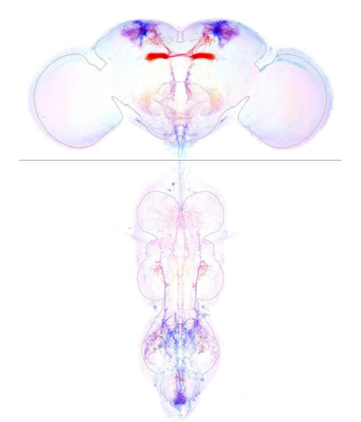
MB063B

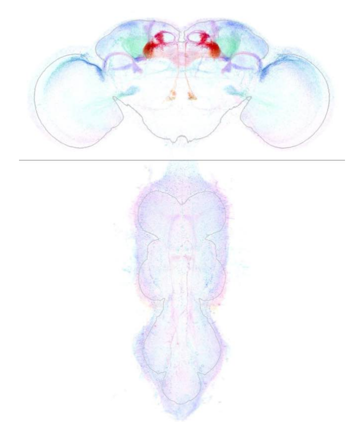
MB077C

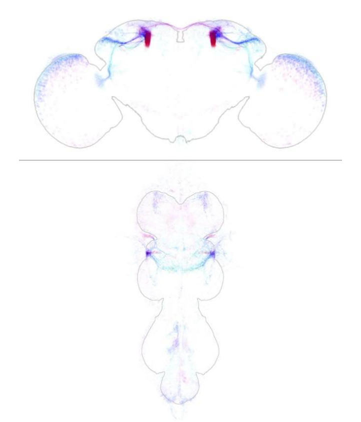
MB080C

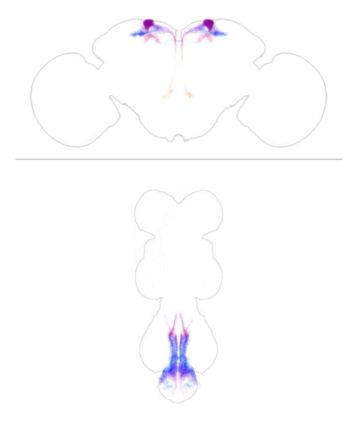
MB082C

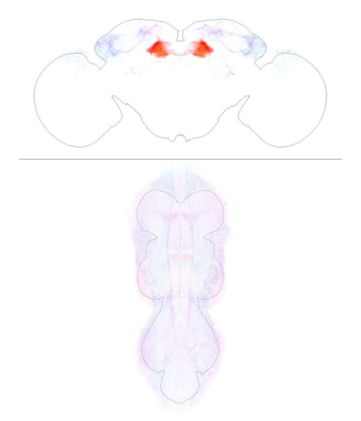
MB083C

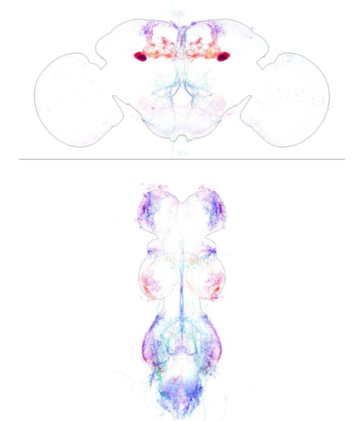
MB085C

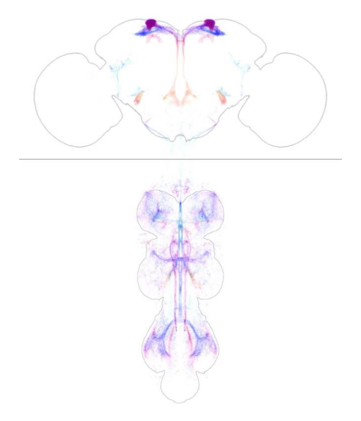
MB093C

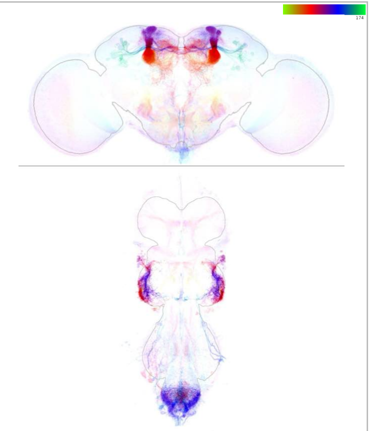
MB099C

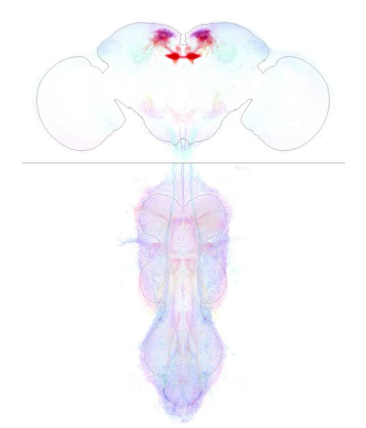
MB109B

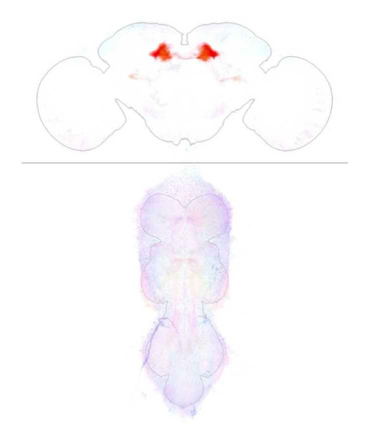
MB110C

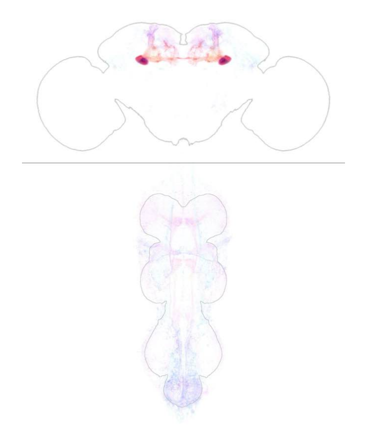
MB112C

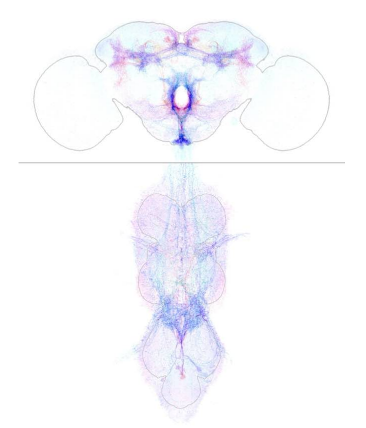
MB113C

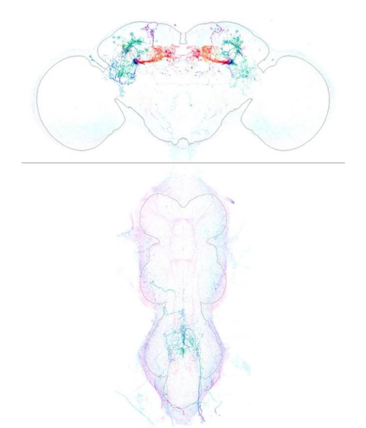
MB116B

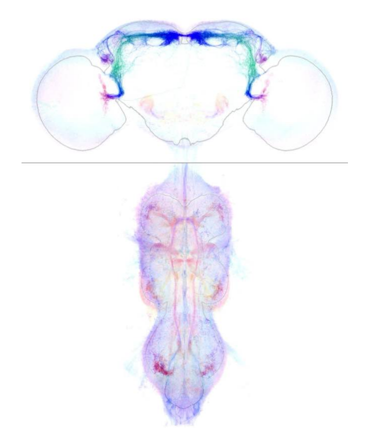
MB122B

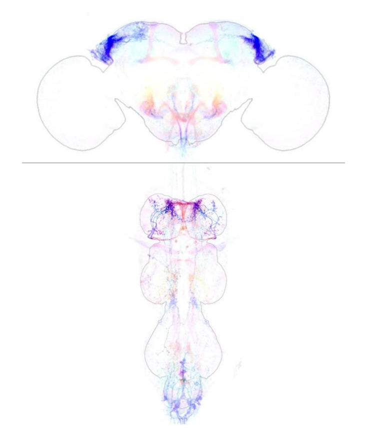
MB155B

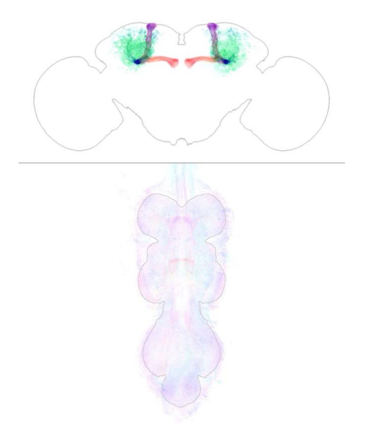
MB185B

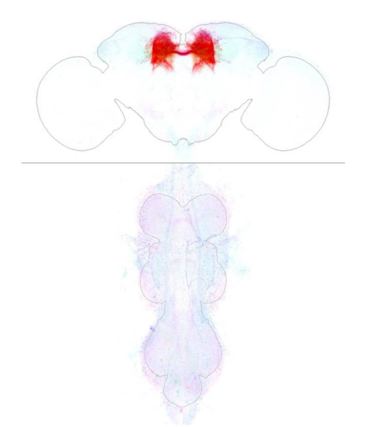
MB188B

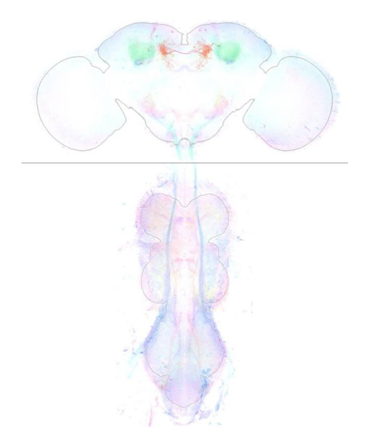
MB195B

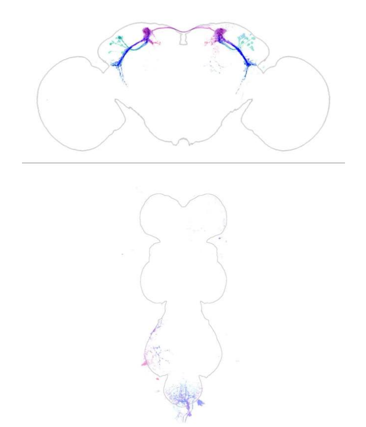
MB203B

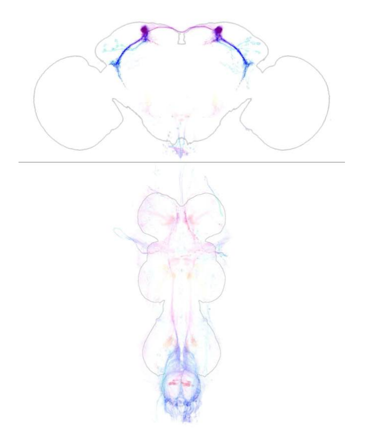
MB206B

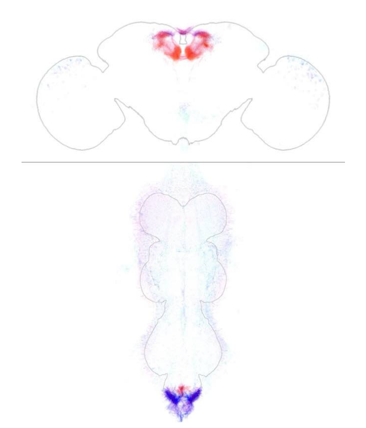
MB210B

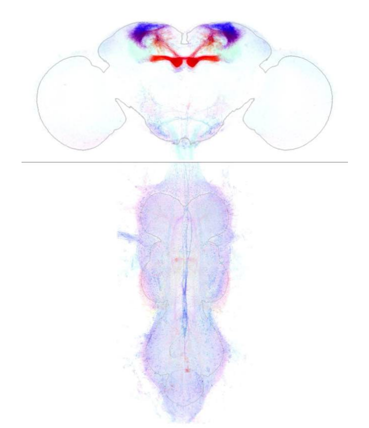
MB213B

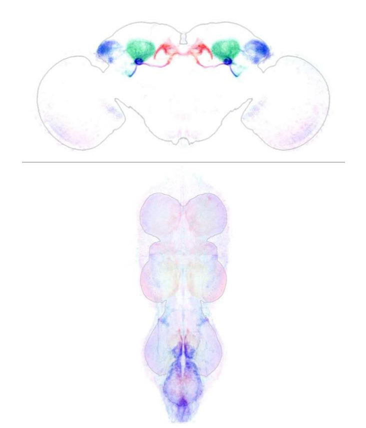
MB242A

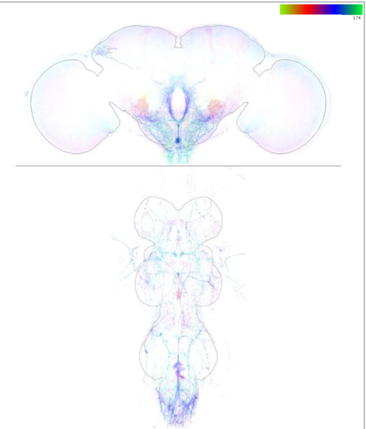
MB246A

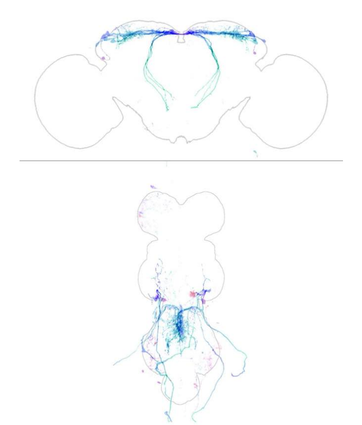
MB252C

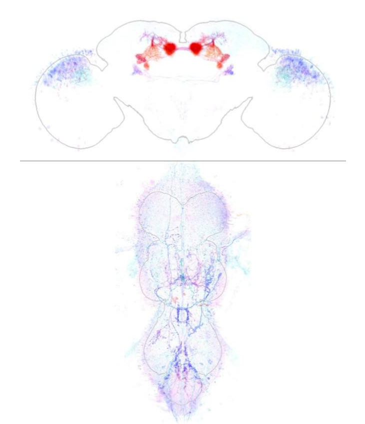
MB298B

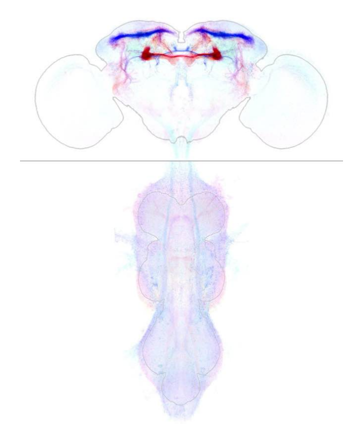
MB299B

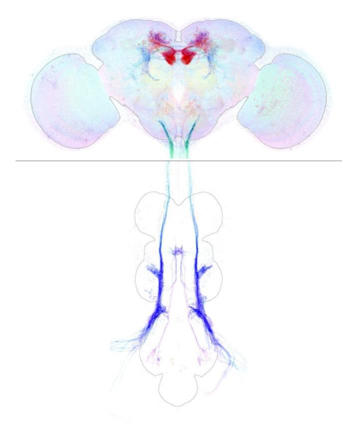
MB301B

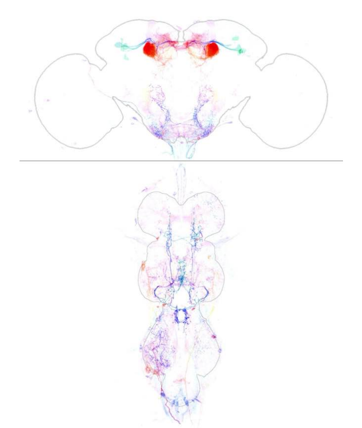
MB302B

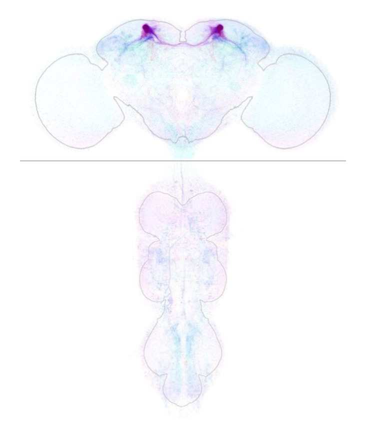
MB304B

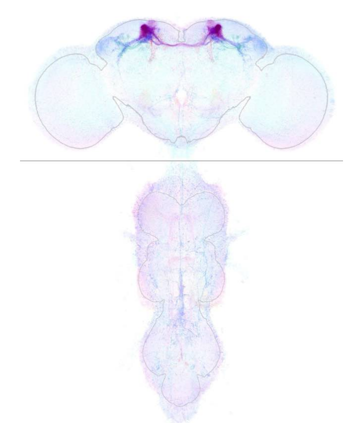
MB308B

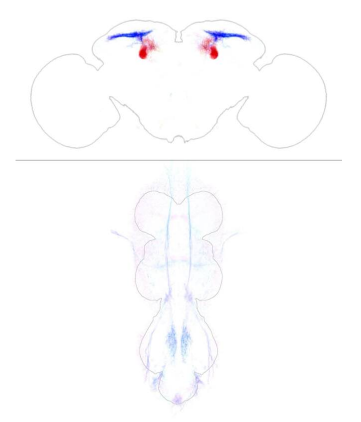
MB310C

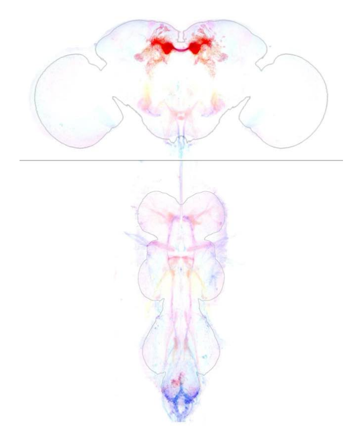
MB312C

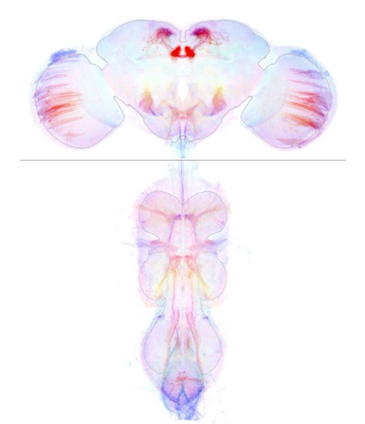
MB313C

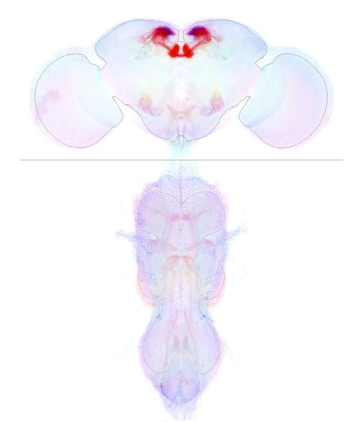
MB315C

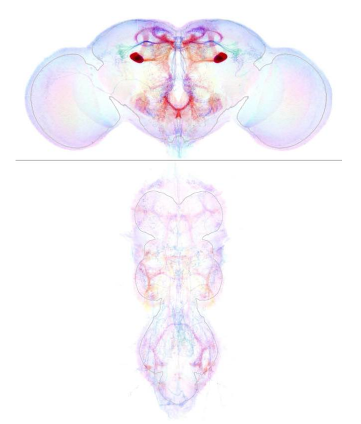
MB320C

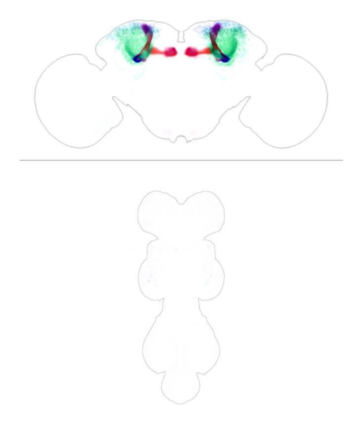
MB370B

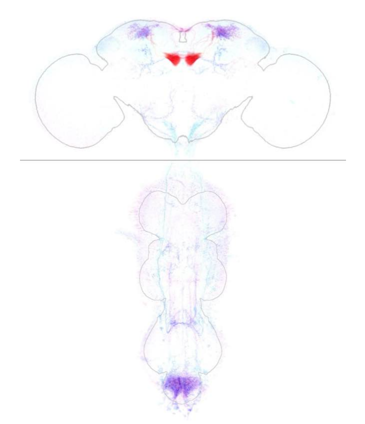
MB399B

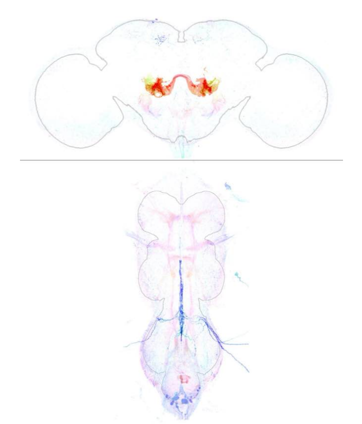
MB408B

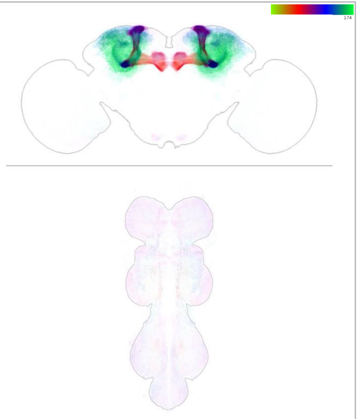
MB417B

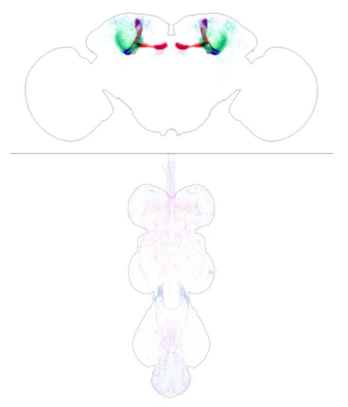
MB418B

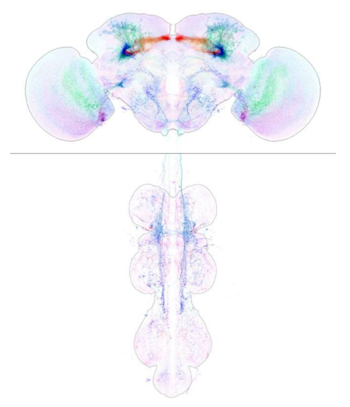
MB419B

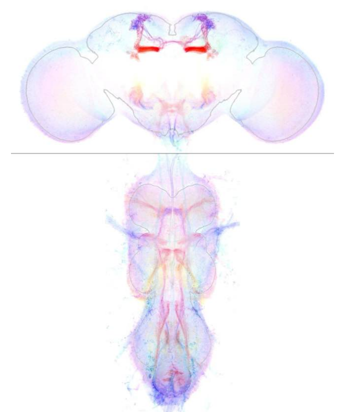
MB433B

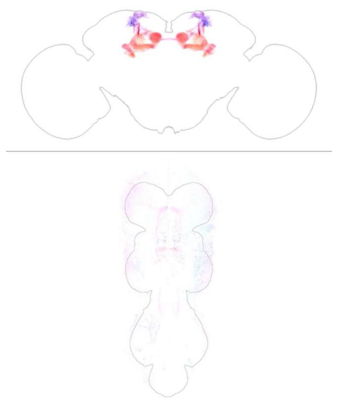
MB434B

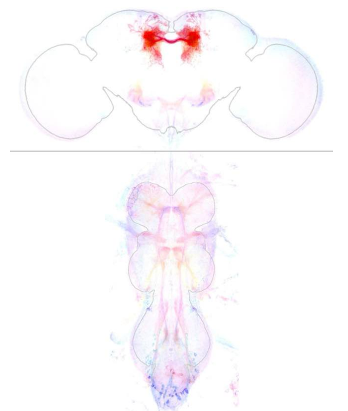
MB441B

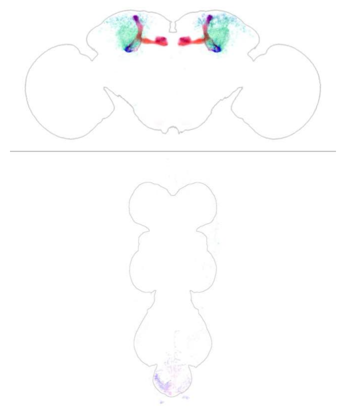
MB463B

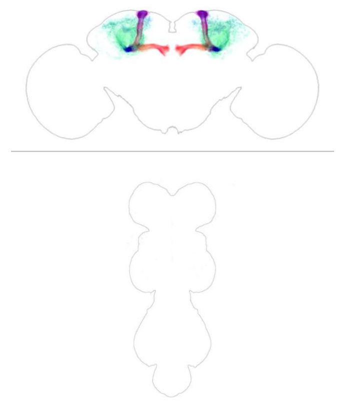
MB477B

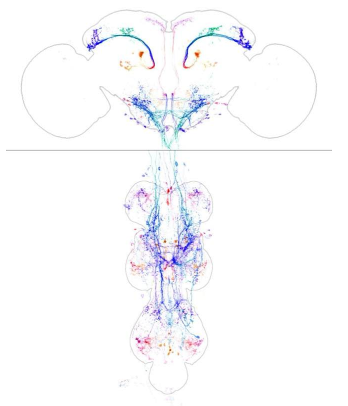
MB485B

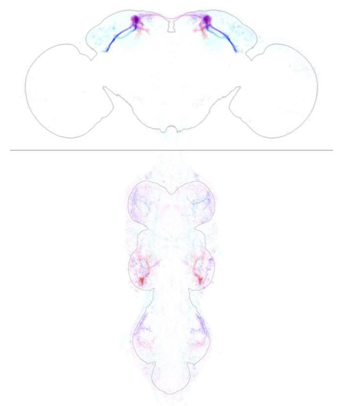
MB542B

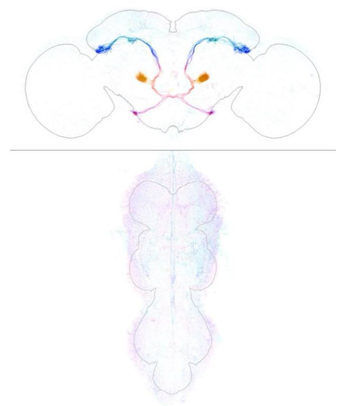
MB546B

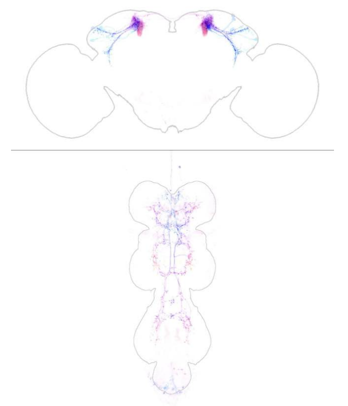
MB549B

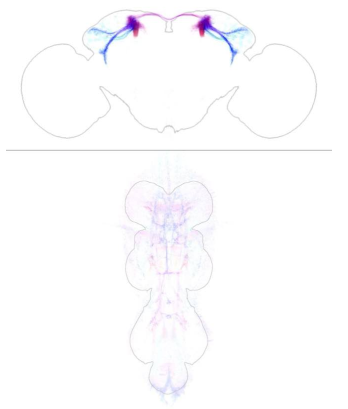
MB549C

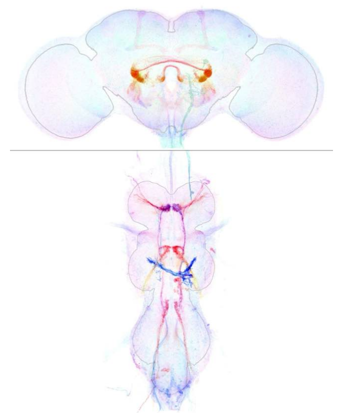
MB563B

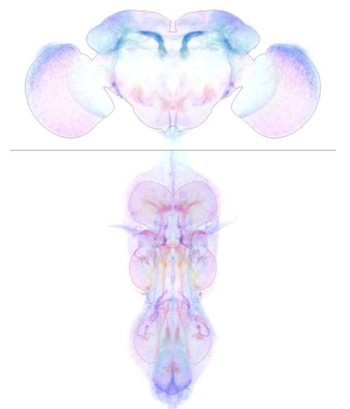
MB583B

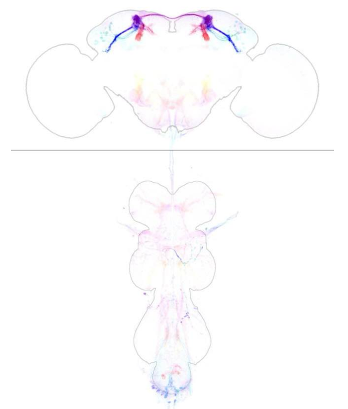
MB618B

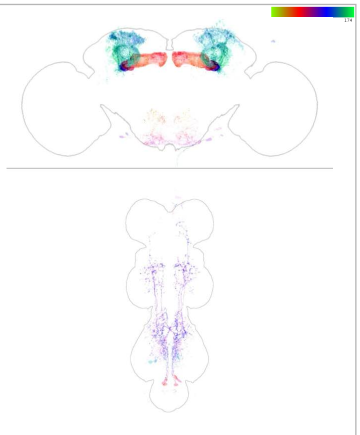

MB623C

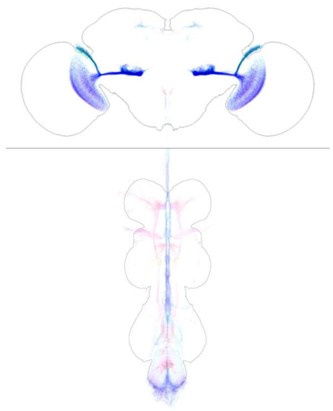

OL0001B

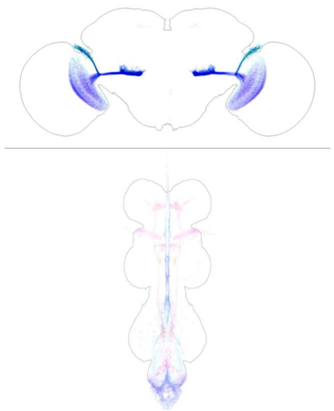

OL0002B

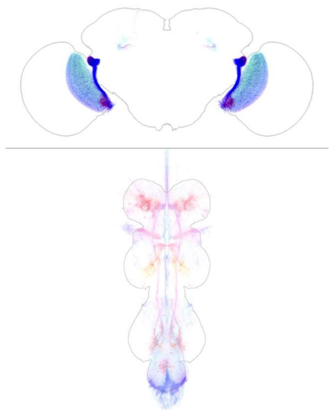

OL0003B

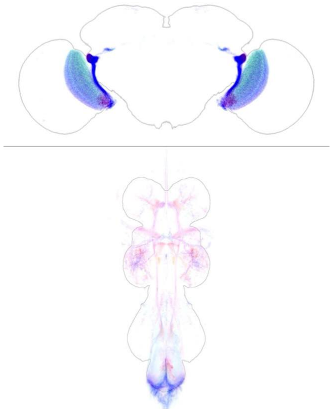

OL0004B

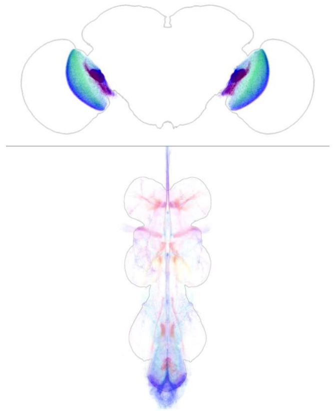

OL0005B

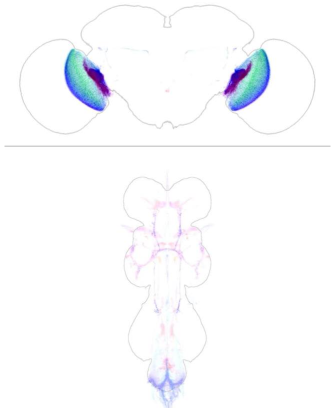

OL0006B

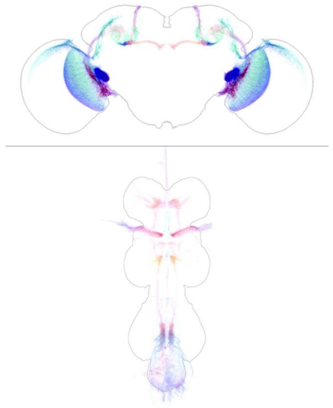

OL0007B

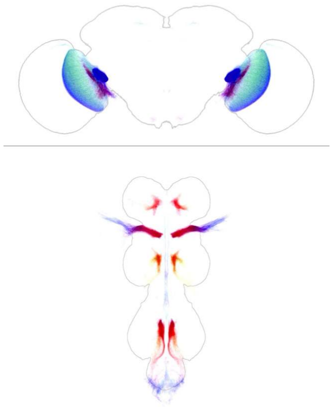

OL0008B

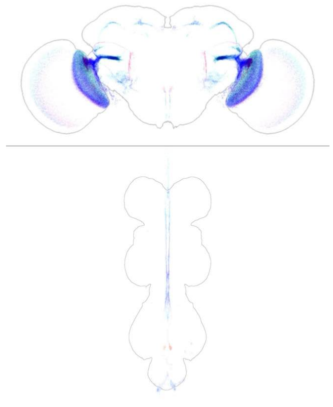

OL0009B

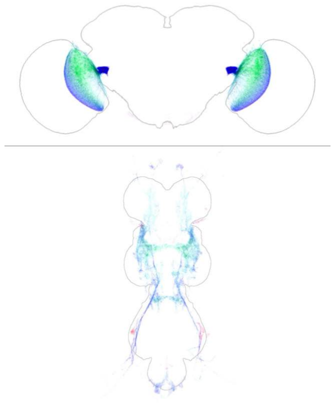

OL0010B

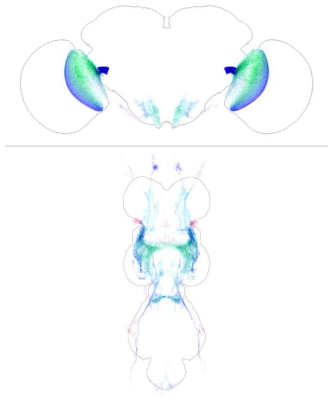

OL0011B

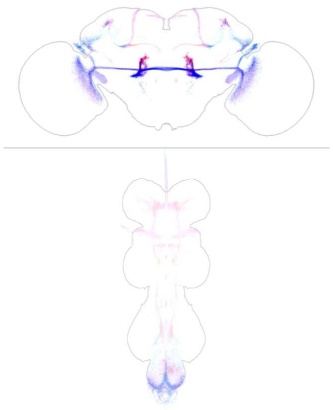

OL0012B

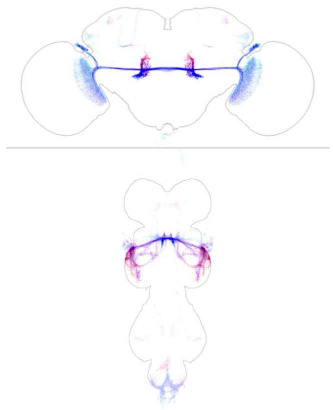

OL0014B

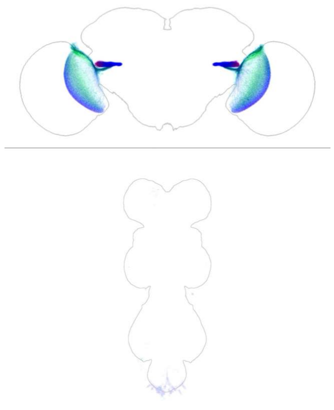

OL0015B

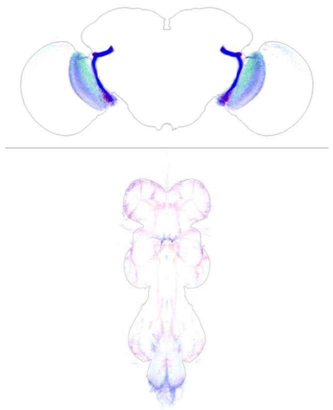

OL0017B

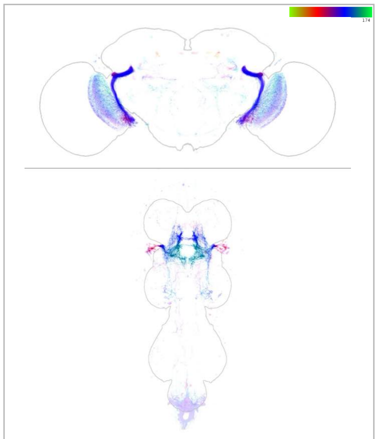
OL0018B

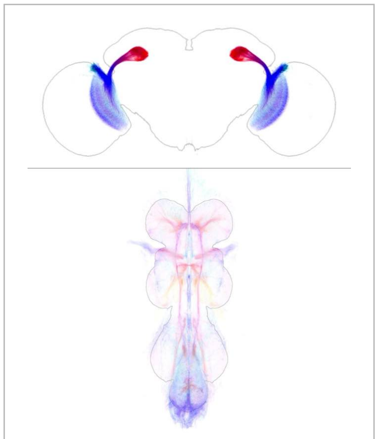
OL0019B

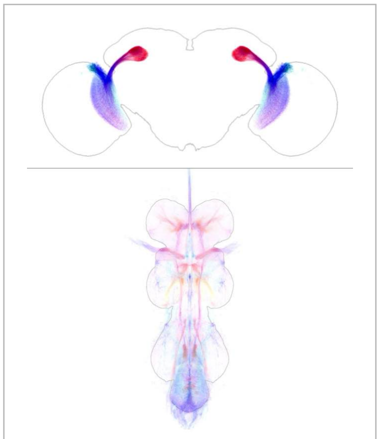
OL0020B

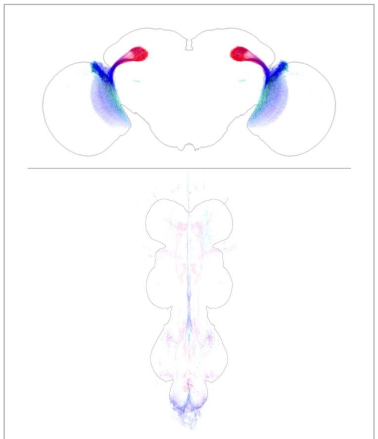
OL0021B

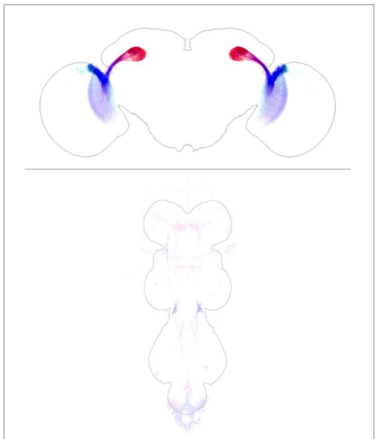
OL0022B

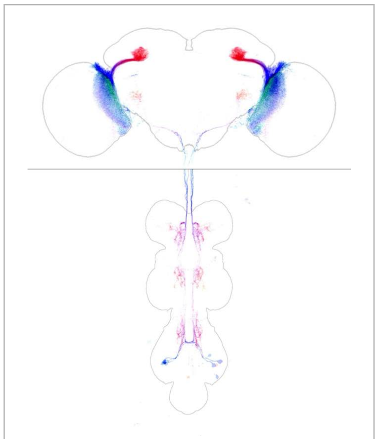
OL0023B

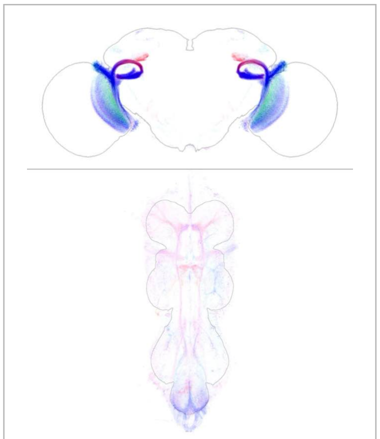
OL0024B

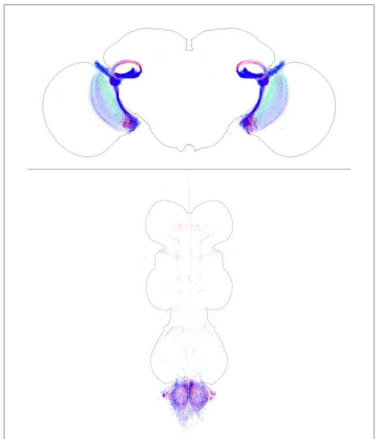
OL0026B

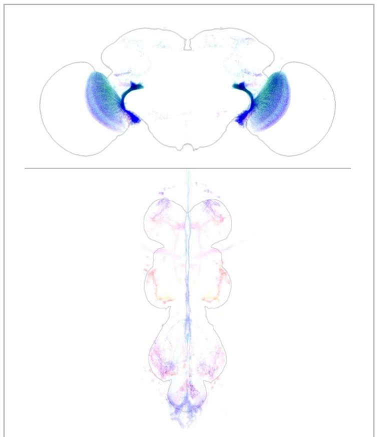
OL0027B

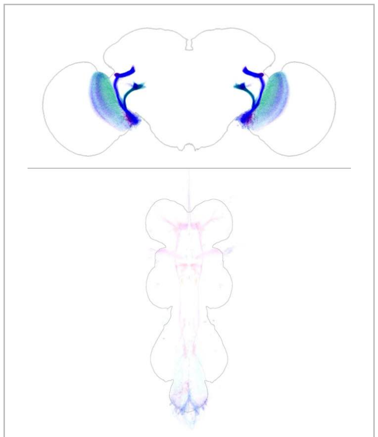
OL0028B

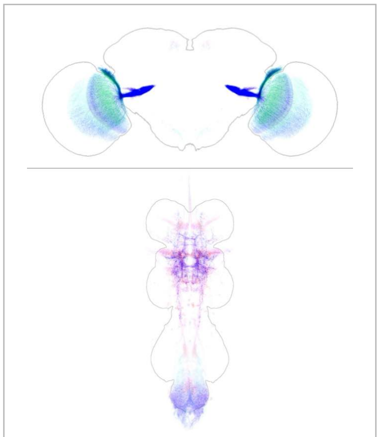
OL0029B

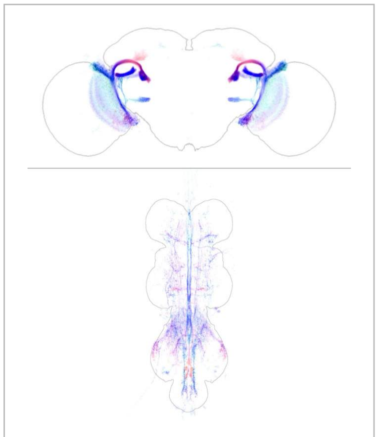
OL0030B

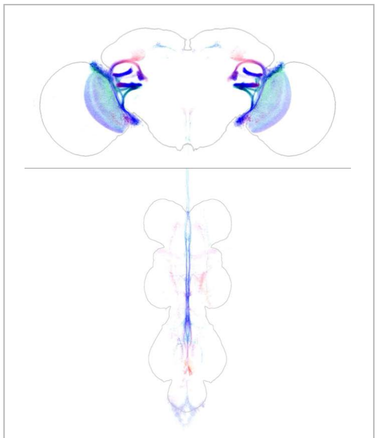
OL0031B

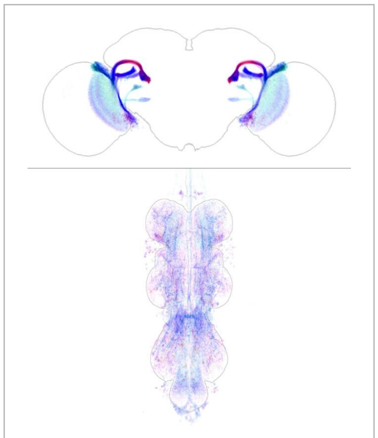
OL0035B

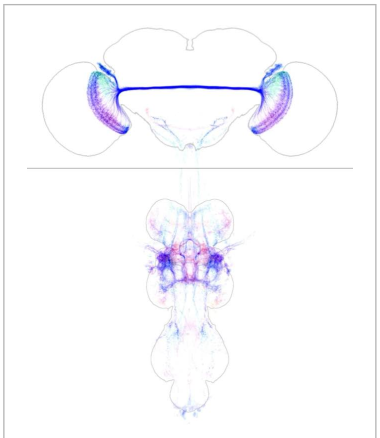
OL0037B

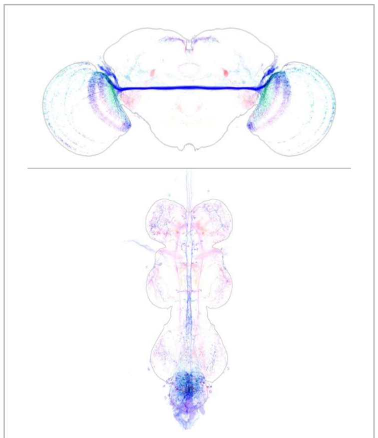
OL0038B

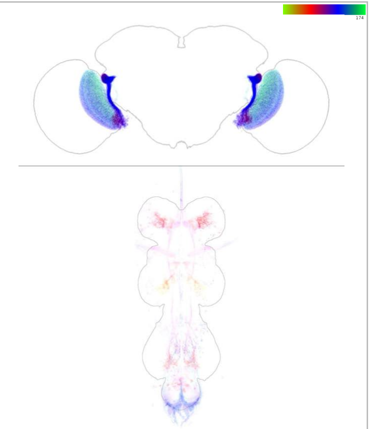
OL0042B

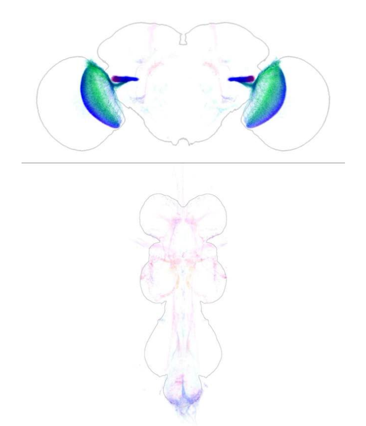
OL0043B

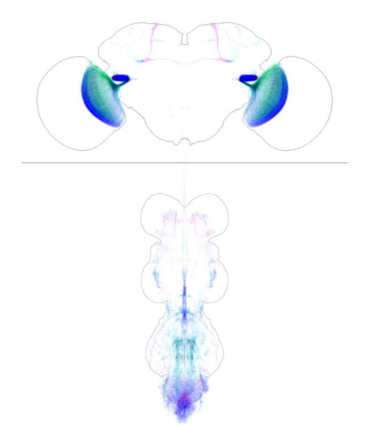
OL0044B

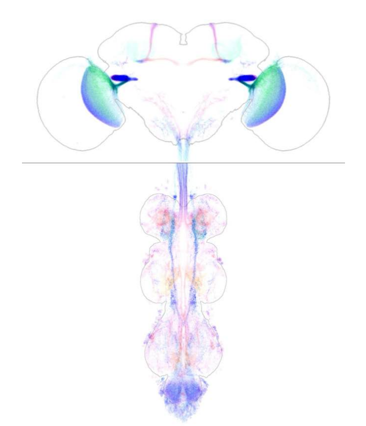
OL0045B

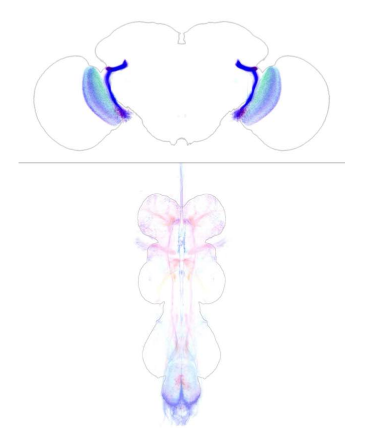
OL0046B

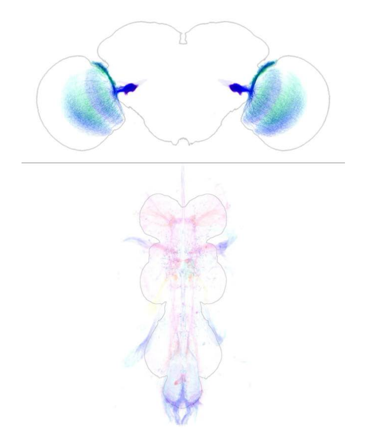
OL0047B

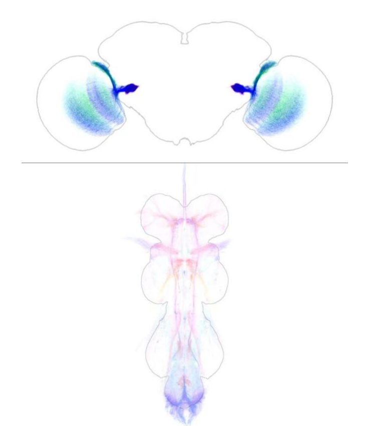
OL0048B

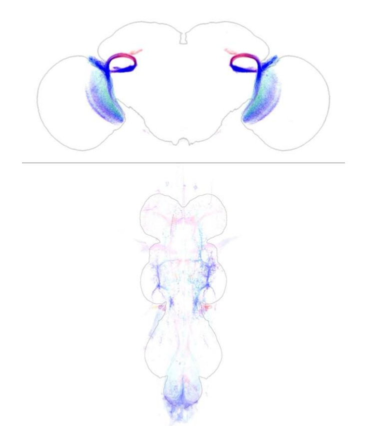
OL0070B

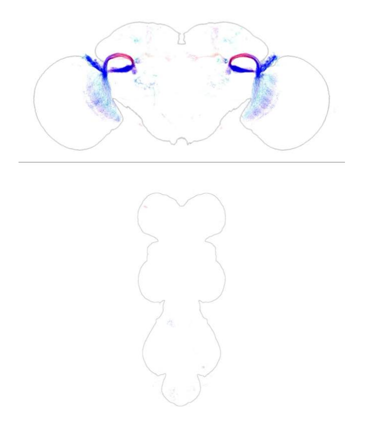
OL0071B

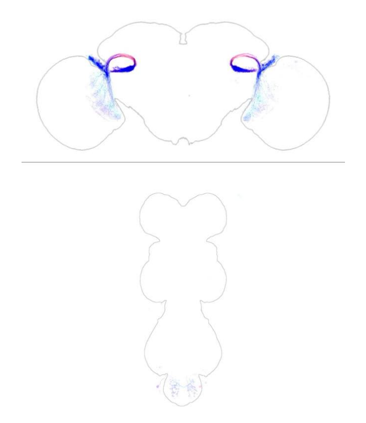
OL0077B

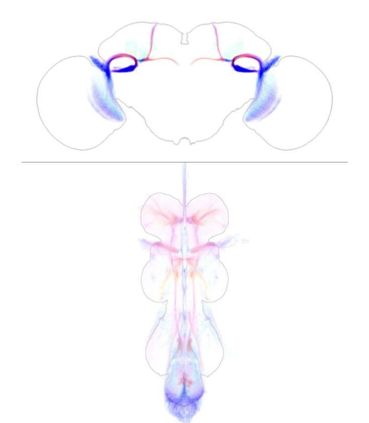
OL0081B

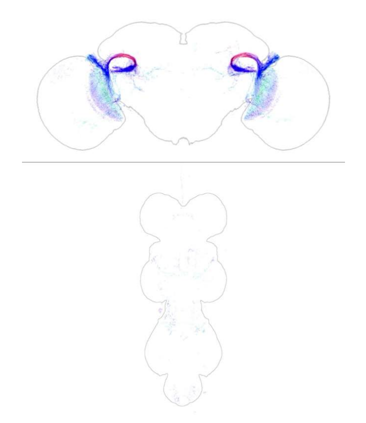
OL0087B

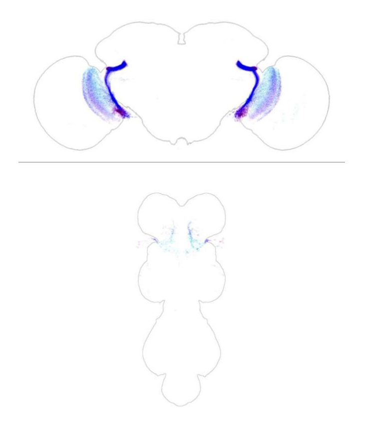
OL0092C

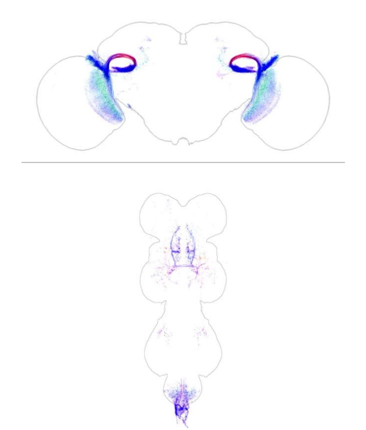
OL0202B

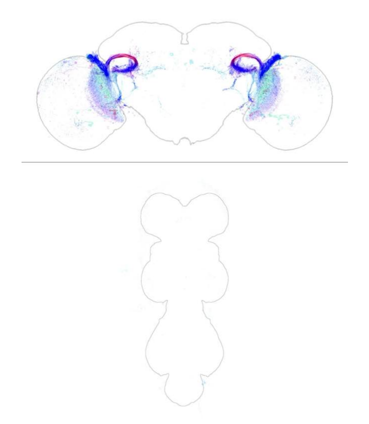
OL0203B

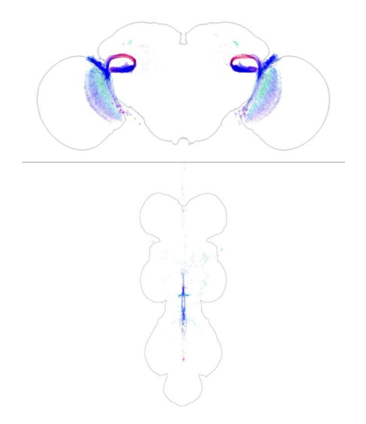
OL0218B

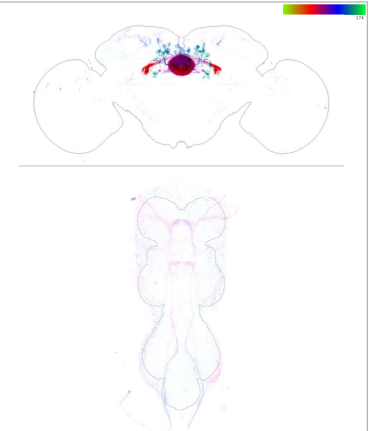
SS00026

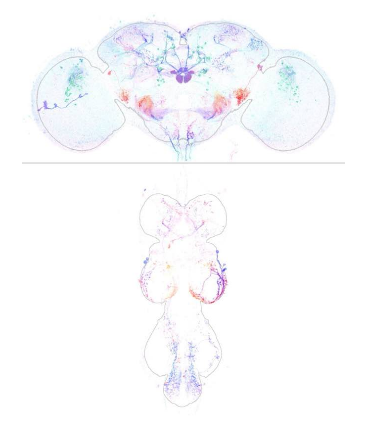
SS00029

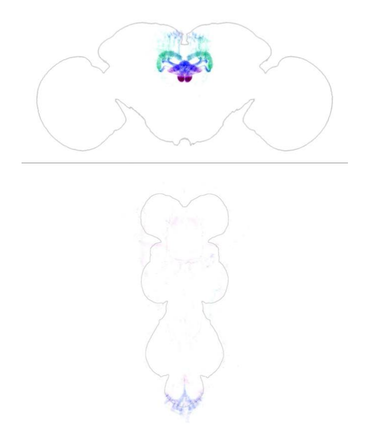
SS00043

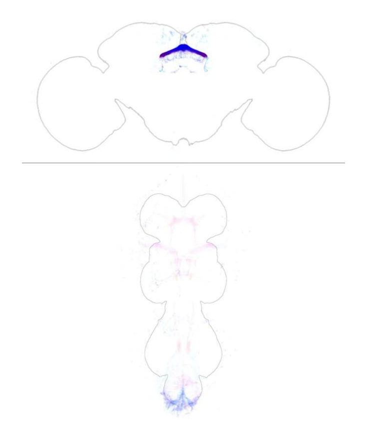
SS00045

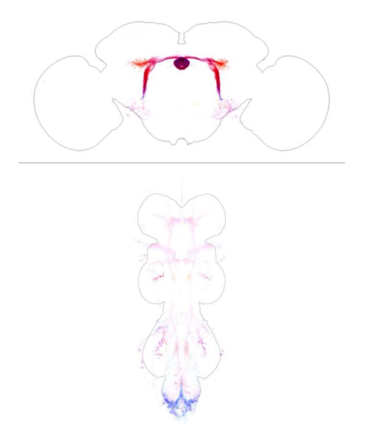
SS00057

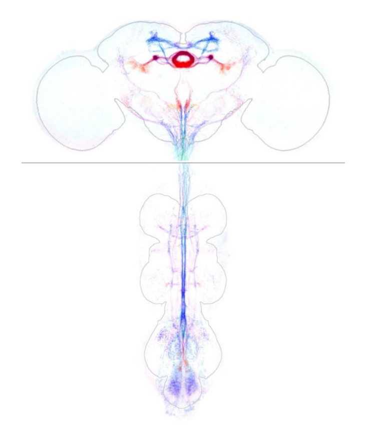
SS00070

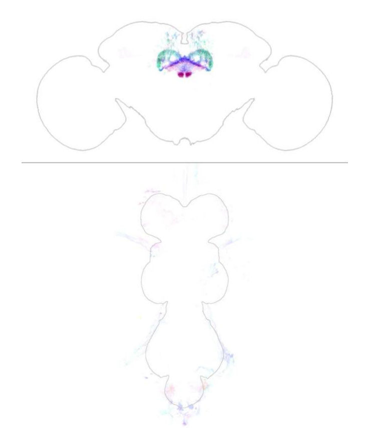
SS00078

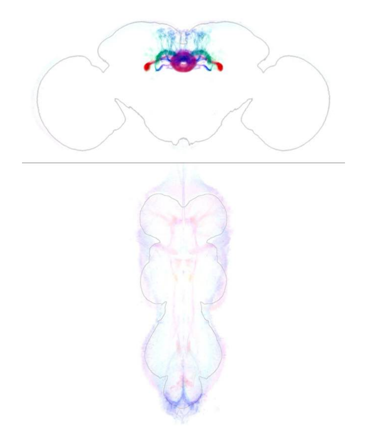
SS00090

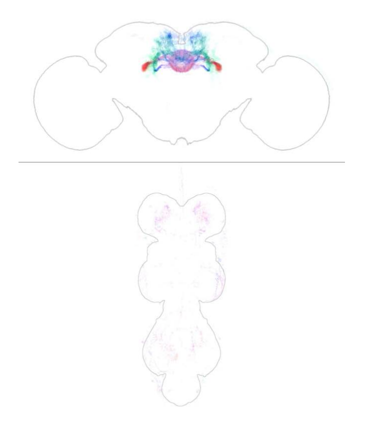
SS00098

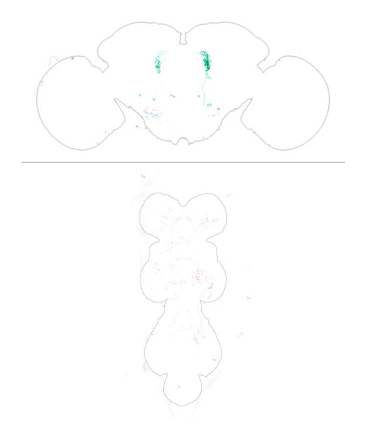
SS00118

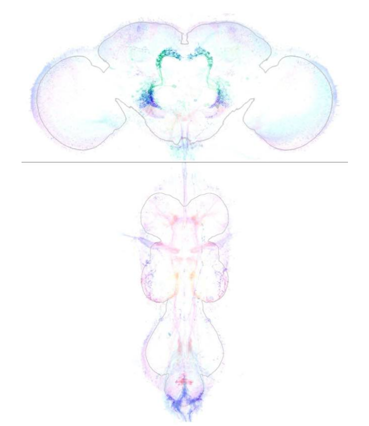
SS00153

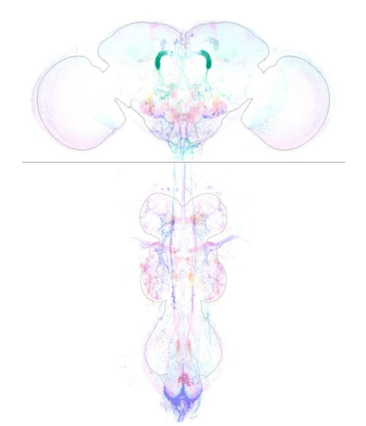
SS00189

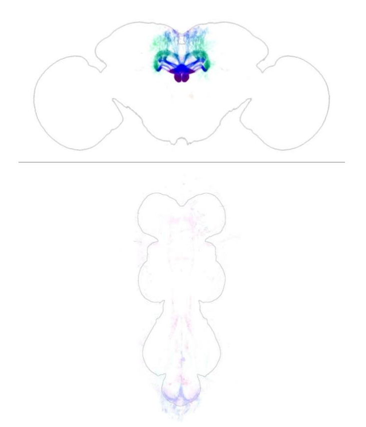
SS00191

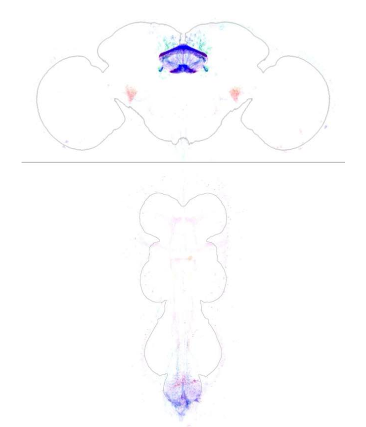
SS00266

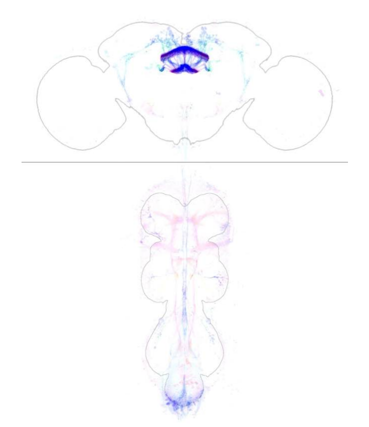
SS00268

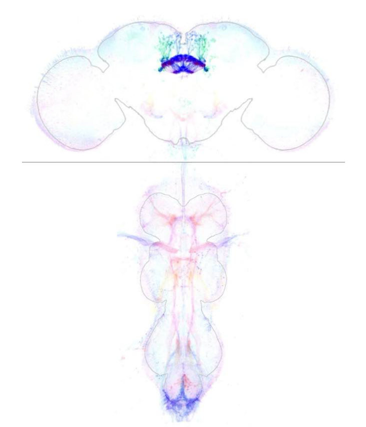
SS00281

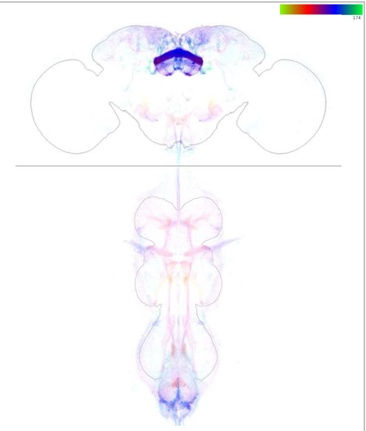

SS00291

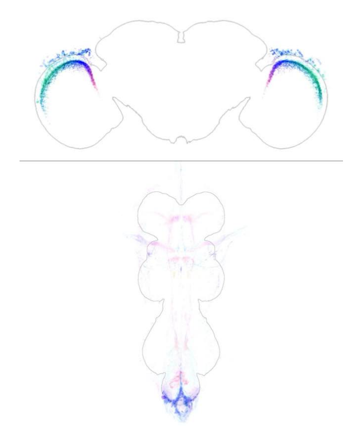

SS00298

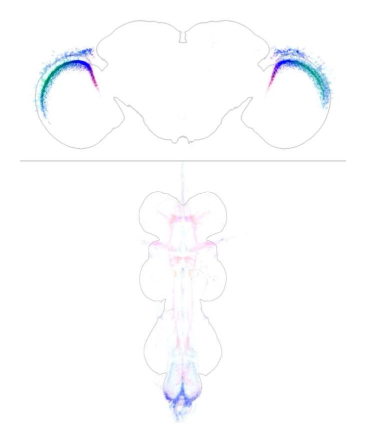

SS00299

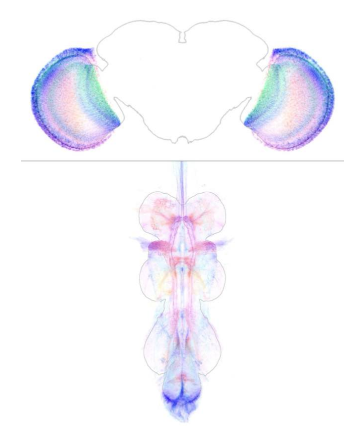

SS00300

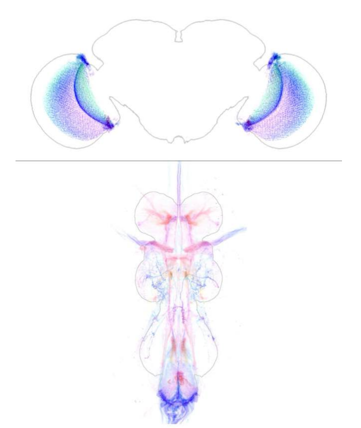

SS00301

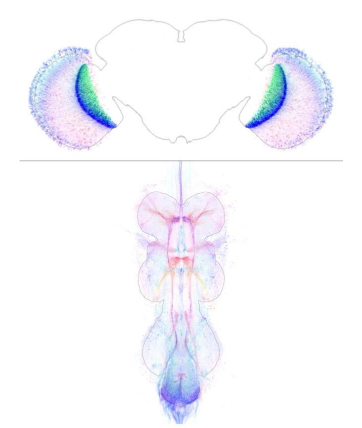

SS00307

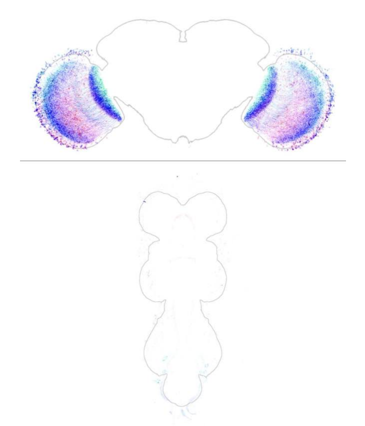

SS00308

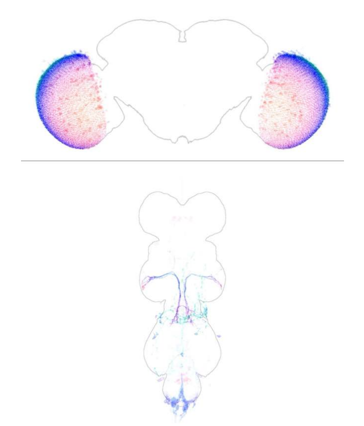

SS00309

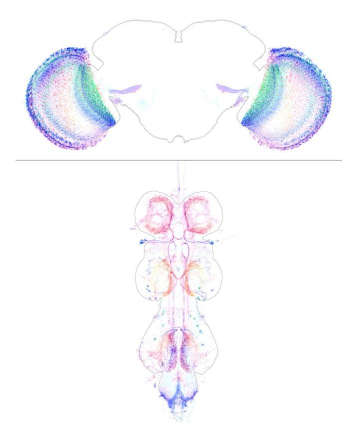

SS00313

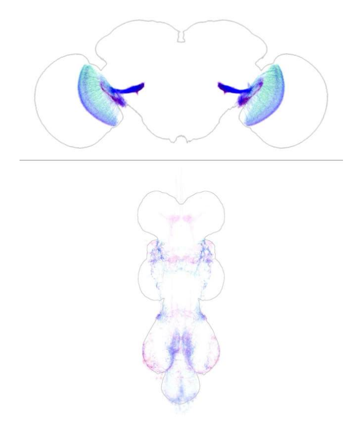

SS00315

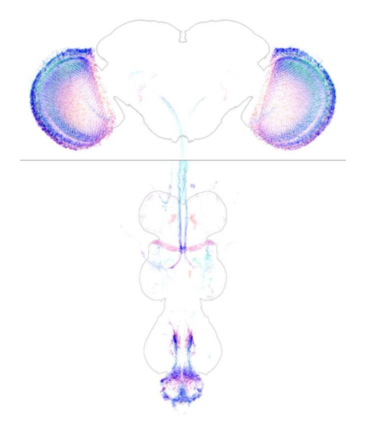

SS00316

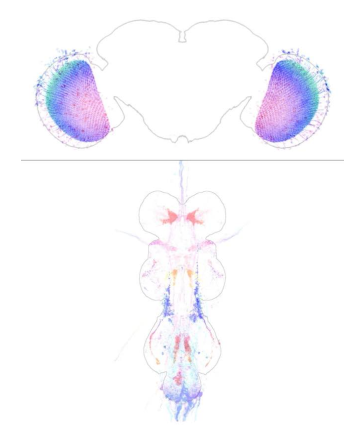

SS00317

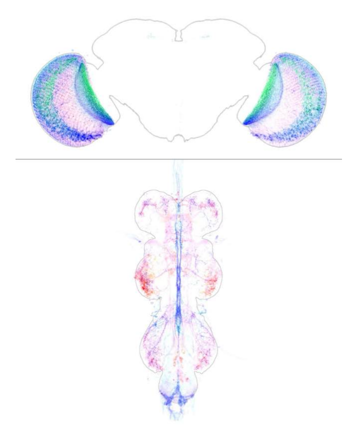

SS00318

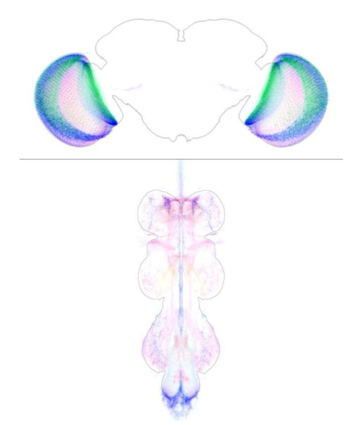

SS00319

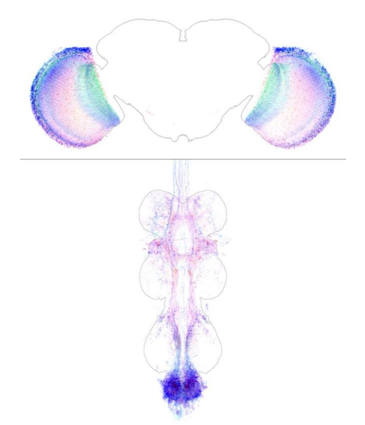

SS00320

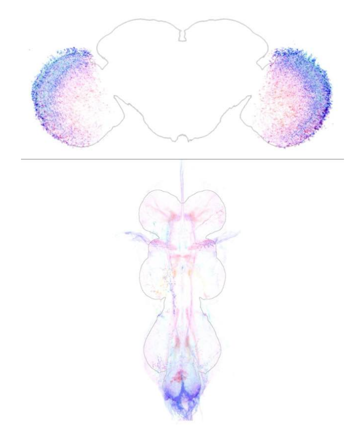

SS00321

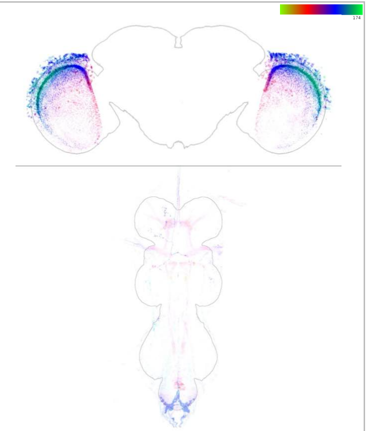
SS00322

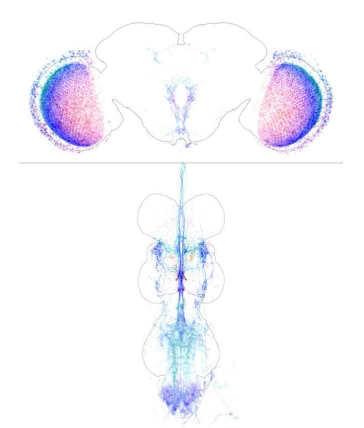
SS00323

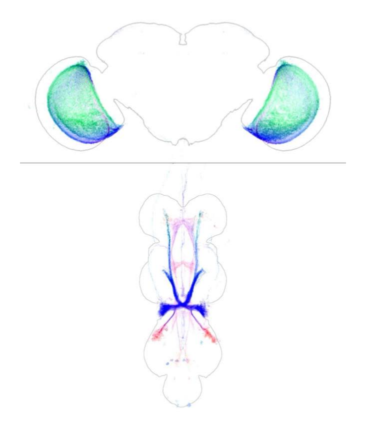
SS00324

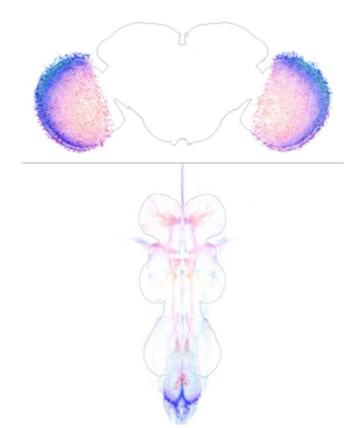
SS00327

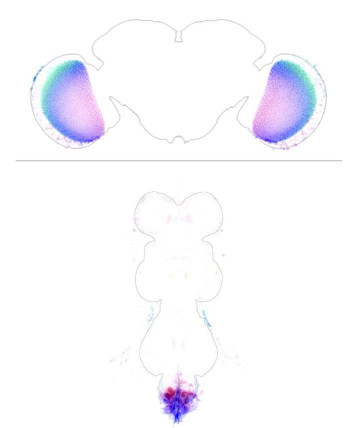
SS00328

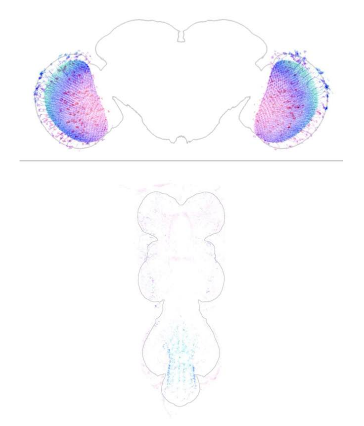
SS00330

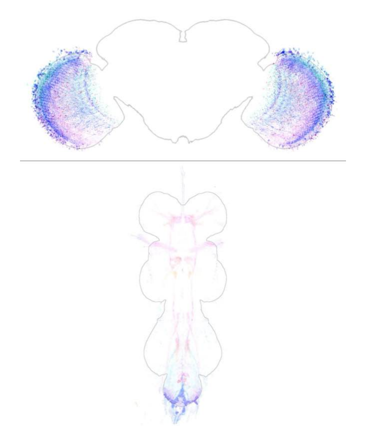
SS00331

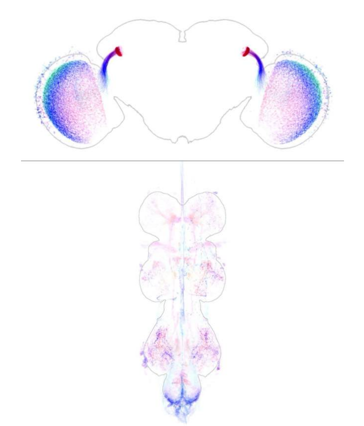
SS00332

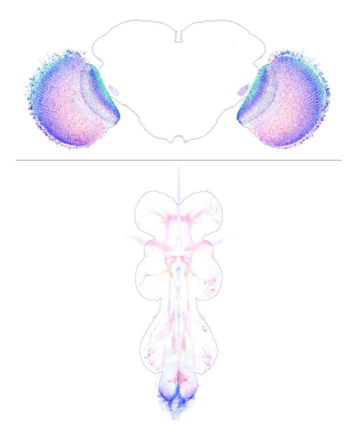
SS00335

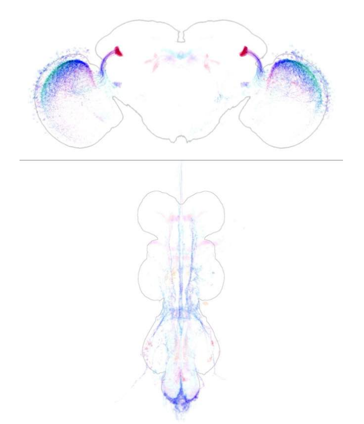
SS00336

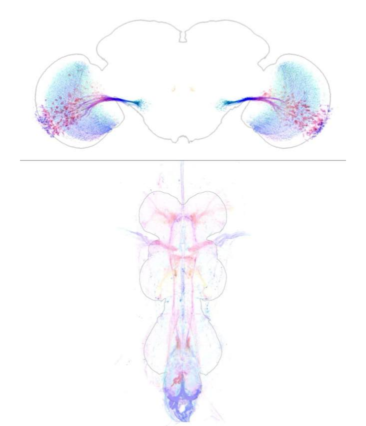
SS00340

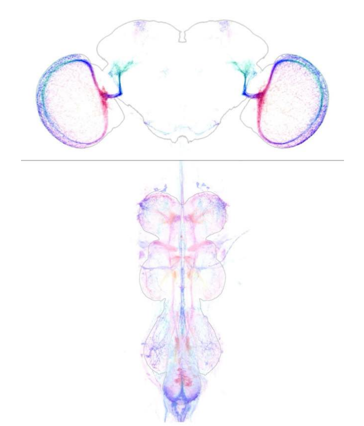
SS00342

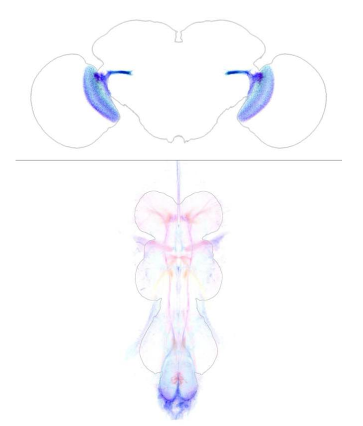
SS00343

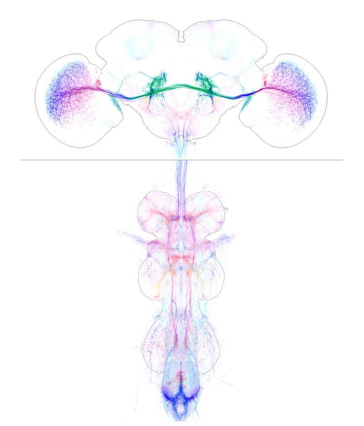
SS00346

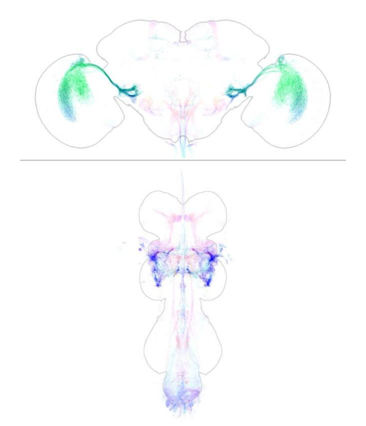
SS00347

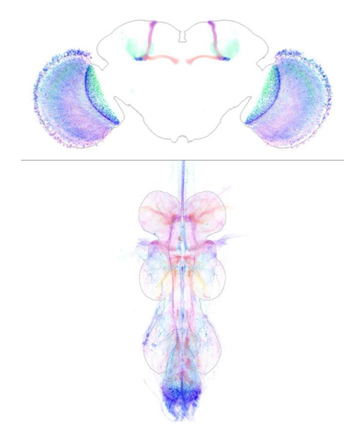
SS00349

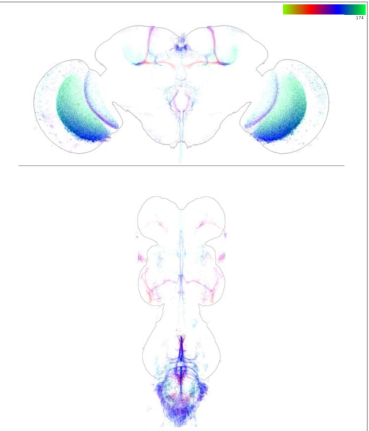
SS00353

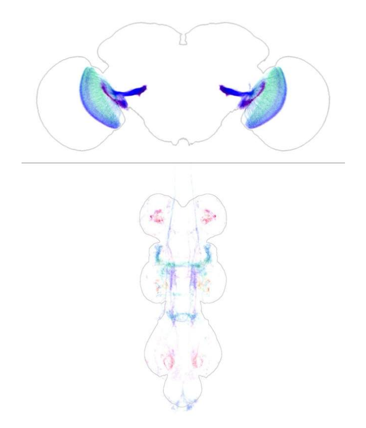
SS00356

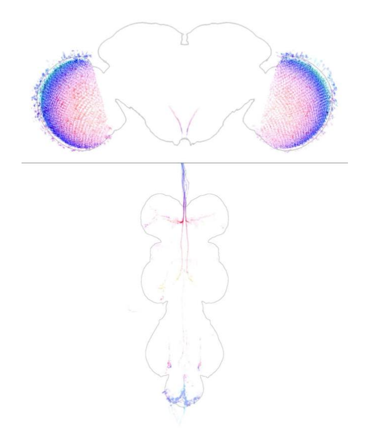
SS00359

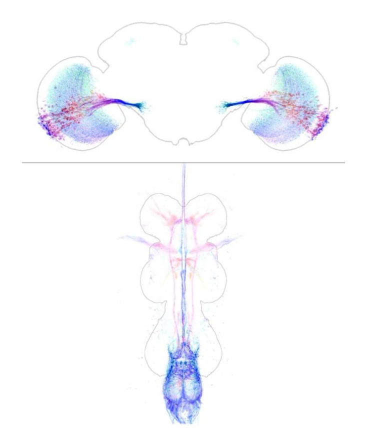
SS00360

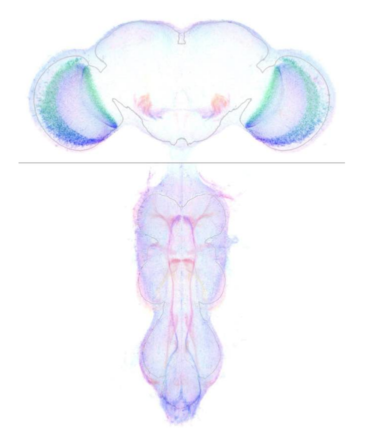
SS00362

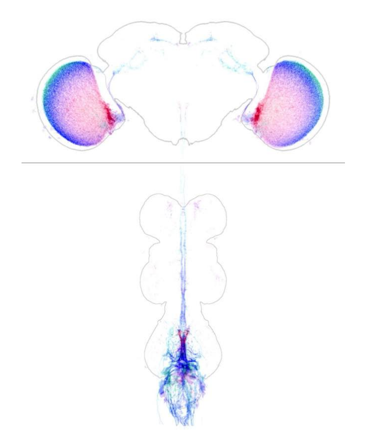
SS00363

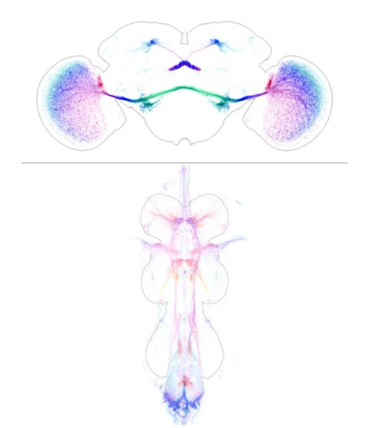
SS00365

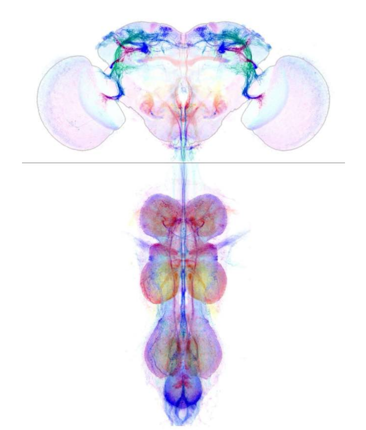
SS00367

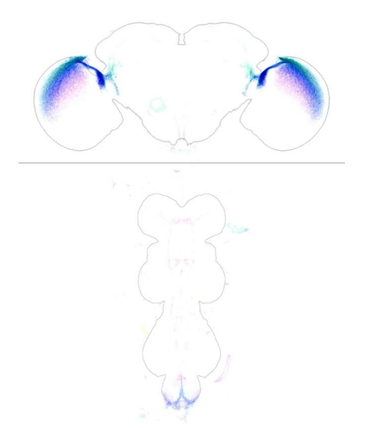
SS00368

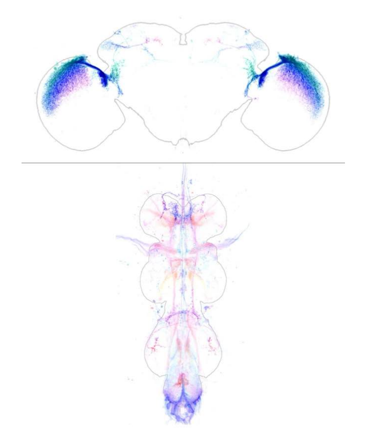
SS00369

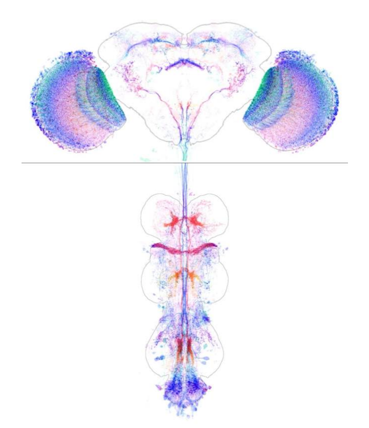
SS00370

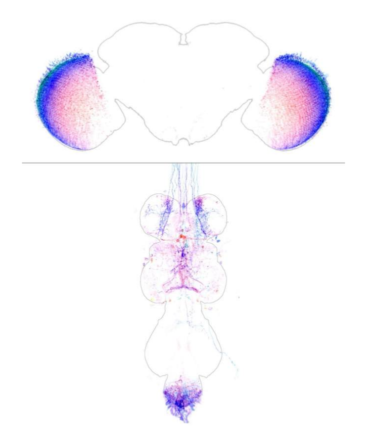
SS00373

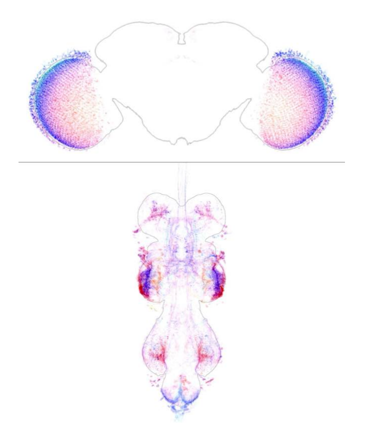
SS00374

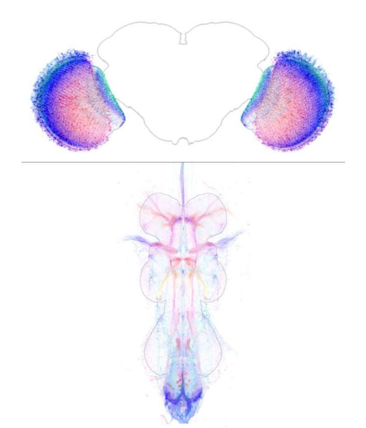
SS00375

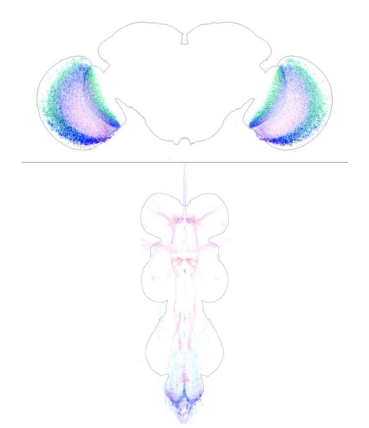
SS00382

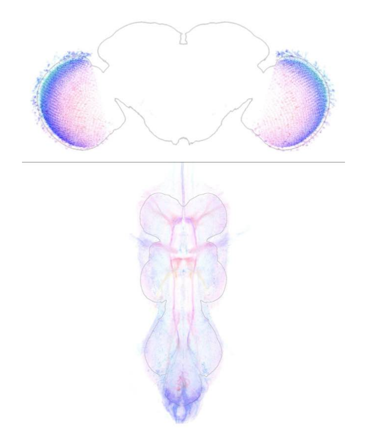
SS00384

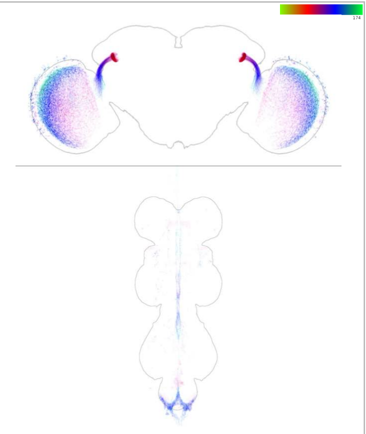

SS00385

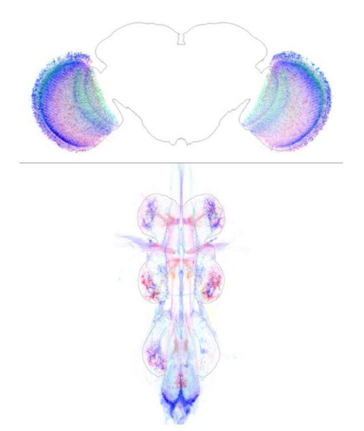

SS00395

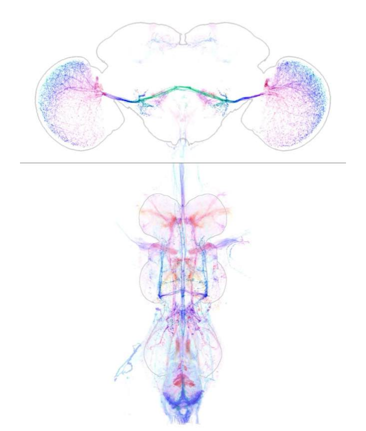

SS00399

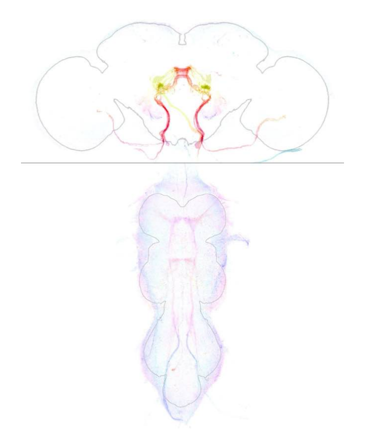

SS00445

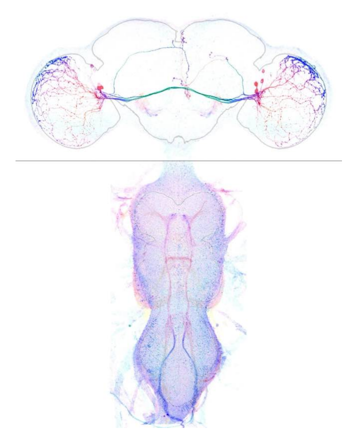

SS00447

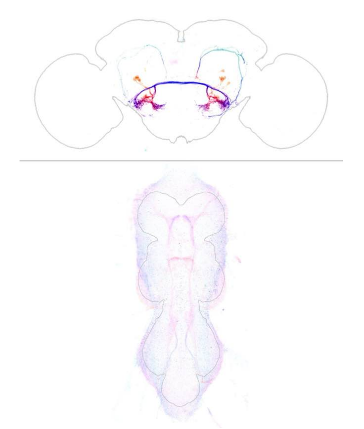

SS00455

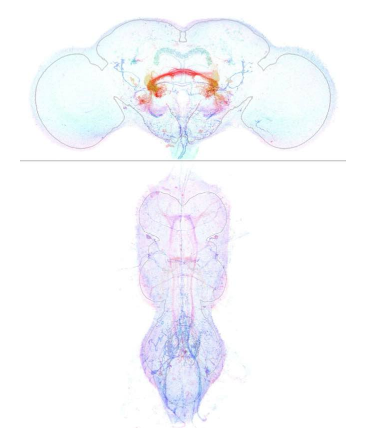

SS00459

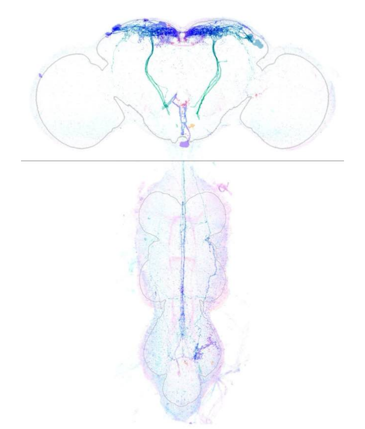

SS00470

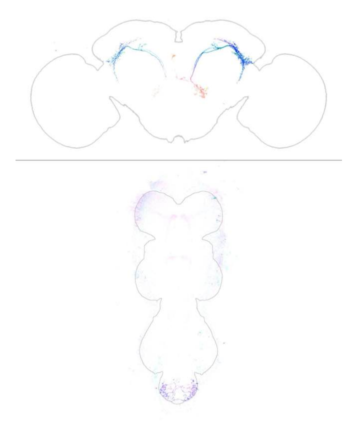

SS00486

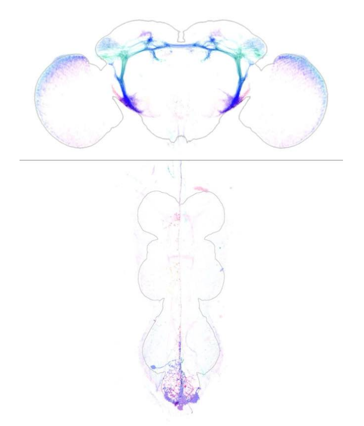

SS00502

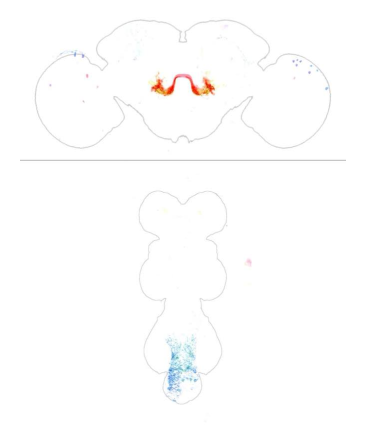

SS00503

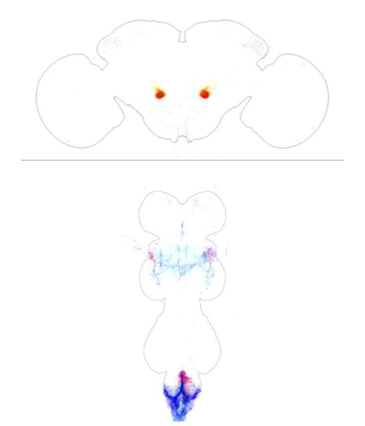

SS00525

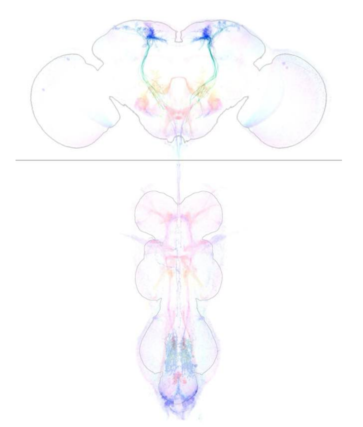

SS00532

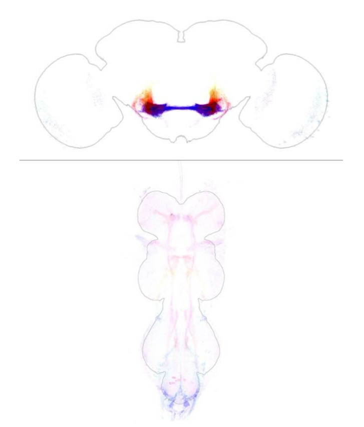

SS00550

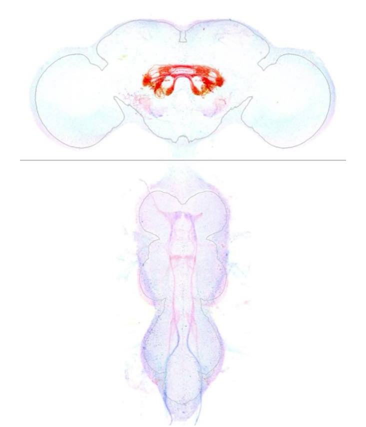

SS00560

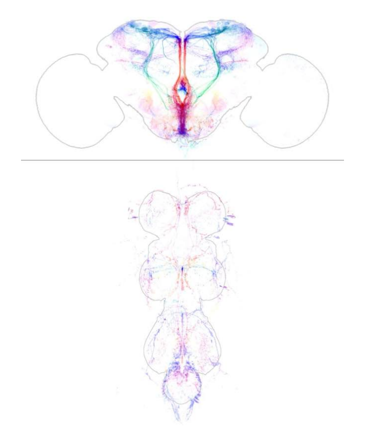

SS00589

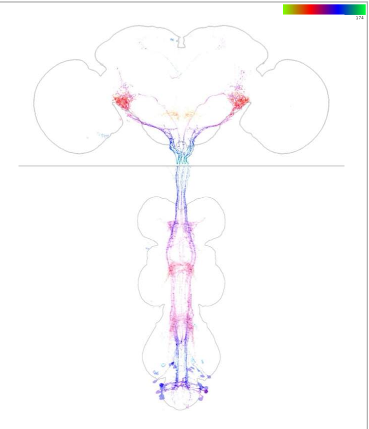
SS00603

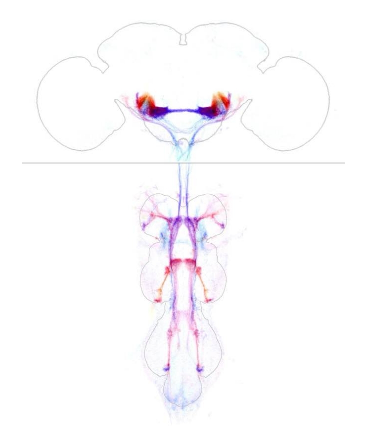
SS00612

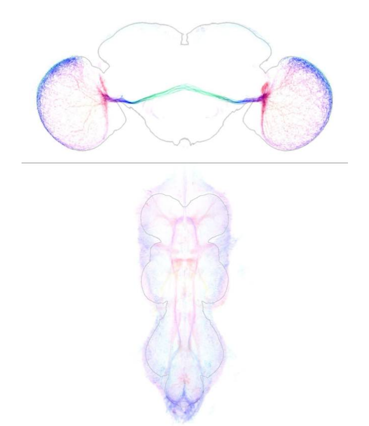
SS00645

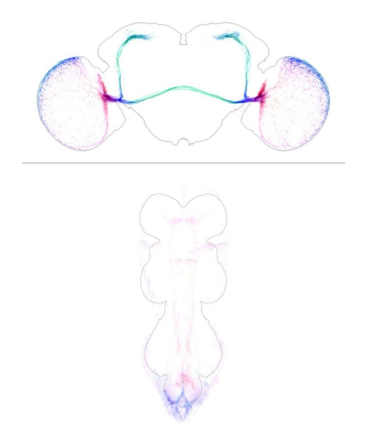
SS00651

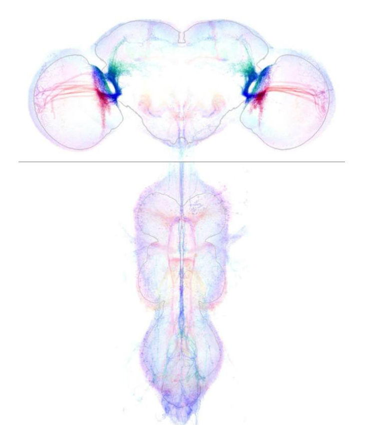
SS00657

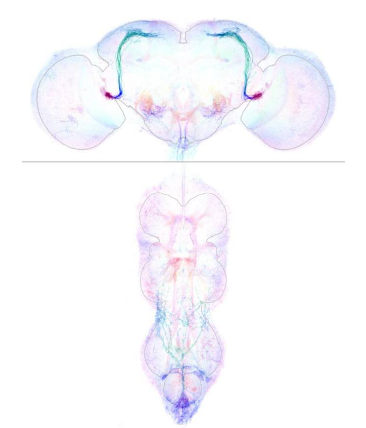
SS00681

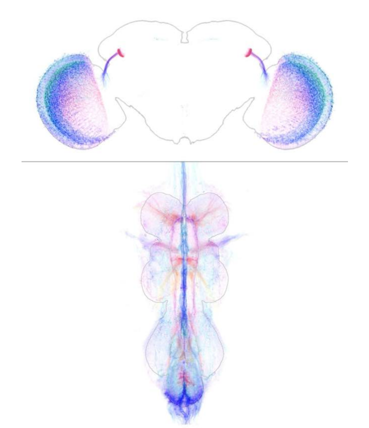
SS00688

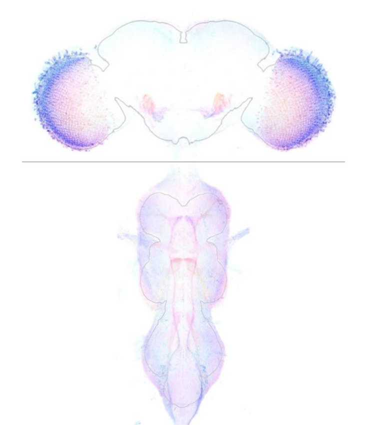
SS00689

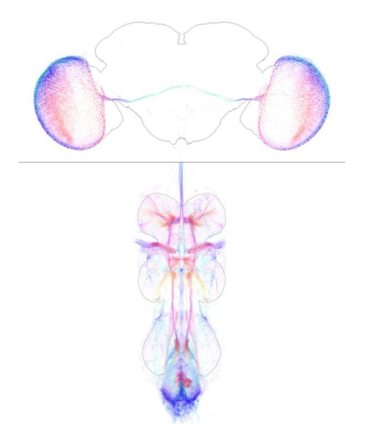
SS00690

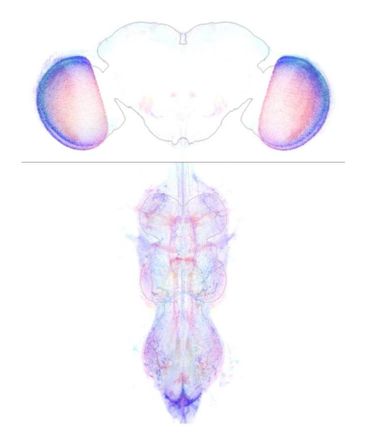
SS00692

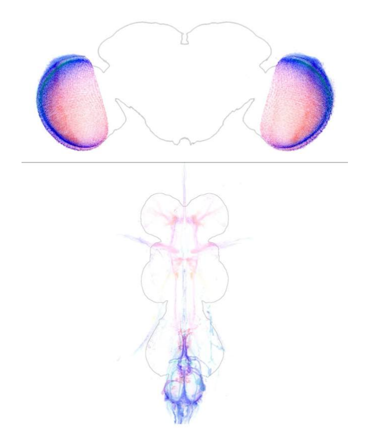
SS00695

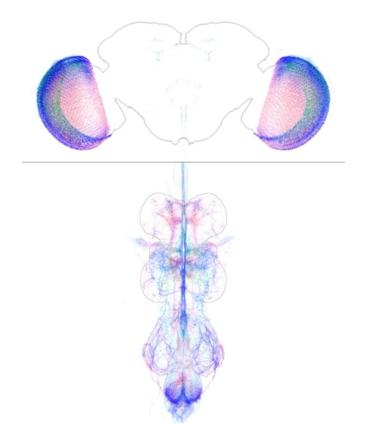
SS00696

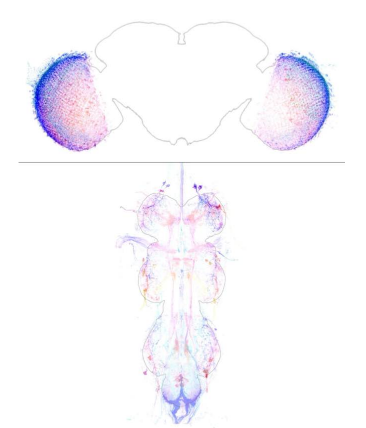
SS00698

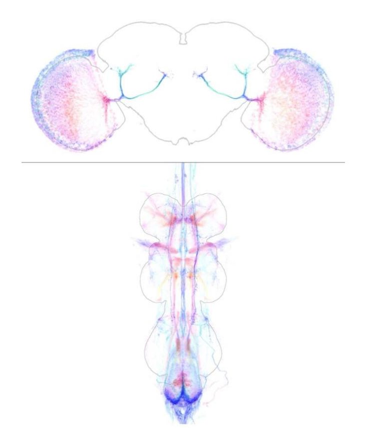
SS00700

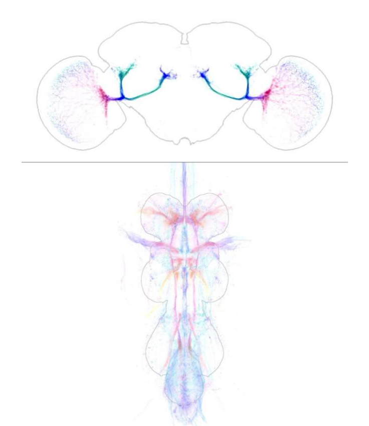
SS00705

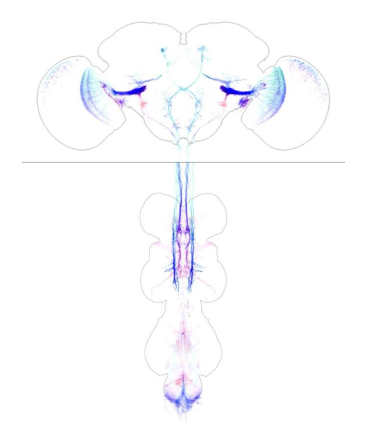
SS00724

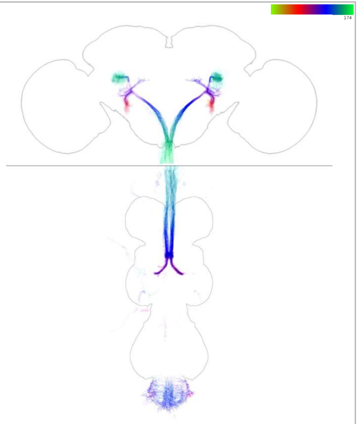

SS00727

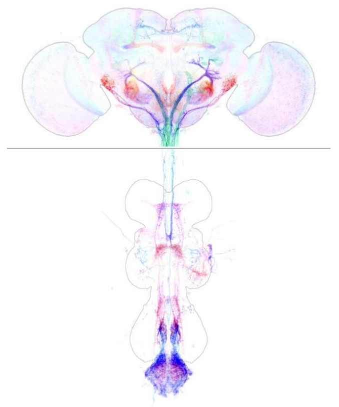

SS00728

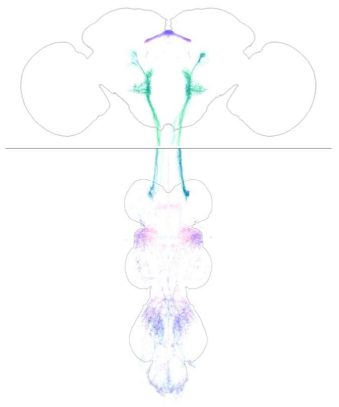

SS00729

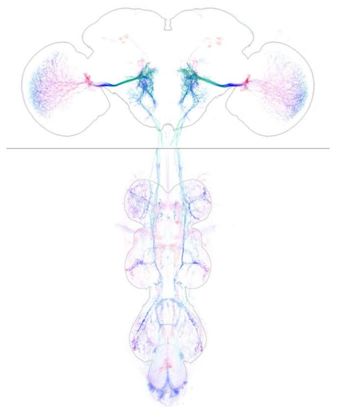

SS00730

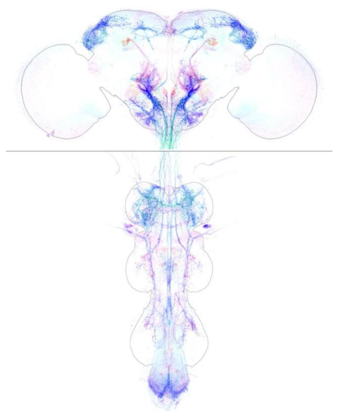

SS00731

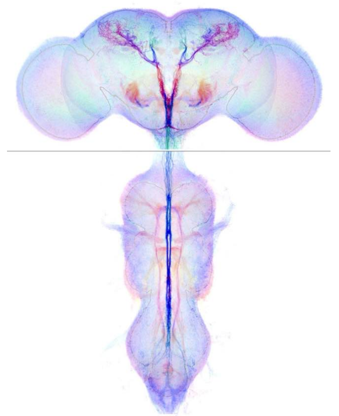

SS00732

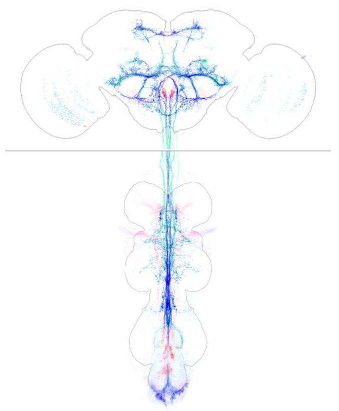

SS00733

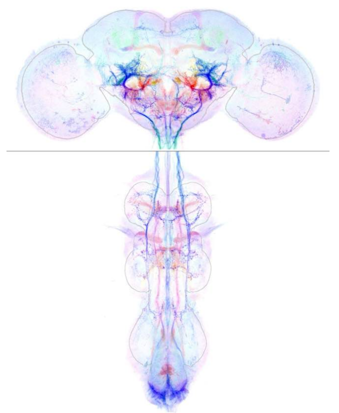

SS00734

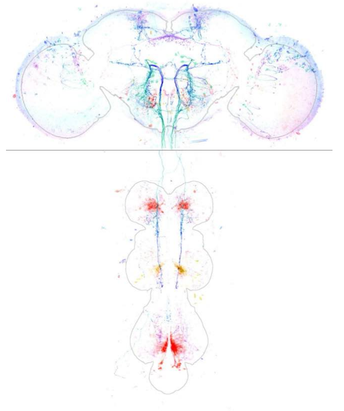

SS00735

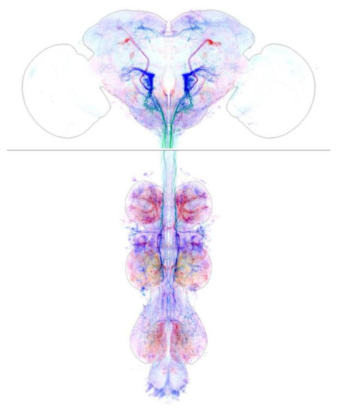

SS00736

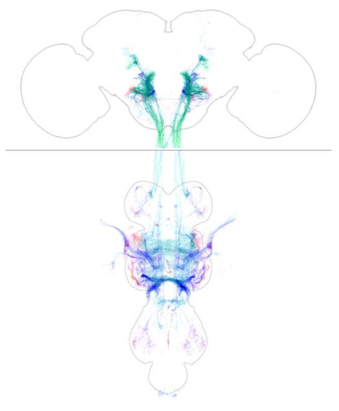

SS00737

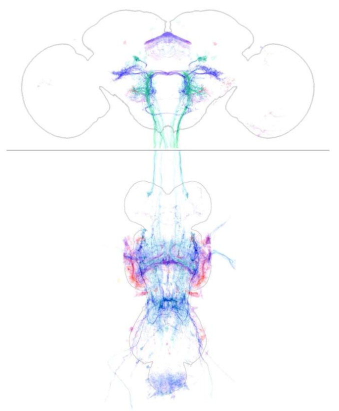

SS00738

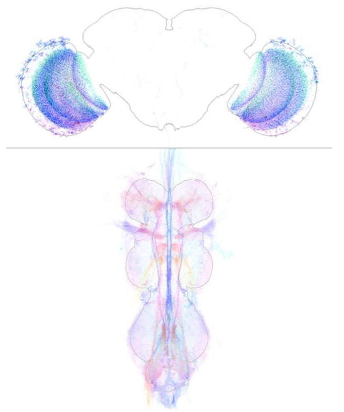

SS00764

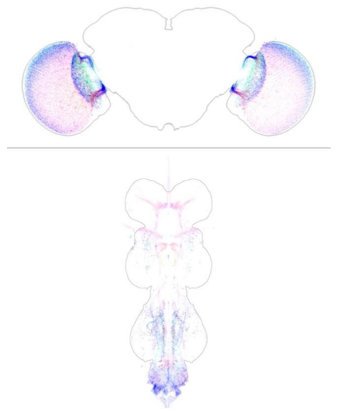

SS00769

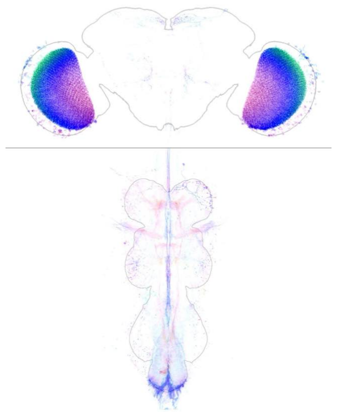

SS00770

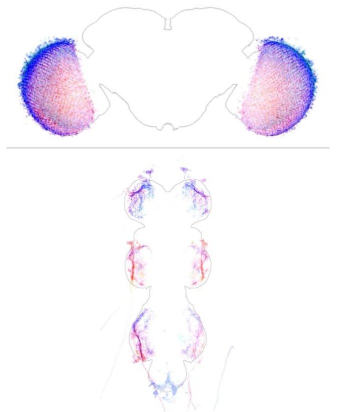

SS00771

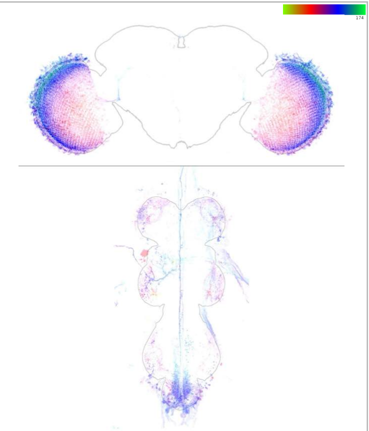

SS00772

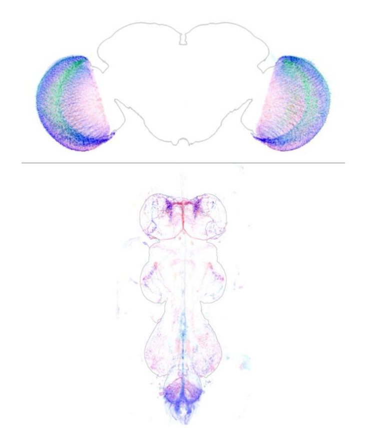

SS00778

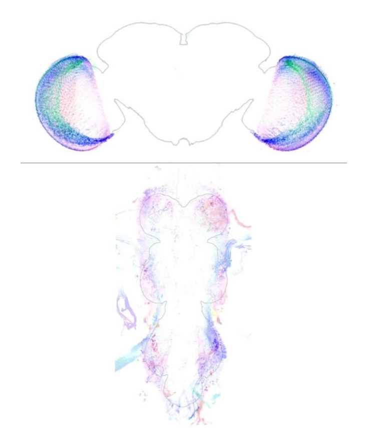

SS00779

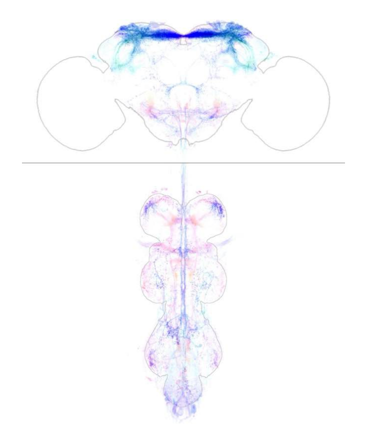

SS00781

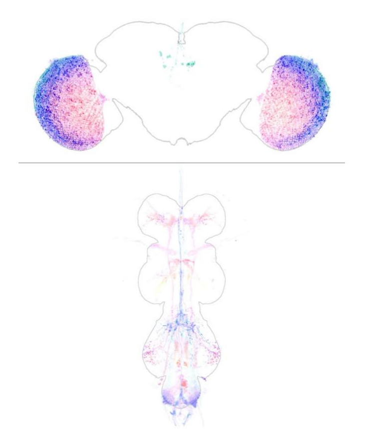

SS00782

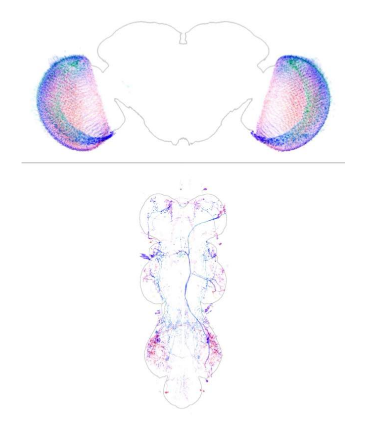

SS00783

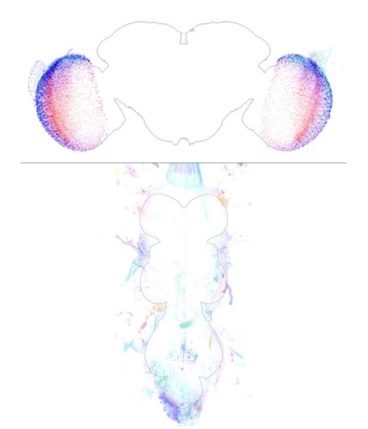

SS00789

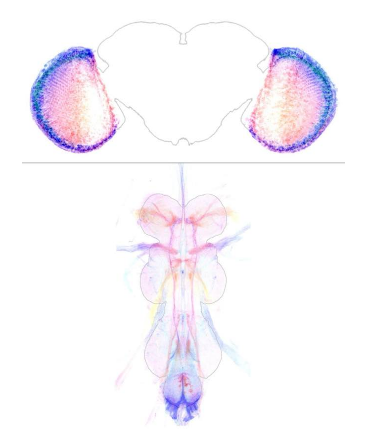

SS00790

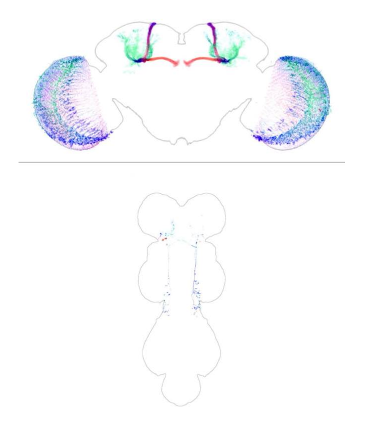

SS00792

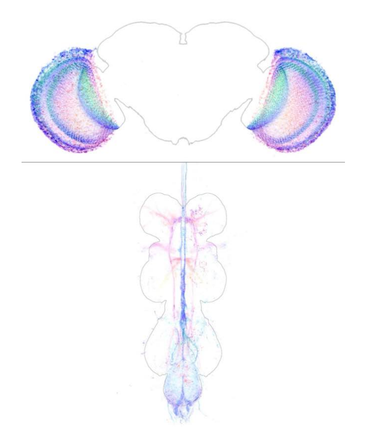

SS00796

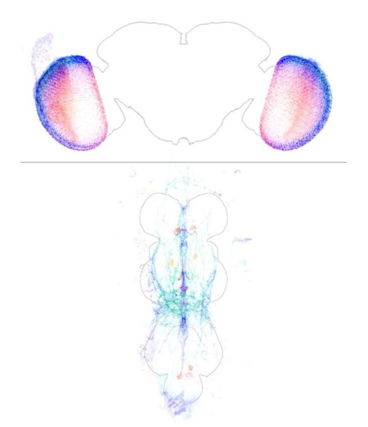

SS00797

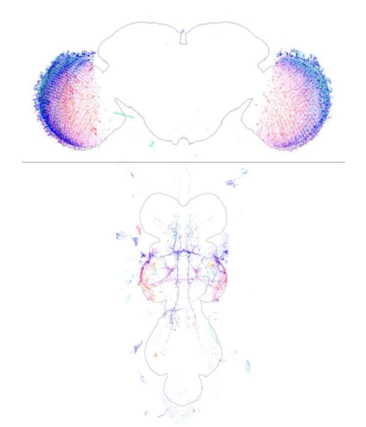

SS00800

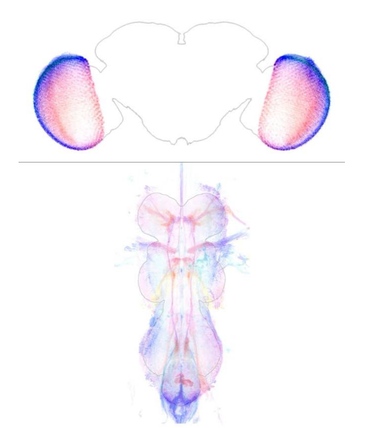

SS00801

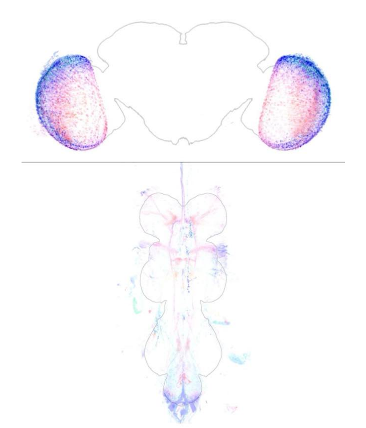

SS00806

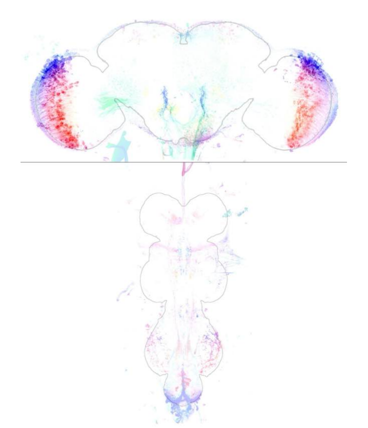

SS00807

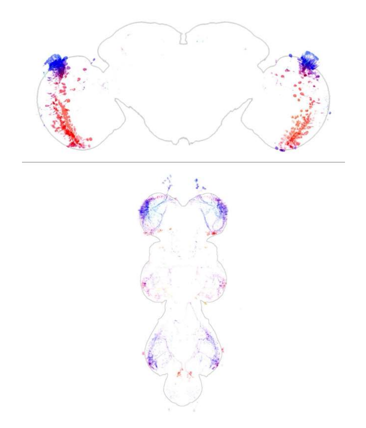

SS00808

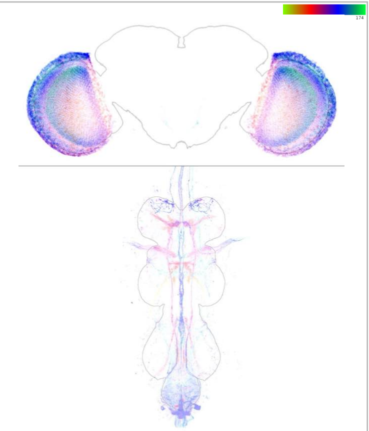
SS00809

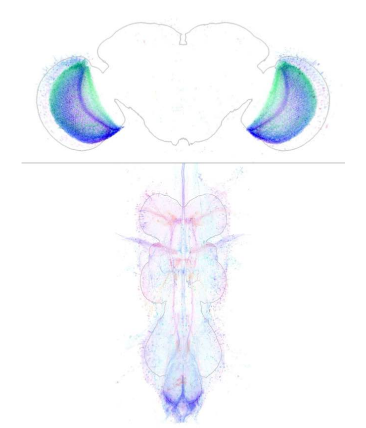
SS00810

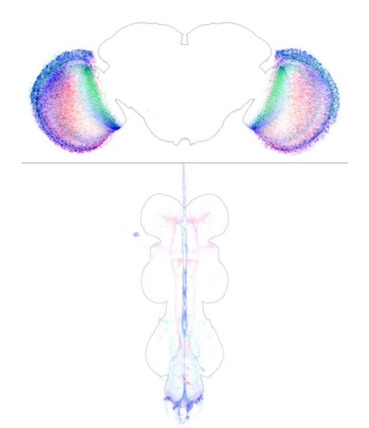
SS00811

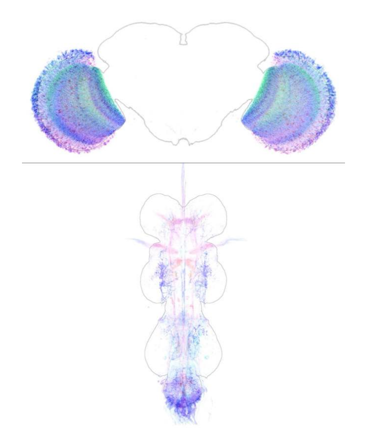
SS00813

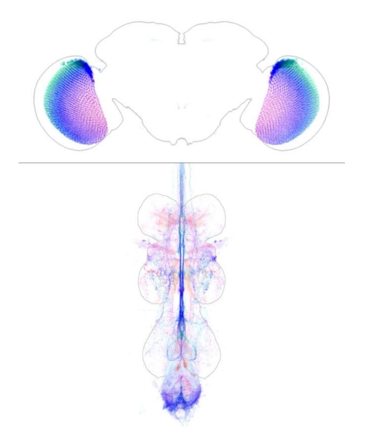
SS00814

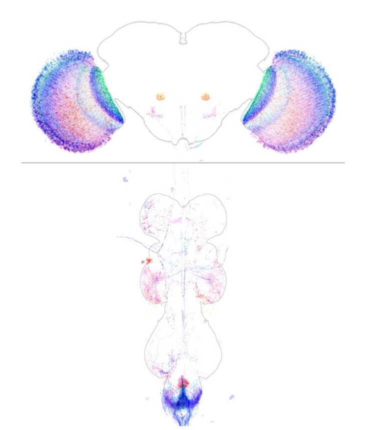
SS00817

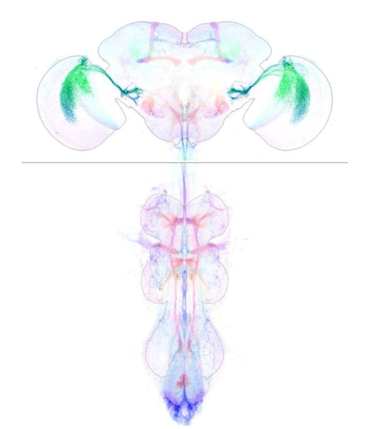
SS00818

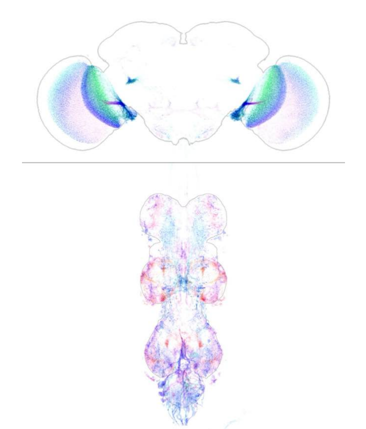
SS00820

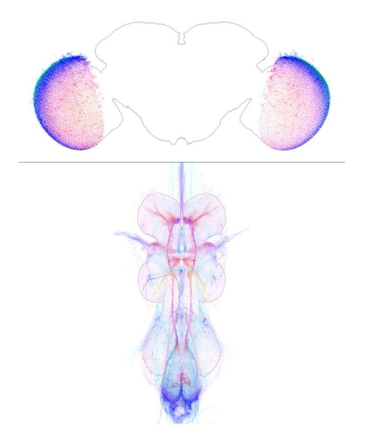
SS00821

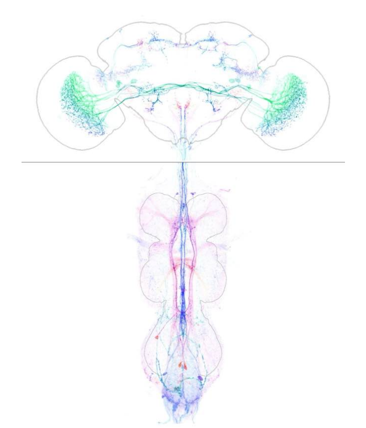
SS00823

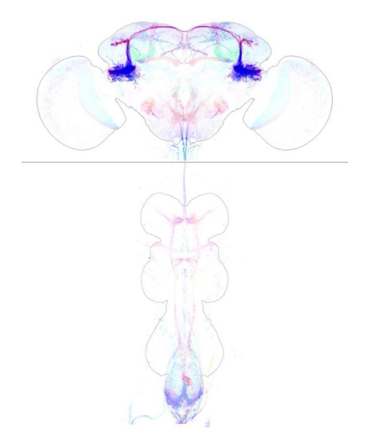
SS00824

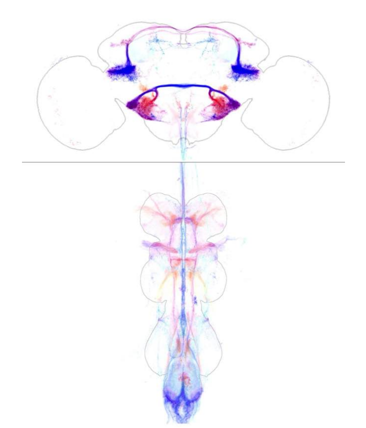
SS00825

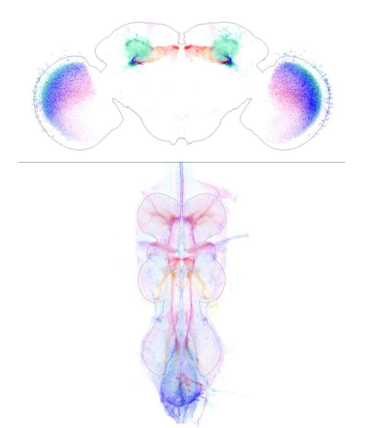
SS00835

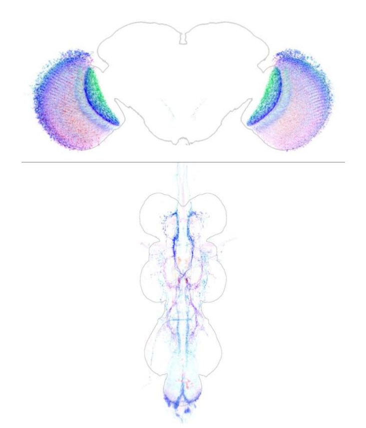
SS00836

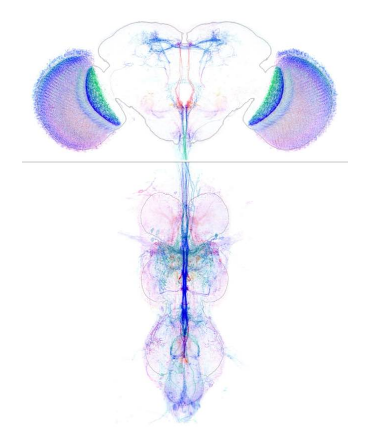
SS00837

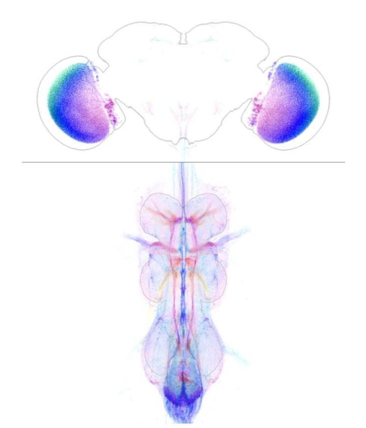
SS00839

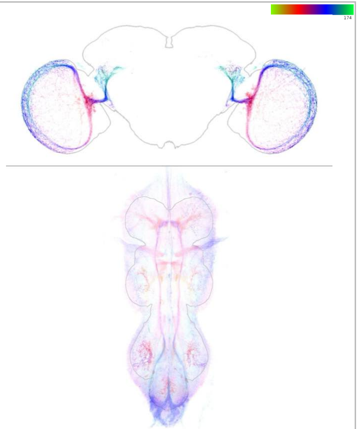
SS00844

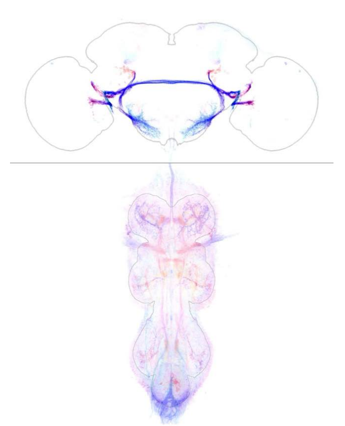
SS00847

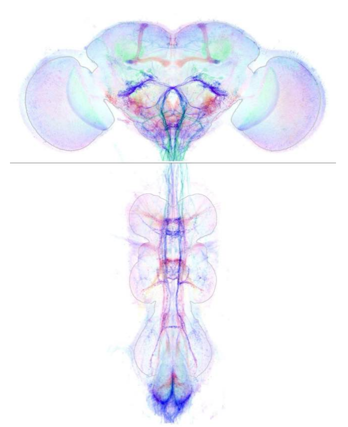
SS00865

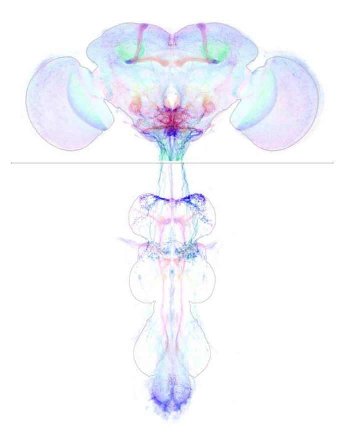
SS00898

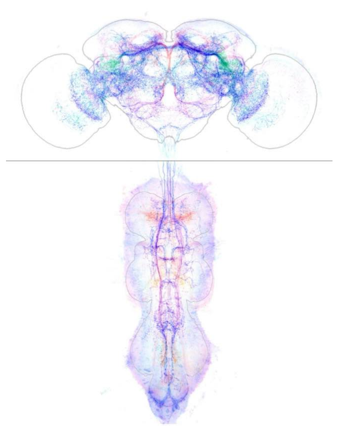
SS00923

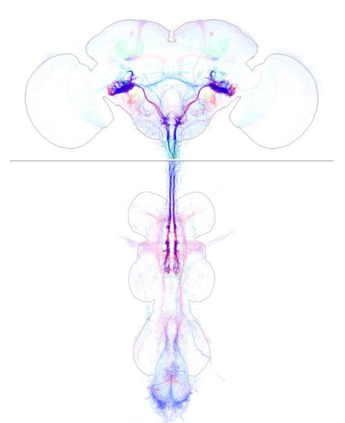
SS00934

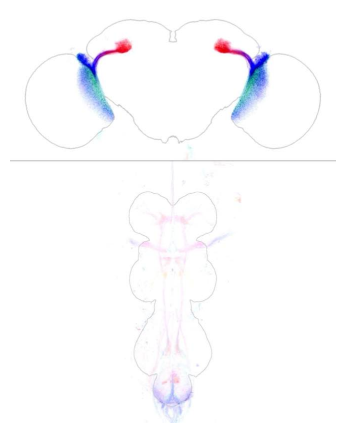
SS00937

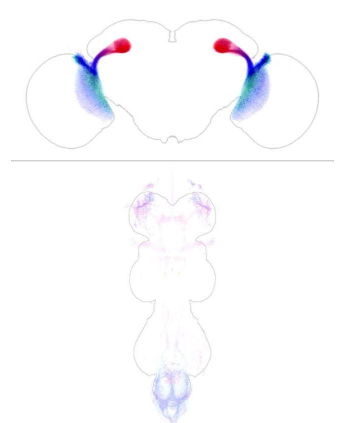
SS00938

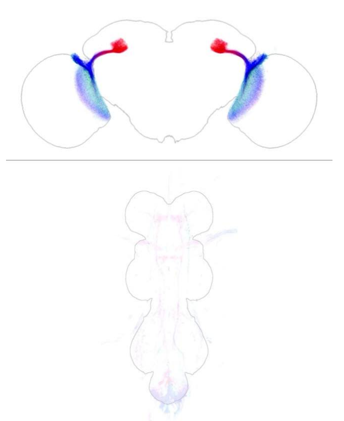
SS00940

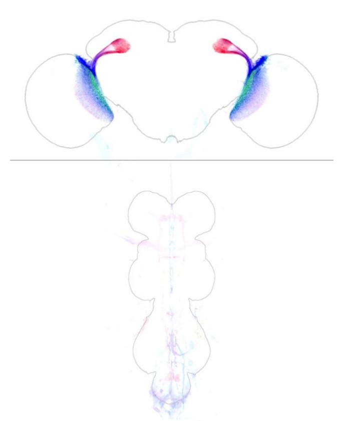
SS00941

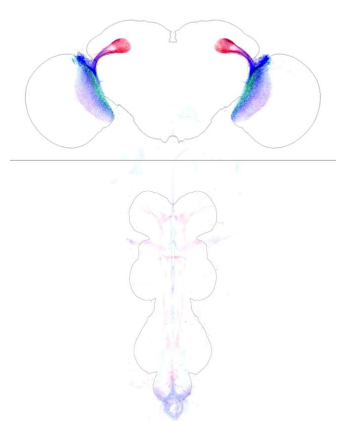
SS00942

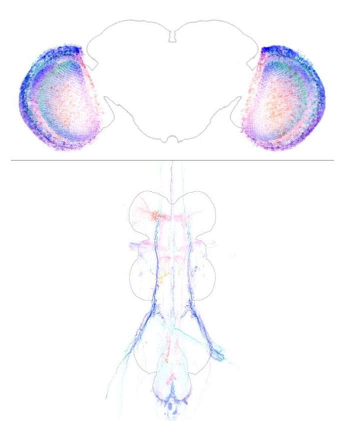
SS00955

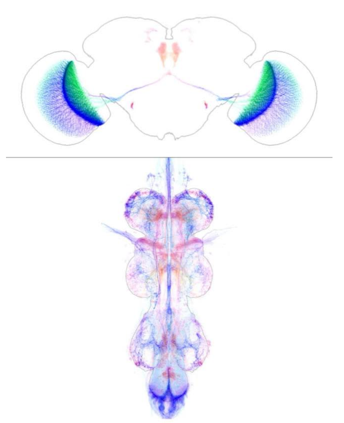
SS01001

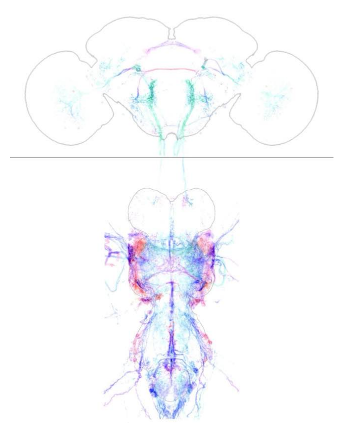
SS01010

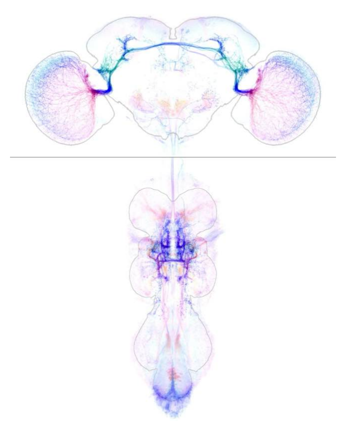
SS01015

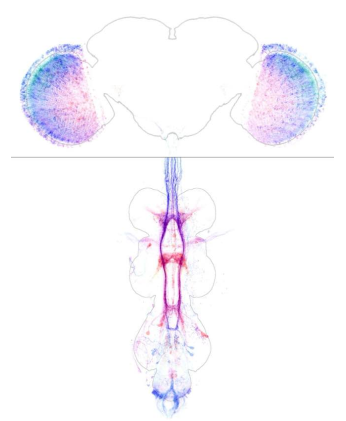
SS01019

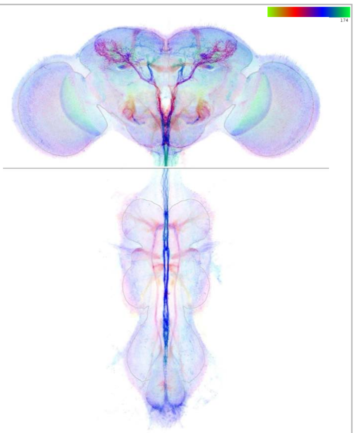
SS01046

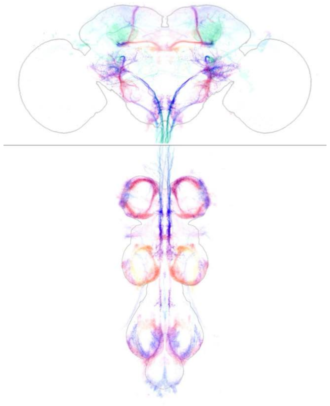
SS01047

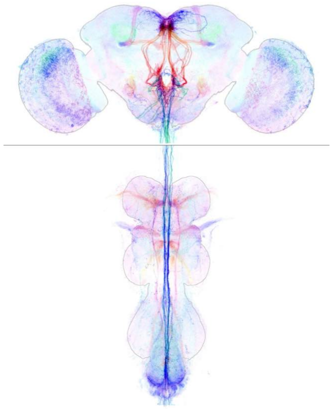
SS01048

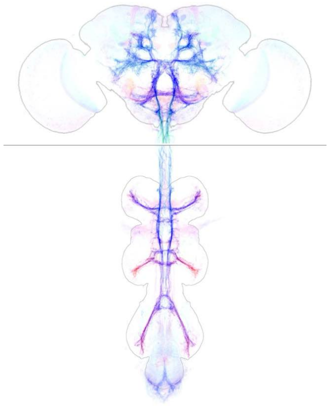
SS01049

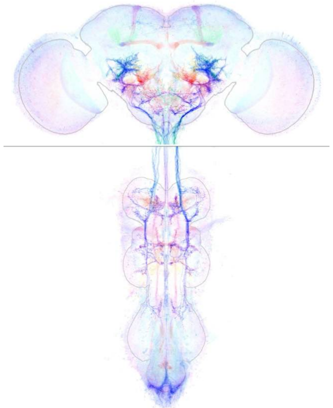
SS01051

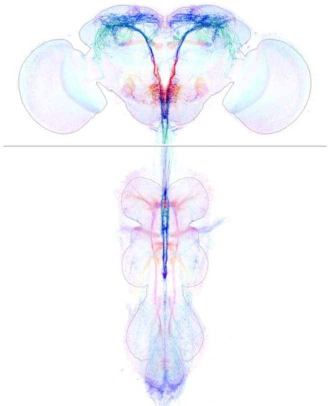
SS01052

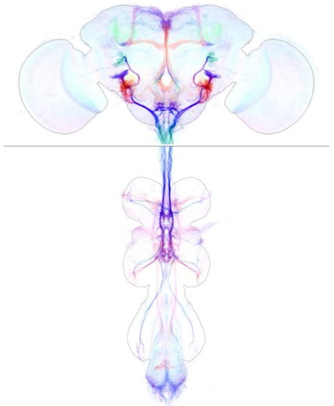
SS01053

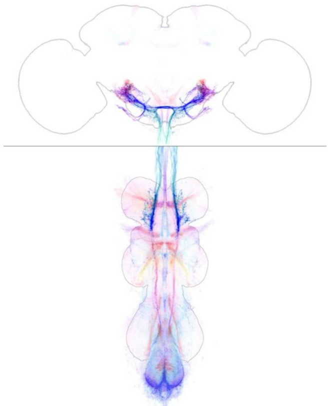
SS01058

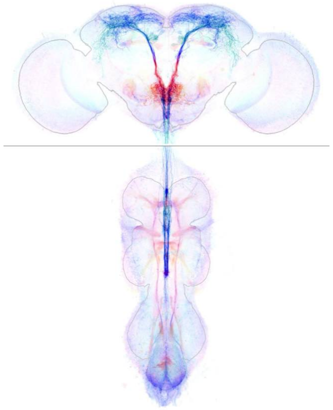
SS01059

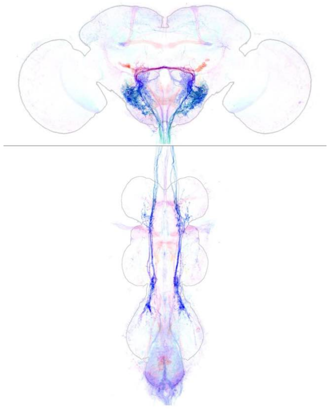
SS01060

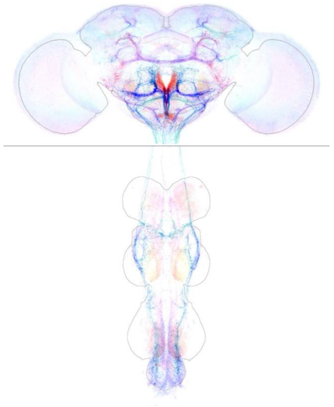
SS01061

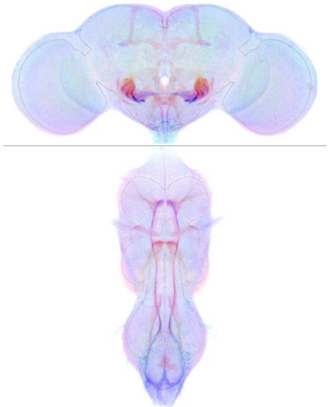
SS01062

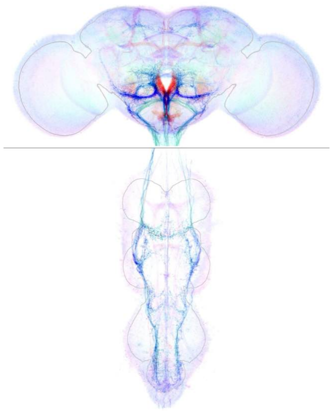
SS01064

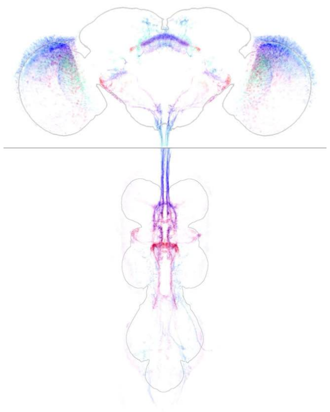
SS01065

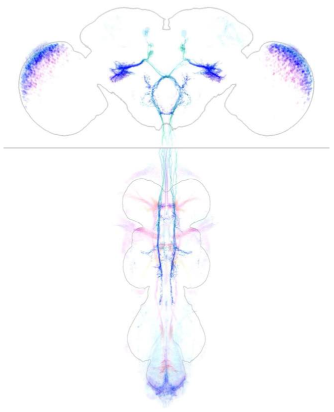
SS01067

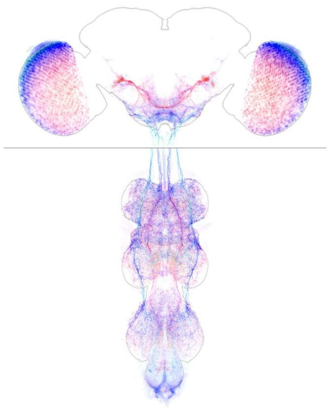
SS01068

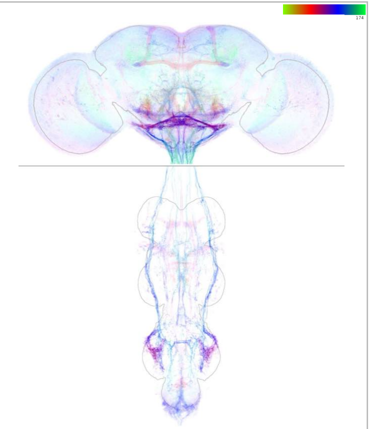
SS01069

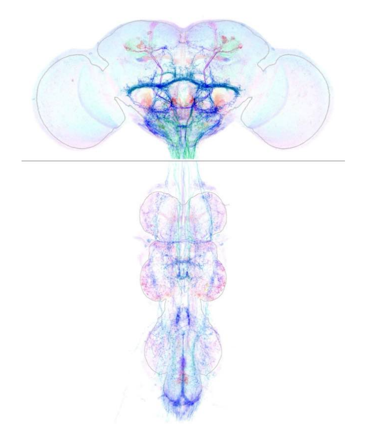
SS01070

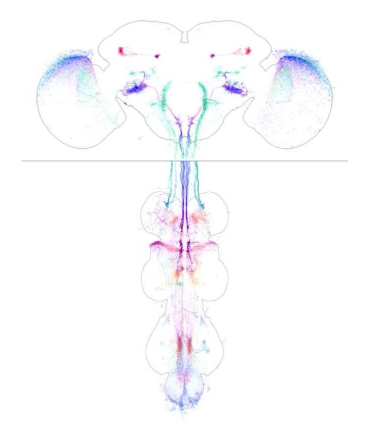
SS01071

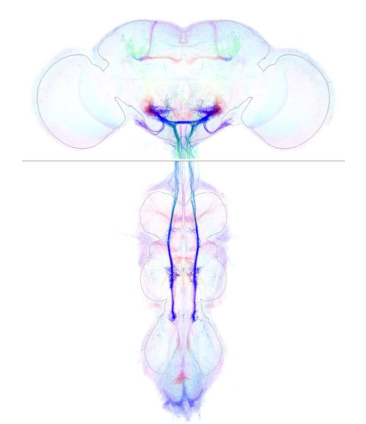
SS01072

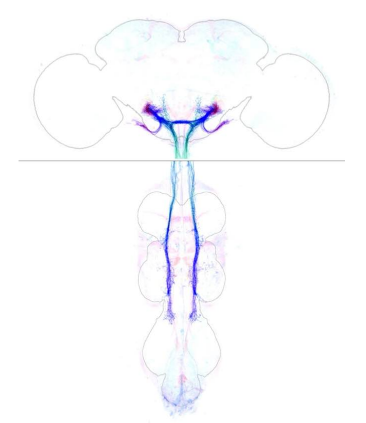
SS01074

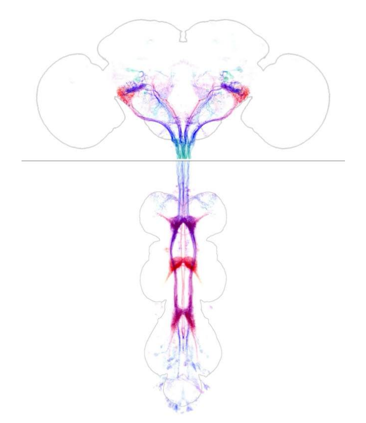
SS01076

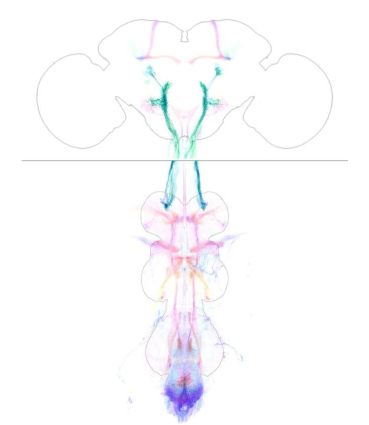
SS01078

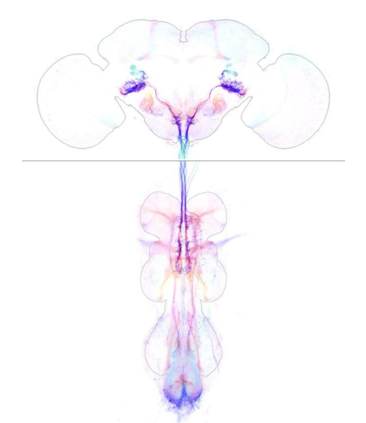
SS01080

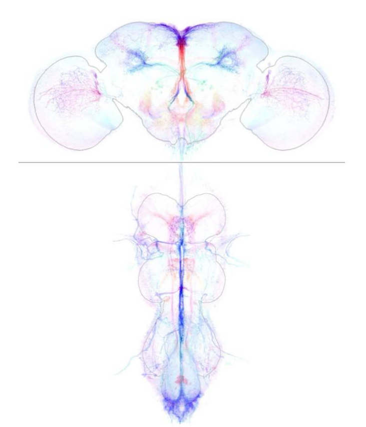
SS01082

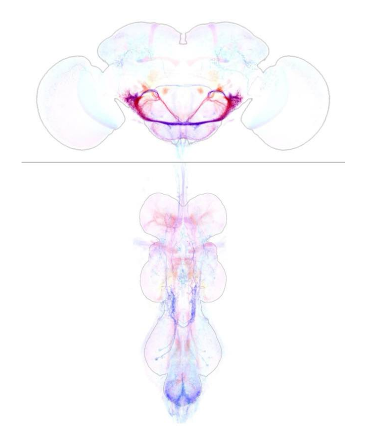
SS01090

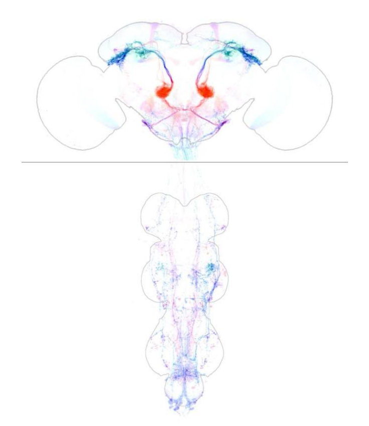
SS01100

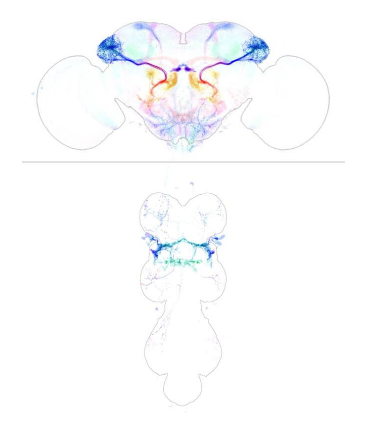
SS01108

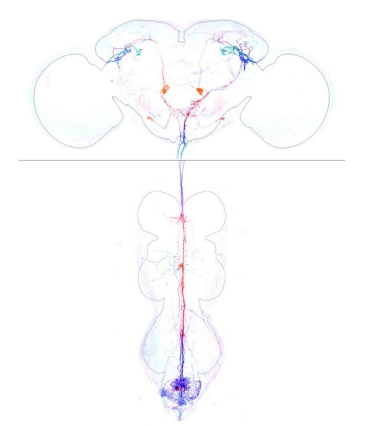
SS01111

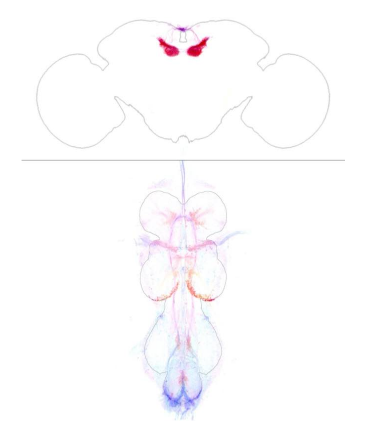
SS01127

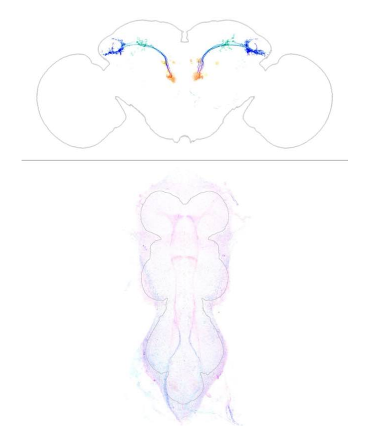
SS01132

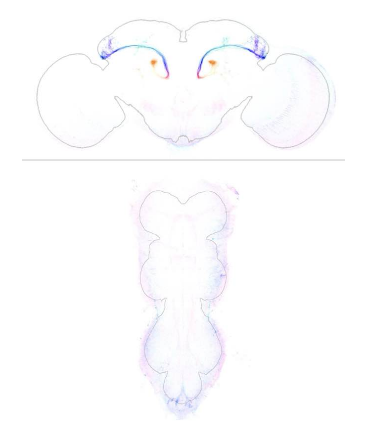
SS01147

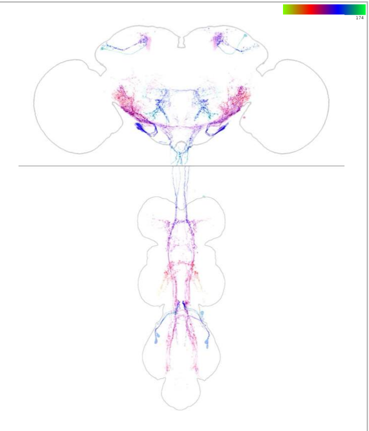

SS01154

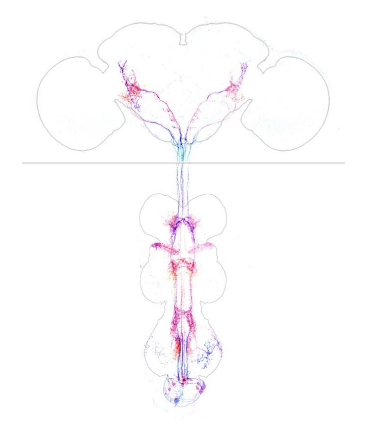

SS01160

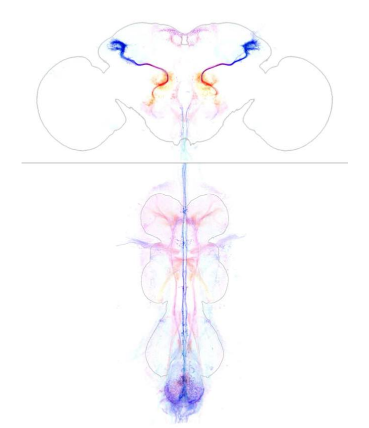

SS01168

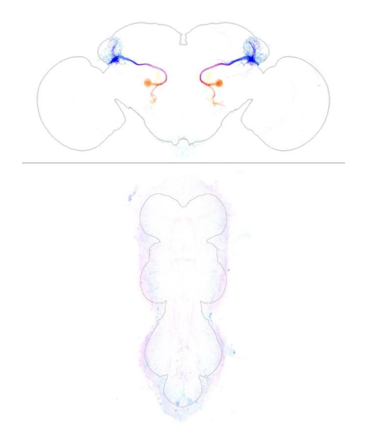

SS01171

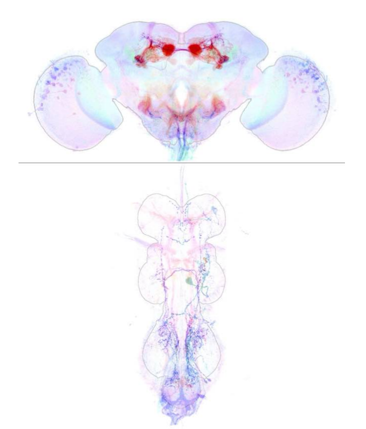

SS01188

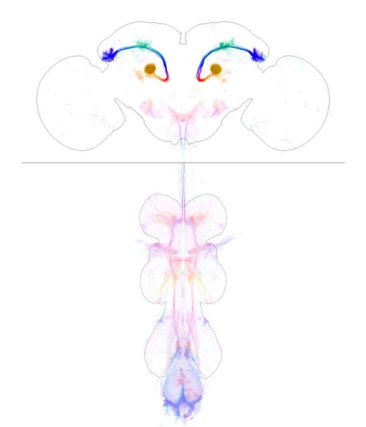

SS01189

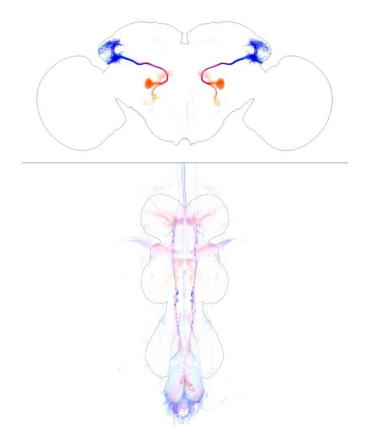

SS01196

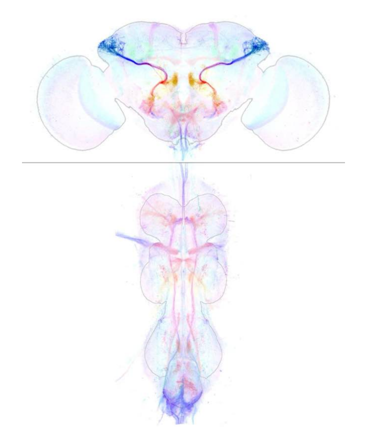

SS01197

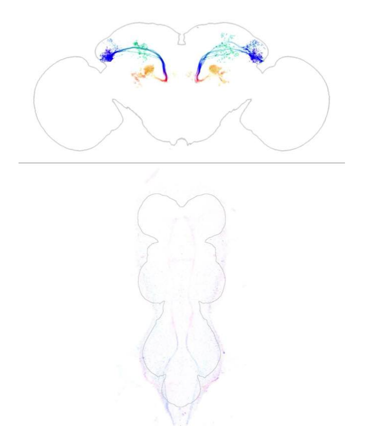

SS01199

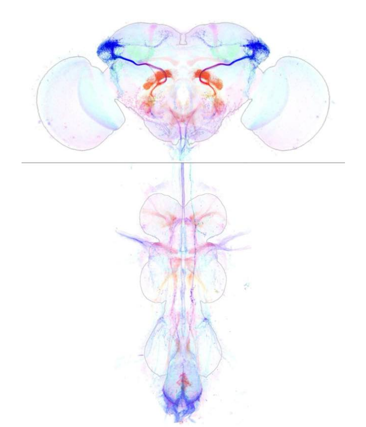

SS01202

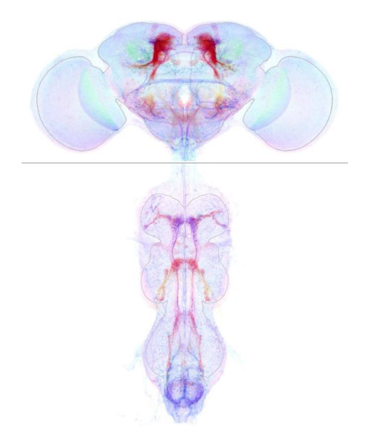

SS01203

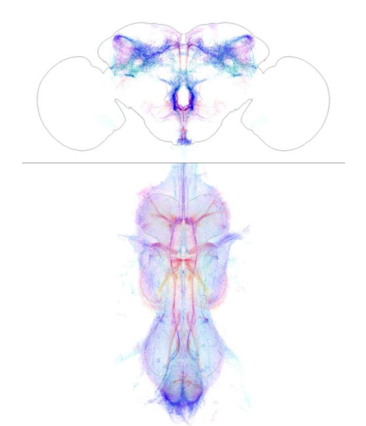

SS01208

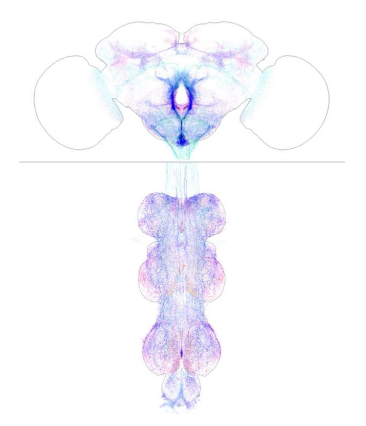

SS01210

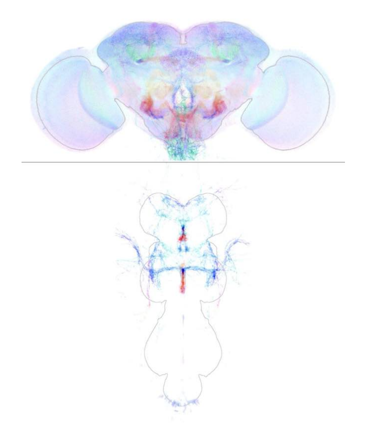

SS01216

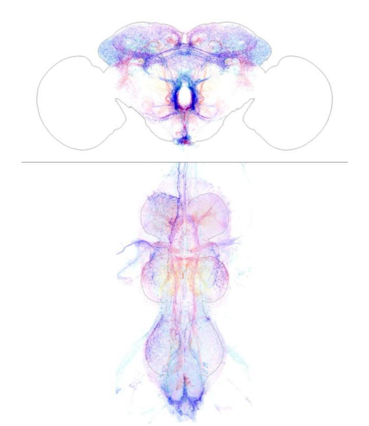

SS01217

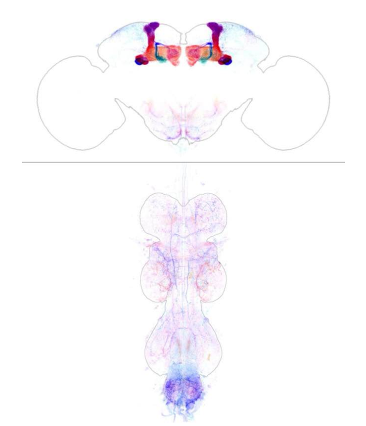

SS01241

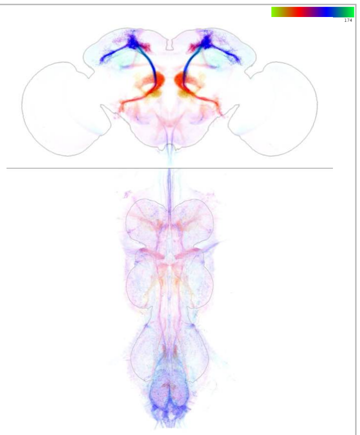
SS01262

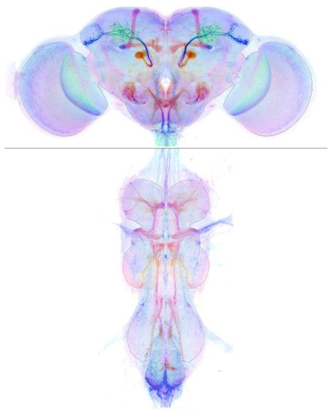
SS01269

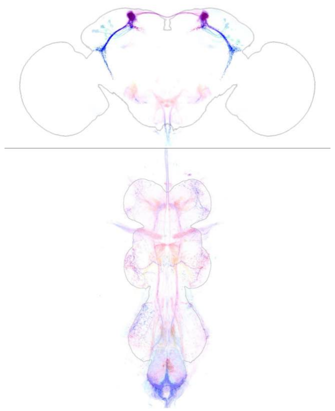
SS01298

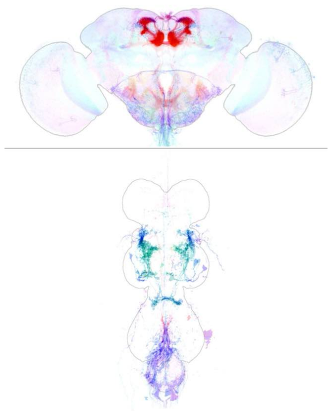
SS01308

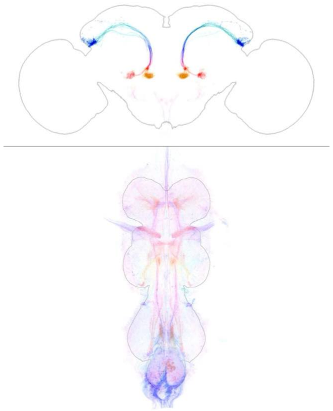
SS01318

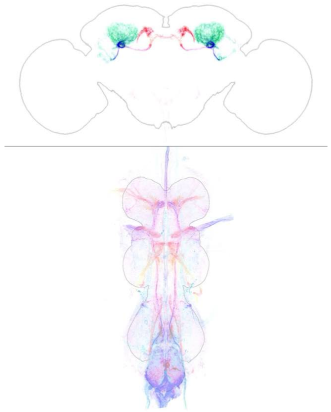
SS01388

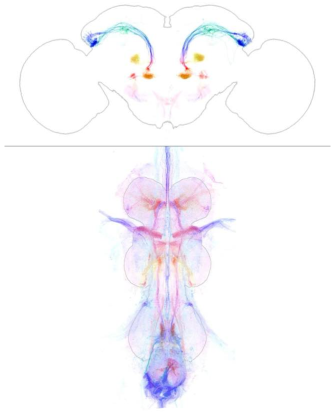
SS01390

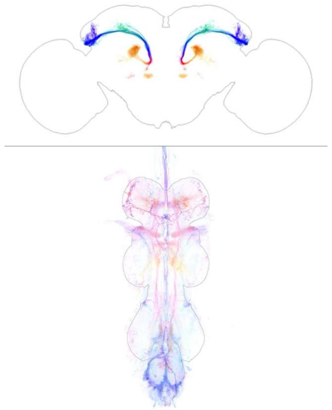
SS01391

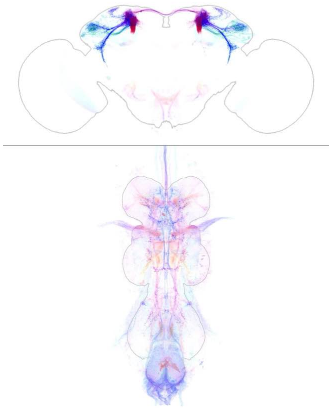
SS01396

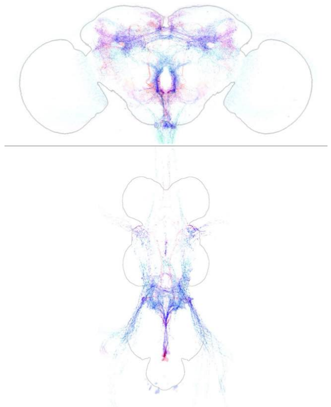
SS01427

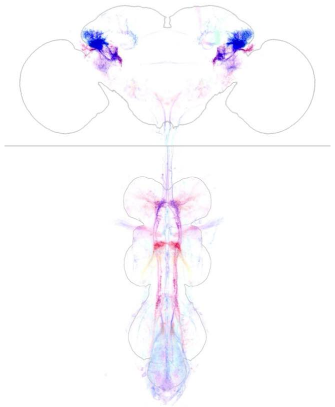
SS01475

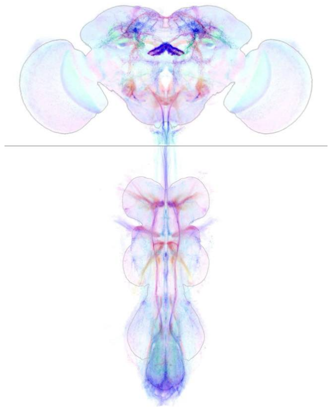
SS01491

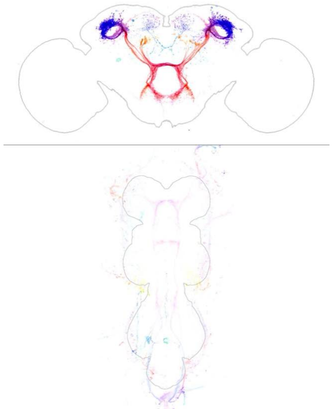
SS01506

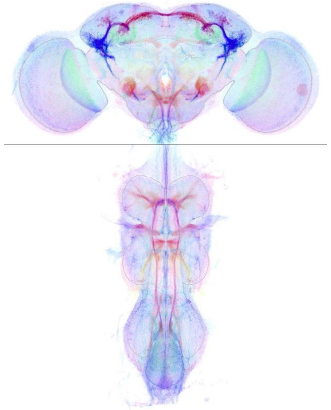
SS01516

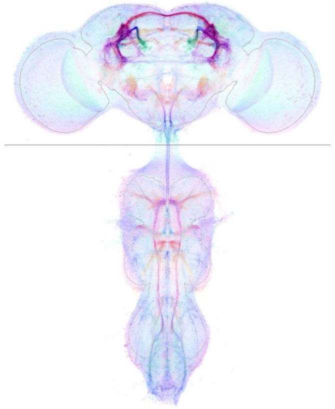
SS01539

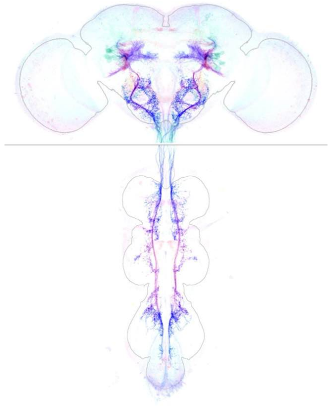
SS01540

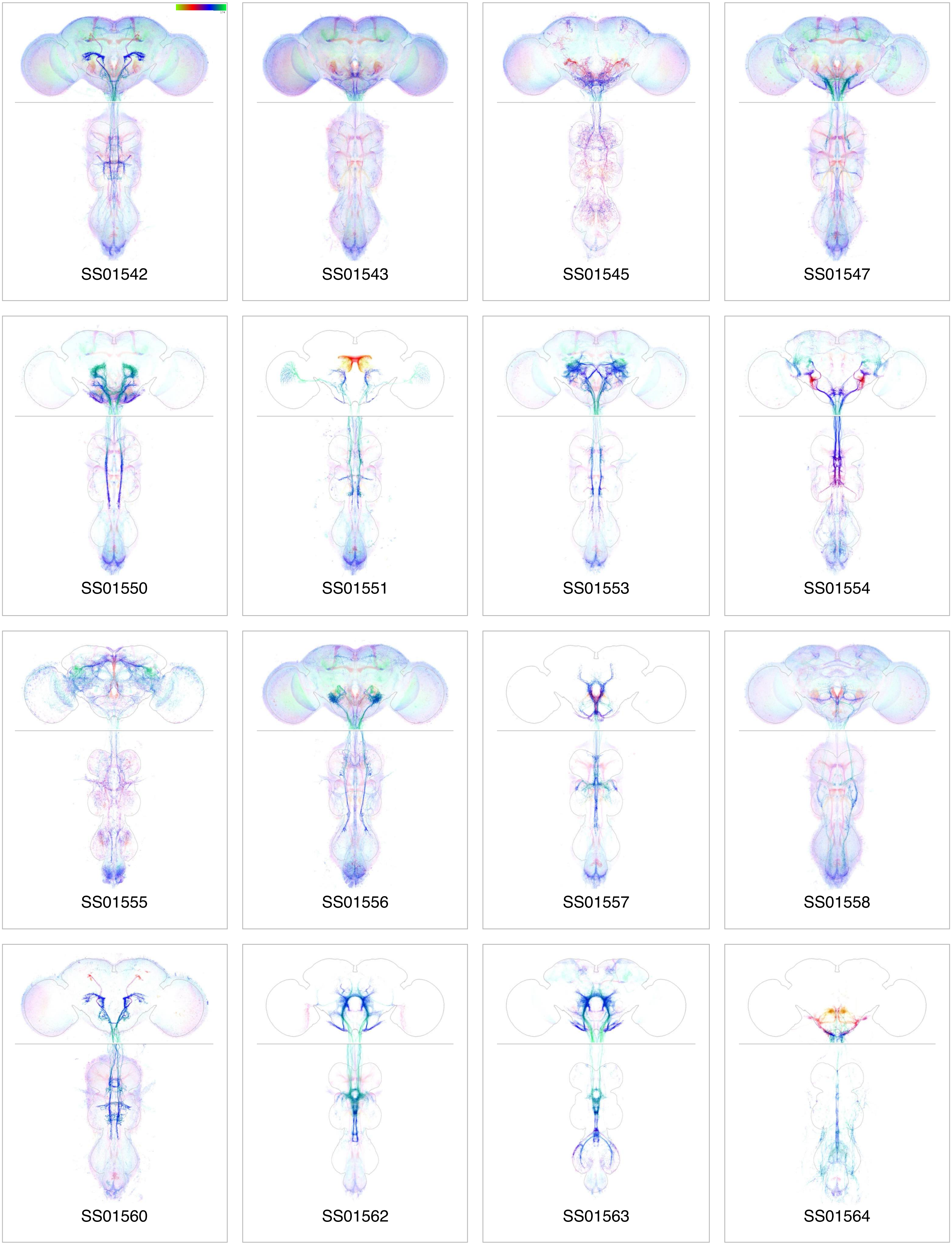
SS01542
SS01543
SS01545
SS01547
SS01550
SS01551
SS01553
SS01554
SS01555
SS01556
SS01557
SS01558
SS01560
SS01562
SS01563
SS01564

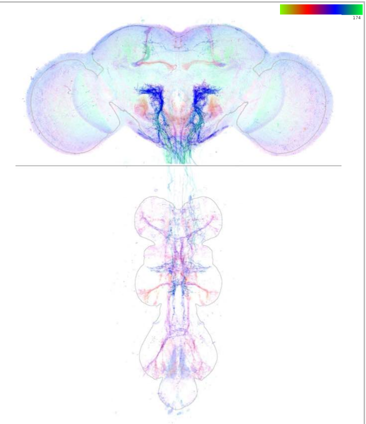
SS01565

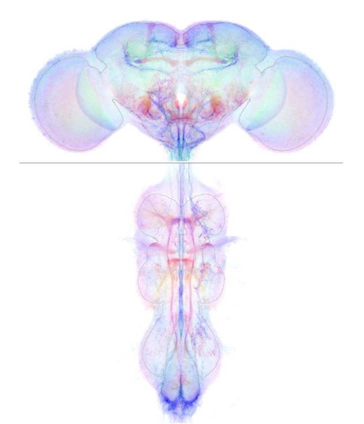
SS01567

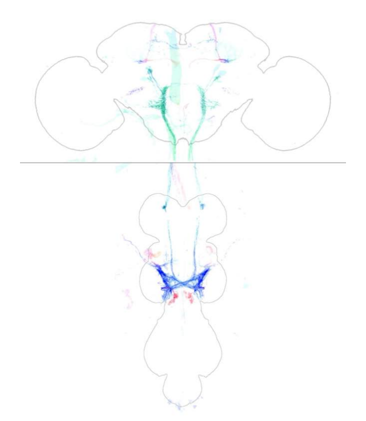
SS01568

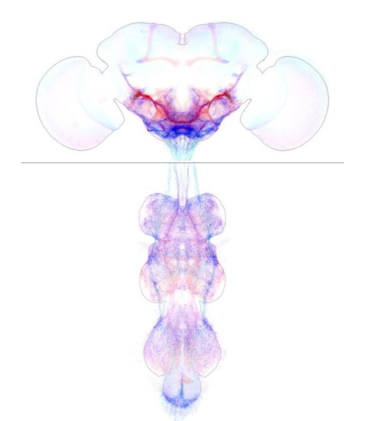
SS01570

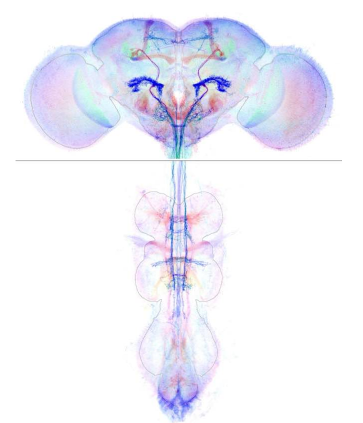
SS01571

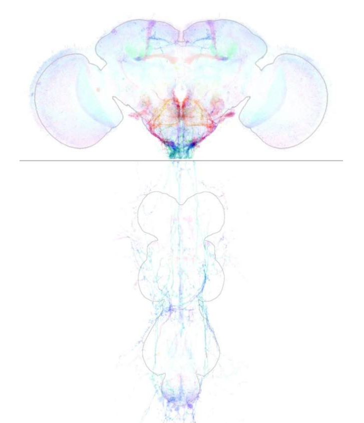
SS01574

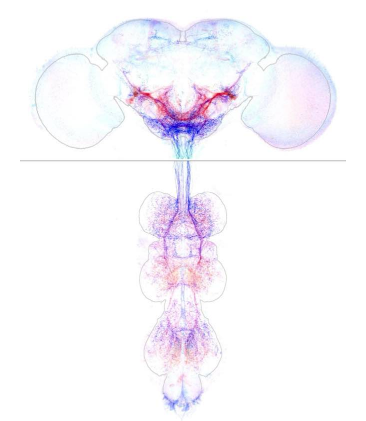
SS01575

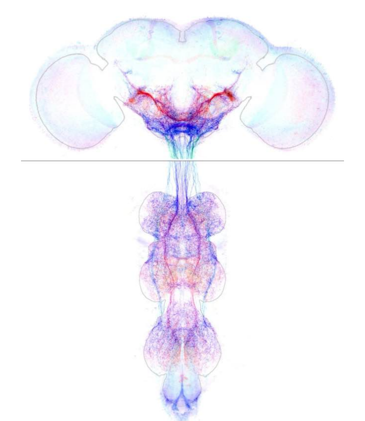
SS01576

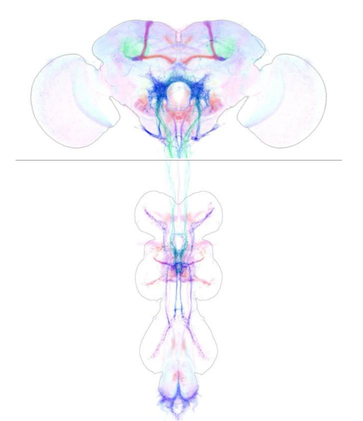
SS01577

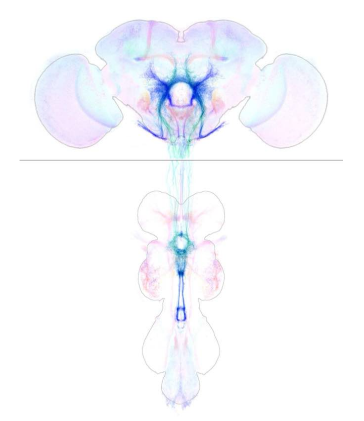
SS01578

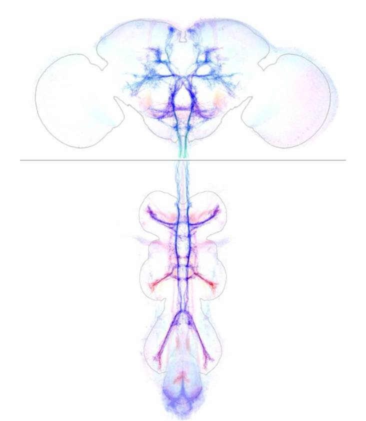
SS01580

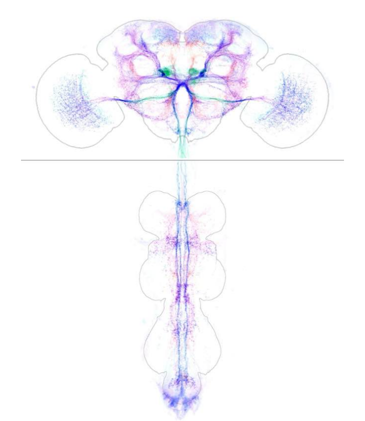
SS01581

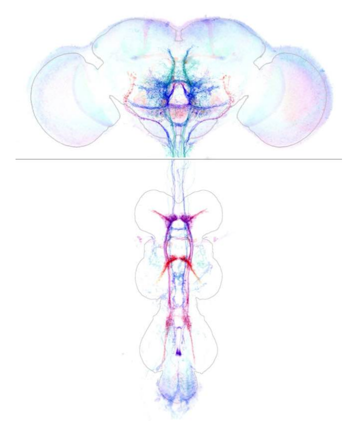
SS01582

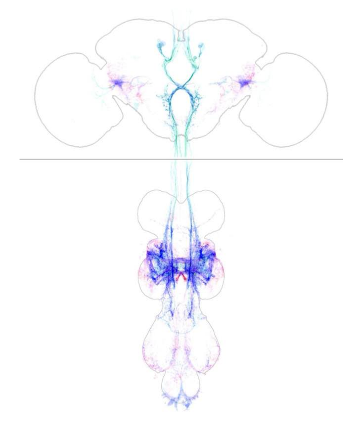
SS01583

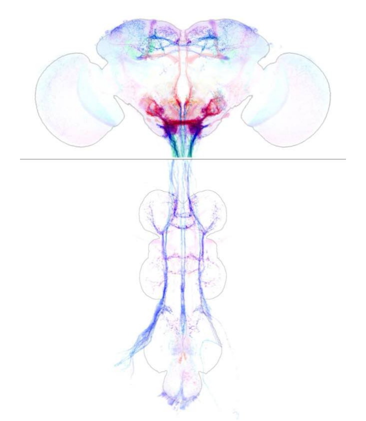
SS01584

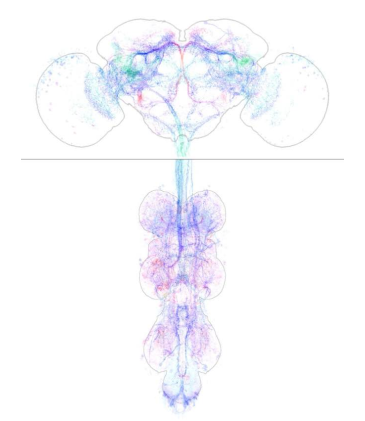
SS01585

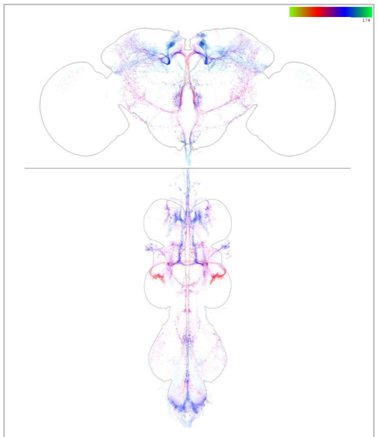

SS01587

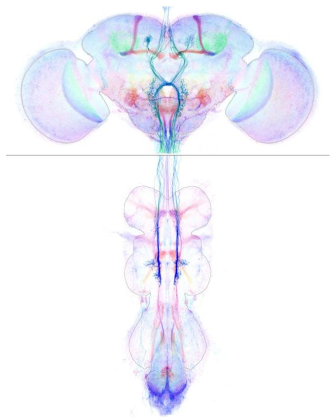

SS01589

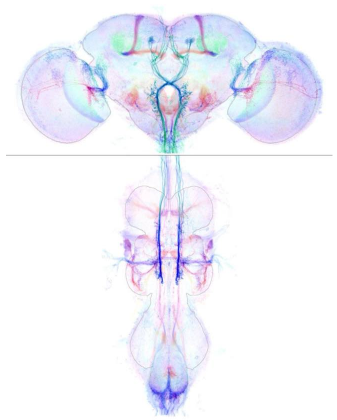

SS01590

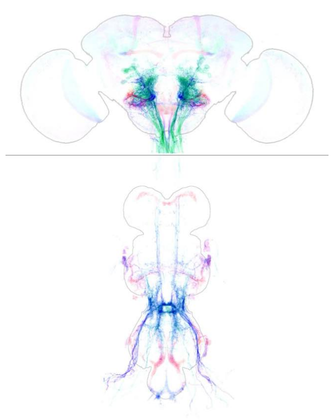

SS01592

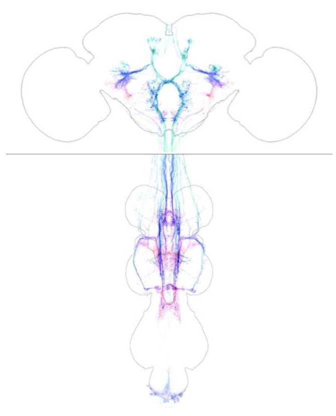

SS01594

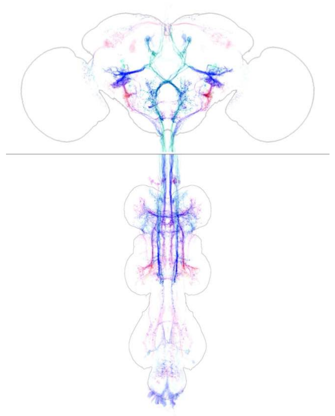

SS01595

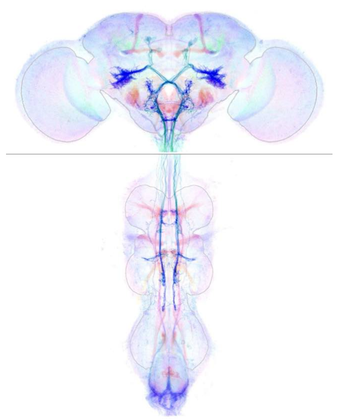

SS01596

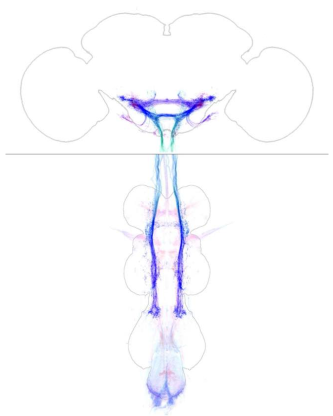

SS01597

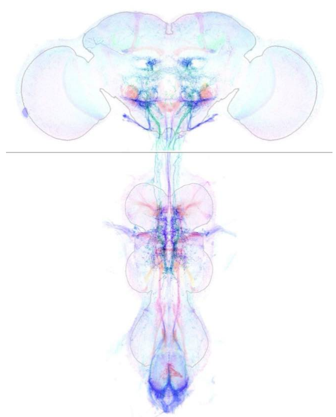

SS01598

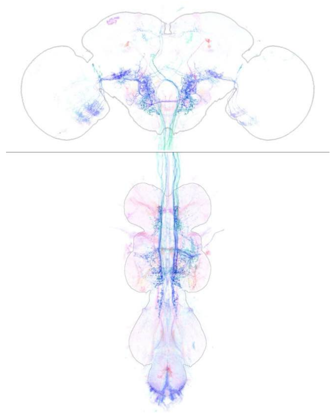

SS01599

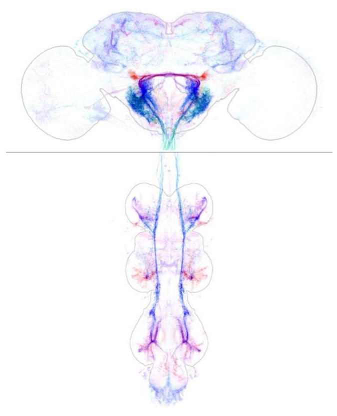

SS01600

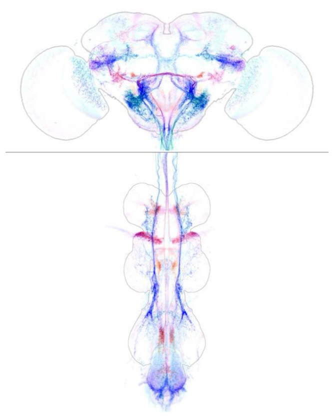

SS01601

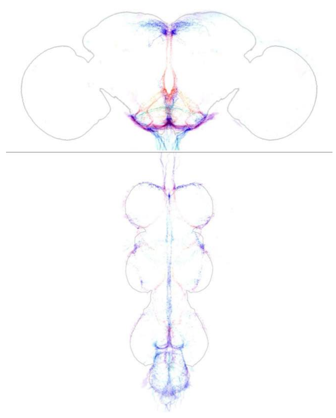

SS01602

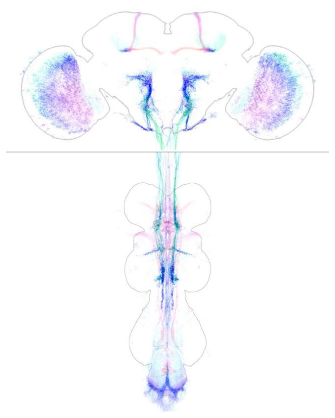

SS01603

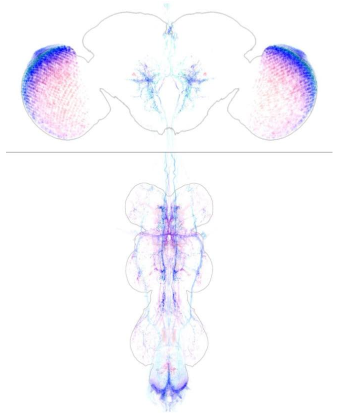

SS01605

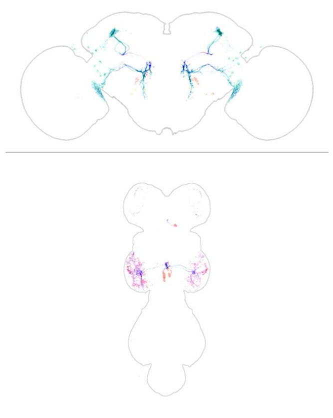

SS01828

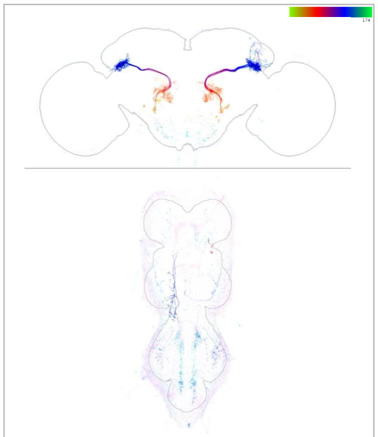
SS01845

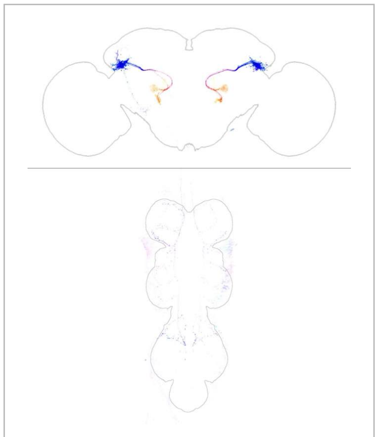
SS01850

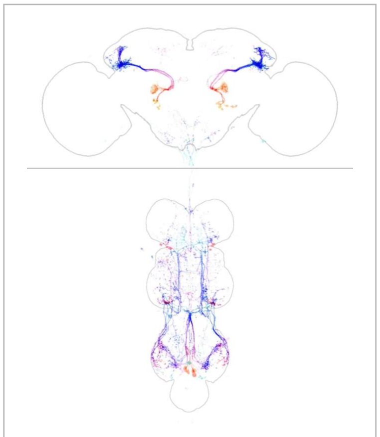
SS01854

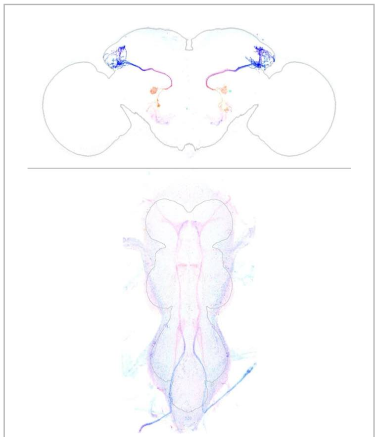
SS01868

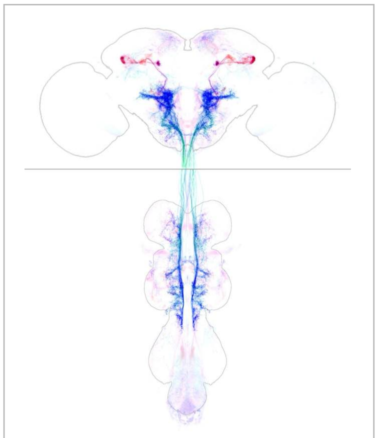
SS01965

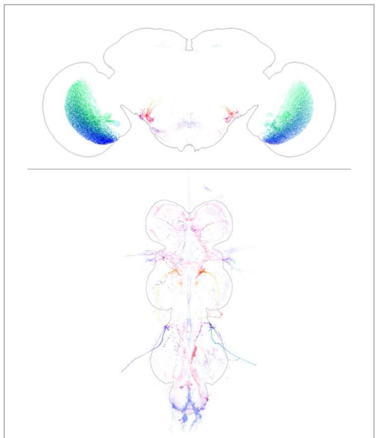
SS02023

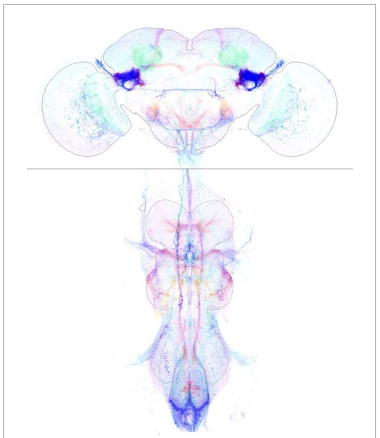
SS02036

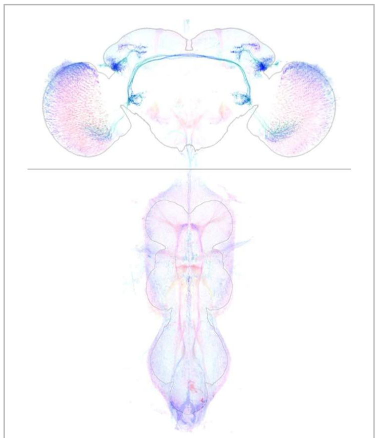
SS02037

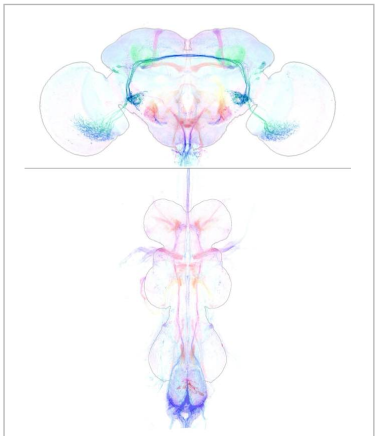
SS02038

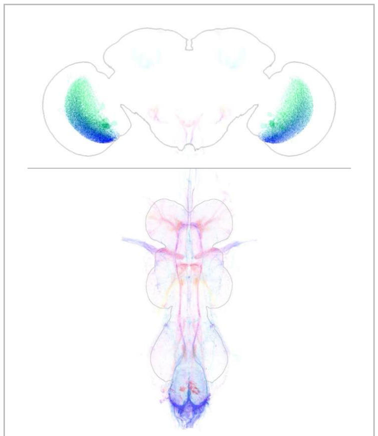
SS02043

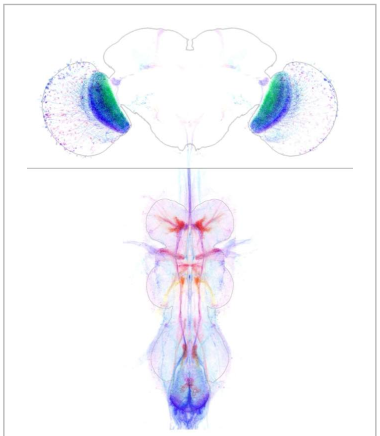
SS02044

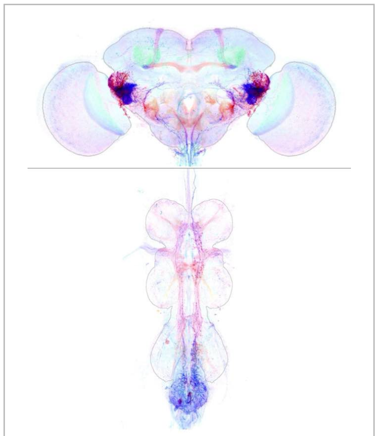
SS02050

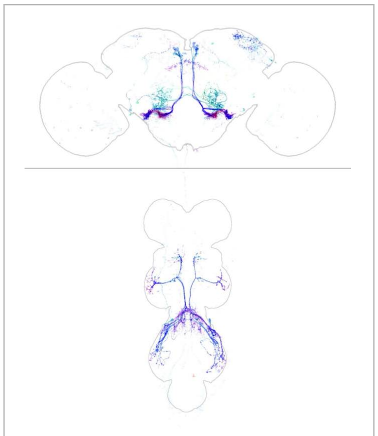
SS02052

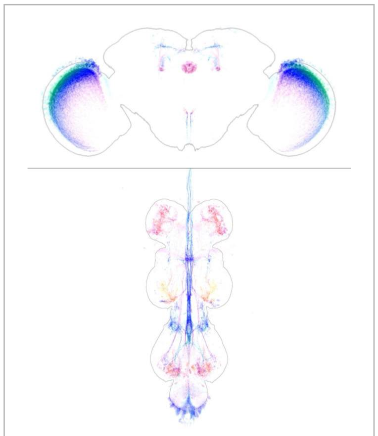
SS02054

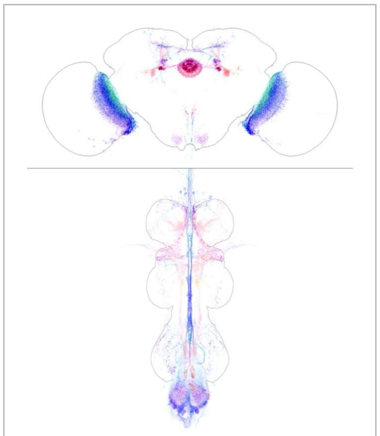
SS02056

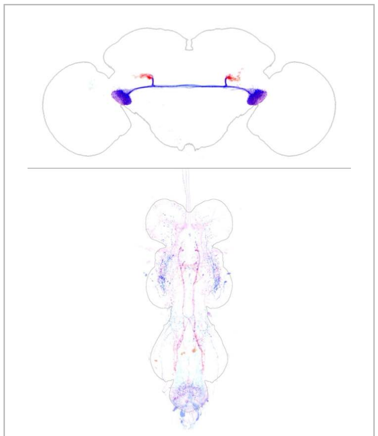
SS02057

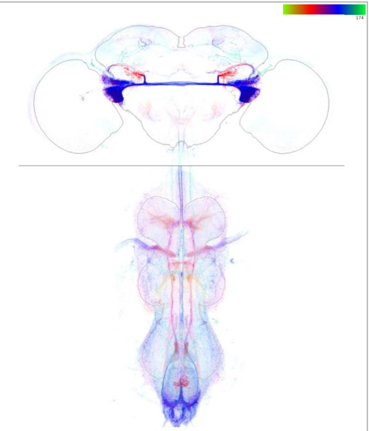

SS02058

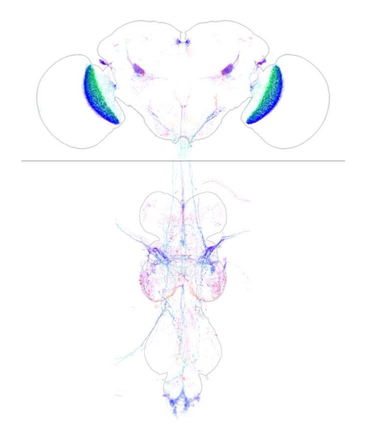

SS02062

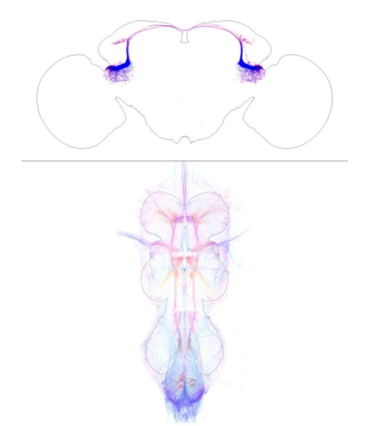

SS02072

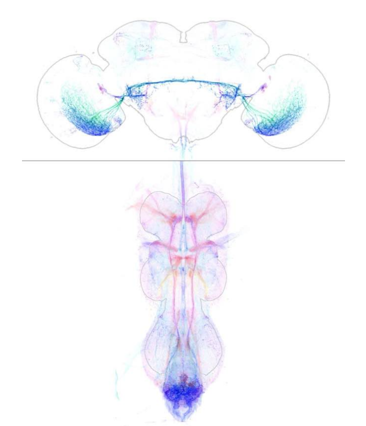

SS02075

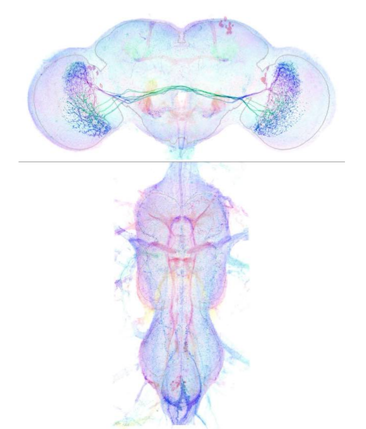

SS02082

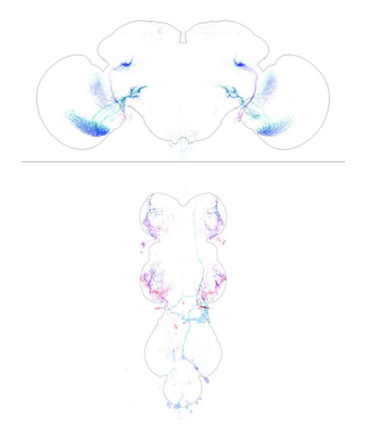

SS02087

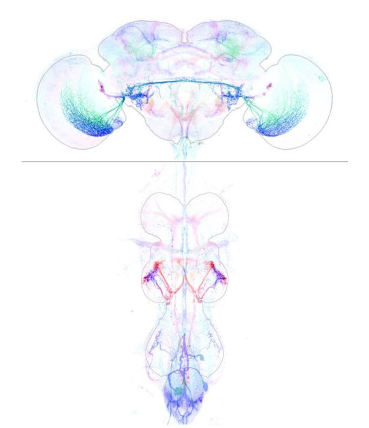

SS02092

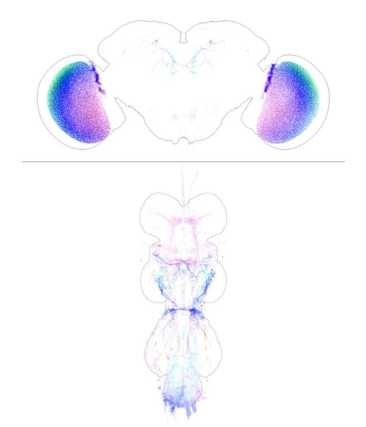

SS02096

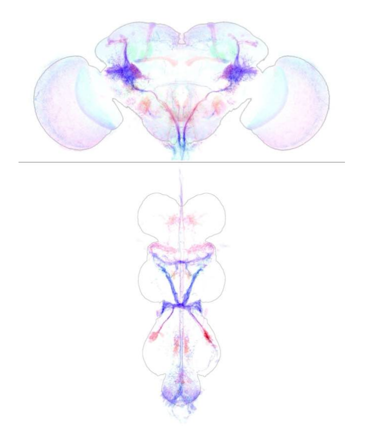

SS02099

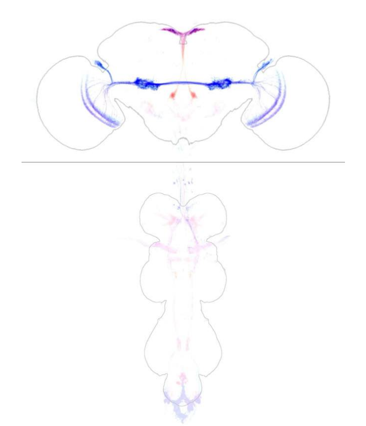

SS02102

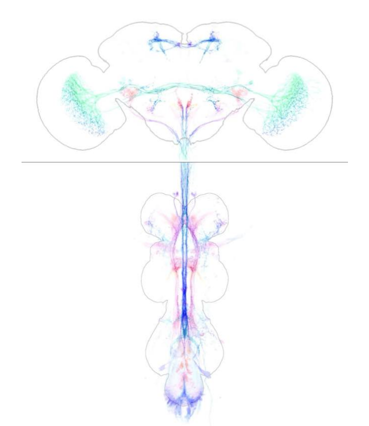

SS02103

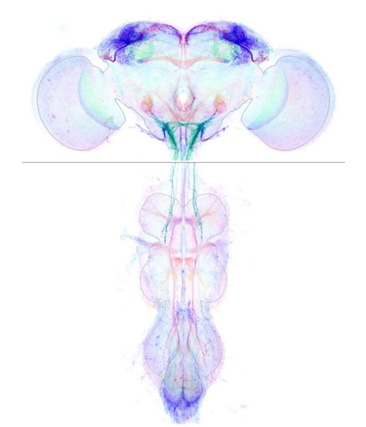

SS02111

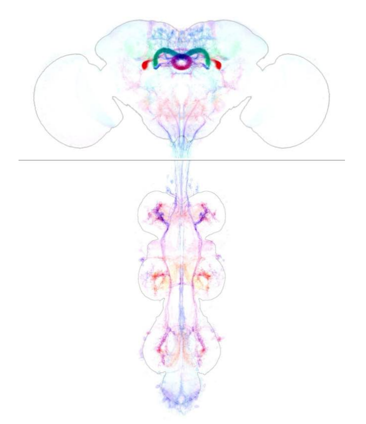

SS02191

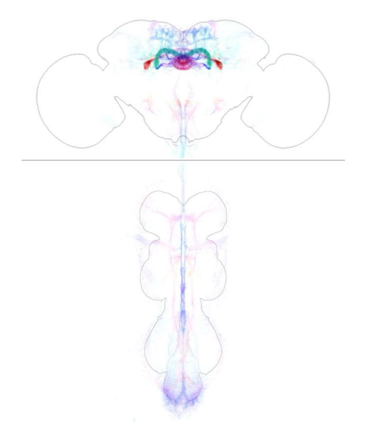

SS02195

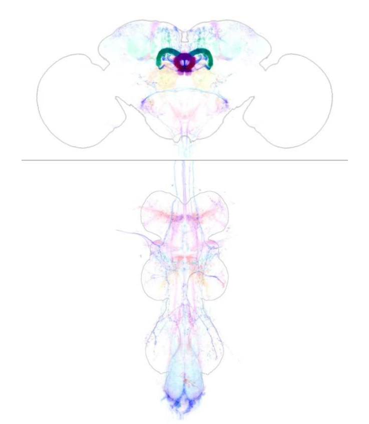

SS02232

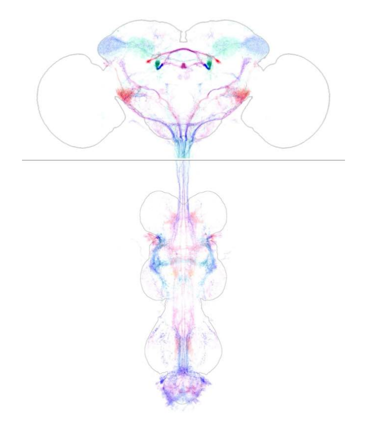

SS02254

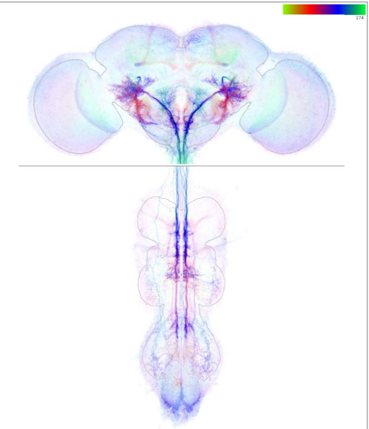
SS02256

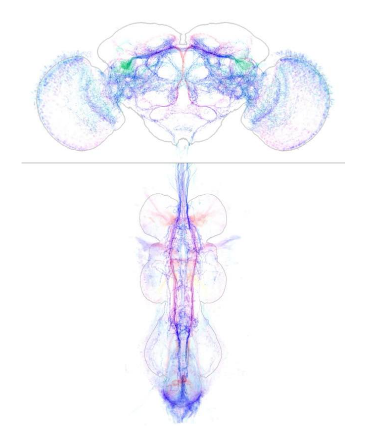
SS02257

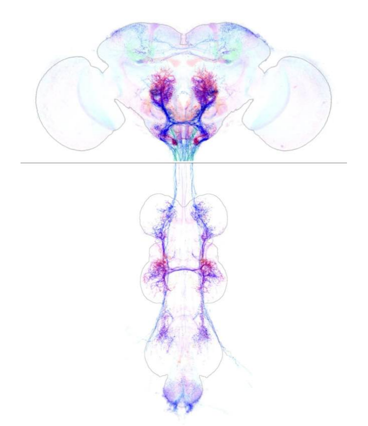
SS02259

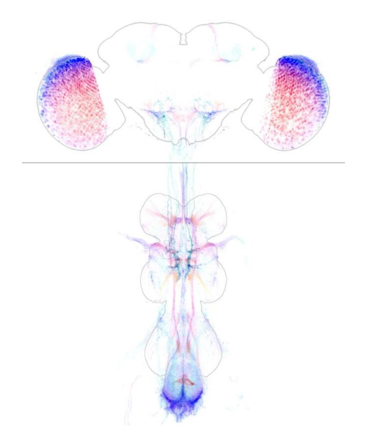
SS02260

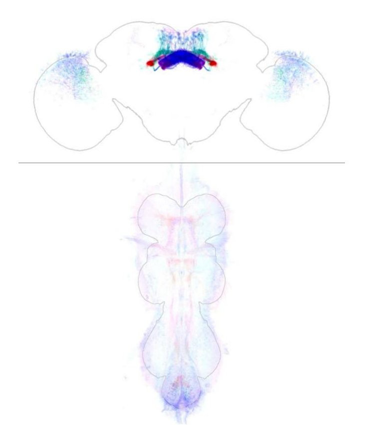
SS02270

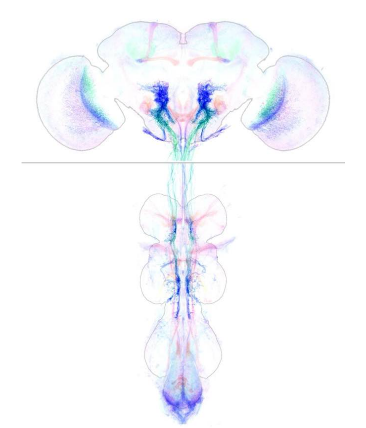
SS02275

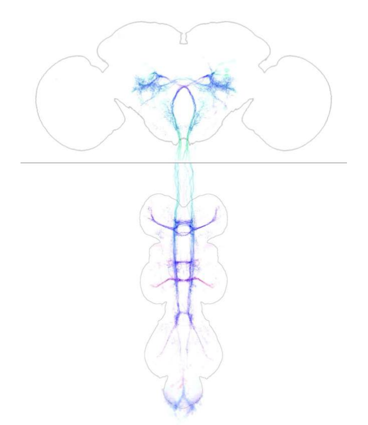
SS02276

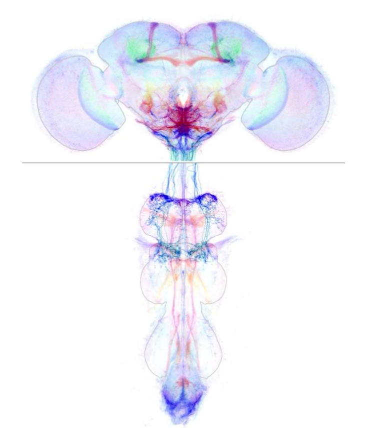
SS02279

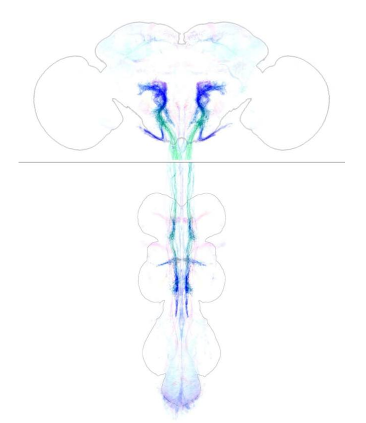
SS02285

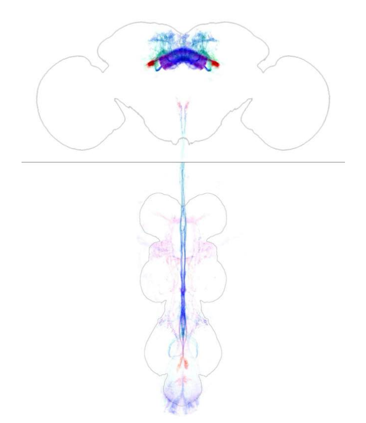
SS02293

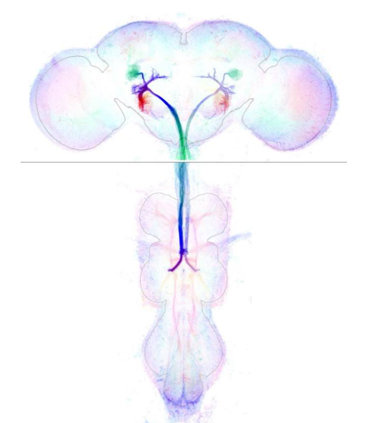
SS02299

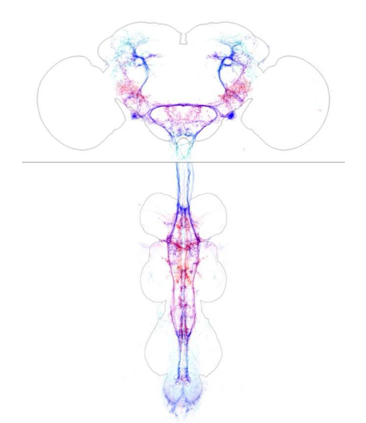
SS02316

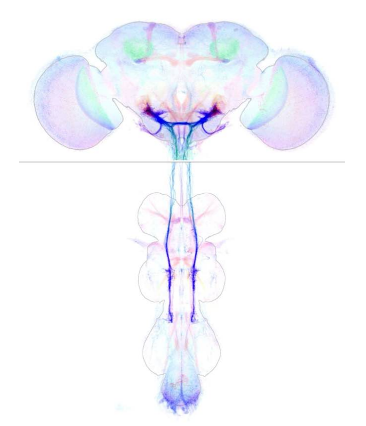
SS02324

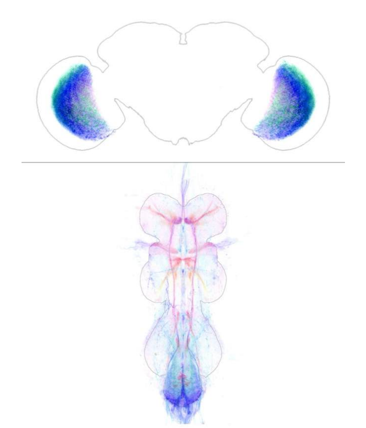
SS02344

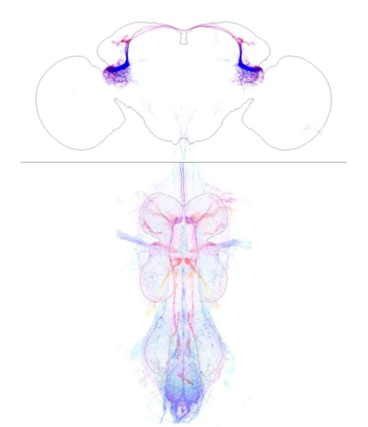
SS02347

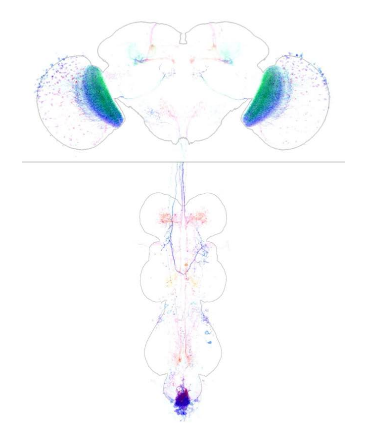
SS02349

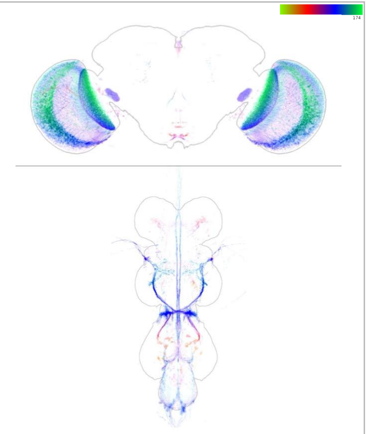
SS02353

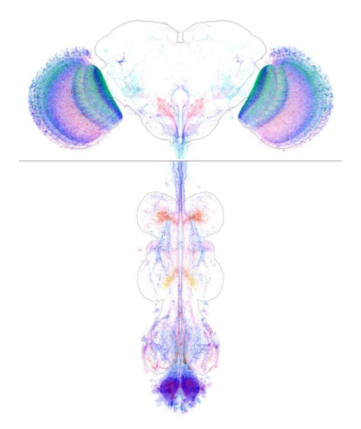
SS02358

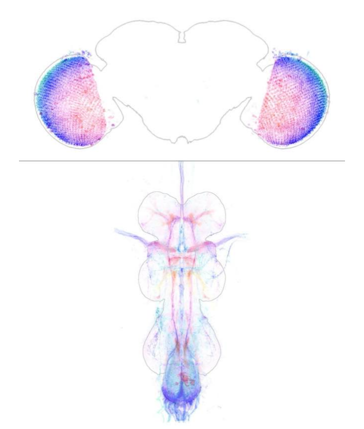
SS02360

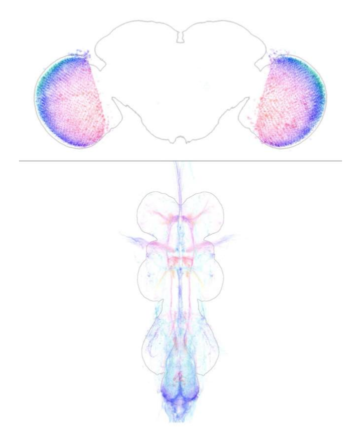
SS02361

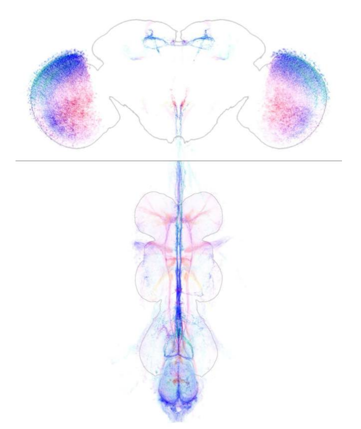
SS02364

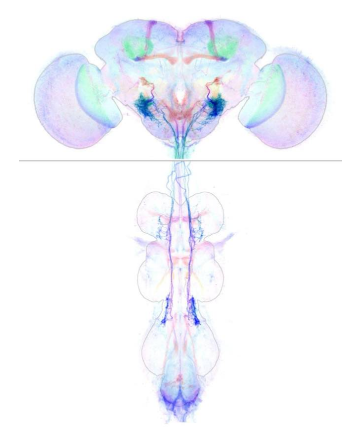
SS02370

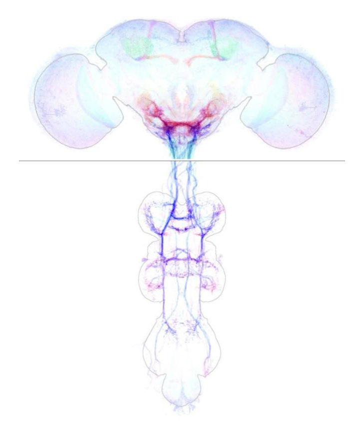
SS02377

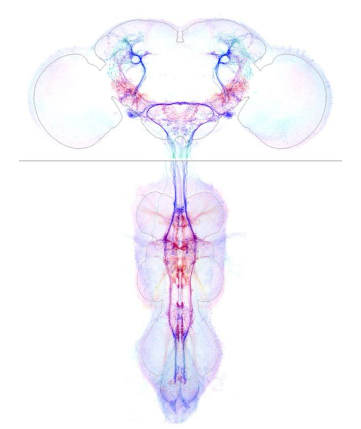
SS02378

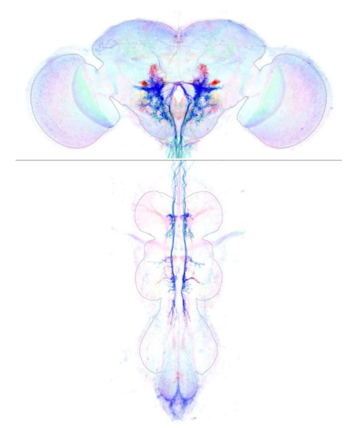
SS02383

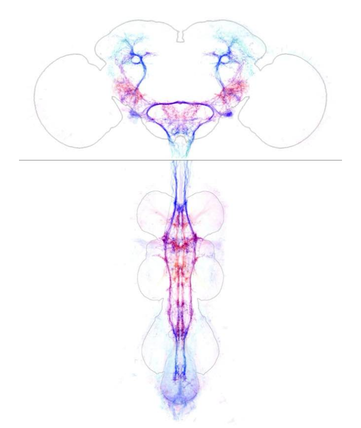
SS02388

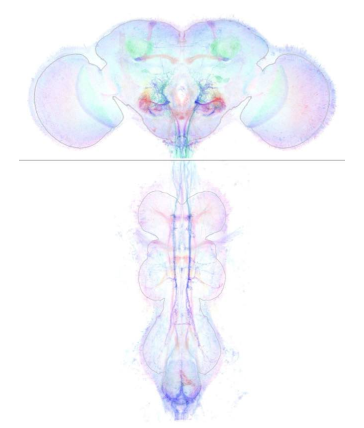
SS02392

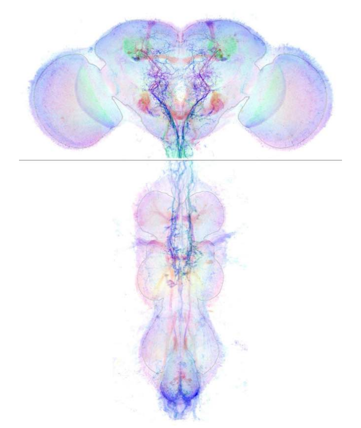
SS02393

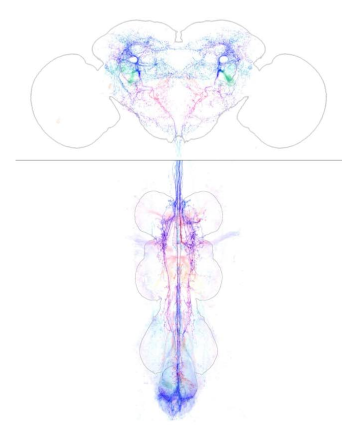
SS02394

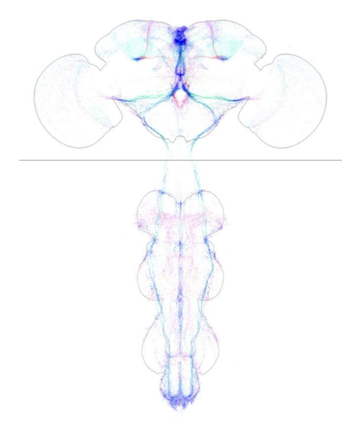
SS02395

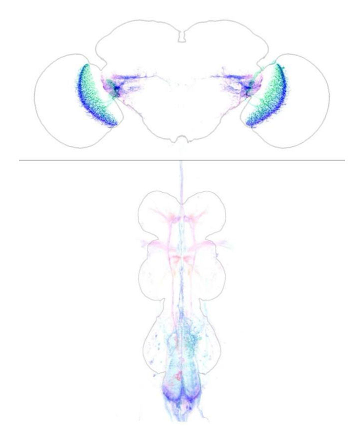
SS02405

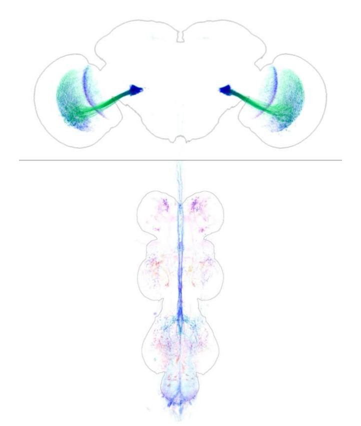
SS02406

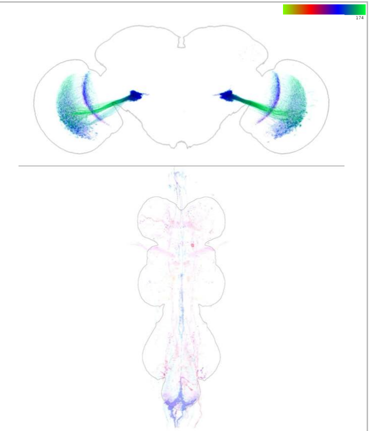
SS02407

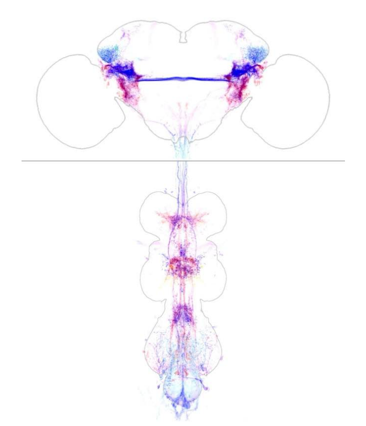
SS02409

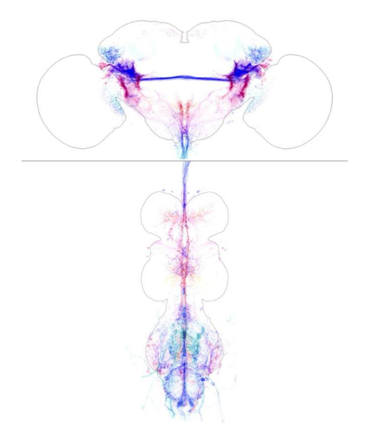
SS02410

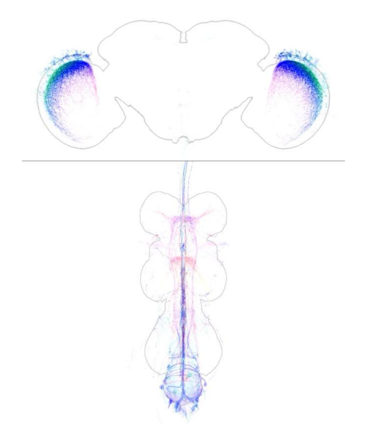
SS02413

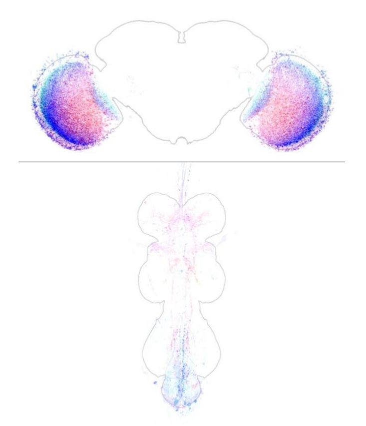
SS02418

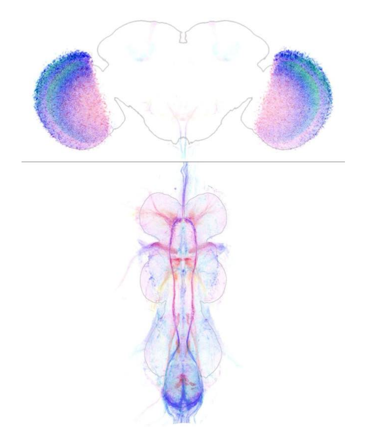
SS02419

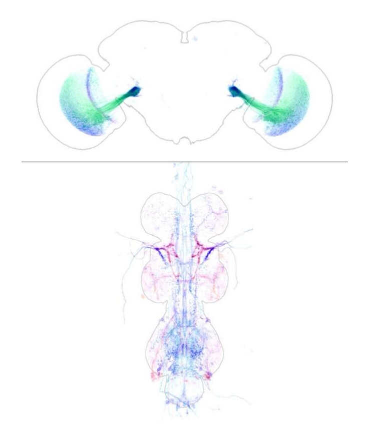
SS02422

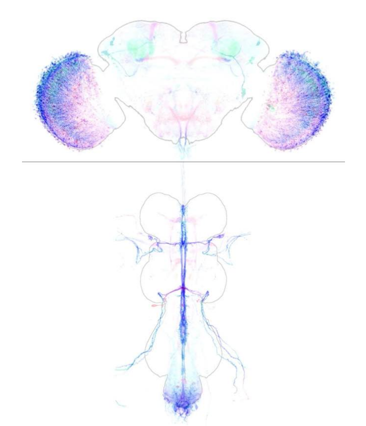
SS02423

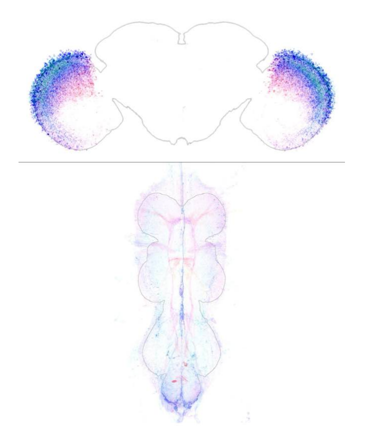
SS02425

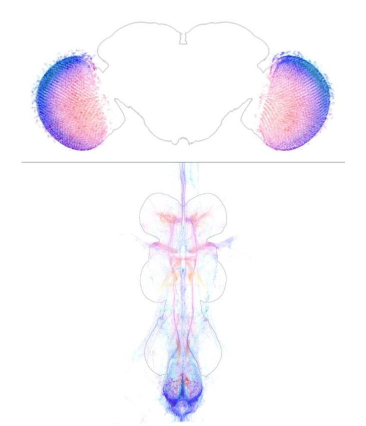
SS02426

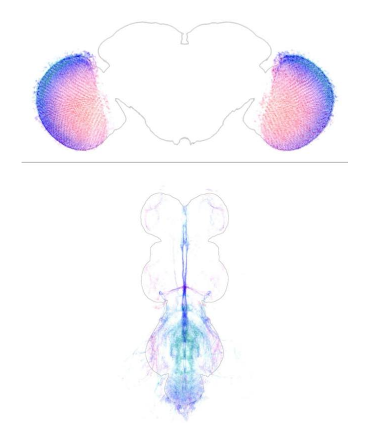
SS02427

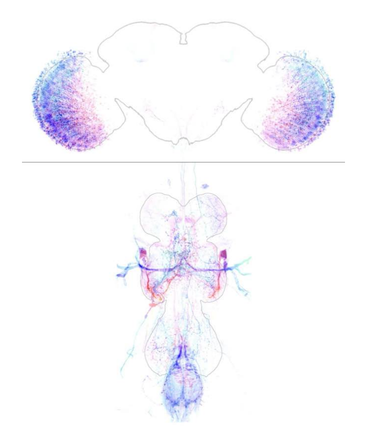
SS02430

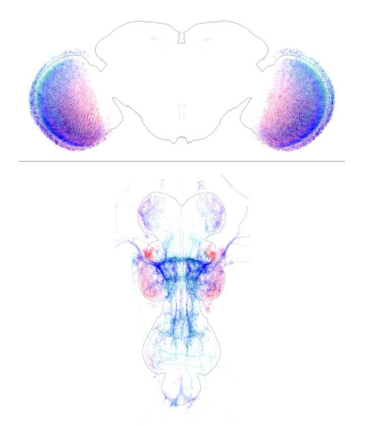
SS02432

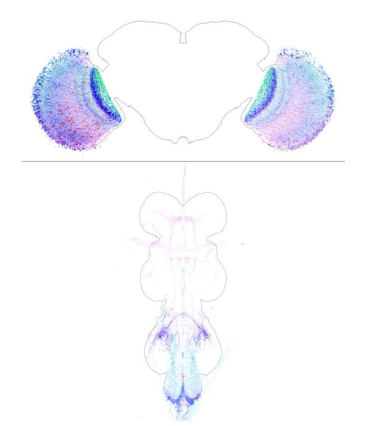
SS02433

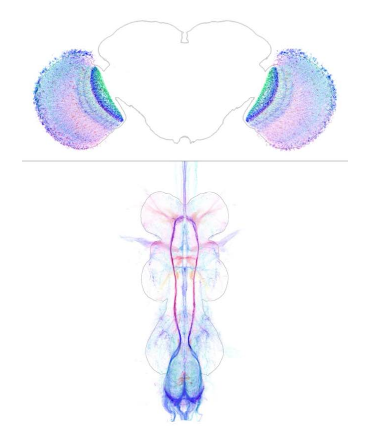
SS02435

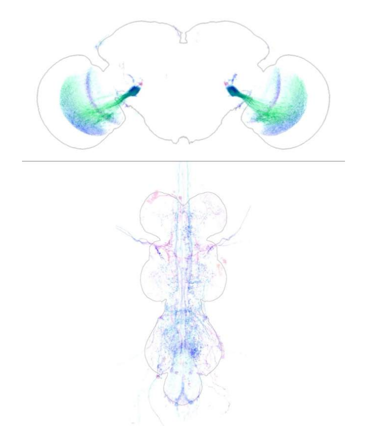
SS02436

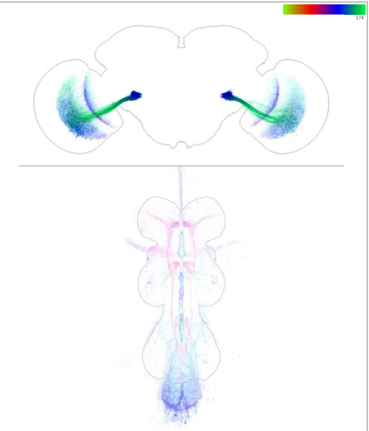
SS02437

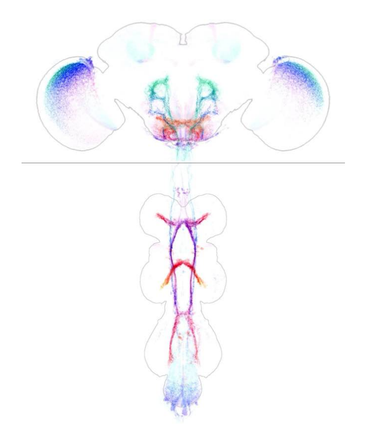
SS02438

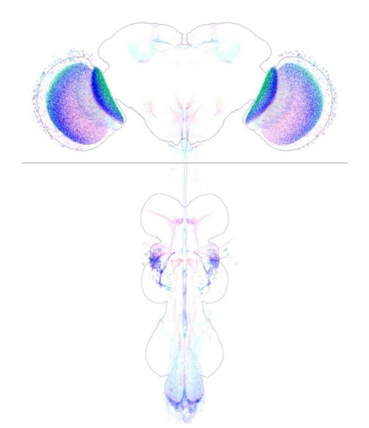
SS02439

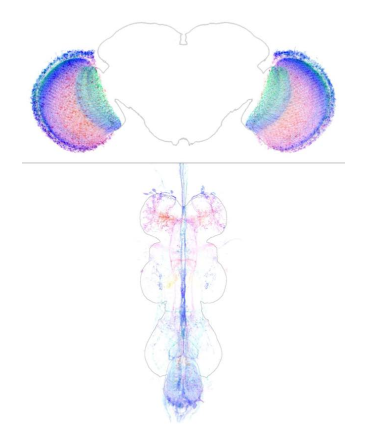
SS02444

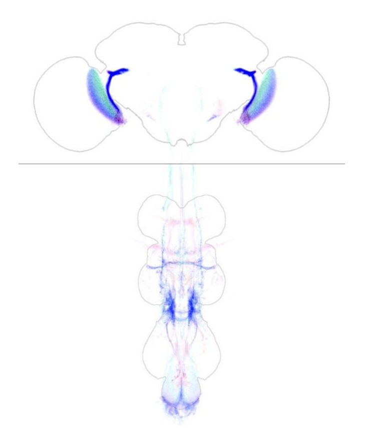
SS02445

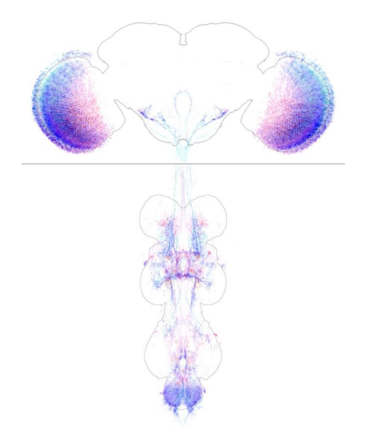
SS02448

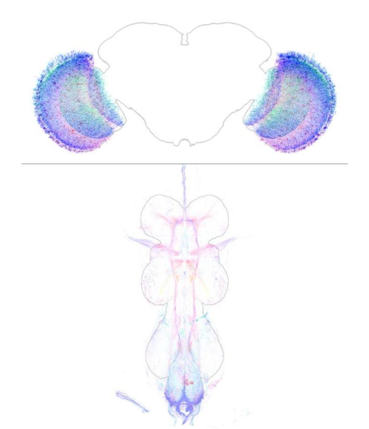
SS02449

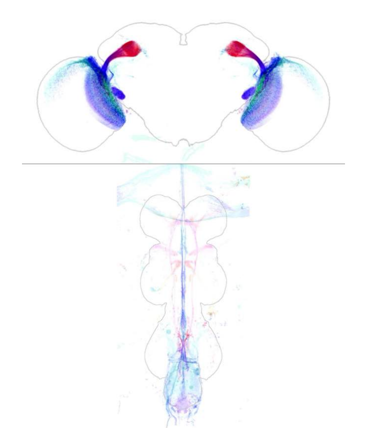
SS02512

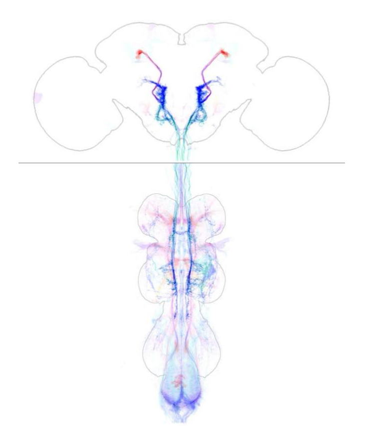
SS02536

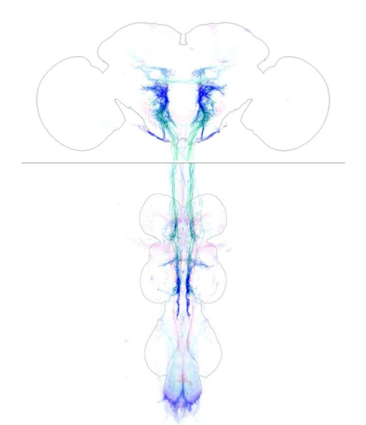
SS02538

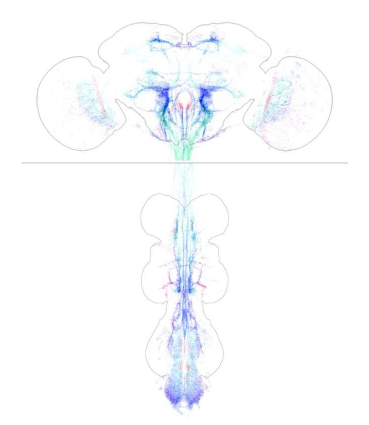
SS02541

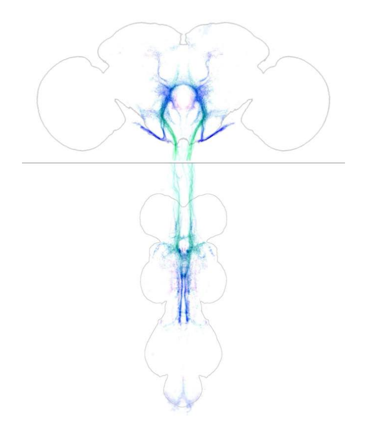
SS02544

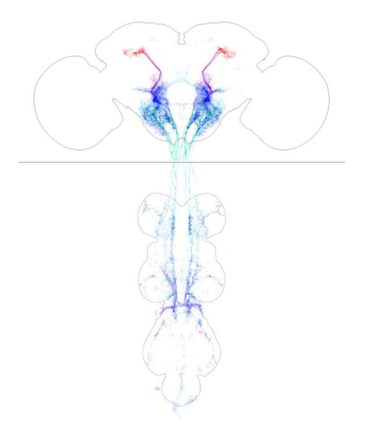
SS02545

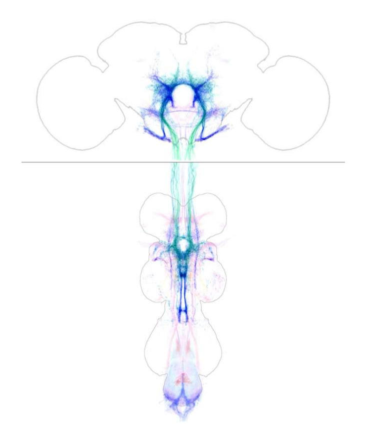
SS02550

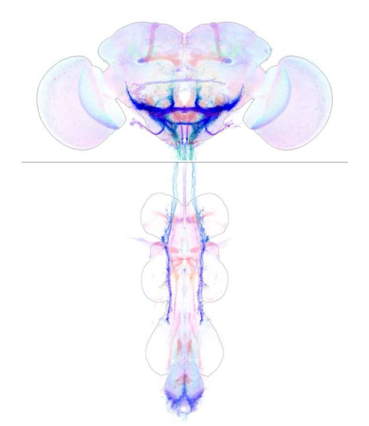
SS02551

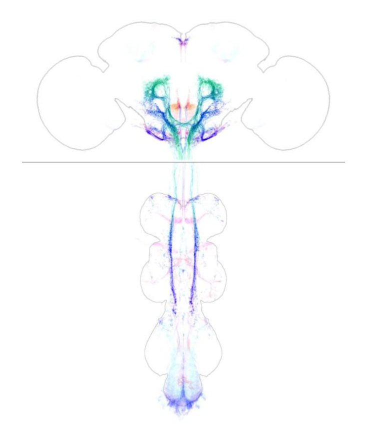
SS02555

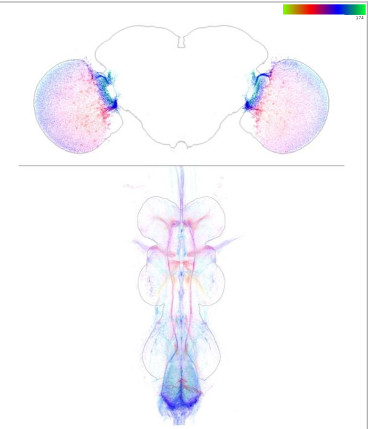

SS02557

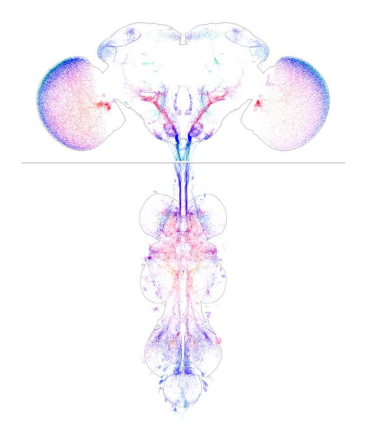

SS02559

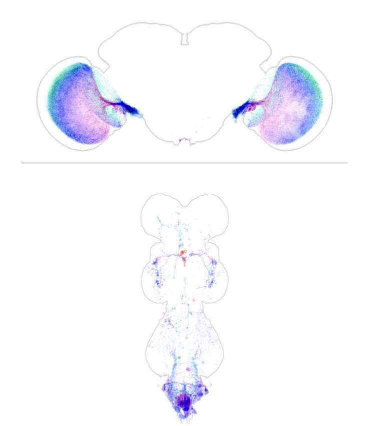

SS02563

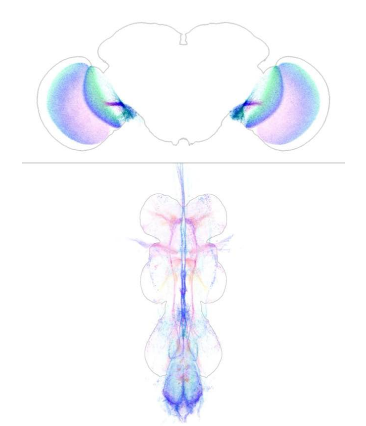

SS02574

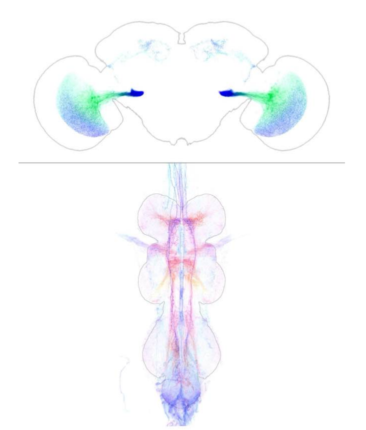

SS02575

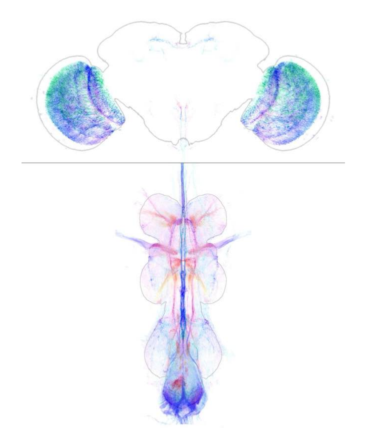

SS02577

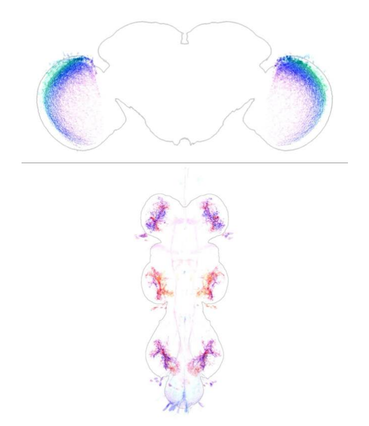

SS02582

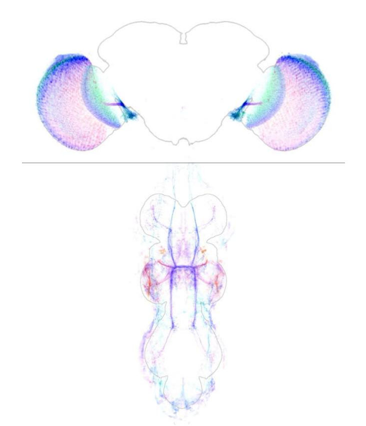

SS02586

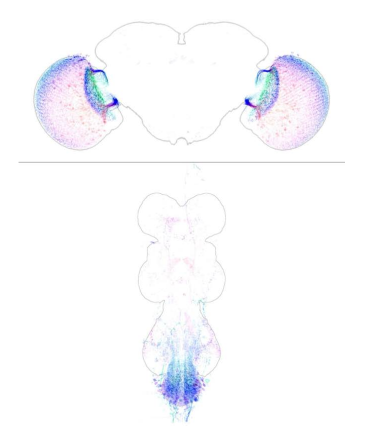

SS02588

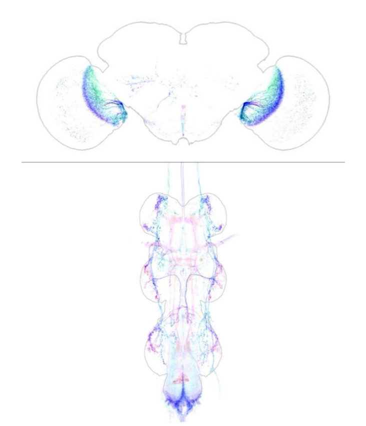

SS02591

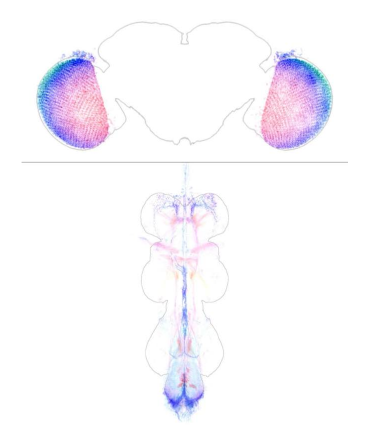

SS02592

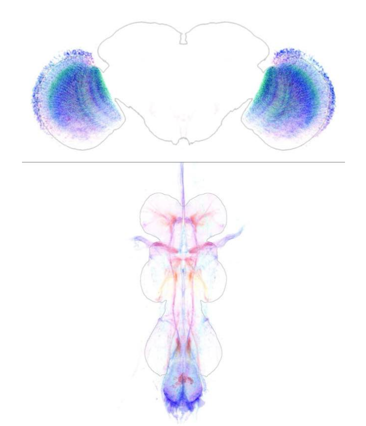

SS02594

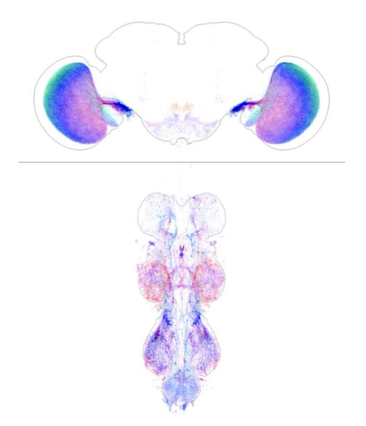

SS02597

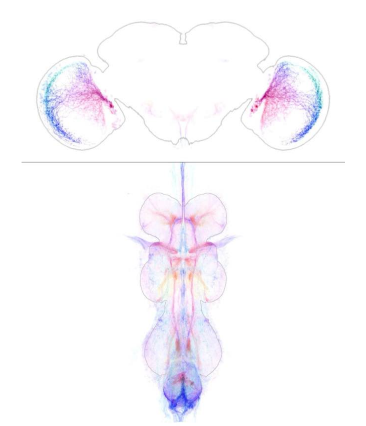

SS02598

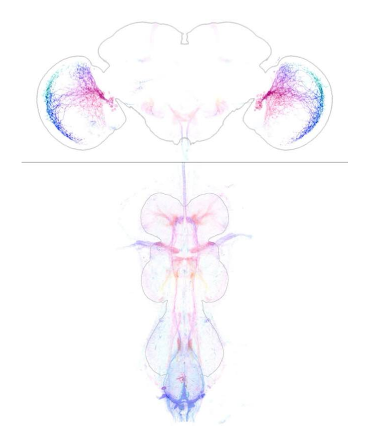

SS02599

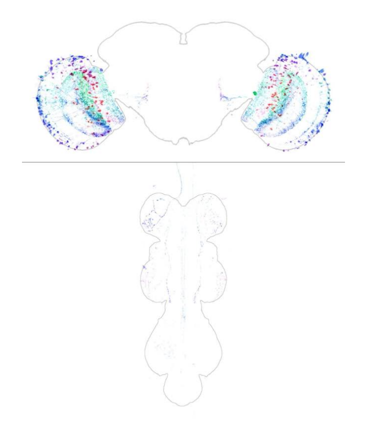

SS02603

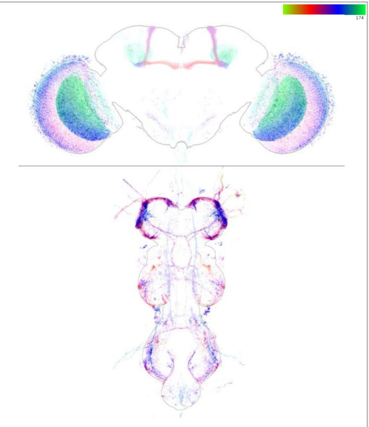
SS02604

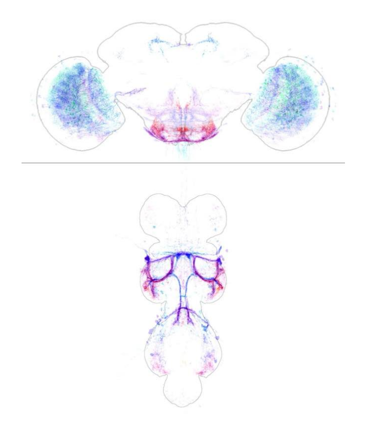
SS02605

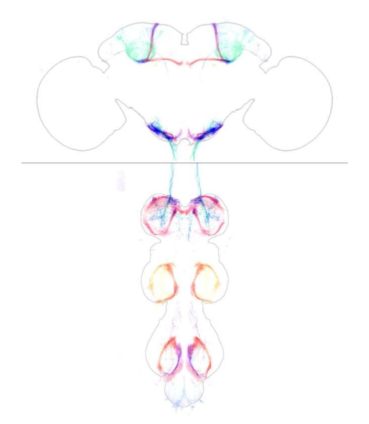
SS02608

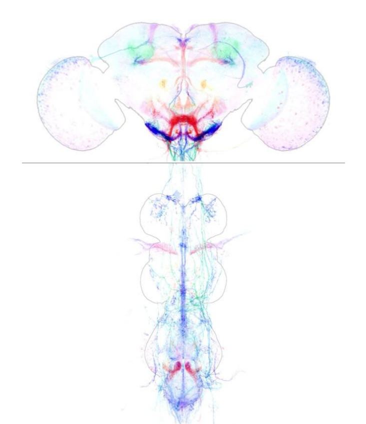
SS02609

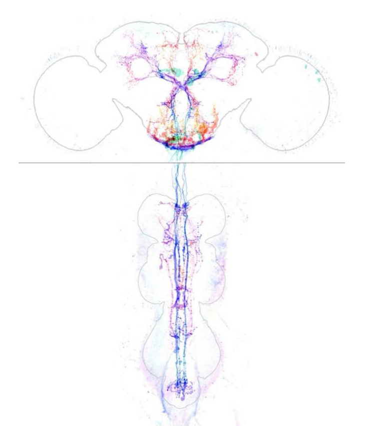
SS02610

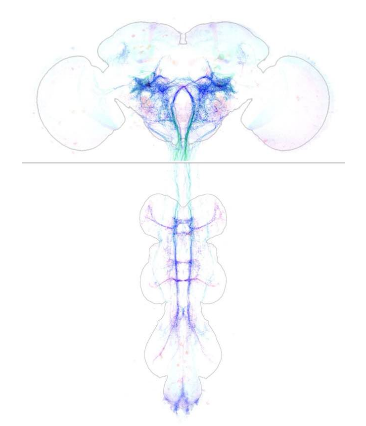
SS02612

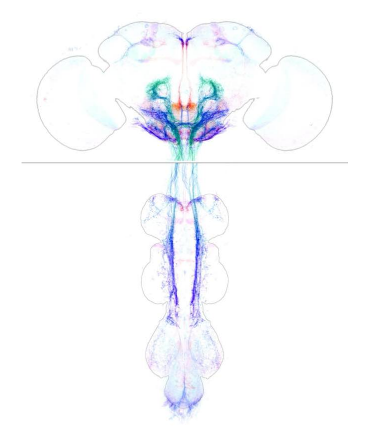
SS02617

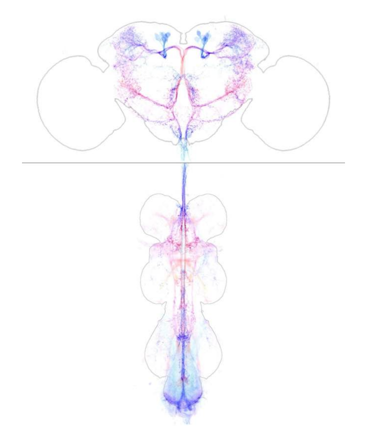
SS02618

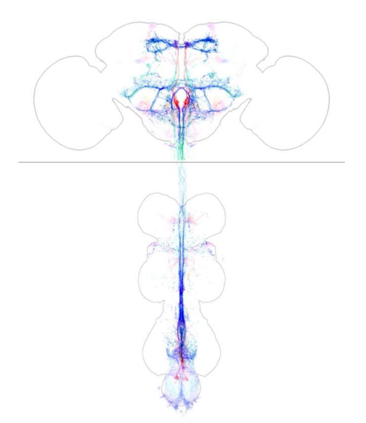
SS02621

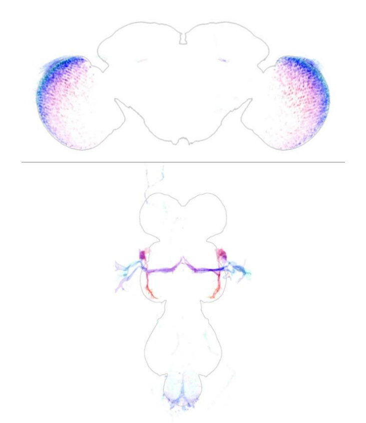
SS02623

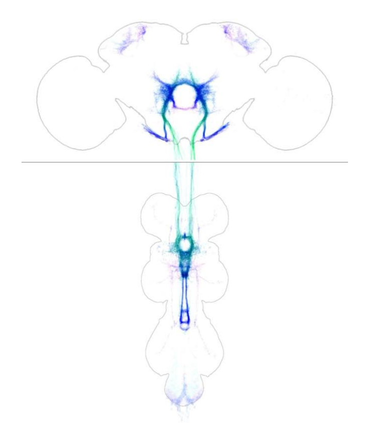
SS02624

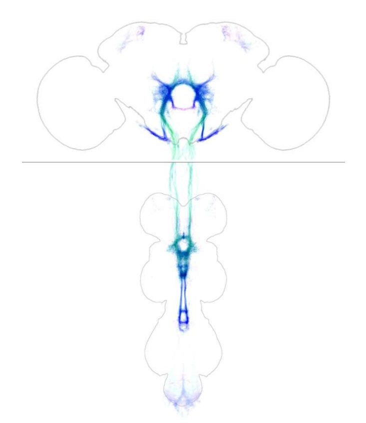
SS02625

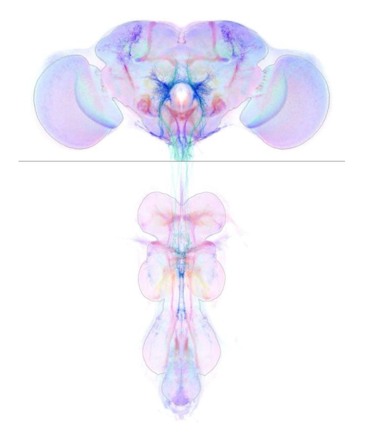
SS02627

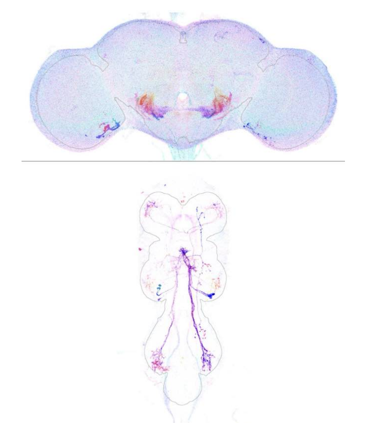
SS02628

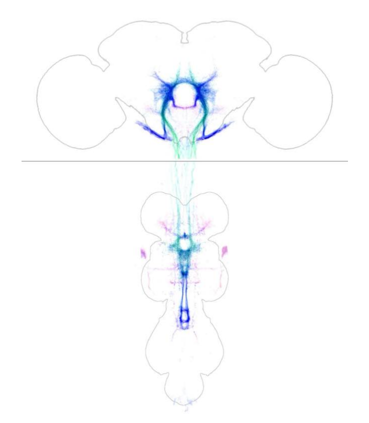
SS02630

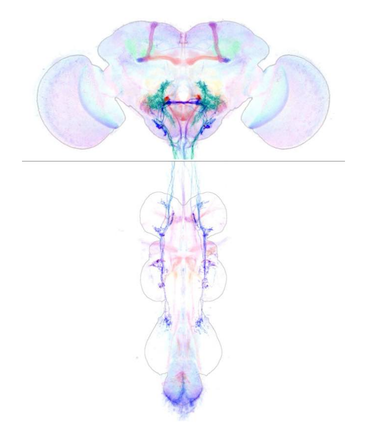
SS02631

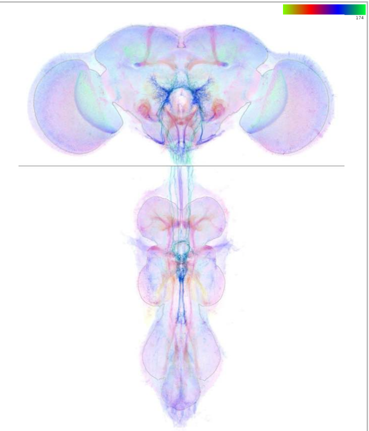
SS02634

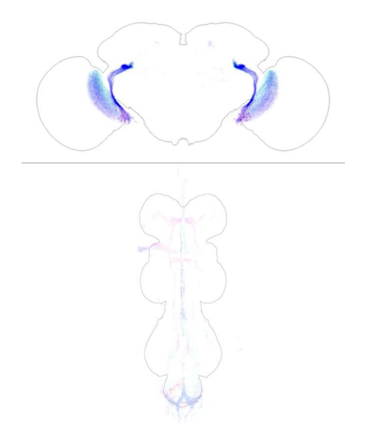
SS02638

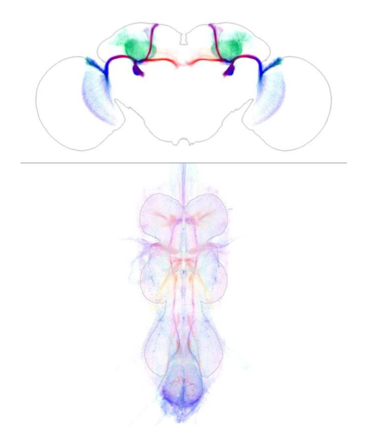
SS02645

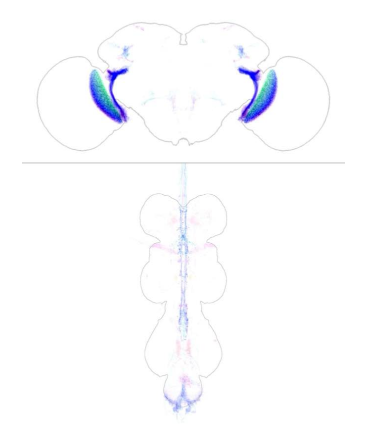
SS02650

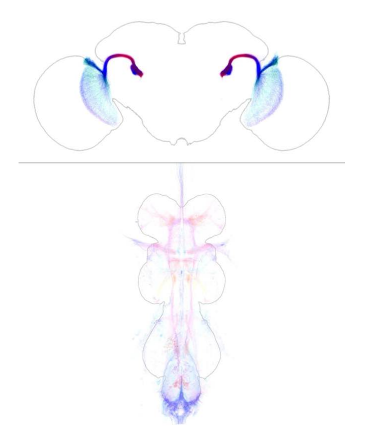
SS02651

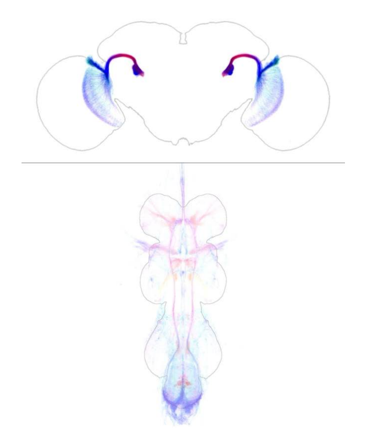
SS02652

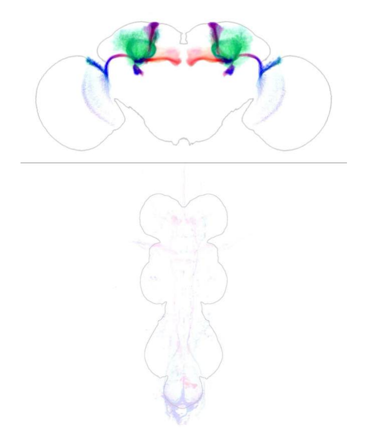
SS02662

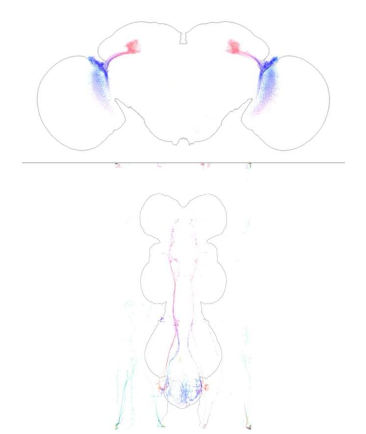
SS02663

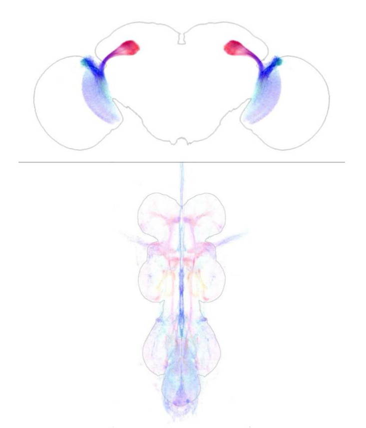
SS02664

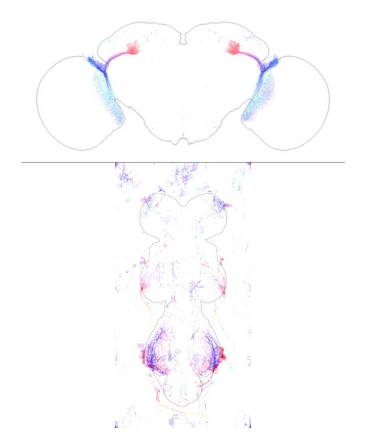
SS02665

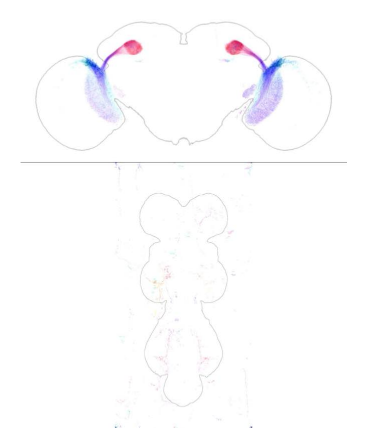
SS02666

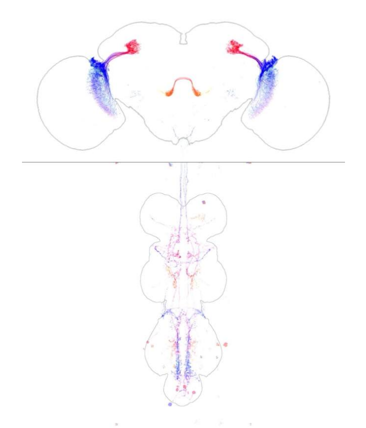
SS02669

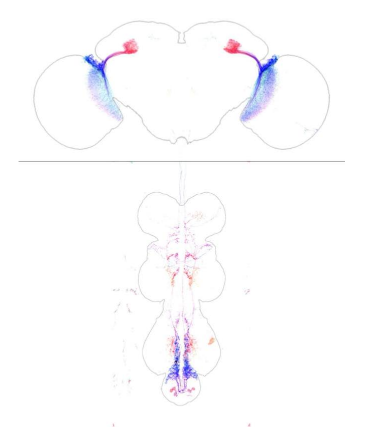
SS02681

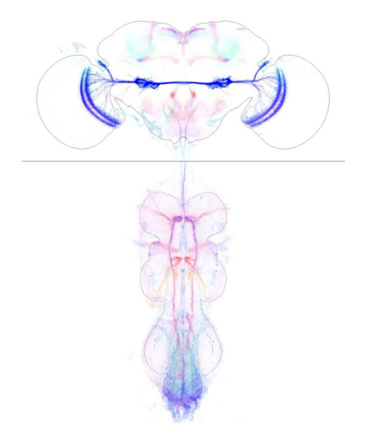
SS02691

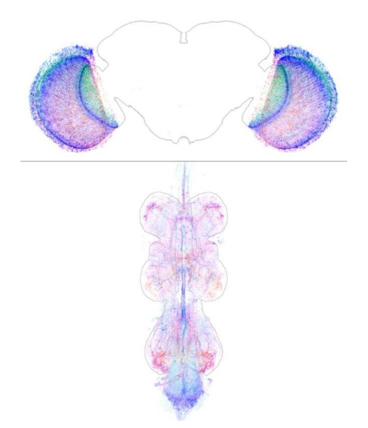
SS02692

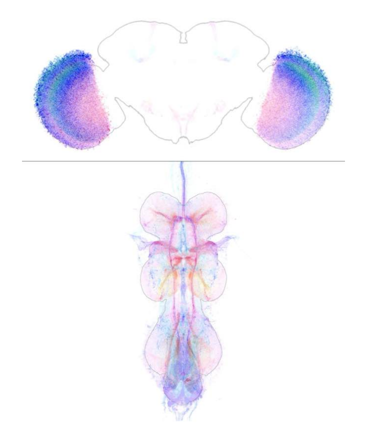
SS02693

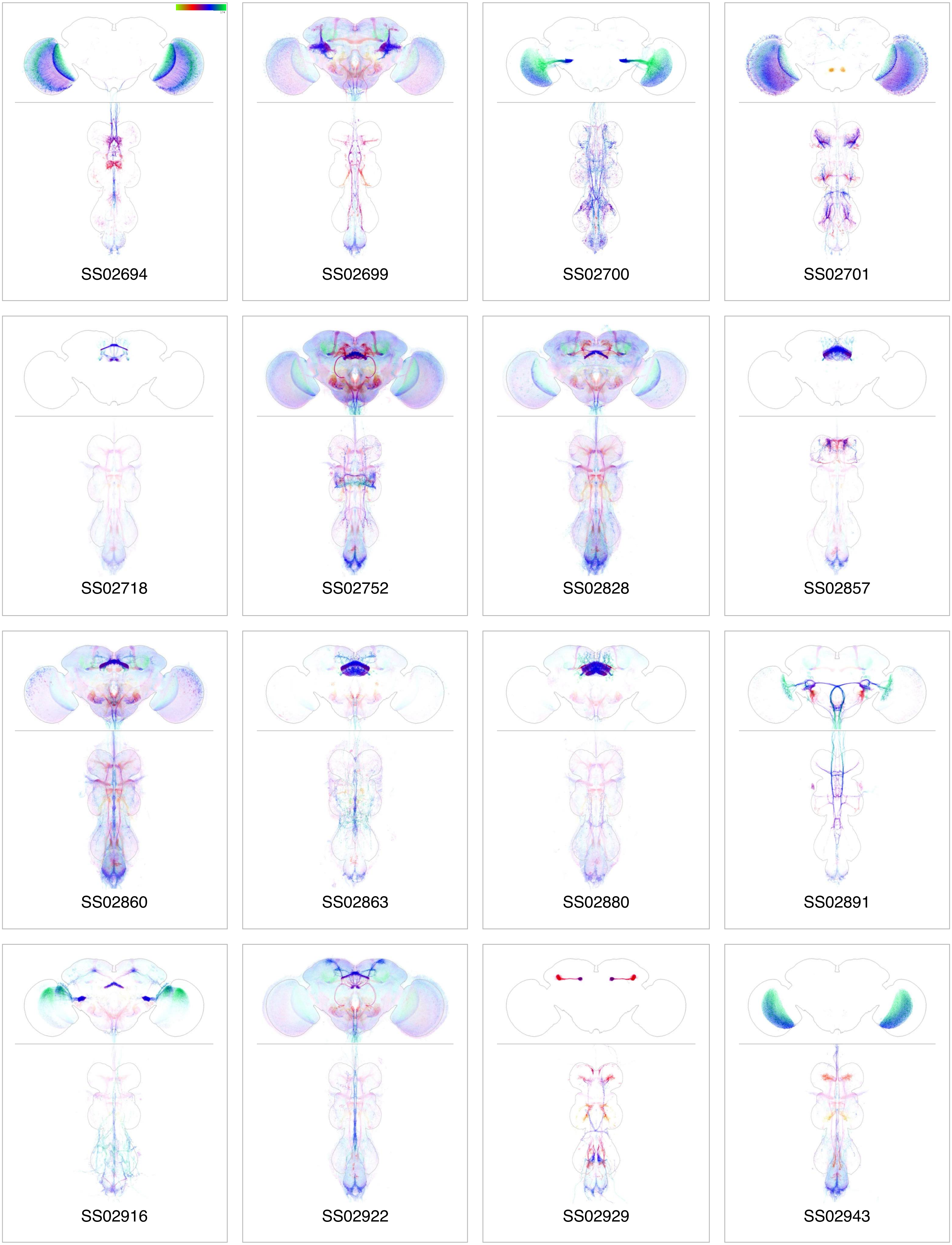
SS02694
SS02699
SS02700
SS02701
SS02718
SS02752
SS02828
SS02857
SS02860
SS02863
SS02880
SS02891
SS02916
SS02922
SS02929
SS02943

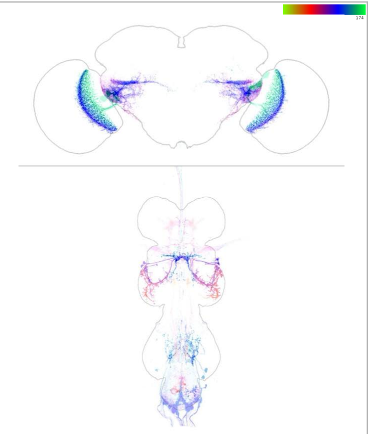
SS02945

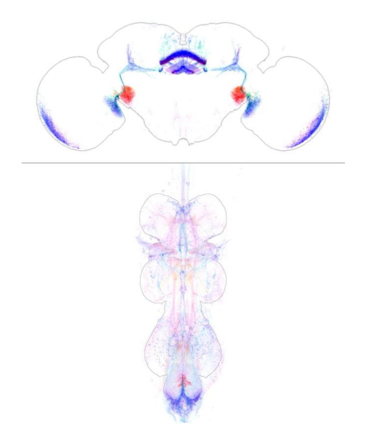
SS02950

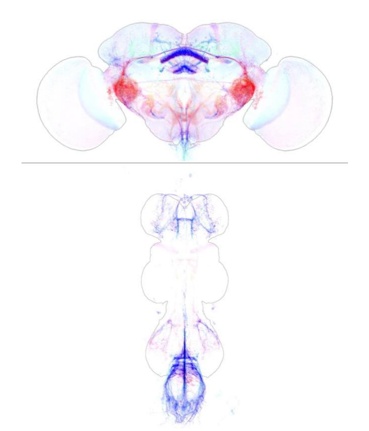
SS02951

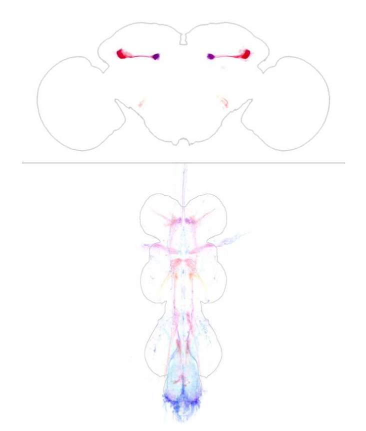
SS02974

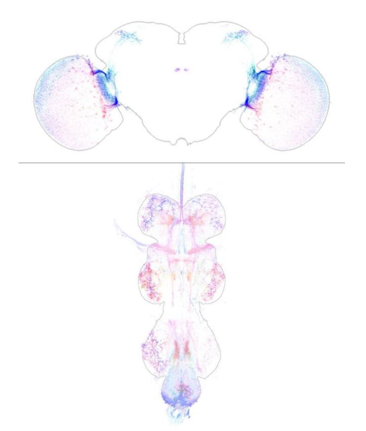
SS02978

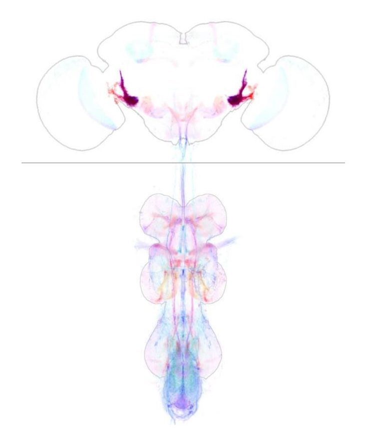
SS02996

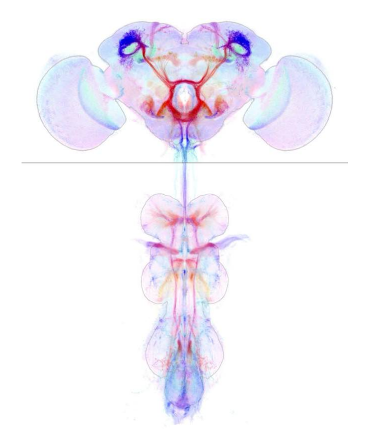
SS03049

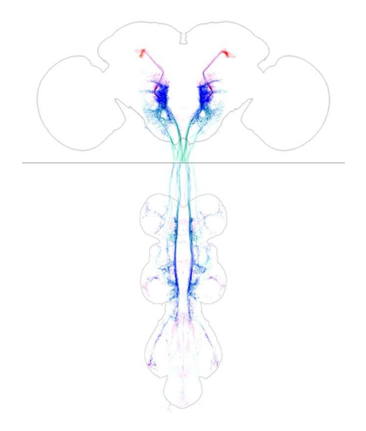
SS03103

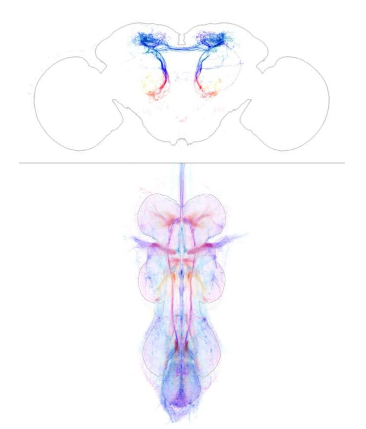
SS03112

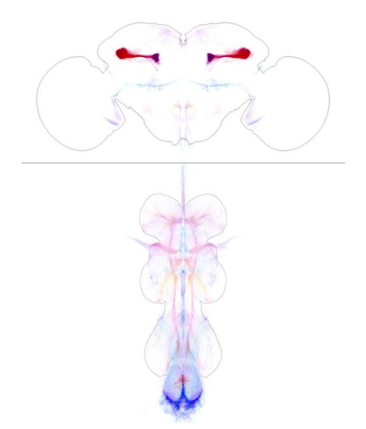
SS03144

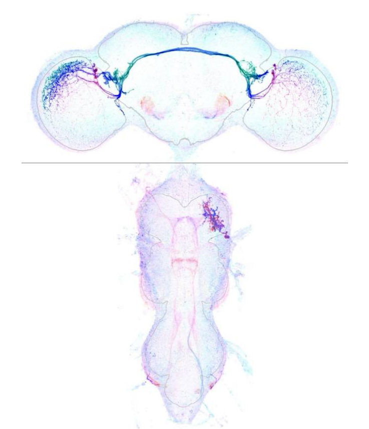
SS03149

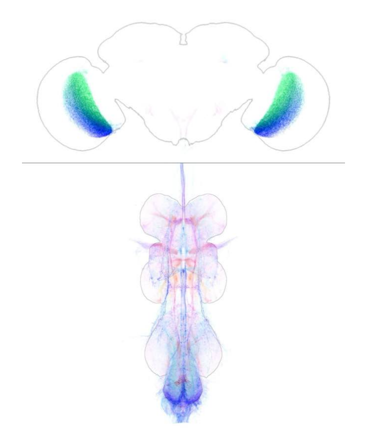
SS03151

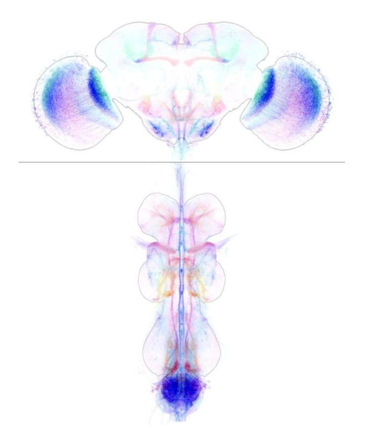
SS03152

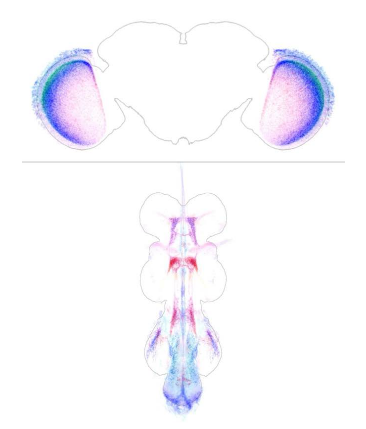
SS03159

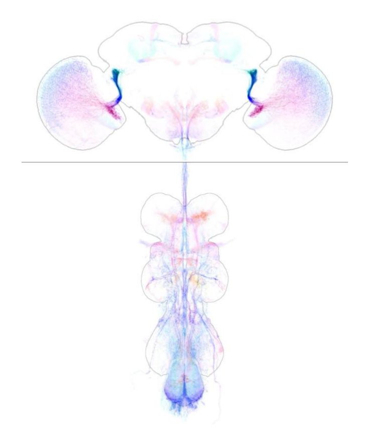
SS03162

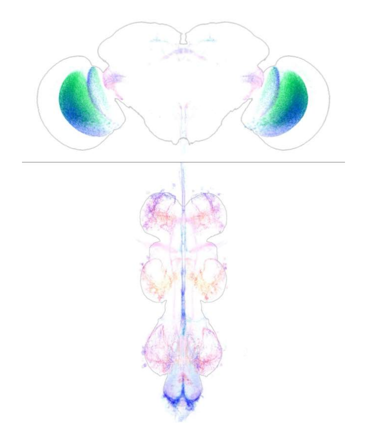
SS03163

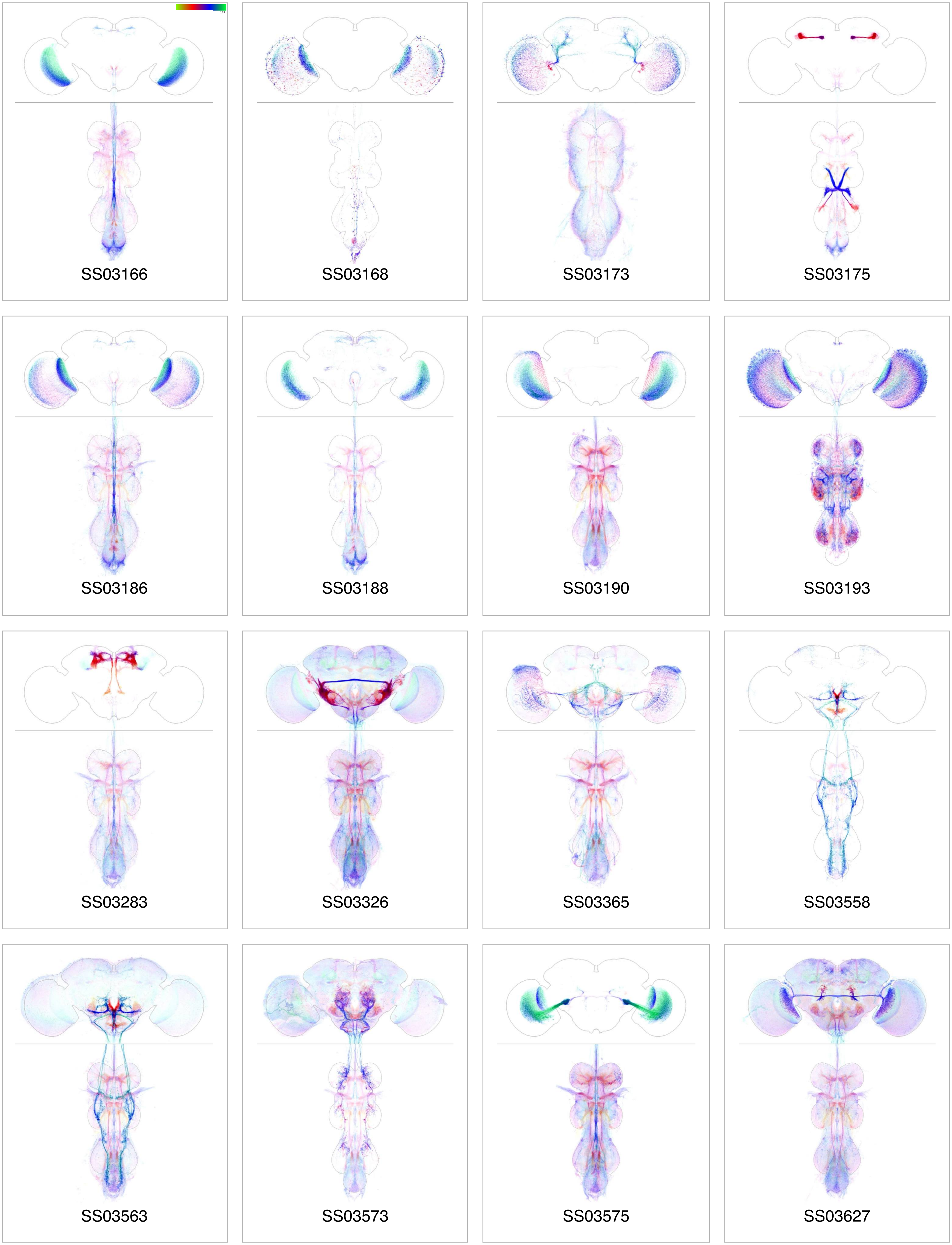
SS03166
SS03168
SS03173
SS03175
SS03186
SS03188
SS03190
SS03193
SS03283
SS03326
SS03365
SS03558
SS03563
SS03573
SS03575
SS03627

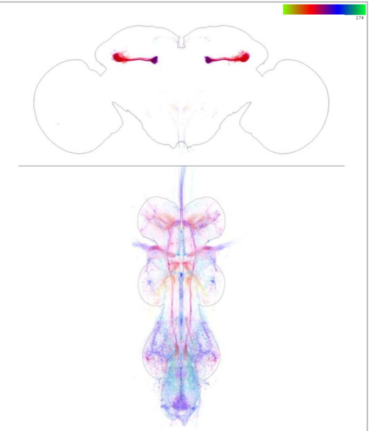

SS03640

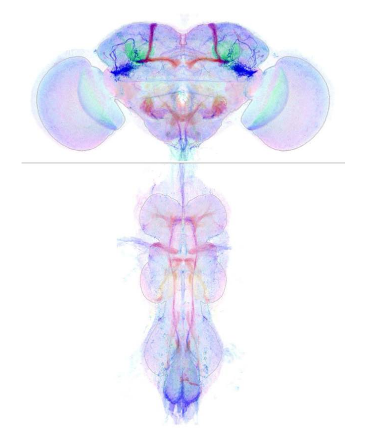

SS03641

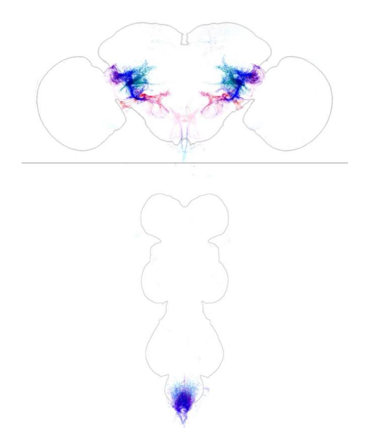

SS03644

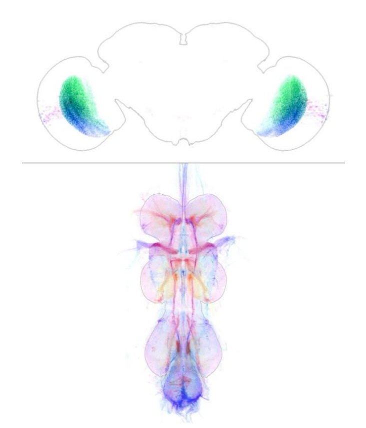

SS03656

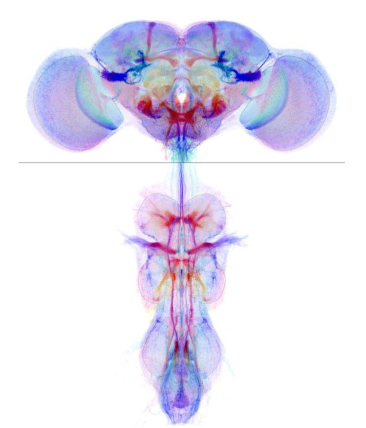

SS03658

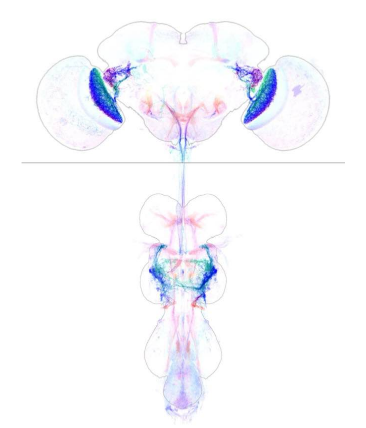

SS03659

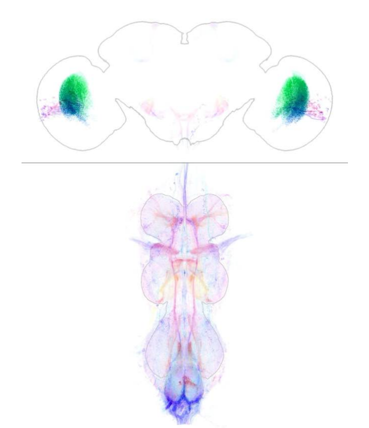

SS03679

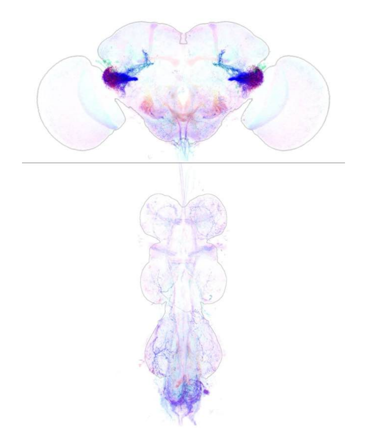

SS03689

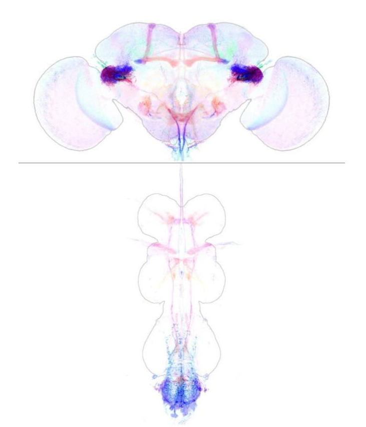

SS03690

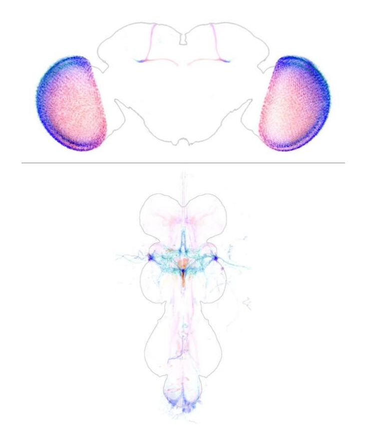

SS03696

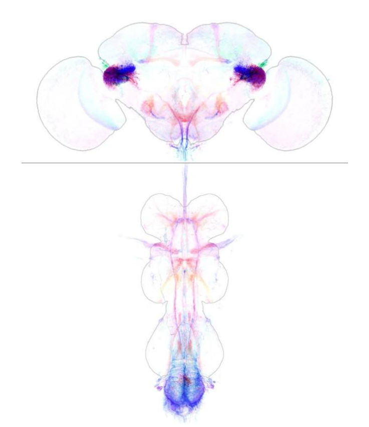

SS03704

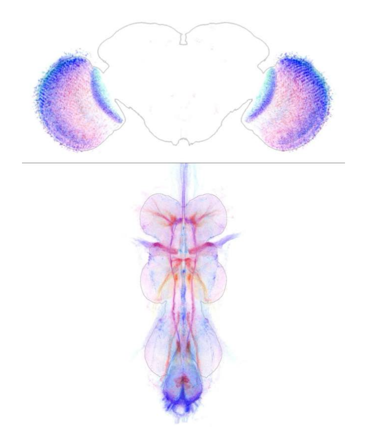

SS03711

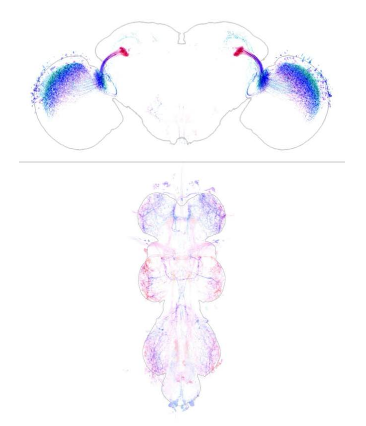

SS03713

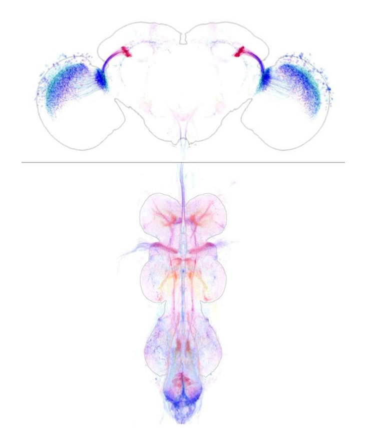

SS03719

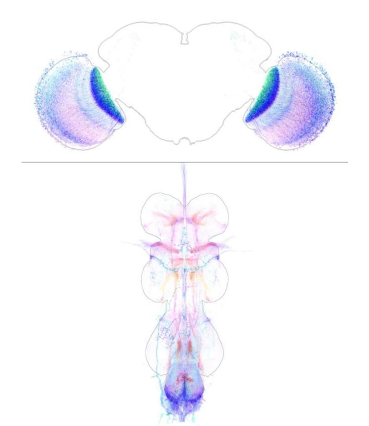

SS03722

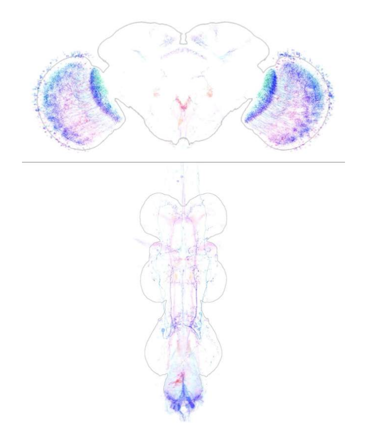

SS03729

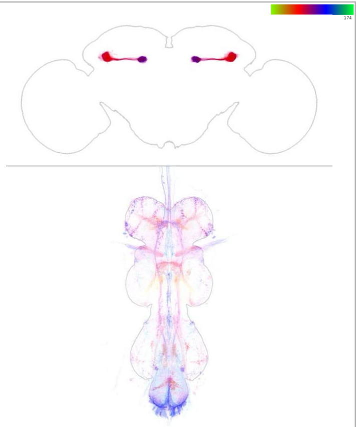
SS03730

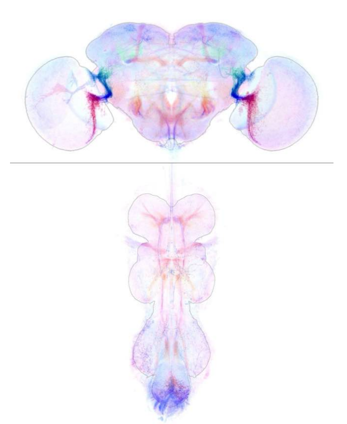
SS03735

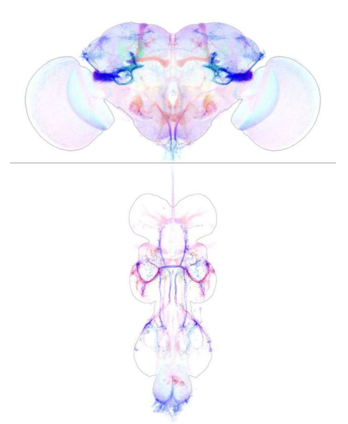
SS03737

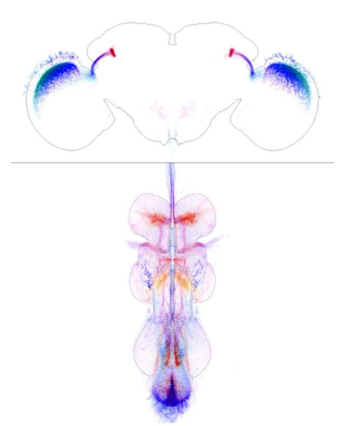
SS03739

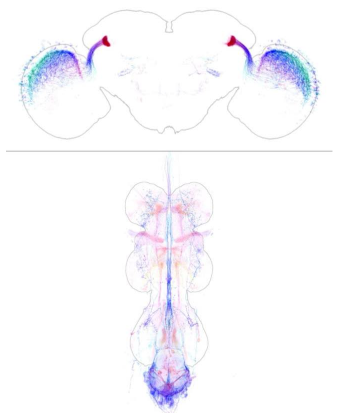
SS03740

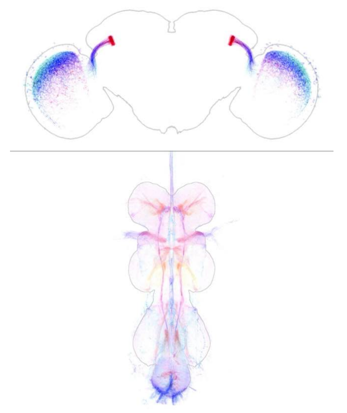
SS03741

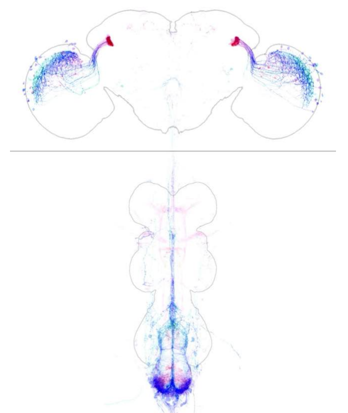
SS03744

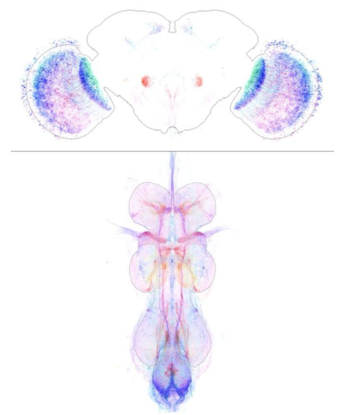
SS03745

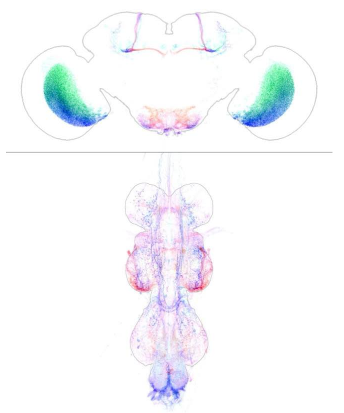
SS03752

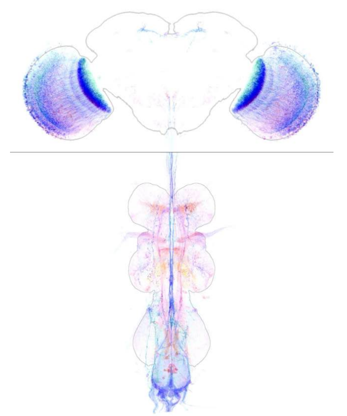
SS03753

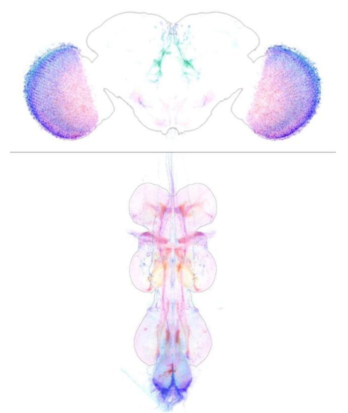
SS03763

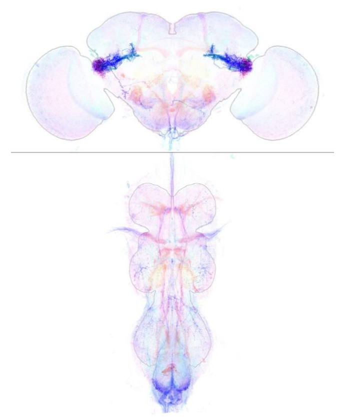
SS03765

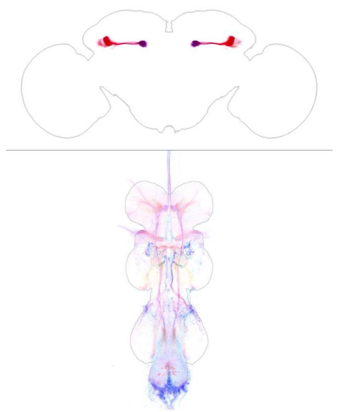
SS03805

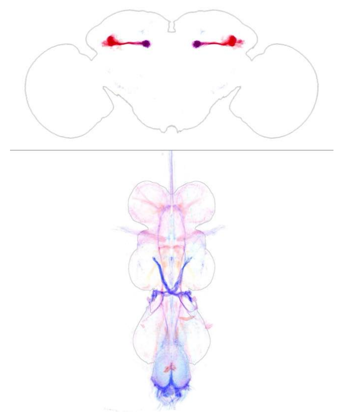
SS03806

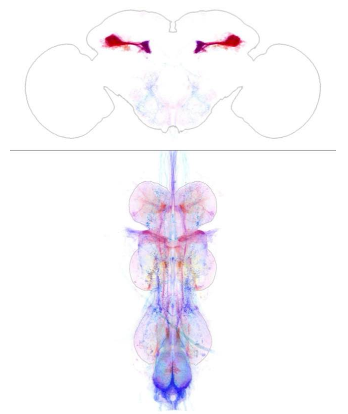
SS03807

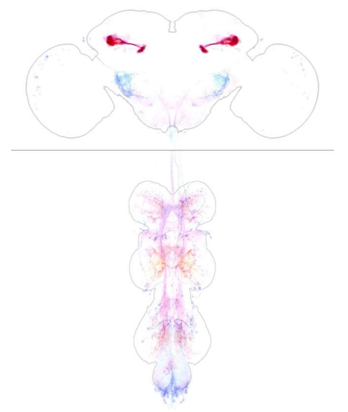
SS03808

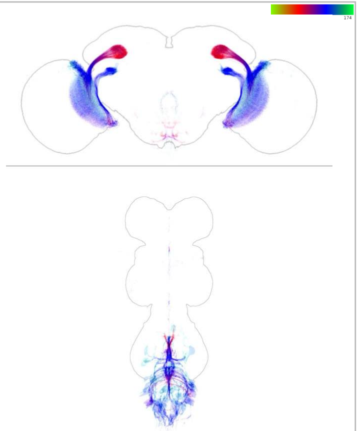

SS03815

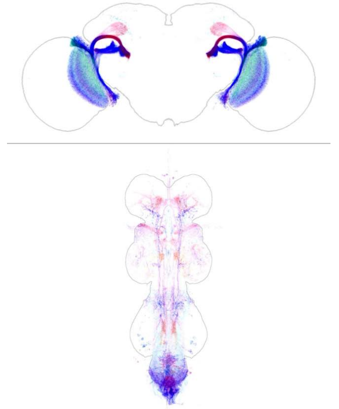

SS03816

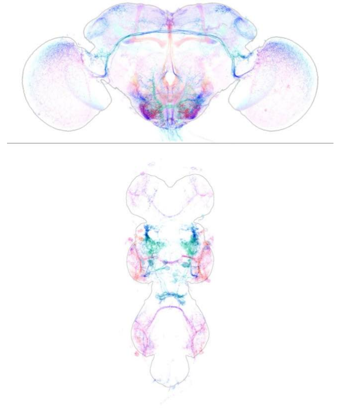

SS03817

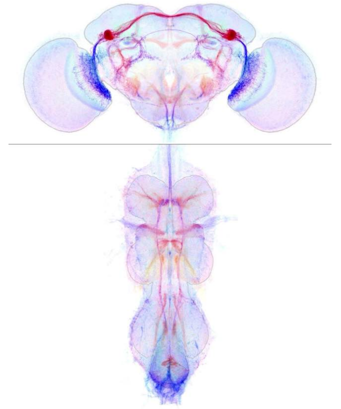

SS03819

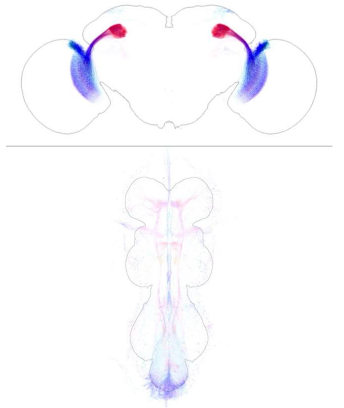

SS03822

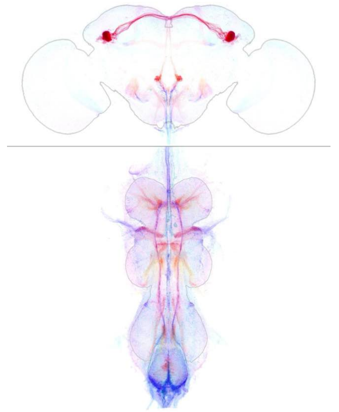

SS03825

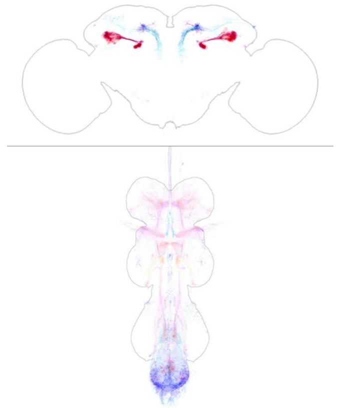

SS03828

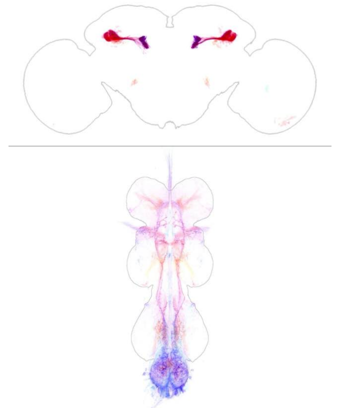

SS03831

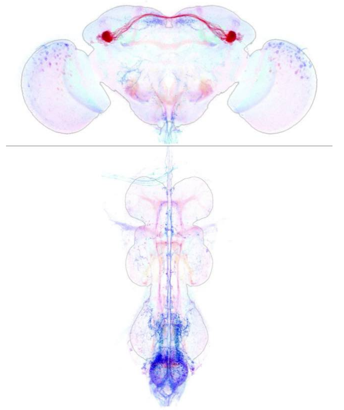

SS03832

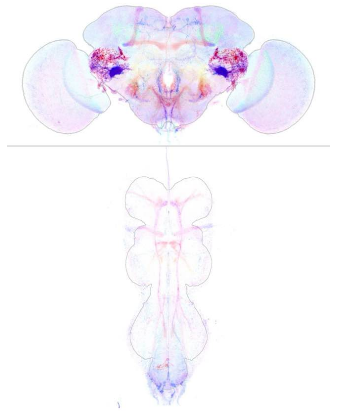

SS03838

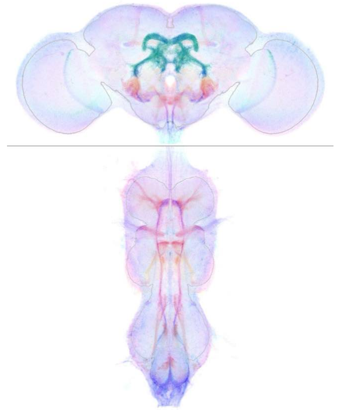

SS03951

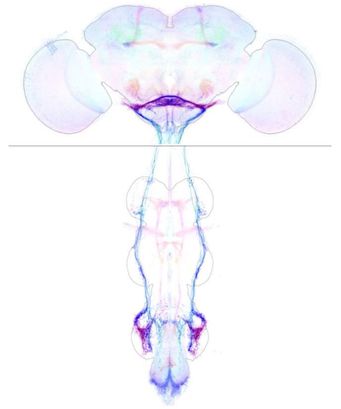

SS04158

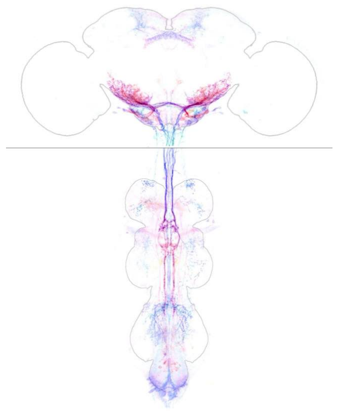

SS04159

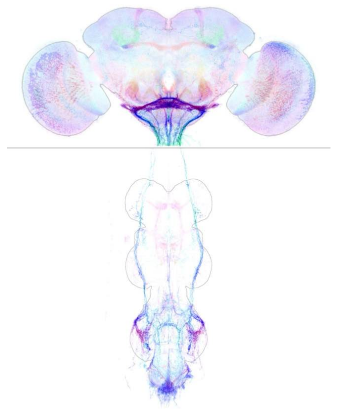

SS04160

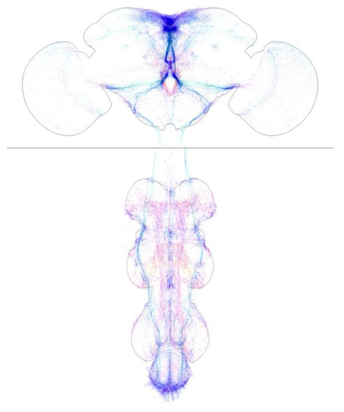

SS04161

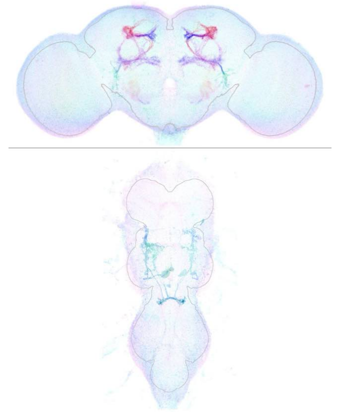

SS04259

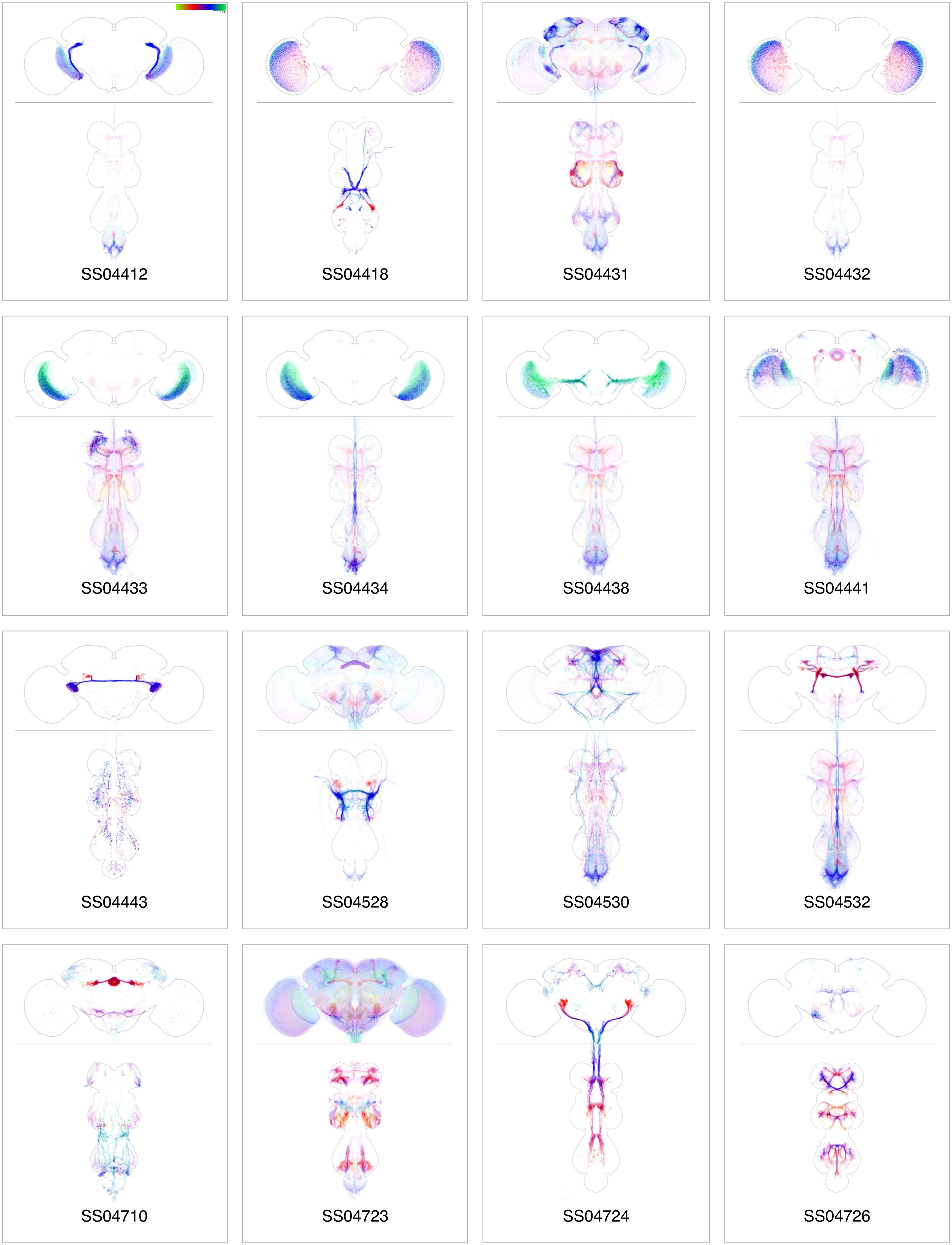
SS04412
SS04418
SS04431
SS04432
SS04433
SS04434
SS04438
SS04441
SS04443
SS04528
SS04530
SS04532
SS04710
SS04723
SS04724
SS04726

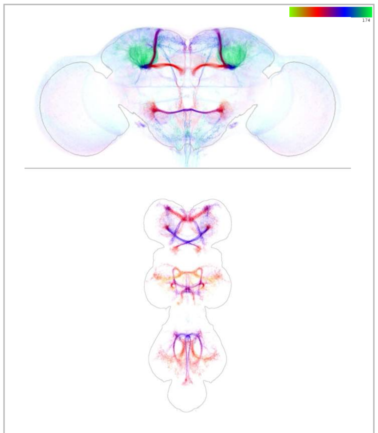

SS04728

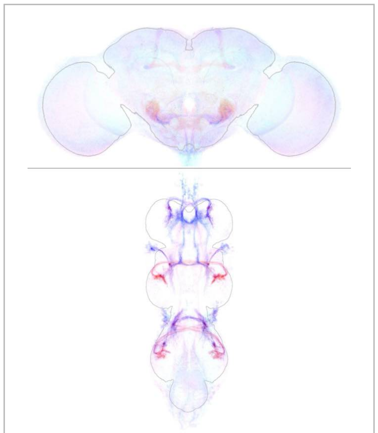

SS04730

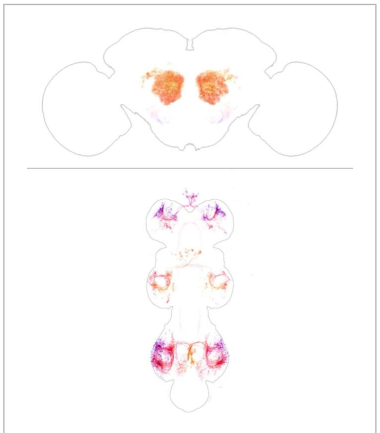

SS04732

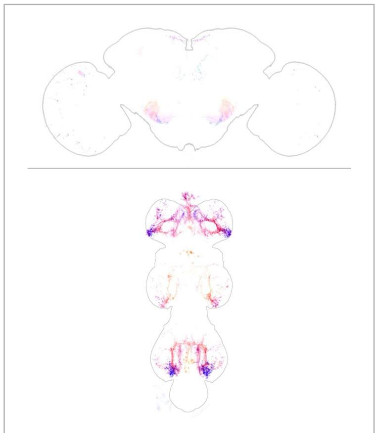

SS04733

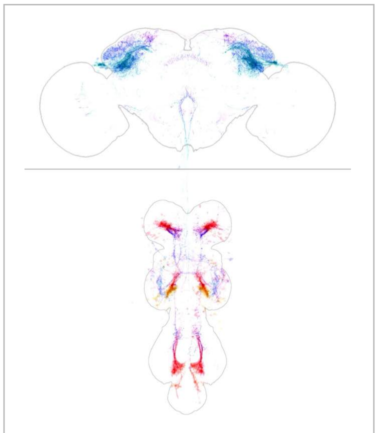

SS04736

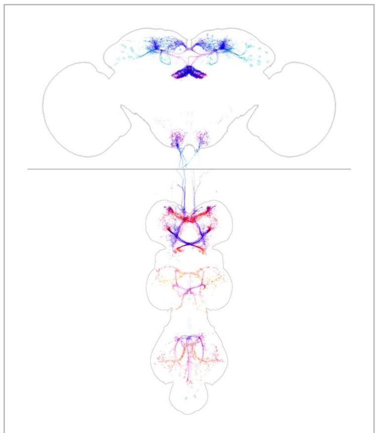

SS04738

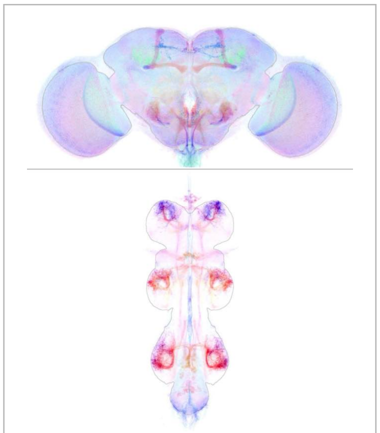

SS04739

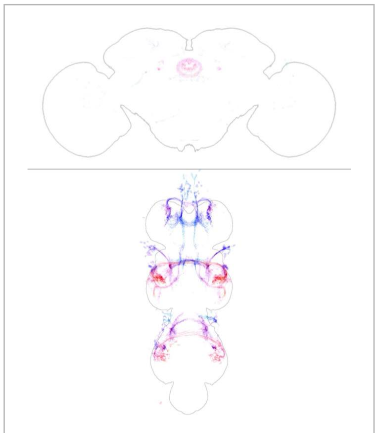

SS04741

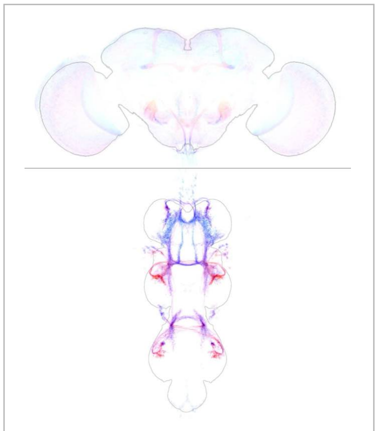

SS04742

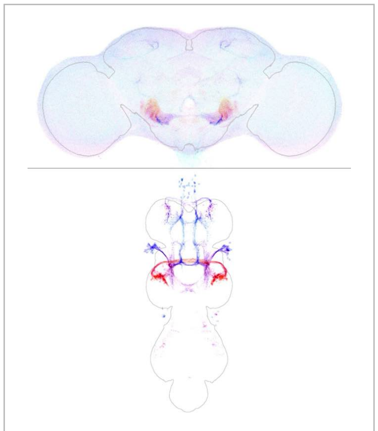

SS04744

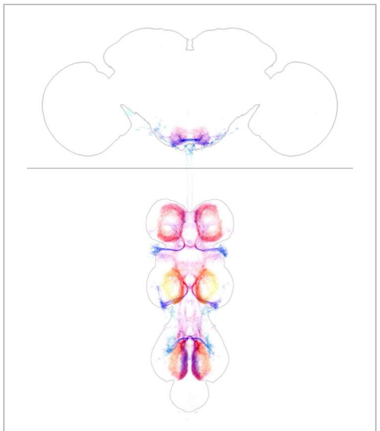

SS04745

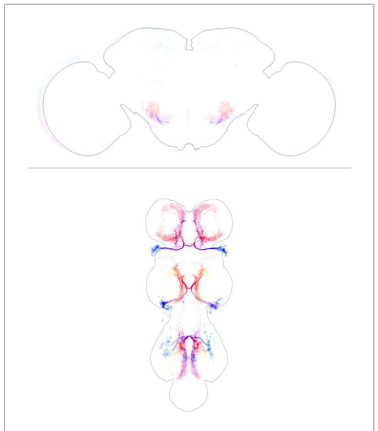

SS04746

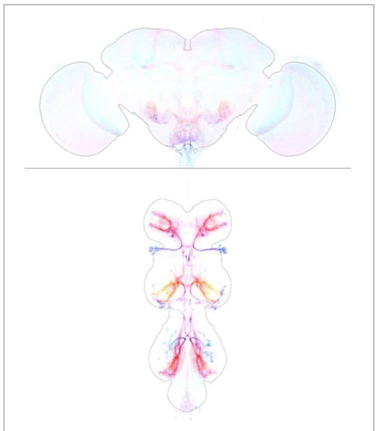

SS04747

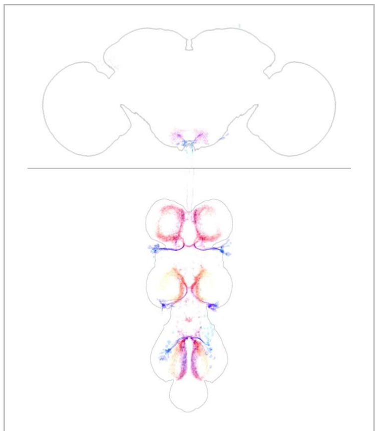

SS04748

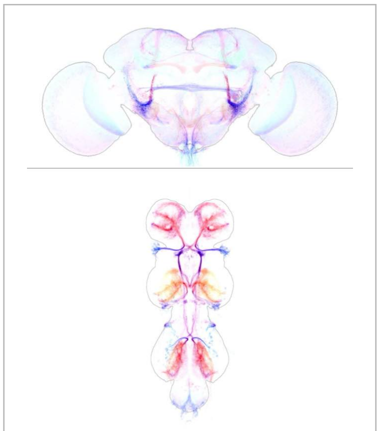

SS04749

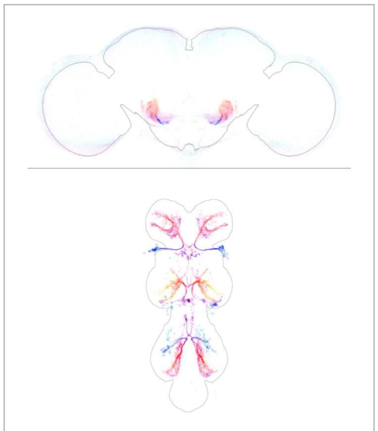

SS04750

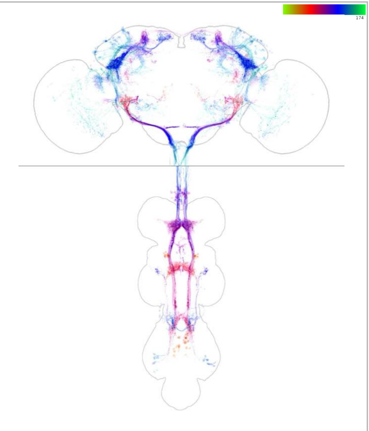
SS04751
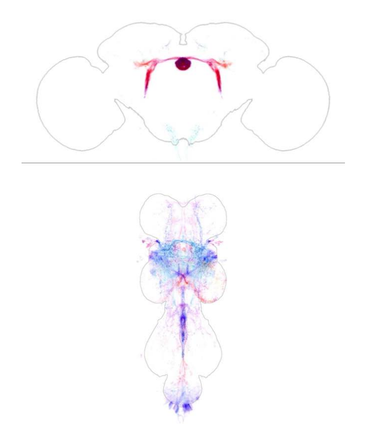
SS04764
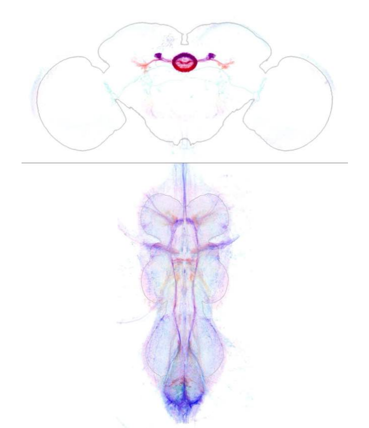
SS04772
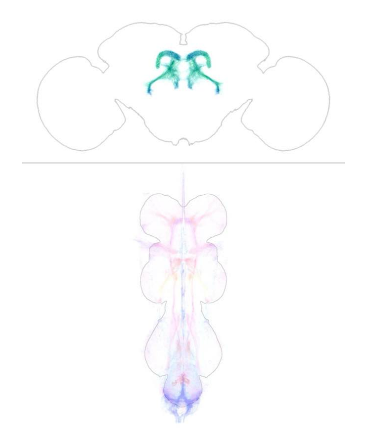
SS04778
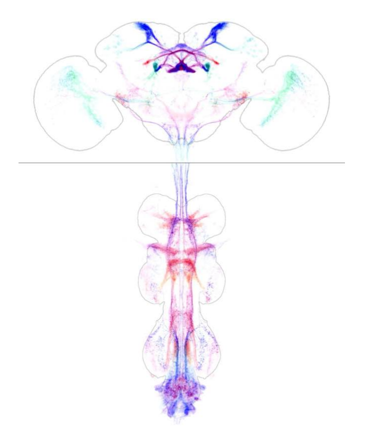
SS04782
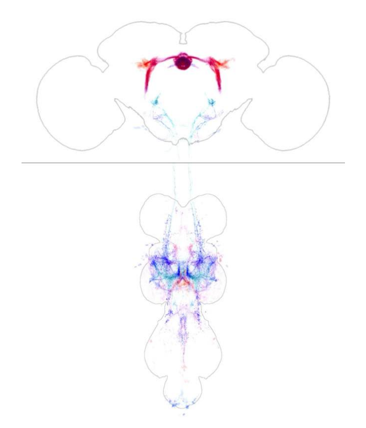
SS04804
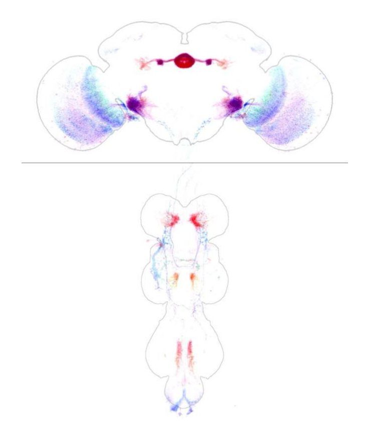
SS04815
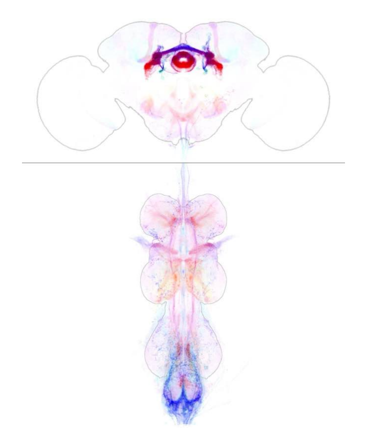
SS04830
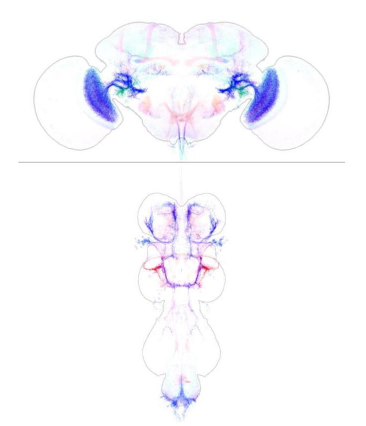
SS04920
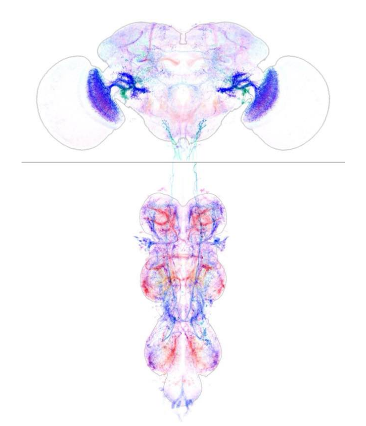
SS04922
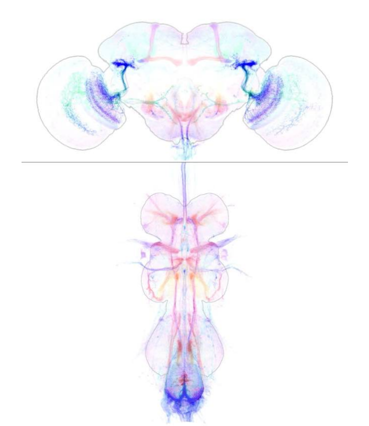
SS04924
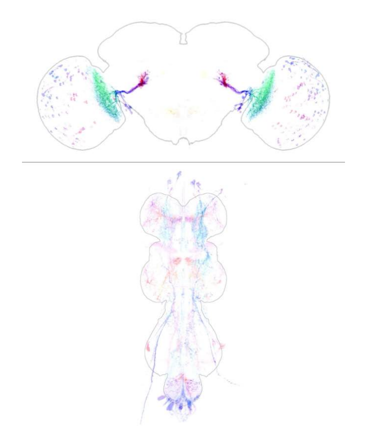
SS04926
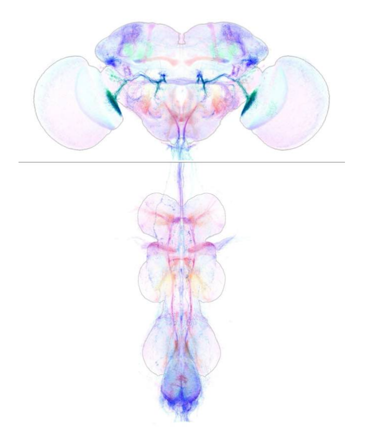
SS04979
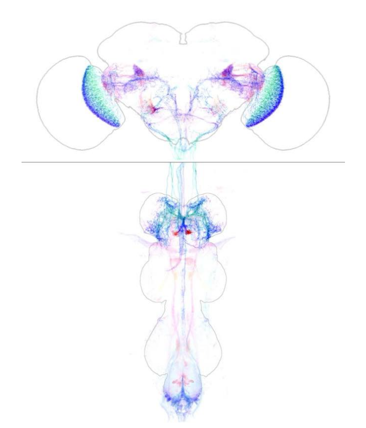
SS05005
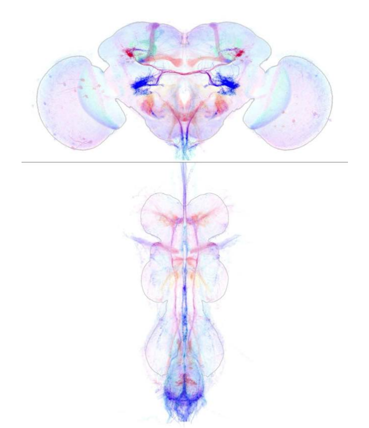
SS05012
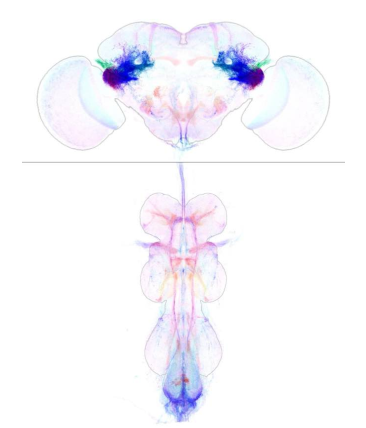
SS05018

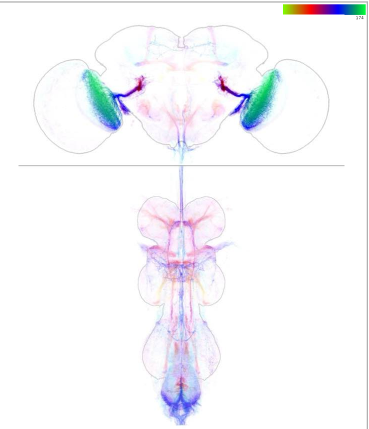

SS05022

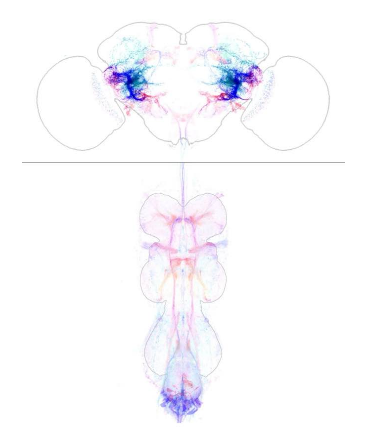

SS05024

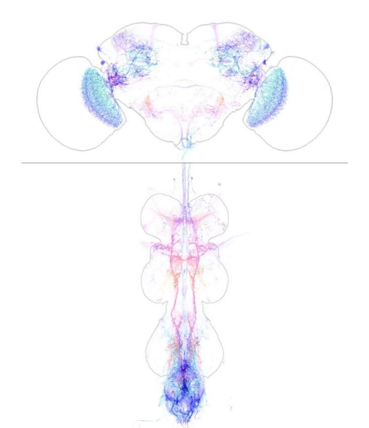

SS05027

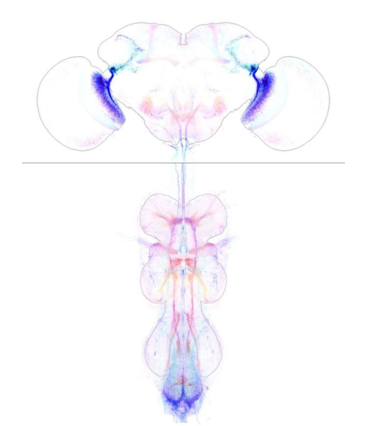

SS05028

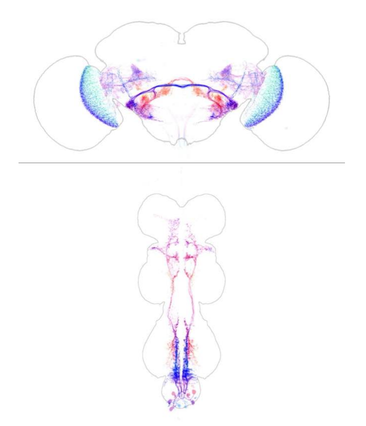

SS05033

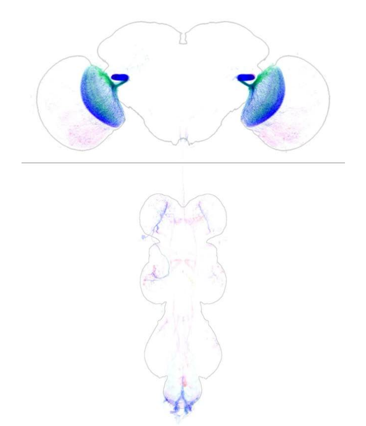

SS05037

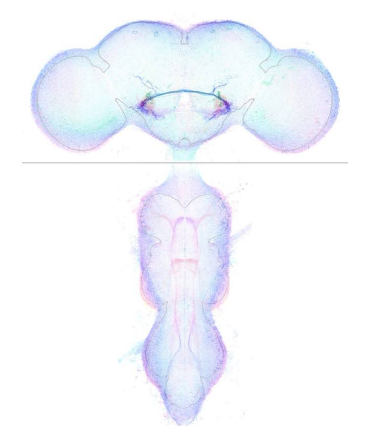

SS05077

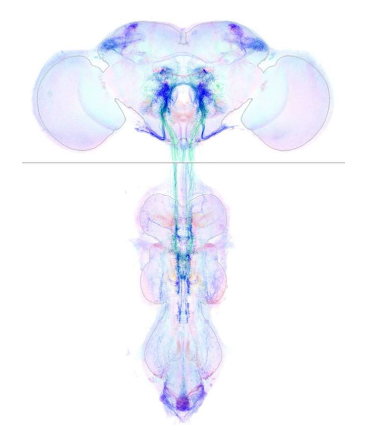

SS05089

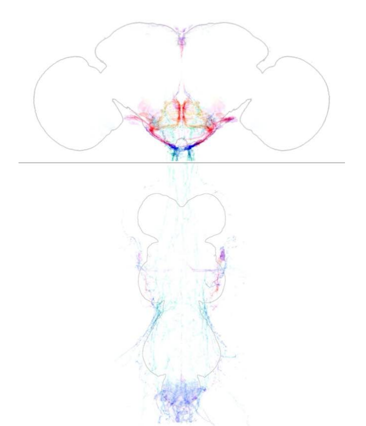

SS05099

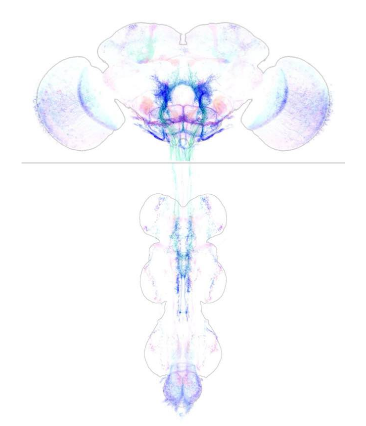

SS05107

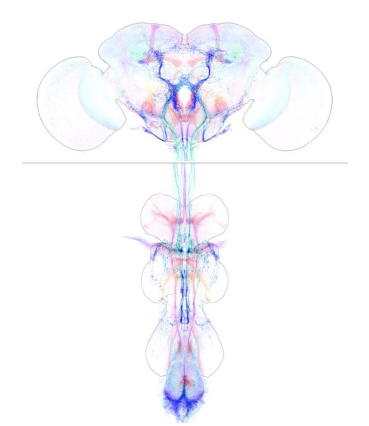

SS05108

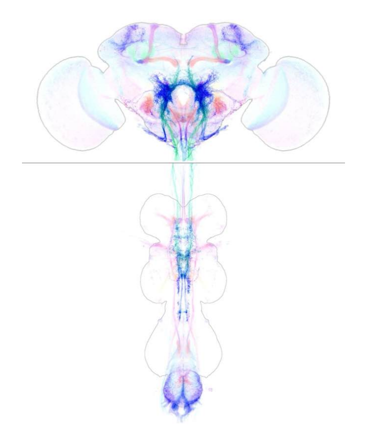

SS05116

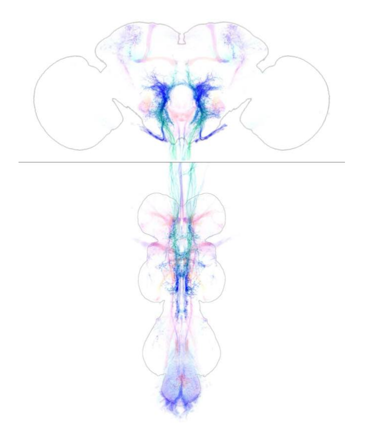

SS05122

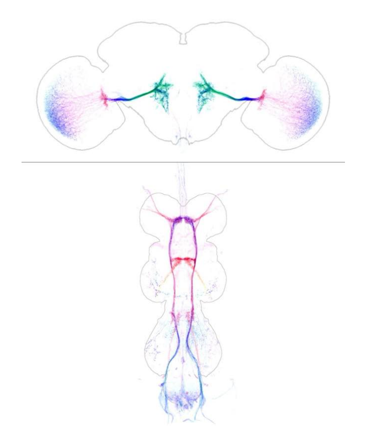

SS05141

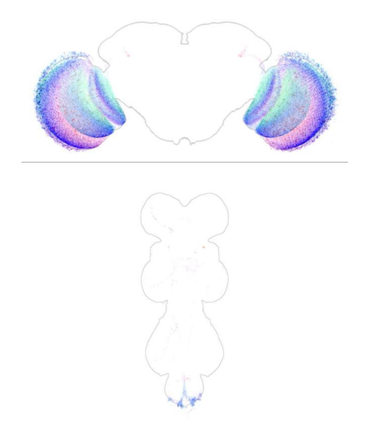

SS05153

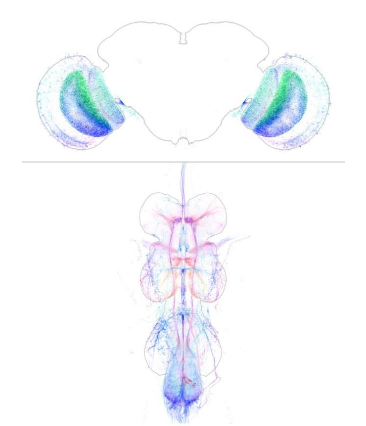

SS05188

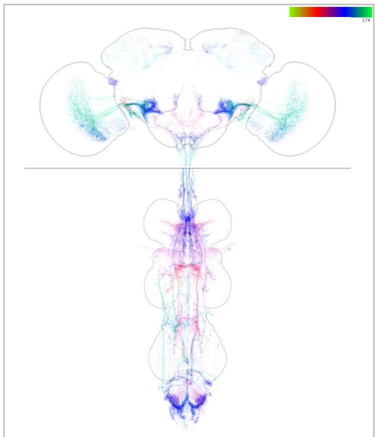
SS05255

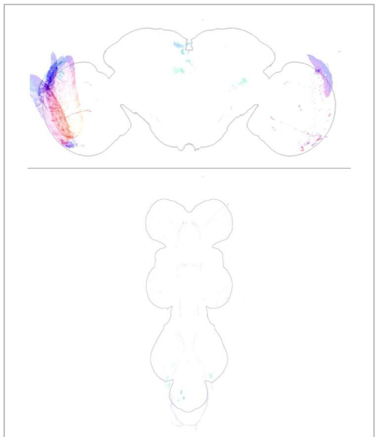
SS08909

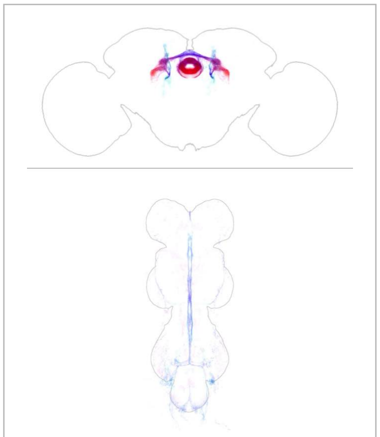
SS09135

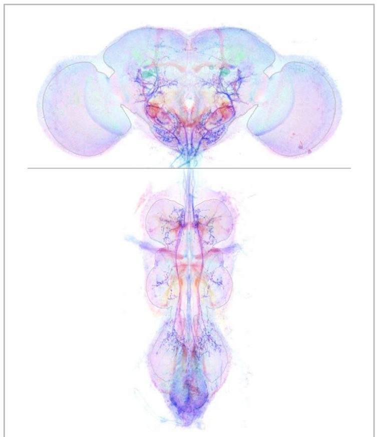
SS11141

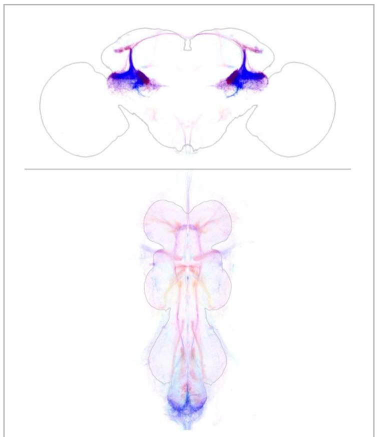
SS12089

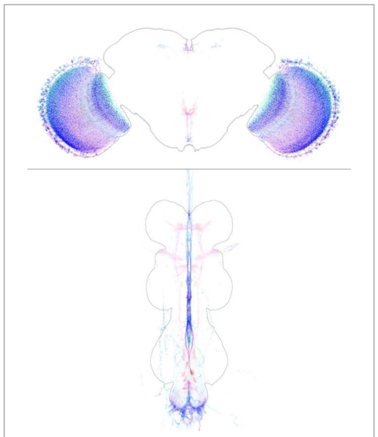
SS12598

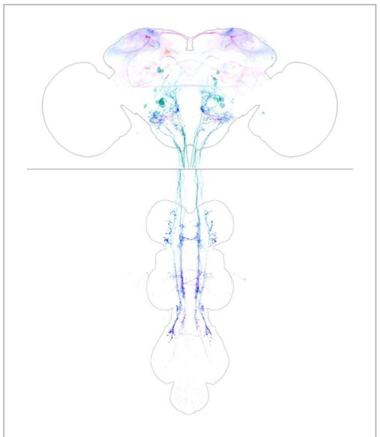
SS12653

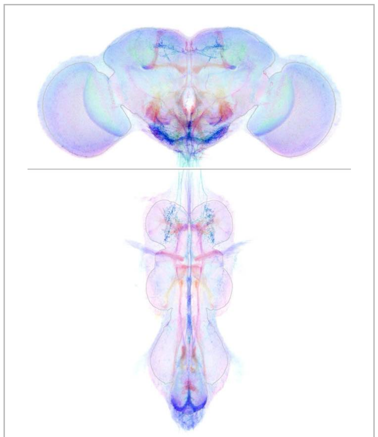
SS13337

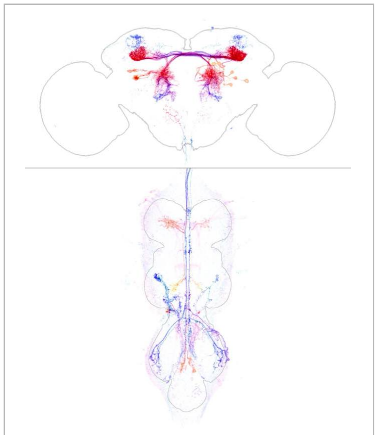
SS13616

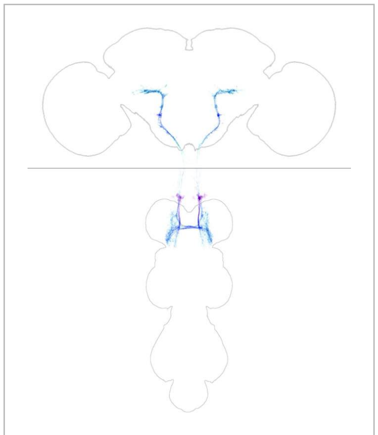
SS14728

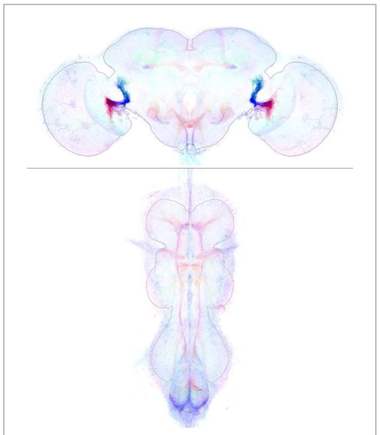
SS14770

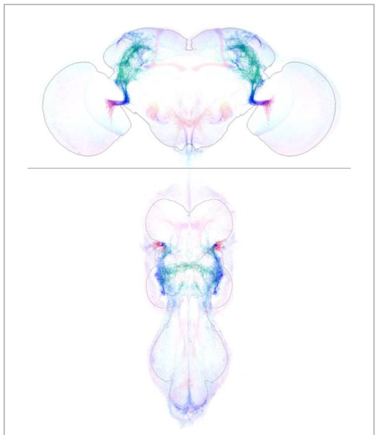
SS14827

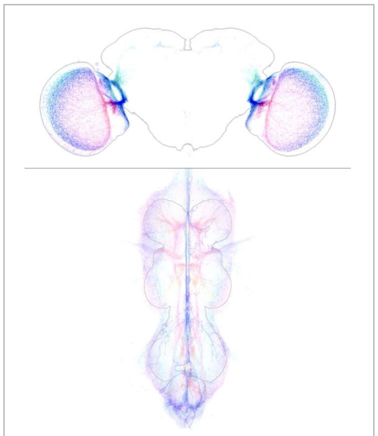
SS15156

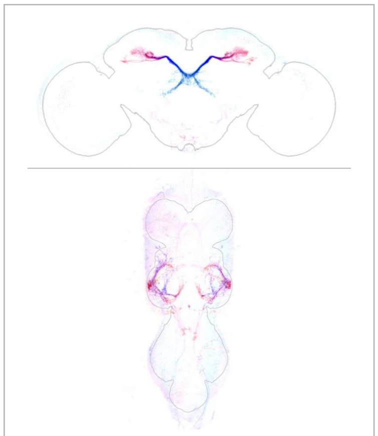
SS15431

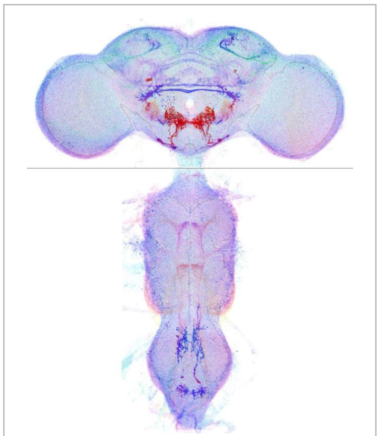
SS15725

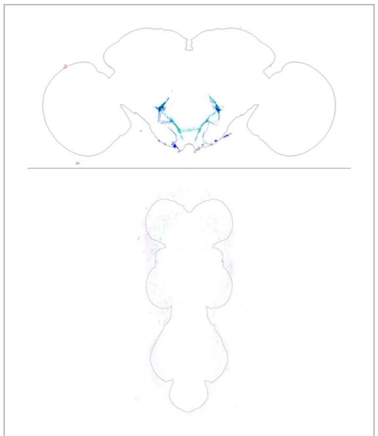
SS15829

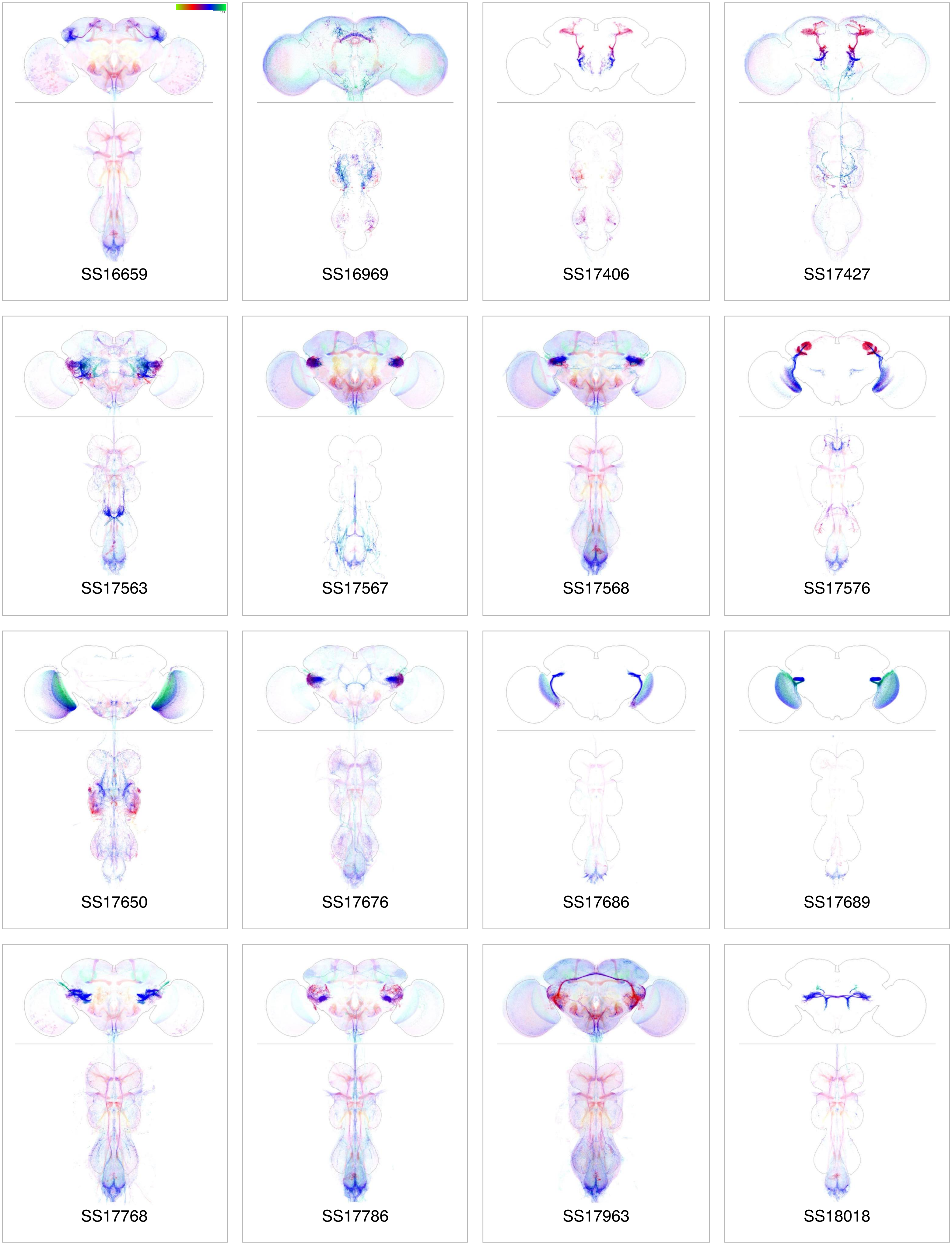
SS16659
SS16969
SS17406
SS17427
SS17563
SS17567
SS17568
SS17576
SS17650
SS17676
SS17686
SS17689
SS17768
SS17786
SS17963
SS18018

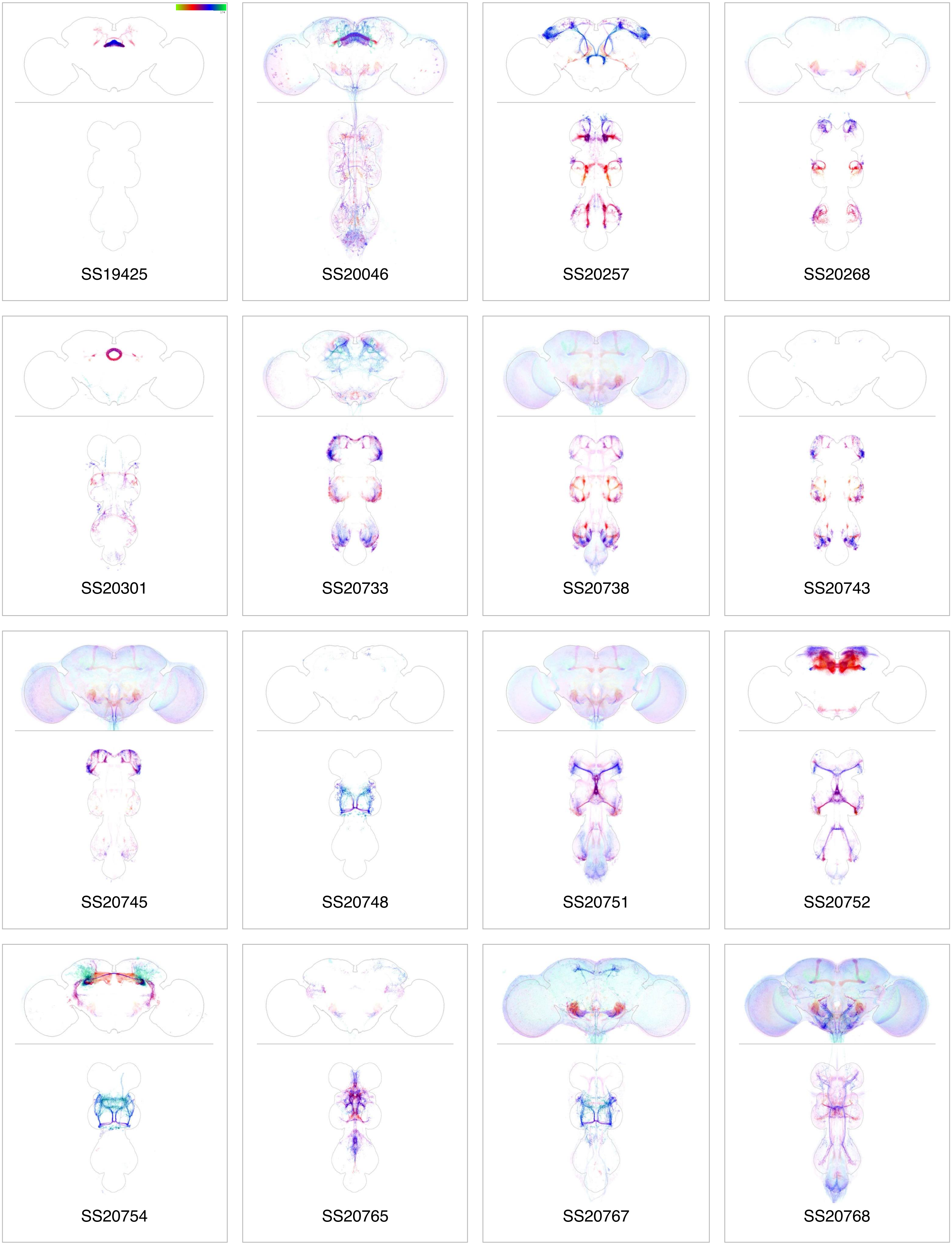
SS19425
SS20046
SS20257
SS20268
SS20301
SS20733
SS20738
SS20743
SS20745
SS20748
SS20751
SS20752
SS20754
SS20765
SS20767
SS20768

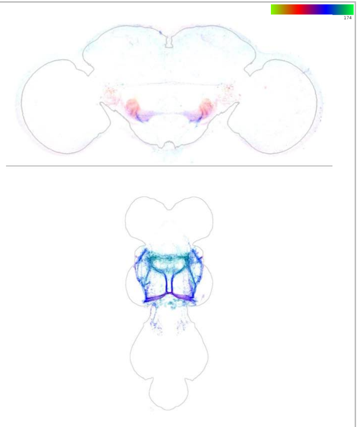
SS20769

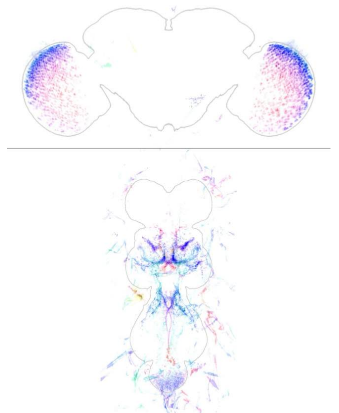
SS20770

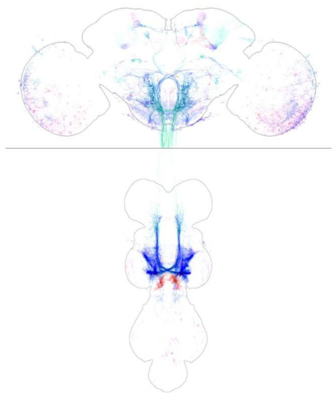
SS20774

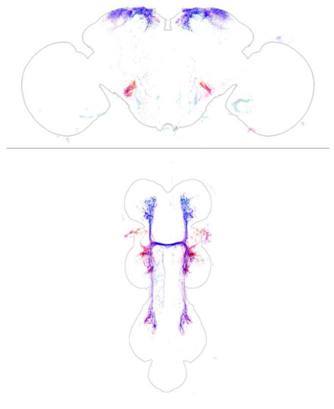
SS20790

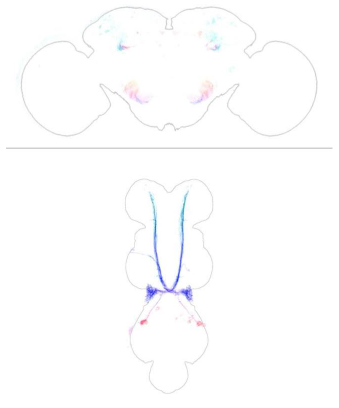
SS20791

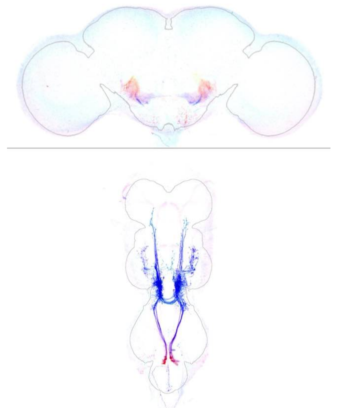
SS20797

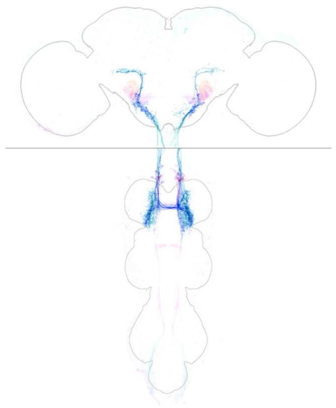
SS20806

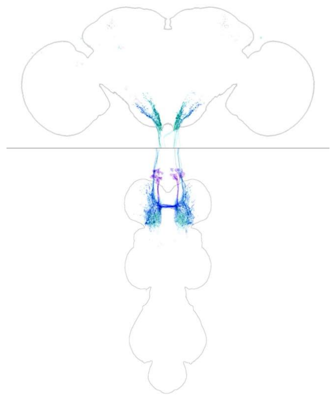
SS20807

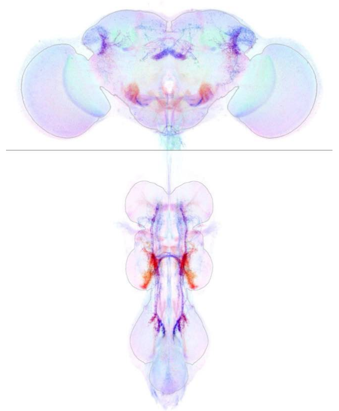
SS20810

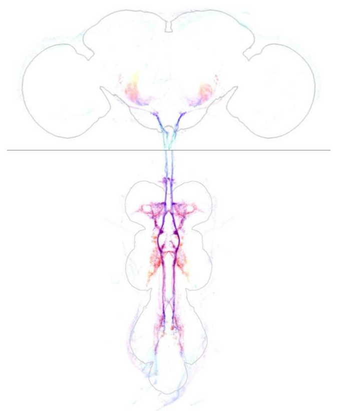
SS20818

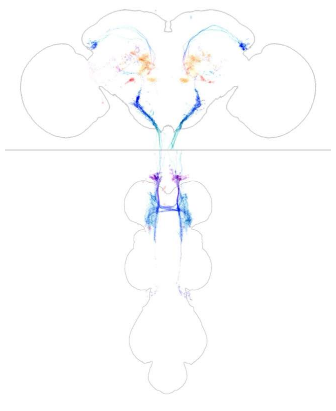
SS20826

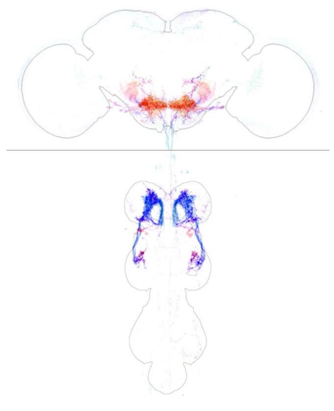
SS20829

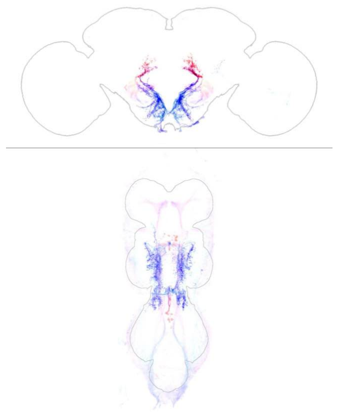
SS20830

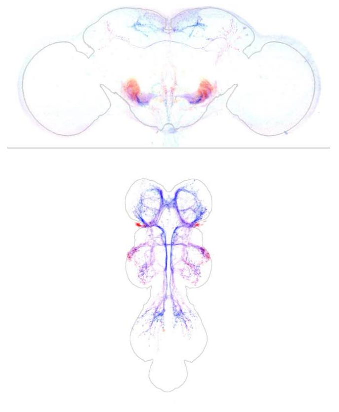
SS20834

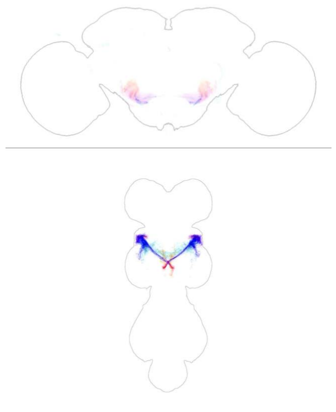
SS20846

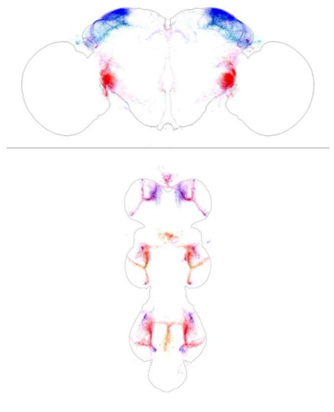
SS20847

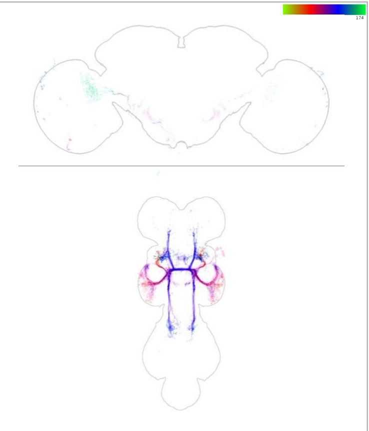
SS20849

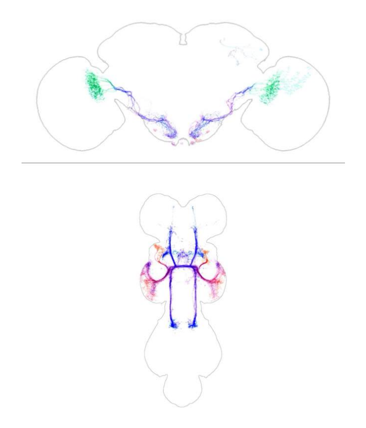
SS20856

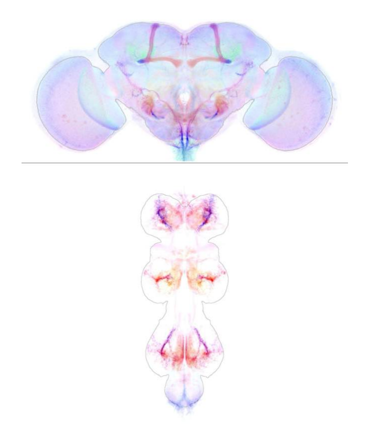
SS20857

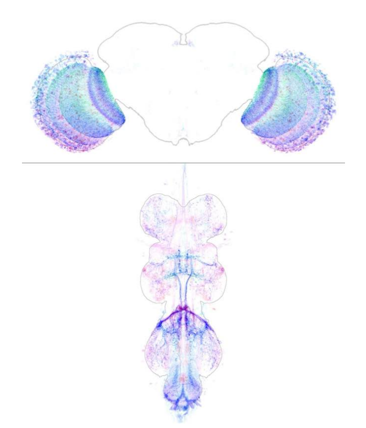
SS20987

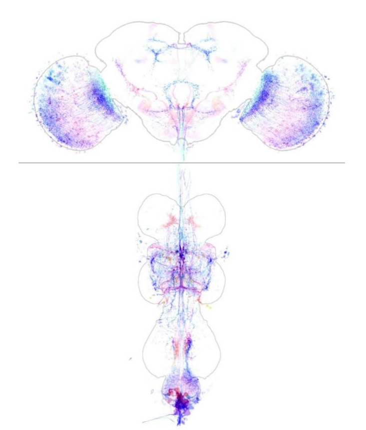
SS20992

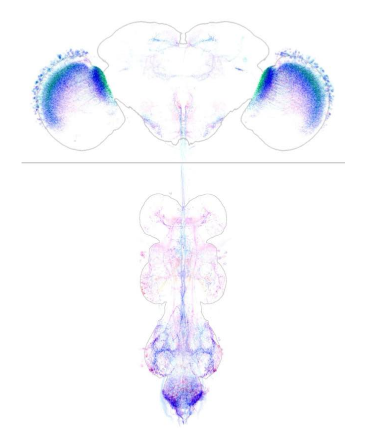
SS21011

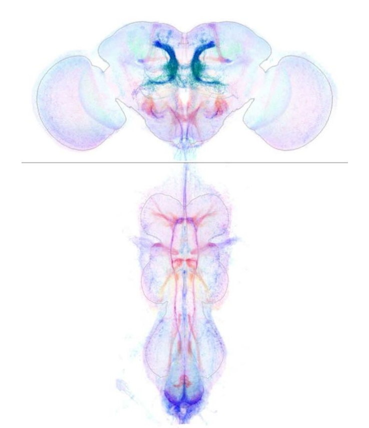
SS21089

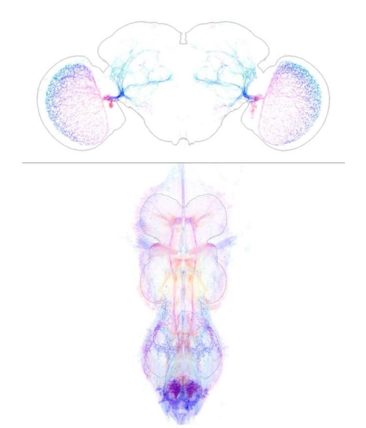
SS21109

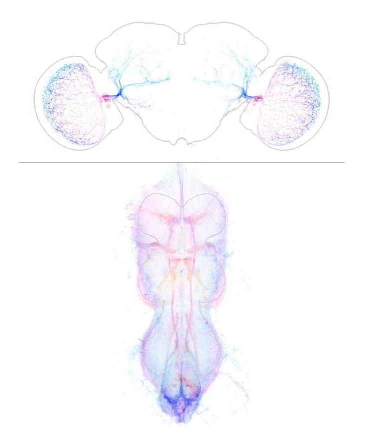
SS21160

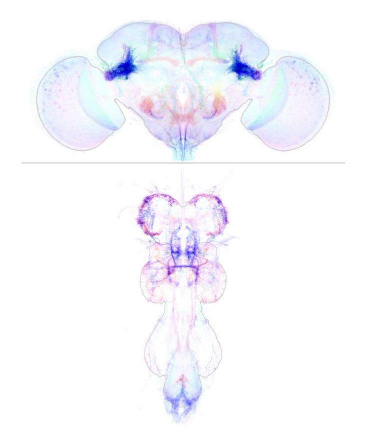
SS21292

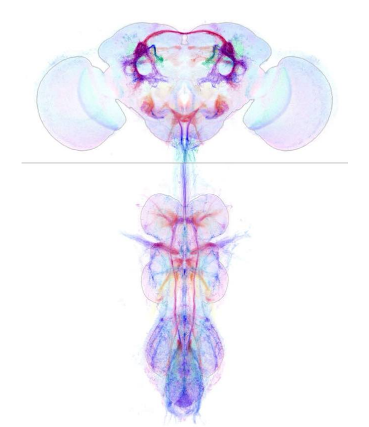
SS21346

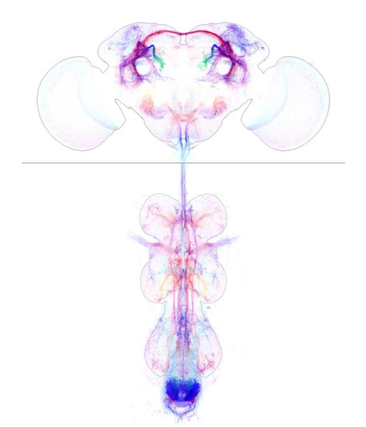
SS21348

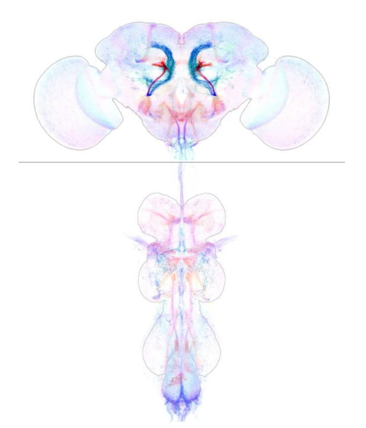
SS21359

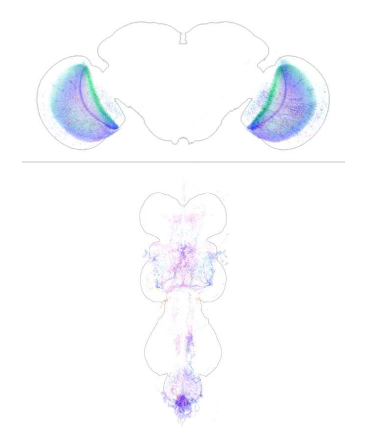
SS21452

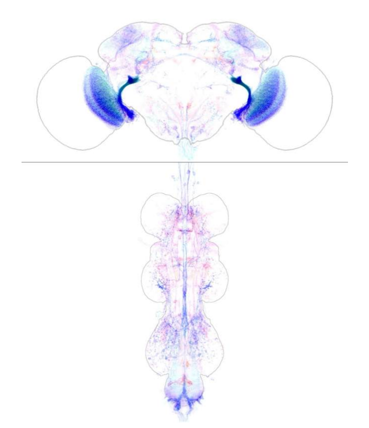
SS21500

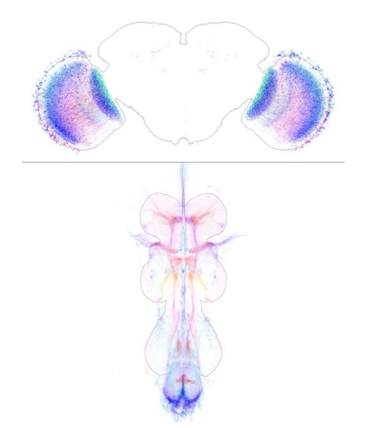
SS21536

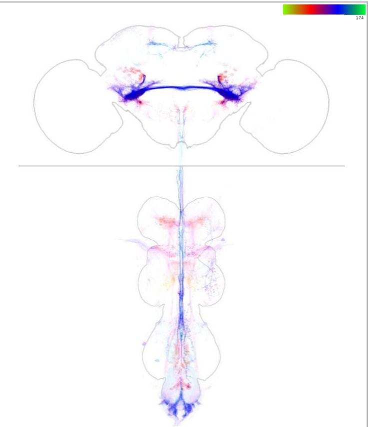

SS21644

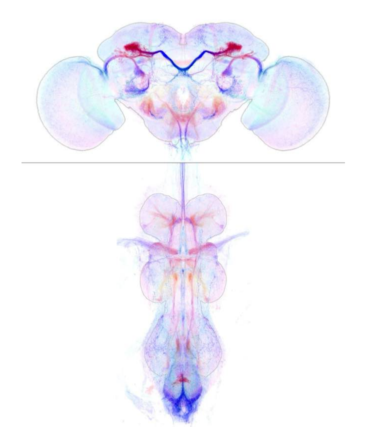

SS21652

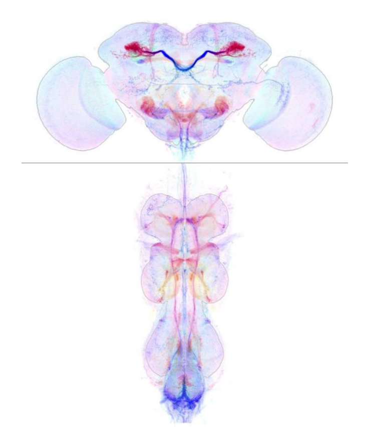

SS21658

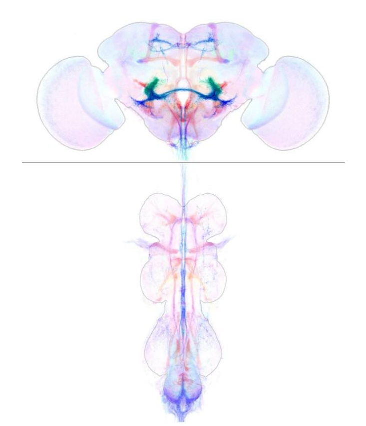

SS21679

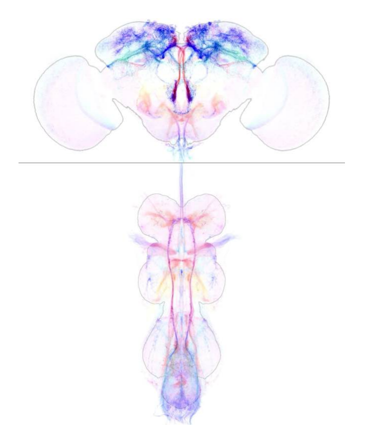

SS21696

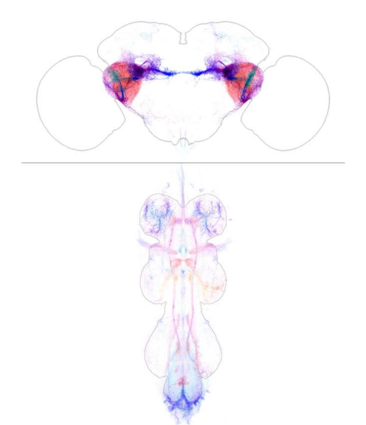

SS21914

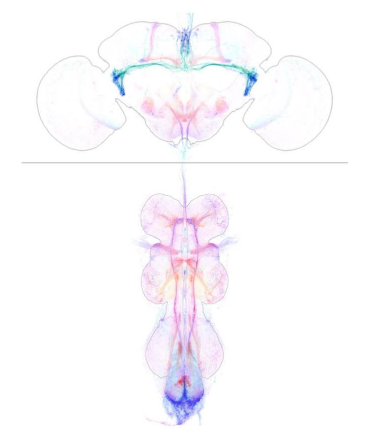

SS21963

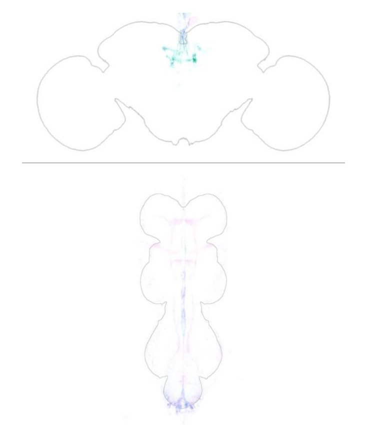

SS21976

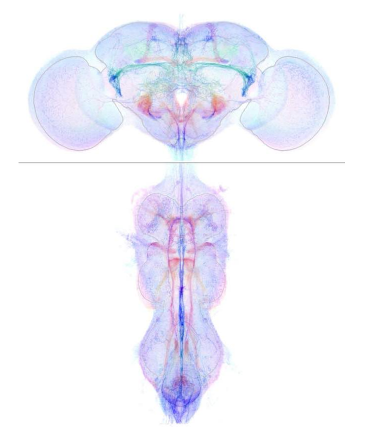

SS22035

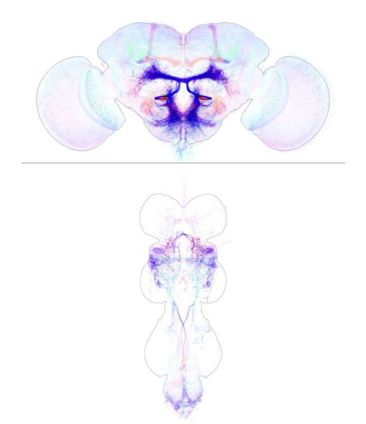

SS22118

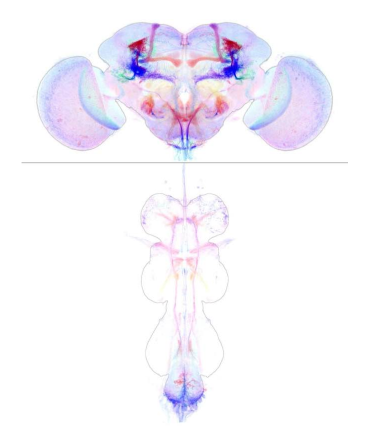

SS22242

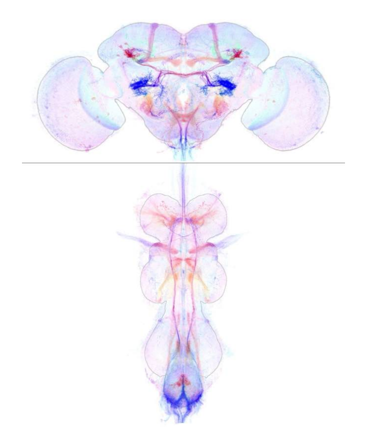

SS22285

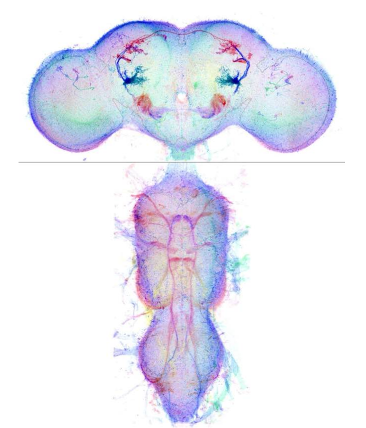

SS22293

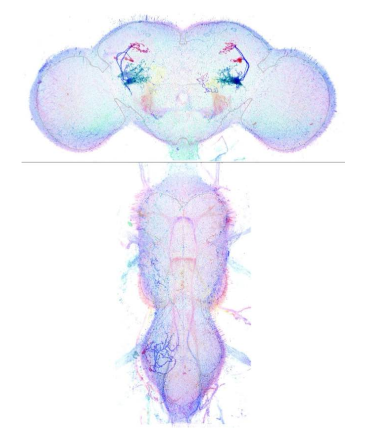

SS22296

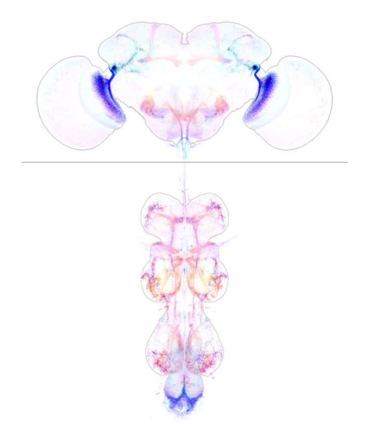

SS22311

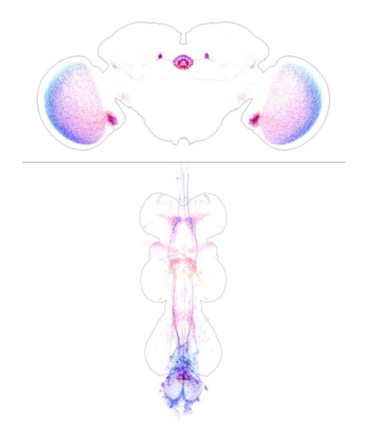

SS22313

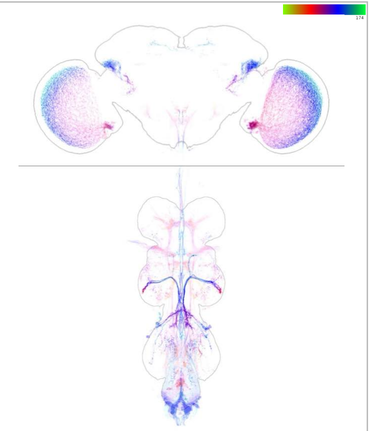
SS22315

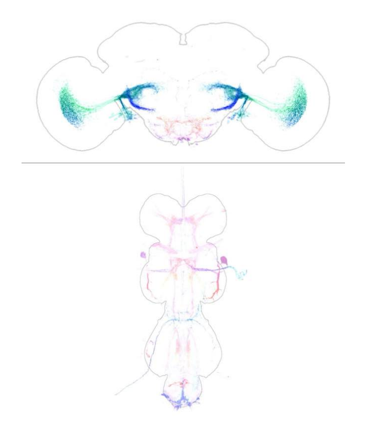
SS22403

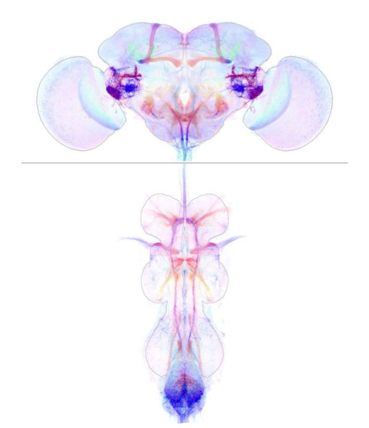
SS22437

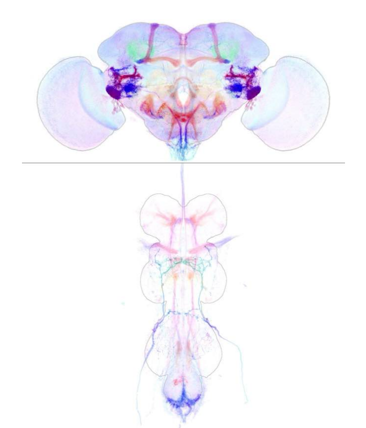
SS22441

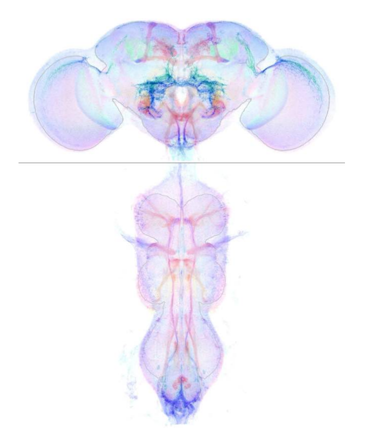
SS22505

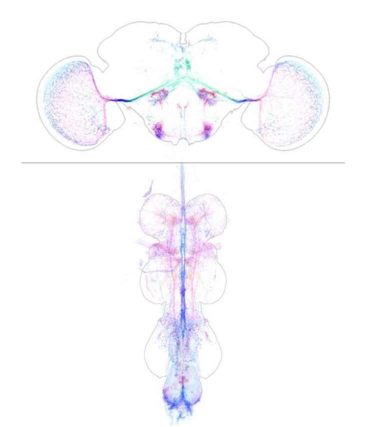
SS22506

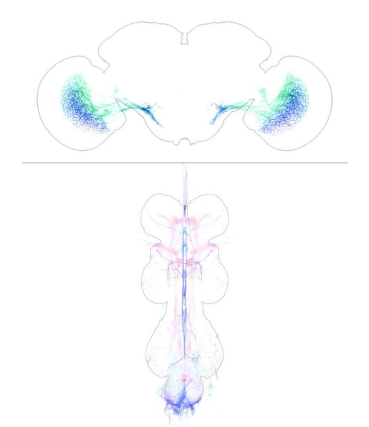
SS22522

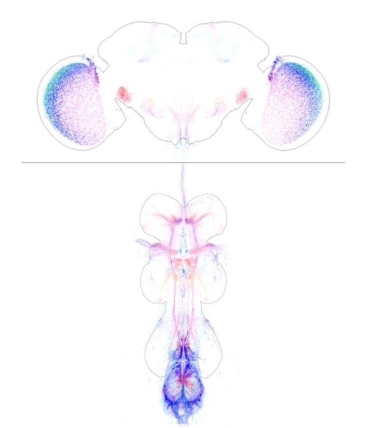
SS22528

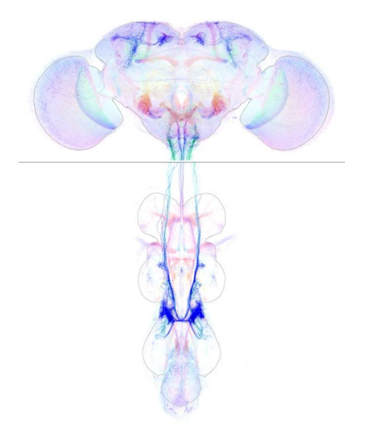
SS23197

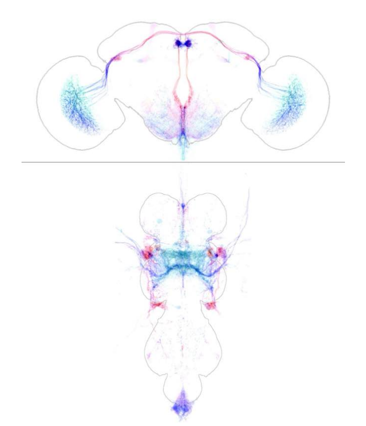
SS23224

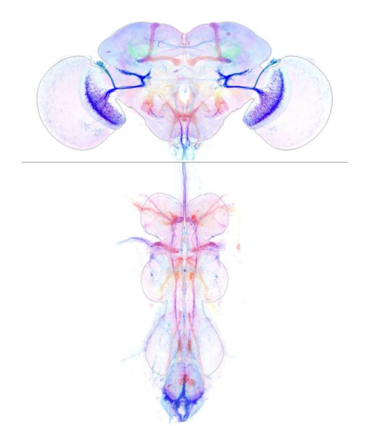
SS23236

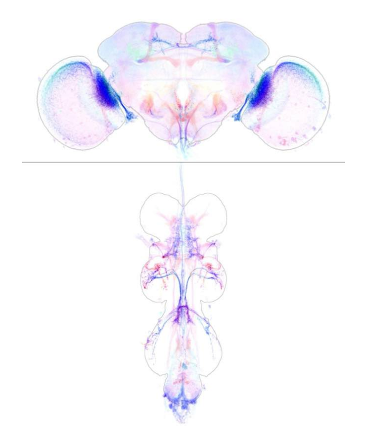
SS23264

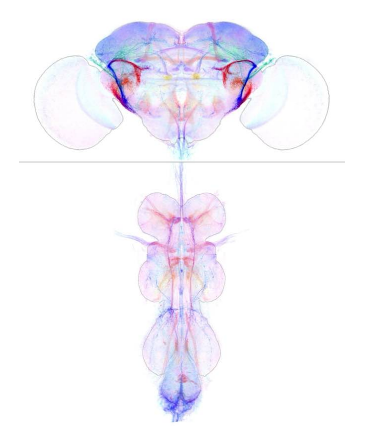
SS23281

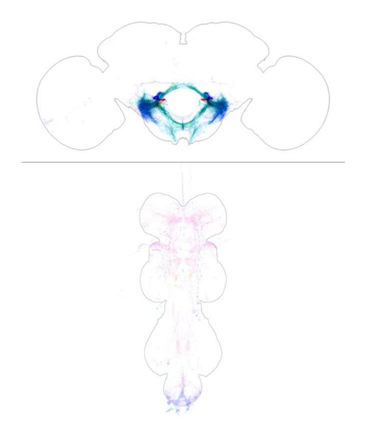
SS23540

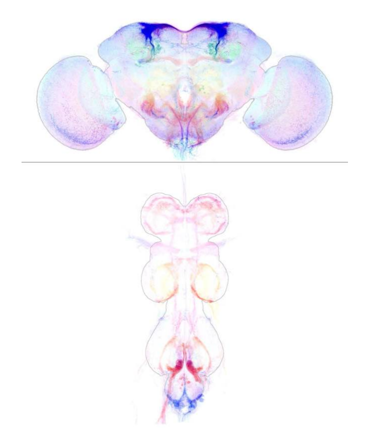
SS23556

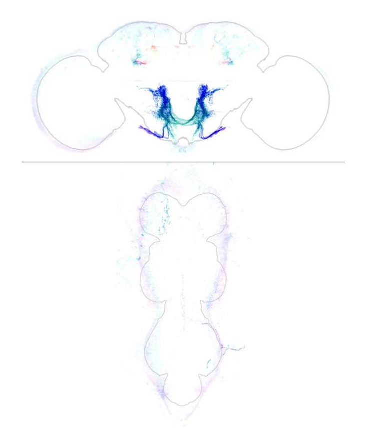
SS23599

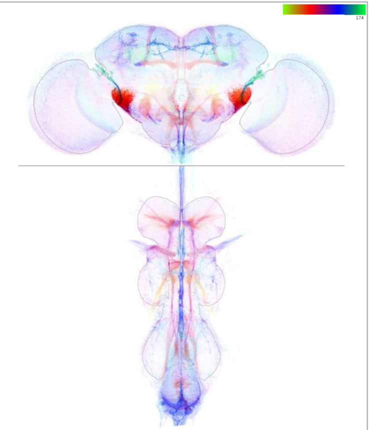

SS23627

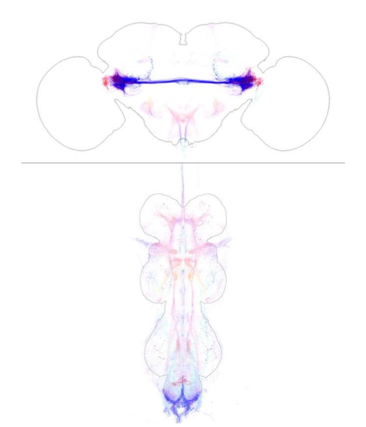

SS23691

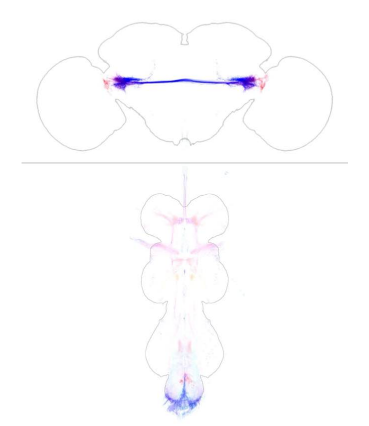

SS23720

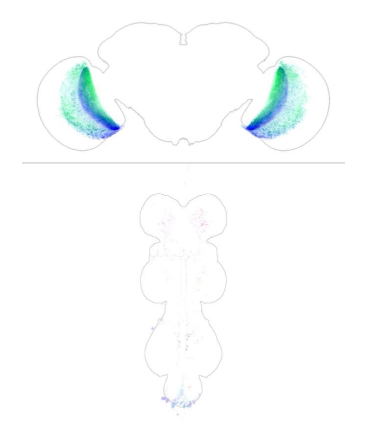

SS23757

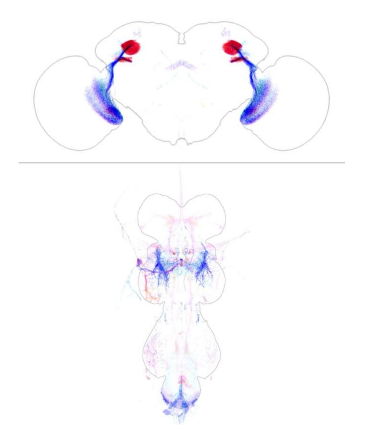

SS23773

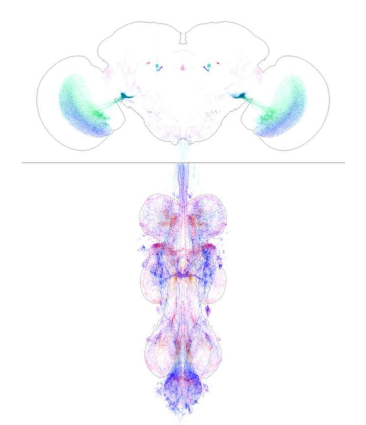

SS23786

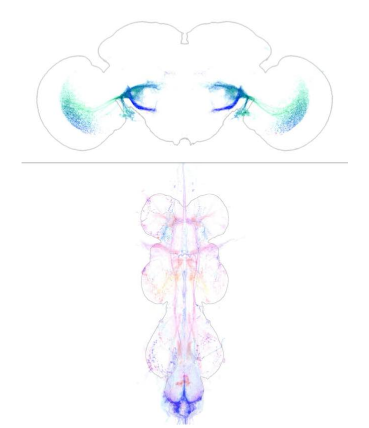

SS23850

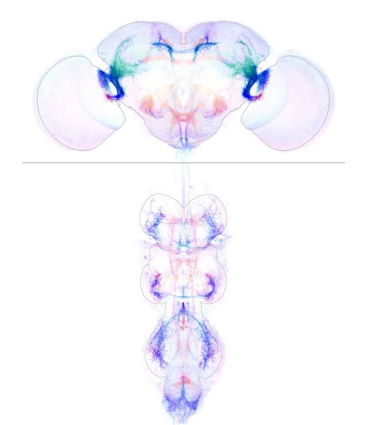

SS23858

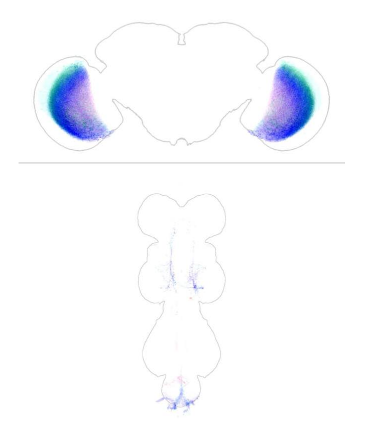

SS23866

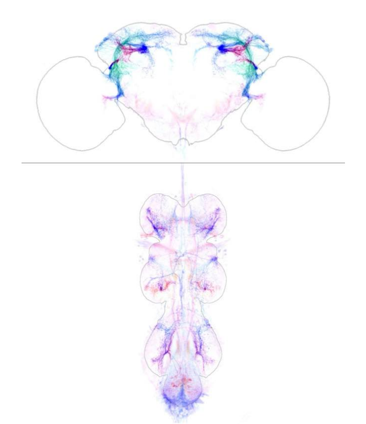

SS23879

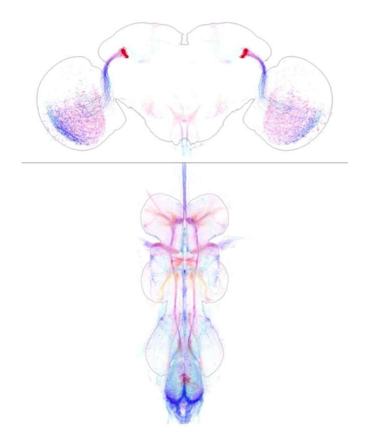

SS23880

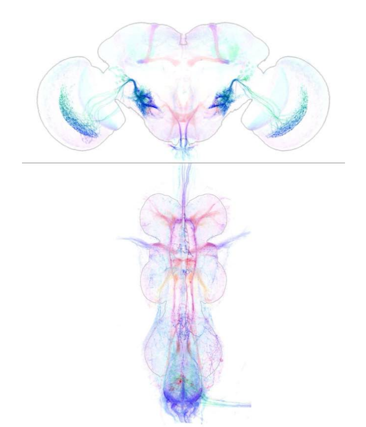

SS23881

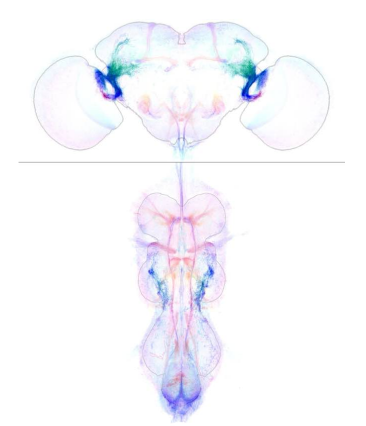

SS23921

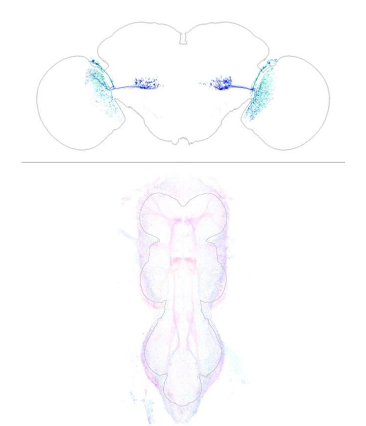

SS23981

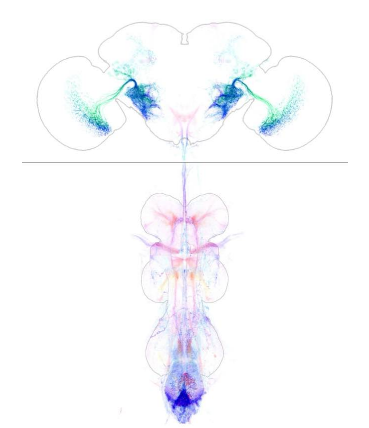

SS24001

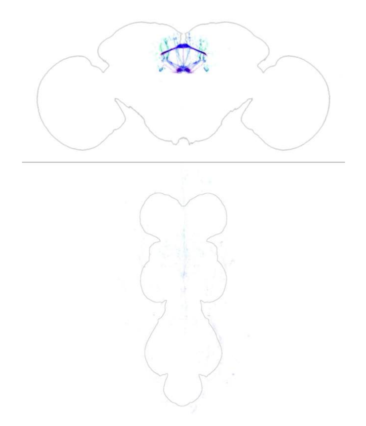

SS24258

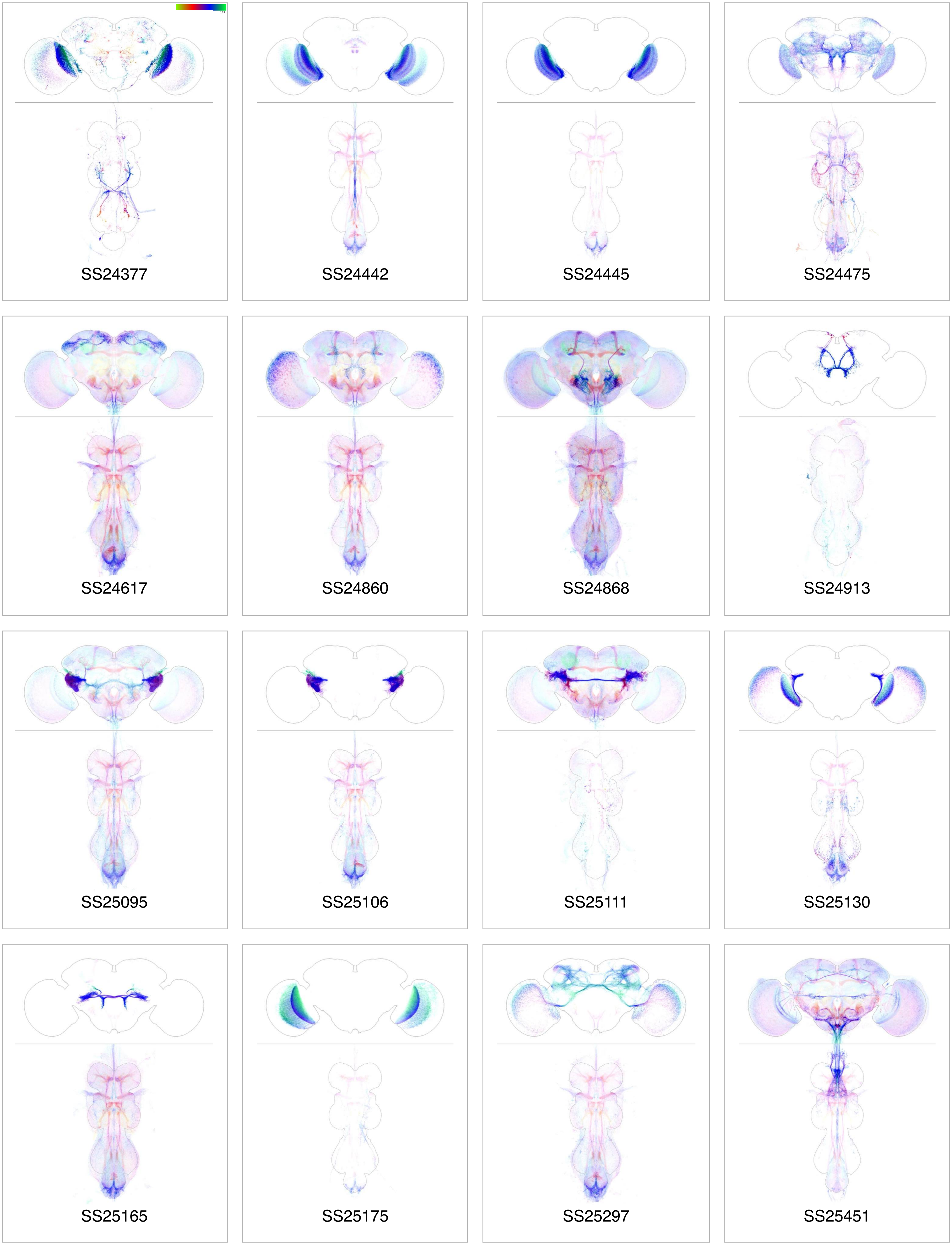
SS24377
SS24442
SS24445
SS24475
SS24617
SS24860
SS24868
SS24913
SS25095
SS25106
SS25111
SS25130
SS25165
SS25175
SS25297
SS25451

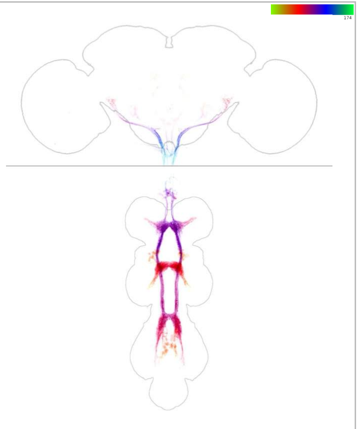
SS25466

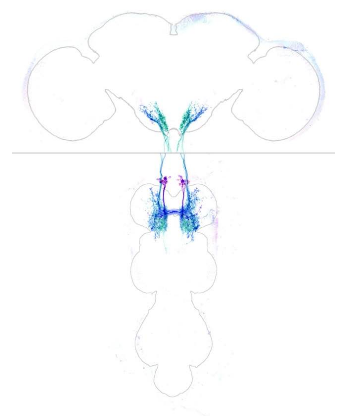
SS25469

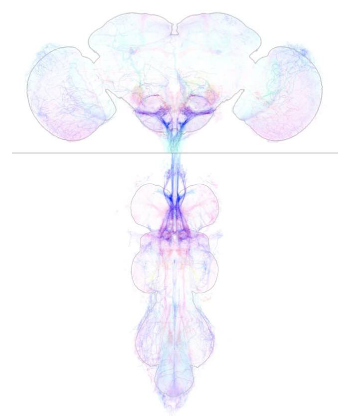
SS25470

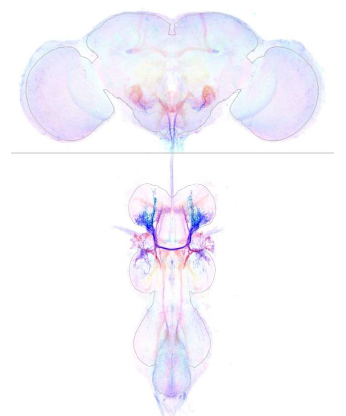
SS25473

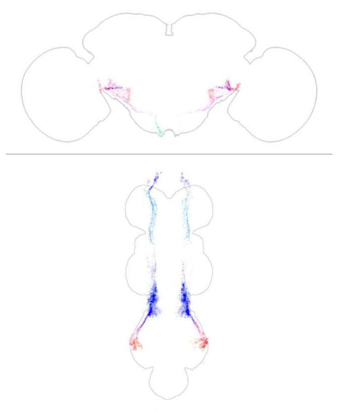
SS25478

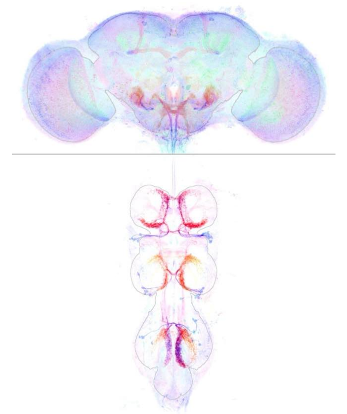
SS25480

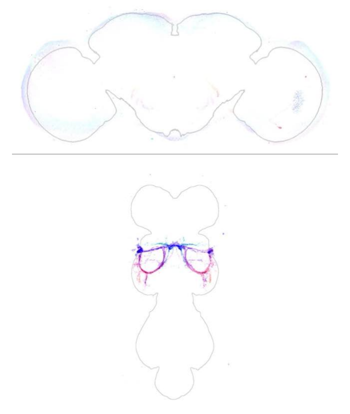
SS25482

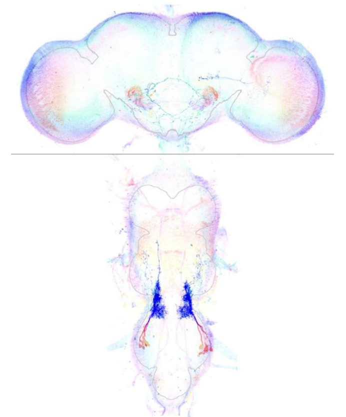
SS25500

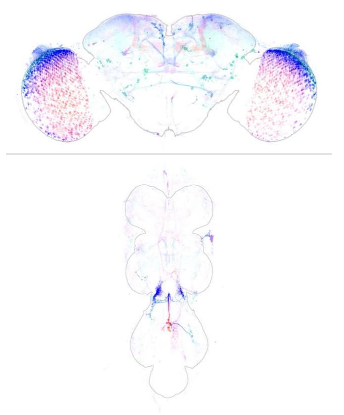
SS25502

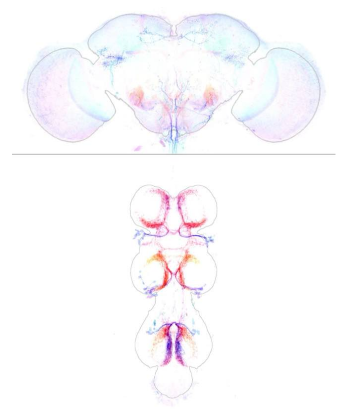
SS25504

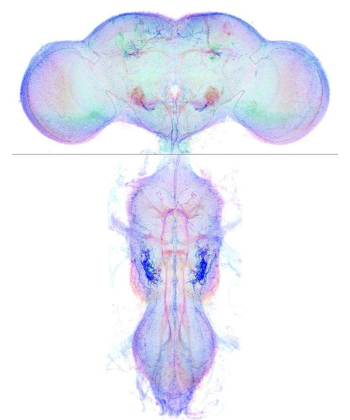
SS25511

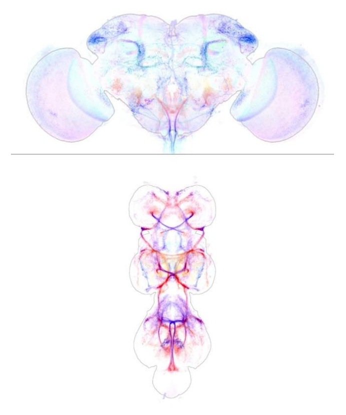
SS25516

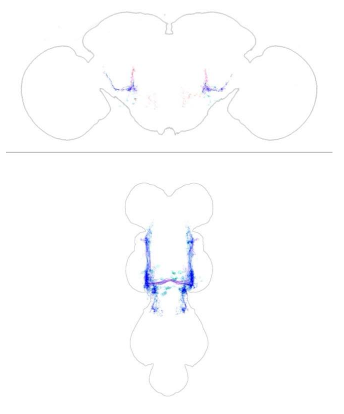
SS25521

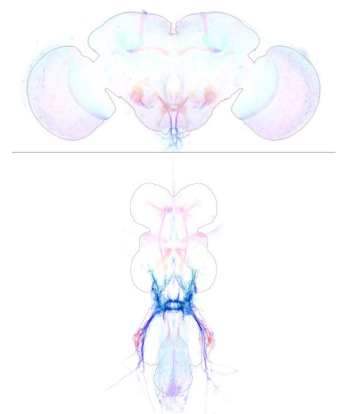
SS25532

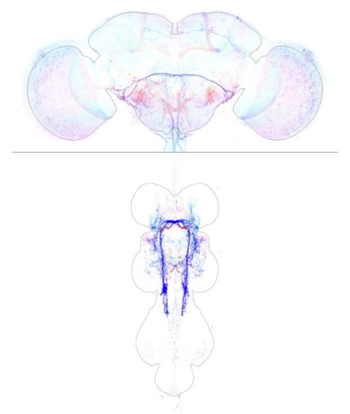
SS25553

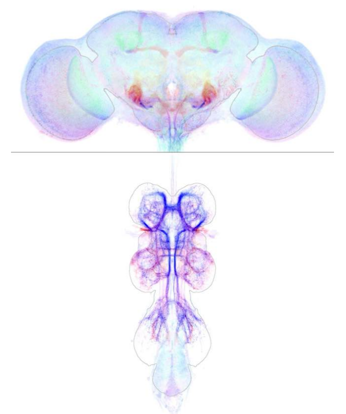
SS25564

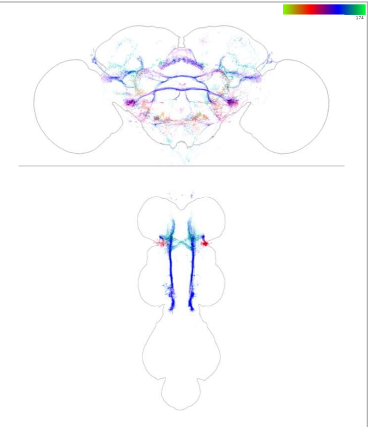
SS25572

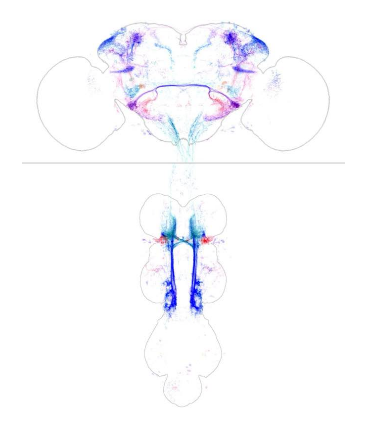
SS25573

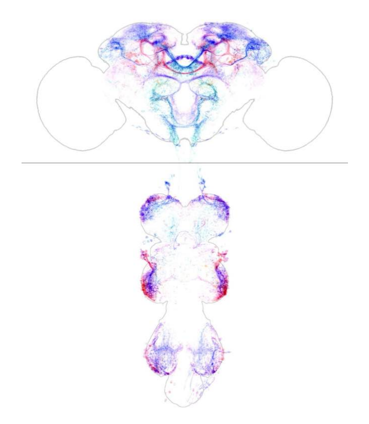
SS25575

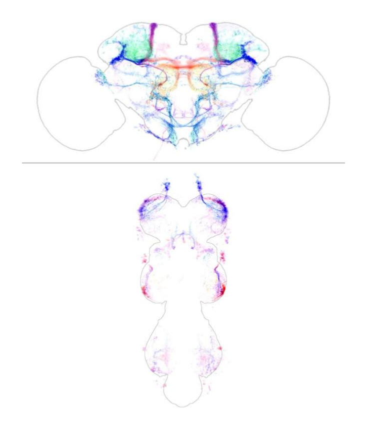
SS25576

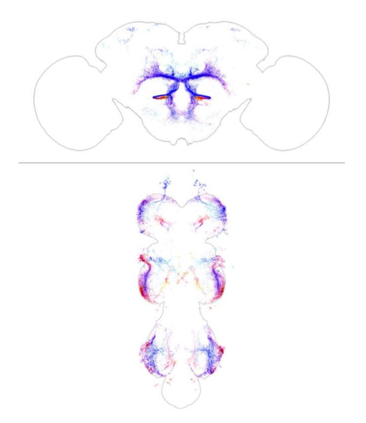
SS25577

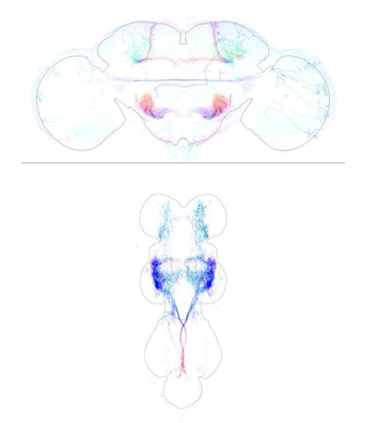
SS25580

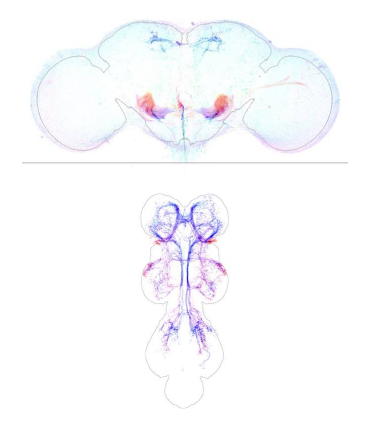
SS25582

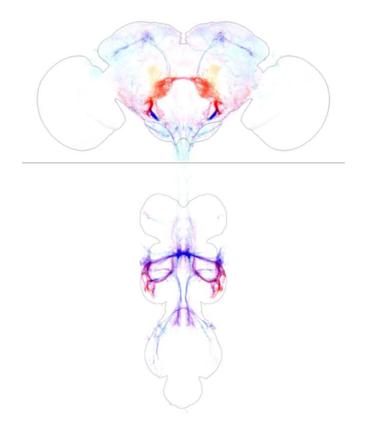
SS25585

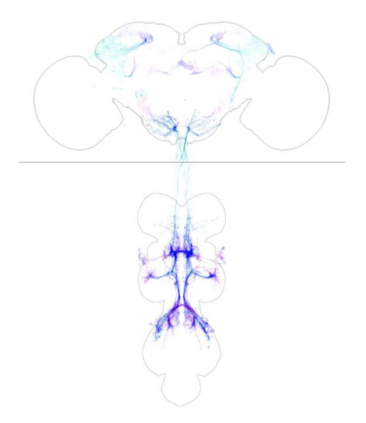
SS25586

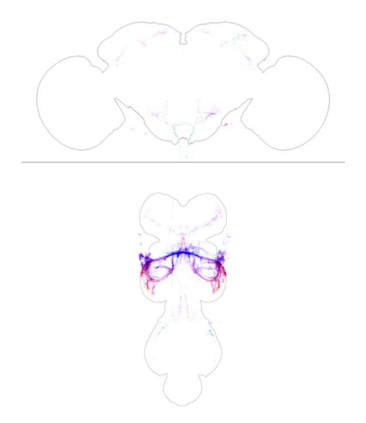
SS25587

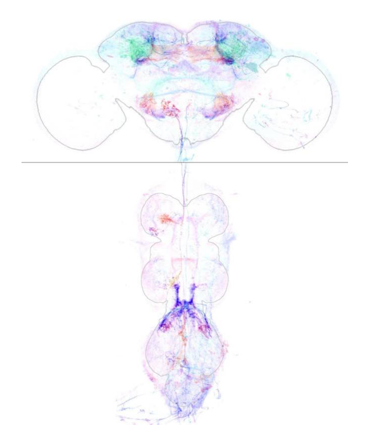
SS25594

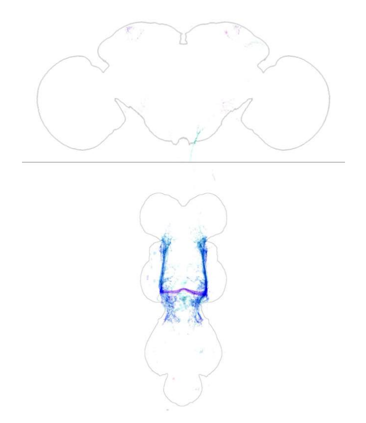
SS25597

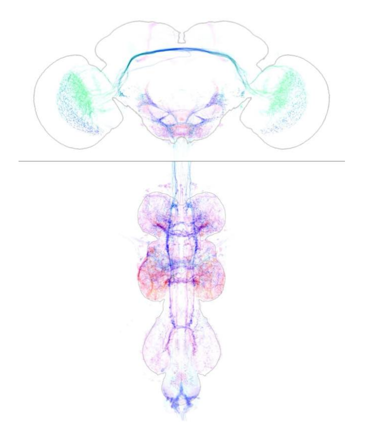
SS25632

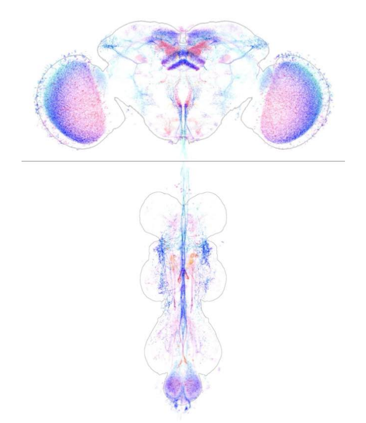
SS25646

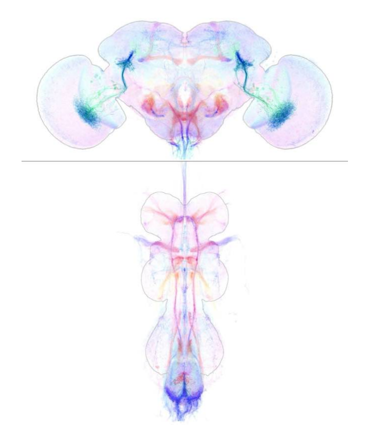
SS25663

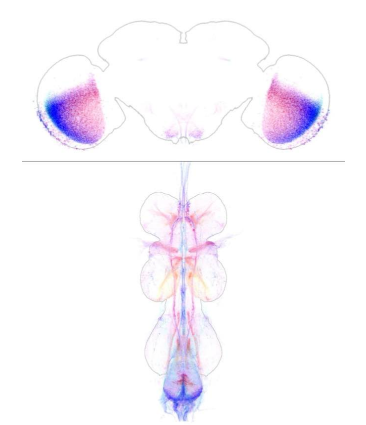
SS25675

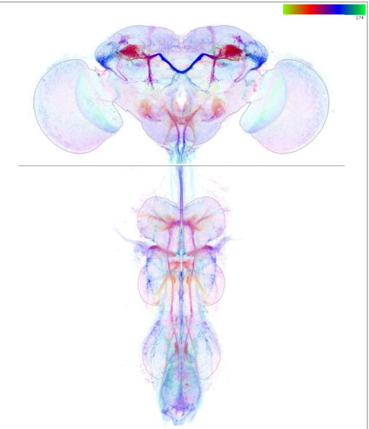
SS25733

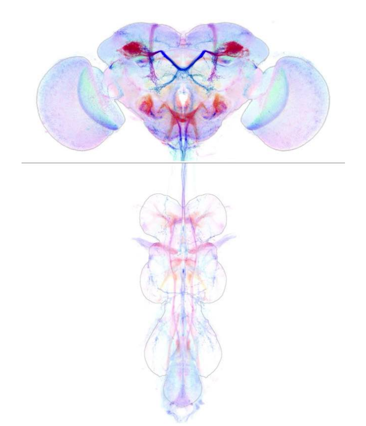
SS25735

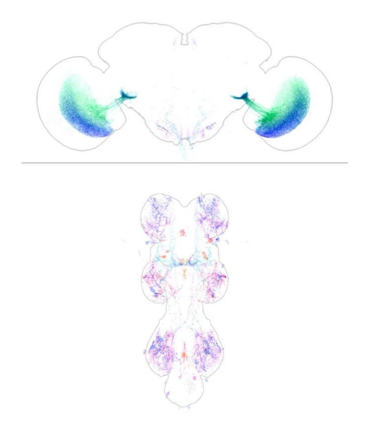
SS25868

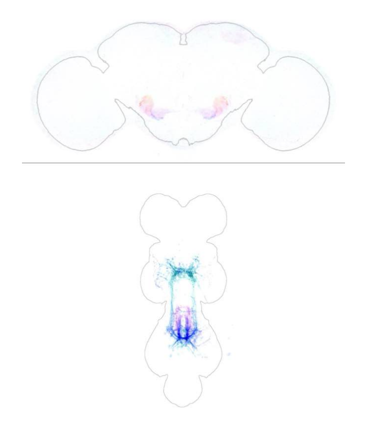
SS25885

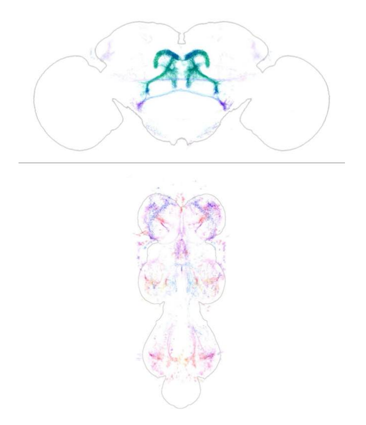
SS25983

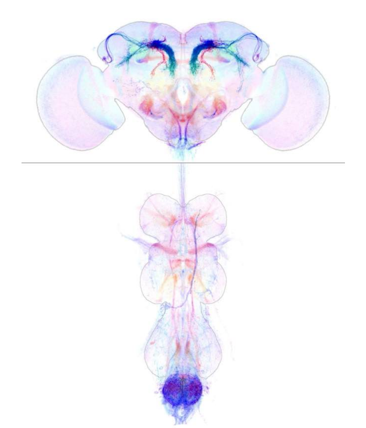
SS26094

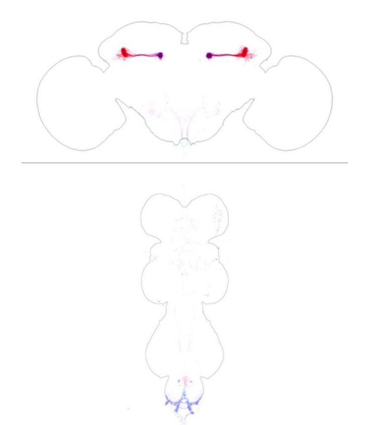
SS26213

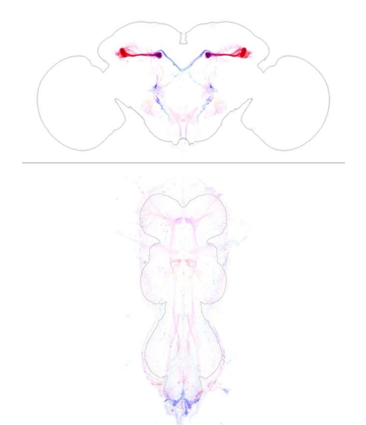
SS26256

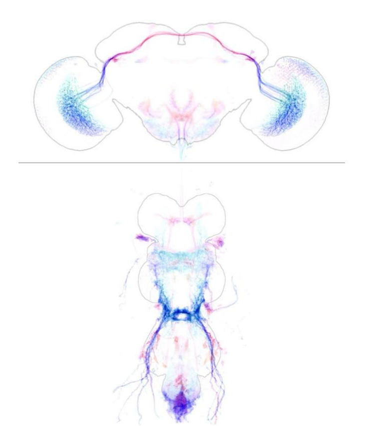
SS26275

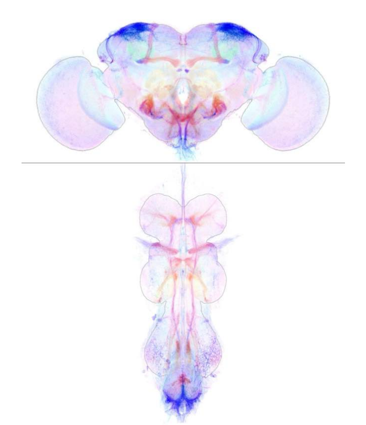
SS26356

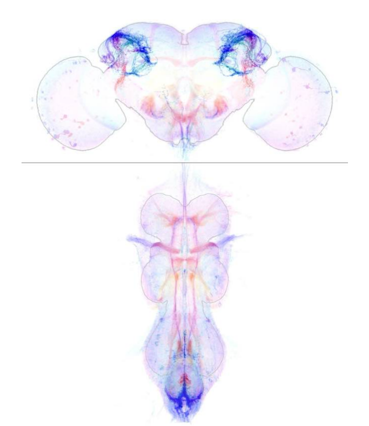
SS26358

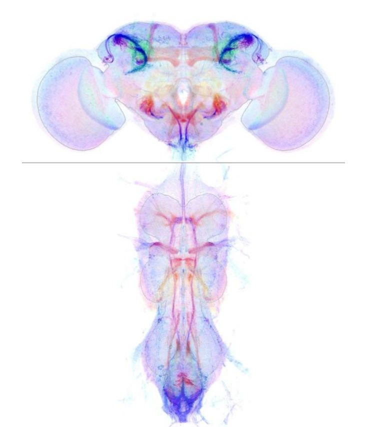
SS26371

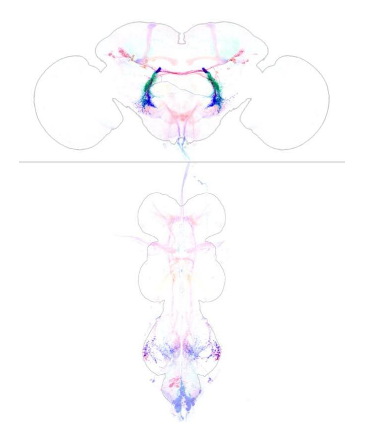
SS26512

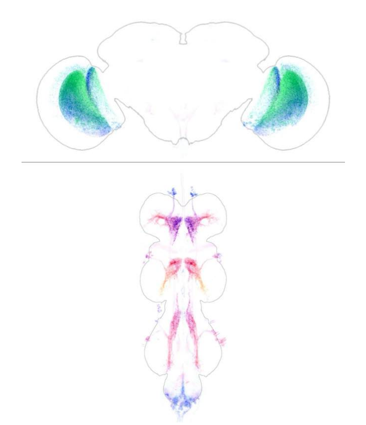
SS26540

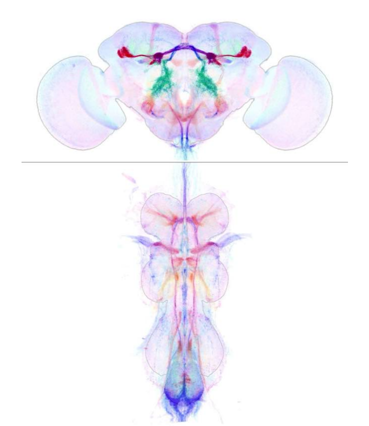
SS26563

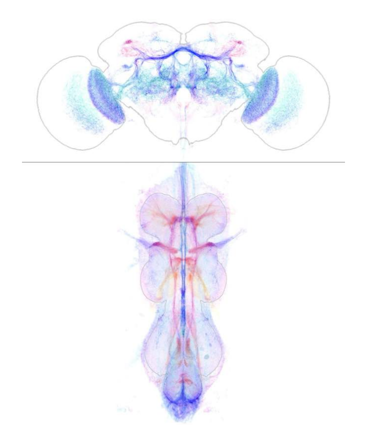
SS26753

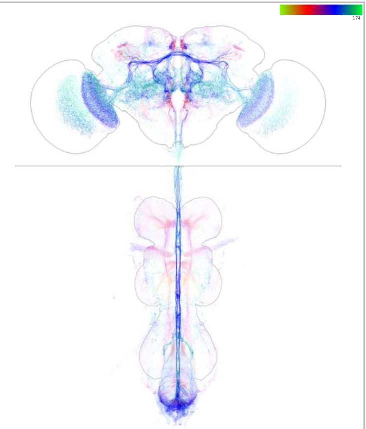

SS26754

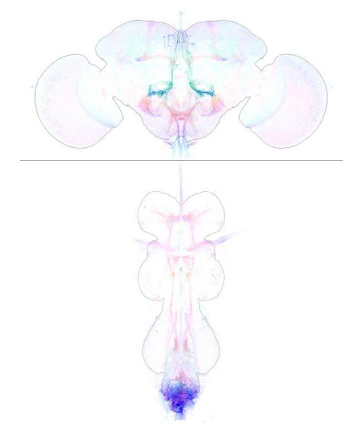

SS26913

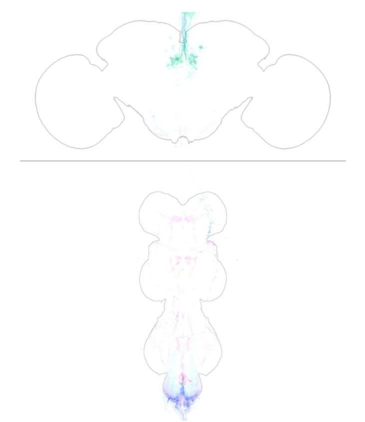

SS26926

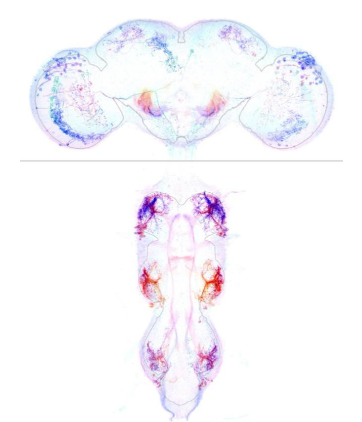

SS26953

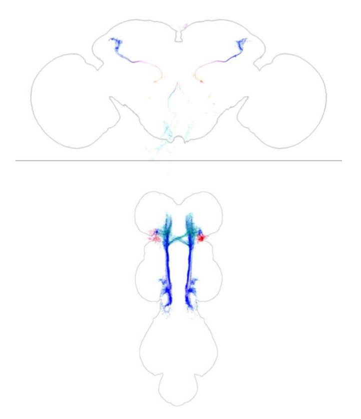

SS26967

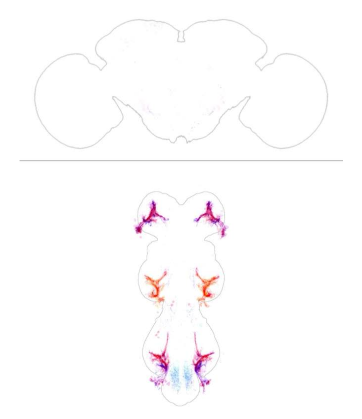

SS26968

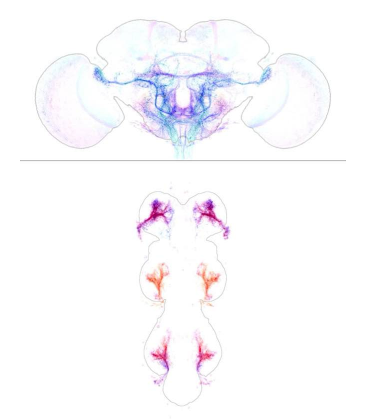

SS26973

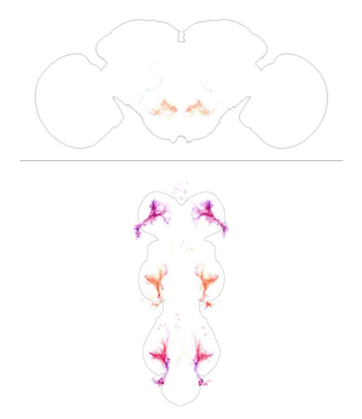

SS26974

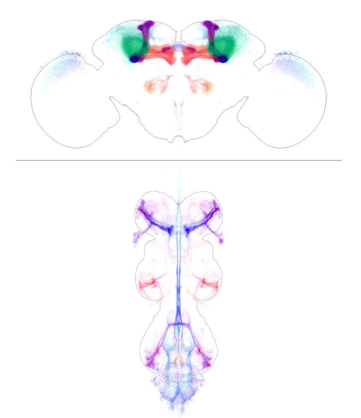

SS26977

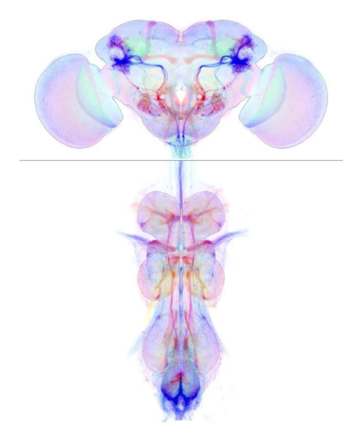

SS27135

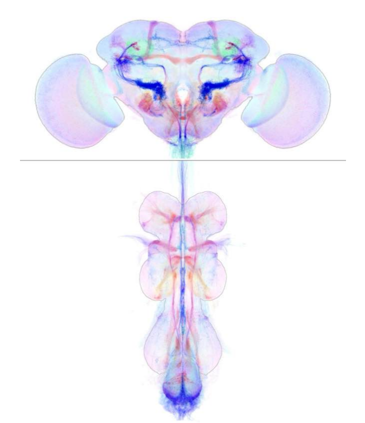

SS27137

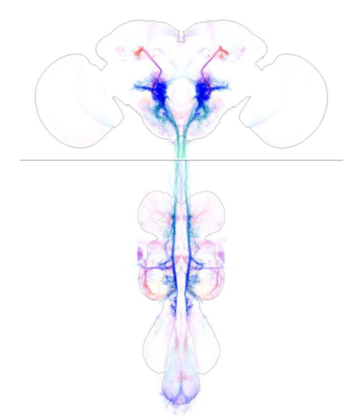

SS27191

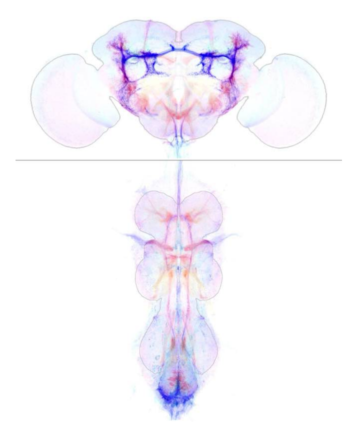

SS27239

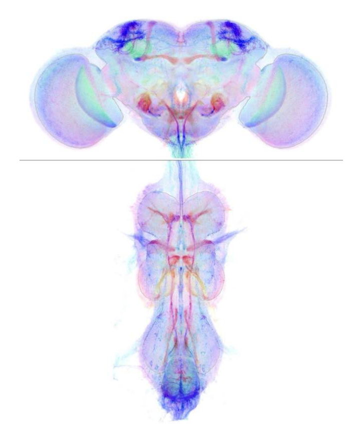

SS27255

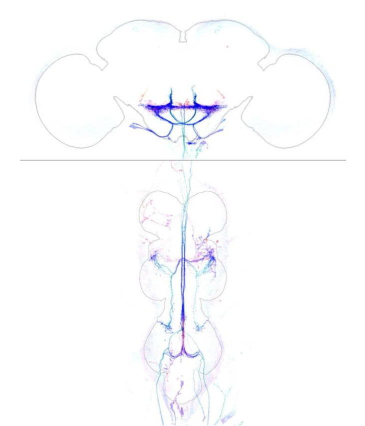

SS27265

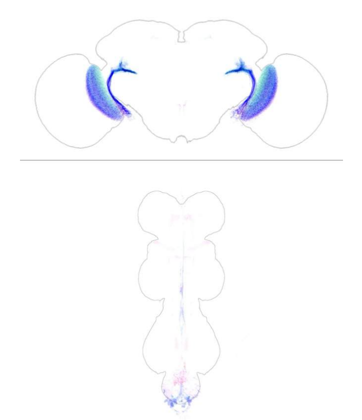

SS27383

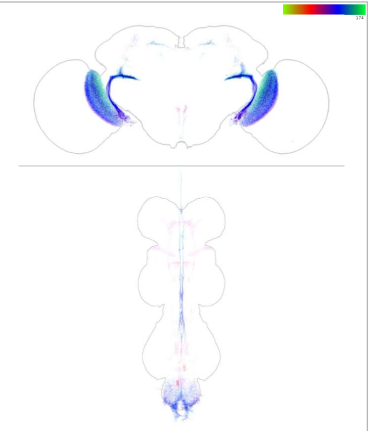
SS27386

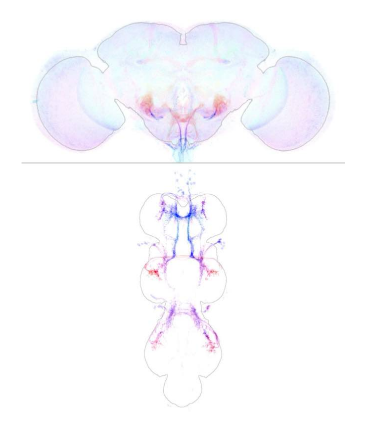
SS27416

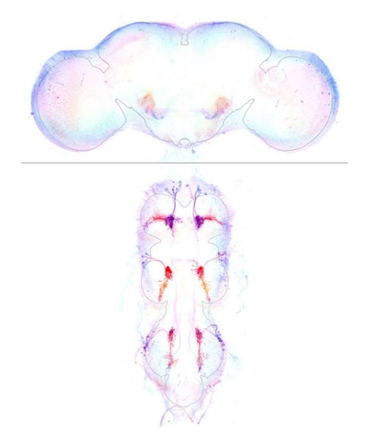
SS27422

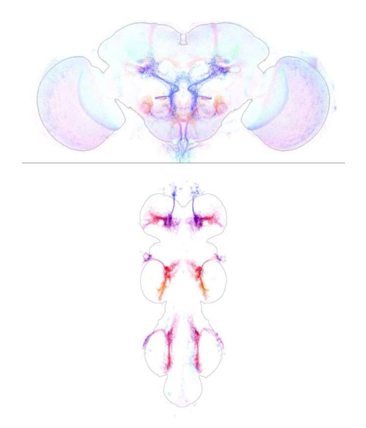
SS27425

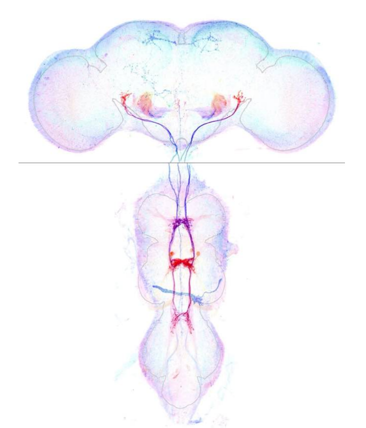
SS27485

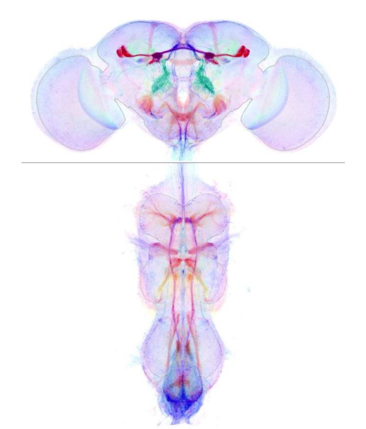
SS27547

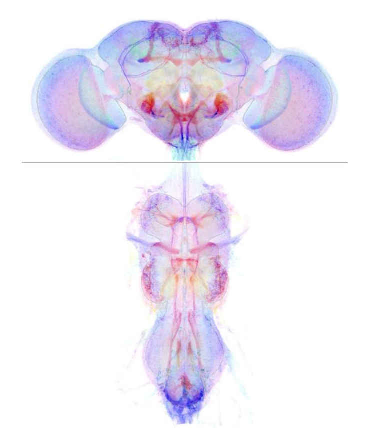
SS27580

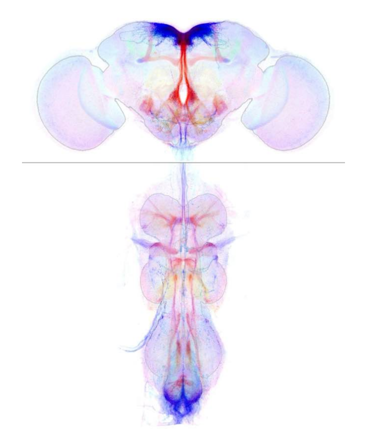
SS27590

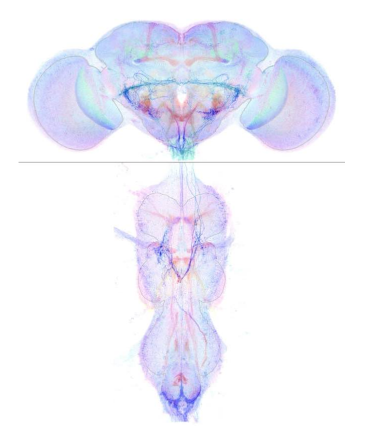
SS27649

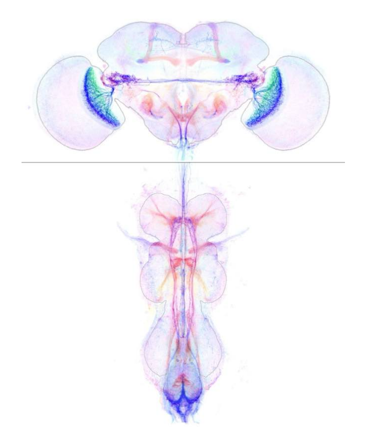
SS27725

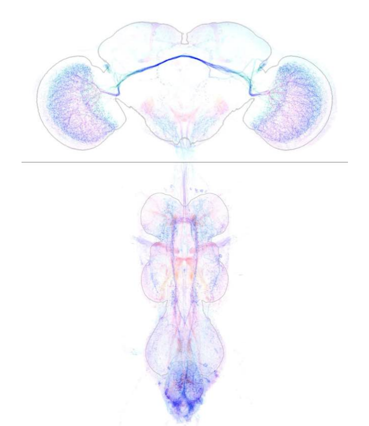
SS27796

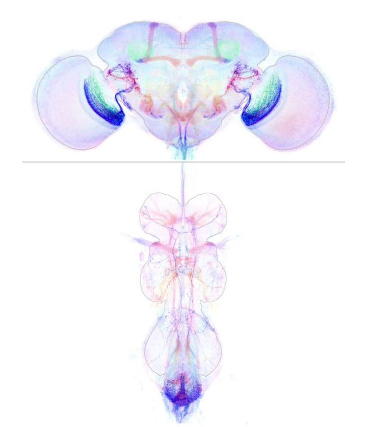
SS27802

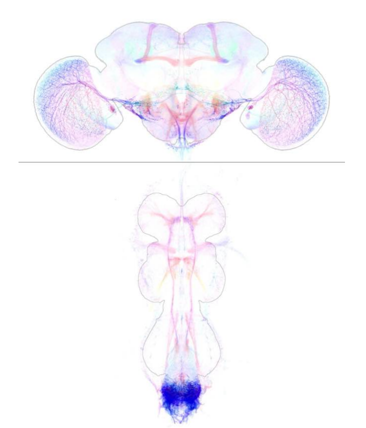
SS27808

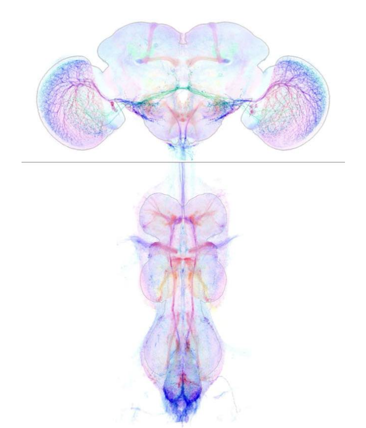
SS27814

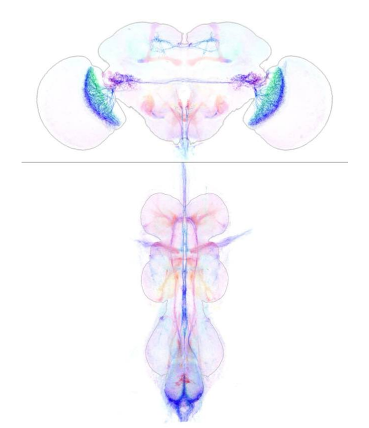
SS27817

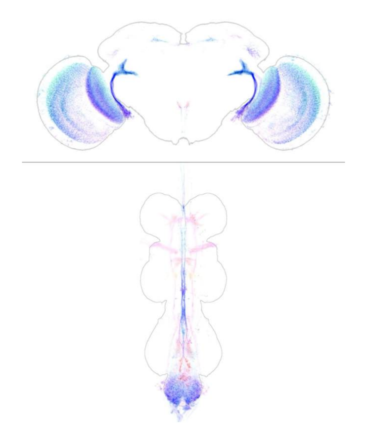
SS27830

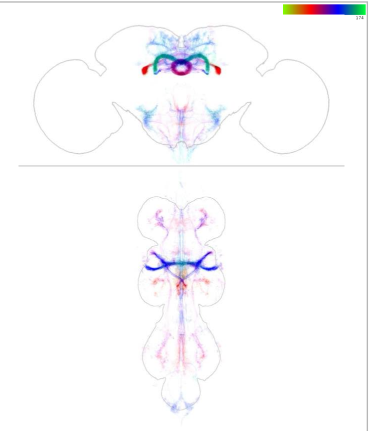

SS27853

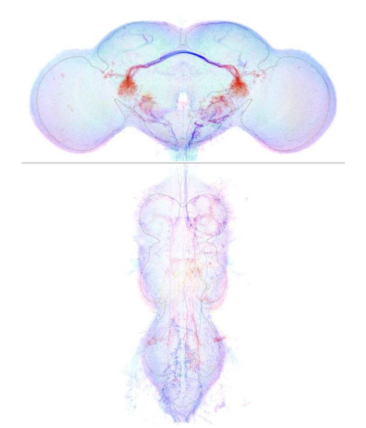

SS27953

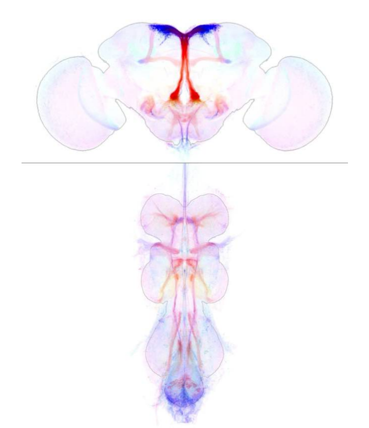

SS27959

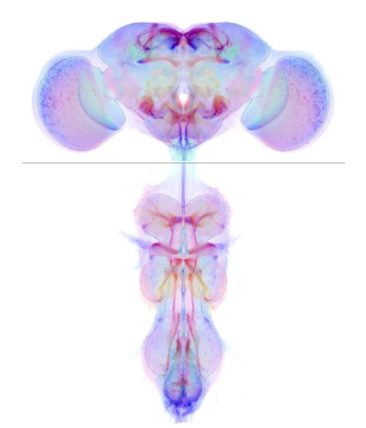

SS27970

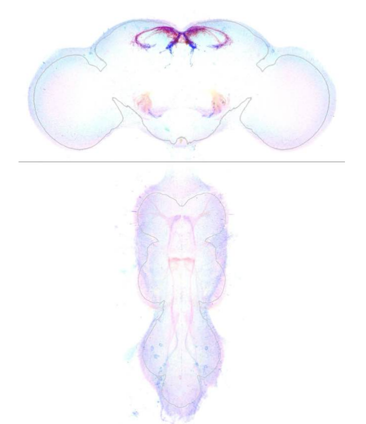

SS27991

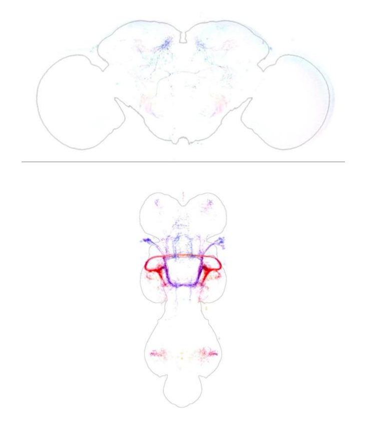

SS28003

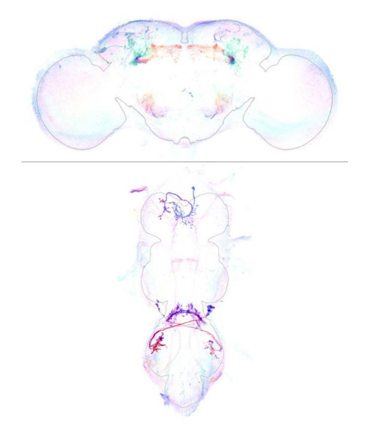

SS28010

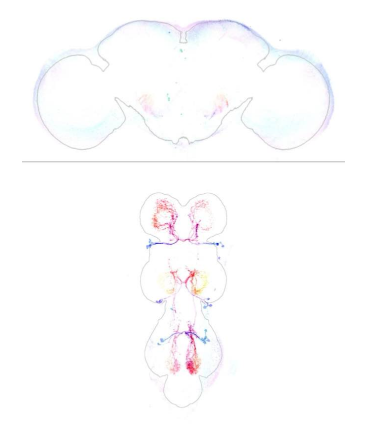

SS28035

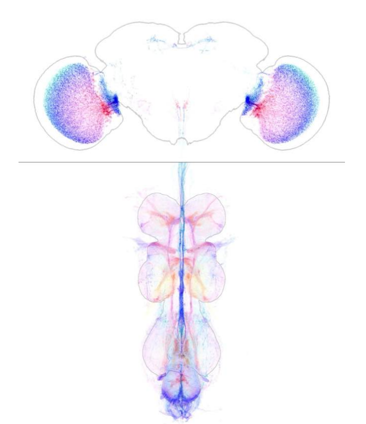

SS28071

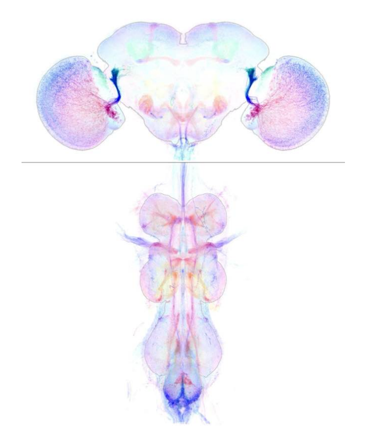

SS28175

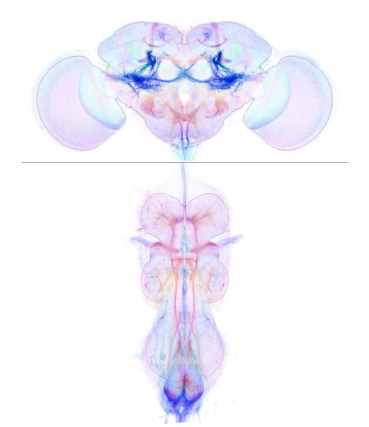

SS28218

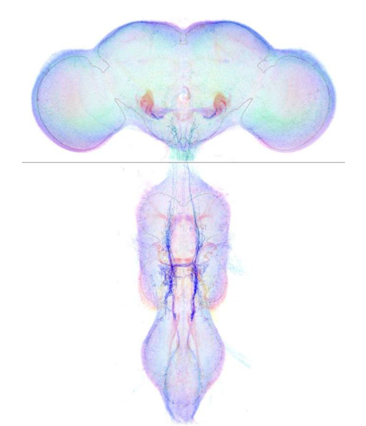

SS28361

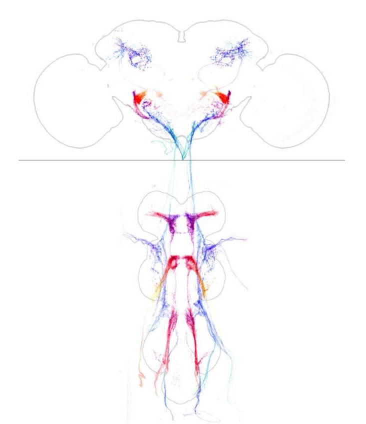

SS28382

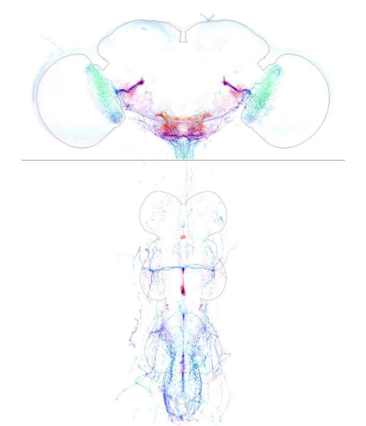

SS28428

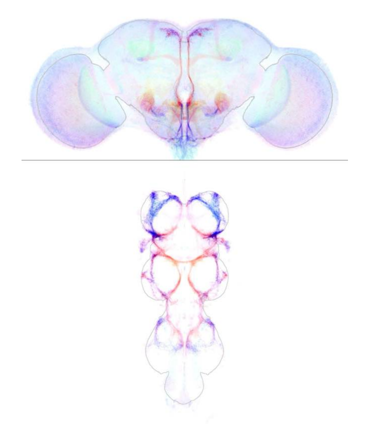

SS28453

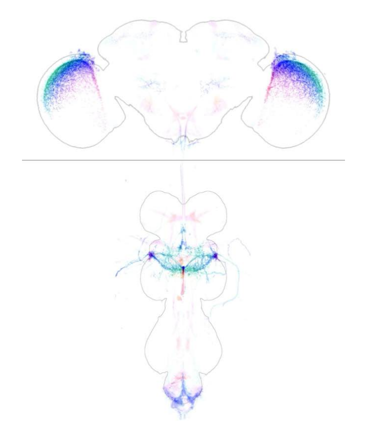

SS28476

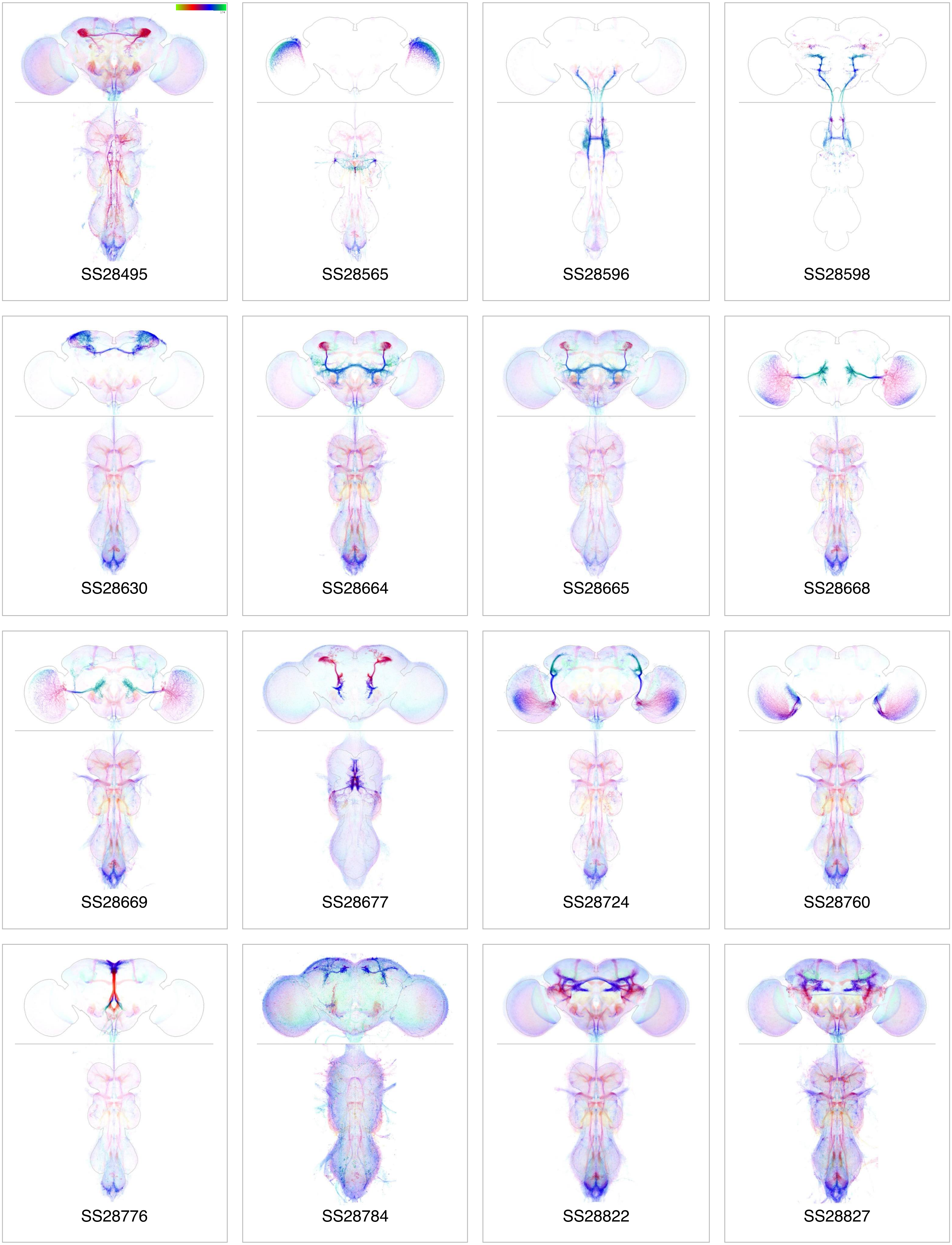
SS28495
SS28565
SS28596
SS28598
SS28630
SS28664
SS28665
SS28668
SS28669
SS28677
SS28724
SS28760
SS28776
SS28784
SS28822
SS28827

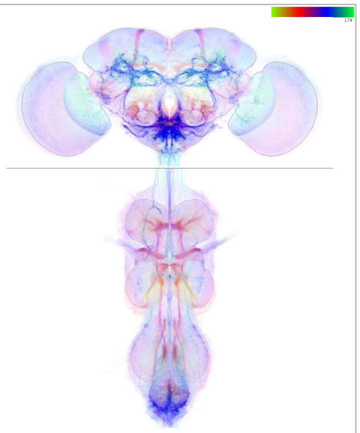

SS28878

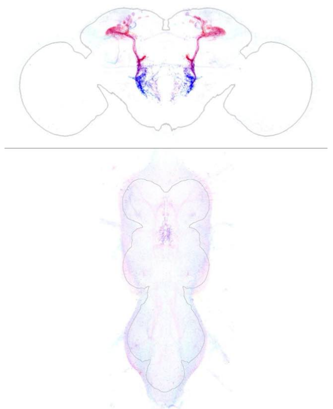

SS28933

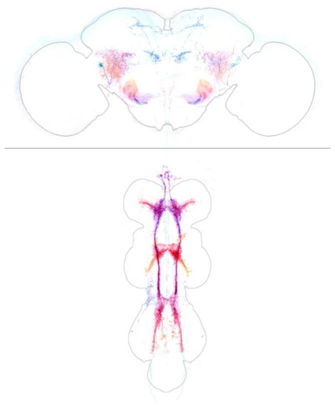

SS28943

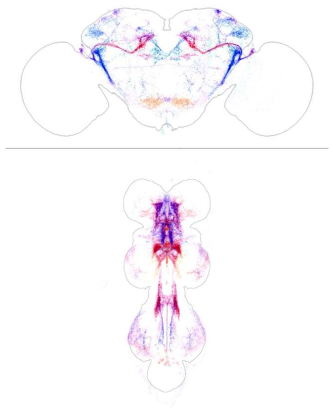

SS28945

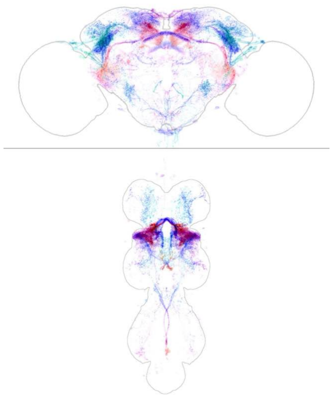

SS28951

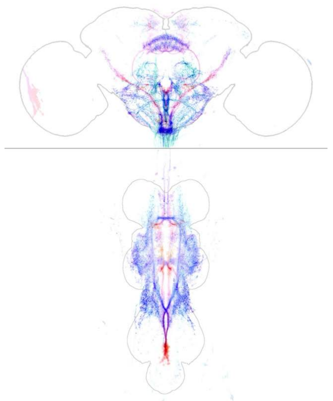

SS28953

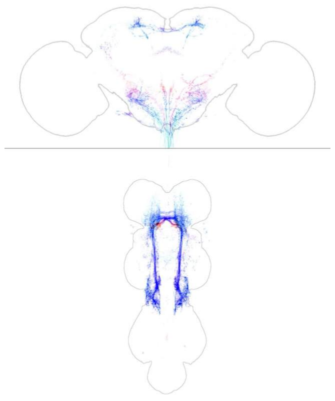

SS28954

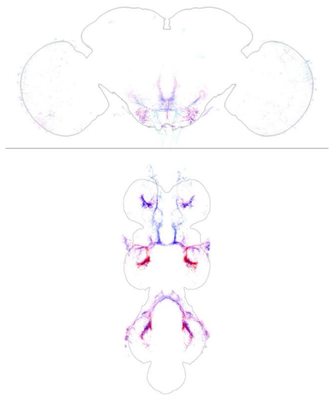

SS28958

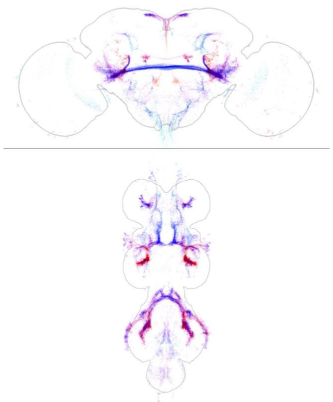

SS28961

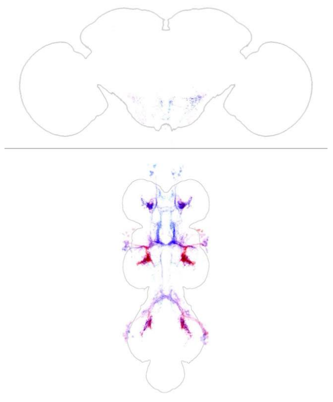

SS28963

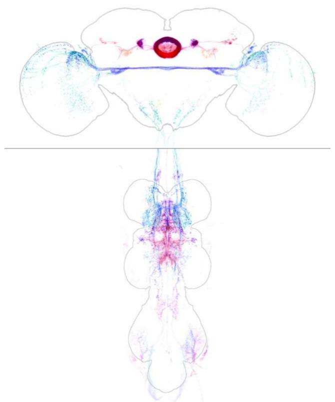

SS28965

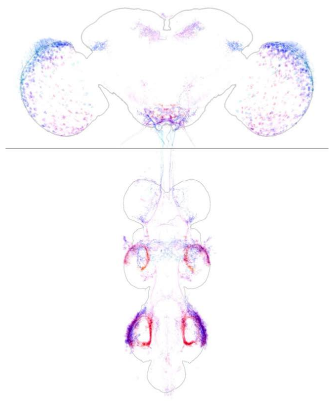

SS28966

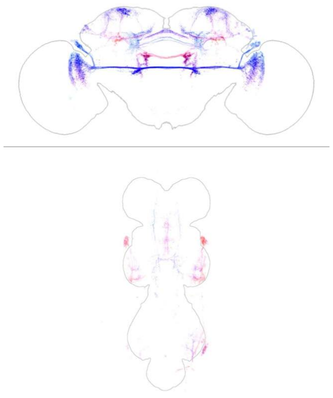

SS28969

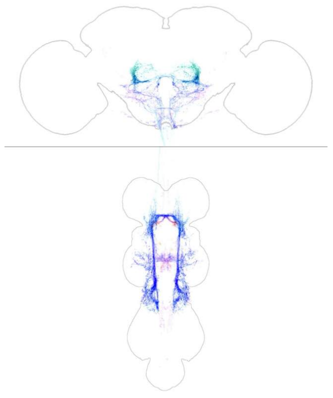

SS28970

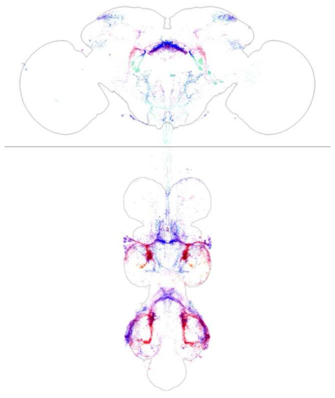

SS28975

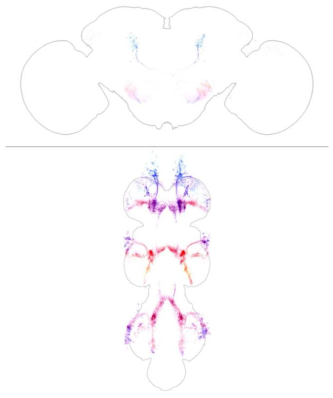

SS28977

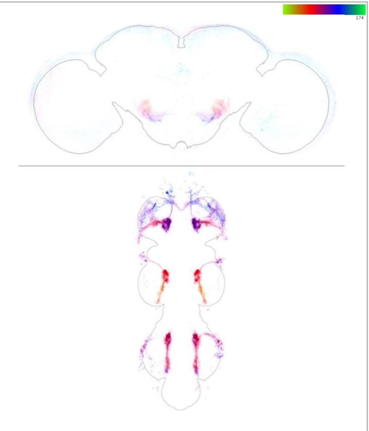

SS28980

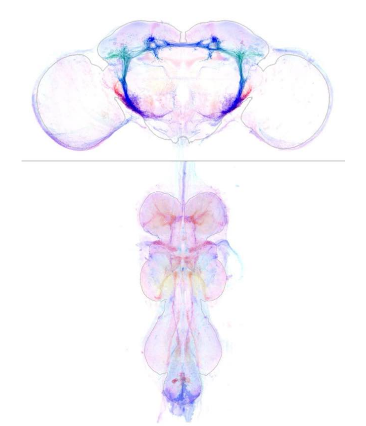

SS29118

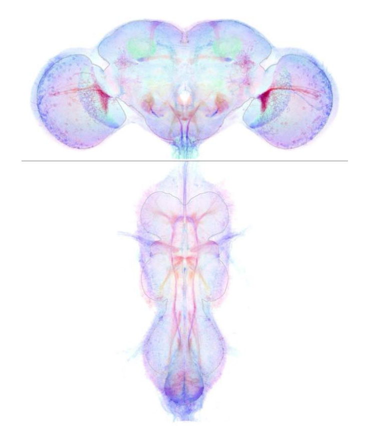

SS29128

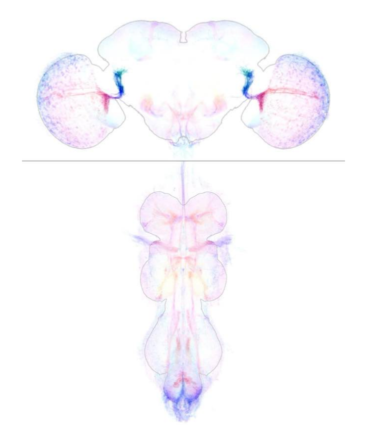

SS29129

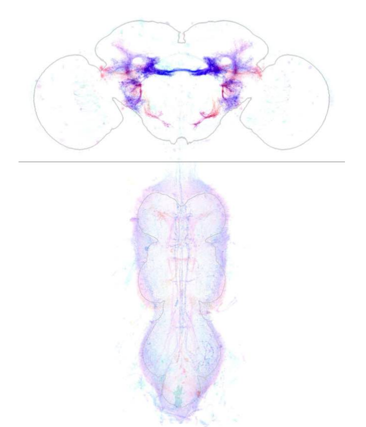

SS29136

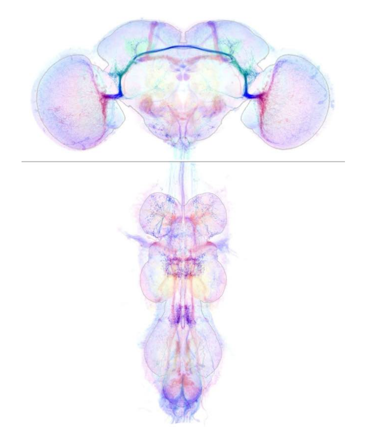

SS29143

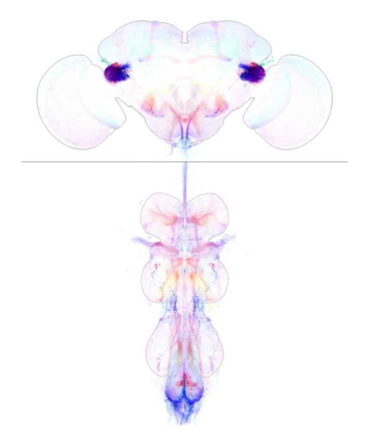

SS29144

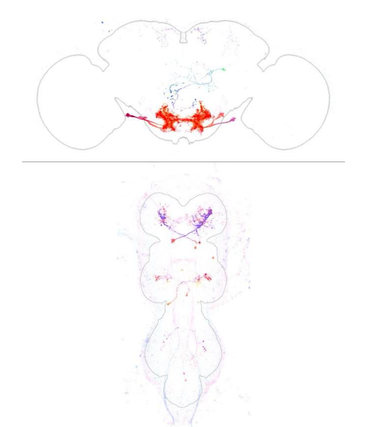

SS29243

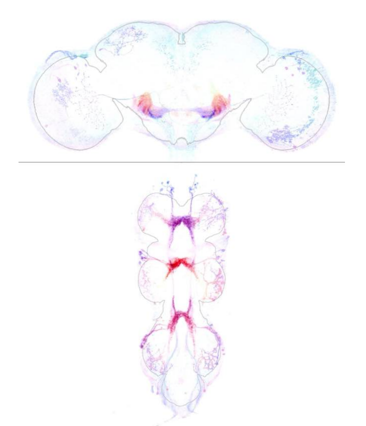

SS29258

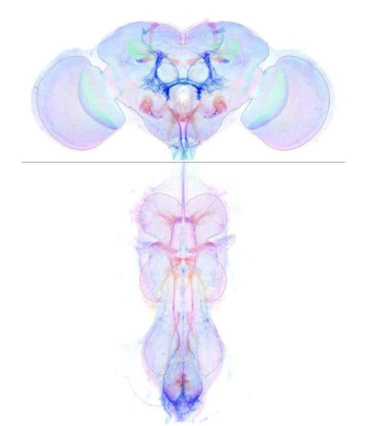

SS29276

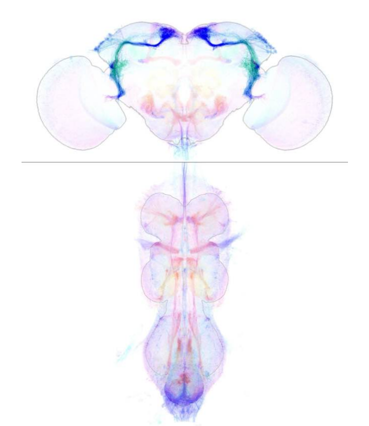

SS29337

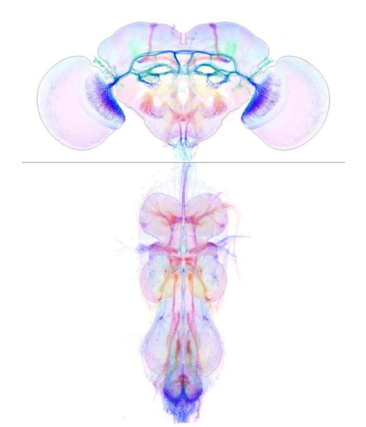

SS29397

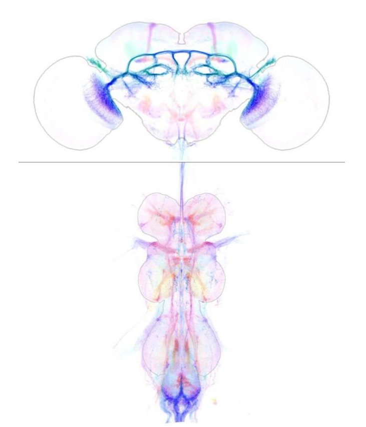

SS29399

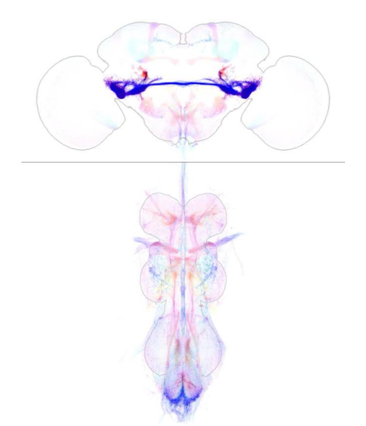

SS29404

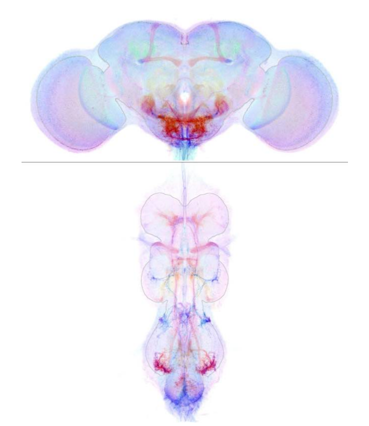

SS29466

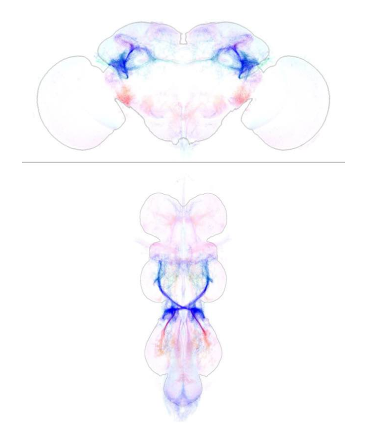

SS29535

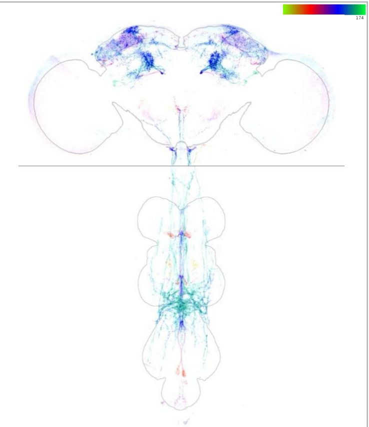
SS29579

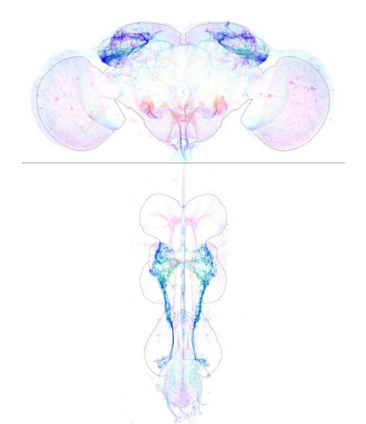
SS29600

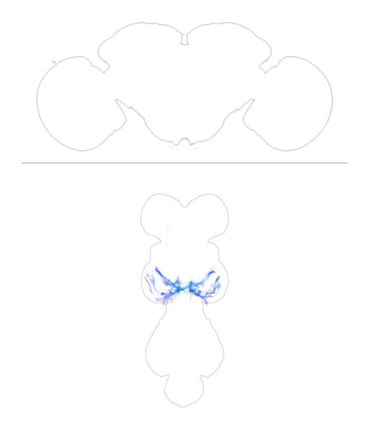
SS29602

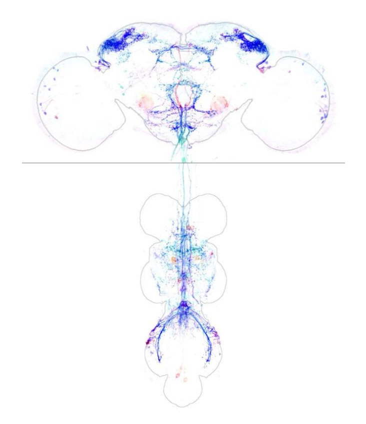
SS29621

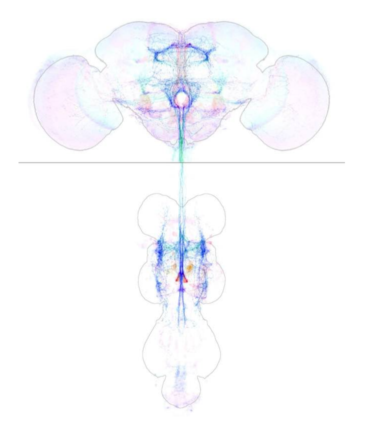
SS29633

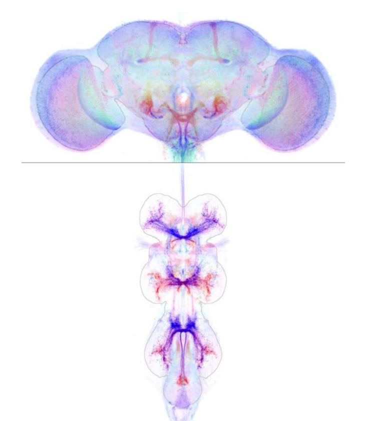
SS29662

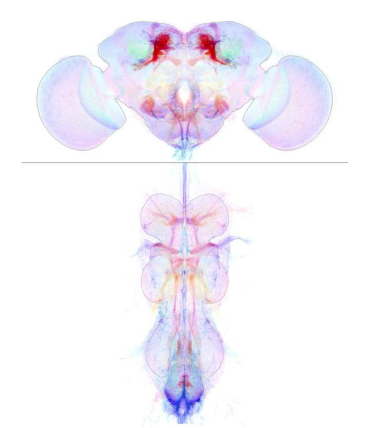
SS29675

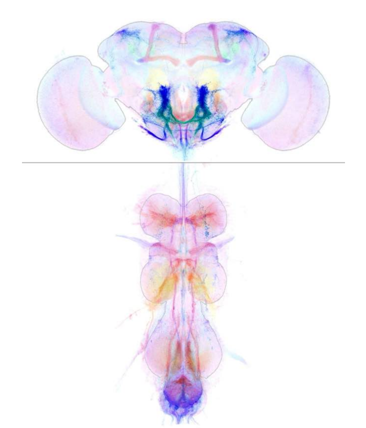
SS29687

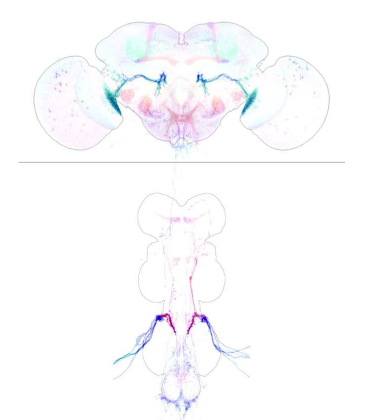
SS29802

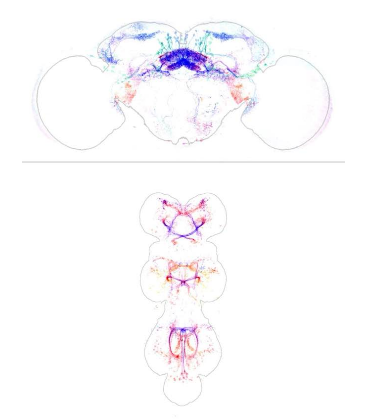
SS29857

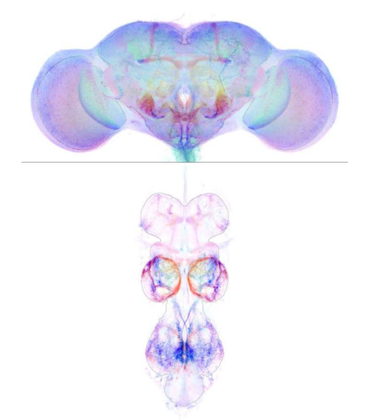
SS29871

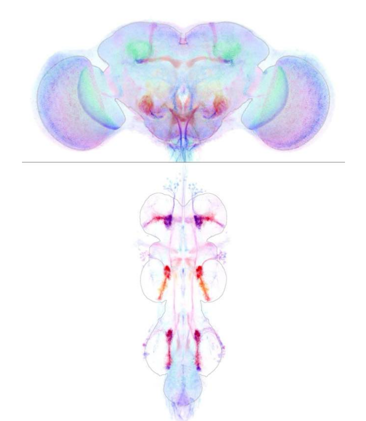
SS29882

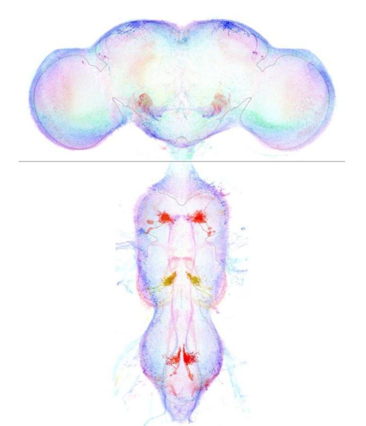
SS29886

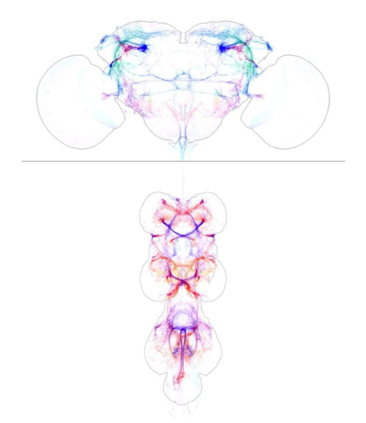
SS29892

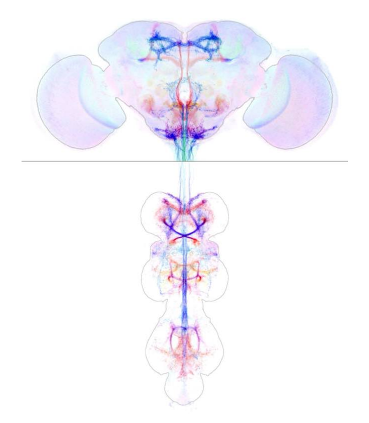
SS29893

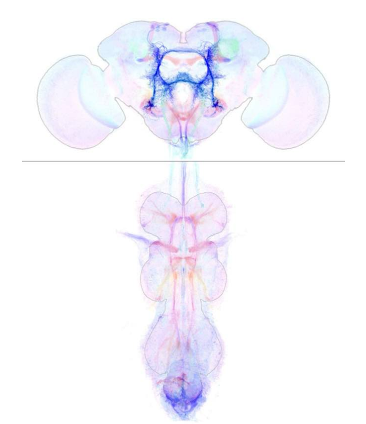
SS29911

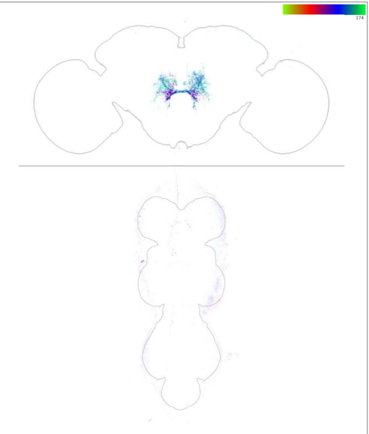
SS29923

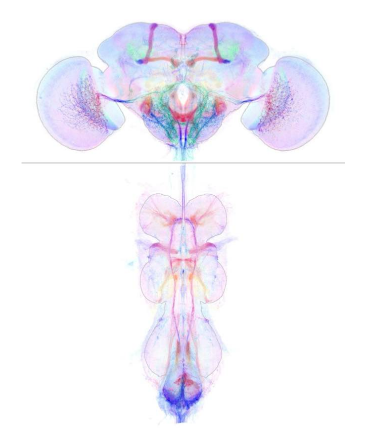
SS29970

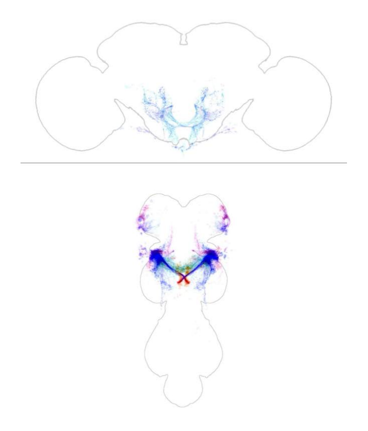
SS30185

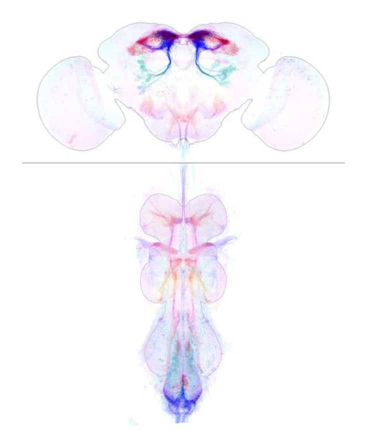
SS30195

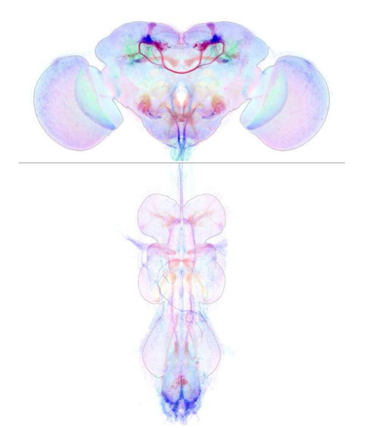
SS30198

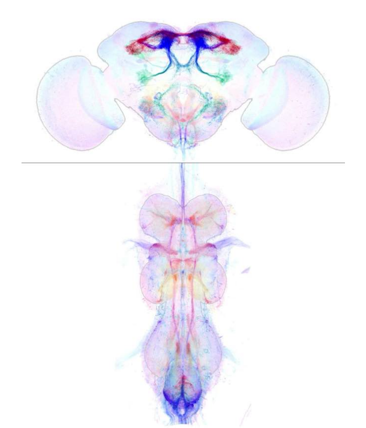
SS30234

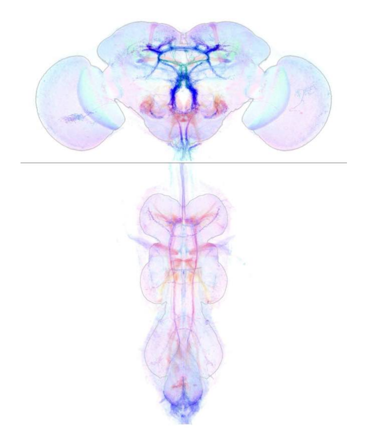
SS30265

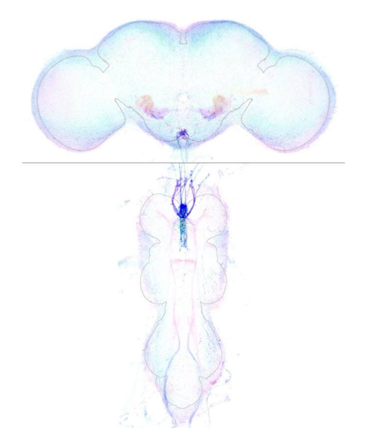
SS30303

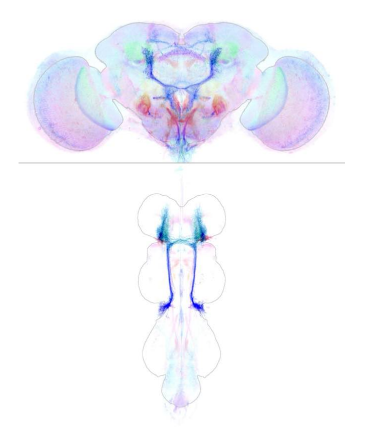
SS30330

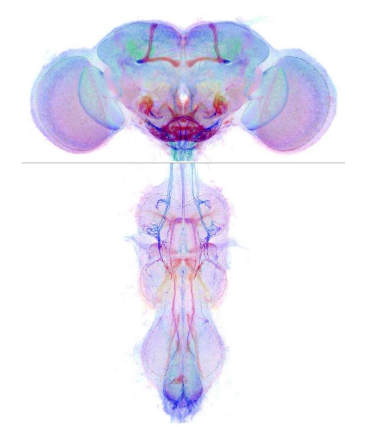
SS30373

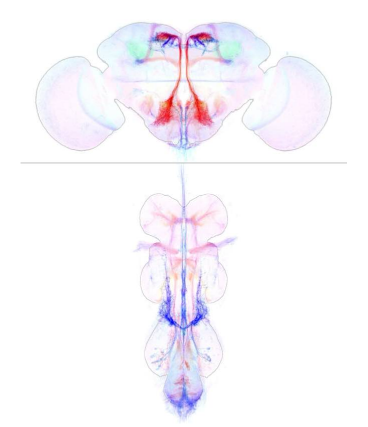
SS30409

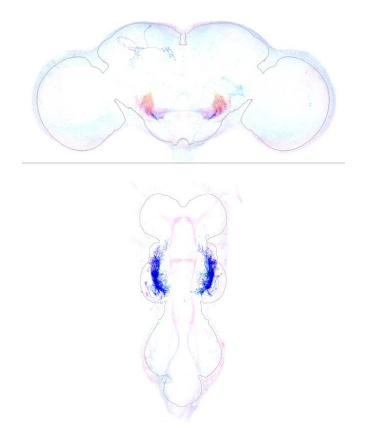
SS30784

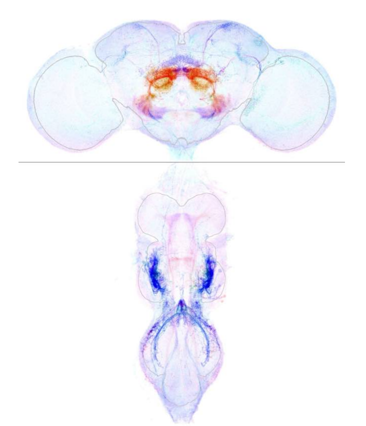
SS30787

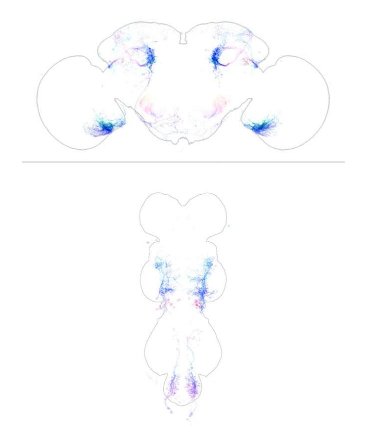
SS30790

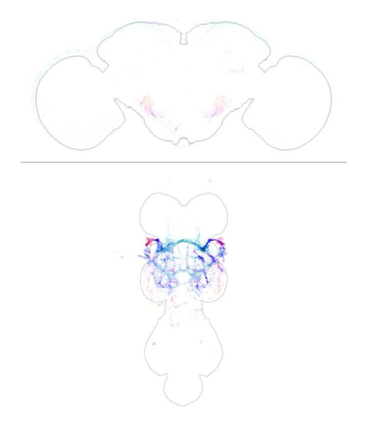
SS30793

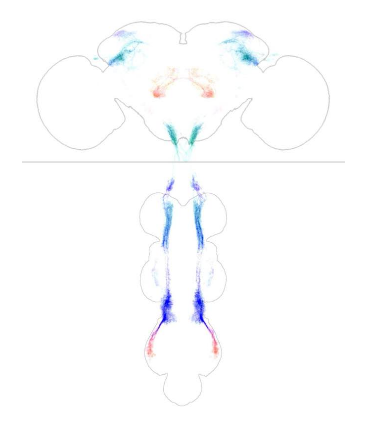
SS30798

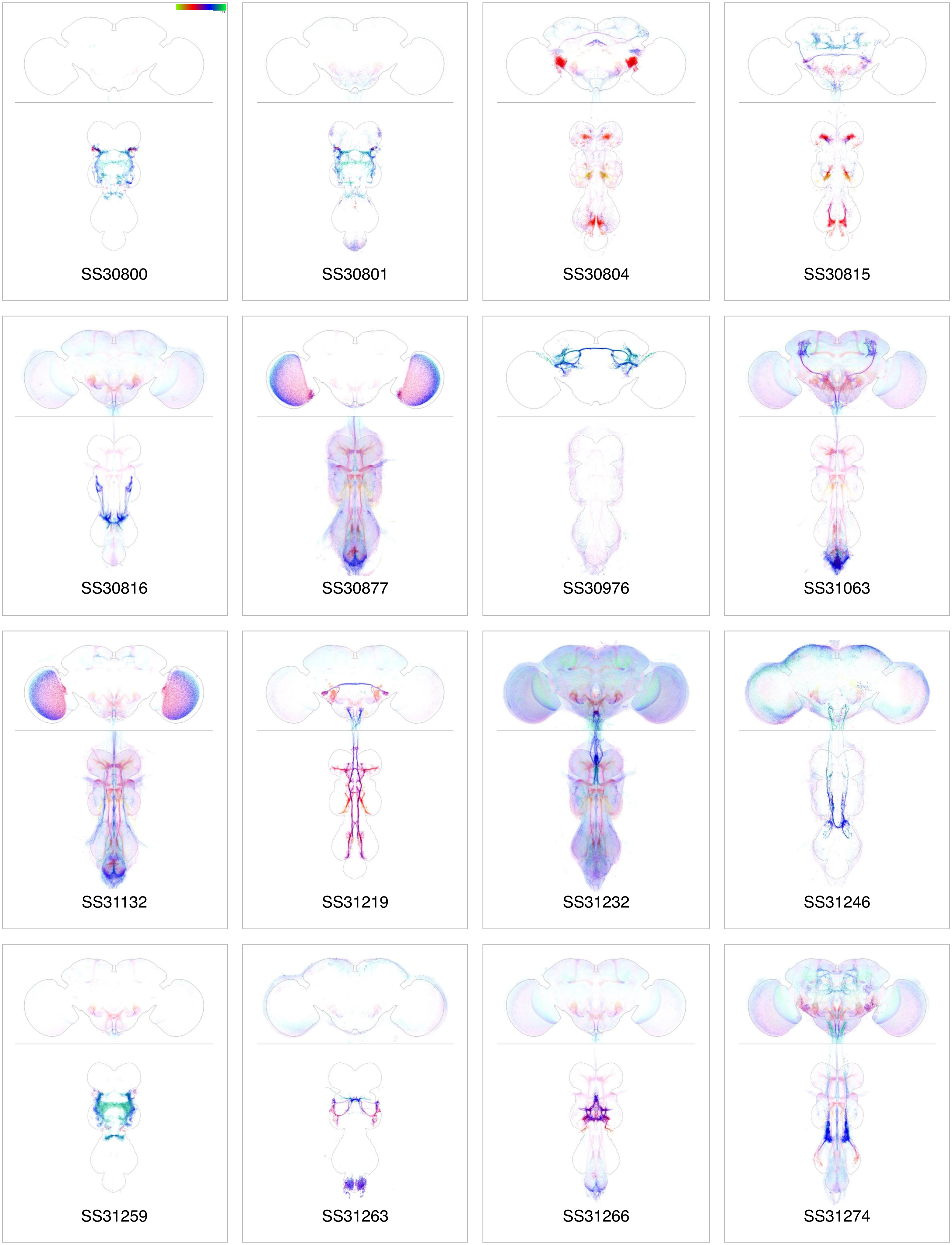
SS30800
SS30801
SS30804
SS30815
SS30816
SS30877
SS30976
SS31063
SS31132
SS31219
SS31232
SS31246
SS31259
SS31263
SS31266
SS31274

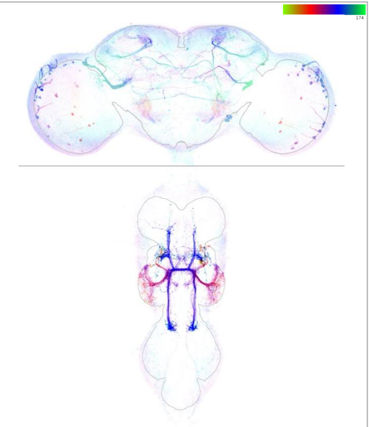

SS31289

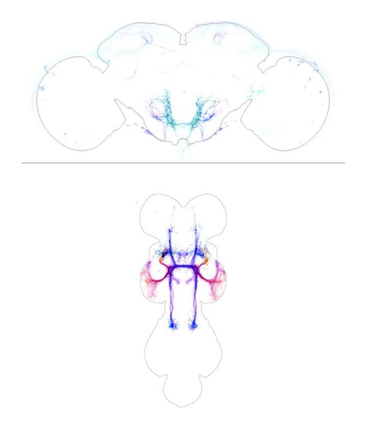

SS31290

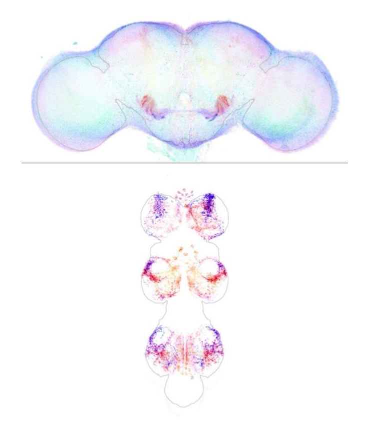

SS31305

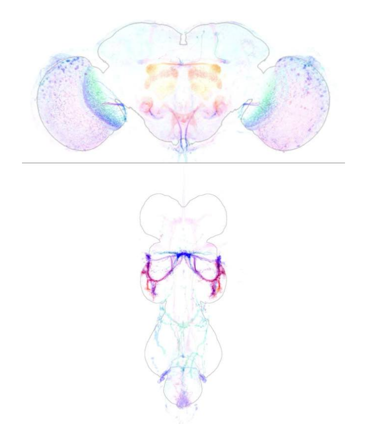

SS31309

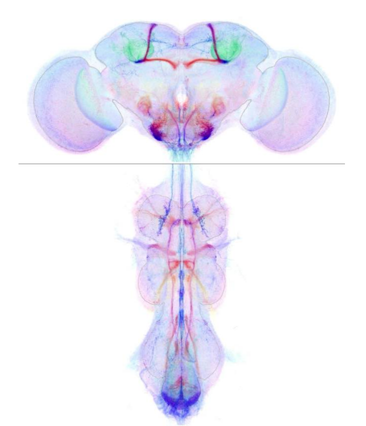

SS31386

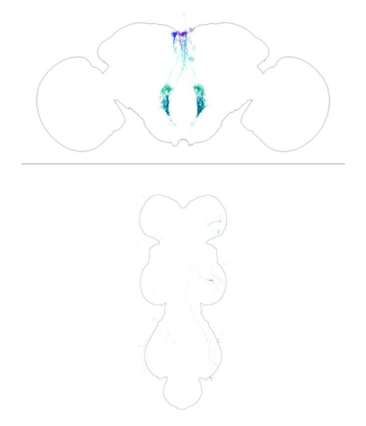

SS31450

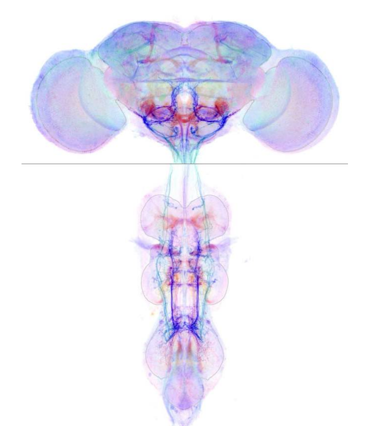

SS31456

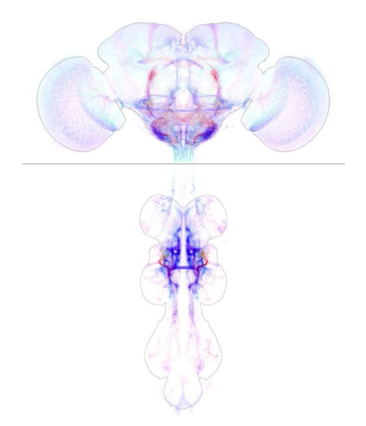

SS31465

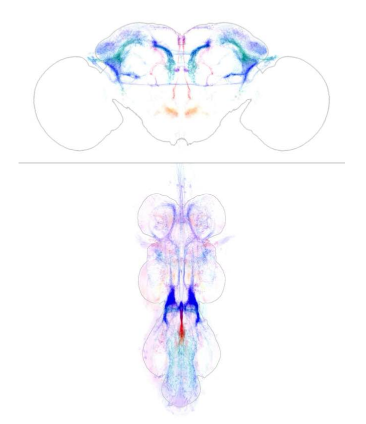

SS31466

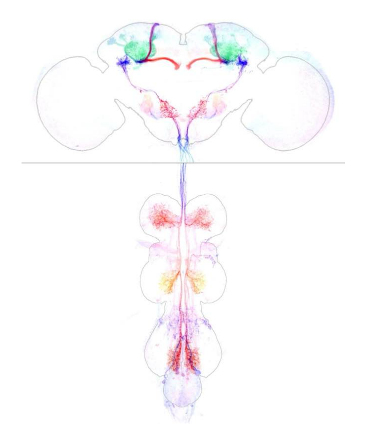

SS31480

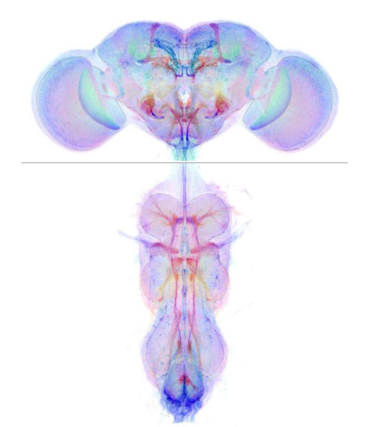

SS31505

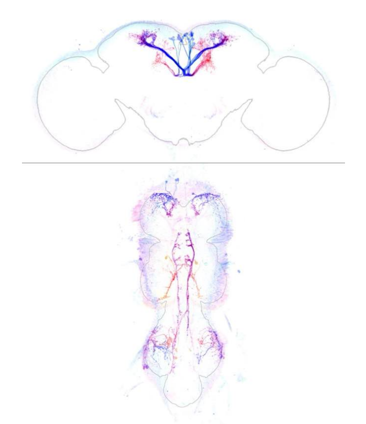

SS31522

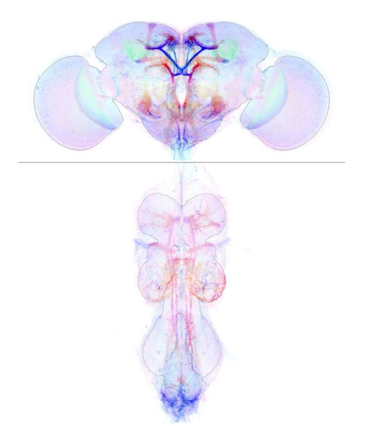

SS31523

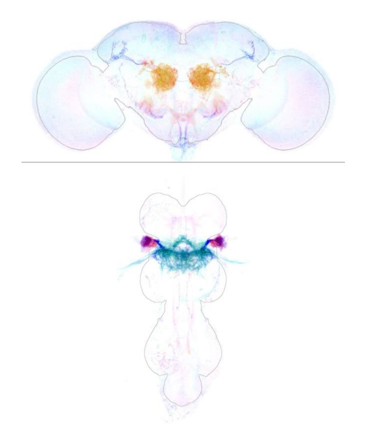

SS31541

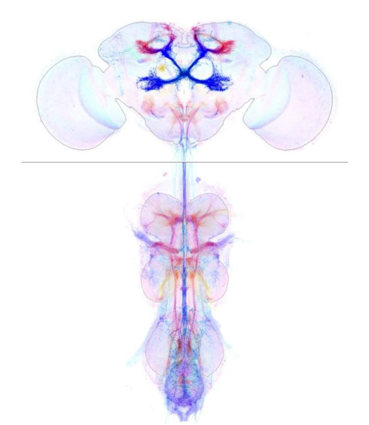

SS31659

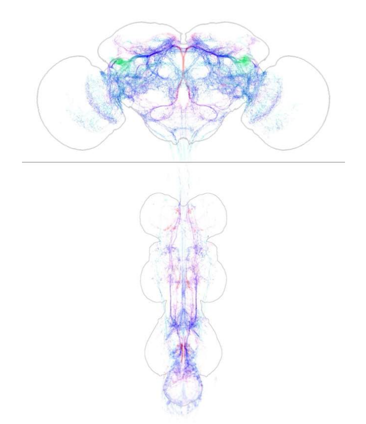

SS31662

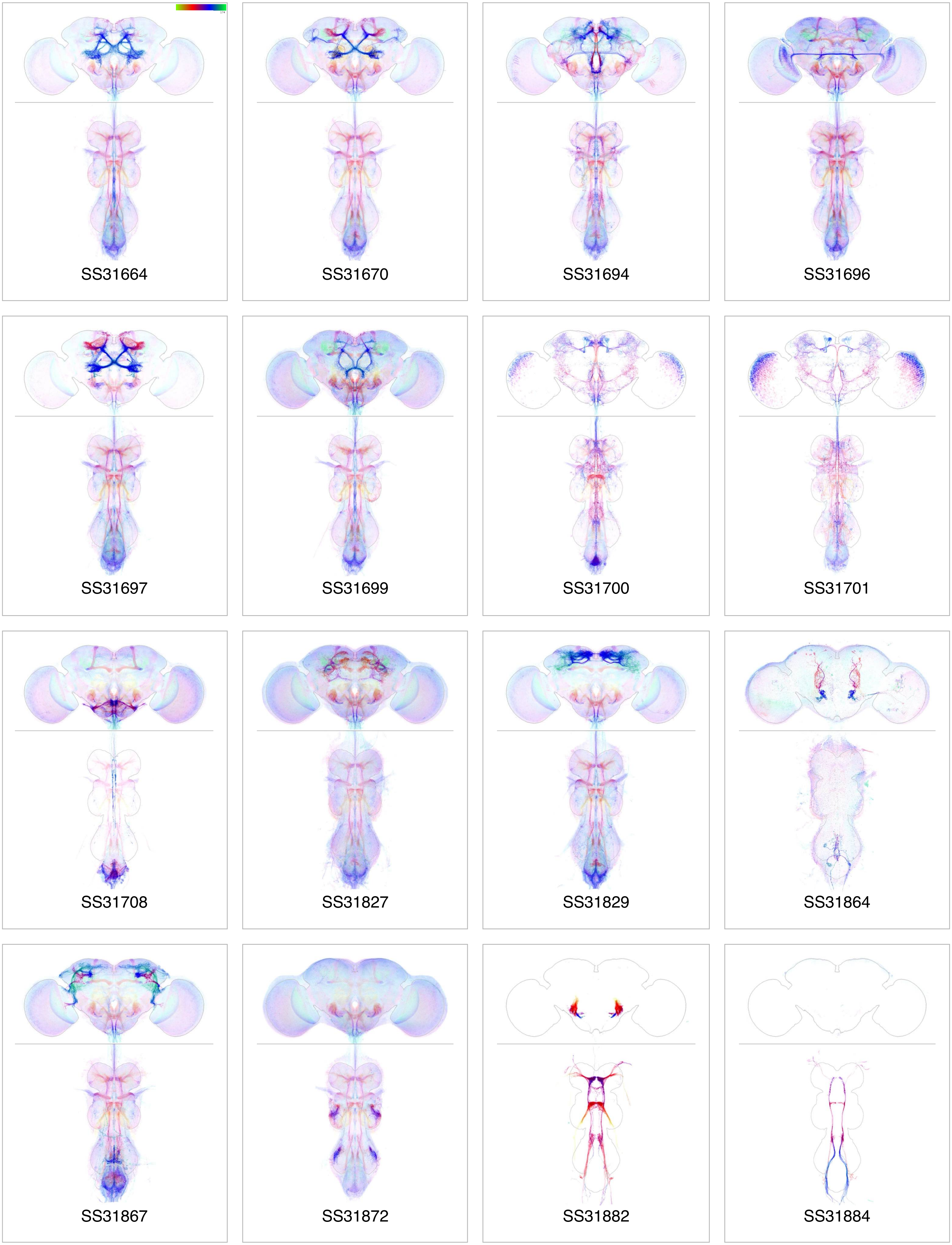
SS31664
SS31670
SS31694
SS31696
SS31697
SS31699
SS31700
SS31701
SS31708
SS31827
SS31829
SS31864
SS31867
SS31872
SS31882
SS31884

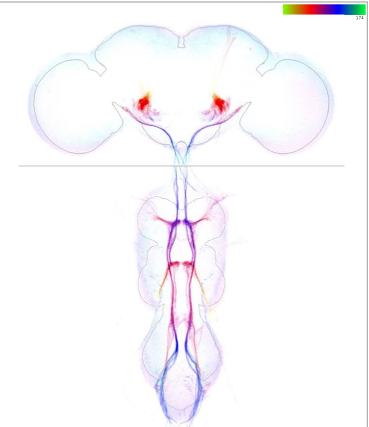
SS31895

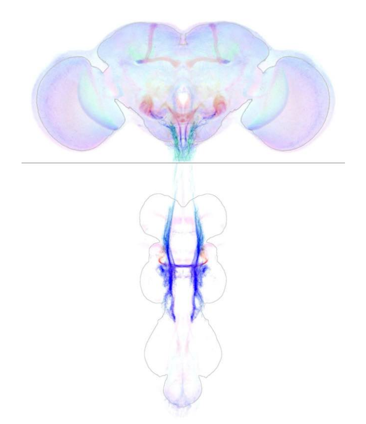
SS31899

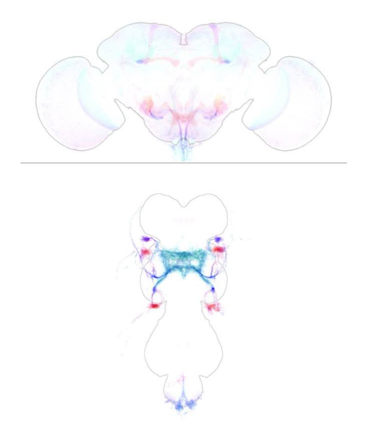
SS31935

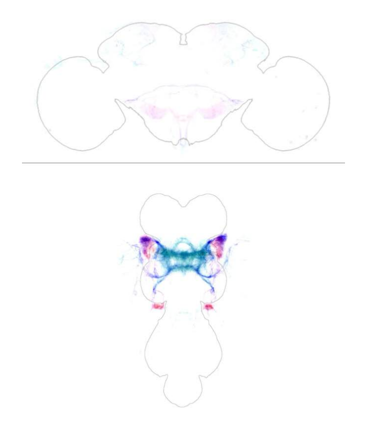
SS31950

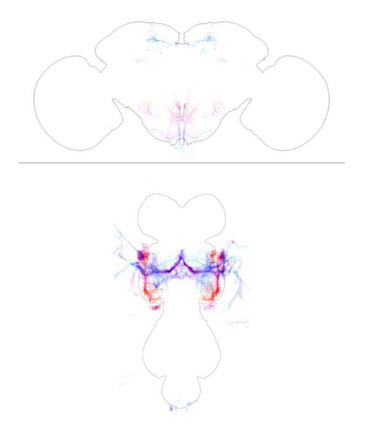
SS31976

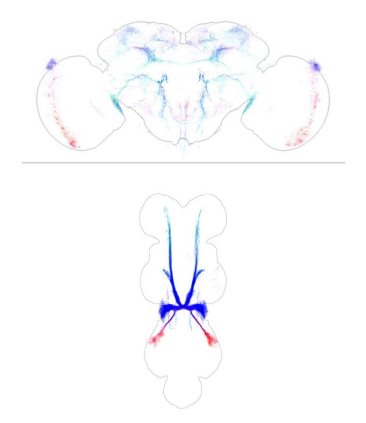
SS31986

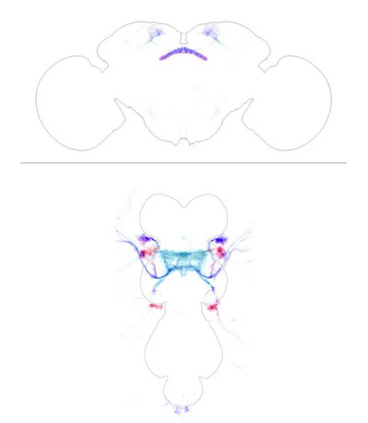
SS31997

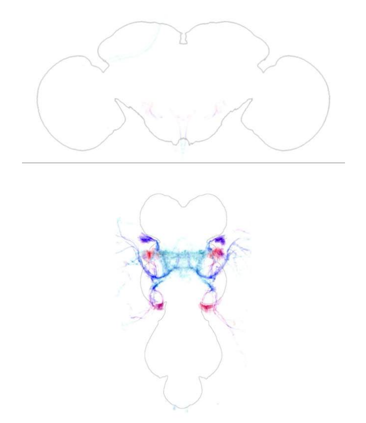
SS31999

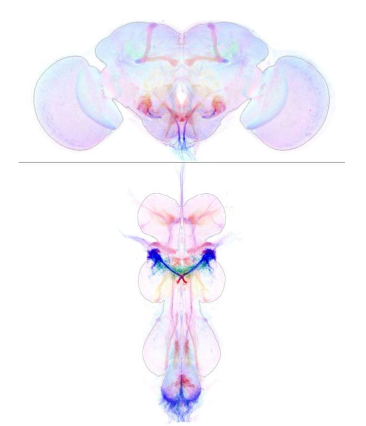
SS32008

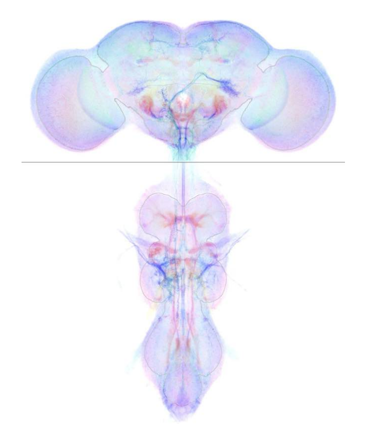
SS32023

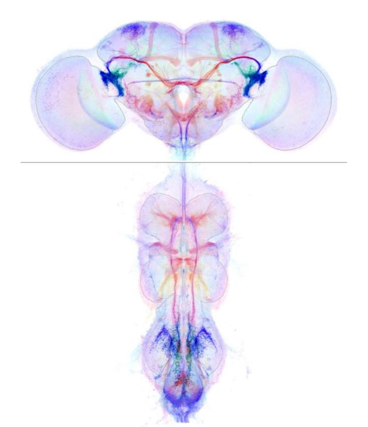
SS32025

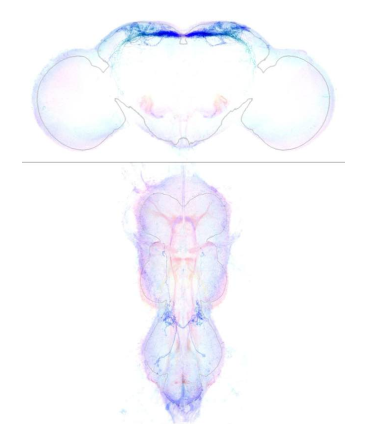
SS32038

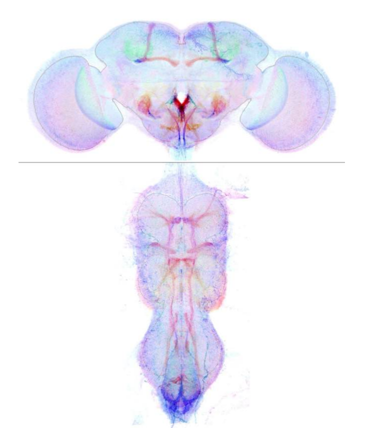
SS32066

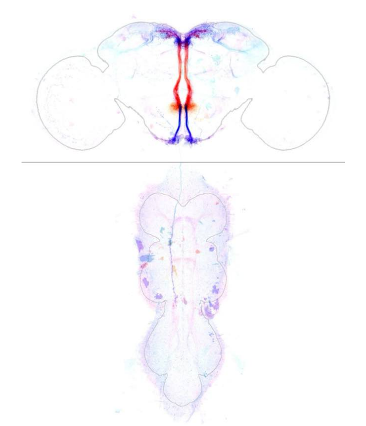
SS32119

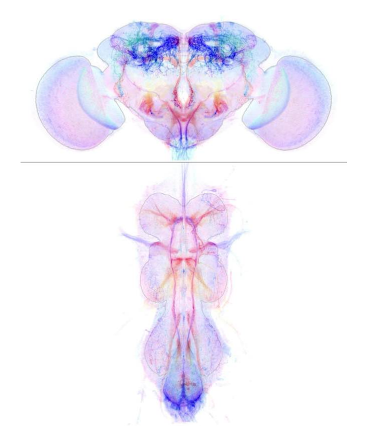
SS32144

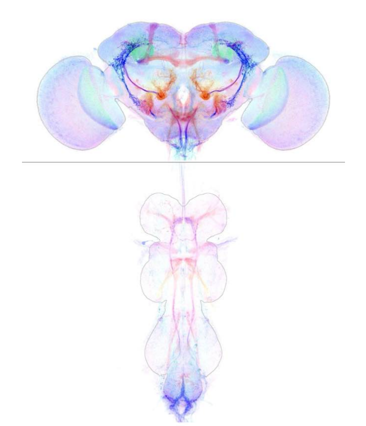
SS32193

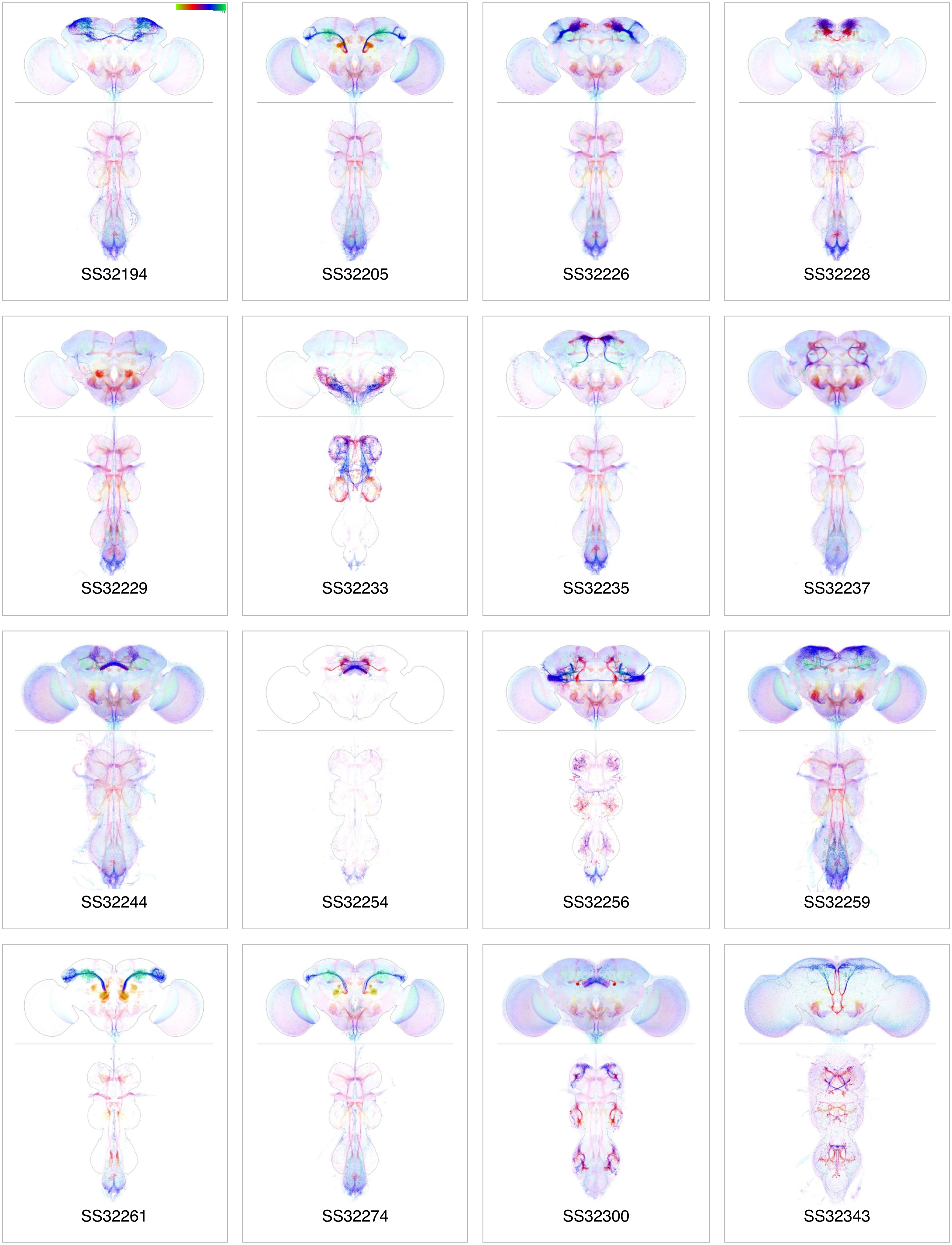
SS32194
SS32205
SS32226
SS32228
SS32229
SS32233
SS32235
SS32237
SS32244
SS32254
SS32256
SS32259
SS32261
SS32274
SS32300
SS32343

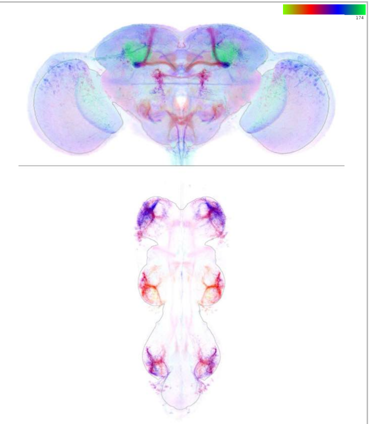
SS32346

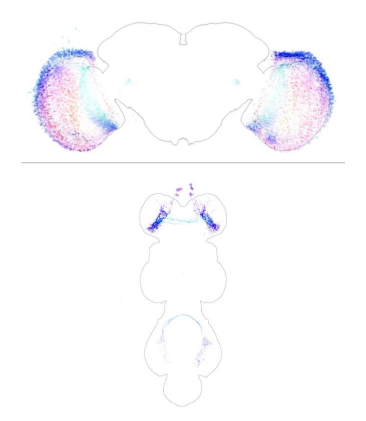
SS32370

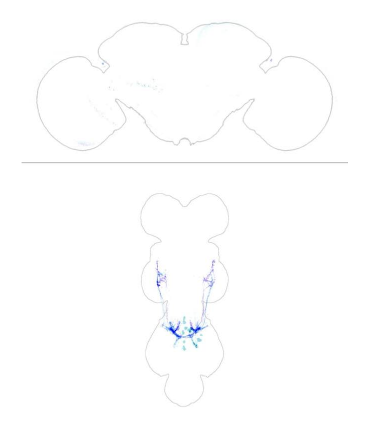
SS32377

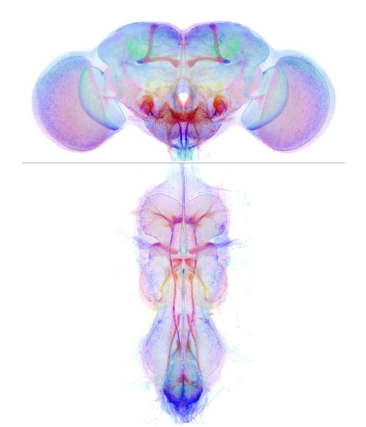
SS32391

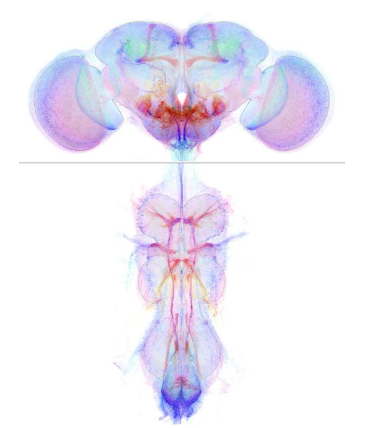
SS32394

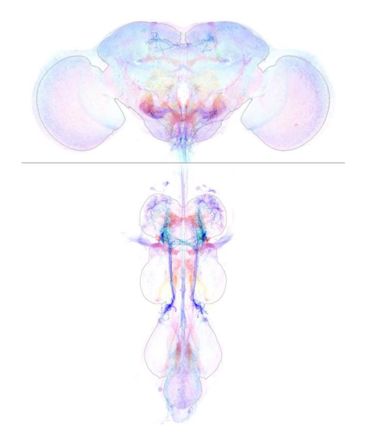
SS32400

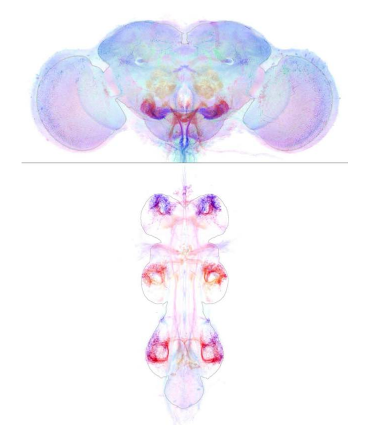
SS32427

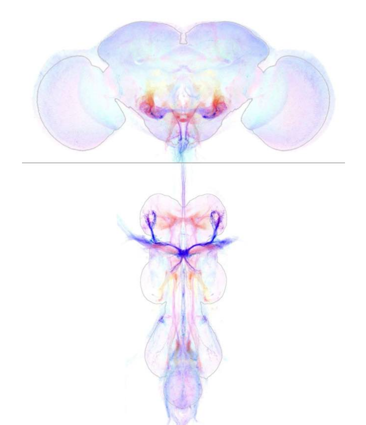
SS32454

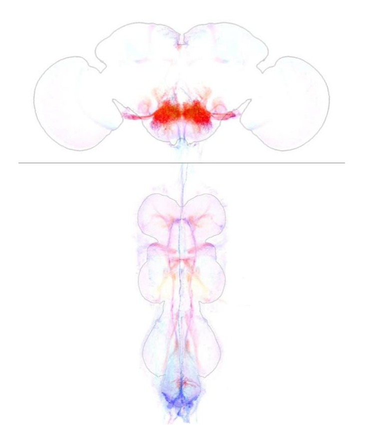
SS32463

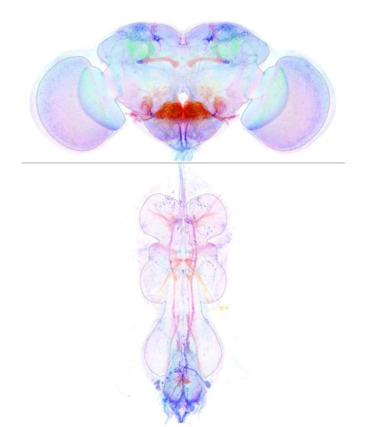
SS32464

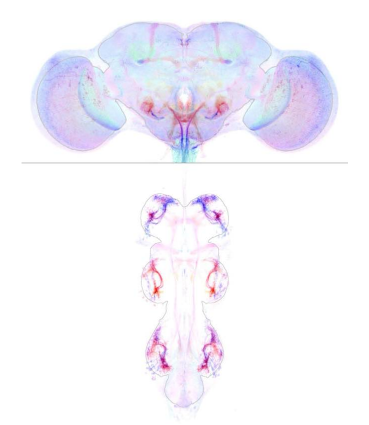
SS32496

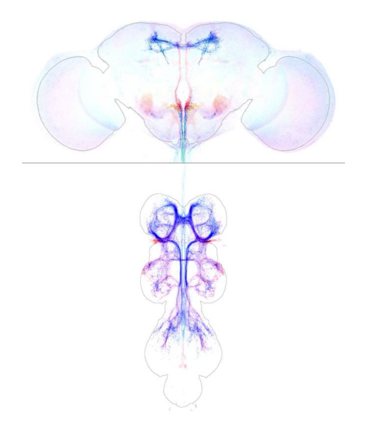
SS32504

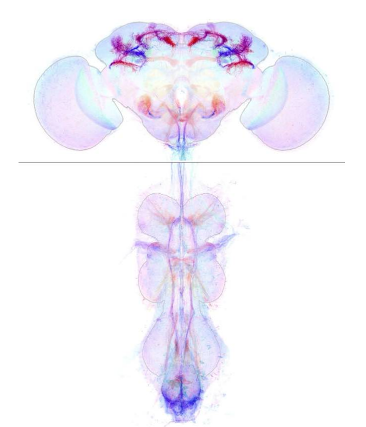
SS32525

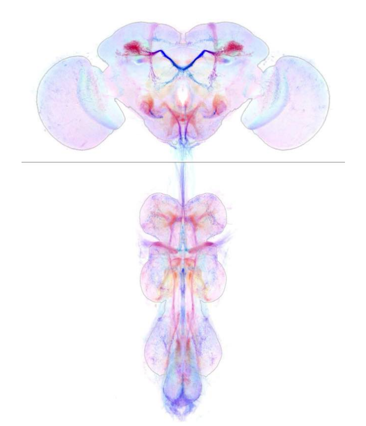
SS32548

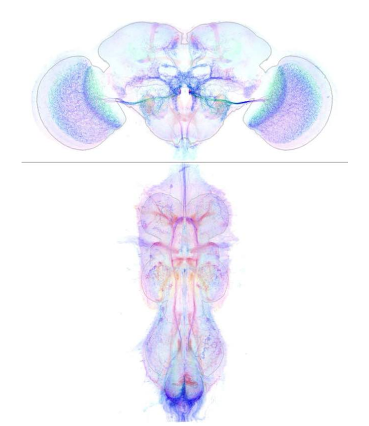
SS32579

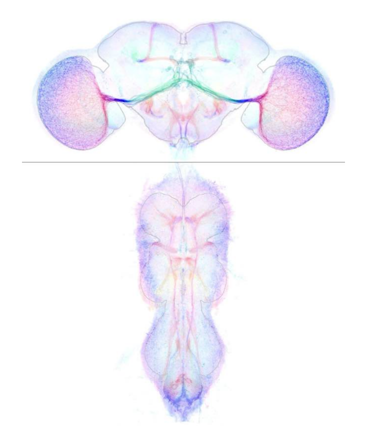
SS32604

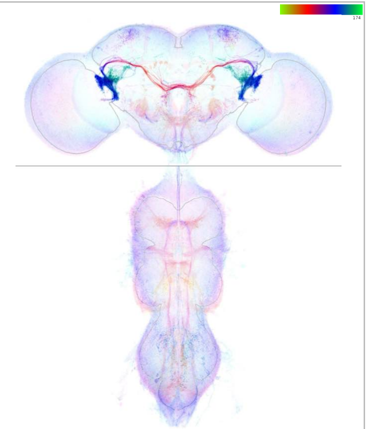

SS32665

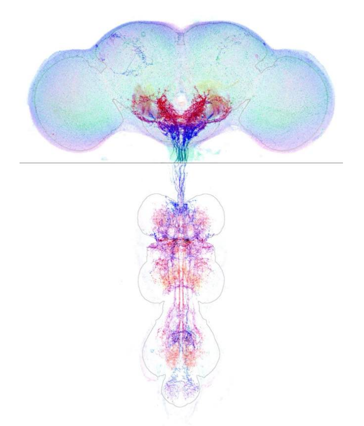

SS32727

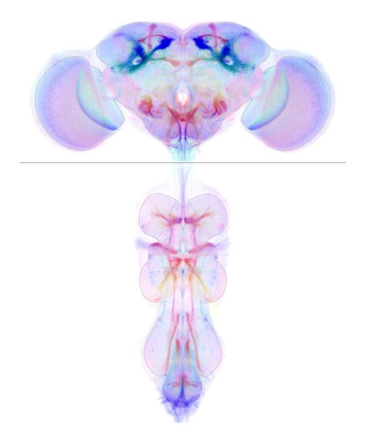

SS32768

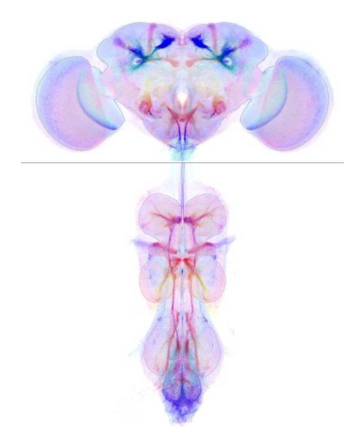

SS32774

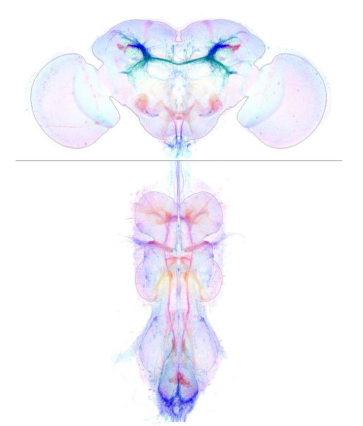

SS32791

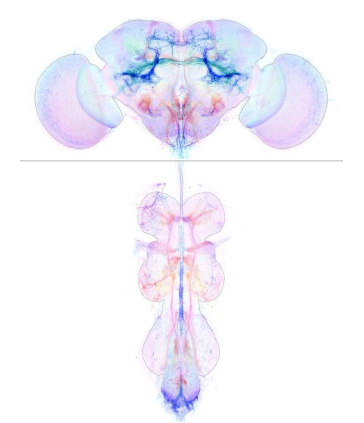

SS32792

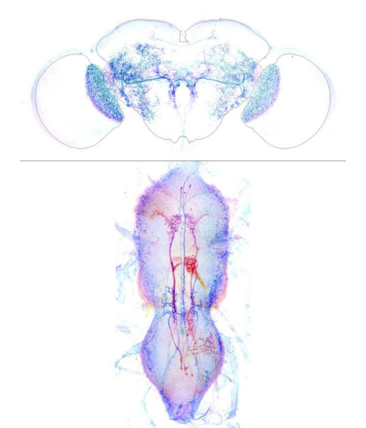

SS32832

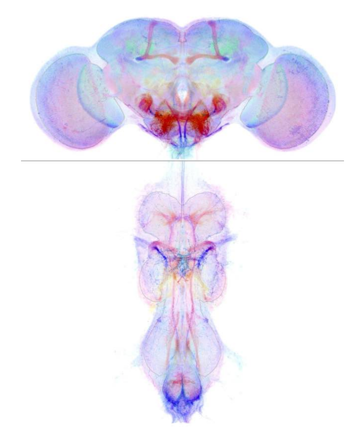

SS32949

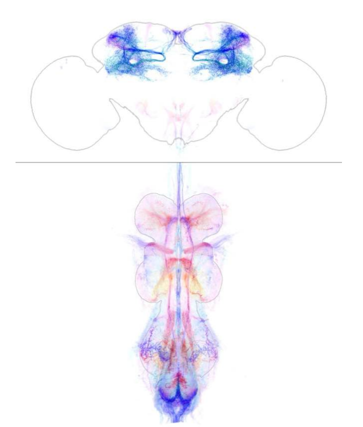

SS32986

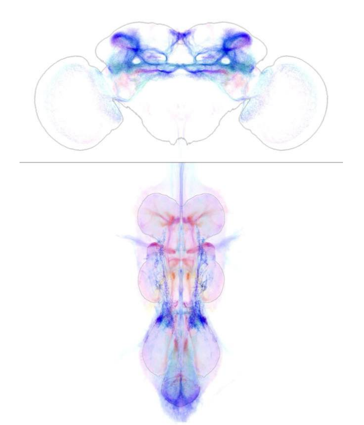

SS33007

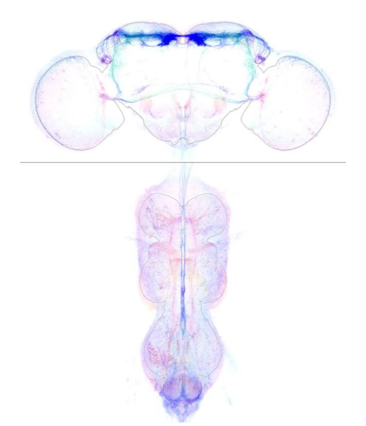

SS33031

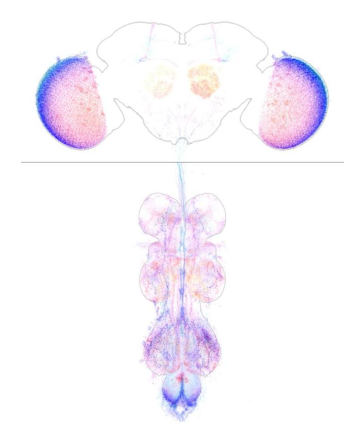

SS33037

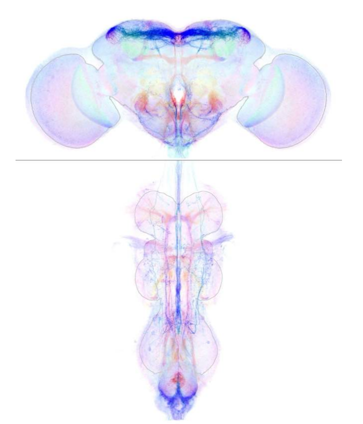

SS33061

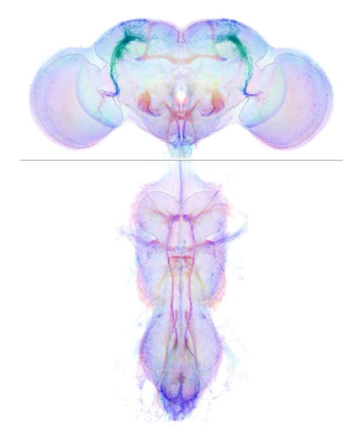

SS33078

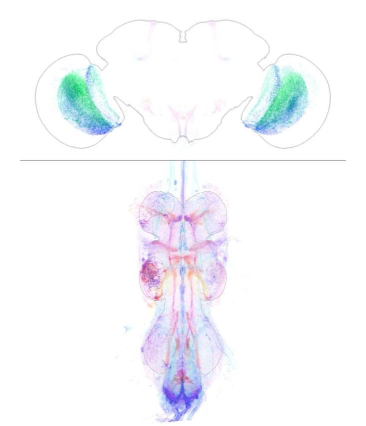

SS33166

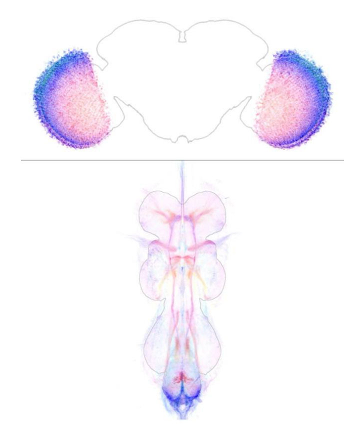

SS33187

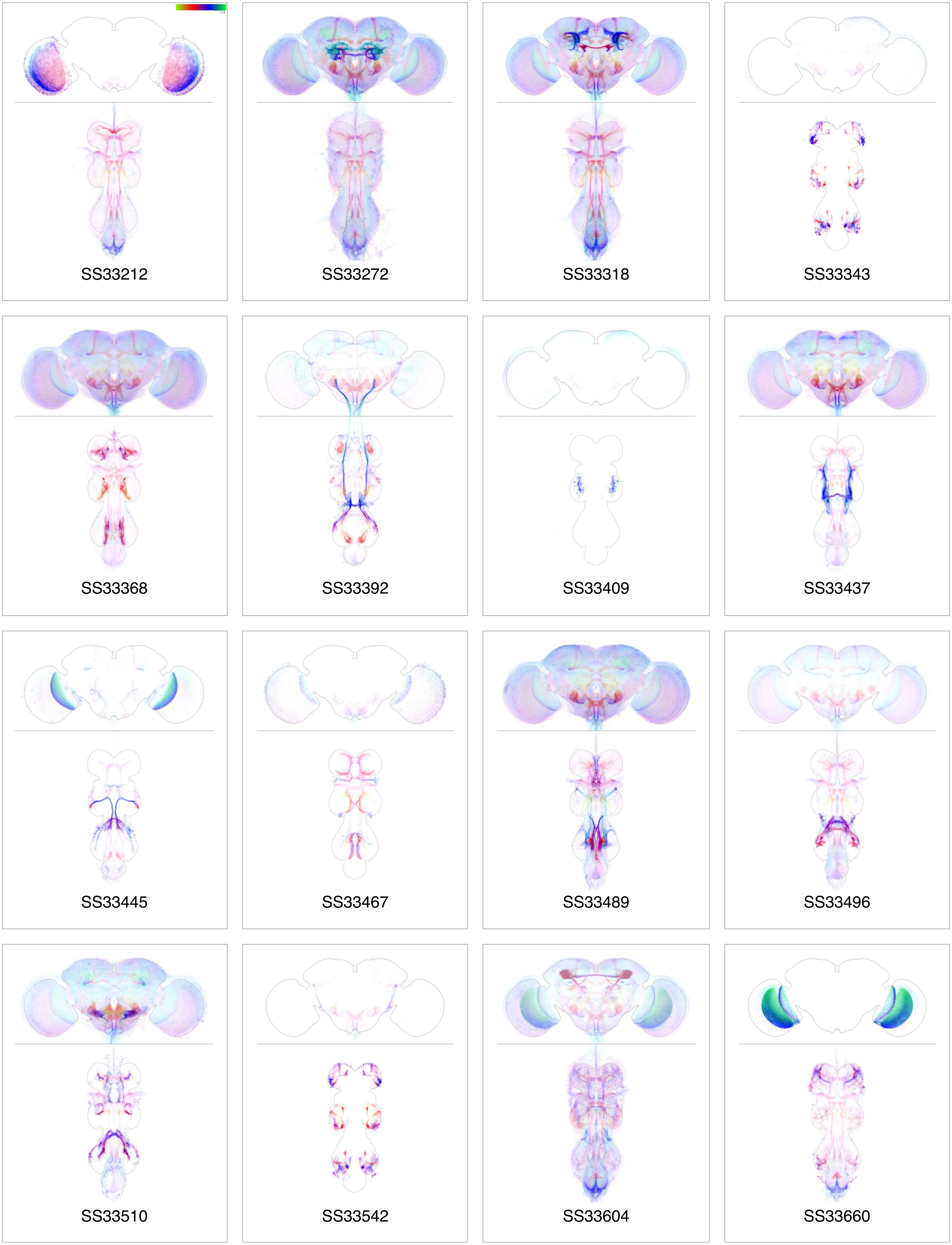
SS33212
SS33272
SS33318
SS33343
SS33368
SS33392
SS33409
SS33437
SS33445
SS33467
SS33489
SS33496
SS33510
SS33542
SS33604
SS33660

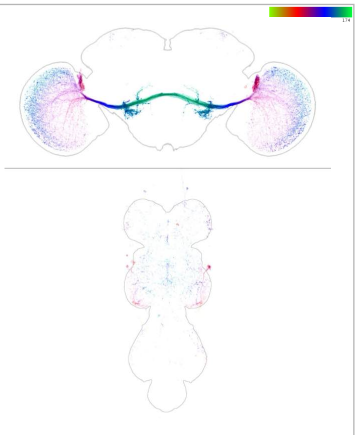
SS33663

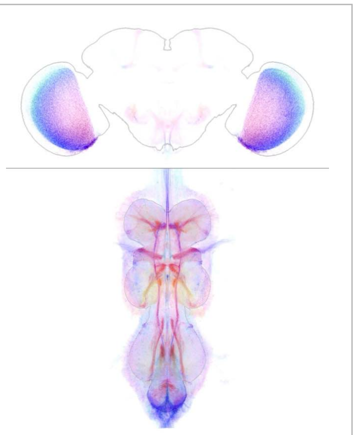
SS33673

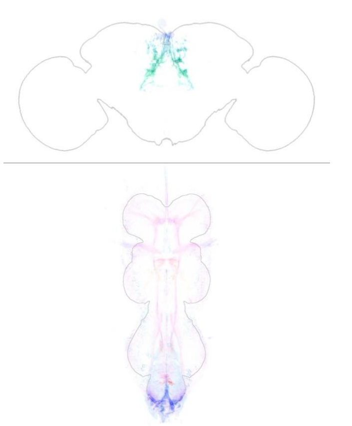
SS33693

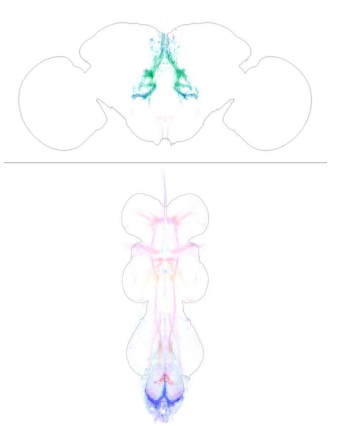
SS33694

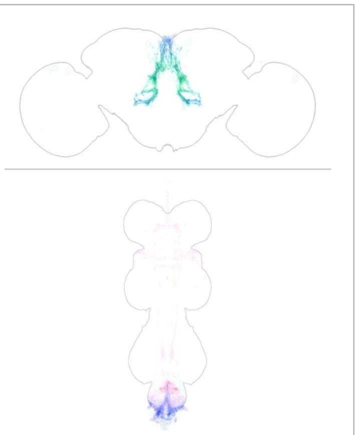
SS33707

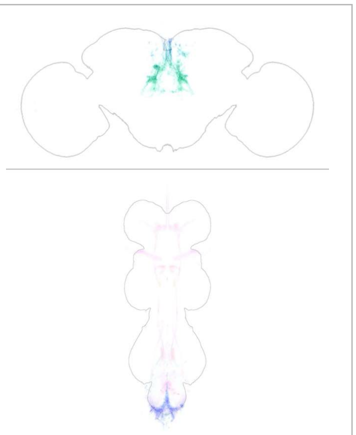
SS33738

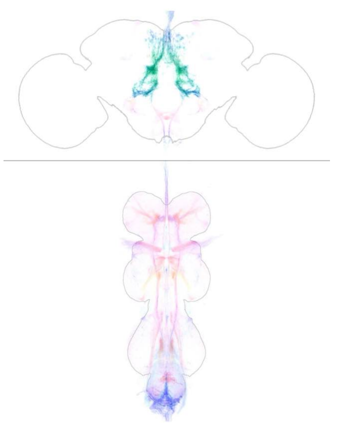
SS33739

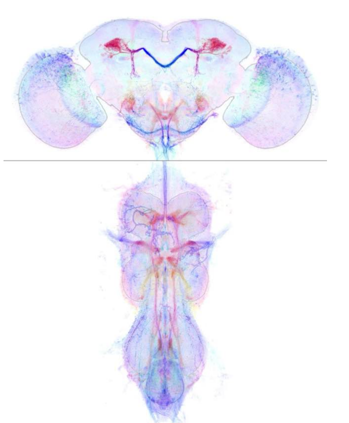
SS33872

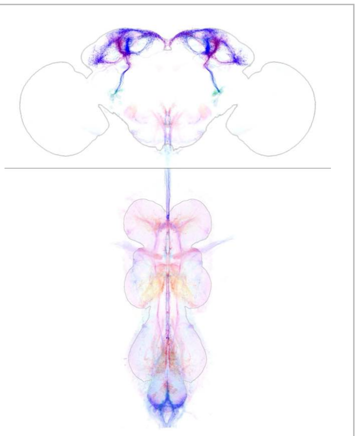
SS33905

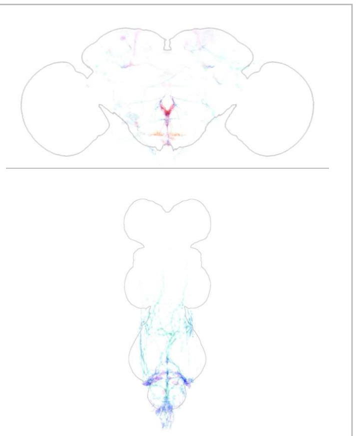
SS33908

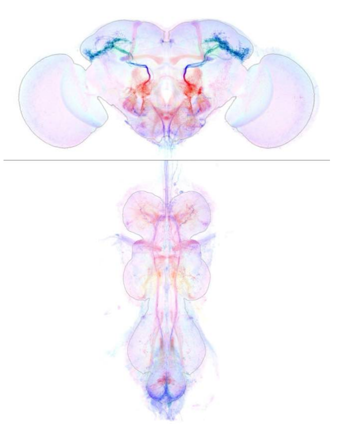
SS33915

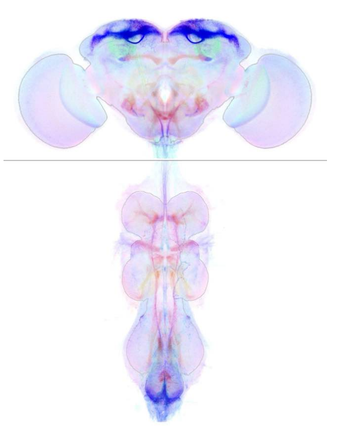
SS33917

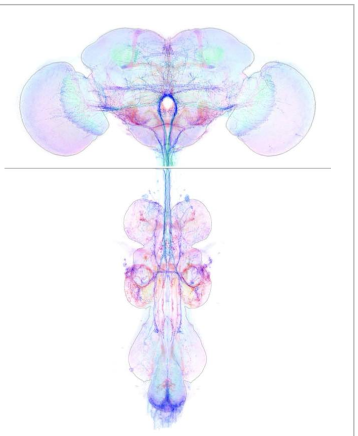
SS33919

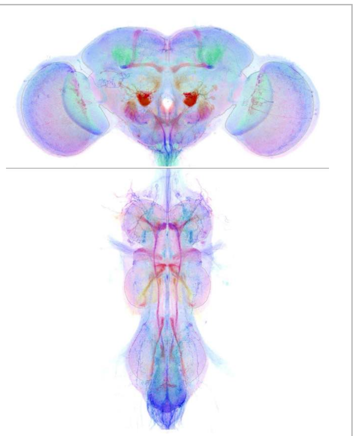
SS33920

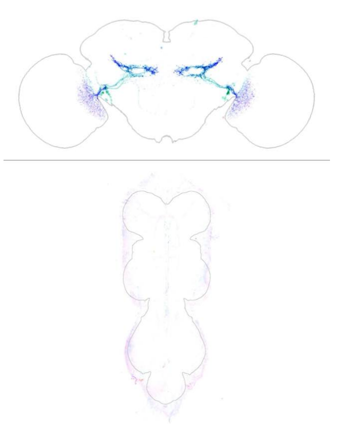
SS33949

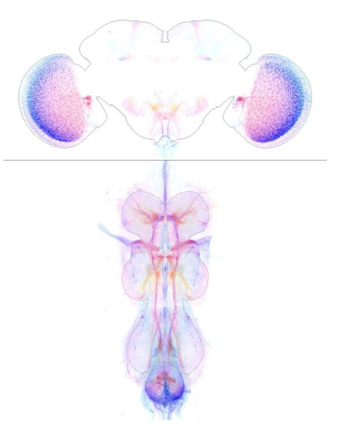
SS33980

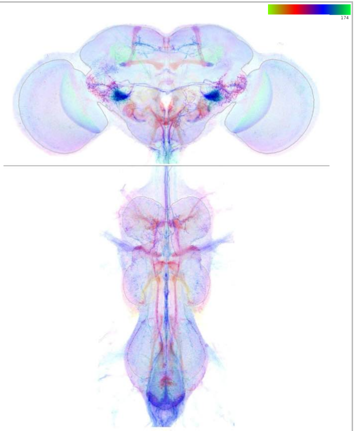
SS33983

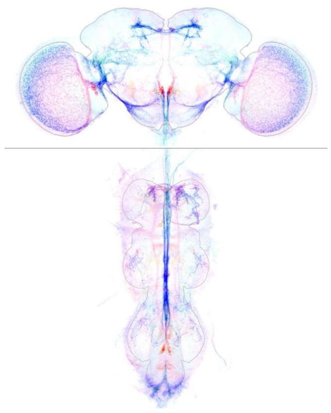
SS34037

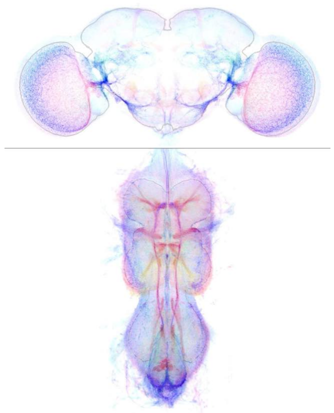
SS34042

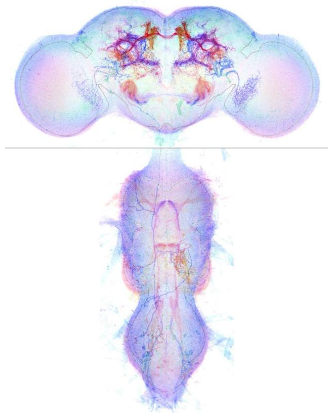
SS34154

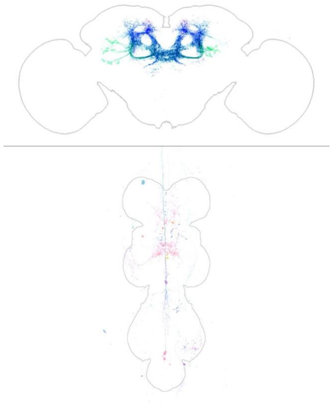
SS34167

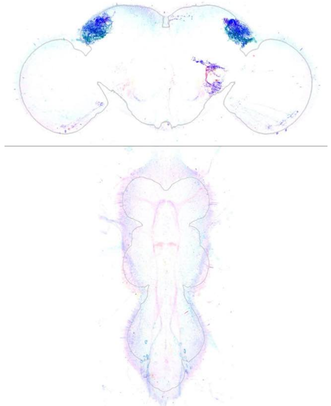
SS34190

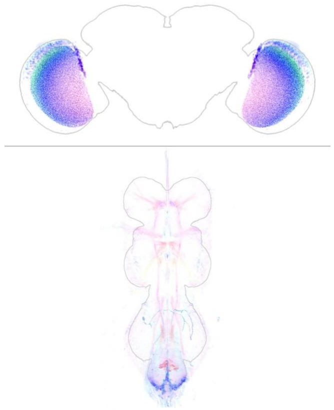
SS34201

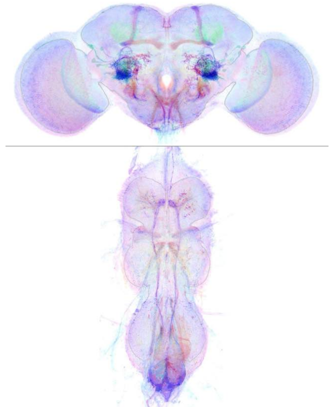
SS34215

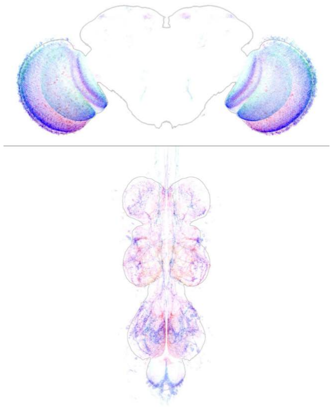
SS34266

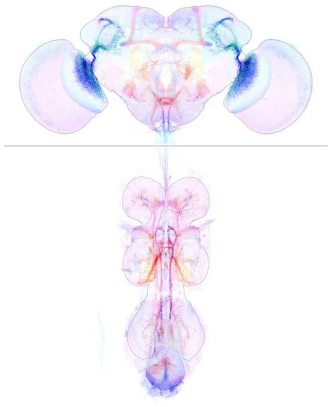
SS34276

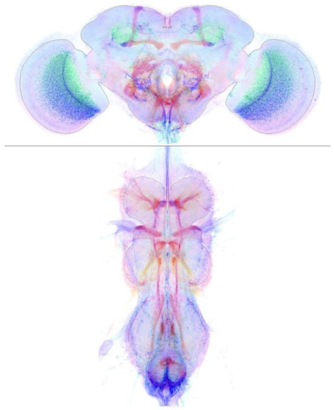
SS34318

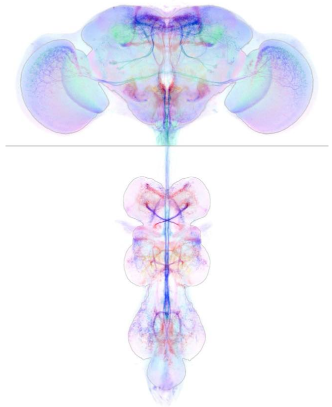
SS34478

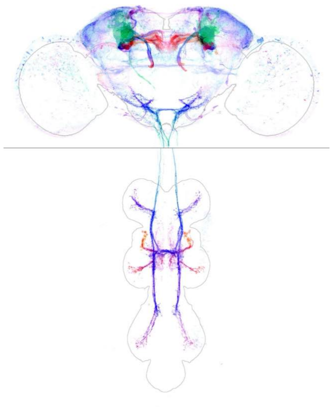
SS34537

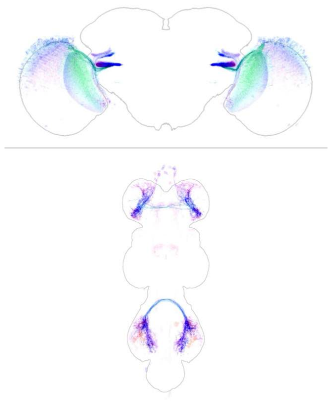
SS34547

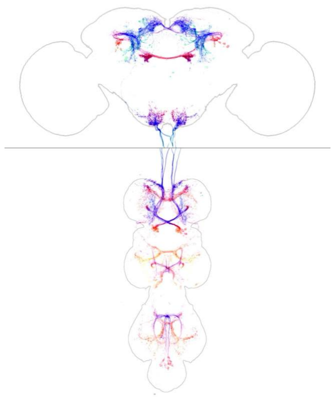
SS34574

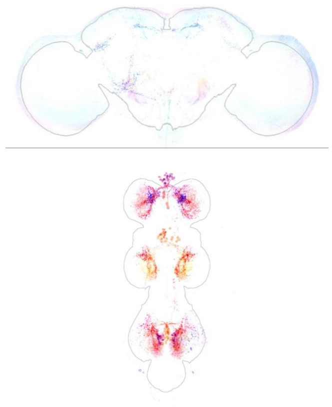
SS34614

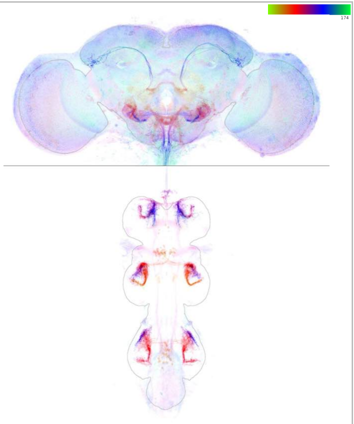
SS34621

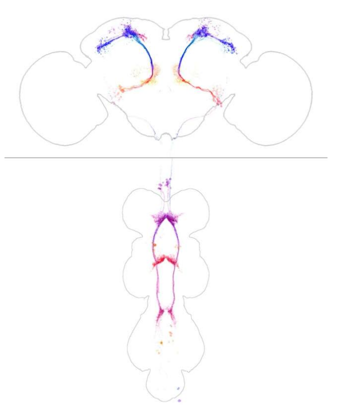
SS34663

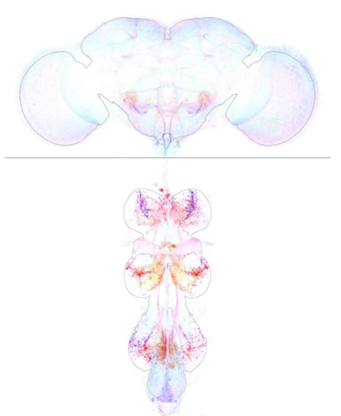
SS34668

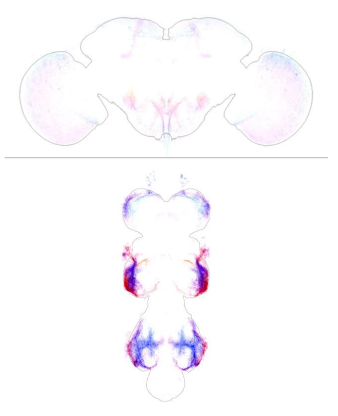
SS34687

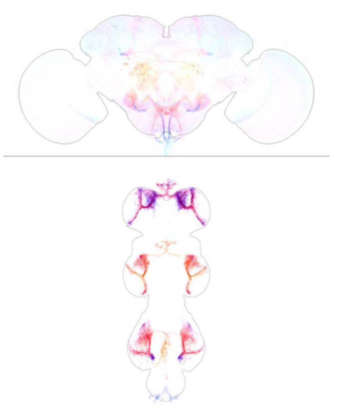
SS34692

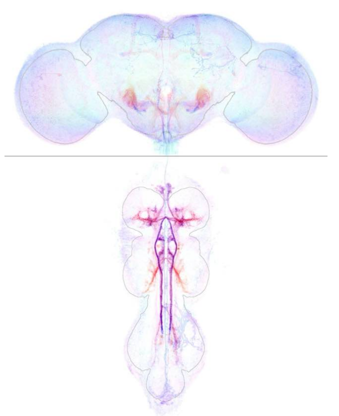
SS34701

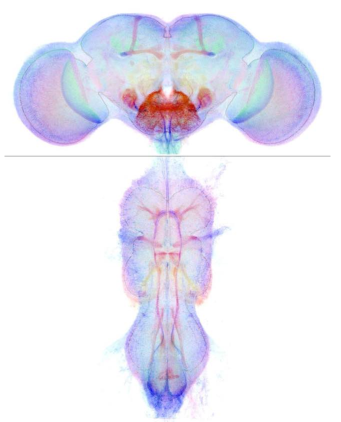
SS34732

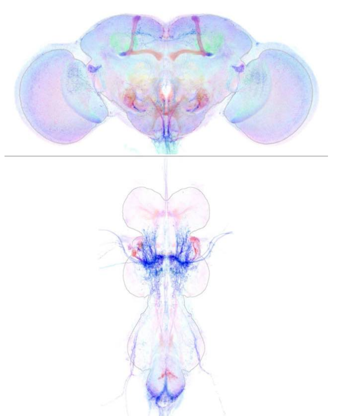
SS34781

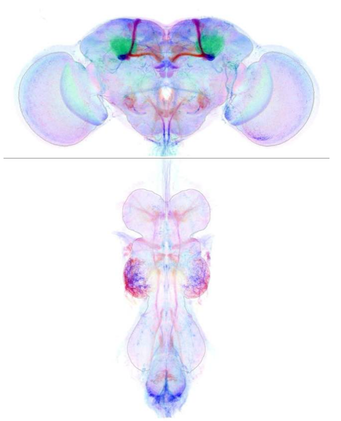
SS34789

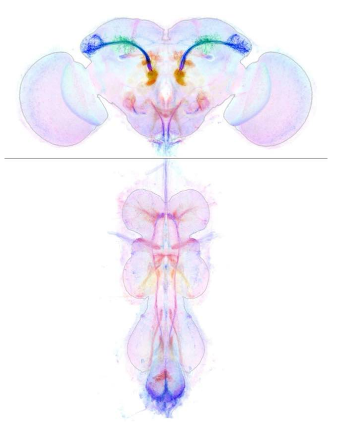
SS35043

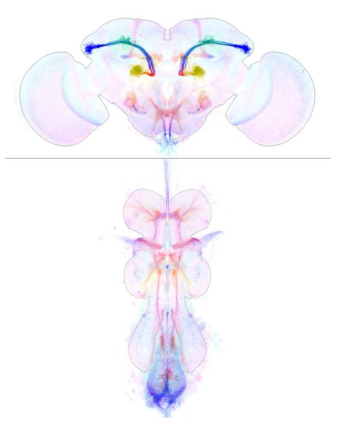
SS35094

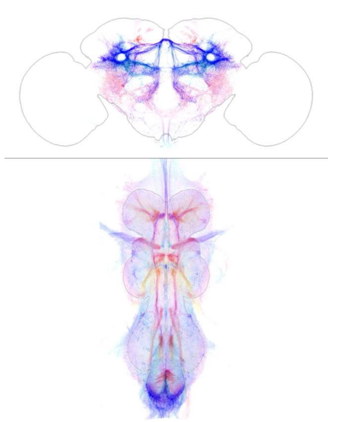
SS35138

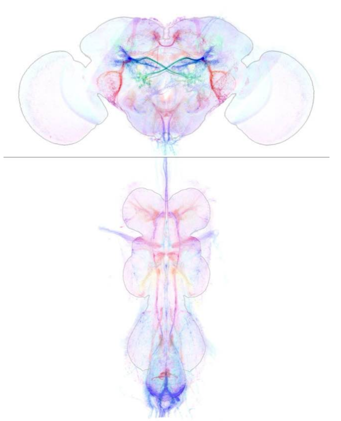
SS35196

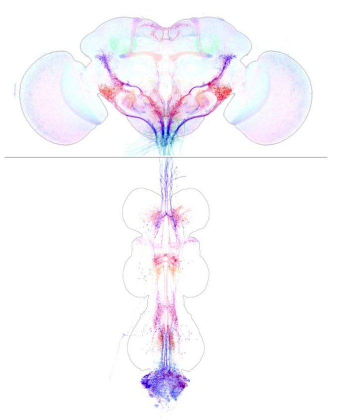
SS35224

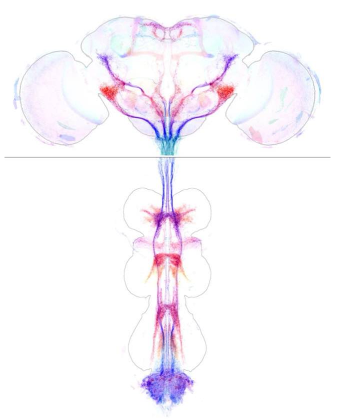
SS35225

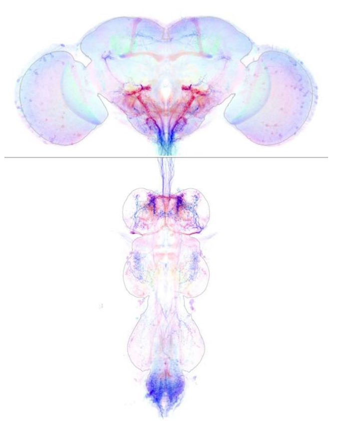
SS35227

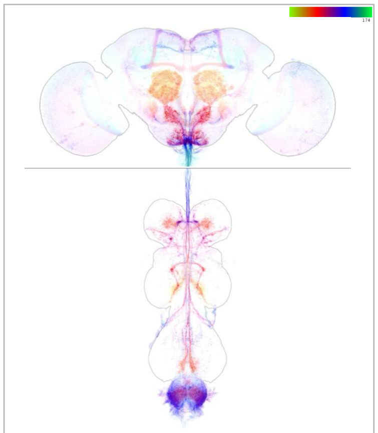

SS35230

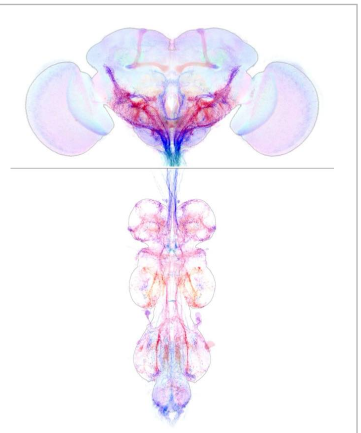

SS35234

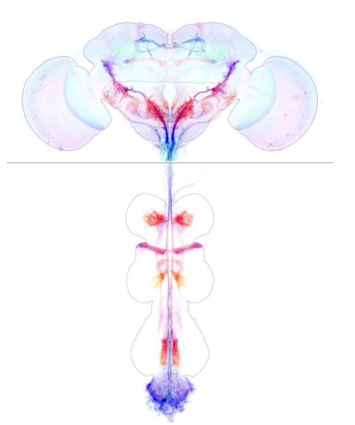

SS35236

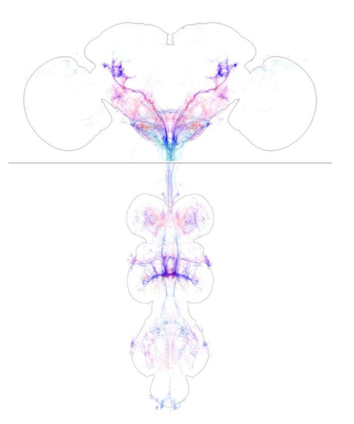

SS35241

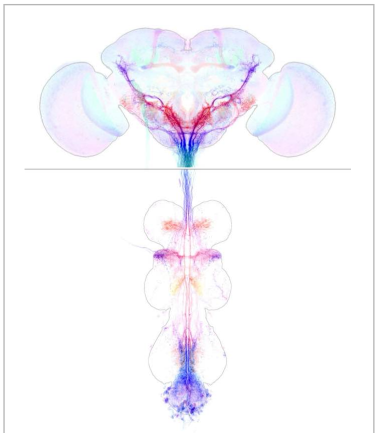

SS35248

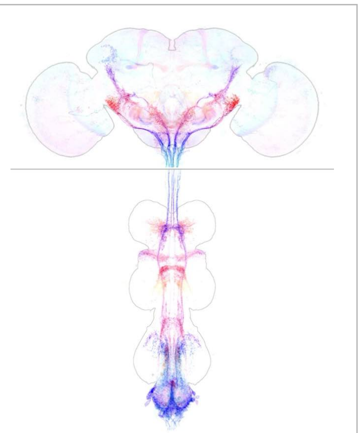

SS35253

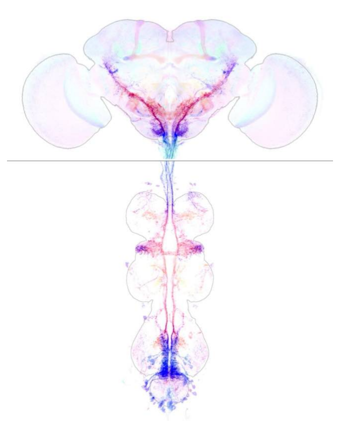

SS35256

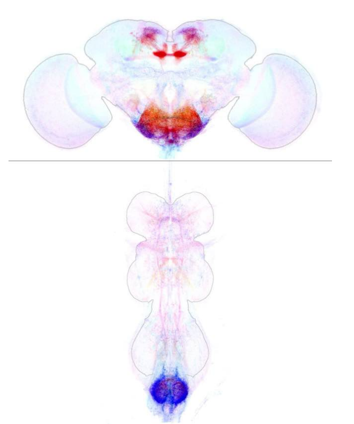

SS35259

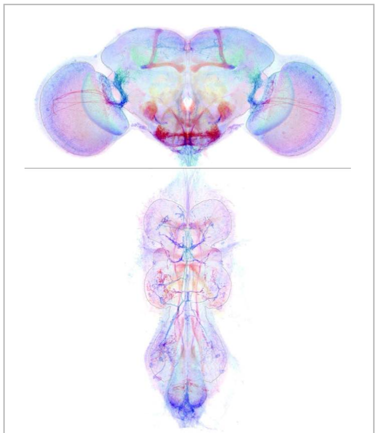

SS35279

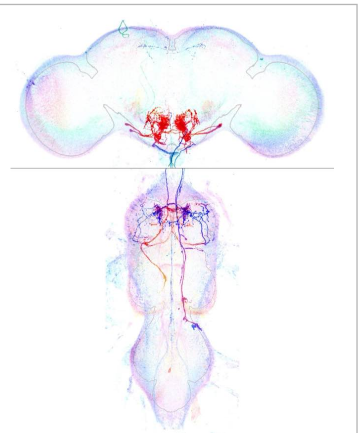

SS35291

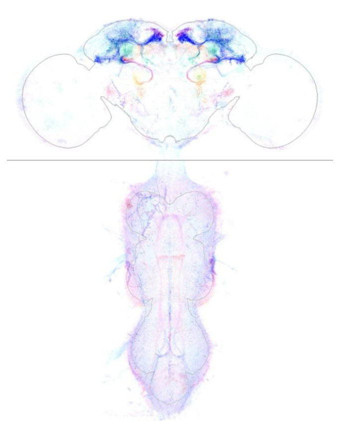

SS35317

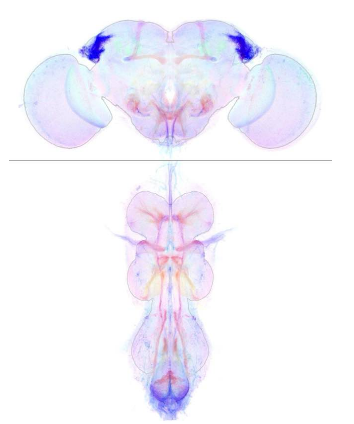

SS35339

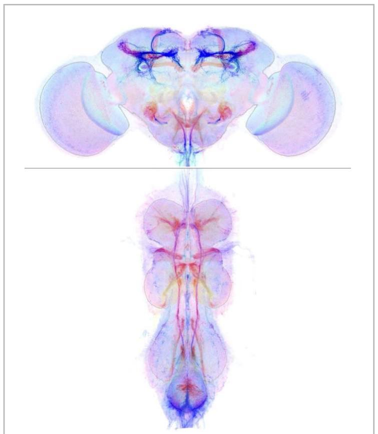

SS35352

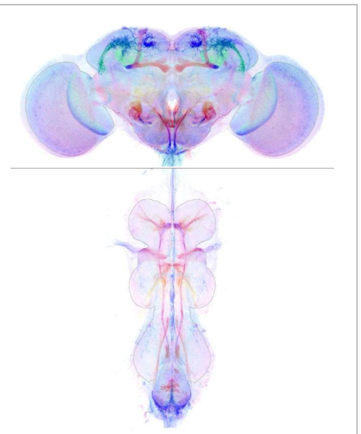

SS35366

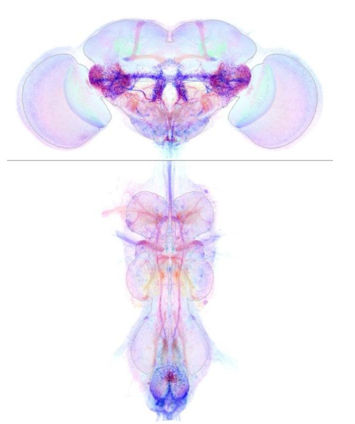

SS35420

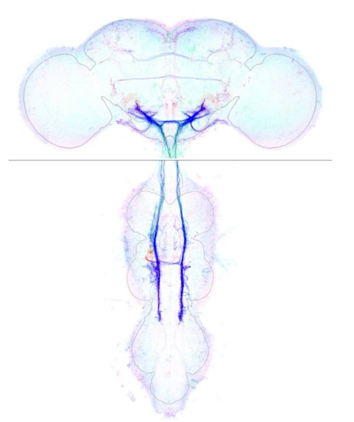

SS35442

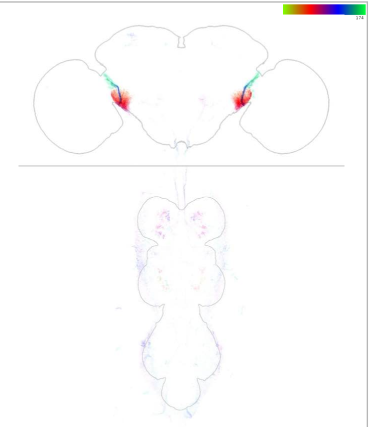
SS35456
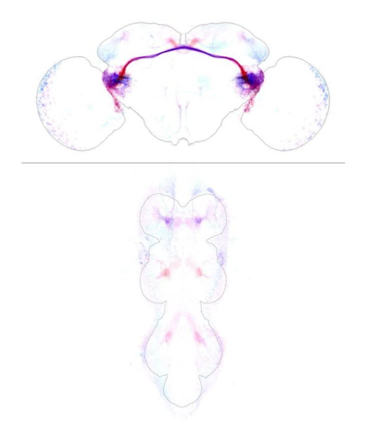
SS35471
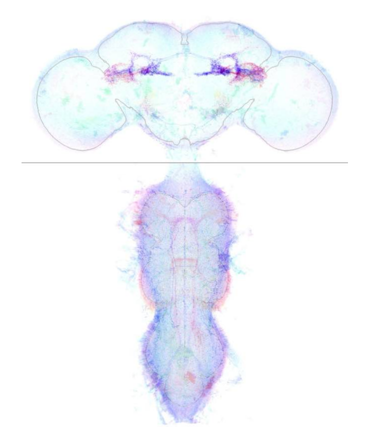
SS35484
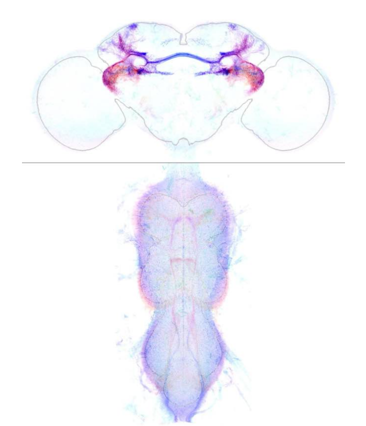
SS35502
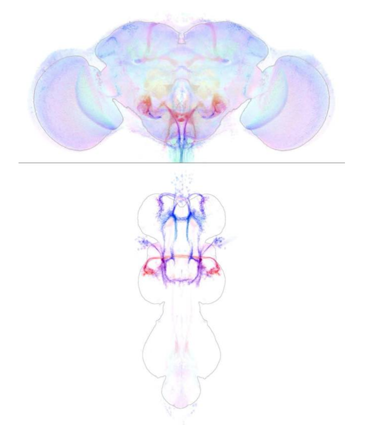
SS35560
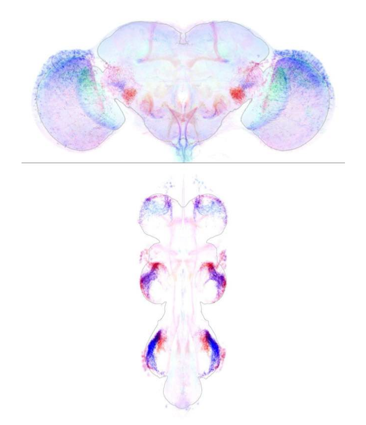
SS35605
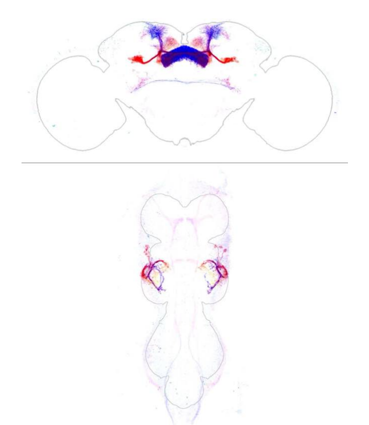
SS35622
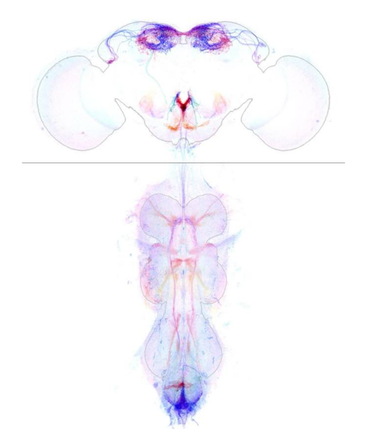
SS35640
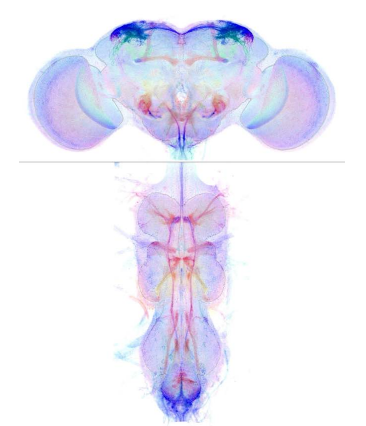
SS35659
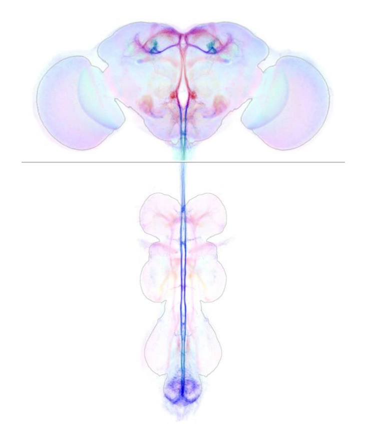
SS35666
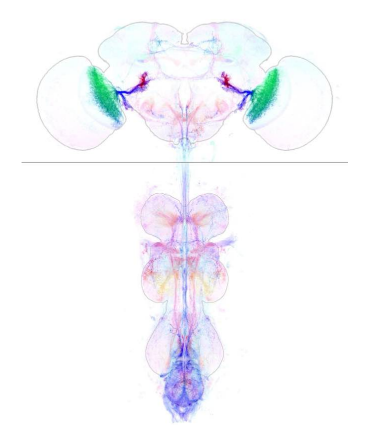
SS35672
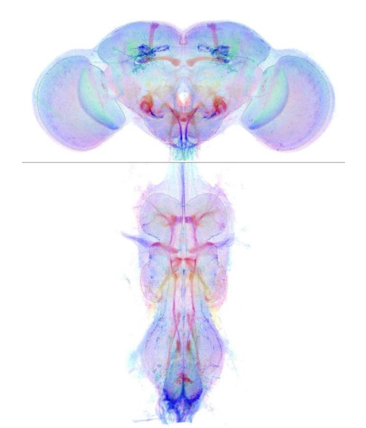
SS35674
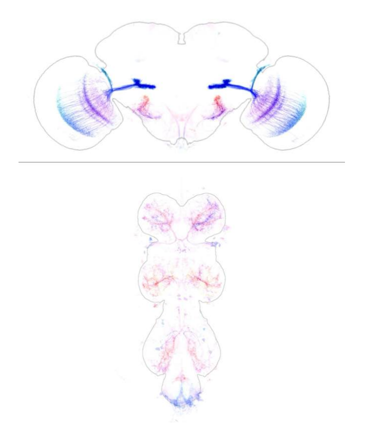
SS35750
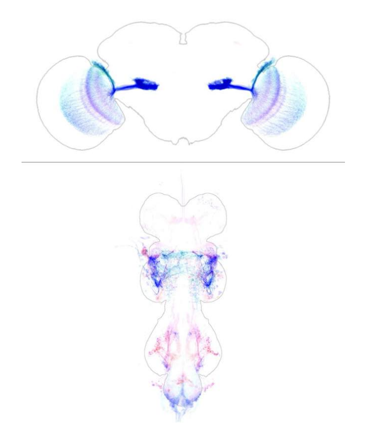
SS35783
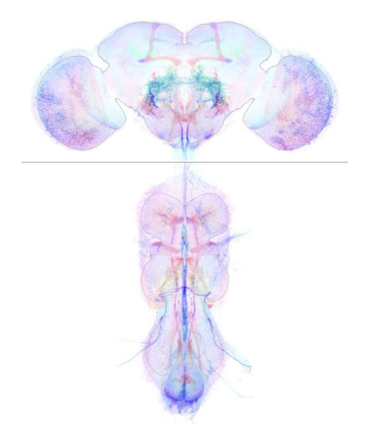
SS35837
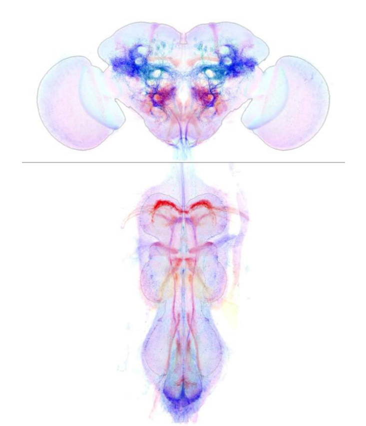
SS35873

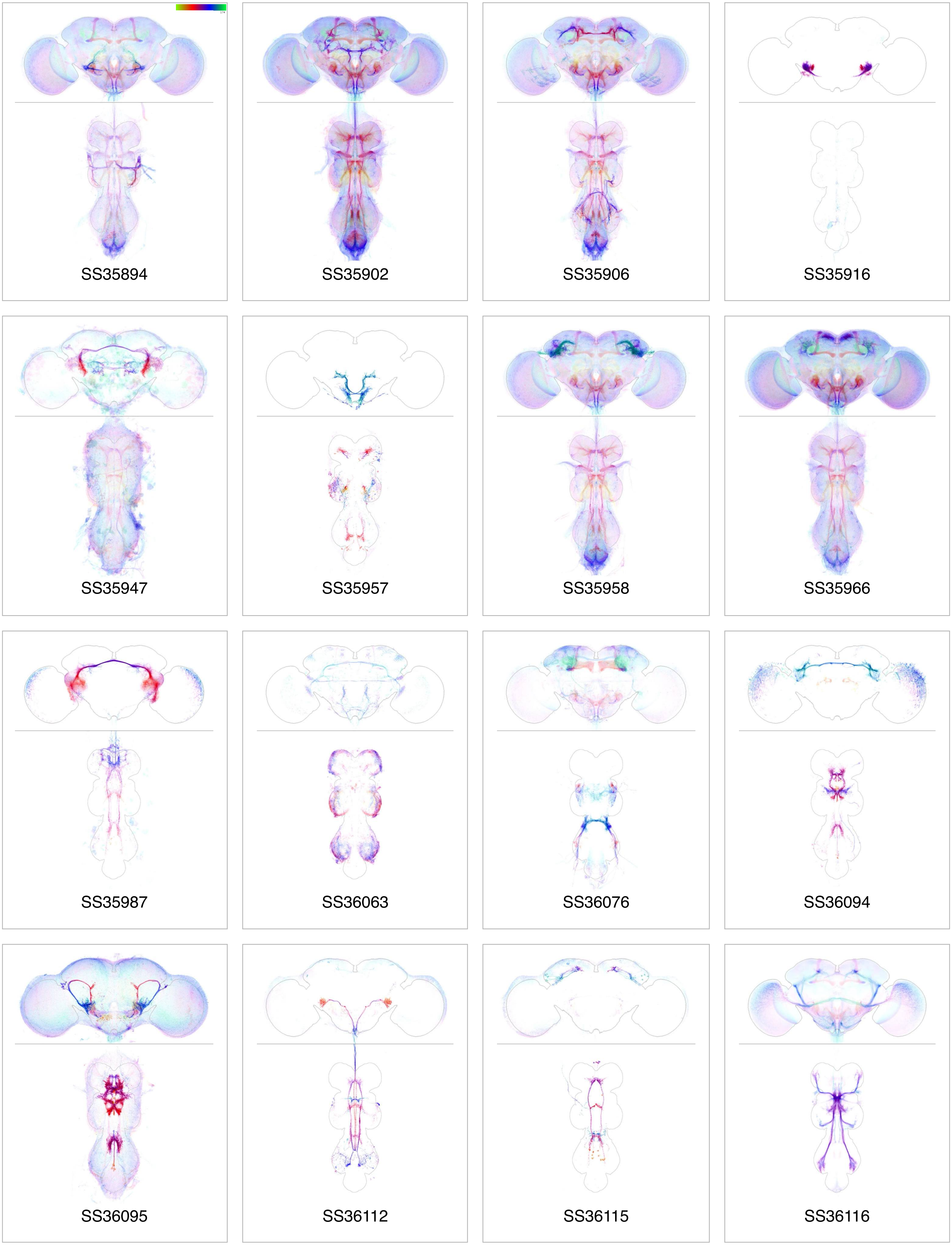
SS35894
SS35902
SS35906
SS35916
SS35947
SS35957
SS35958
SS35966
SS35987
SS36063
SS36076
SS36094
SS36095
SS36112
SS36115
SS36116

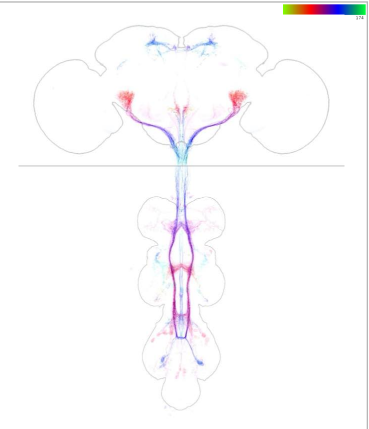

SS36118

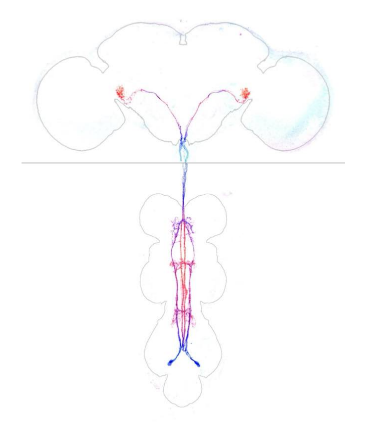

SS36131

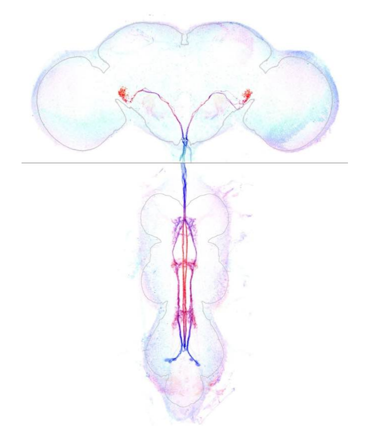

SS36132

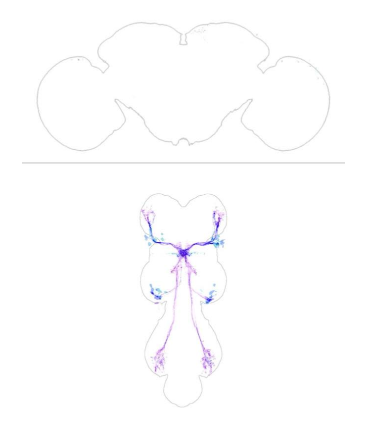

SS36134

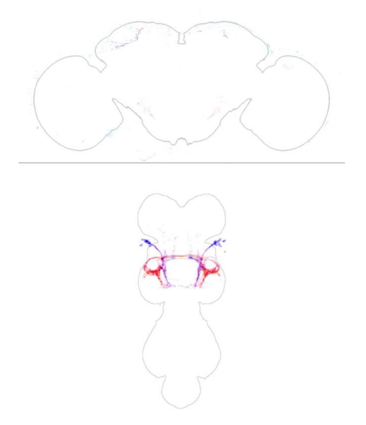

SS36152

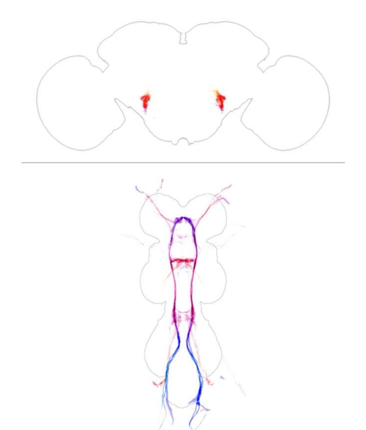

SS36162

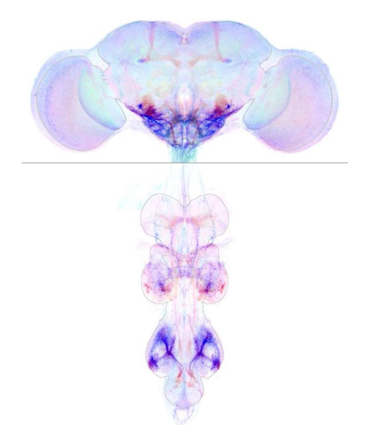

SS36194

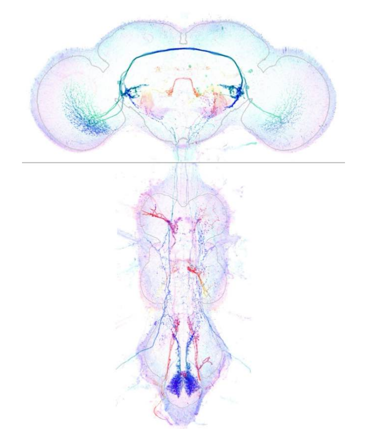

SS36332

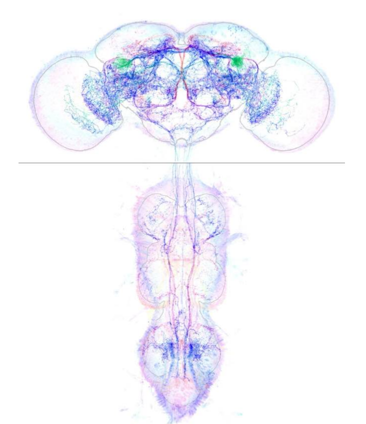

SS36363

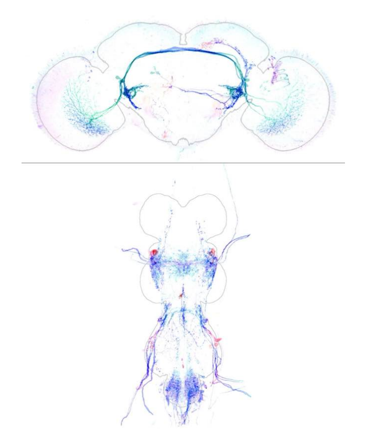

SS36423

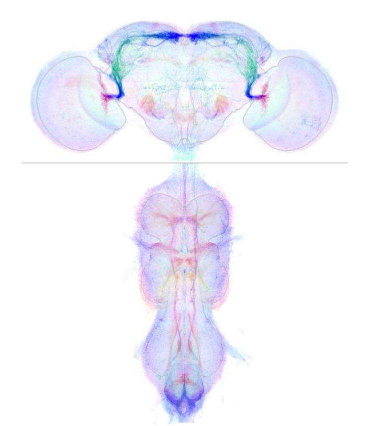

SS36428

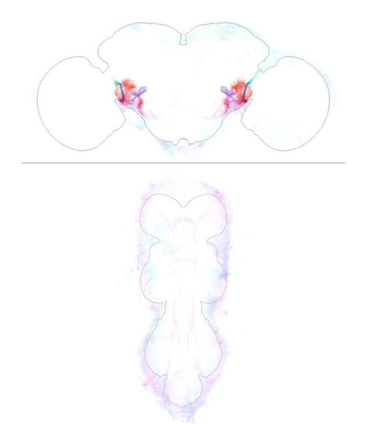

SS36542

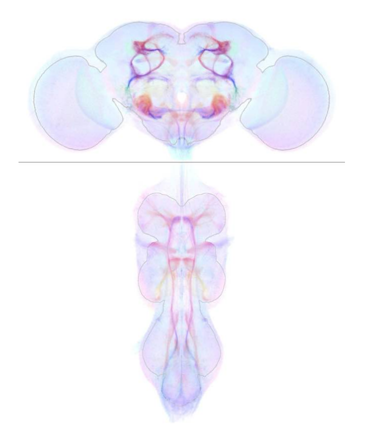

SS36551

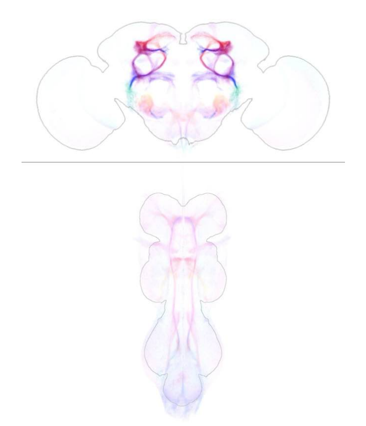

SS36564

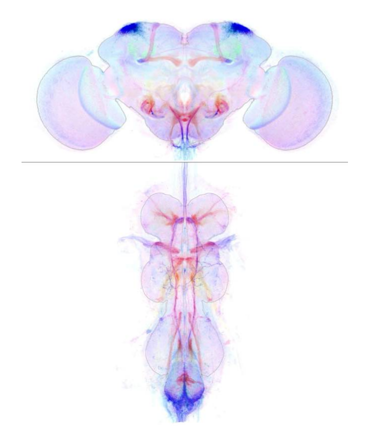

SS36583

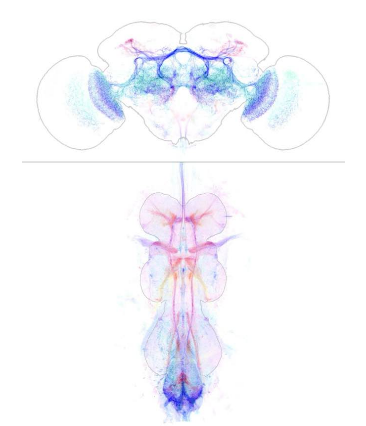

SS36585

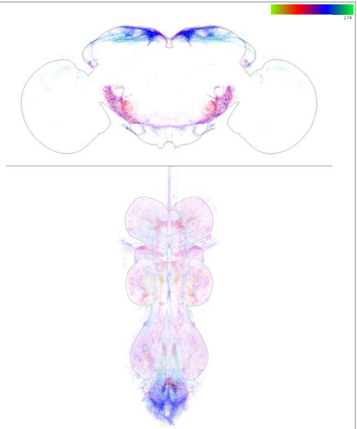
SS36676

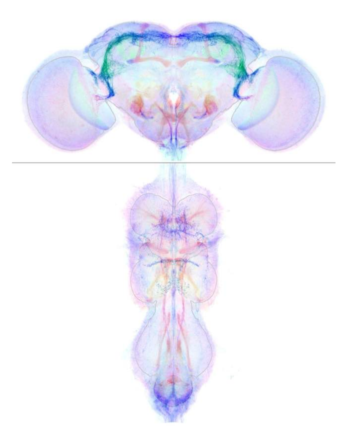
SS36699

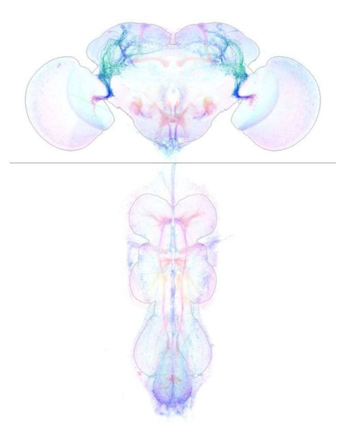
SS36821

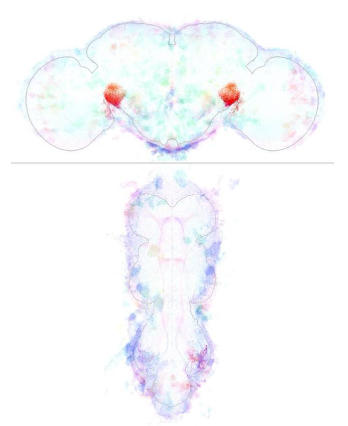
SS36948

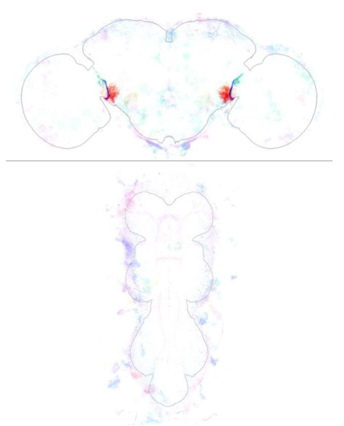
SS36959

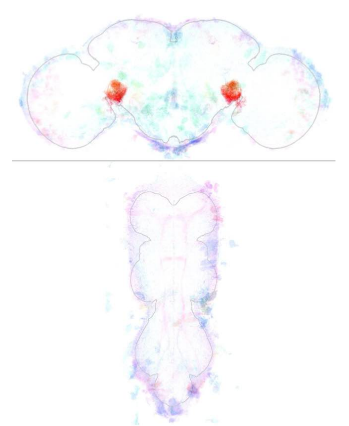
SS36960

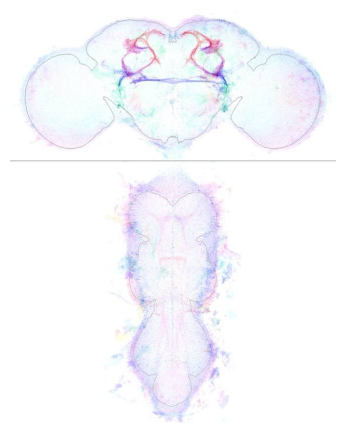
SS36971

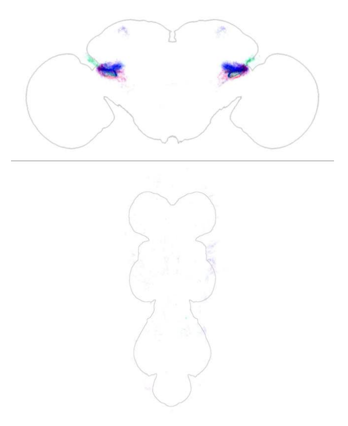
SS36986

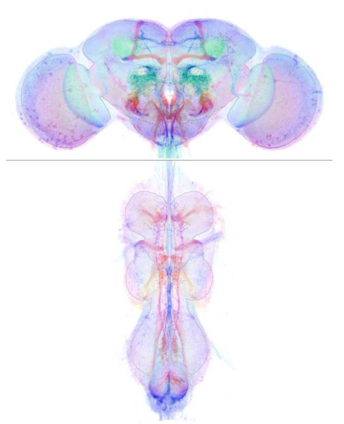
SS37026

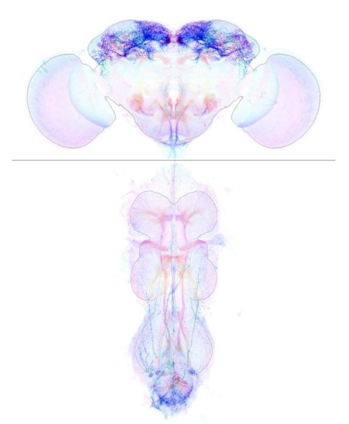
SS37049

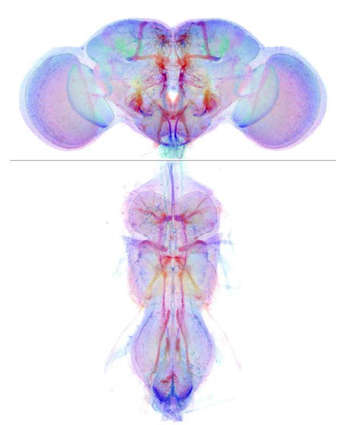
SS37052

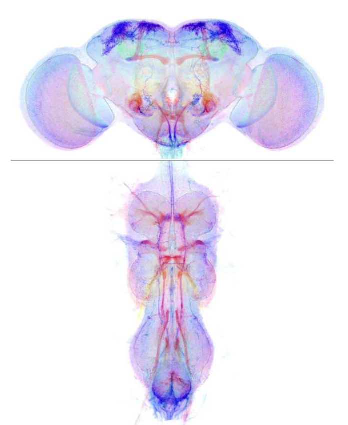
SS37057

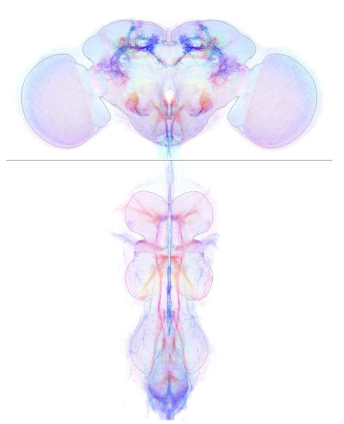
SS37072

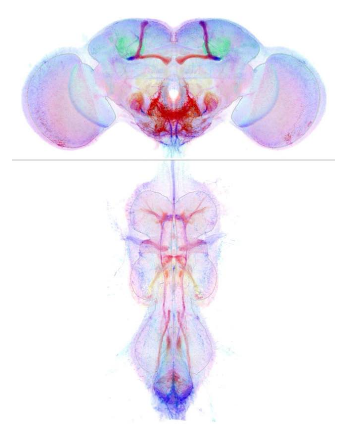
SS37135

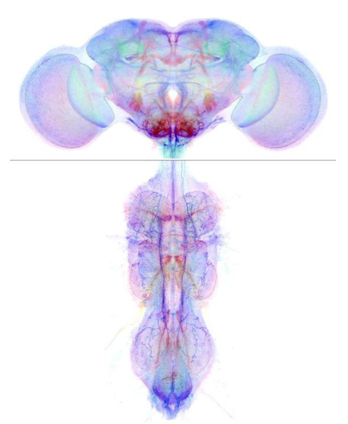
SS37153

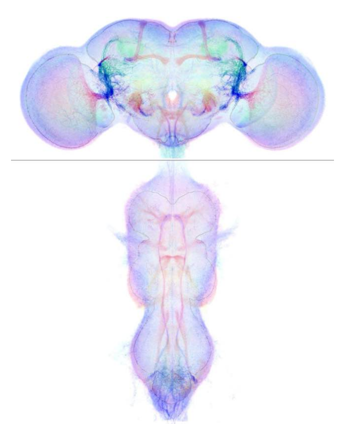
SS37203

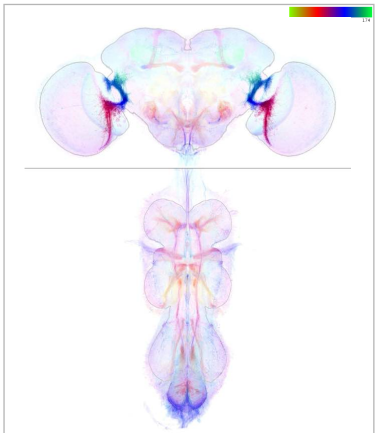
SS37207

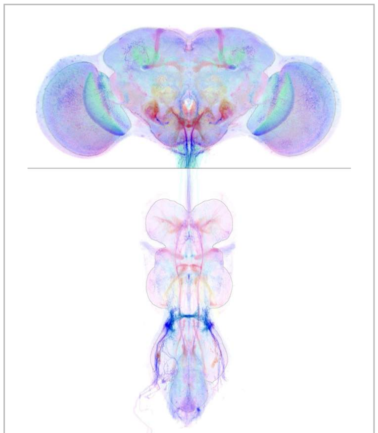
SS37231

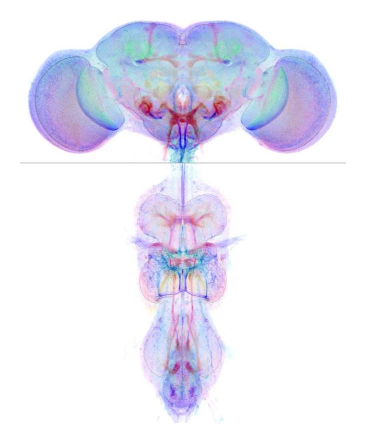
SS37233

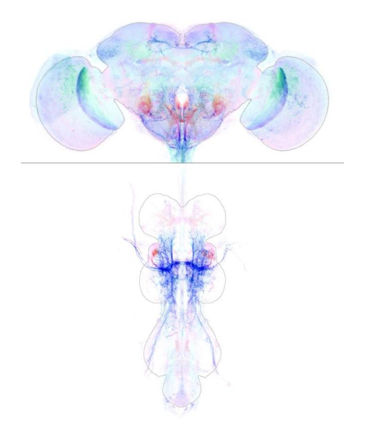
SS37246

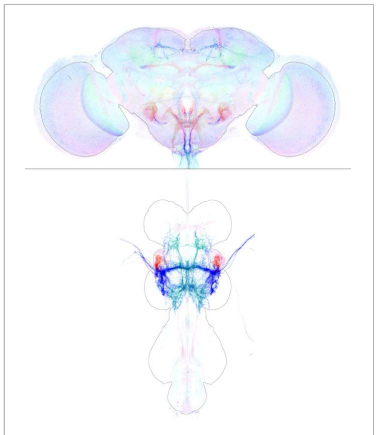
SS37252

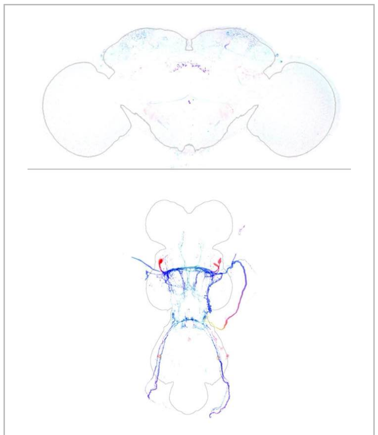
SS37253

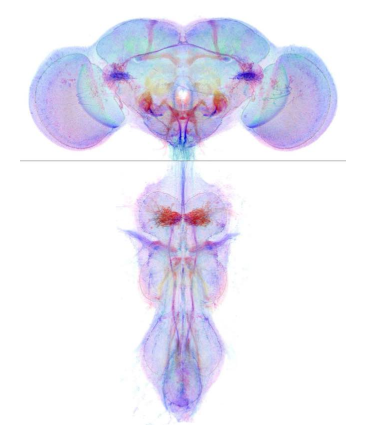
SS37262

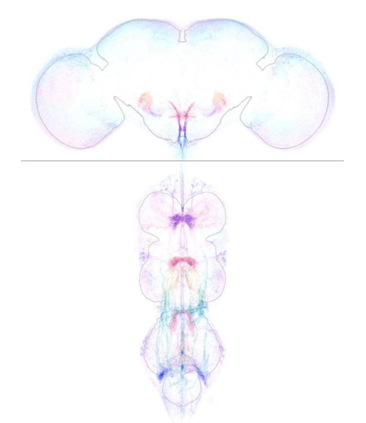
SS37296

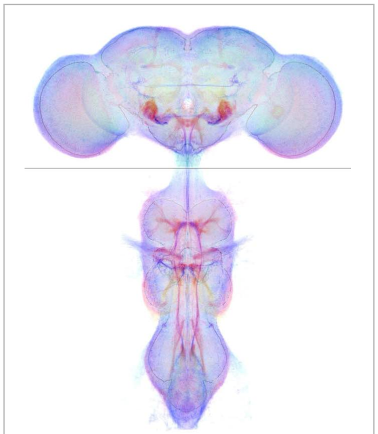
SS37306

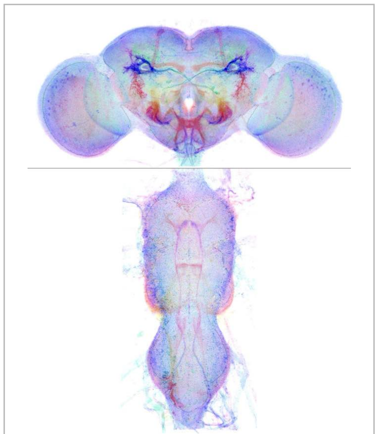
SS37419

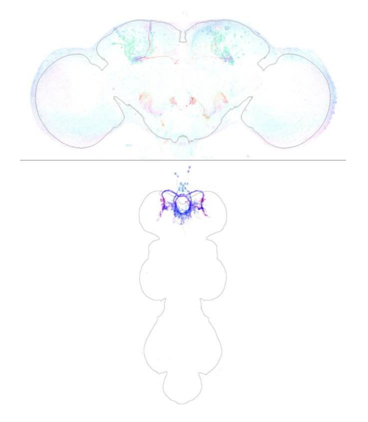
SS37632

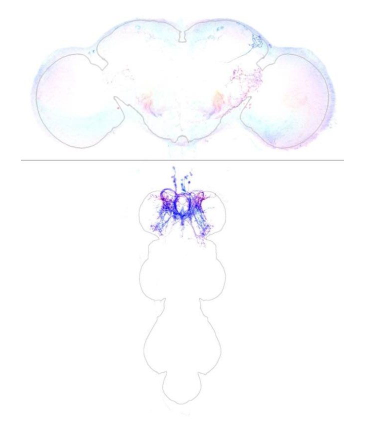
SS37633

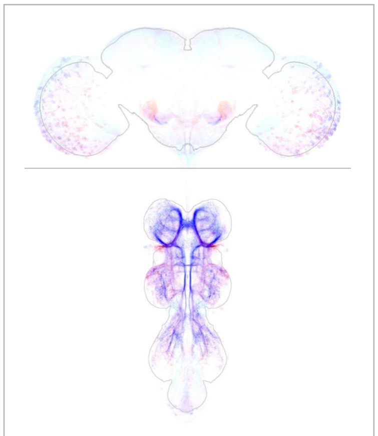
SS37641

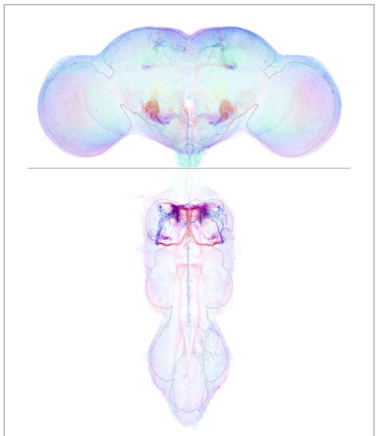
SS37648

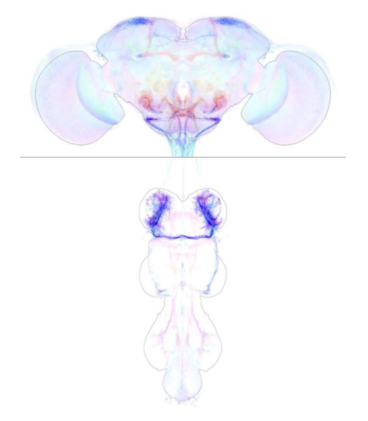
SS37652

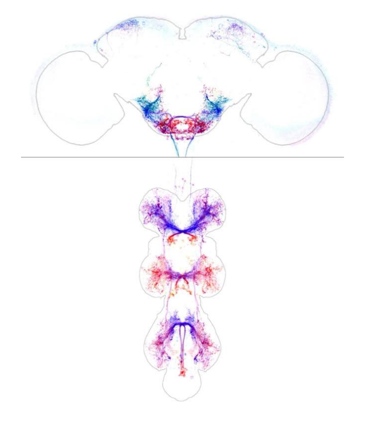
SS37659

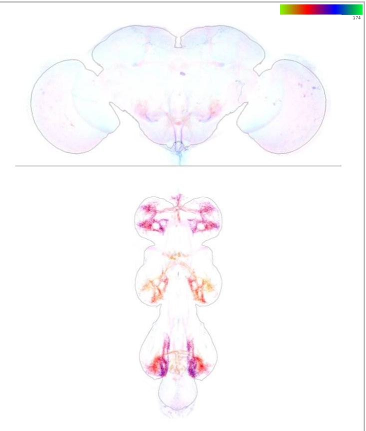
SS37671

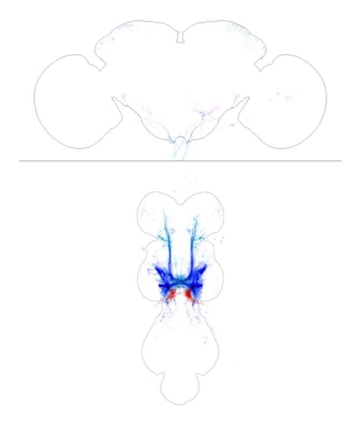
SS37685

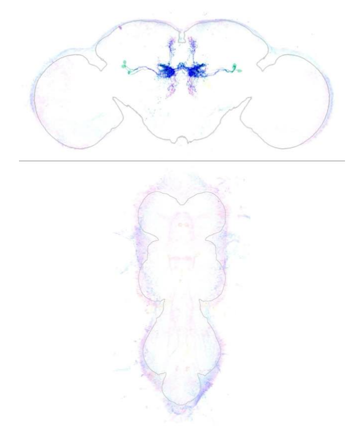
SS37714

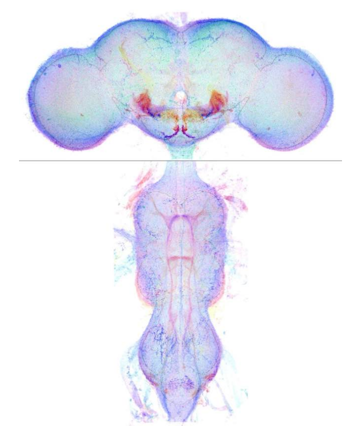
SS37773

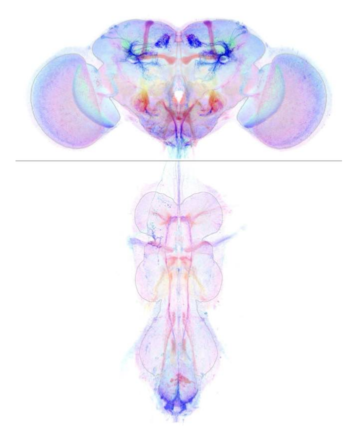
SS37873

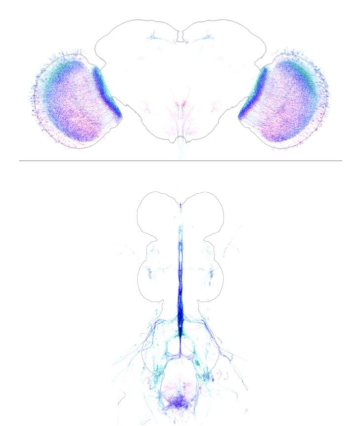
SS37928

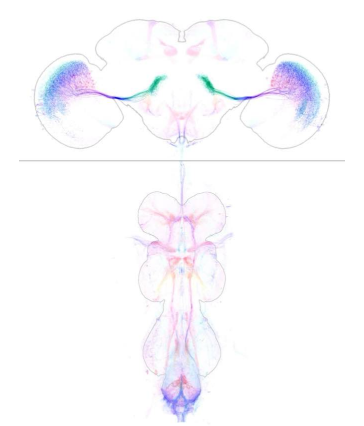
SS37958

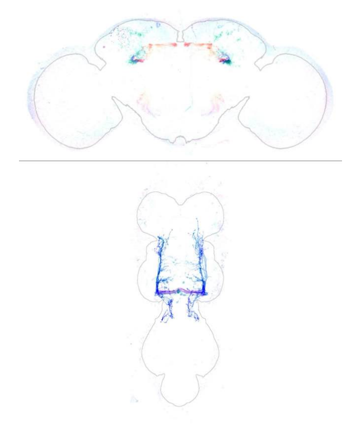
SS37984

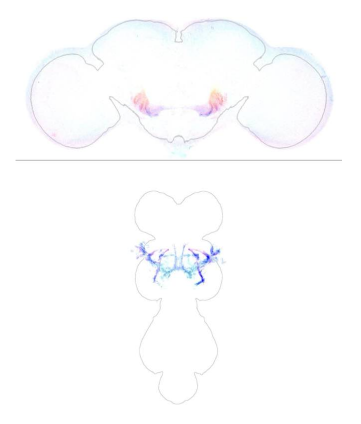
SS38007

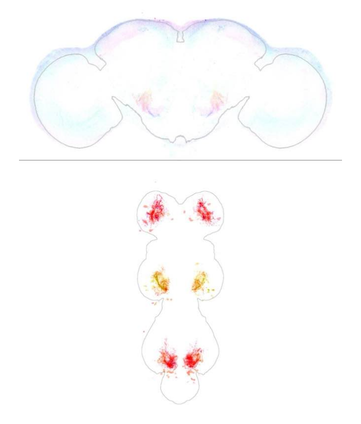
SS38013

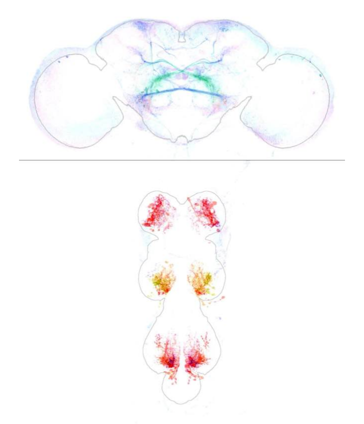
SS38021

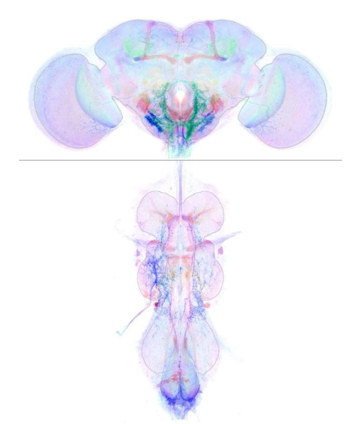
SS38113

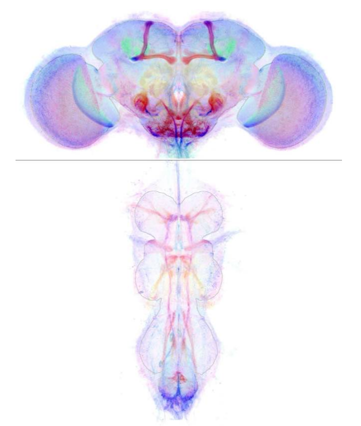
SS38140

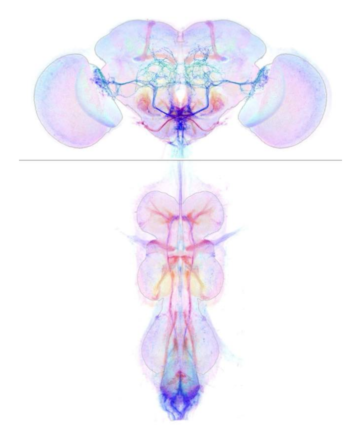
SS38165

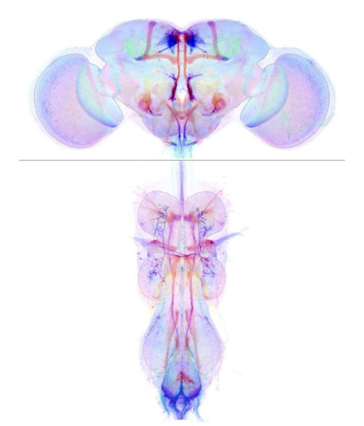
SS38176

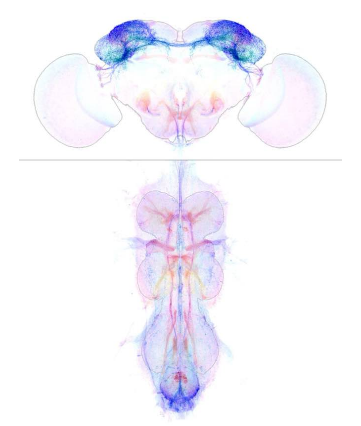
SS38189

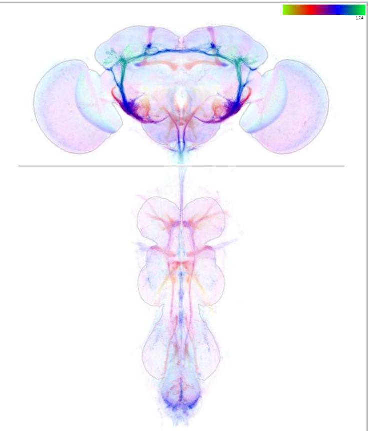
SS38214
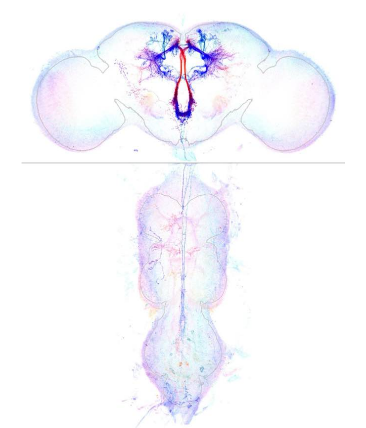
SS38222
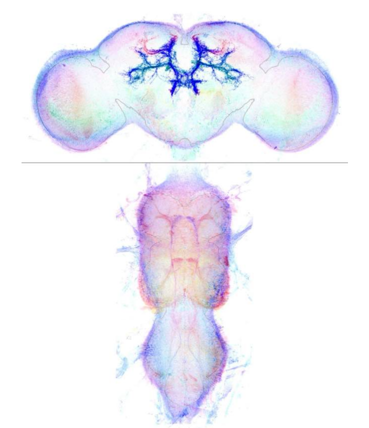
SS38223
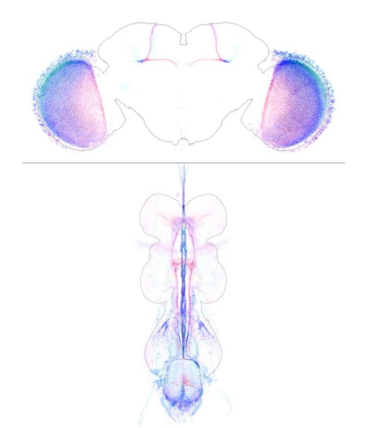
SS38287
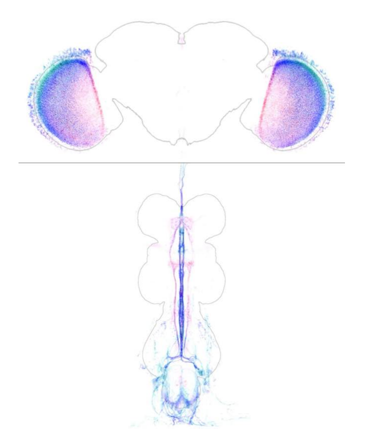
SS38292
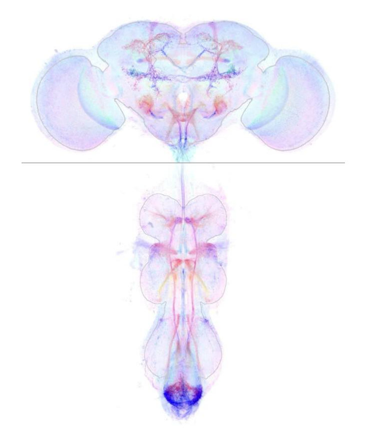
SS38360
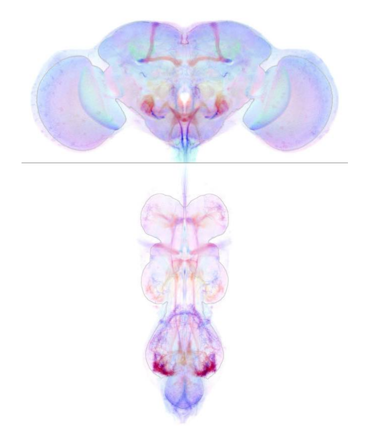
SS38361
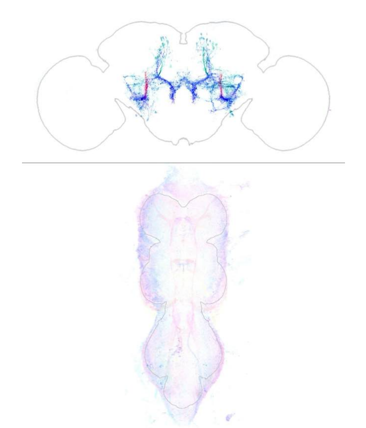
SS38514
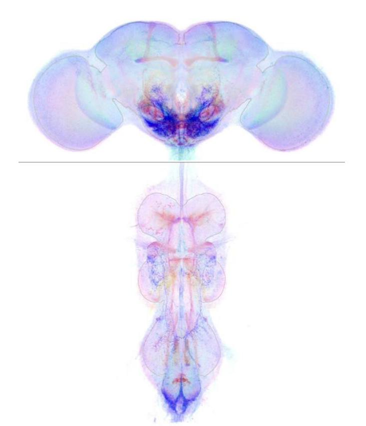
SS38523
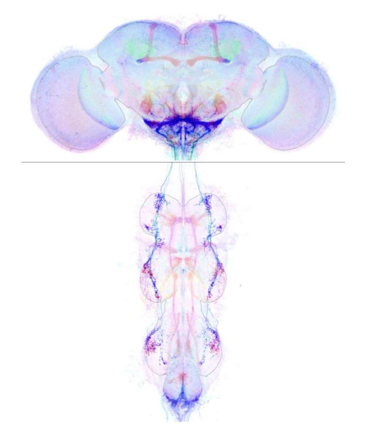
SS38535
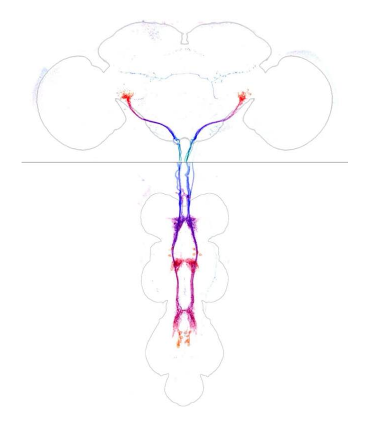
SS38592
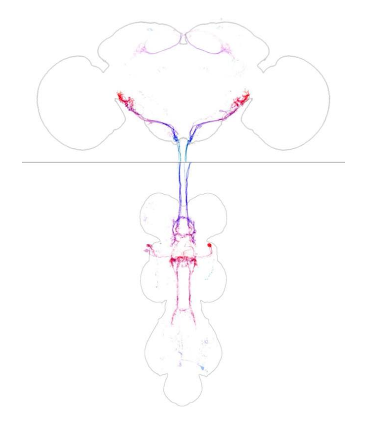
SS38624
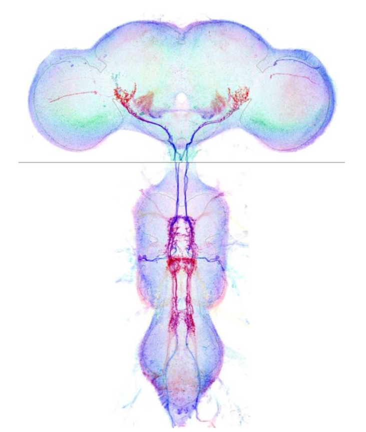
SS38631
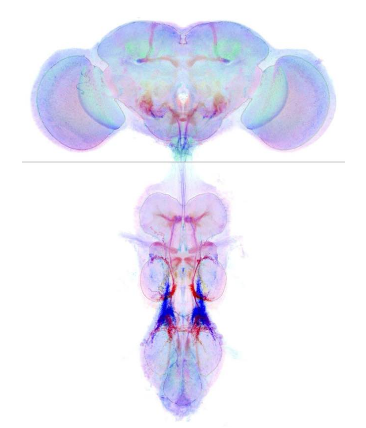
SS38644
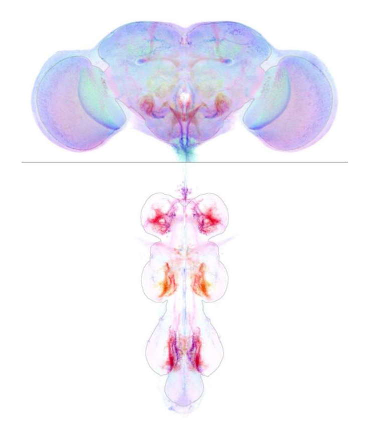
SS38723
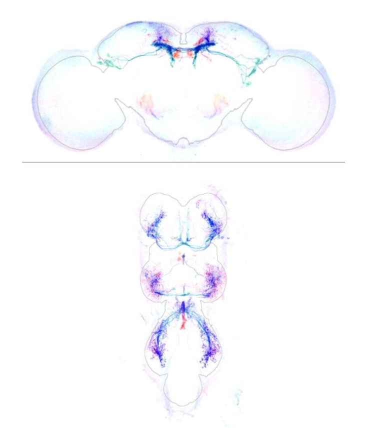
SS38727

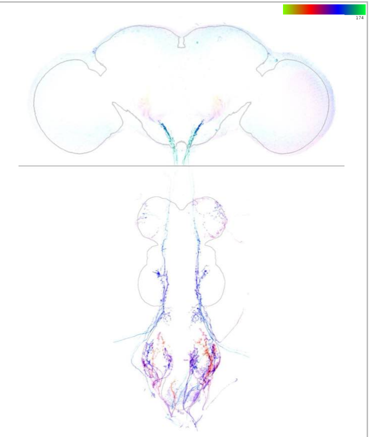
SS38746

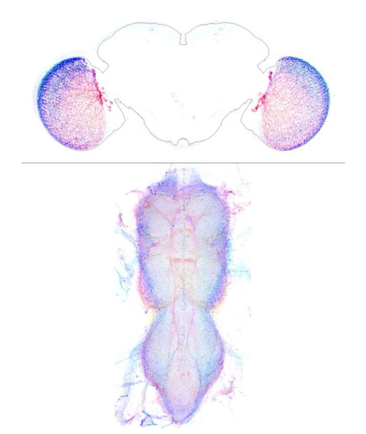
SS38770

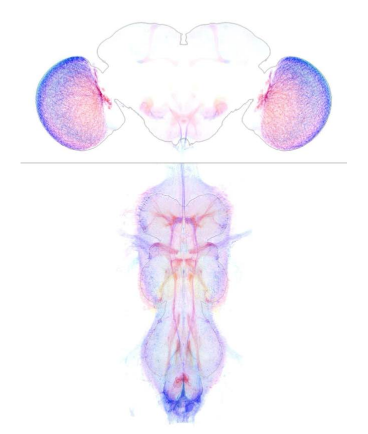
SS38773

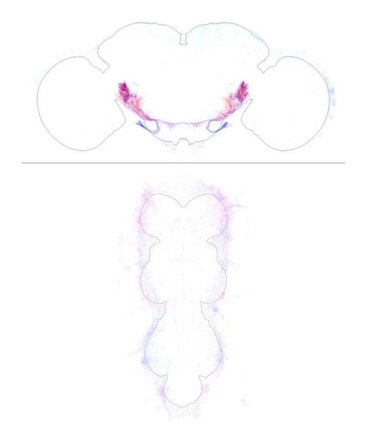
SS38918

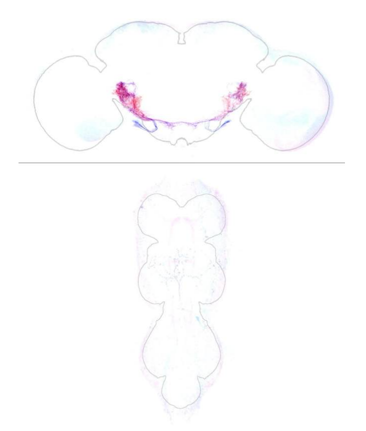
SS38949

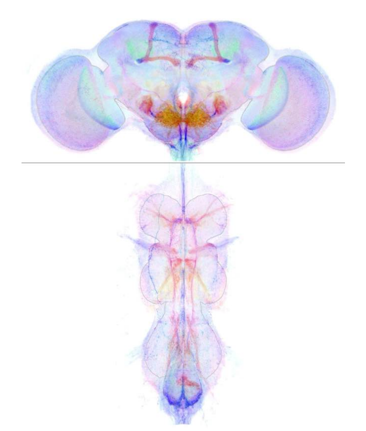
SS39007

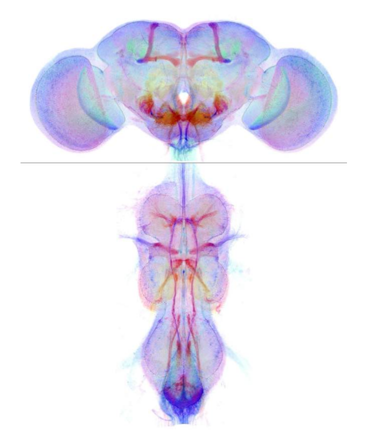
SS39019

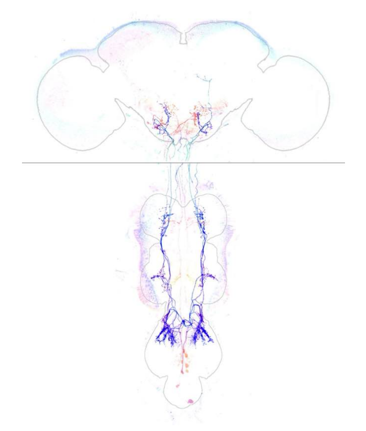
SS39036

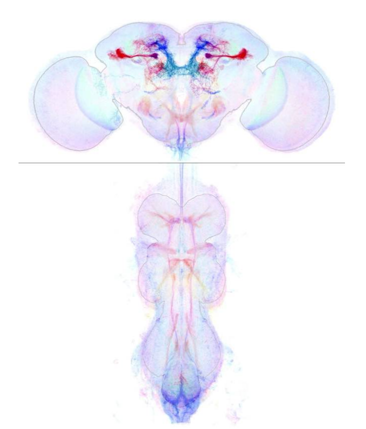
SS39089

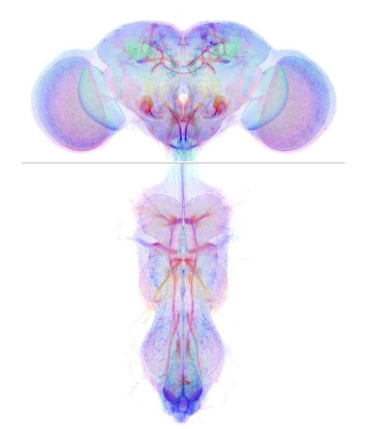
SS39106

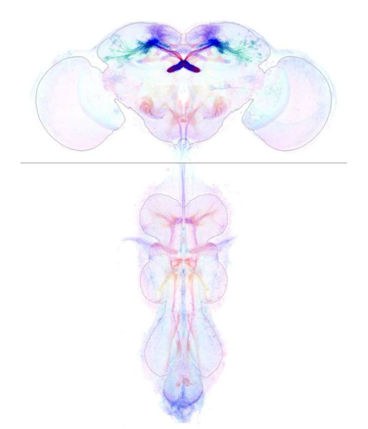
SS39256

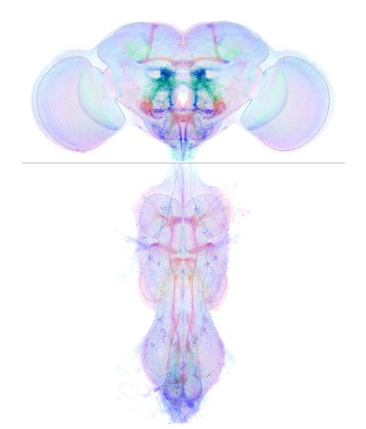
SS39271

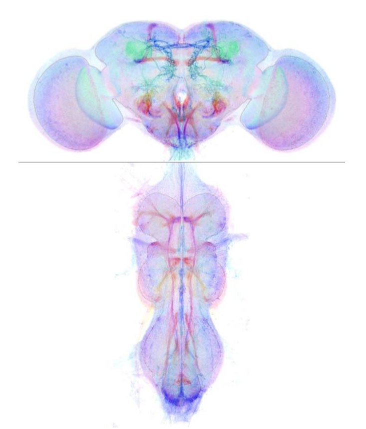
SS39274

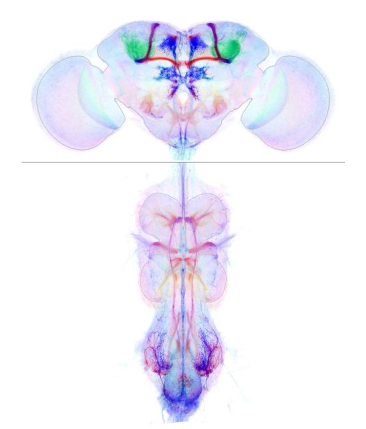
SS39299

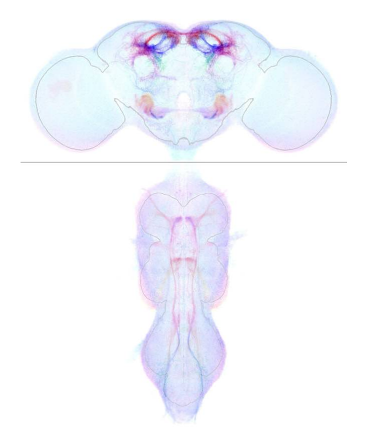
SS39313

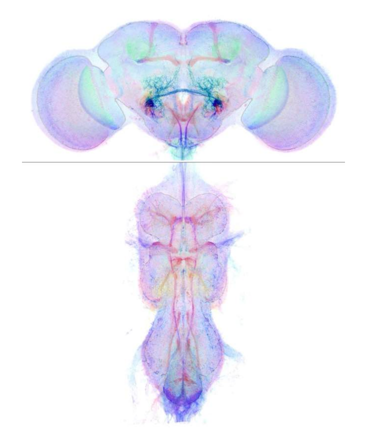
SS39319

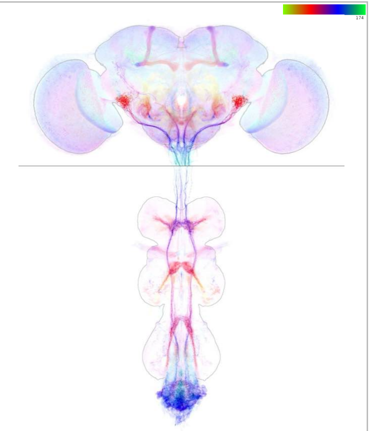

SS39322

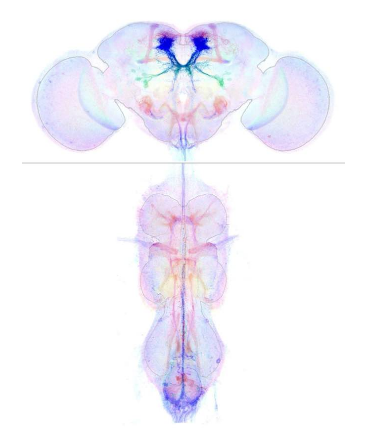

SS39553

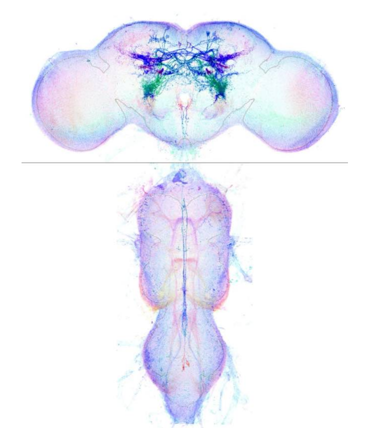

SS39581

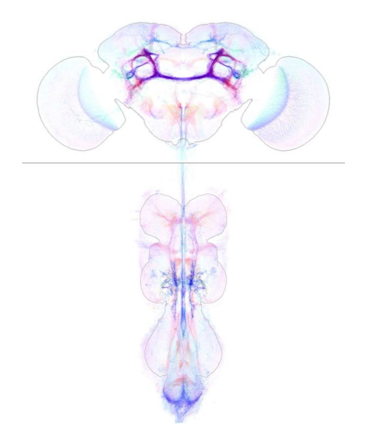

SS39617

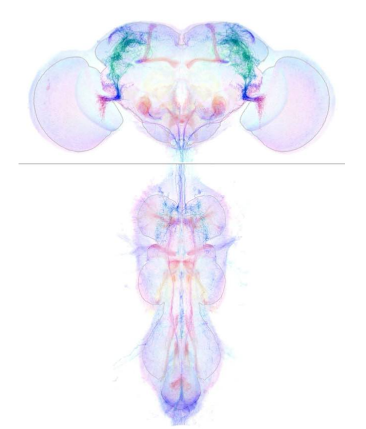

SS39662

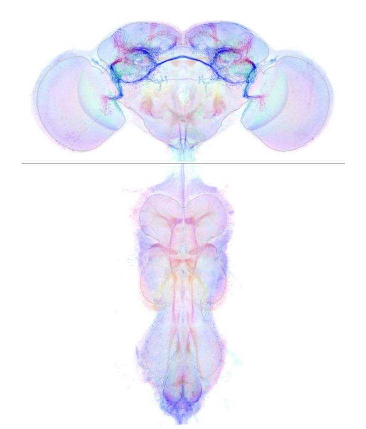

SS39673

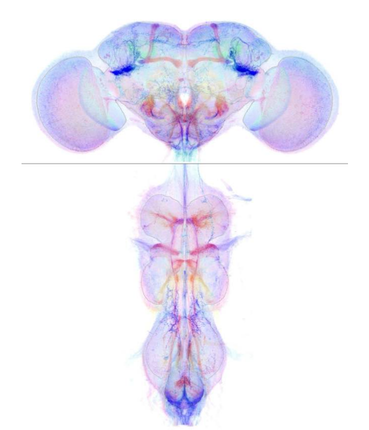

SS39736

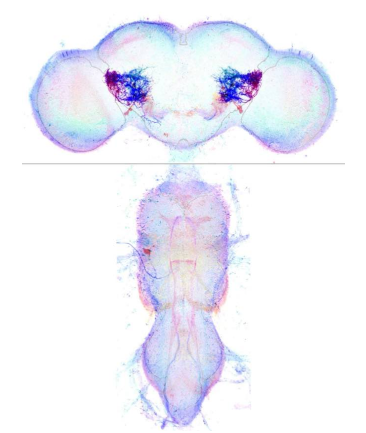

SS39749

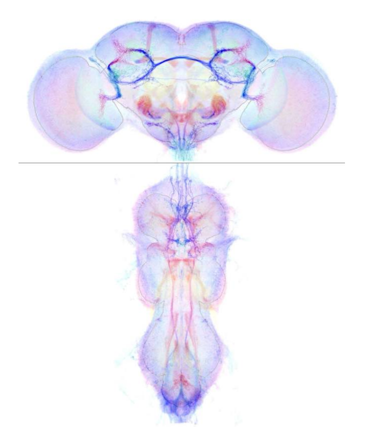

SS39782

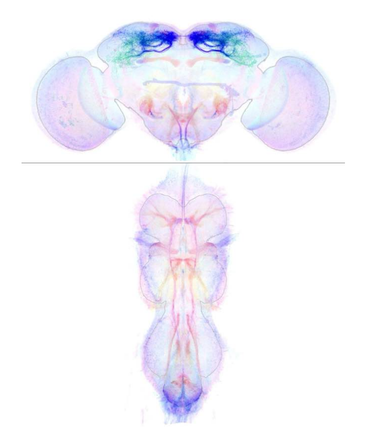

SS39797

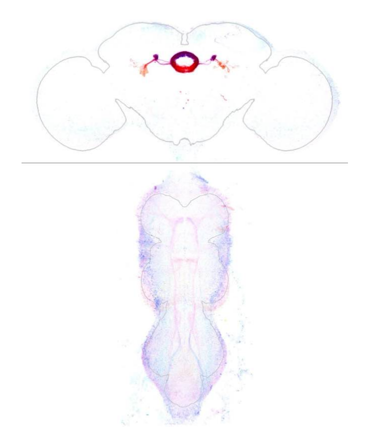

SS39836

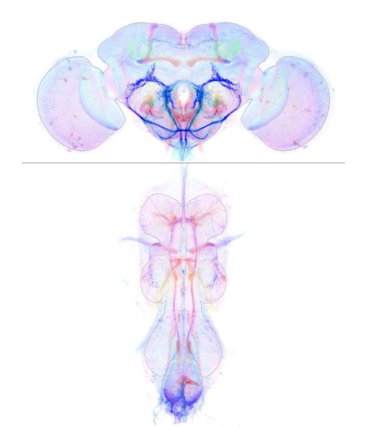

SS39883

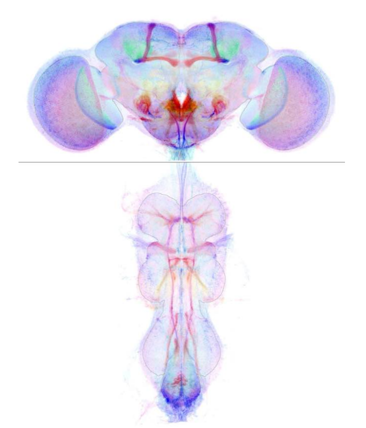

SS39890

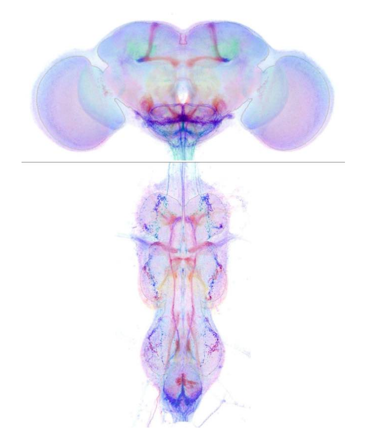

SS39916

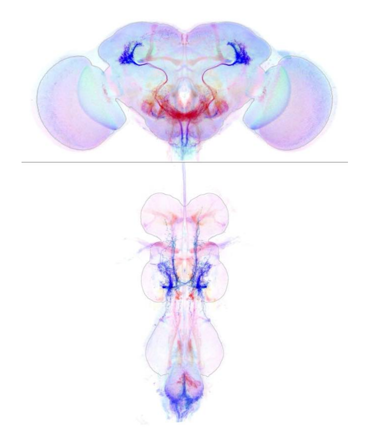

SS39993

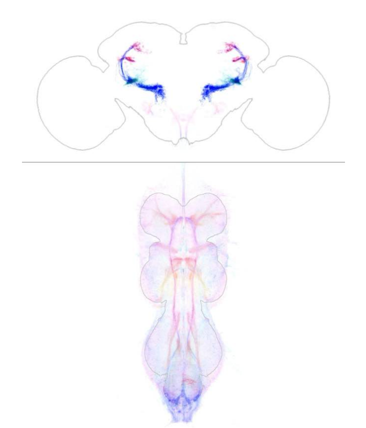

SS40006

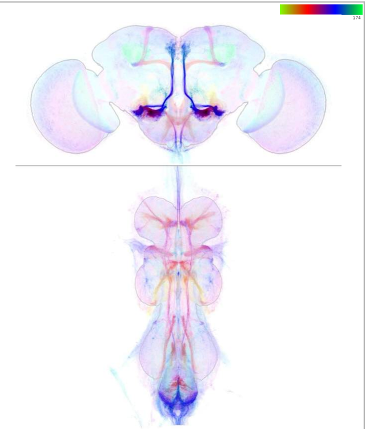
SS40016

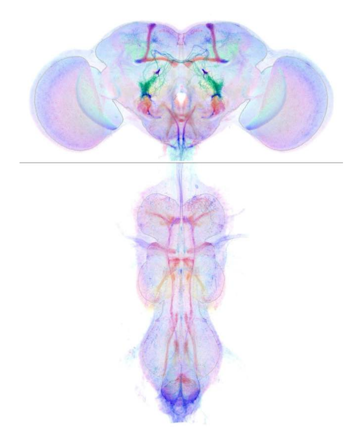
SS40058

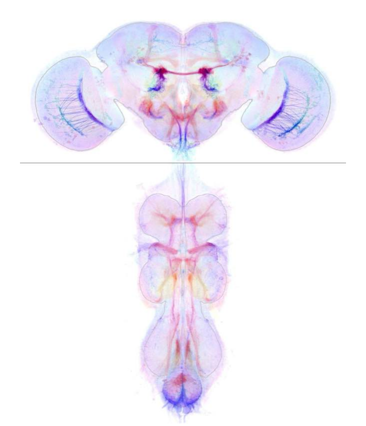
SS40062

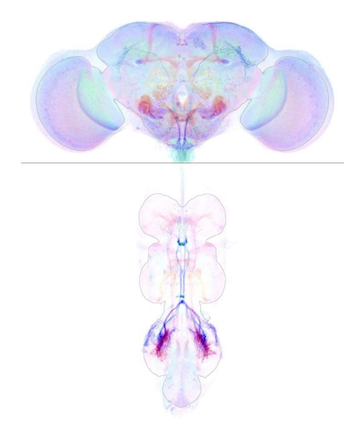
SS40134

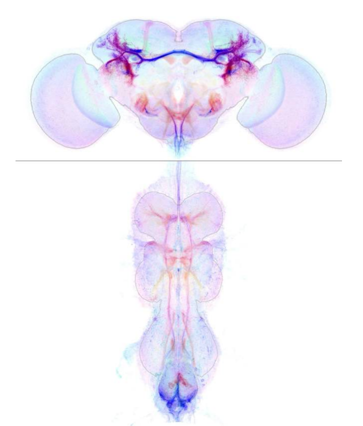
SS40191

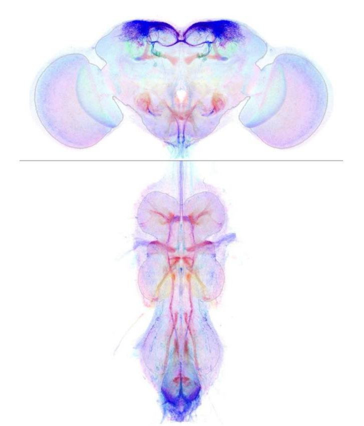
SS40217

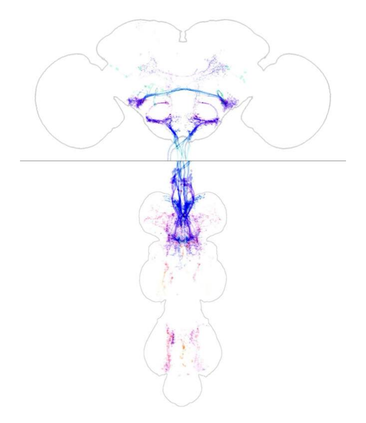
SS40261

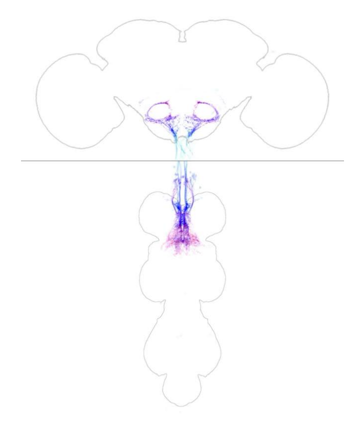
SS40265

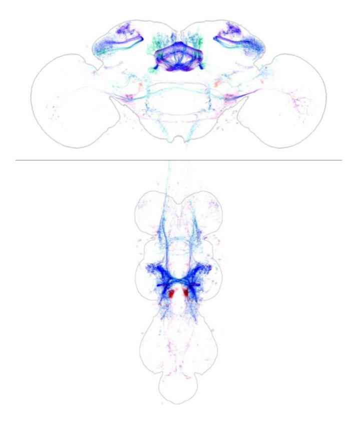
SS40272

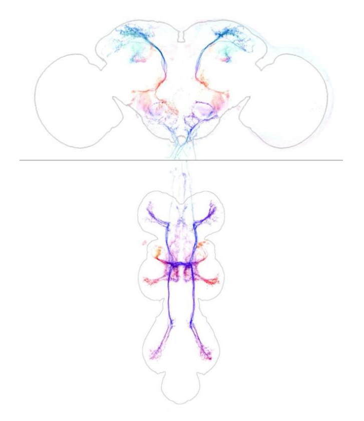
SS40274

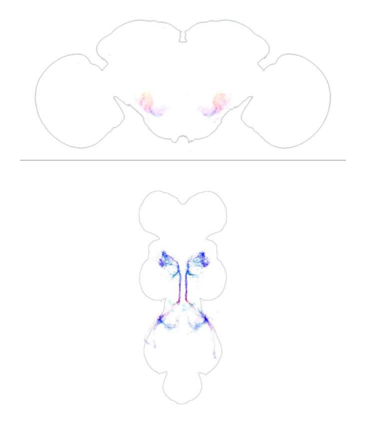
SS40275

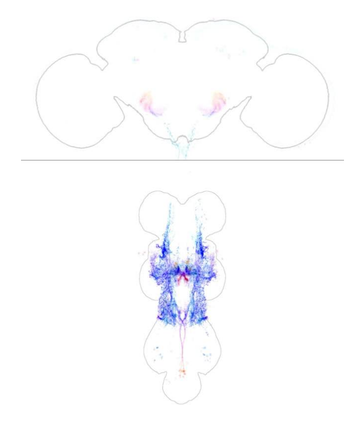
SS40279

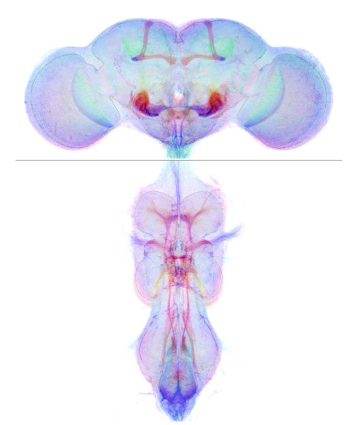
SS40280

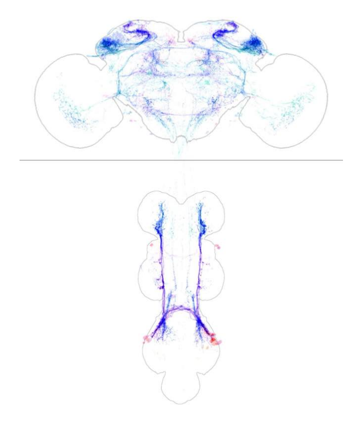
SS40286

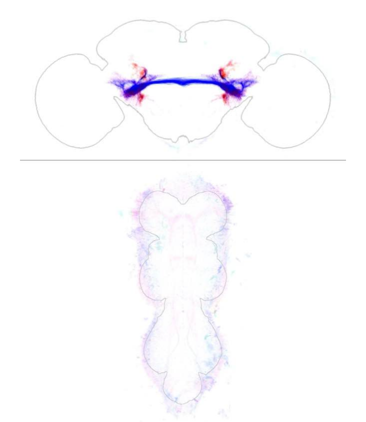
SS40316

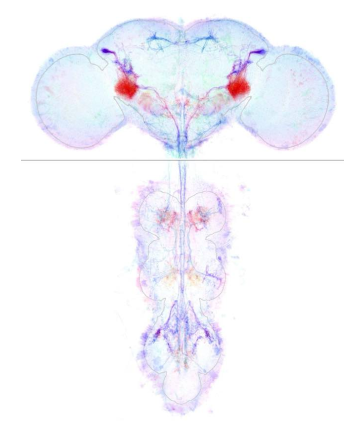
SS40336

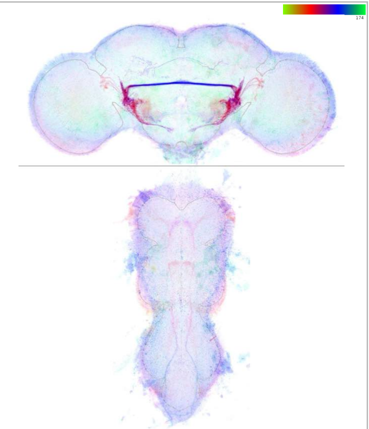

SS40361

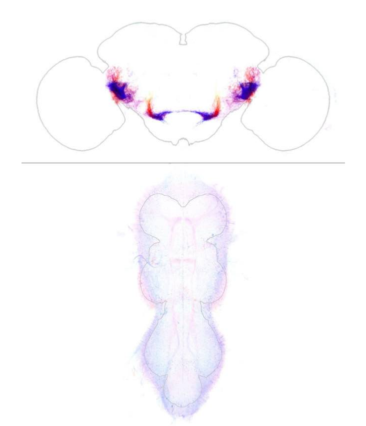

SS40366

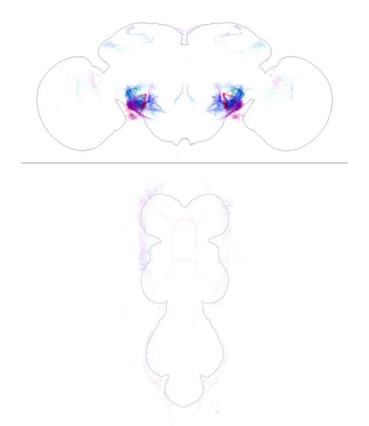

SS40380

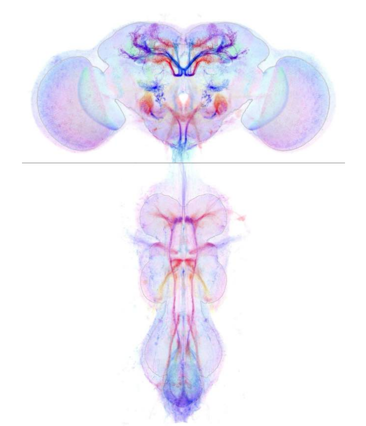

SS40401

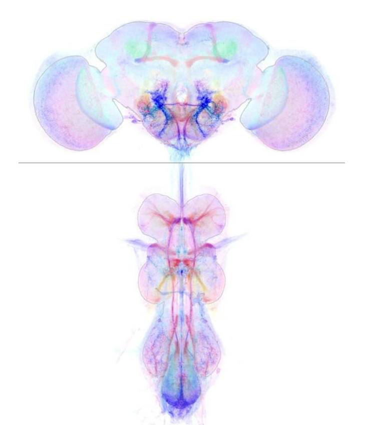

SS40402

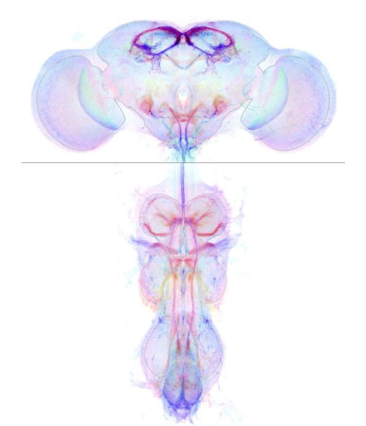

SS40414

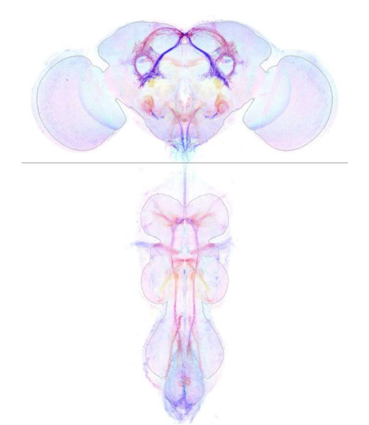

SS40429

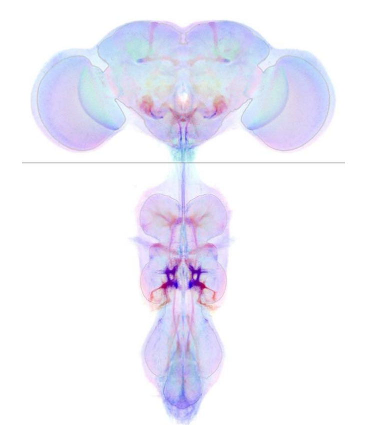

SS40449

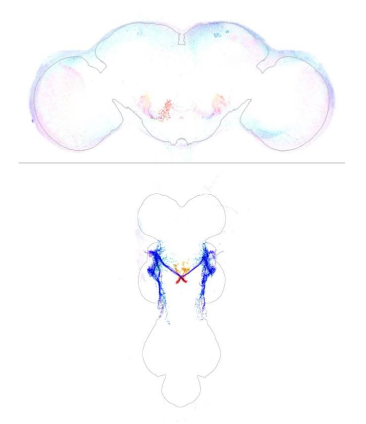

SS40456

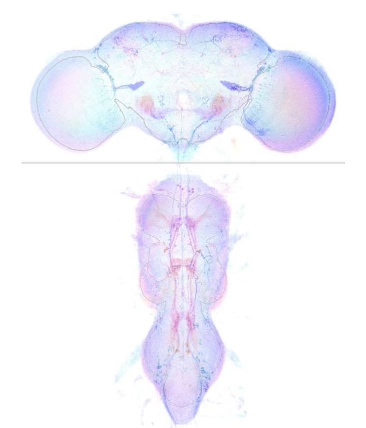

SS40489

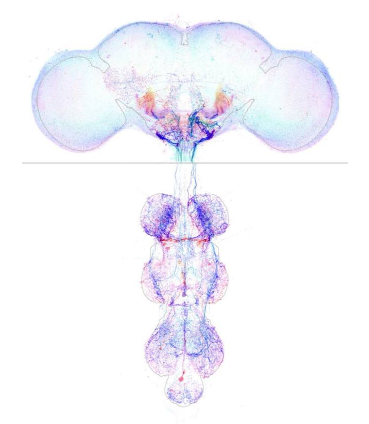

SS40619

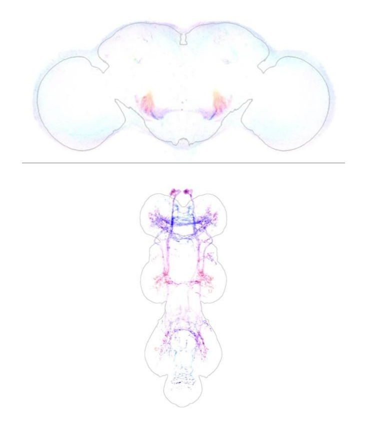

SS40645

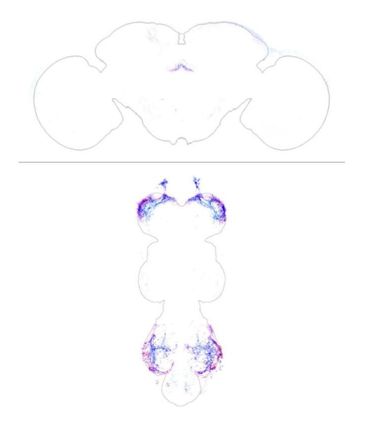

SS40657

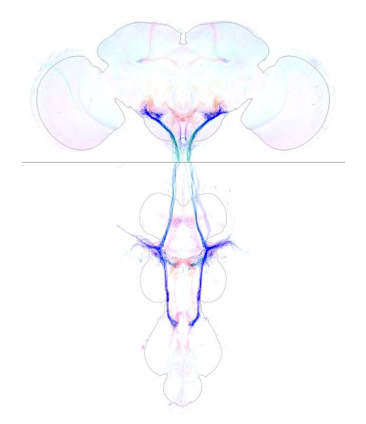

SS40775

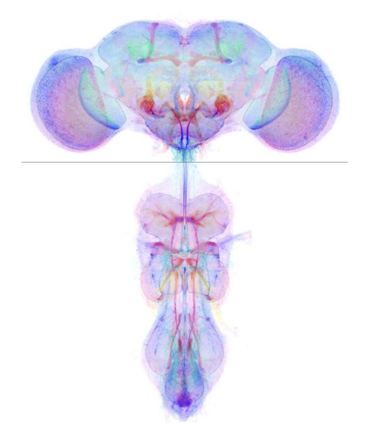

SS40778

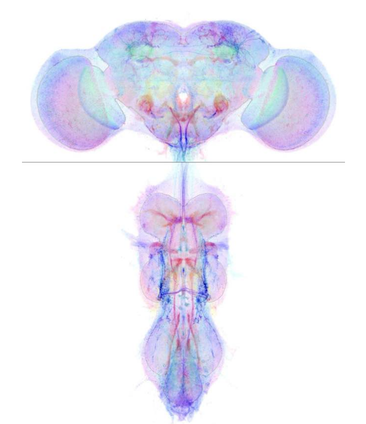

SS40782

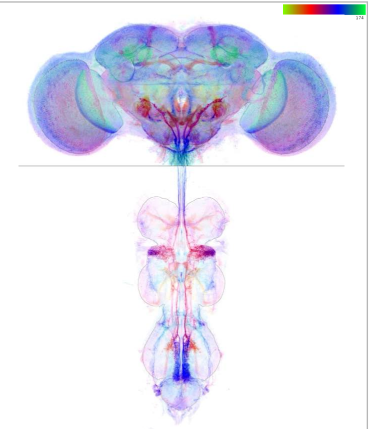
SS40789

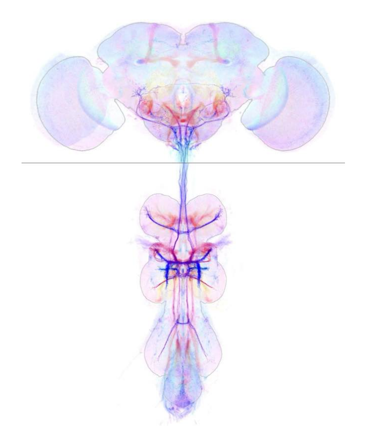
SS40794

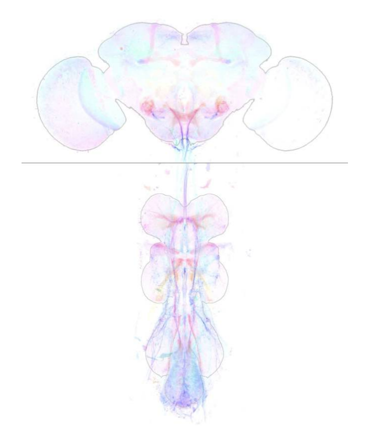
SS40851

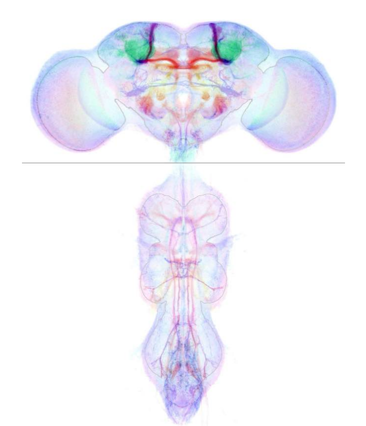
SS40865

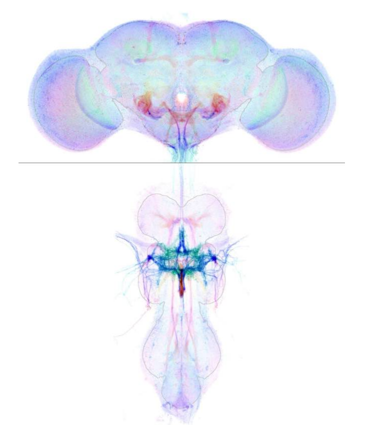
SS40867

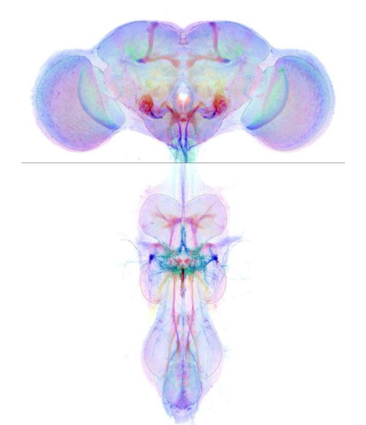
SS40868

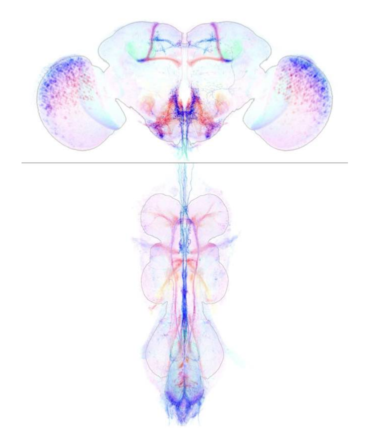
SS40926

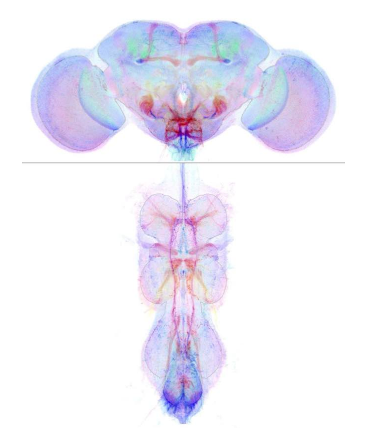
SS40945

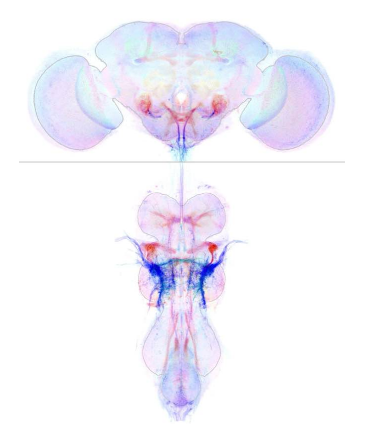
SS40980

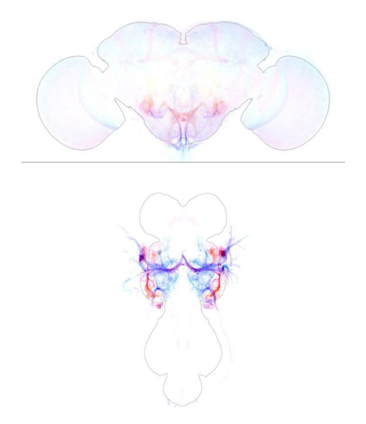
SS41027

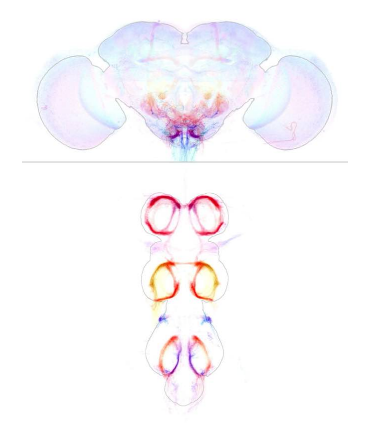
SS41034

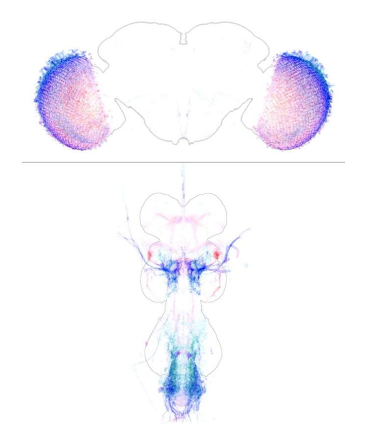
SS41039

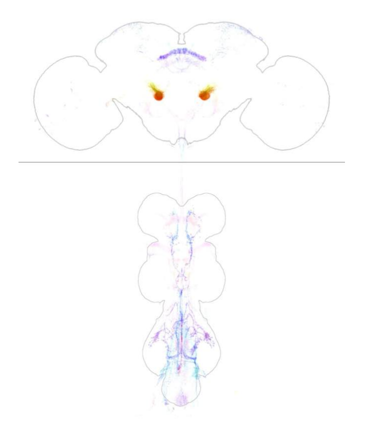
SS41041

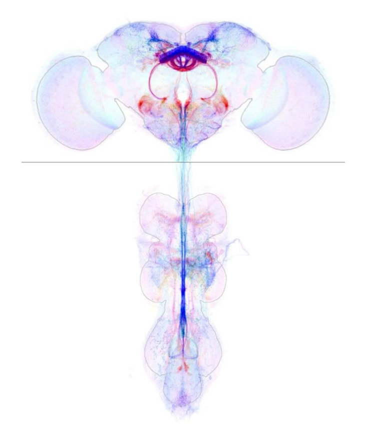
SS41052

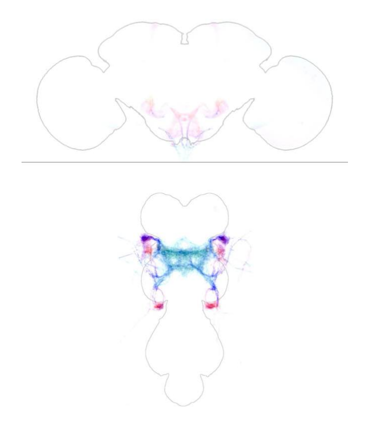
SS41068

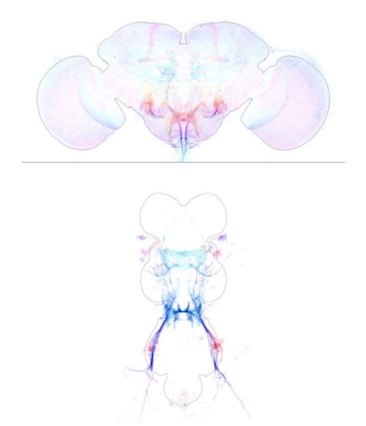
SS41075

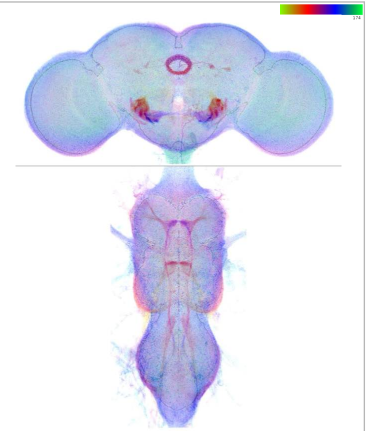
SS41209

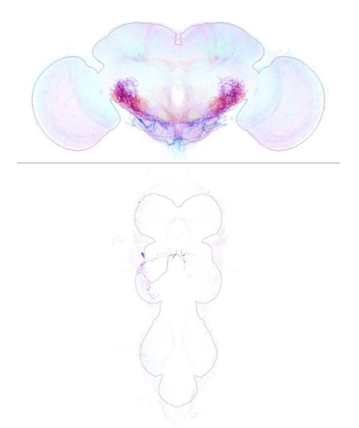
SS41402

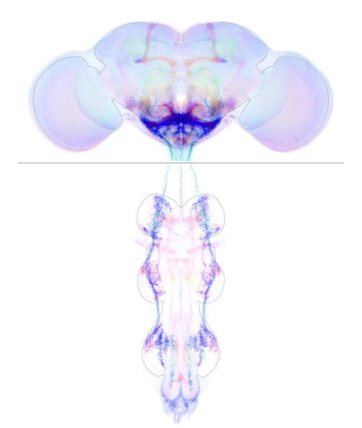
SS41446

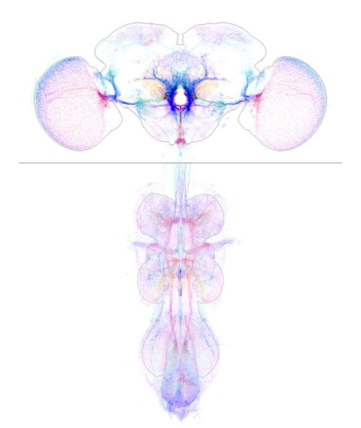
SS41506

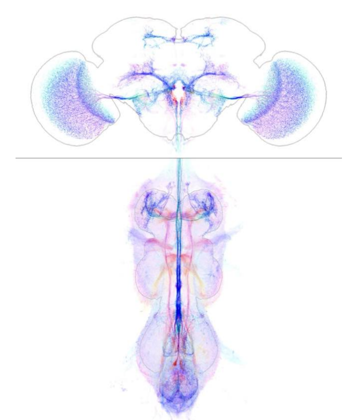
SS41518

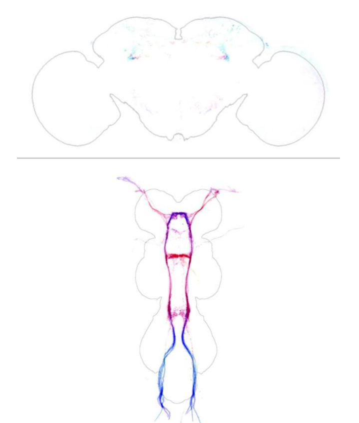
SS41564

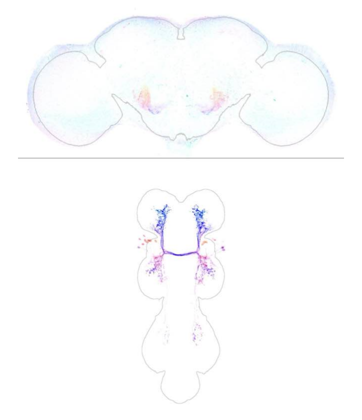
SS41570

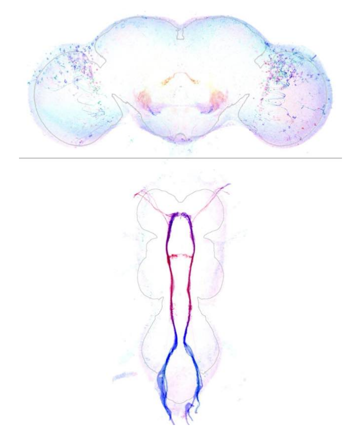
SS41581

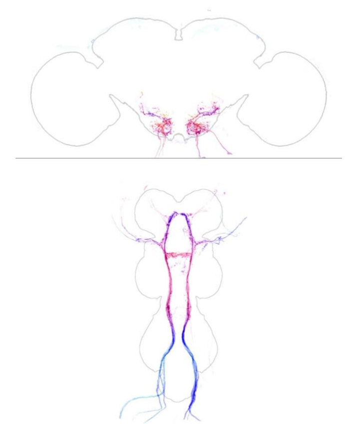
SS41582

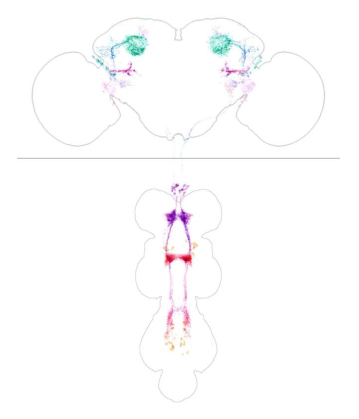
SS41584

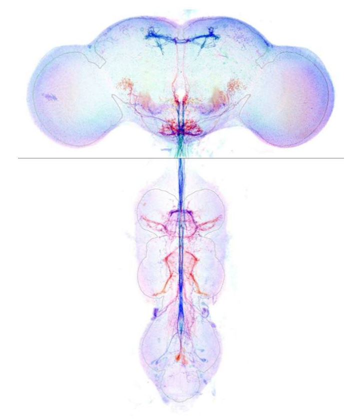
SS41605

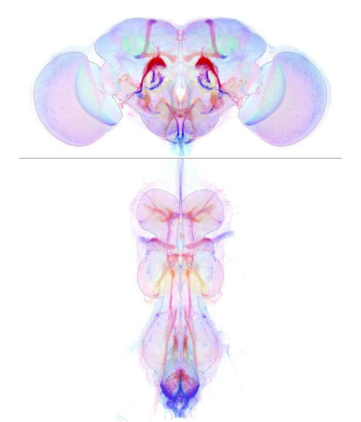
SS41646

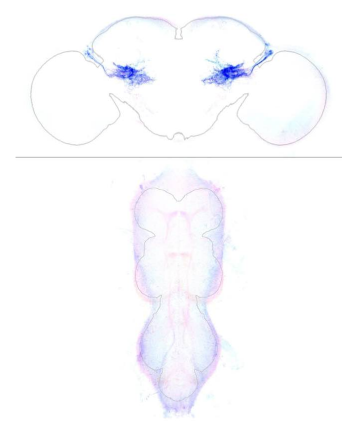
SS41704

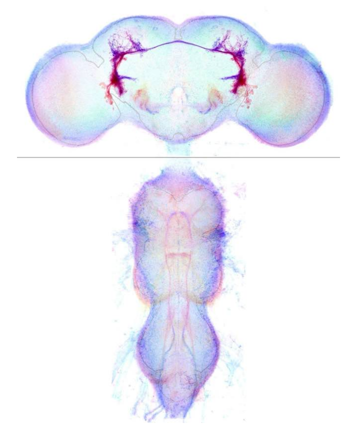
SS41728

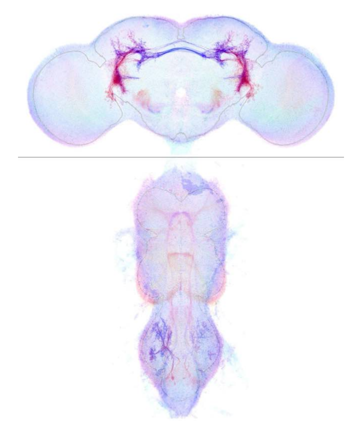
SS41730

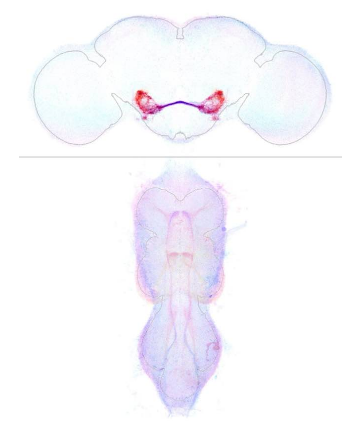
SS41739

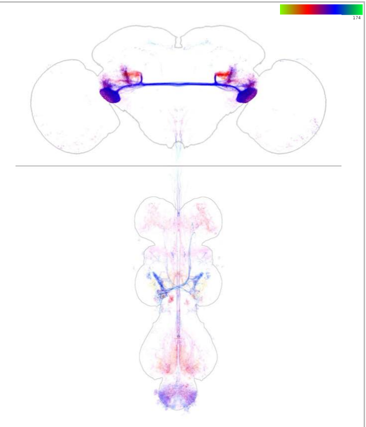
SS41752

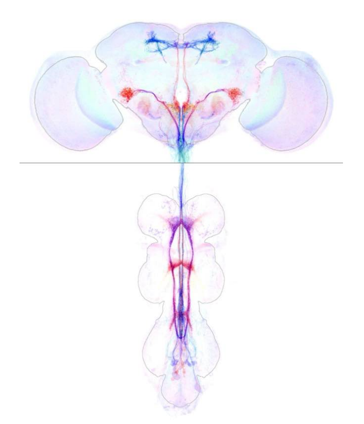
SS41806

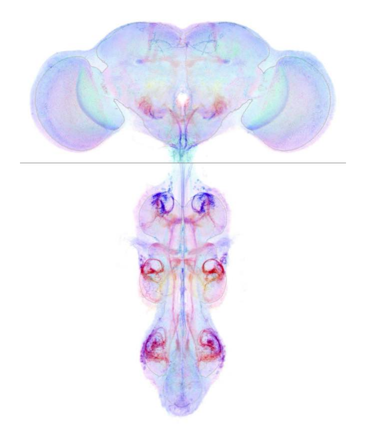
SS41811

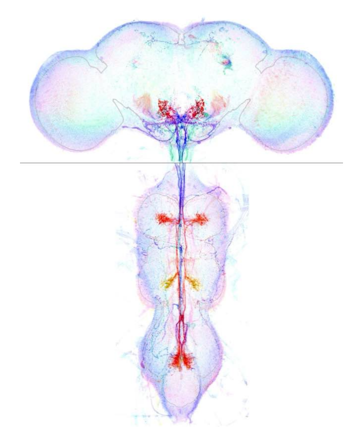
SS41815

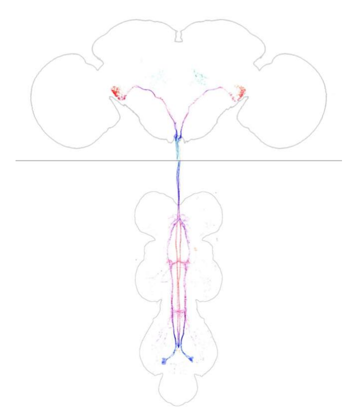
SS41822

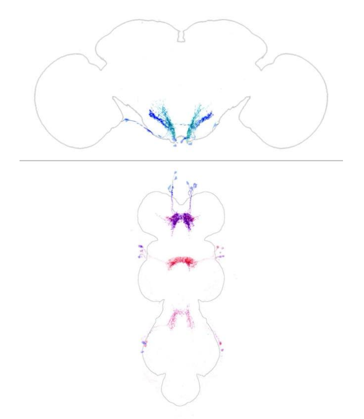
SS41835

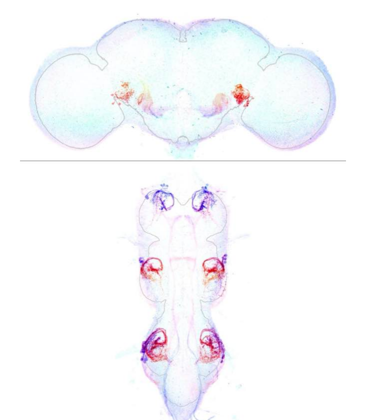
SS41862

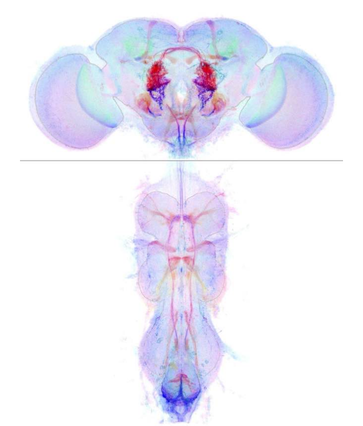
SS41947

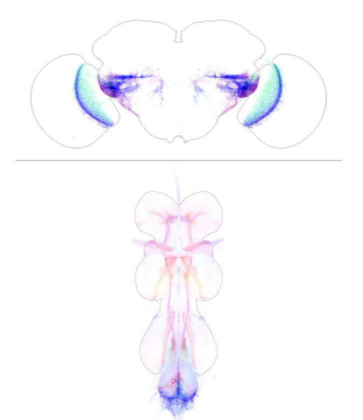
SS41950

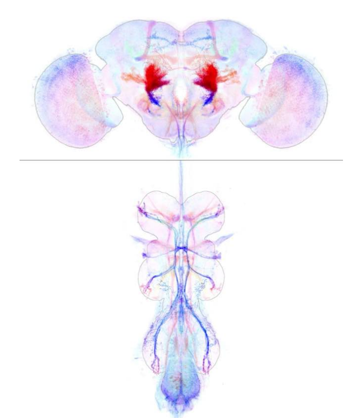
SS41970

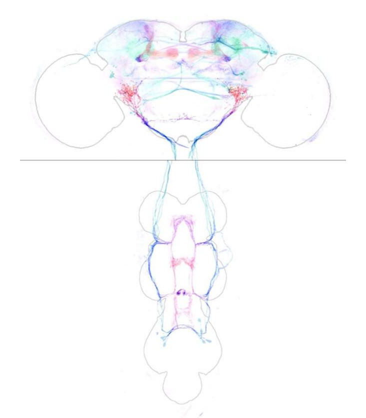
SS42008

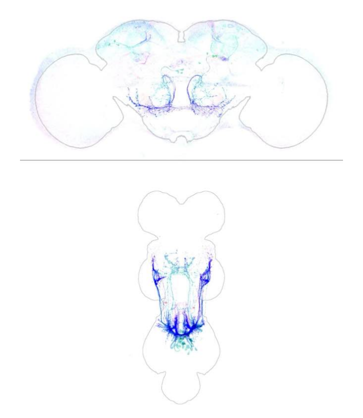
SS42050

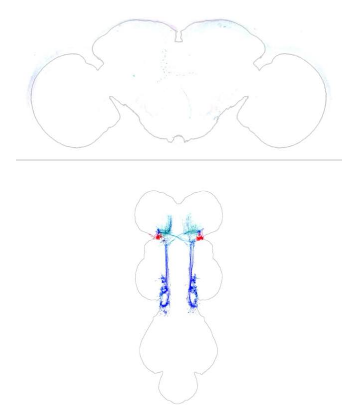
SS42079

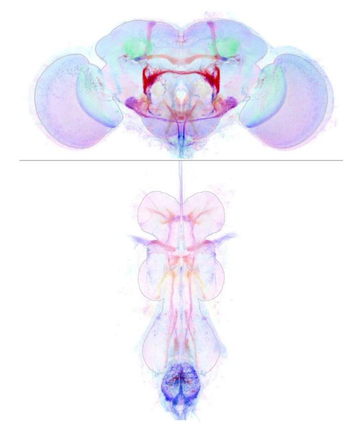
SS42258

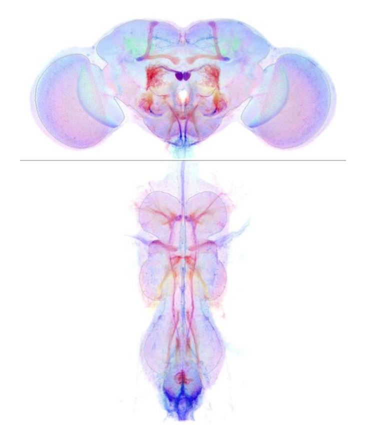
SS42273

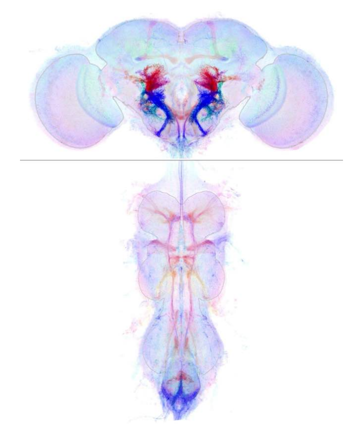
SS42309

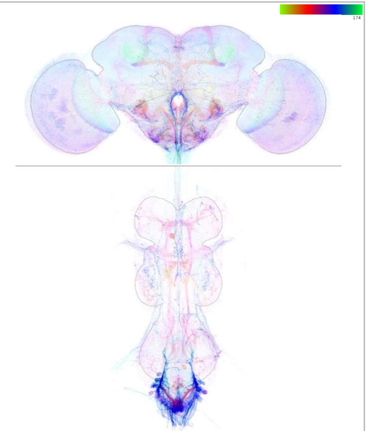
SS42328

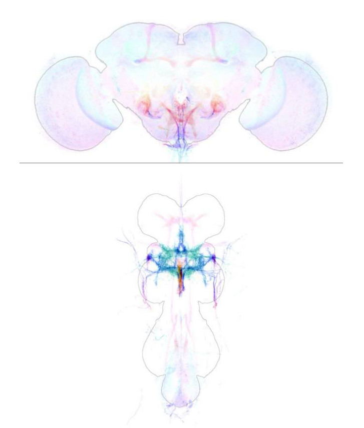
SS42385

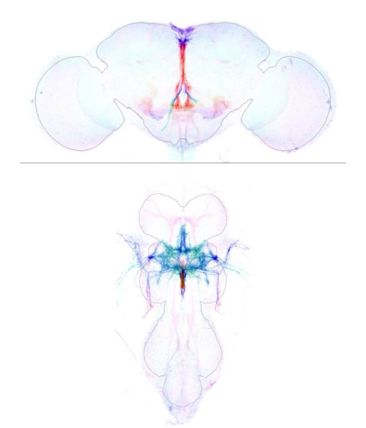
SS42389

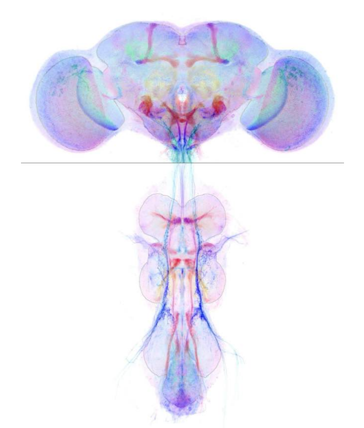
SS42406

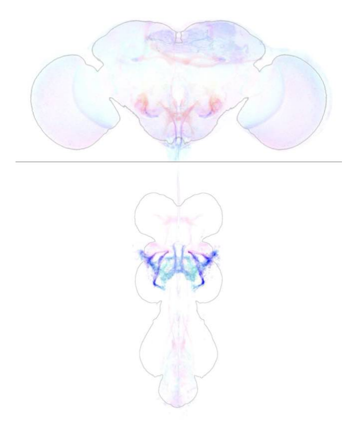
SS42438

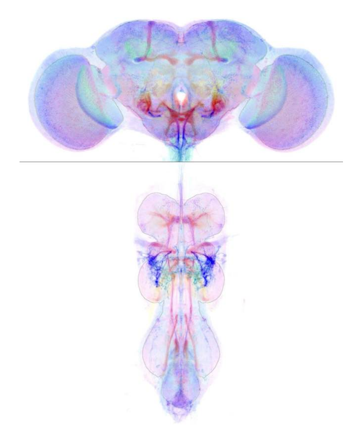
SS42439

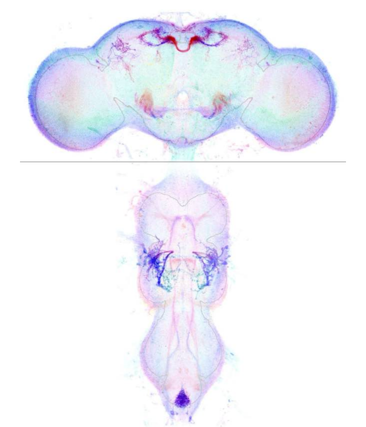
SS42442

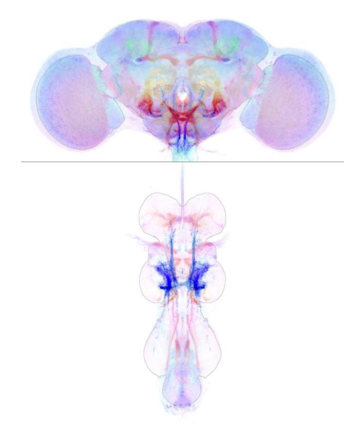
SS42446

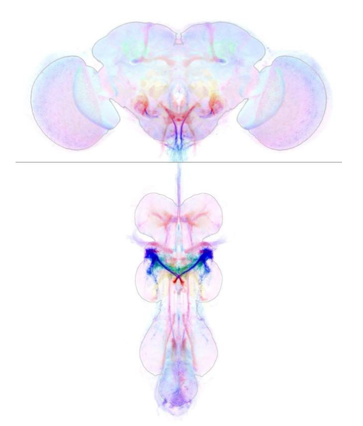
SS42447

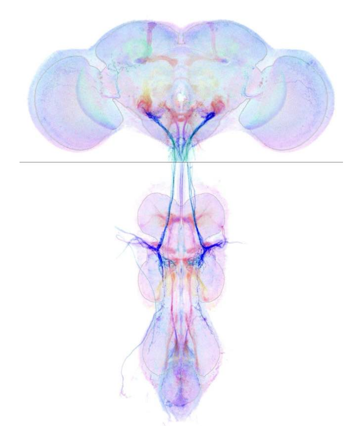
SS42461

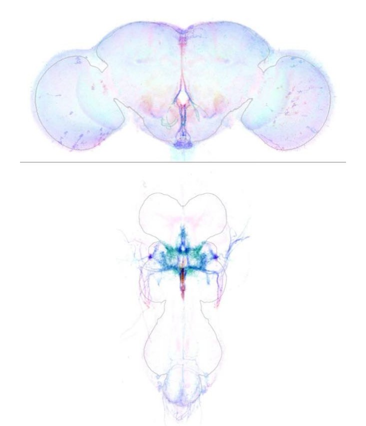
SS42463

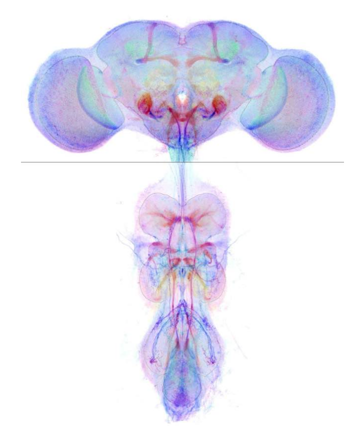
SS42464

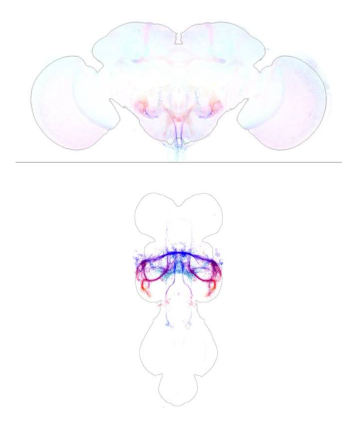
SS42465

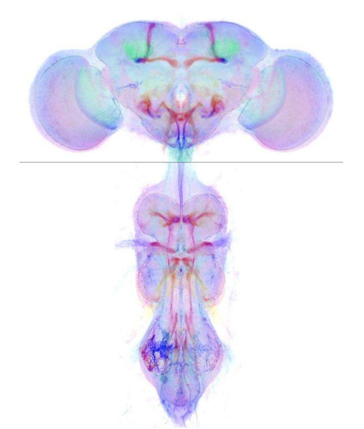
SS42475

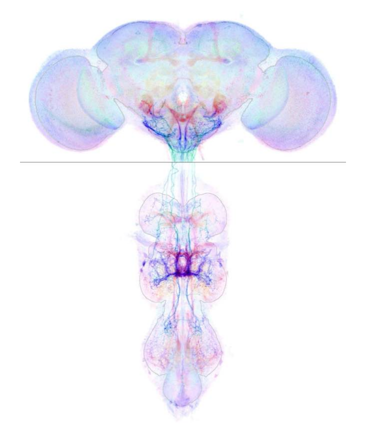
SS42486

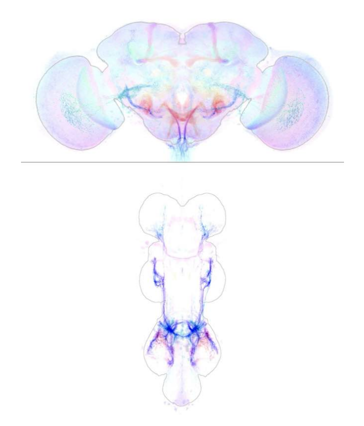
SS42493

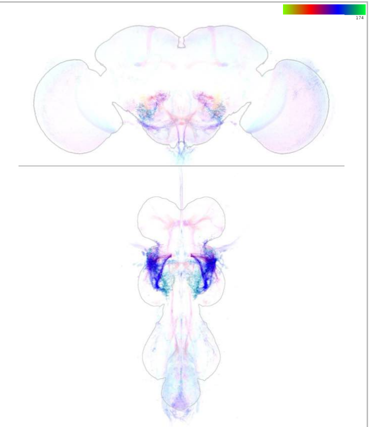
SS42498

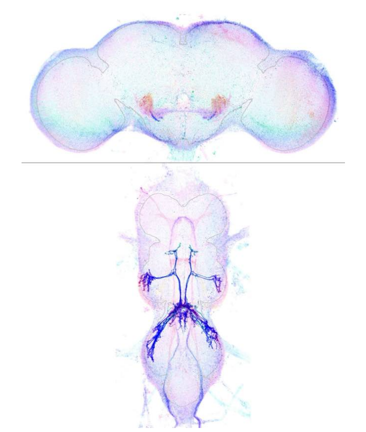
SS42499

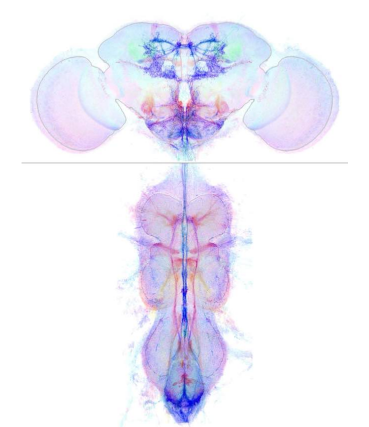
SS42634

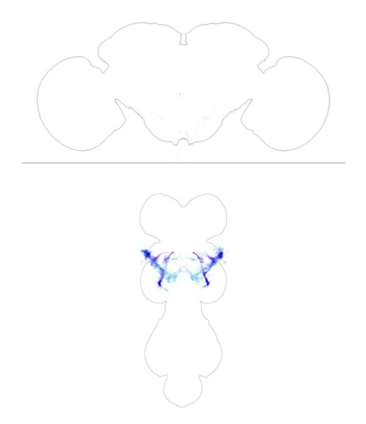
SS42738

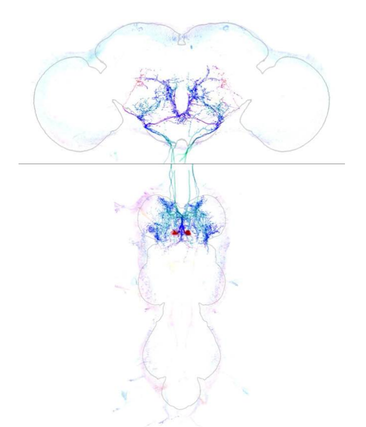
SS42740

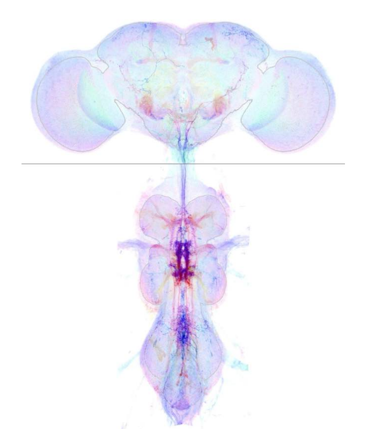
SS42744

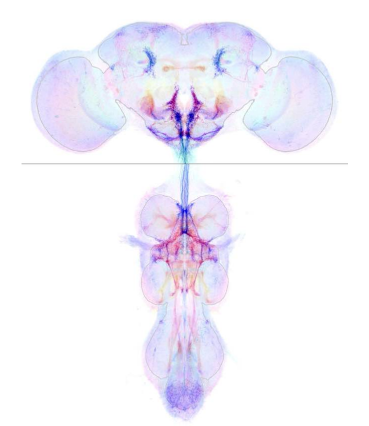
SS42749

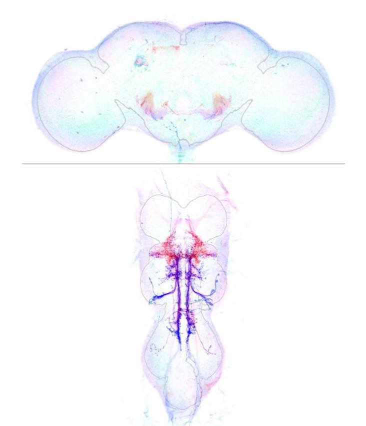
SS42757

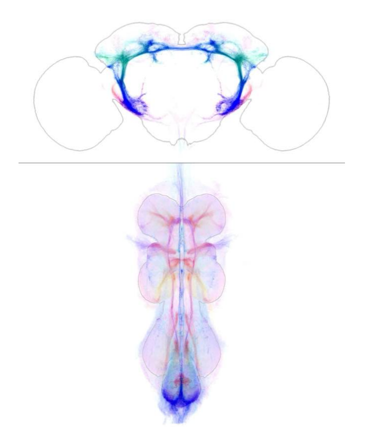
SS42960

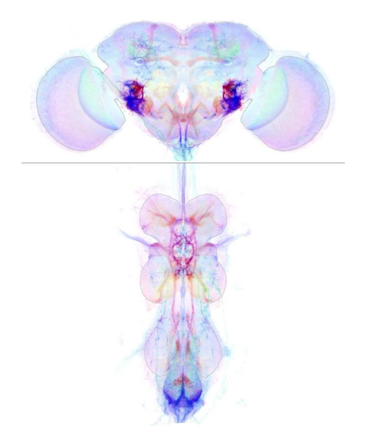
SS42965

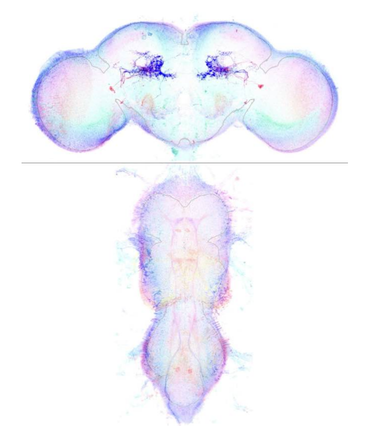
SS42993

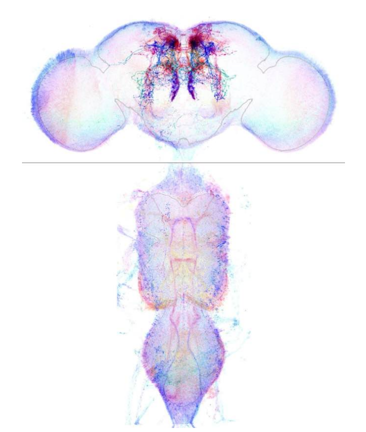
SS43014

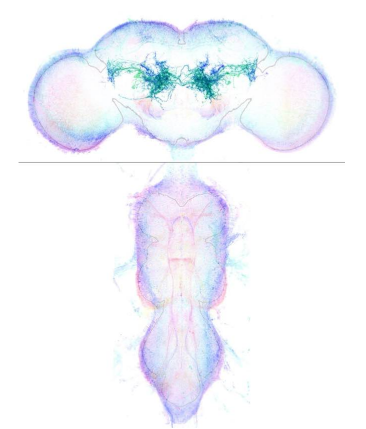
SS43026

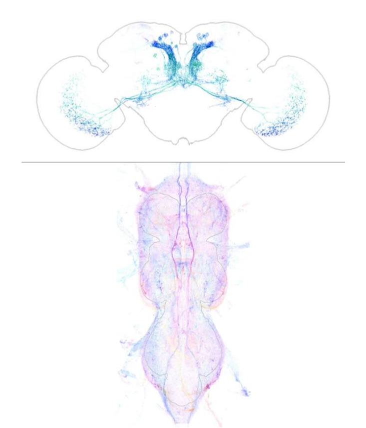
SS43117

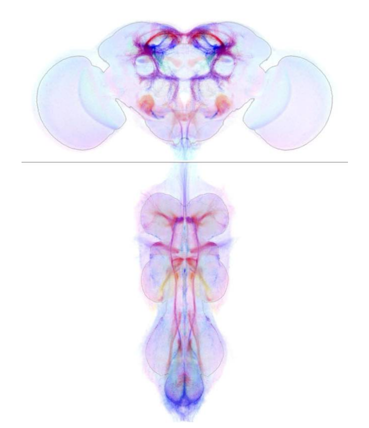
SS43274

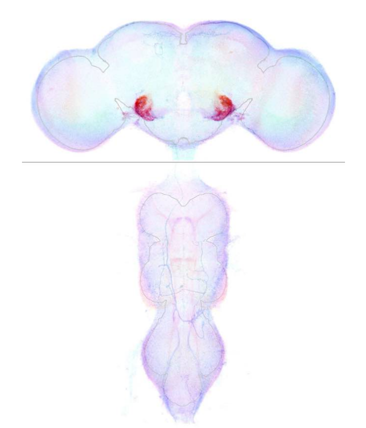
SS43321

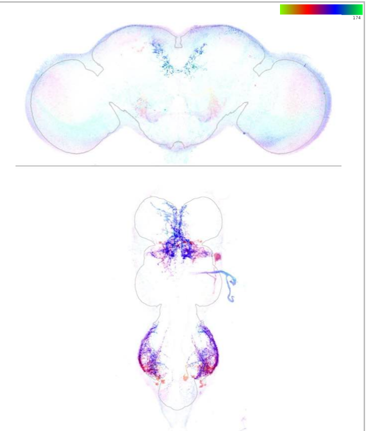
SS43399

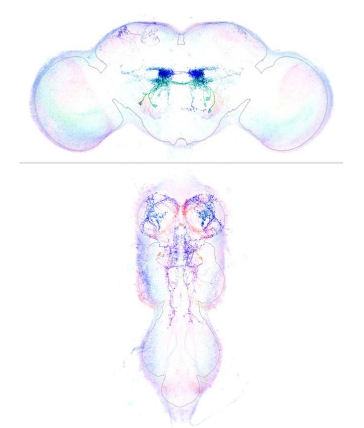
SS43425

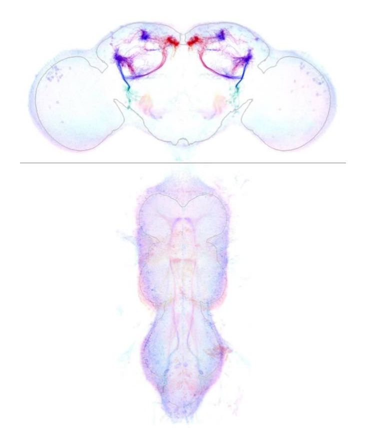
SS43466

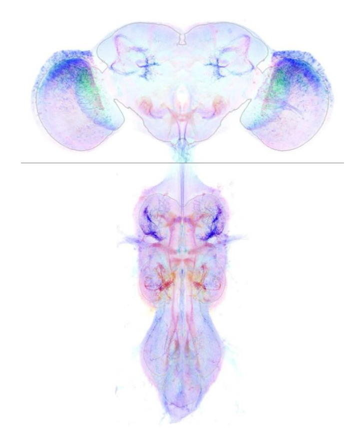
SS43542

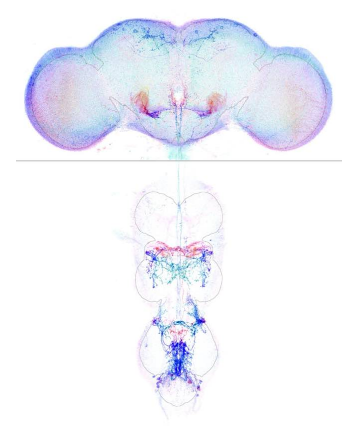
SS43546

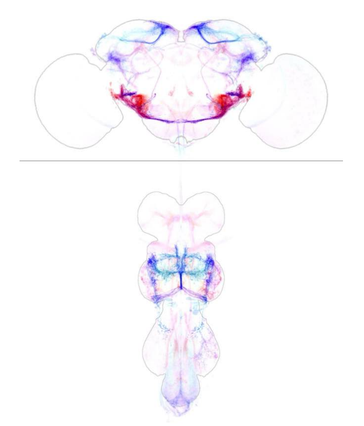
SS43574

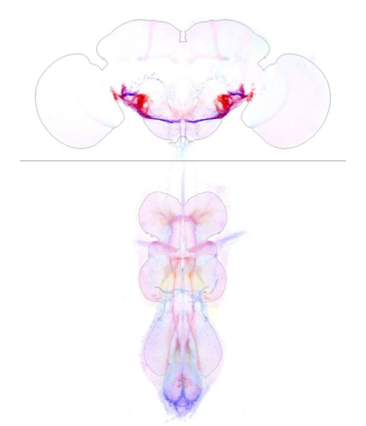
SS43583

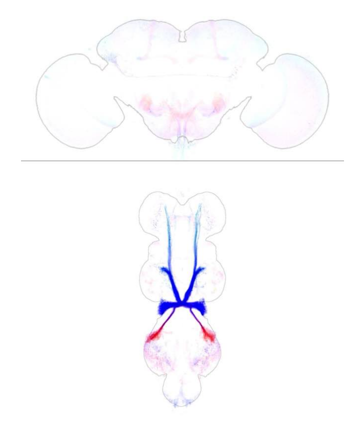
SS43617

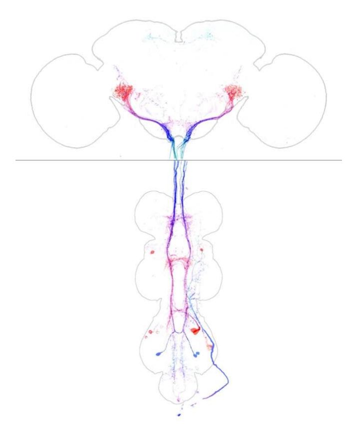
SS43652

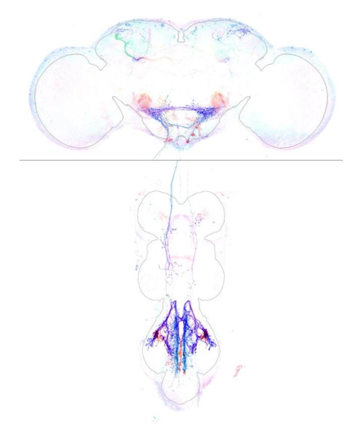
SS43697

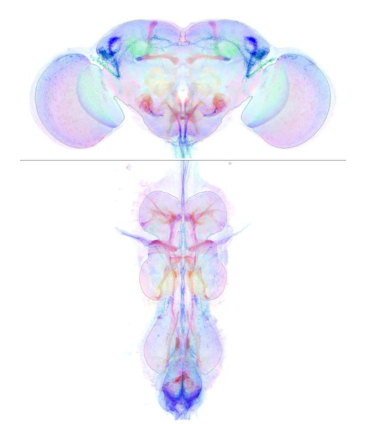
SS43925

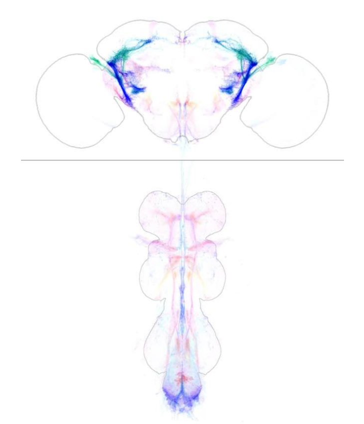
SS43930

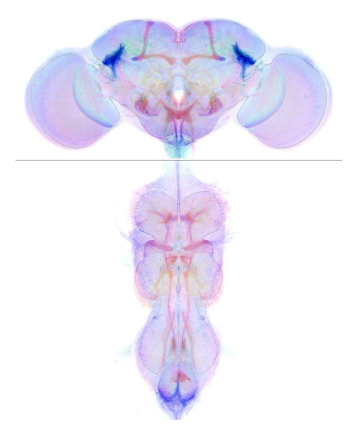
SS43937

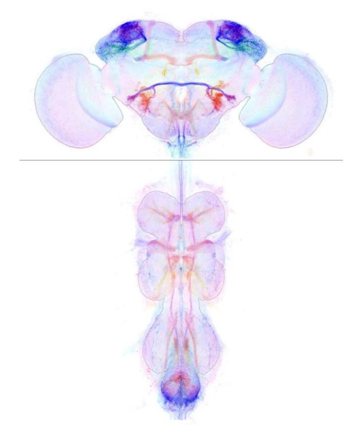
SS43943

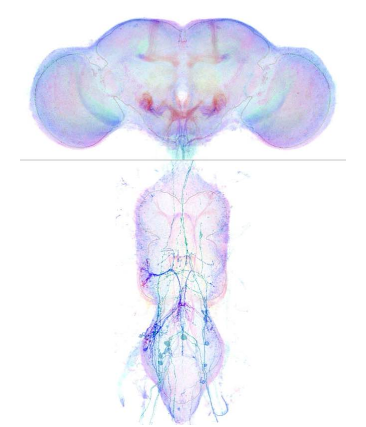
SS43979

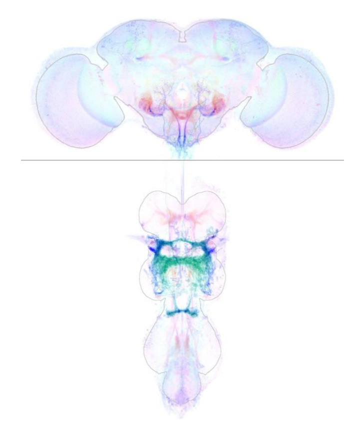
SS44000

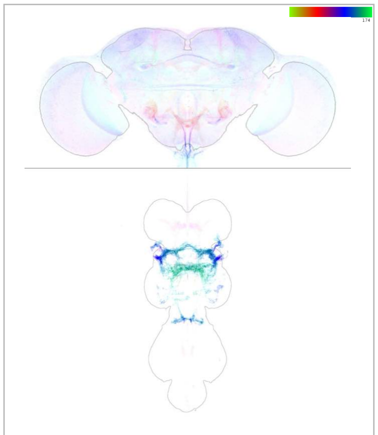
SS44002

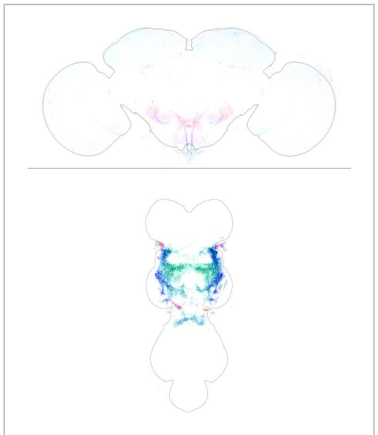
SS44003

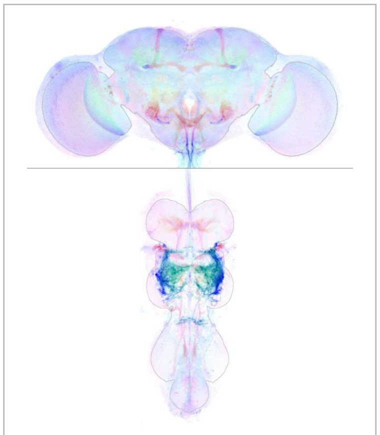
SS44005

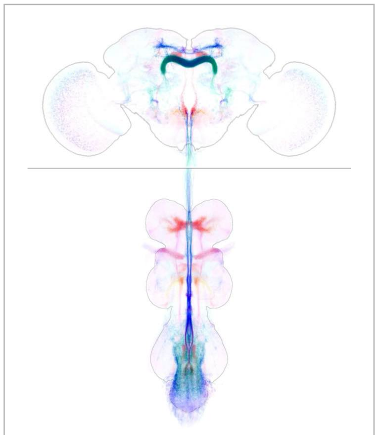
SS44009

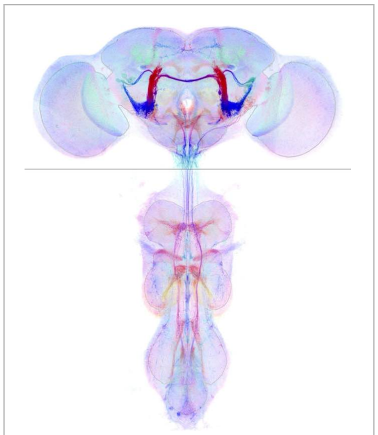
SS44028

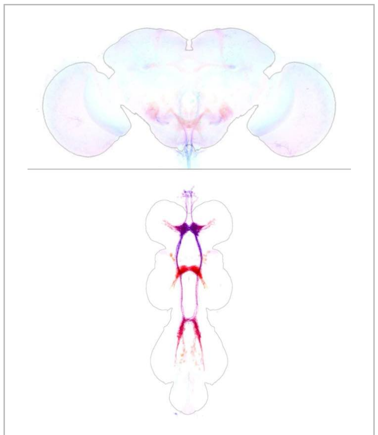
SS44034

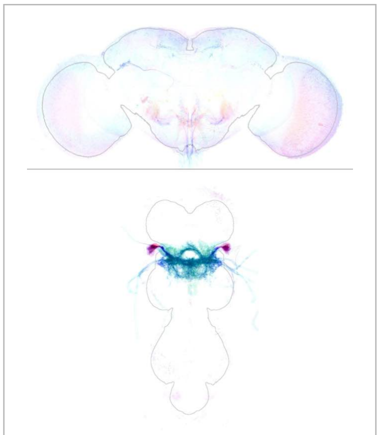
SS44039

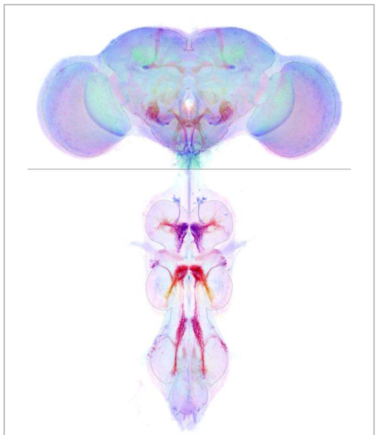
SS44046

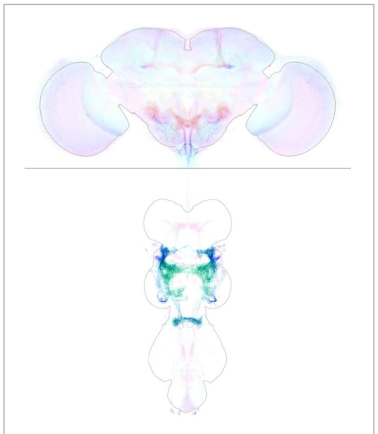
SS44051

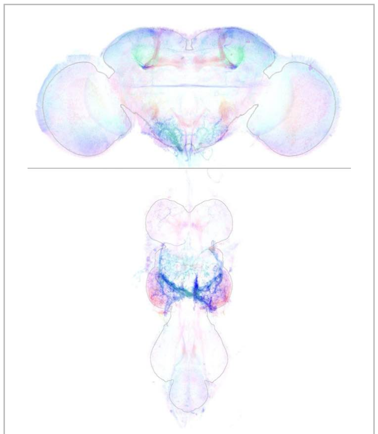
SS44054

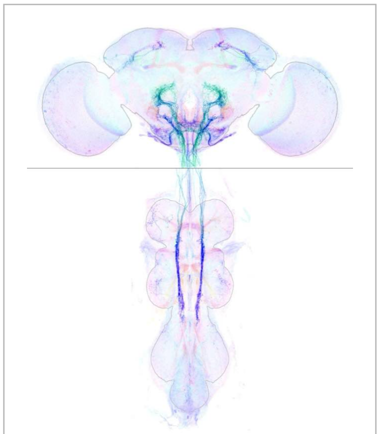
SS44071

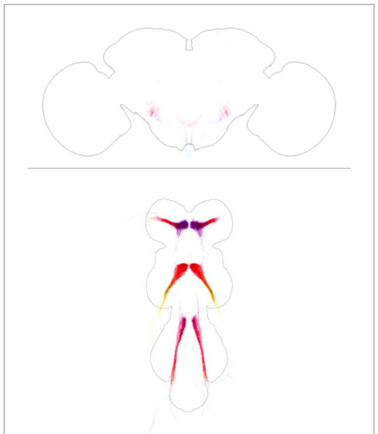
SS44081

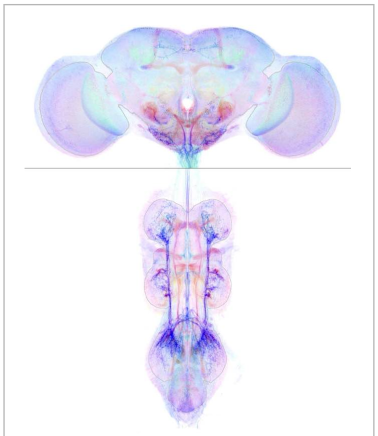
SS44225

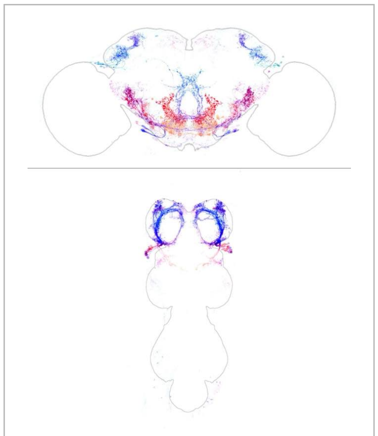
SS44233

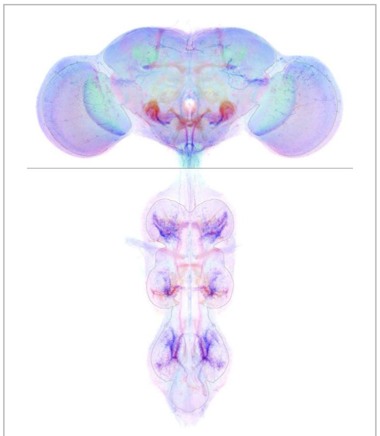
SS44254

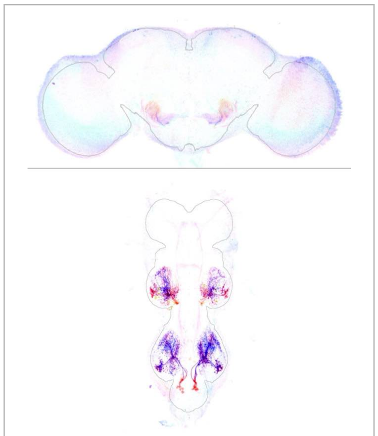
SS44255

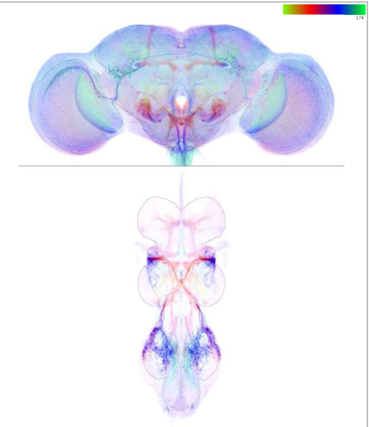
SS44256

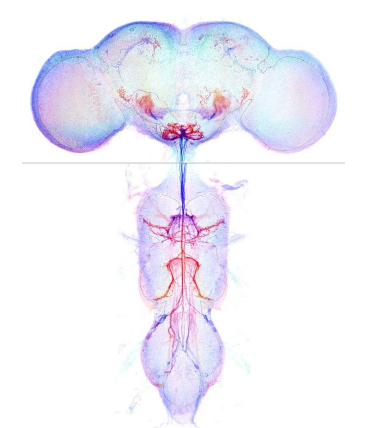
SS44270

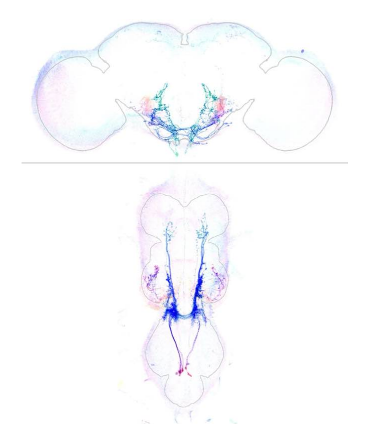
SS44276

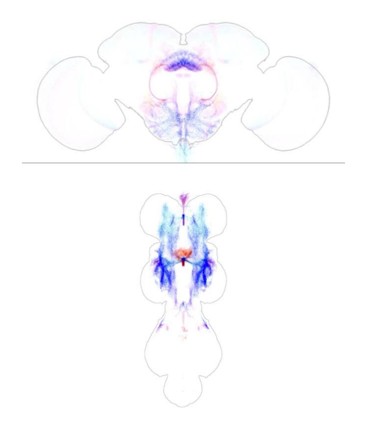
SS44314

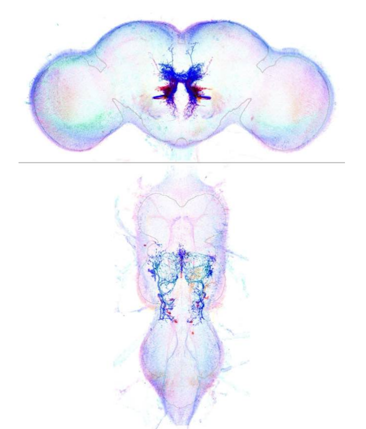
SS44348

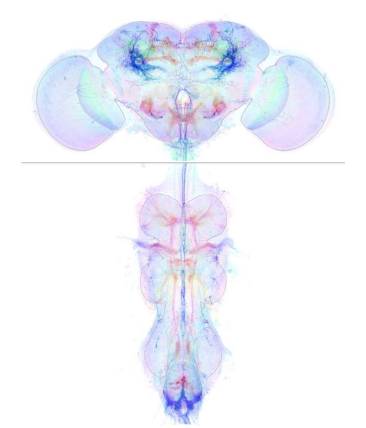
SS44399

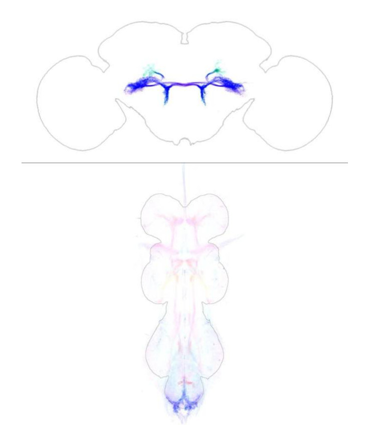
SS44402

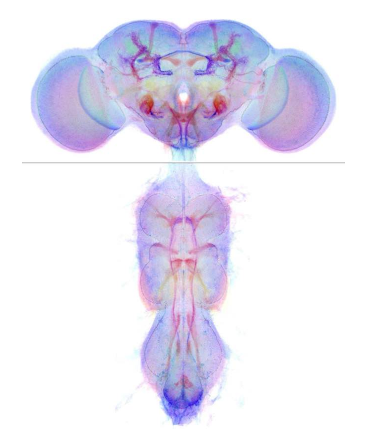
SS44408

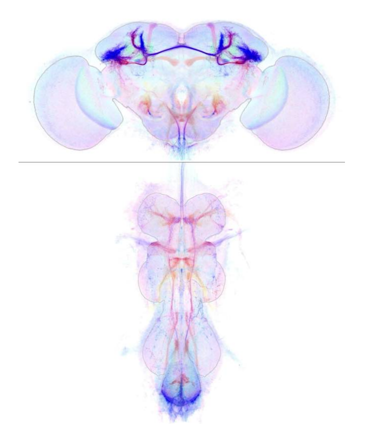
SS44414

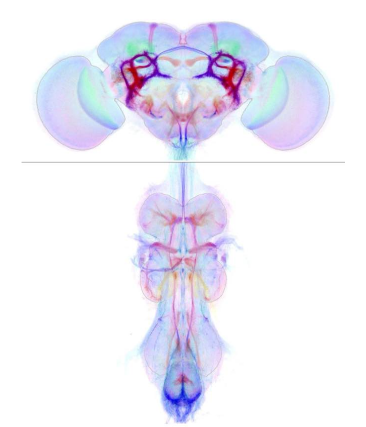
SS44435

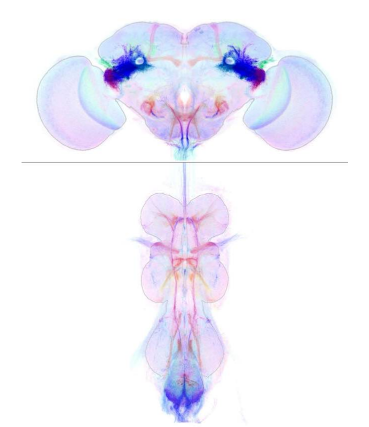
SS44443

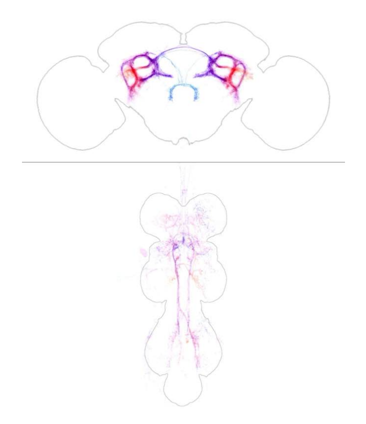
SS44448

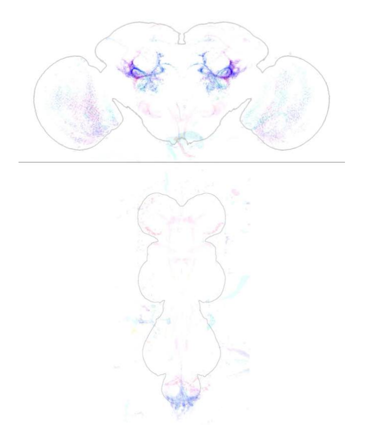
SS44452

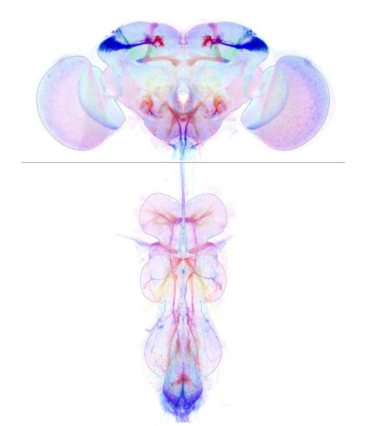
SS44460

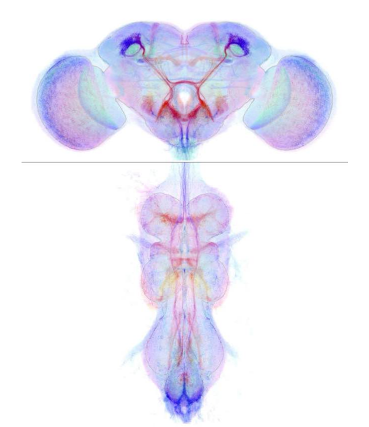
SS44477

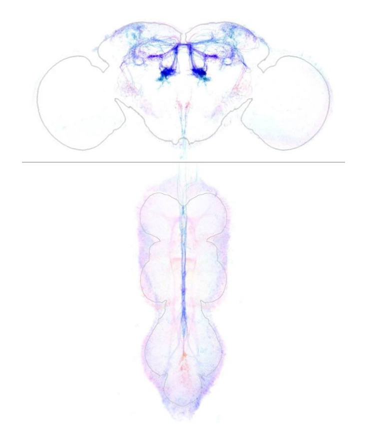
SS44510

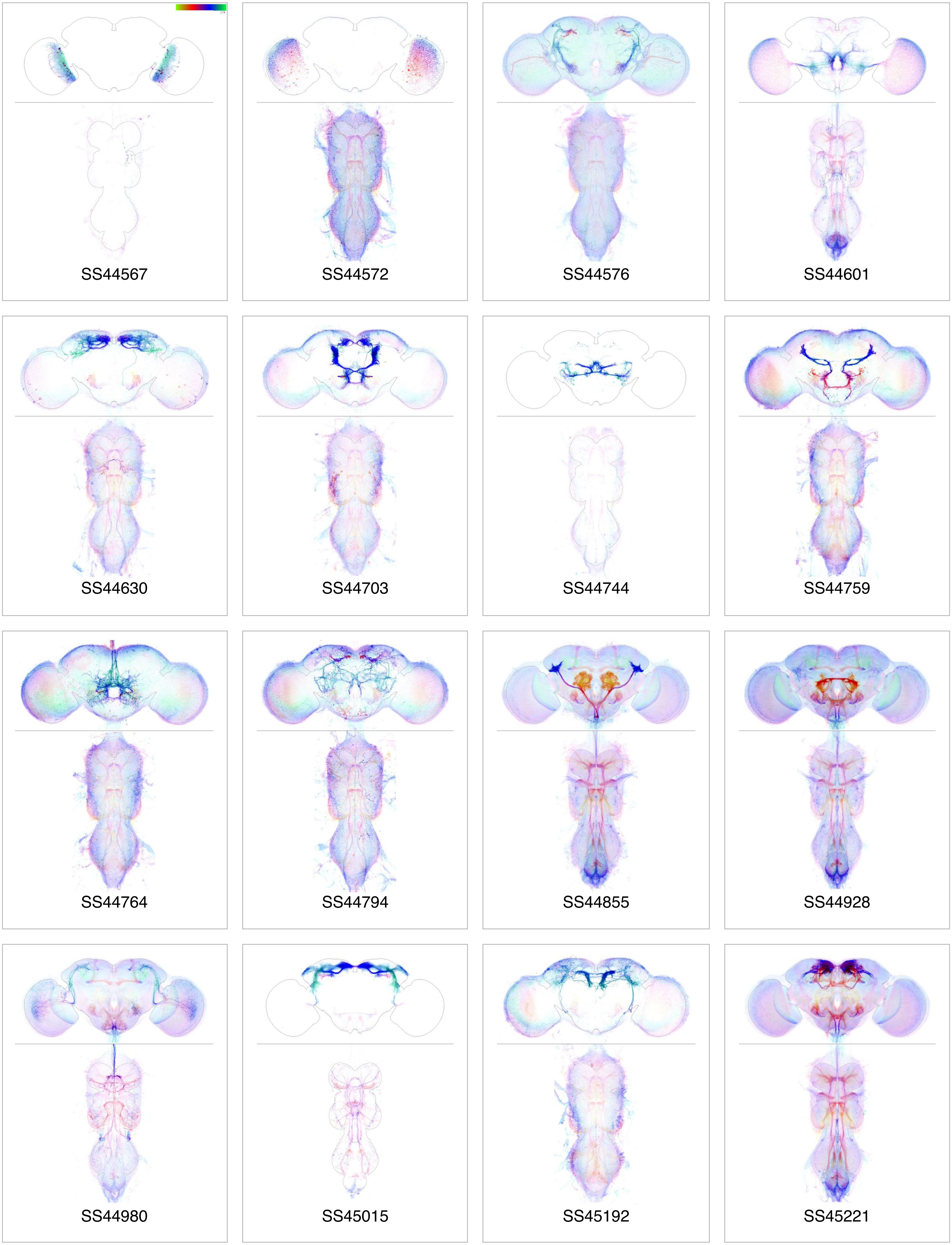
SS44567
SS44572
SS44576
SS44601
SS44630
SS44703
SS44744
SS44759
SS44764
SS44794
SS44855
SS44928
SS44980
SS45015
SS45192
SS45221

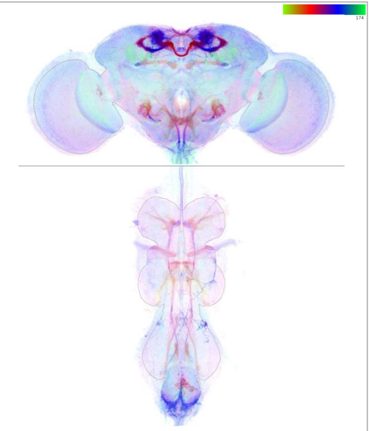
SS45226

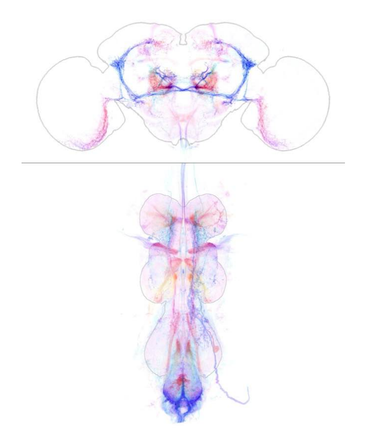
SS45229

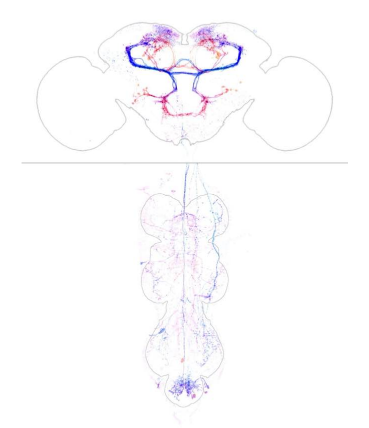
SS45231

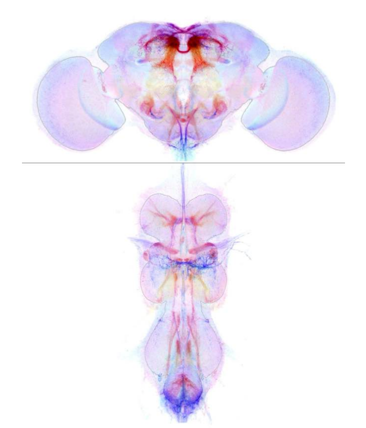
SS45251

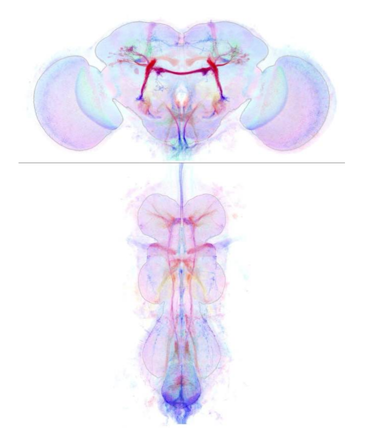
SS45254

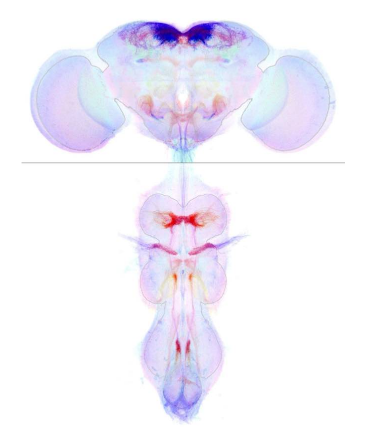
SS45308

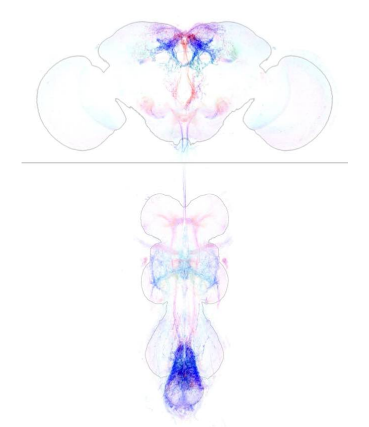
SS45310

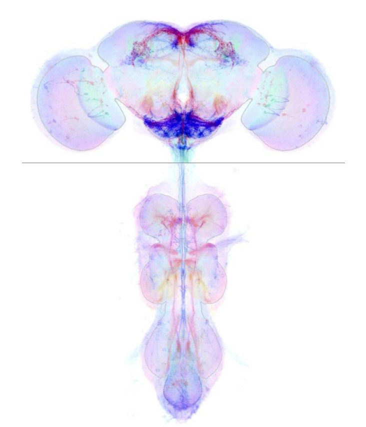
SS45323

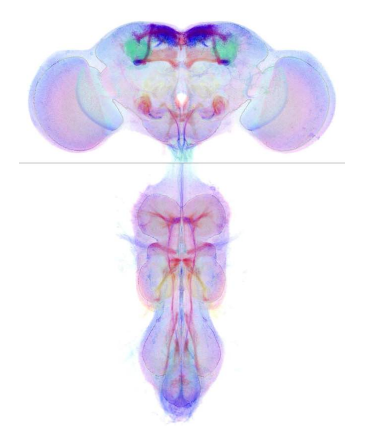
SS45327

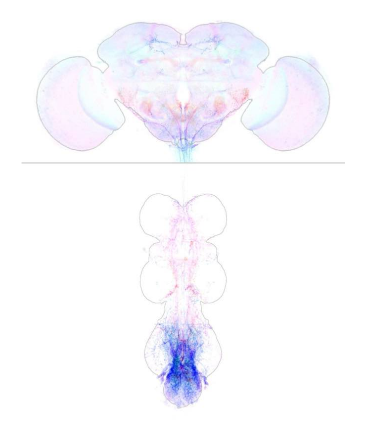
SS45333

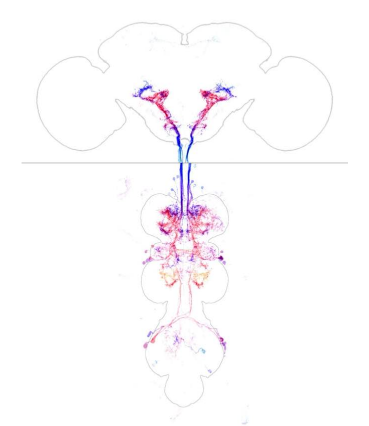
SS45363

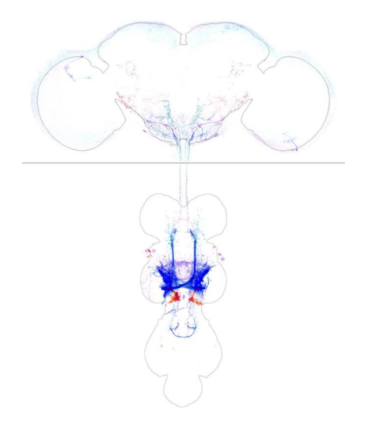
SS45385

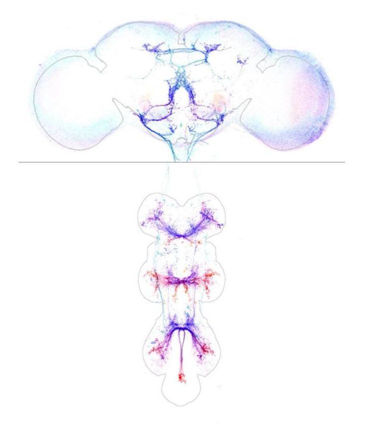
SS45404

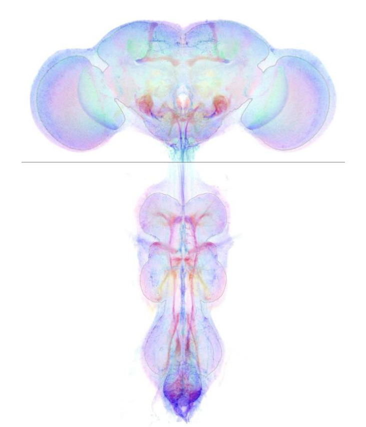
SS45406

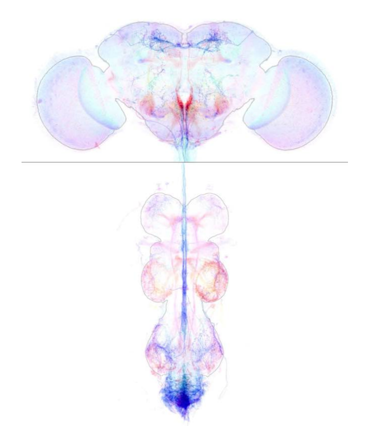
SS45413

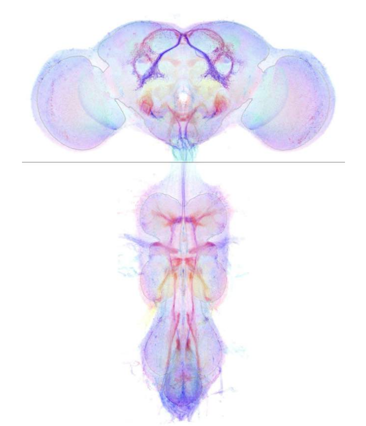
SS45418

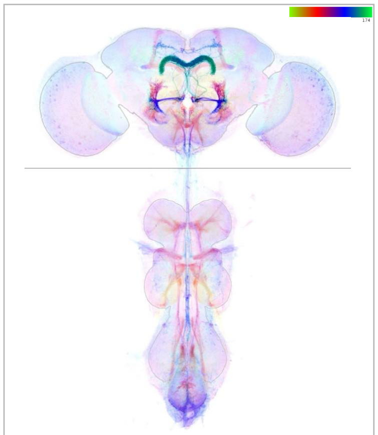
SS45421
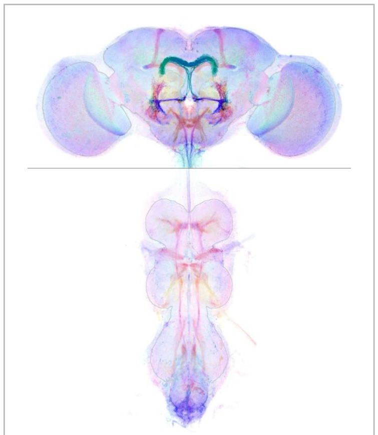
SS45422
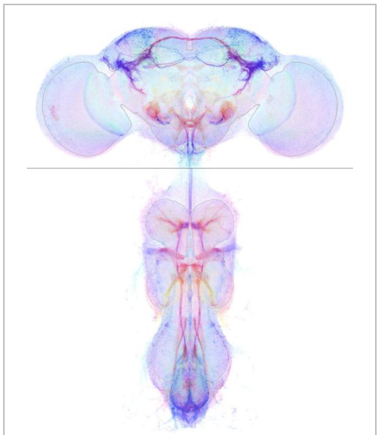
SS45428
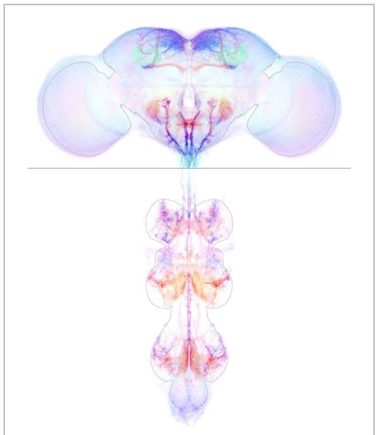
SS45429
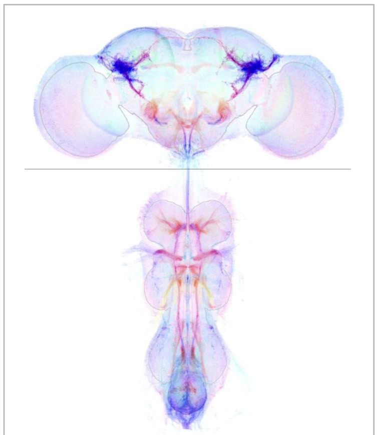
SS45434
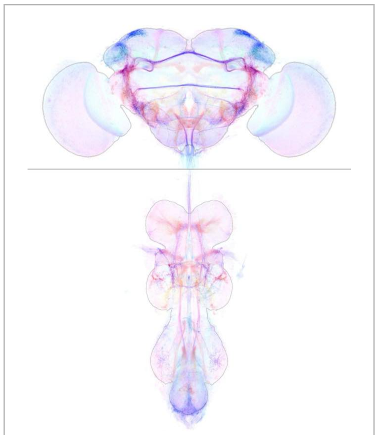
SS45444
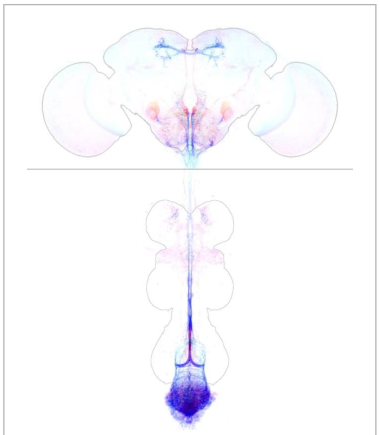
SS45446
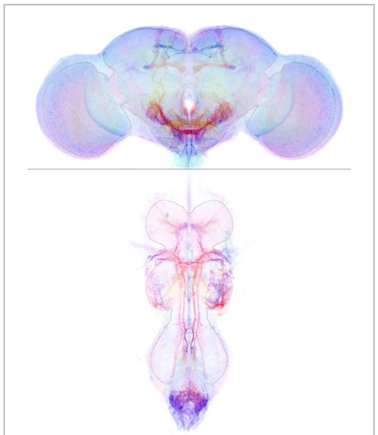
SS45454
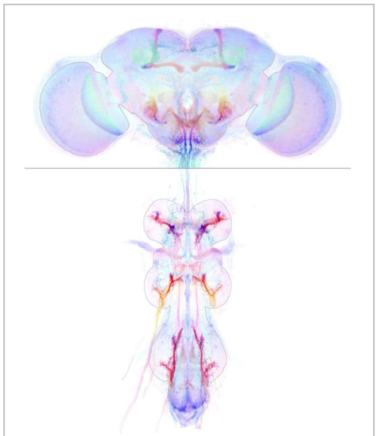
SS45463
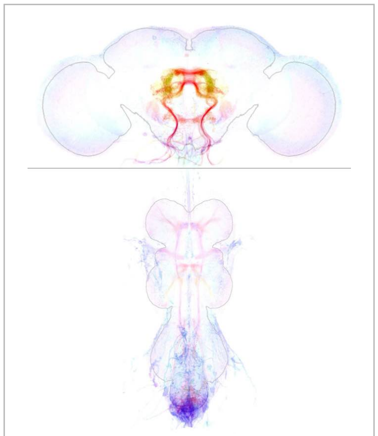
SS45465
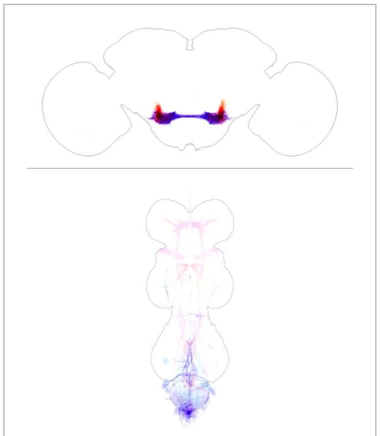
SS45466
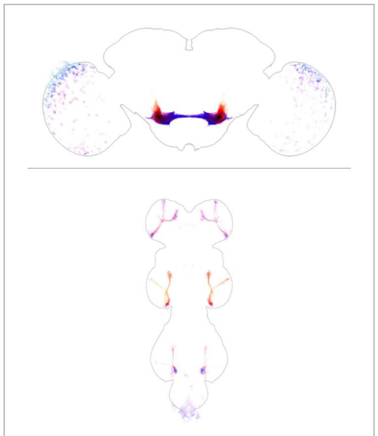
SS45467
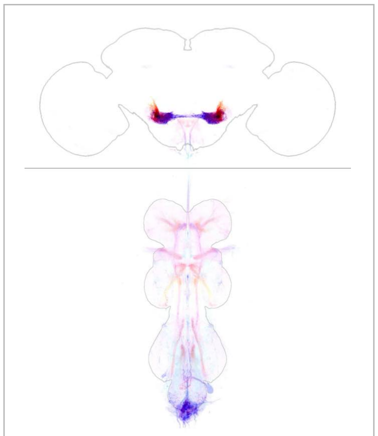
SS45470
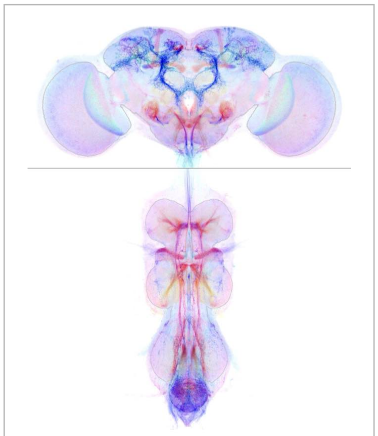
SS45476
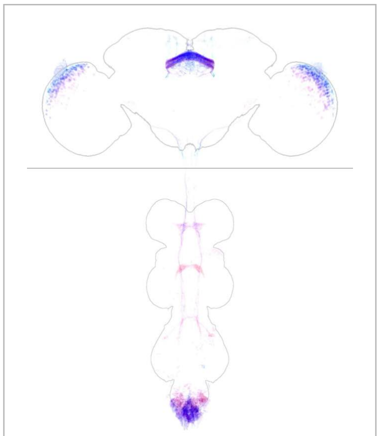
SS45478
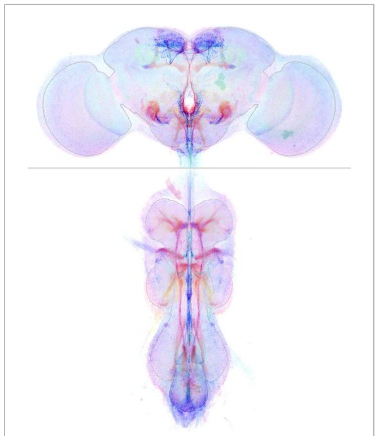
SS45479

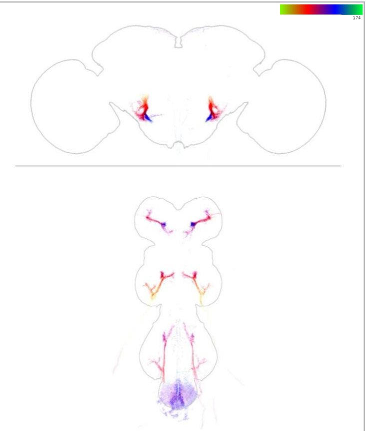
SS45484

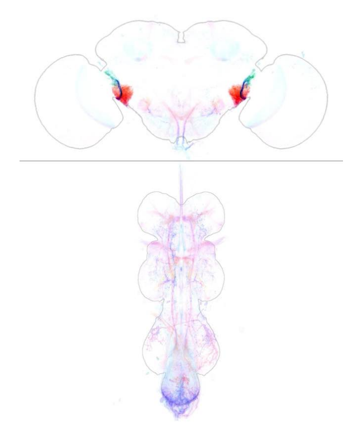
SS45492

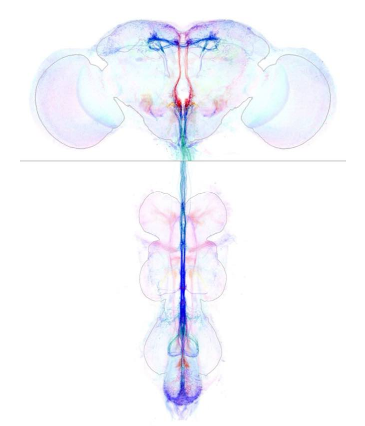
SS45494

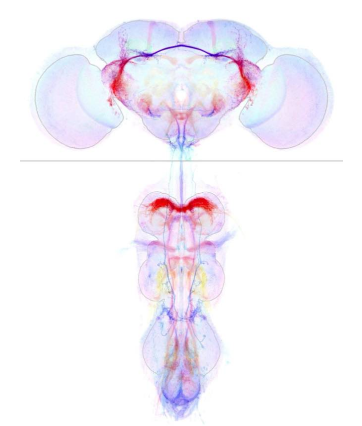
SS45507

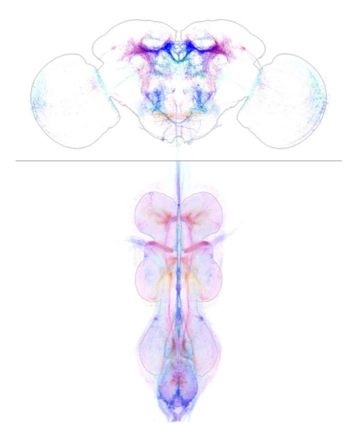
SS45510

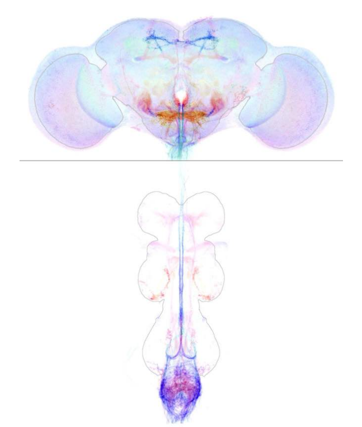
SS45512

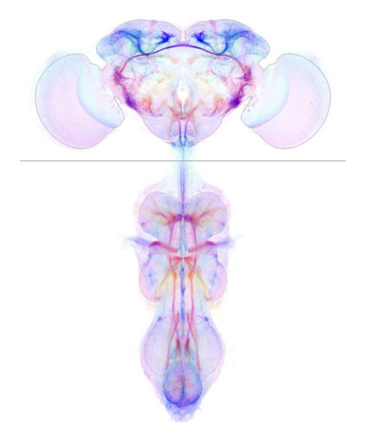
SS45514

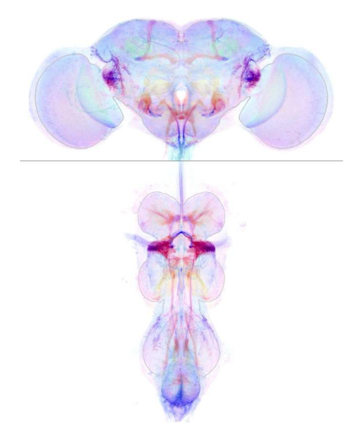
SS45515

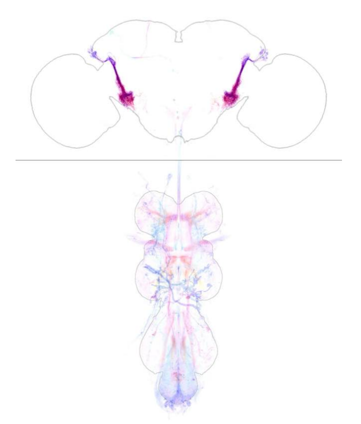
SS45516

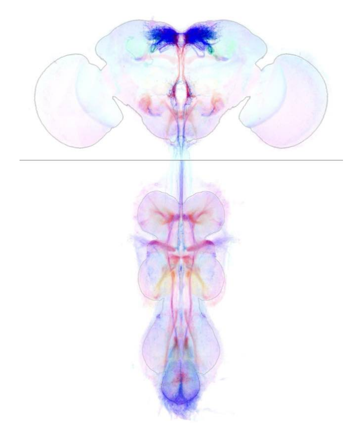
SS45518

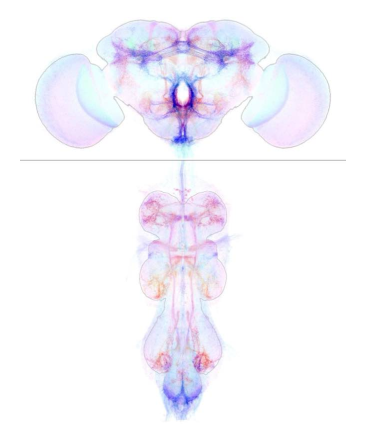
SS45542

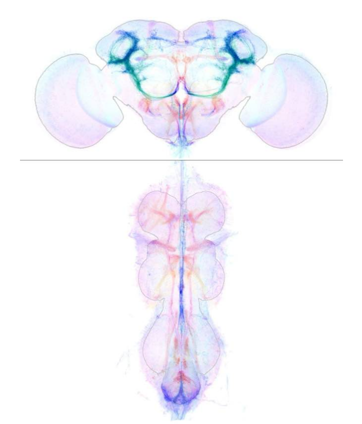
SS45560

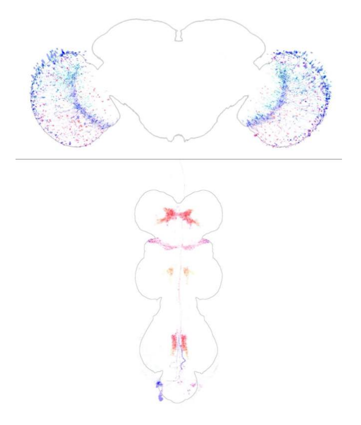
SS45605

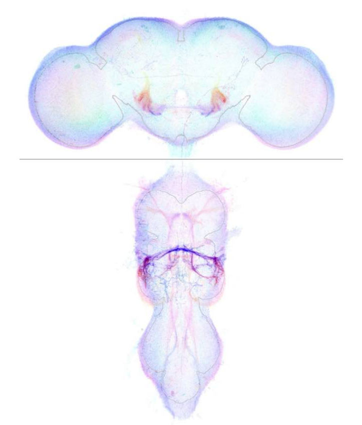
SS45607

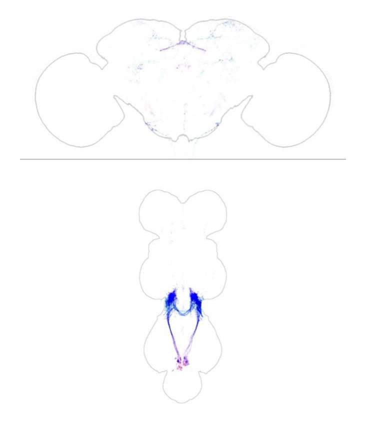
SS45611

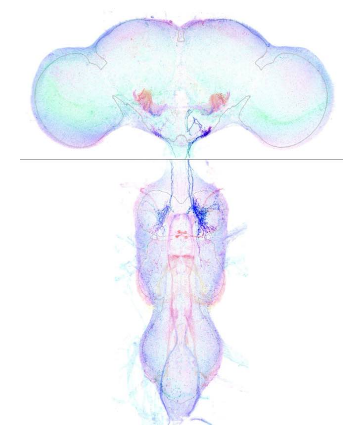
SS45635

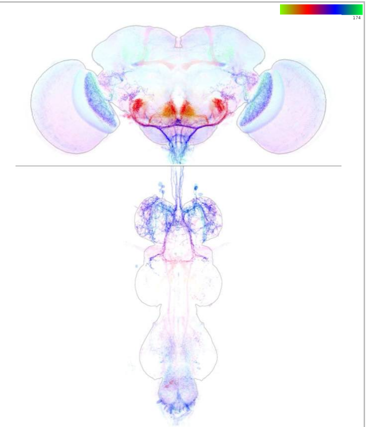

SS45680

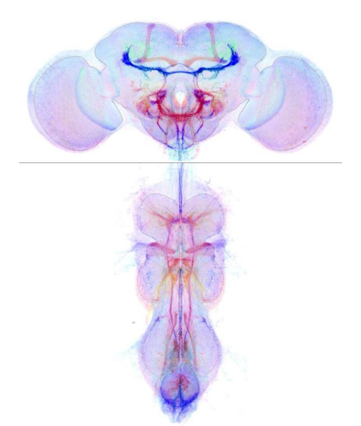

SS45693

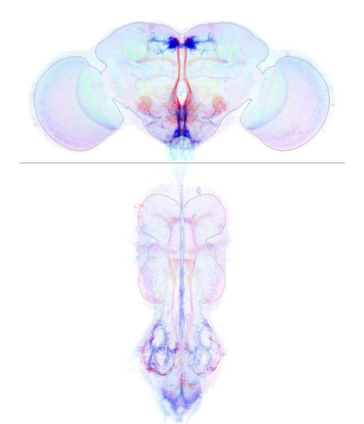

SS45730

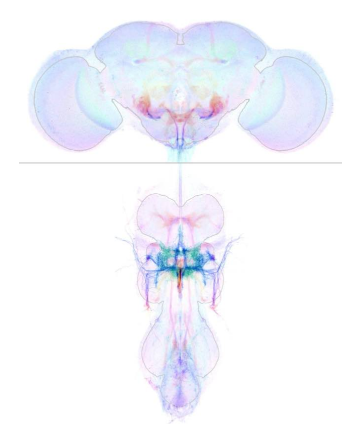

SS45766

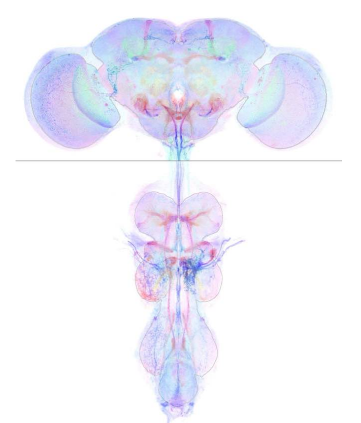

SS45772

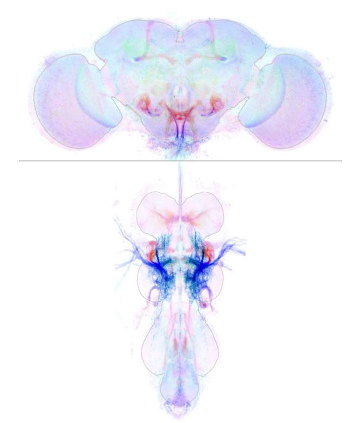

SS45779

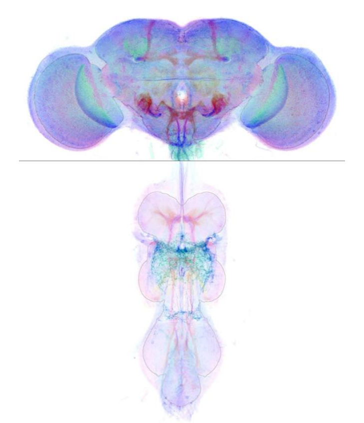

SS45780

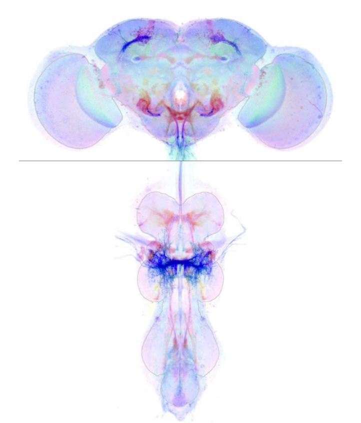

SS45782

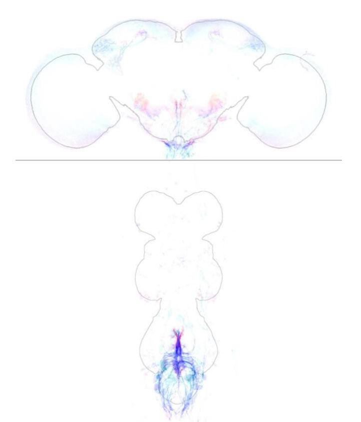

SS45805

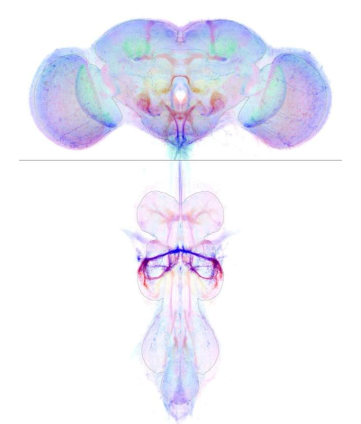

SS45808

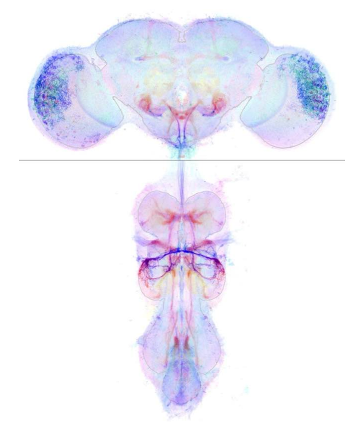

SS45828

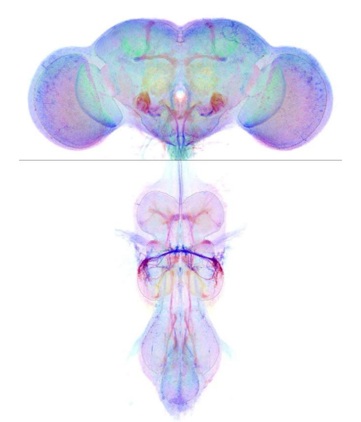

SS45829

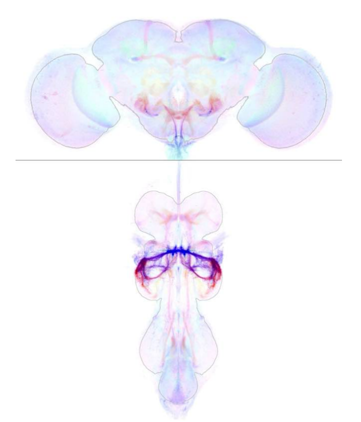

SS45830

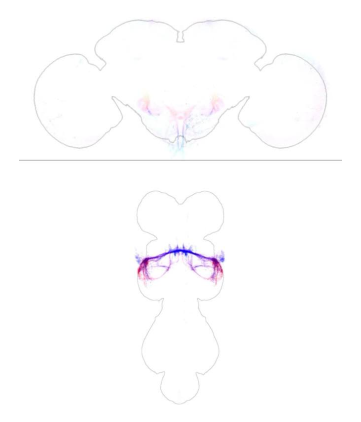

SS45843

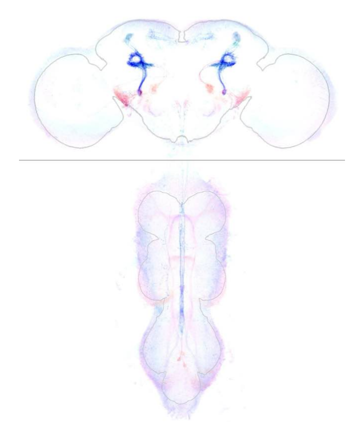

SS45858

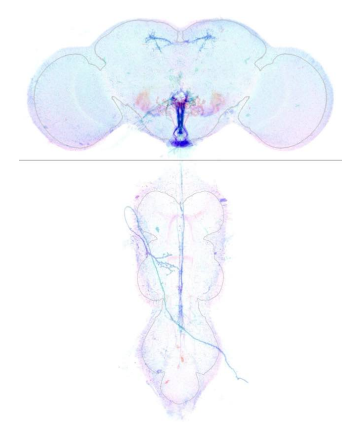

SS45898

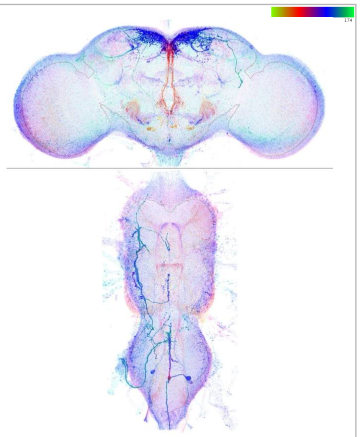
SS45915

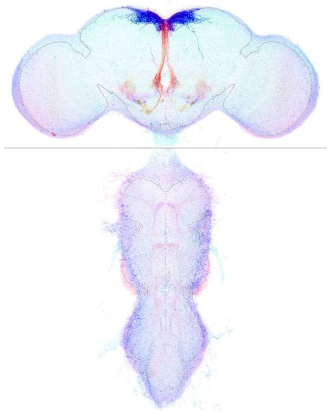
SS45921

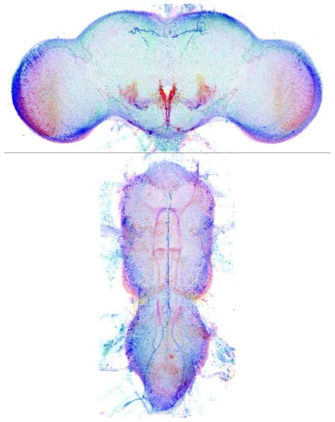
SS45941

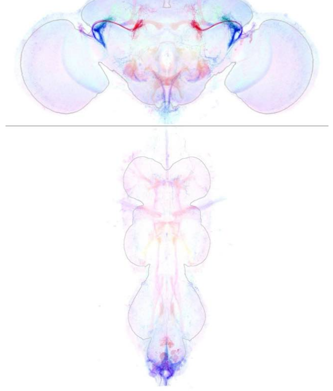
SS45967

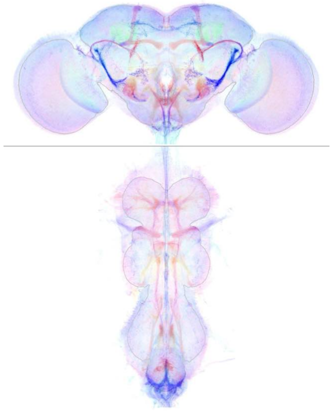
SS45972

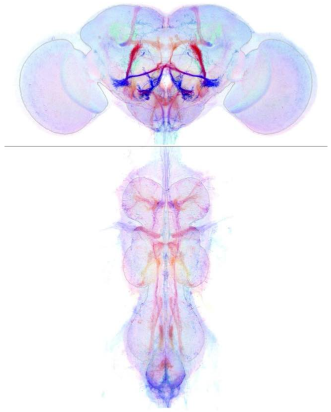
SS45987

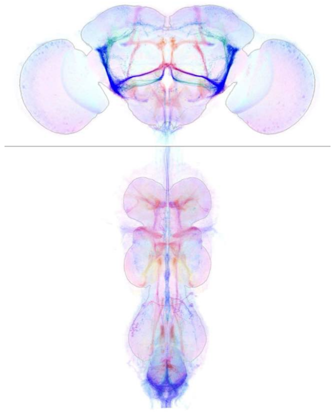
SS45988

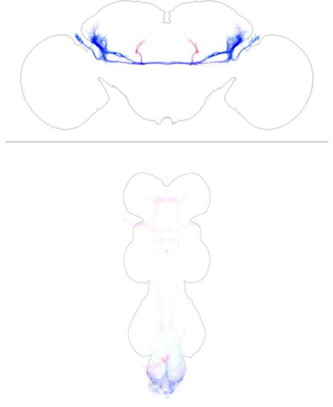
SS45993

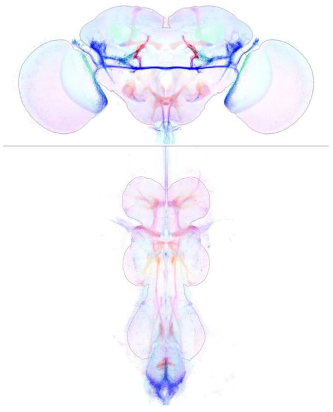
SS45998

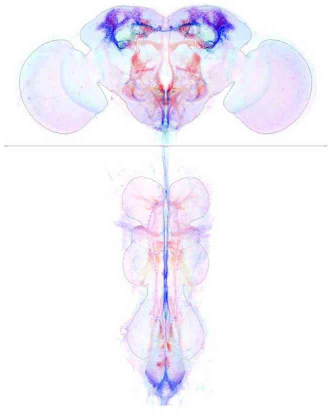
SS46011

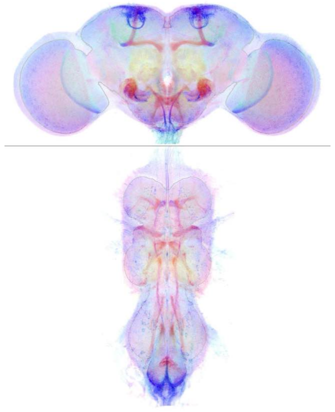
SS46017

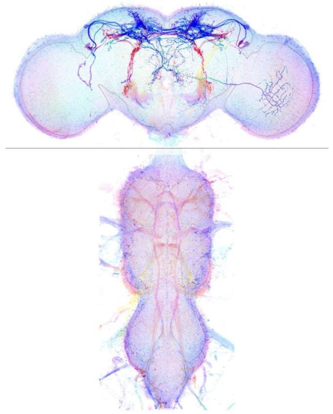
SS46056

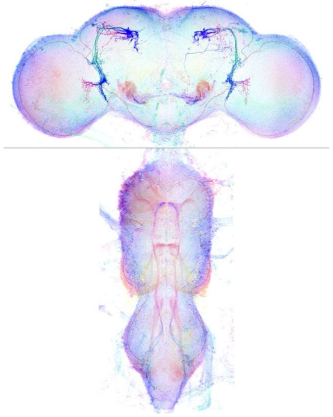
SS46059

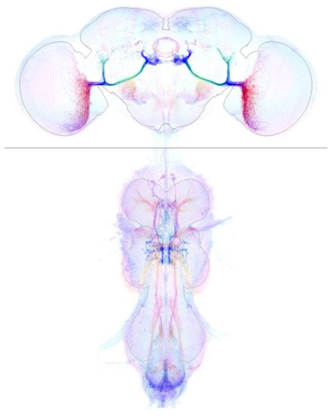
SS46079

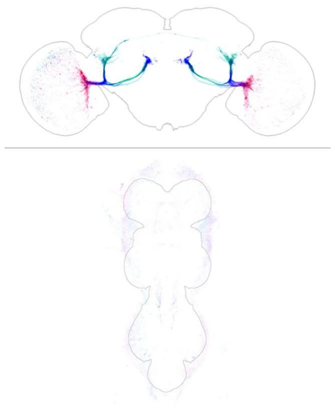
SS46080

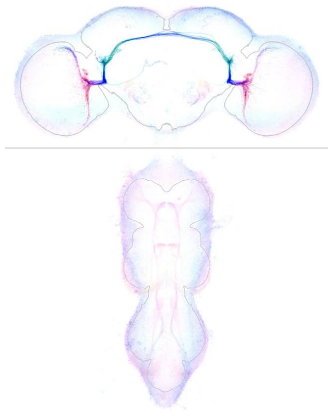
SS46115

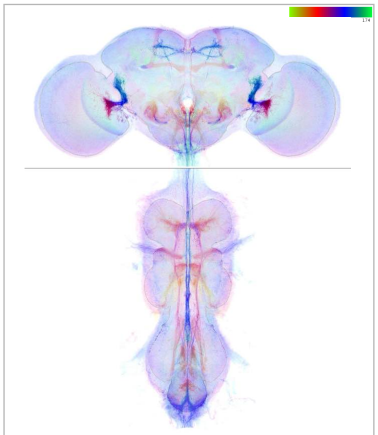

SS46135

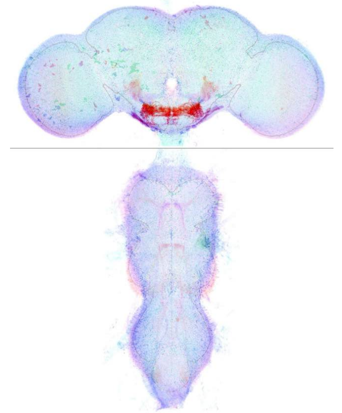

SS46204

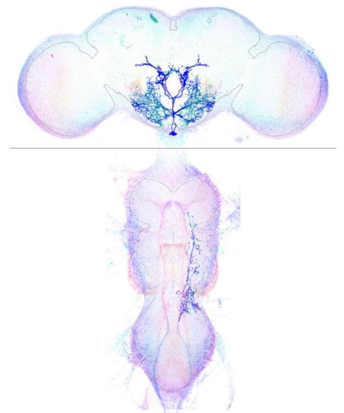

SS46211

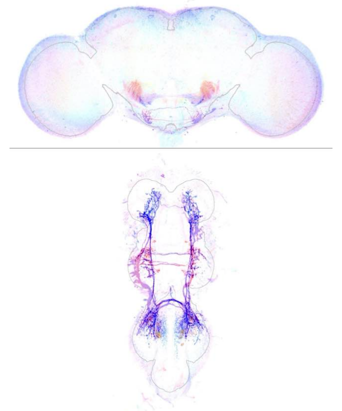

SS46225

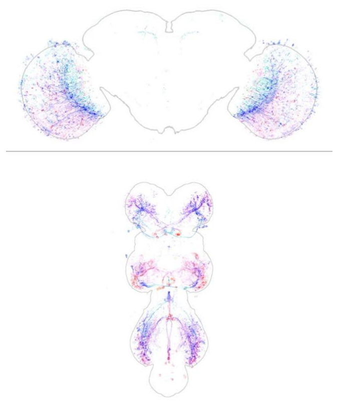

SS46229

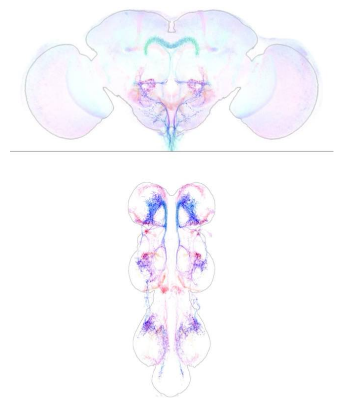

SS46233

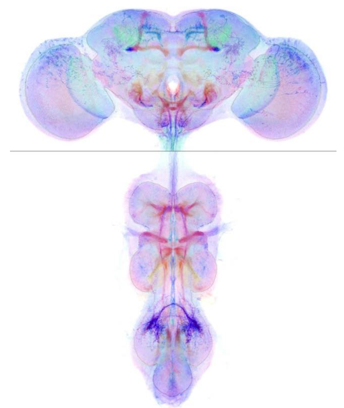

SS46243

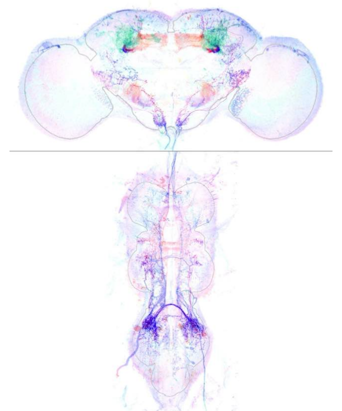

SS46253

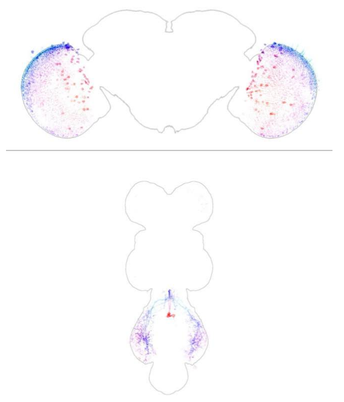

SS46258

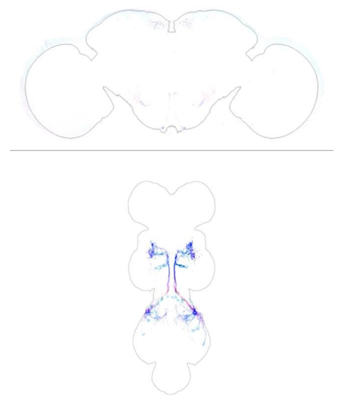

SS46260

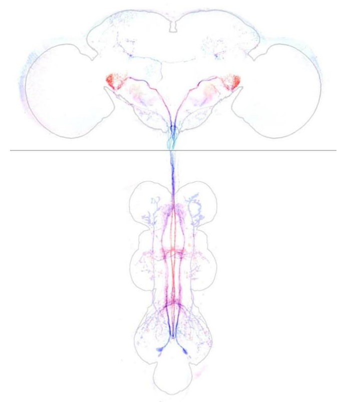

SS46285

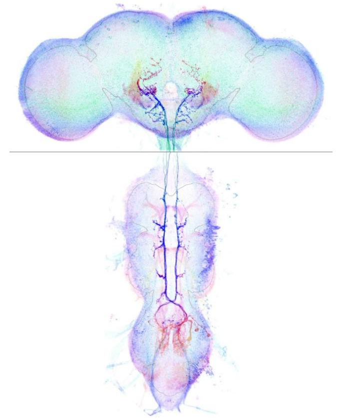

SS46290

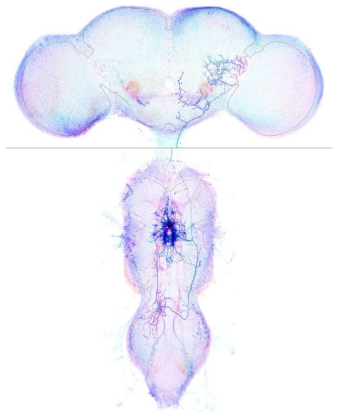

SS46295

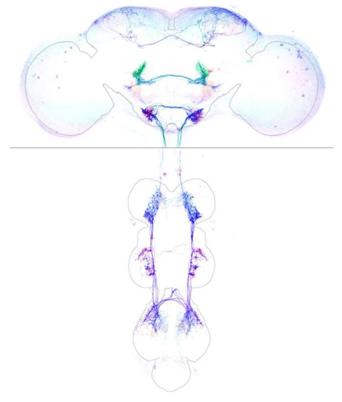

SS46300

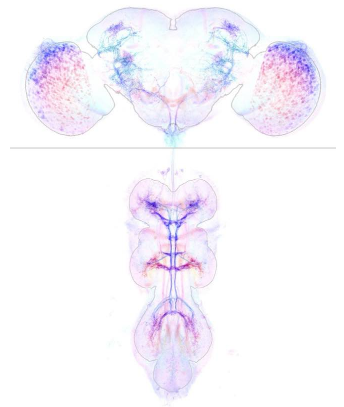

SS46301

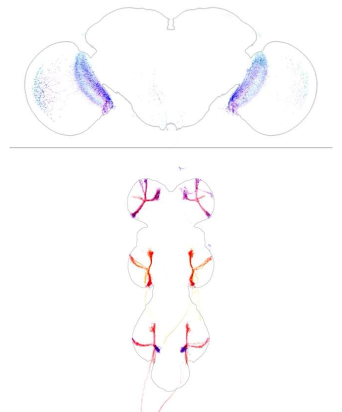

SS46314

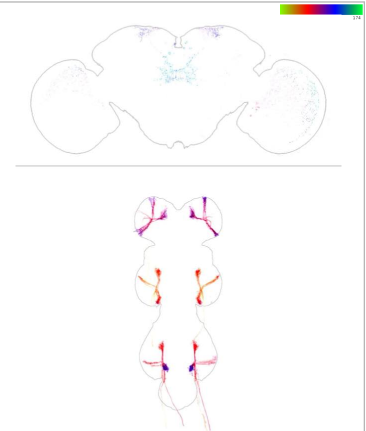
SS46317

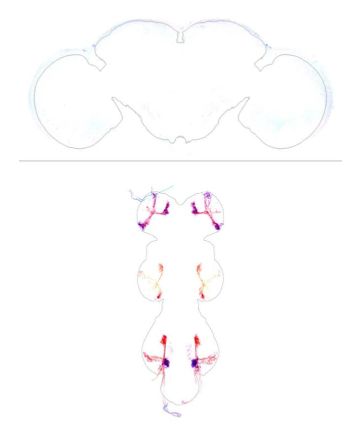
SS46318

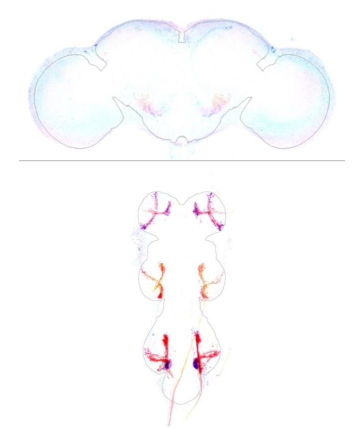
SS46319

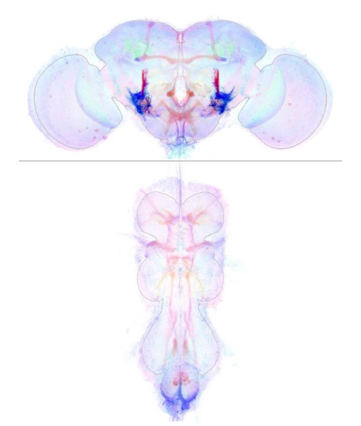
SS46330

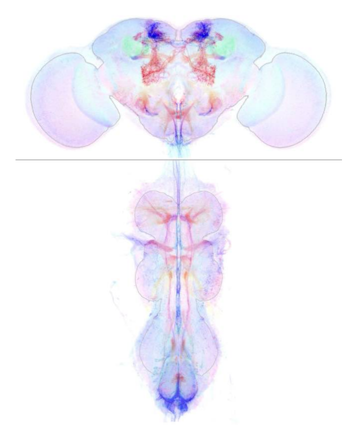
SS46337

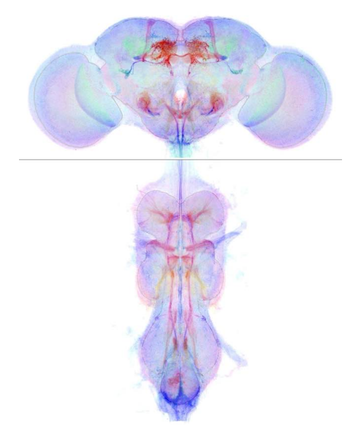
SS46348

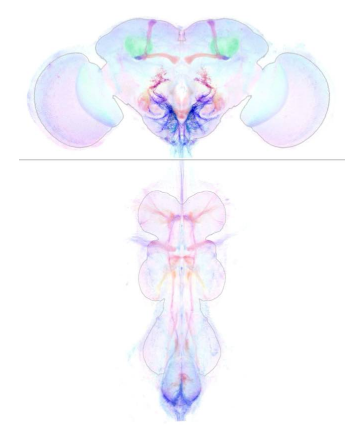
SS46367

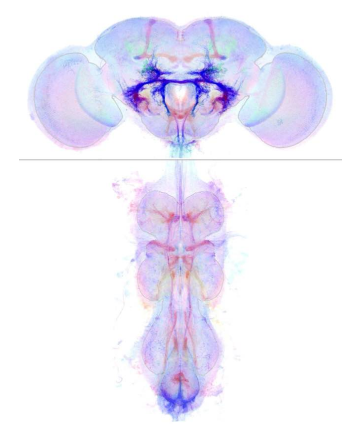
SS46377

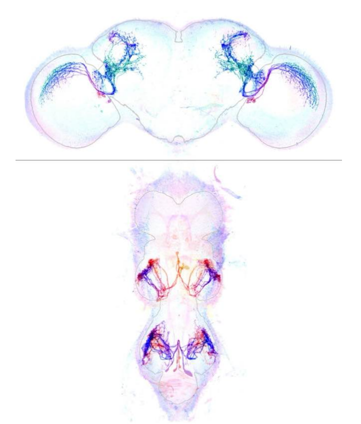
SS46391

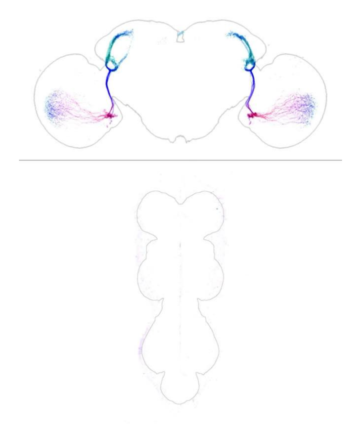
SS46404

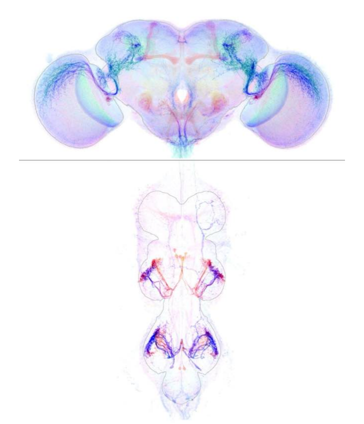
SS46439

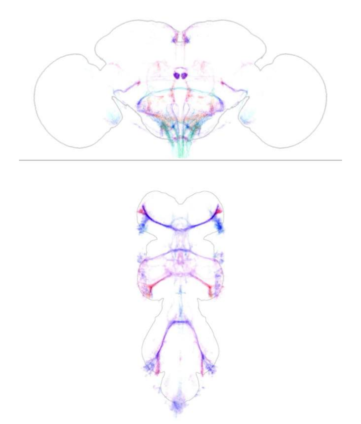
SS46507

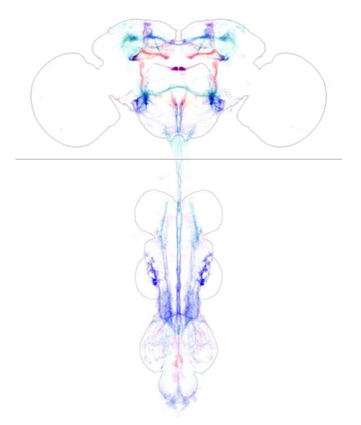
SS46512

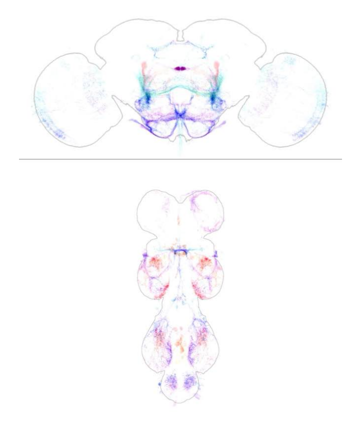
SS46515

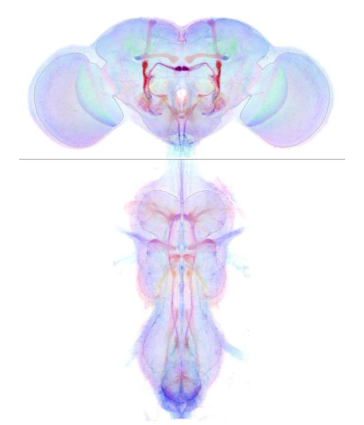
SS46517

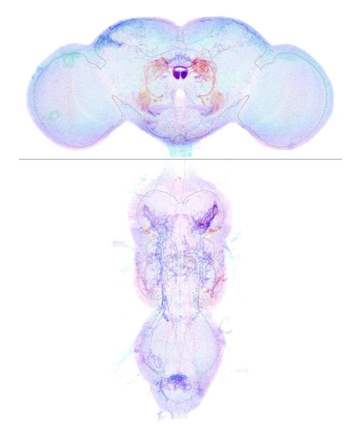
SS46525

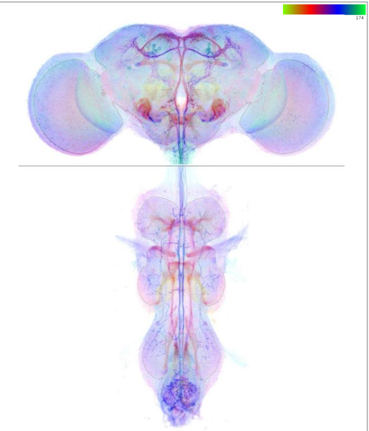
SS46540

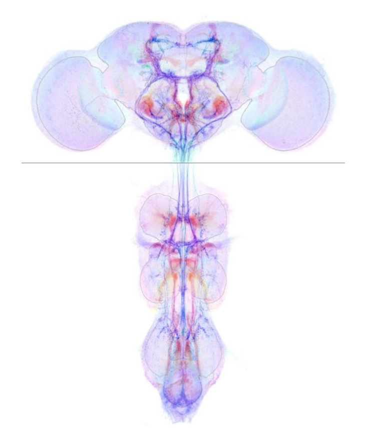
SS46542

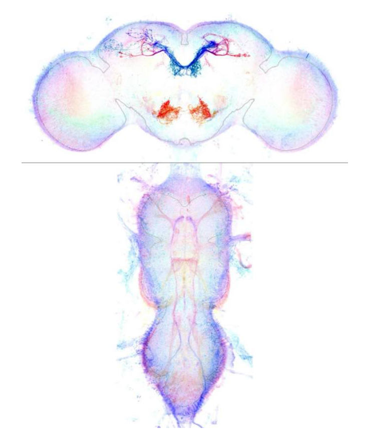
SS46560

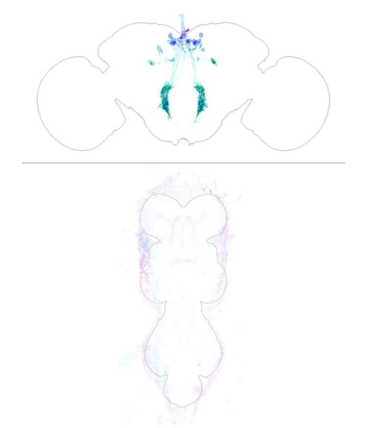
SS46563

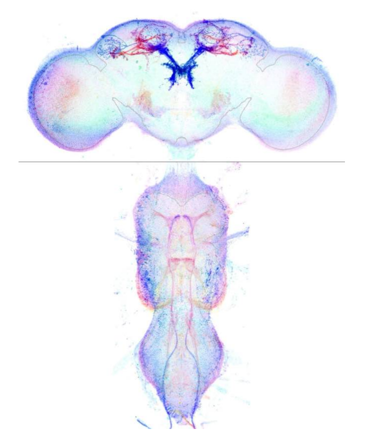
SS46572

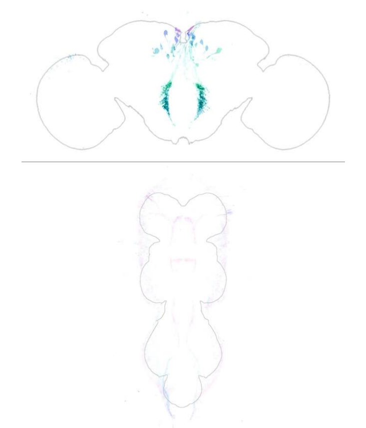
SS46577

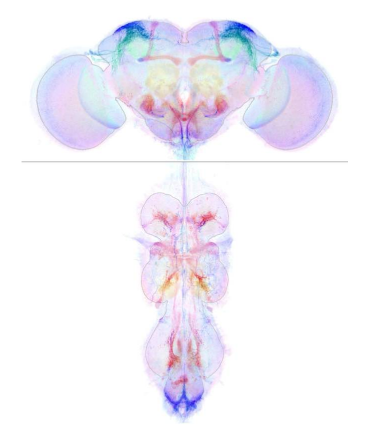
SS46589

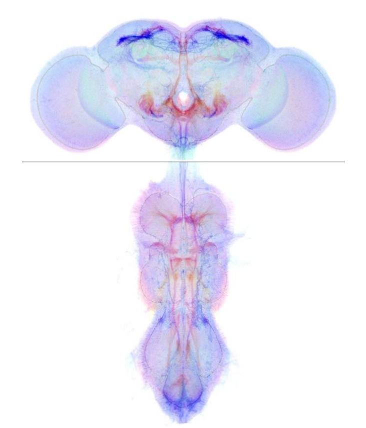
SS46596

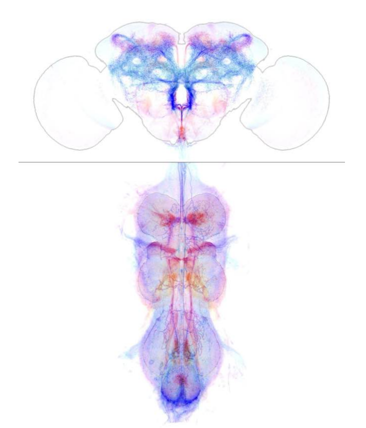
SS46603

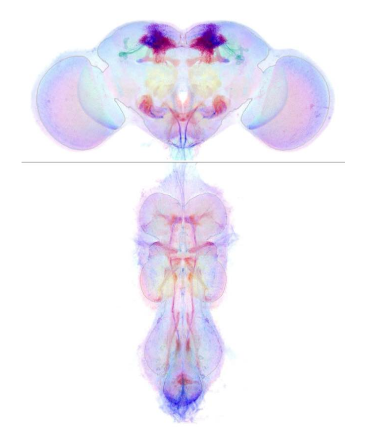
SS46613

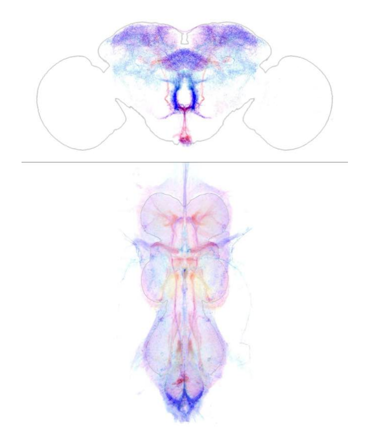
SS46630

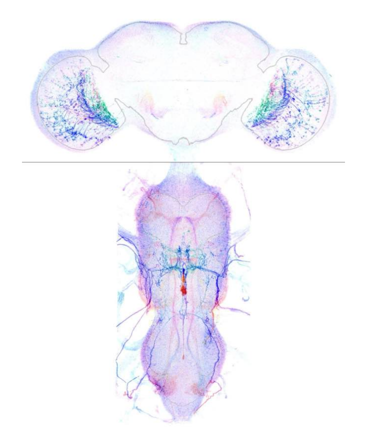
SS46645

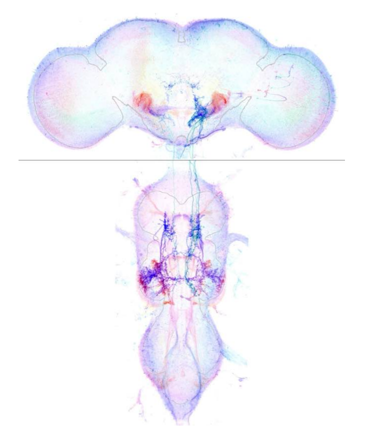
SS46669

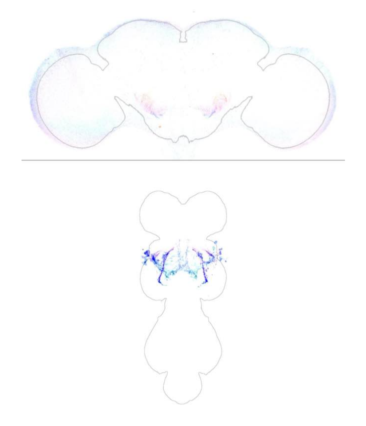
SS46675

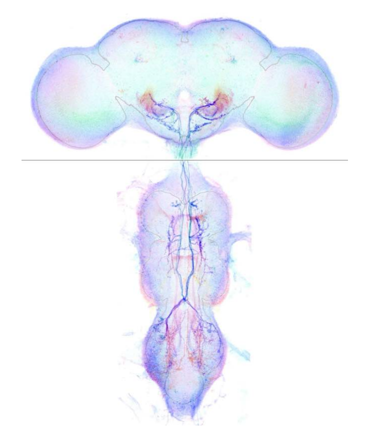
SS46696

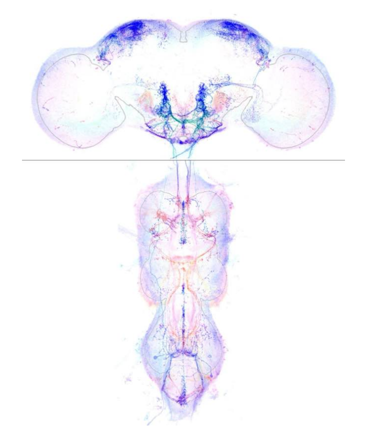
SS46697

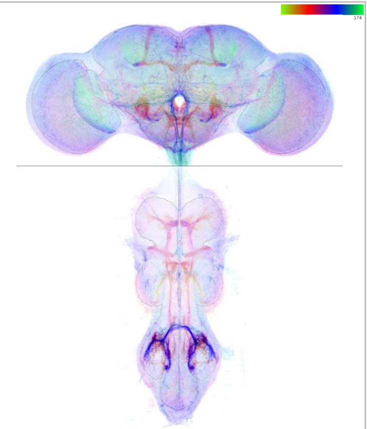
SS46706

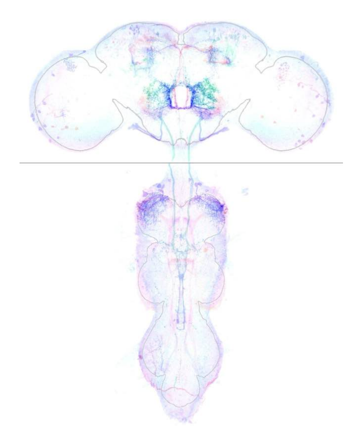
SS46716

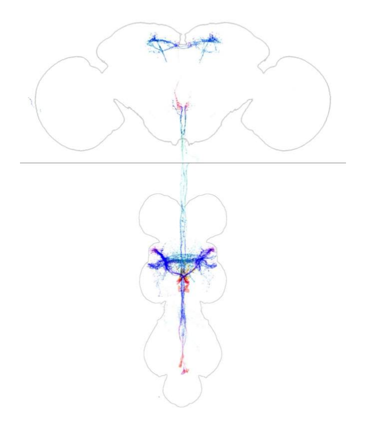
SS46725

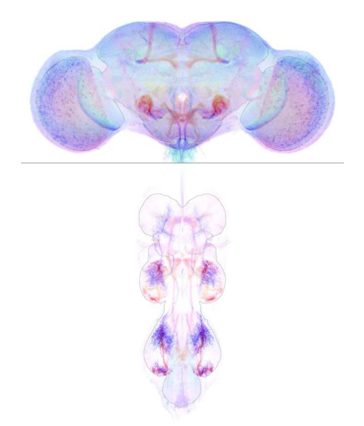
SS46730

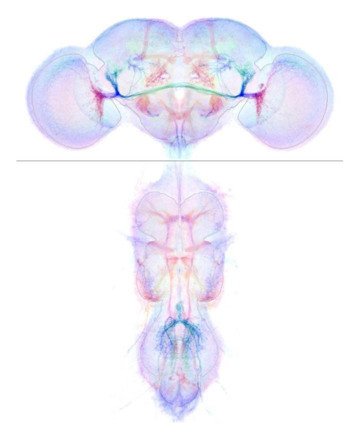
SS46747

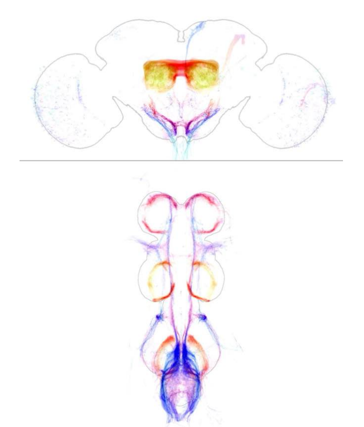
SS46798

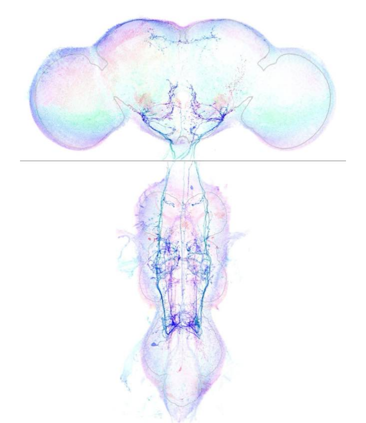
SS46847

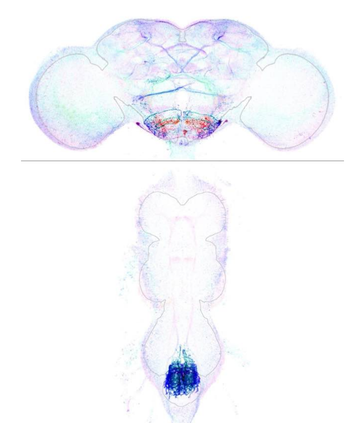
SS46885

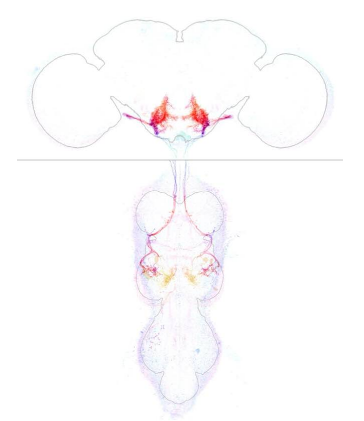
SS46913

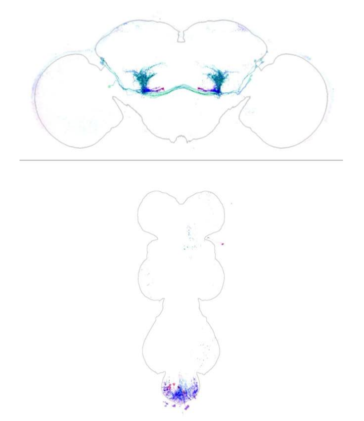
SS47009

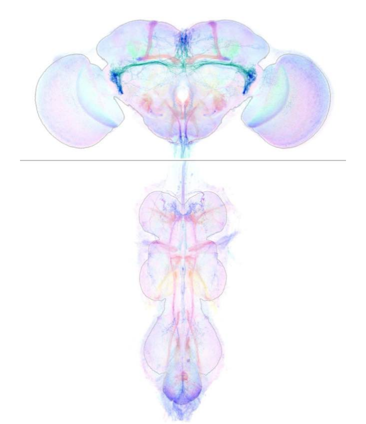
SS47091

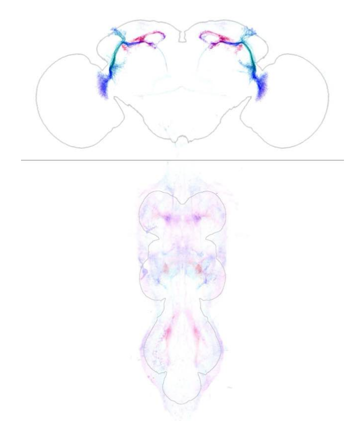
SS47104

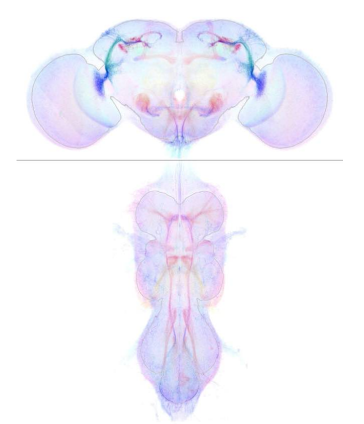
SS47105

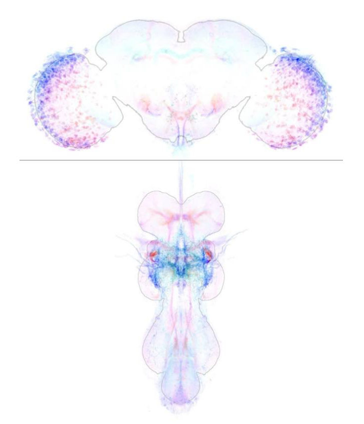
SS47120

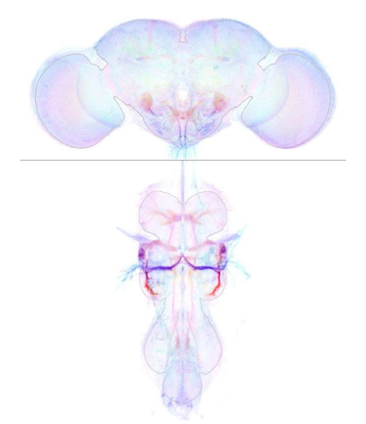
SS47125

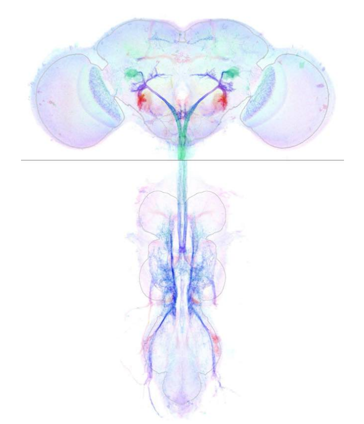
SS47152

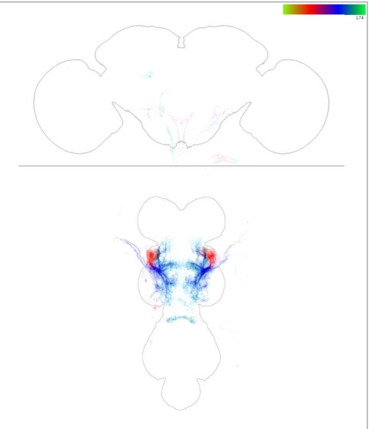

SS47160

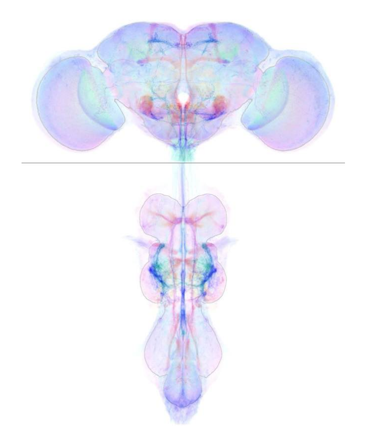

SS47192

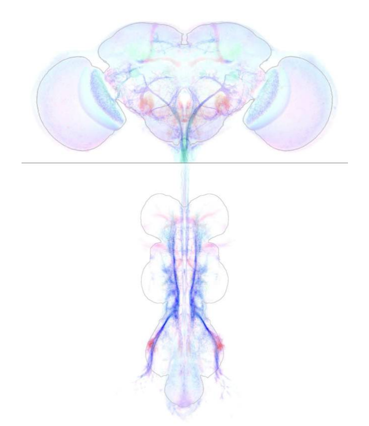

SS47195

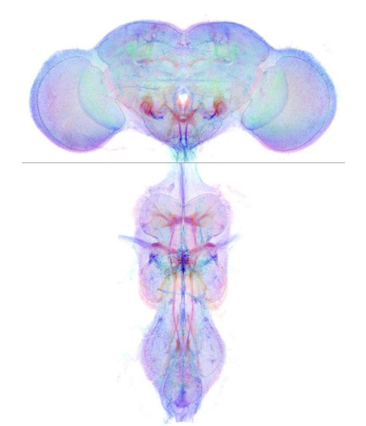

SS47200

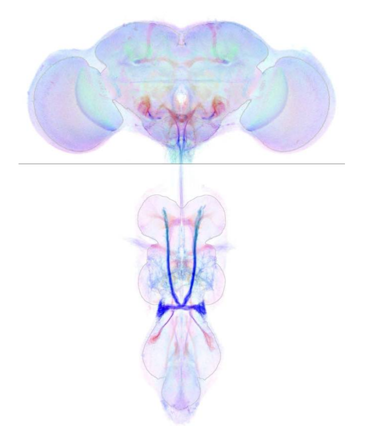

SS47204

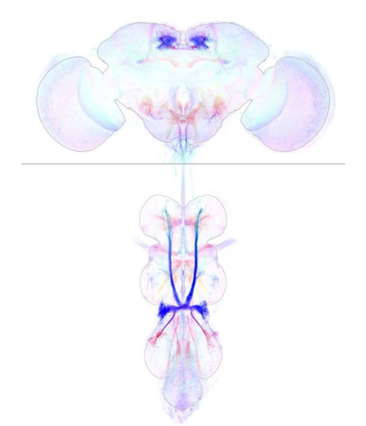

SS47214

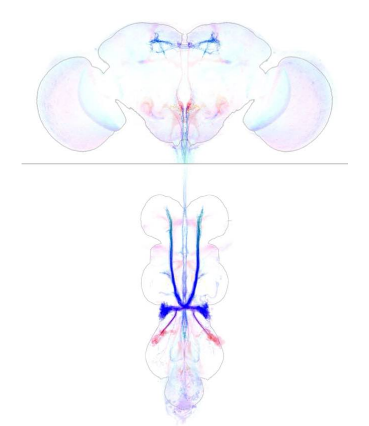

SS47215

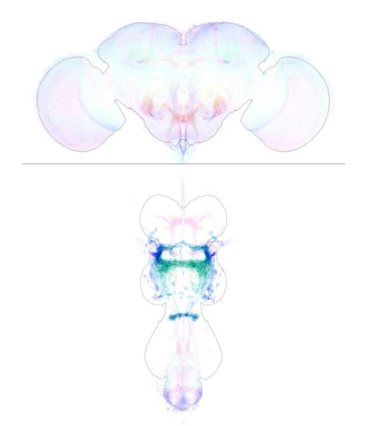

SS47219

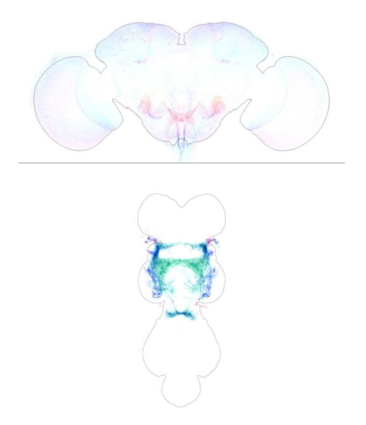

SS47222

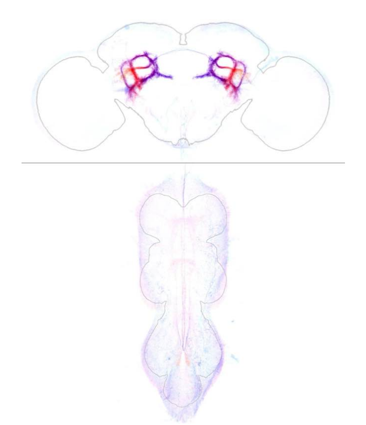

SS47261

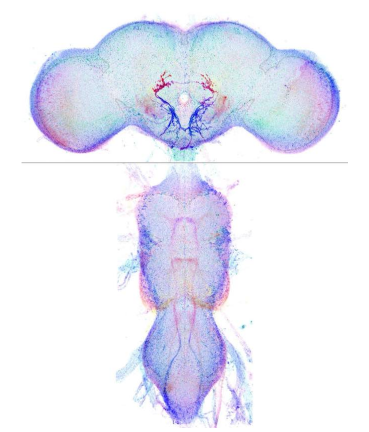

SS47292

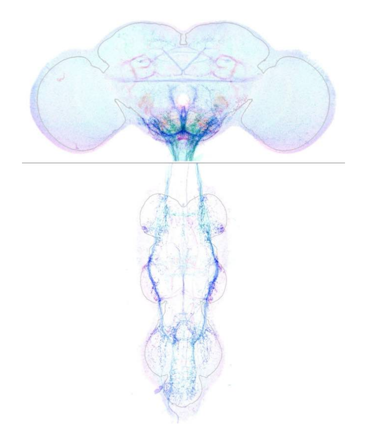

SS47305

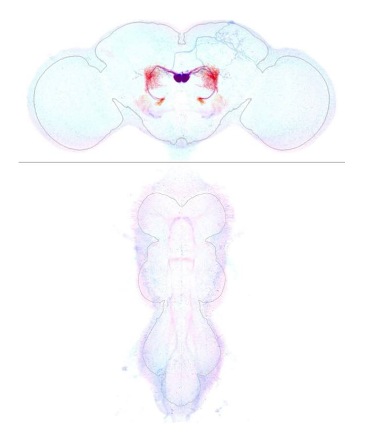

SS47384

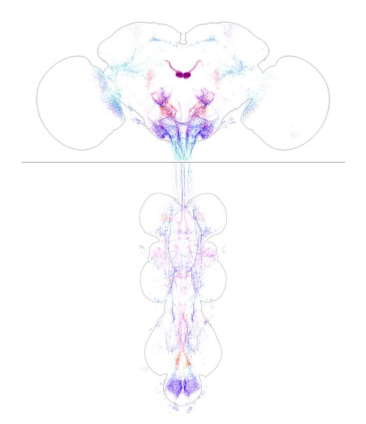

SS47398

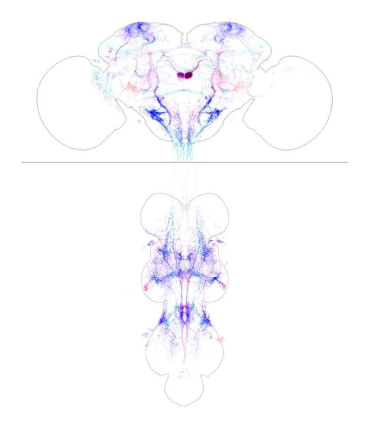

SS47405

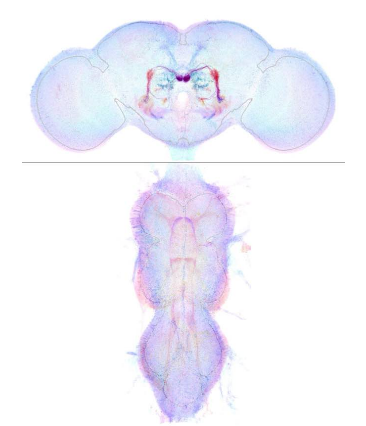

SS47406

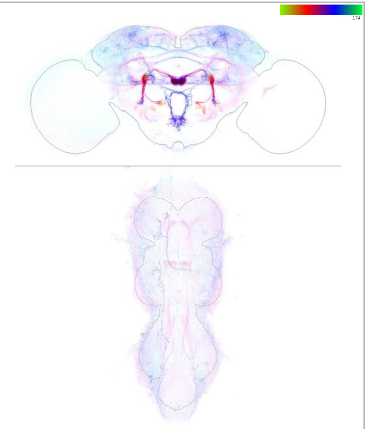
SS47436

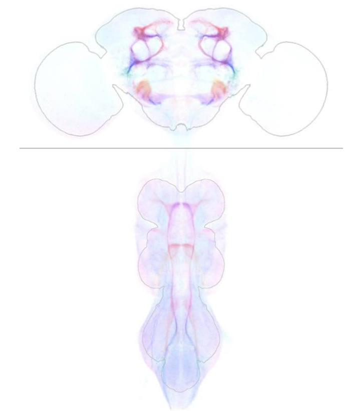
SS47478

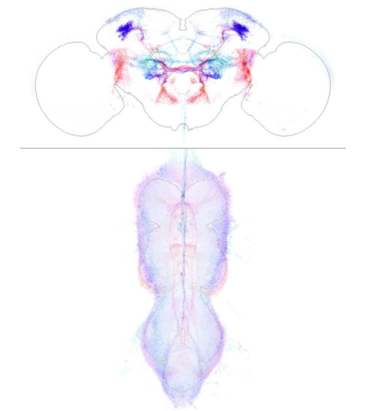
SS47509

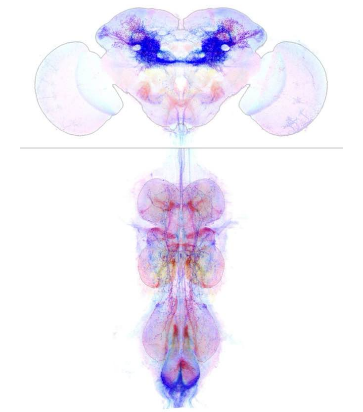
SS47554

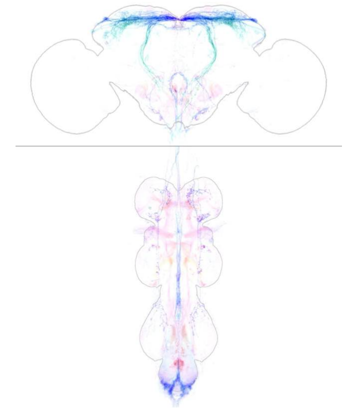
SS47557

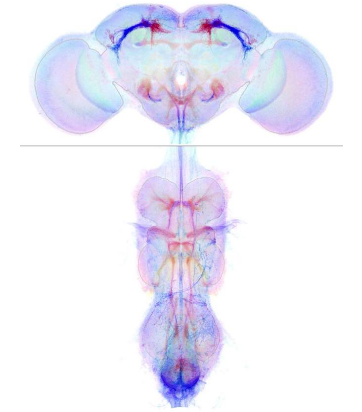
SS47562

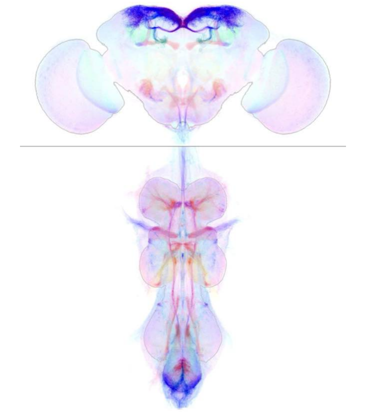
SS47578

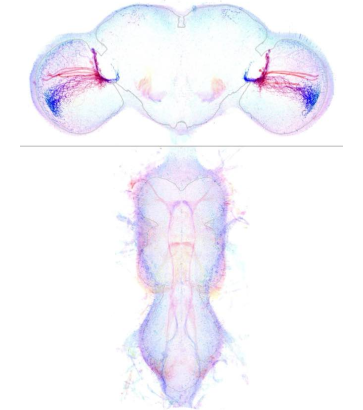
SS47622

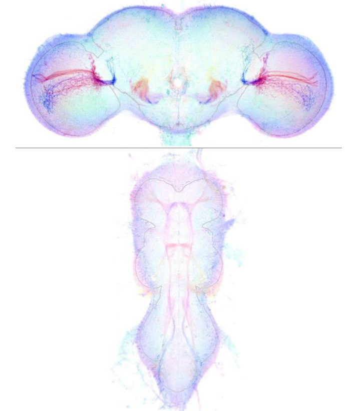
SS47629

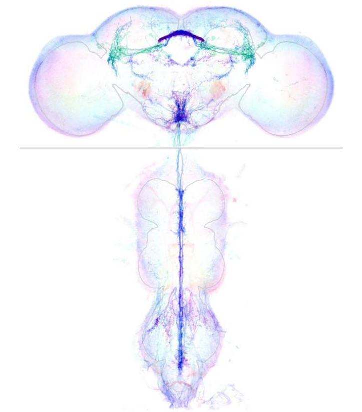
SS47631

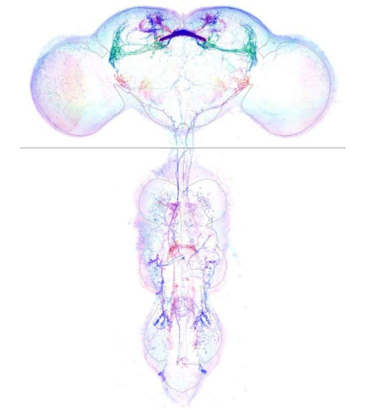
SS47633

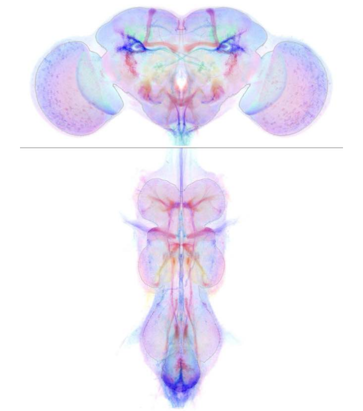
SS47815

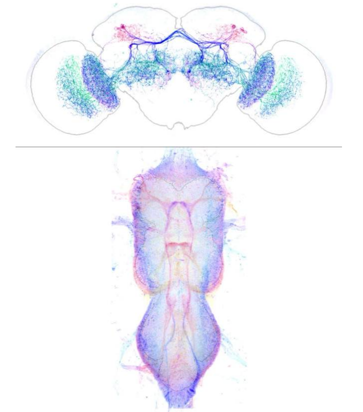
SS47816

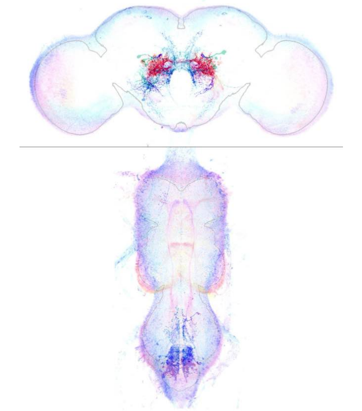
SS47827

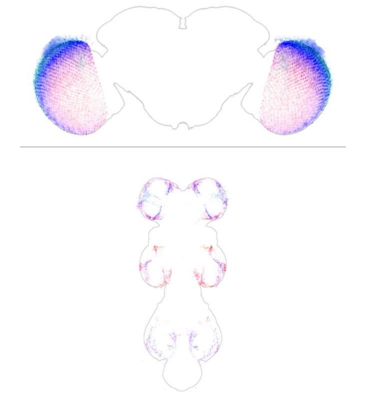
SS47833

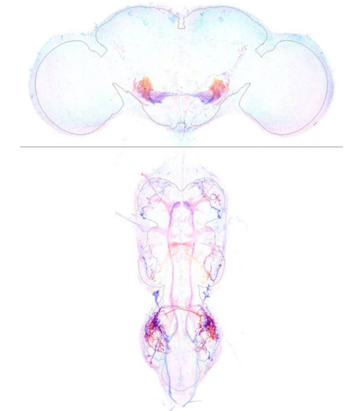
SS47847

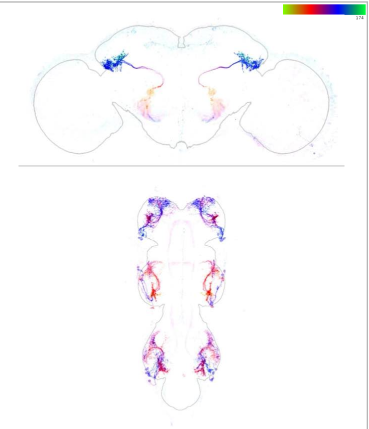

SS47863

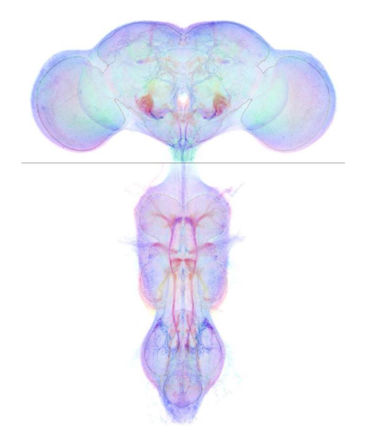

SS47877

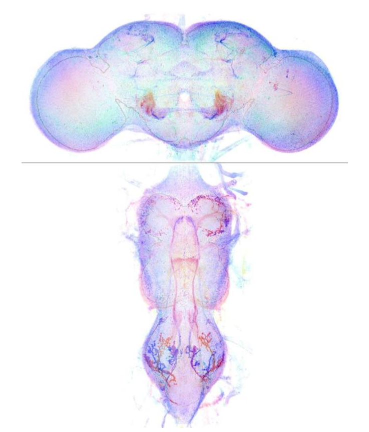

SS47885

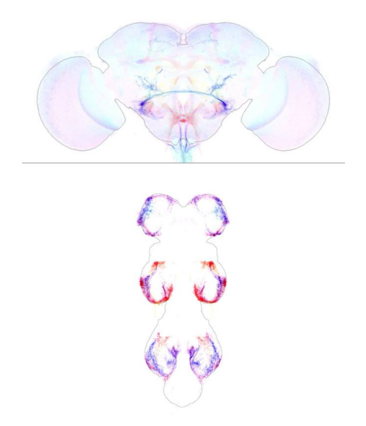

SS47886

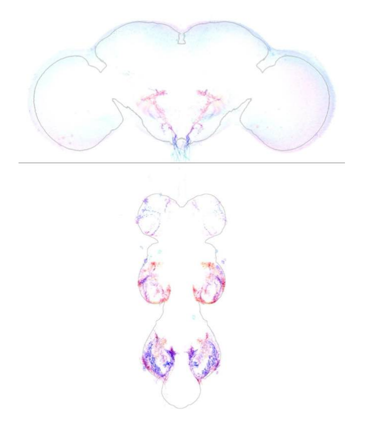

SS47887

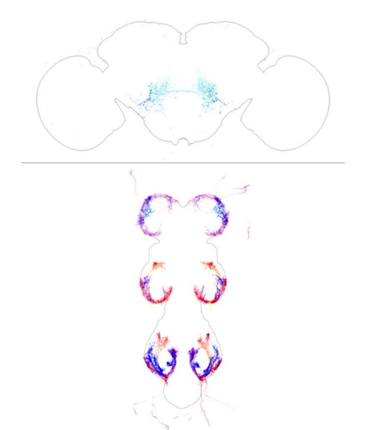

SS47888

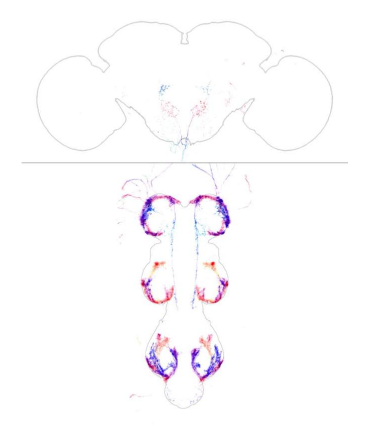

SS47889

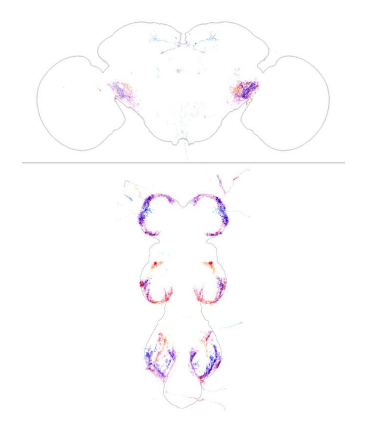

SS47890

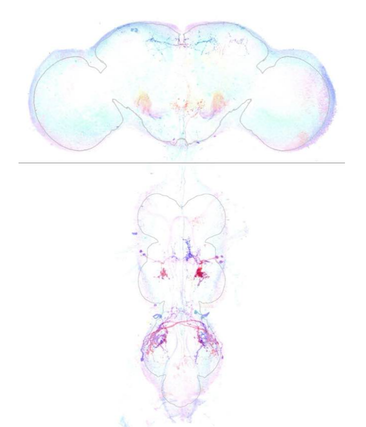

SS47914

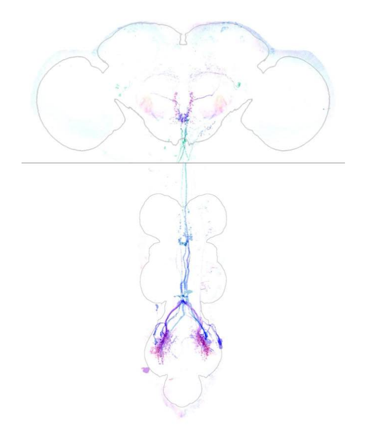

SS47918

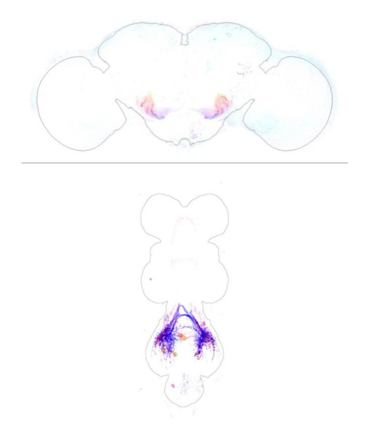

SS47921

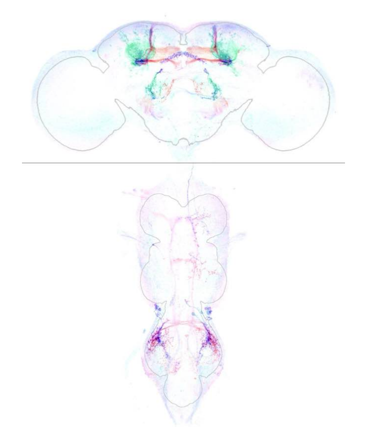

SS47929

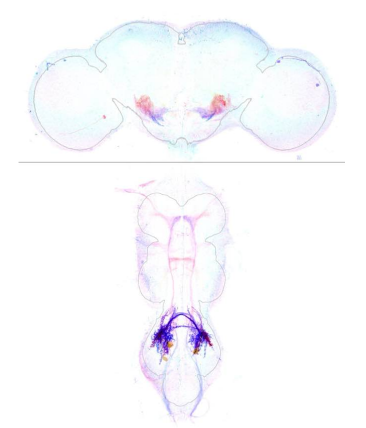

SS47938

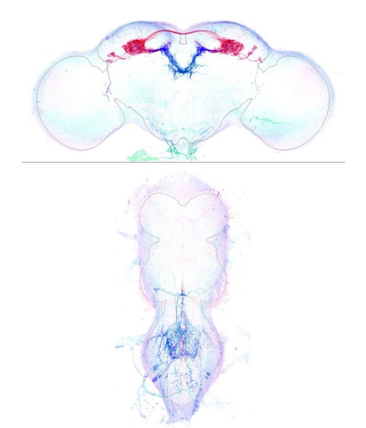

SS48157

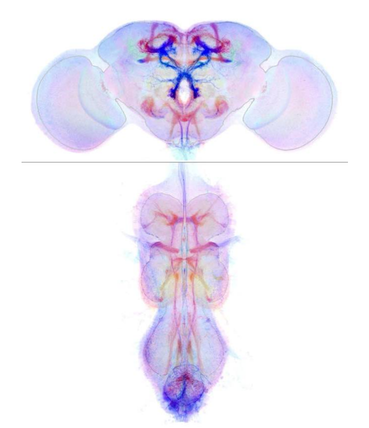

SS48192

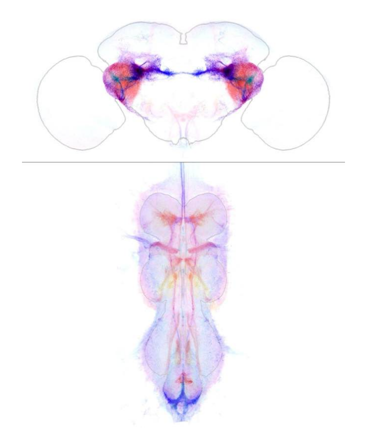

SS48194

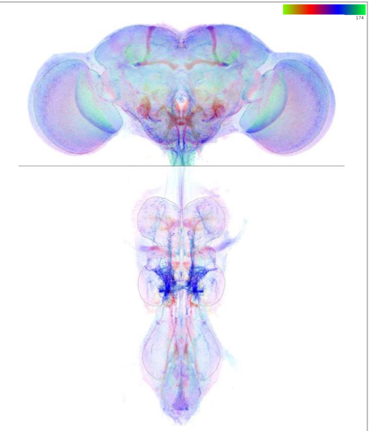

SS48204

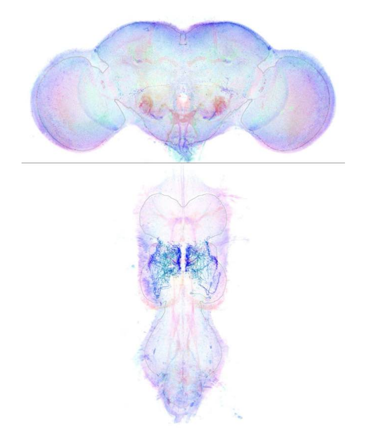

SS48215

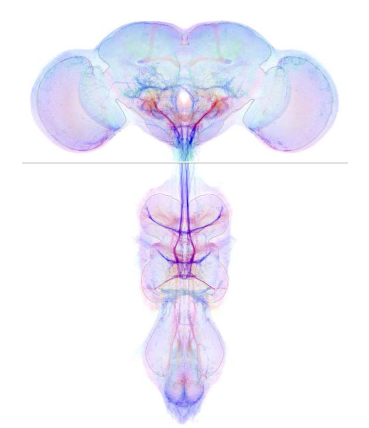

SS48221

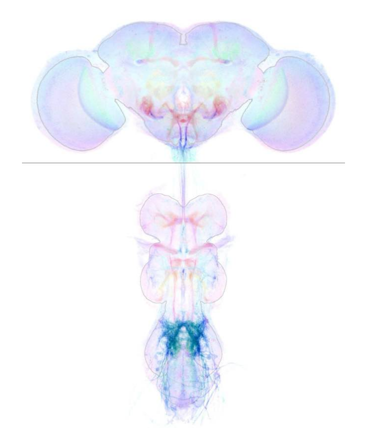

SS48240

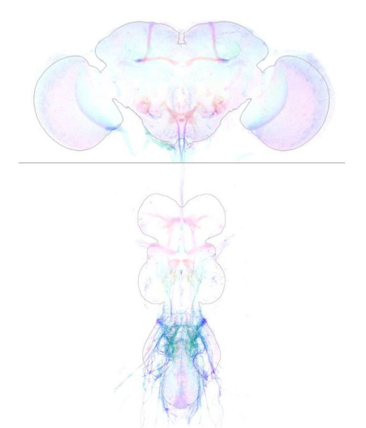

SS48247

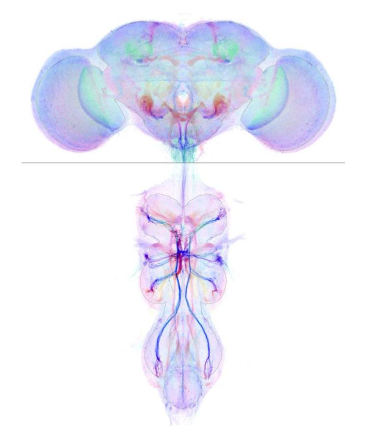

SS48262

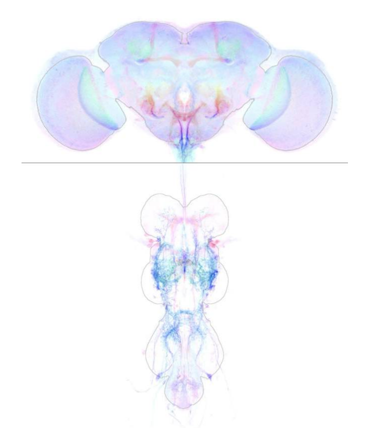

SS48268

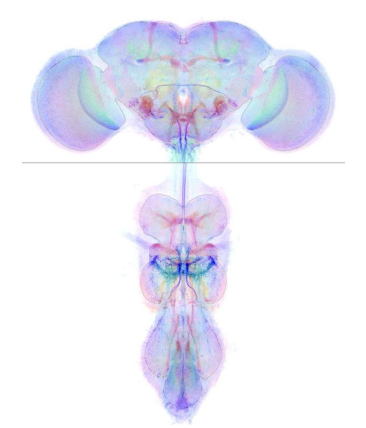

SS48272

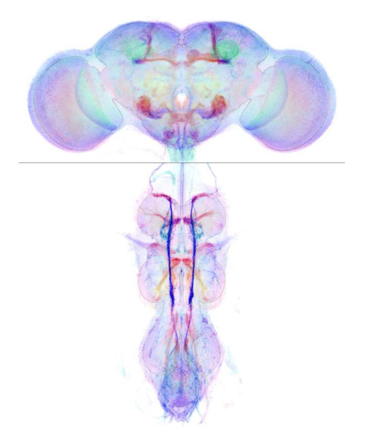

SS48287

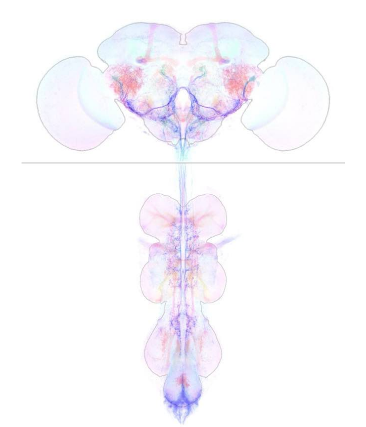

SS48301

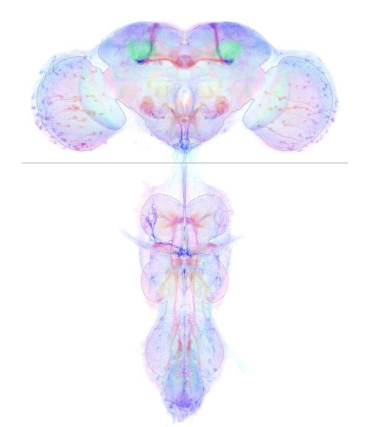

SS48305

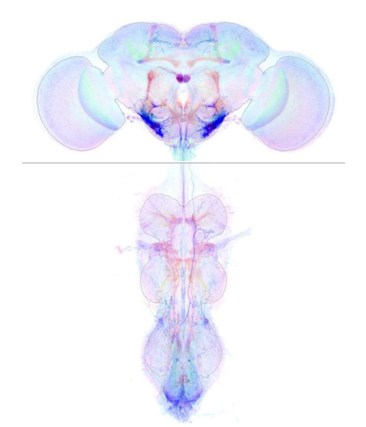

SS48335

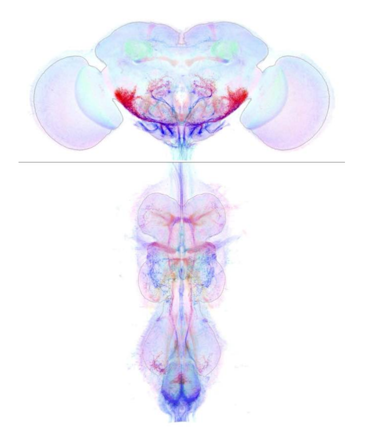

SS48336

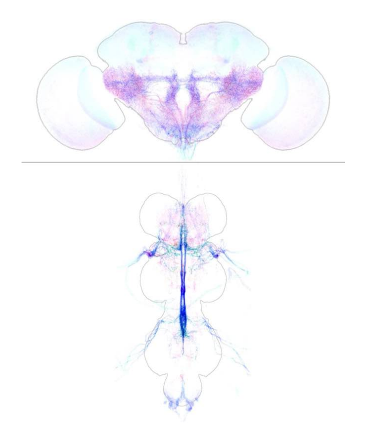

SS48339

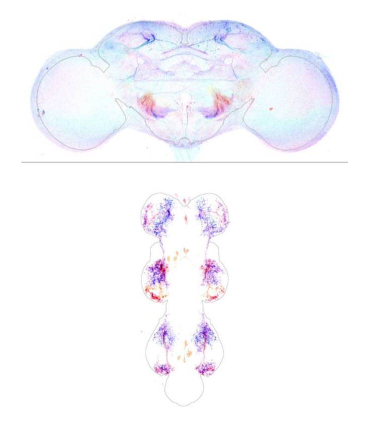

SS48376

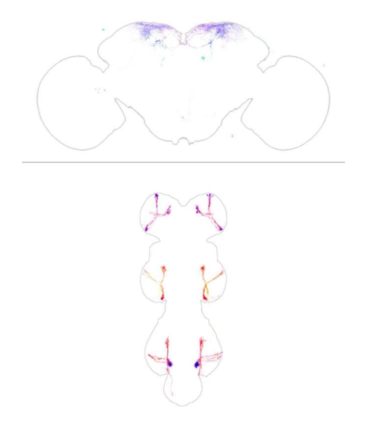

SS48402

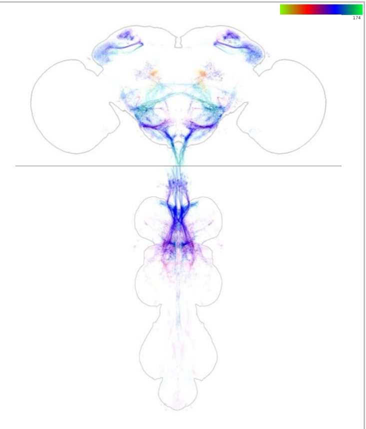
SS48406
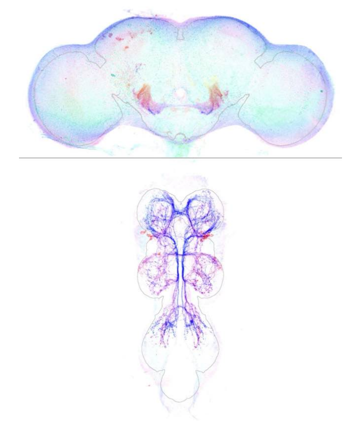
SS48413
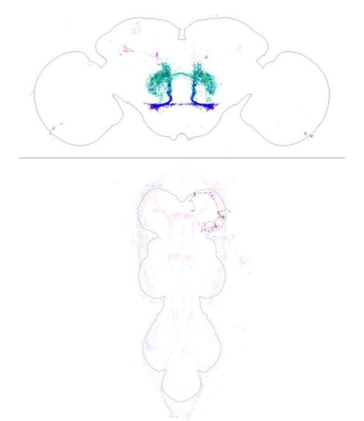
SS48416
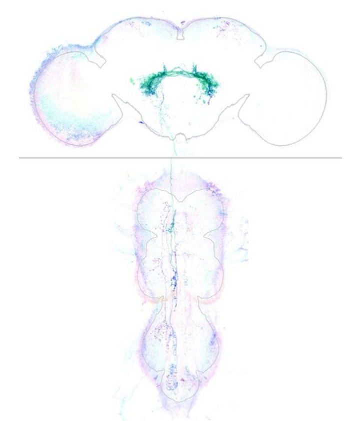
SS48427
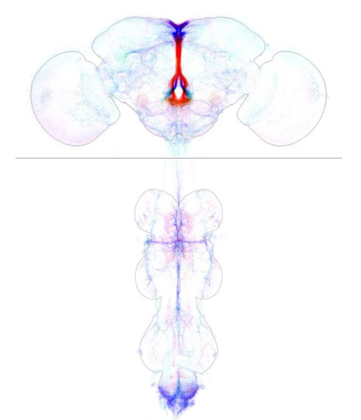
SS48598
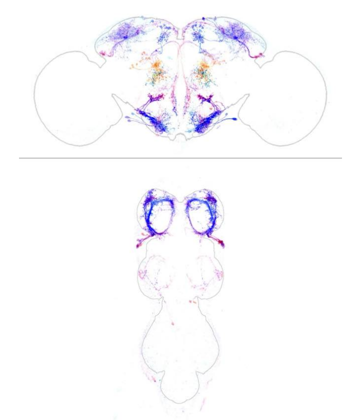
SS48616
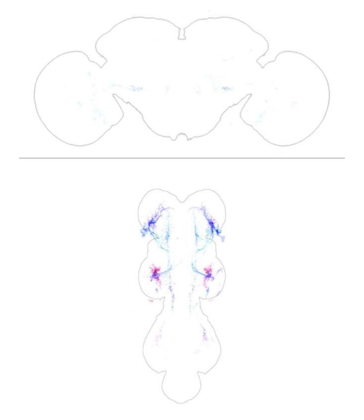
SS48619
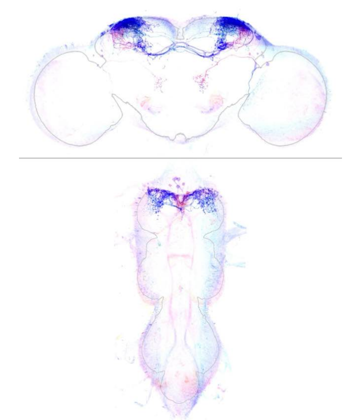
SS48635
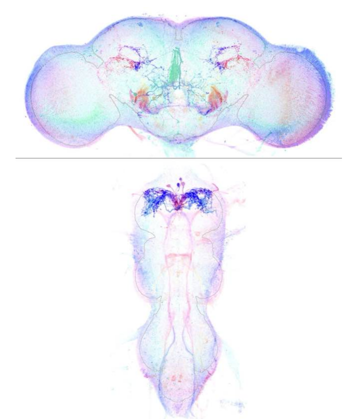
SS48636
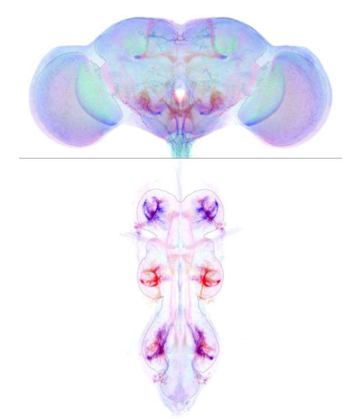
SS48638
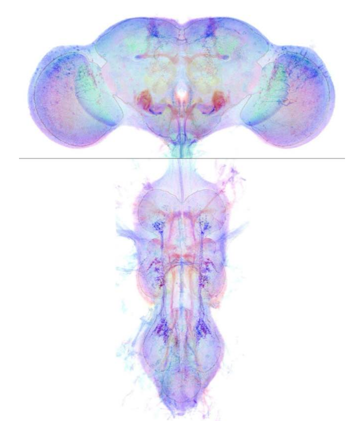
SS48644
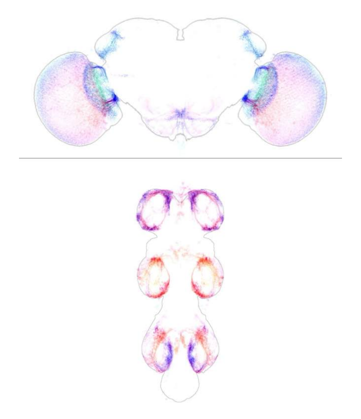
SS48648
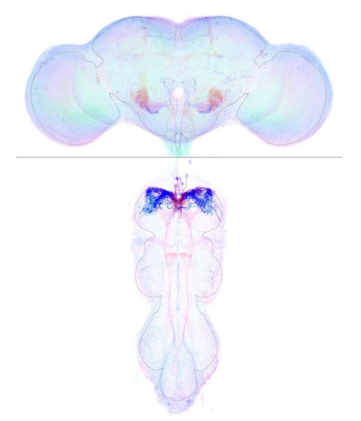
SS48649
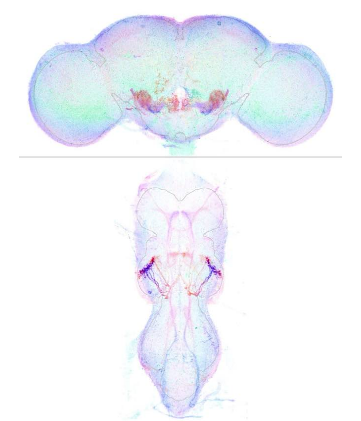
SS48678
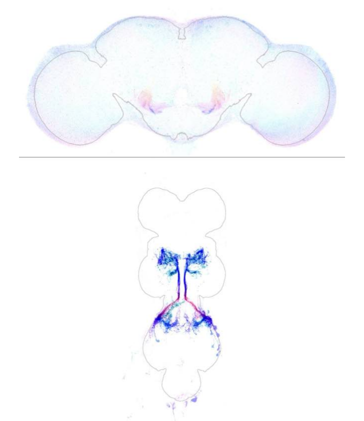
SS48709
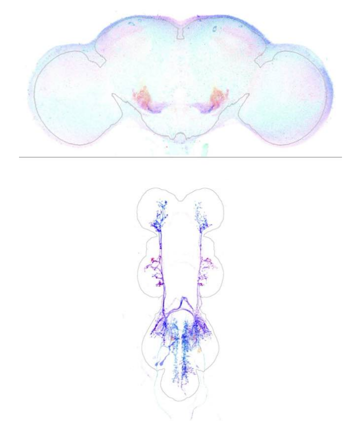
SS48718

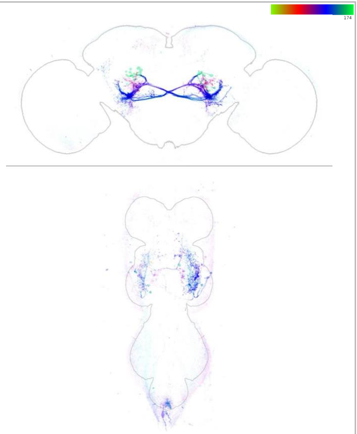
SS48727

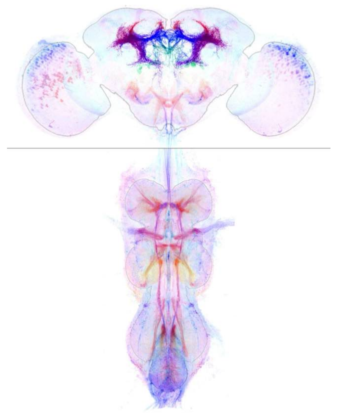
SS48743

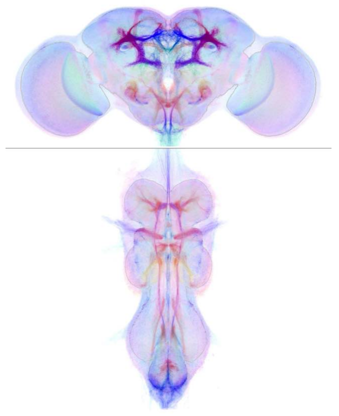
SS48747

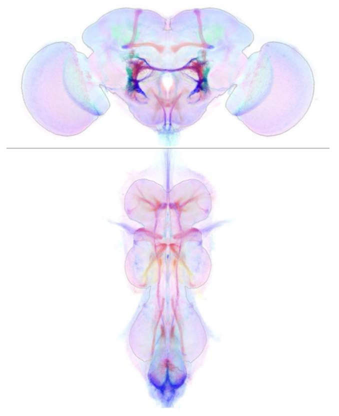
SS48753

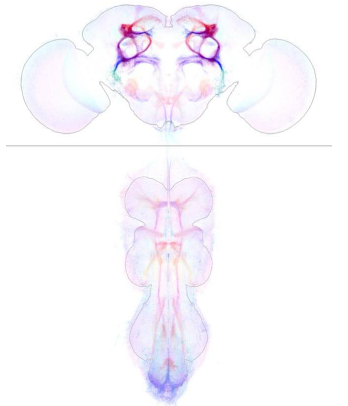
SS48777

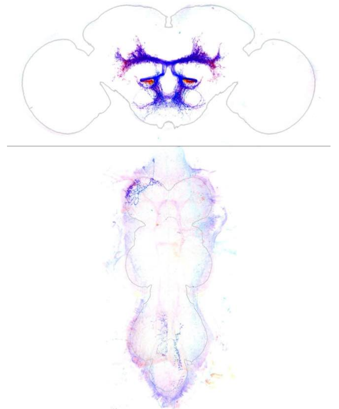
SS48784

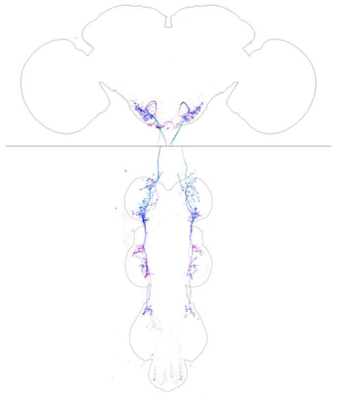
SS48787

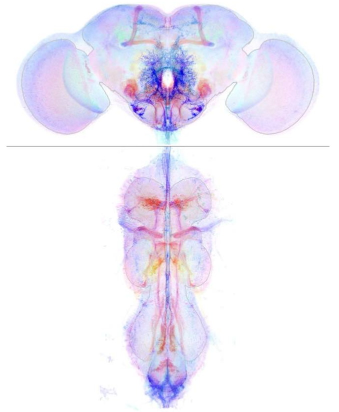
SS48788

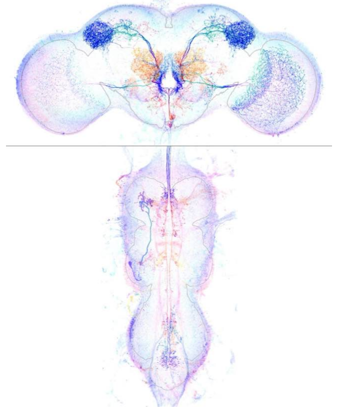
SS48794

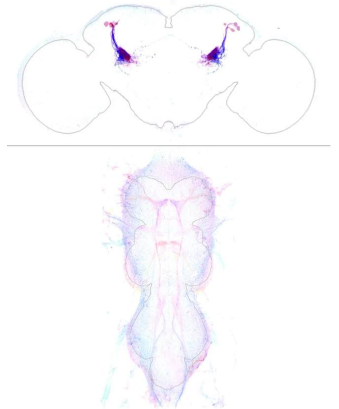
SS48834

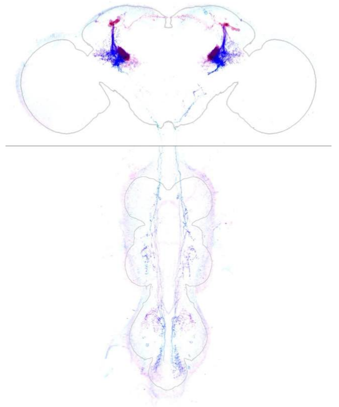
SS48866

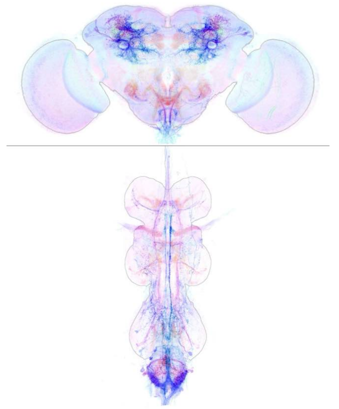
SS48892

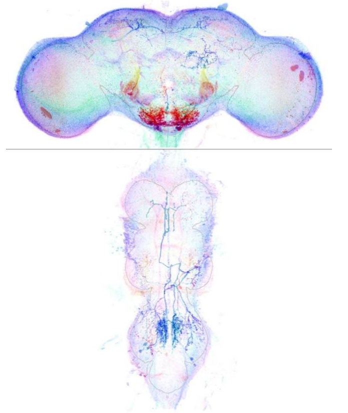
SS48918

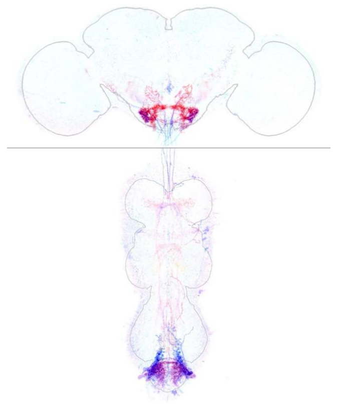
SS48944

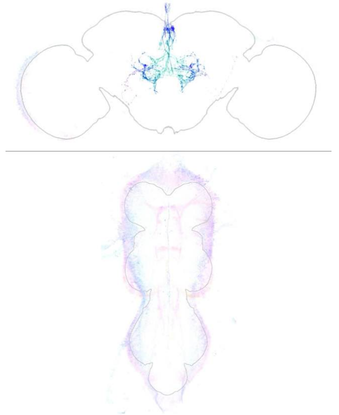
SS48979

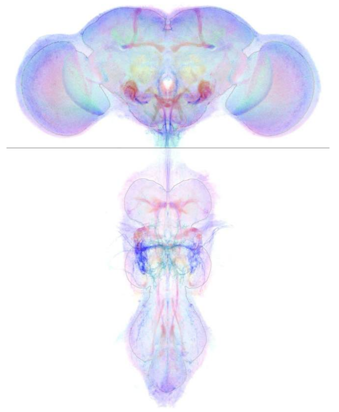
SS49039

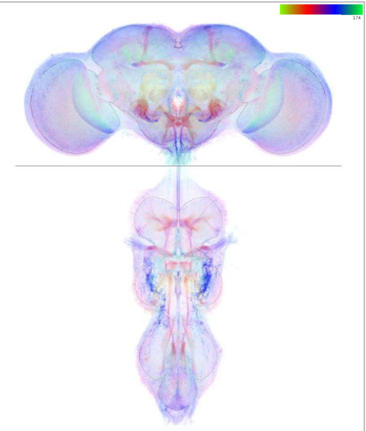

SS49041

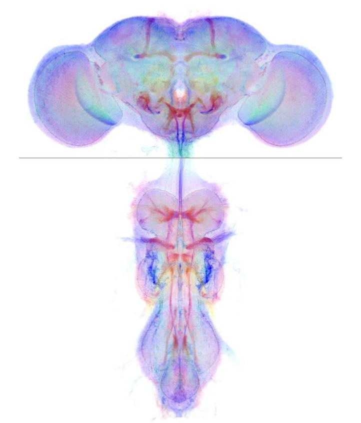

SS49042

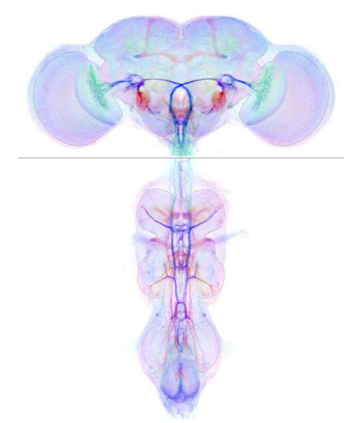

SS49051

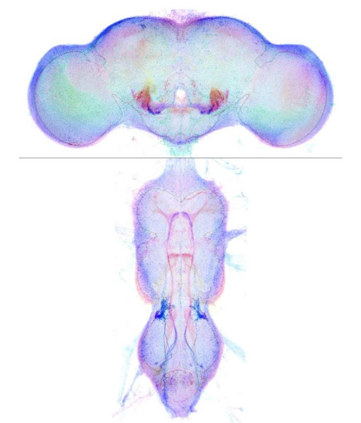

SS49125

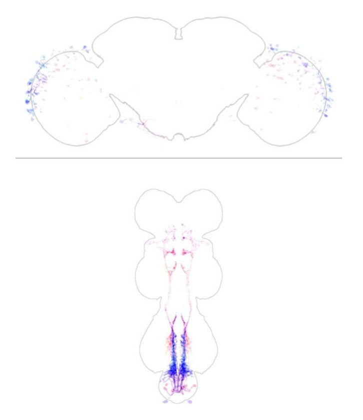

SS49157

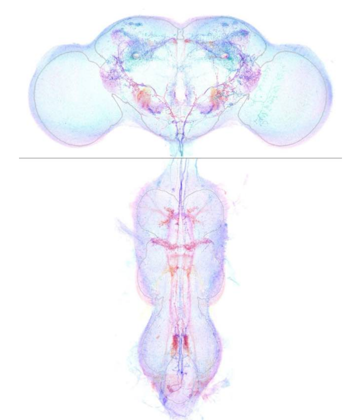

SS49159

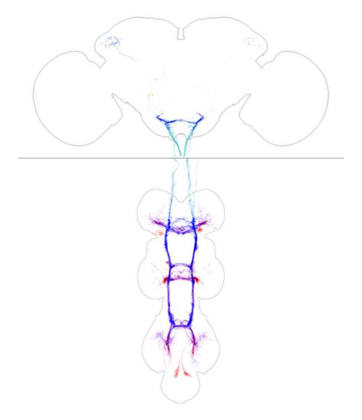

SS49172

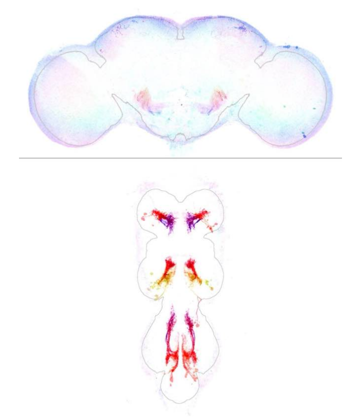

SS49190

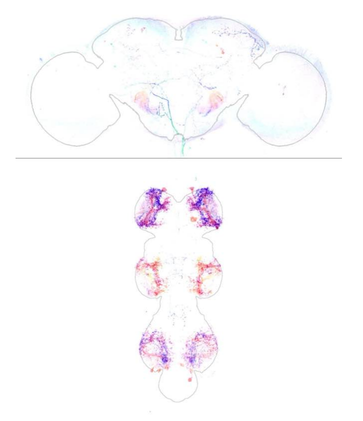

SS49220

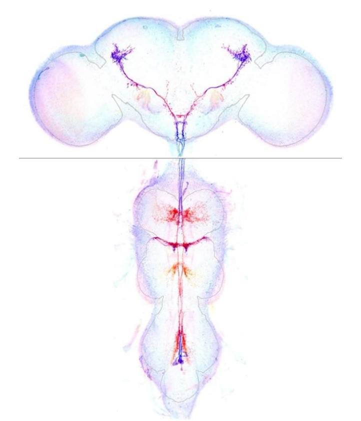

SS49267

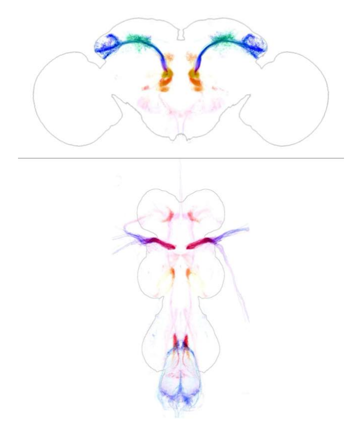

SS49298

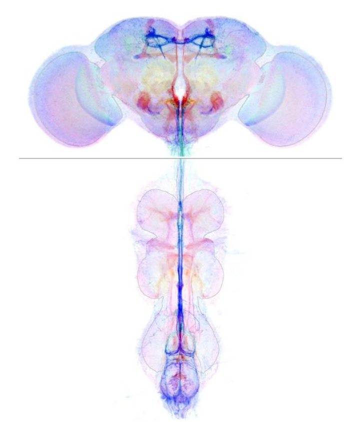

SS49300

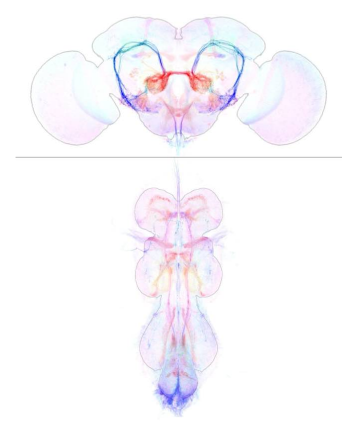

SS49304

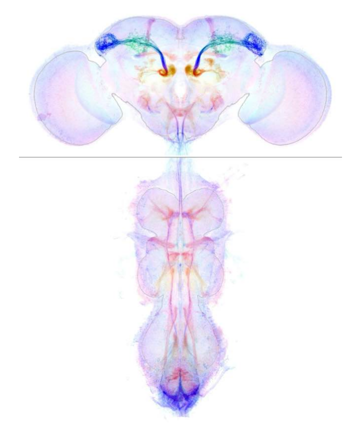

SS49306

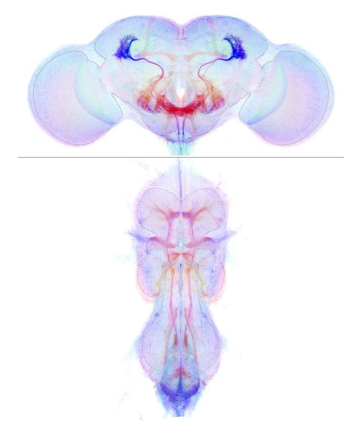

SS49345

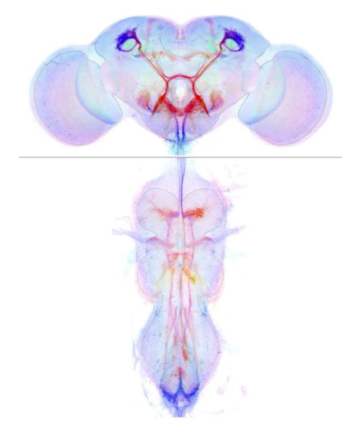

SS49352

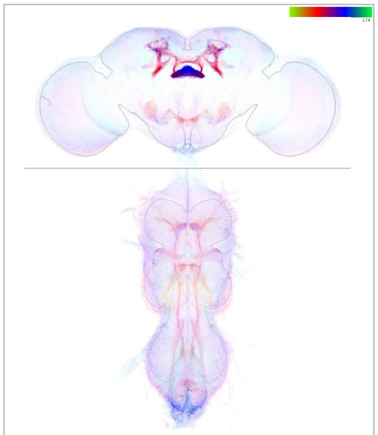
SS49376

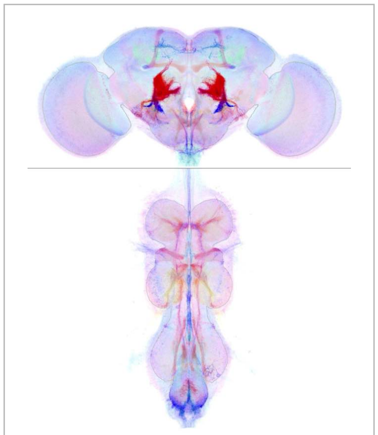
SS49386

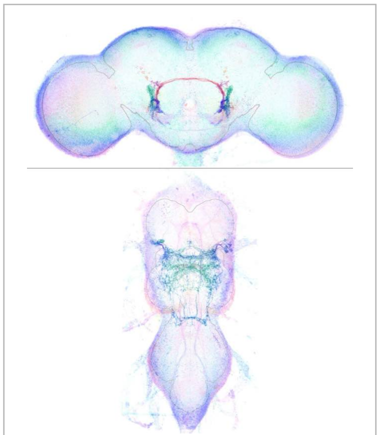
SS49406

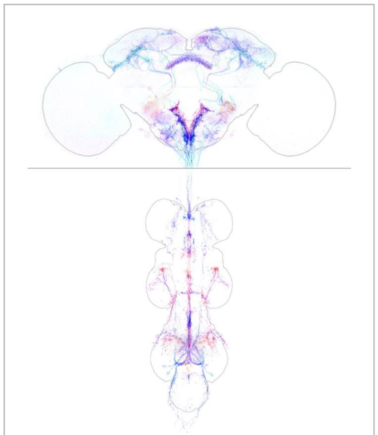
SS49443

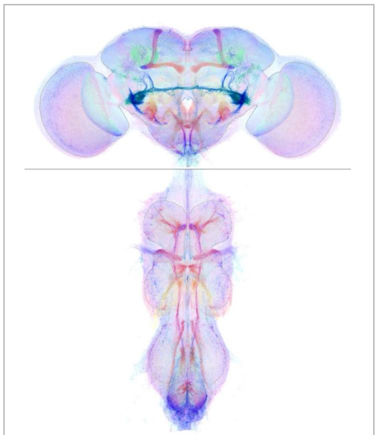
SS49630

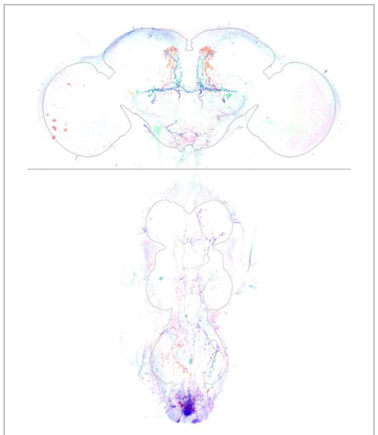
SS49658

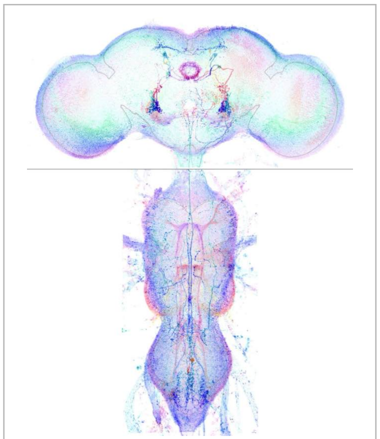
SS49695

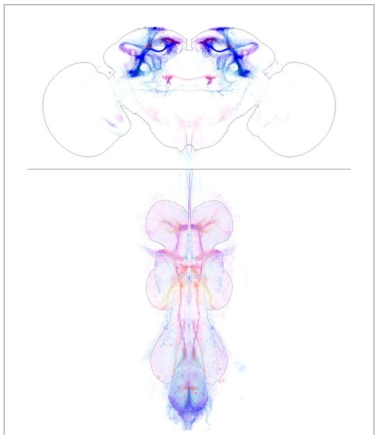
SS49755

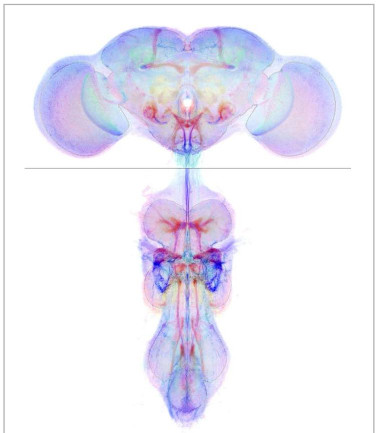
SS49766

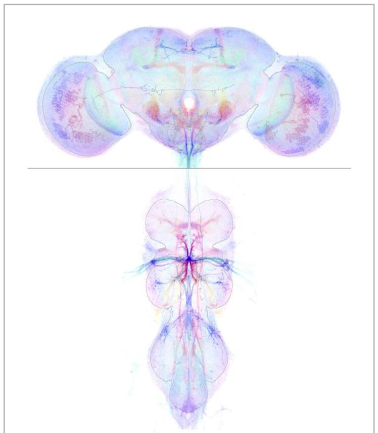
SS49777

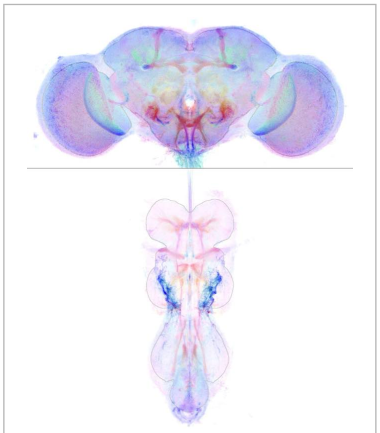
SS49778

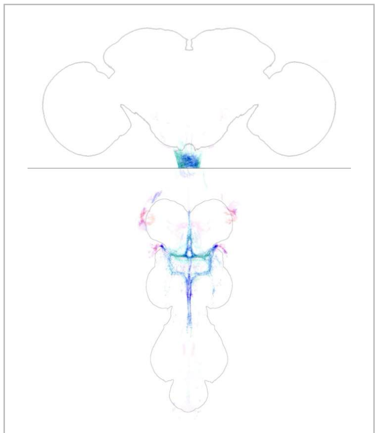
SS49779

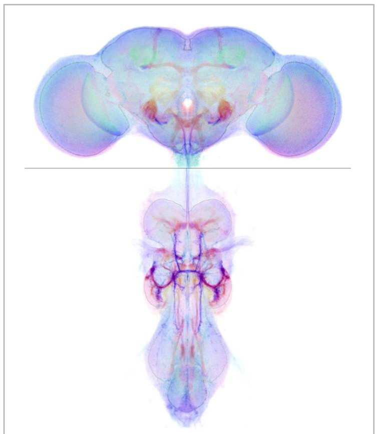
SS49784

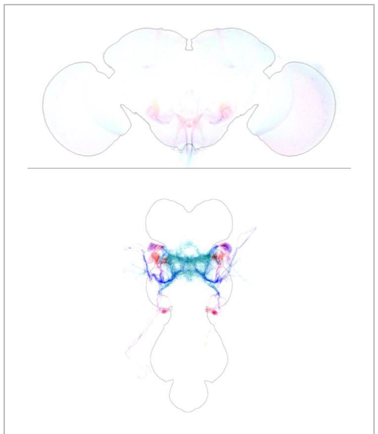
SS49797

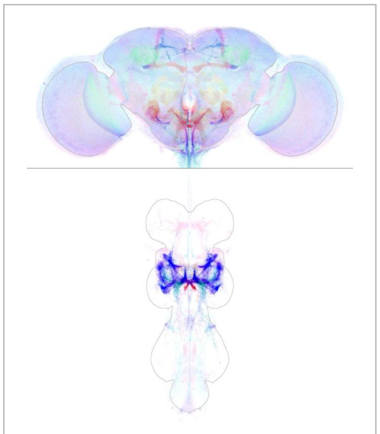
SS49799

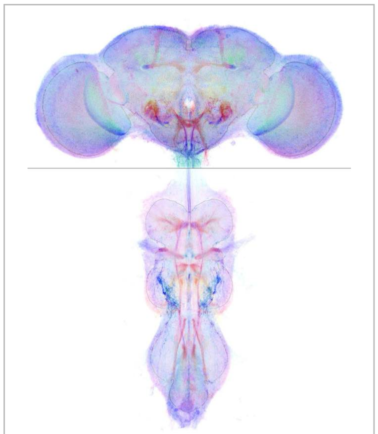
SS49800

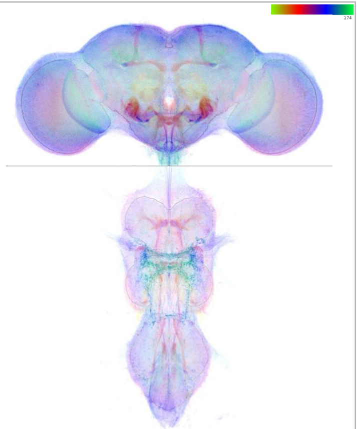
SS49802

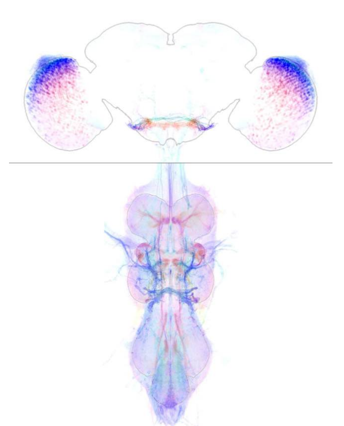
SS49806

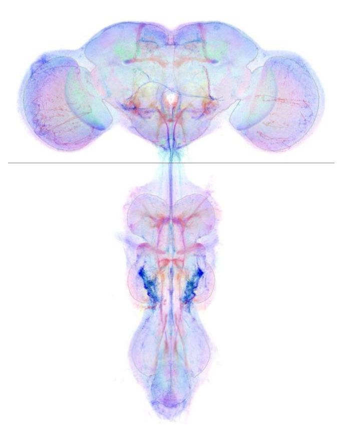
SS49807

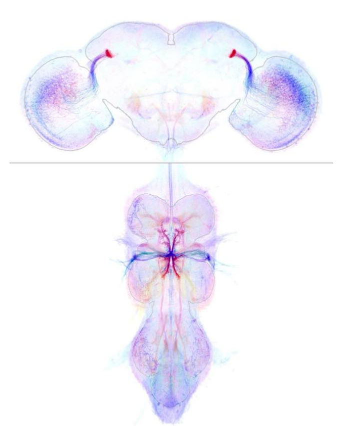
SS49809

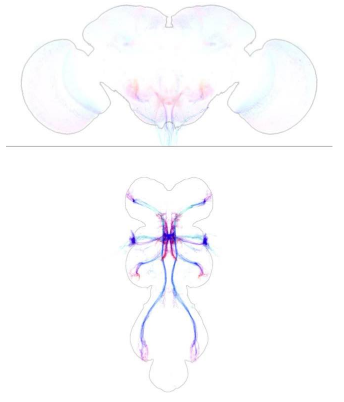
SS49810

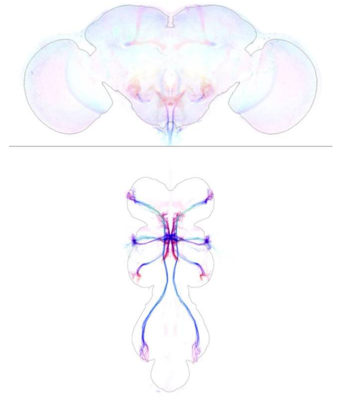
SS49812

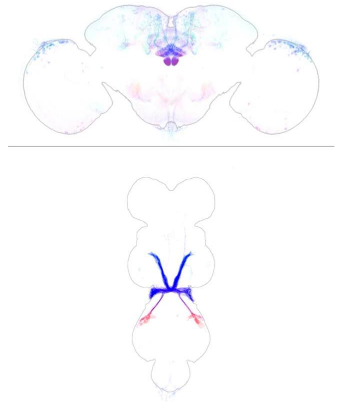
SS49853

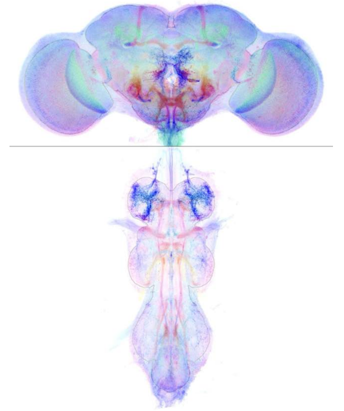
SS49861

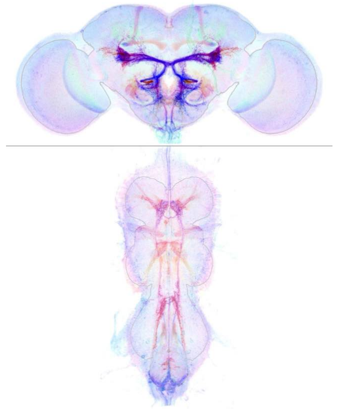
SS49868

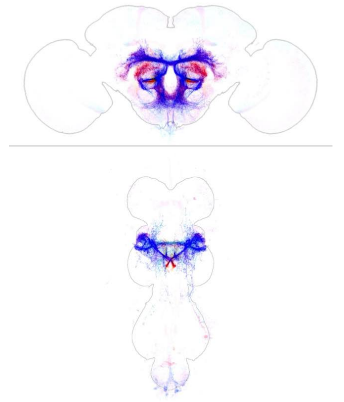
SS49882

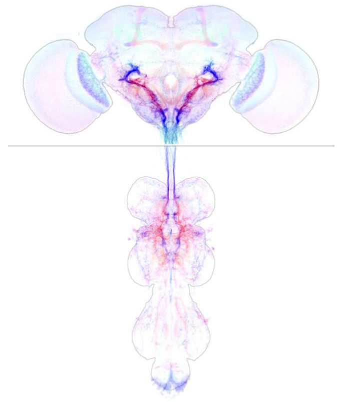
SS49890

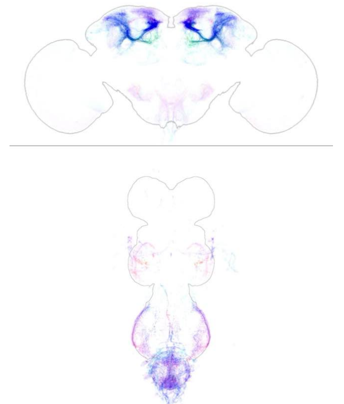
SS49896

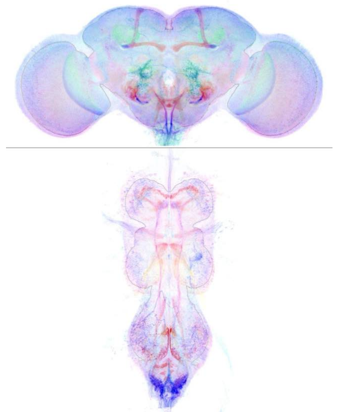
SS49909

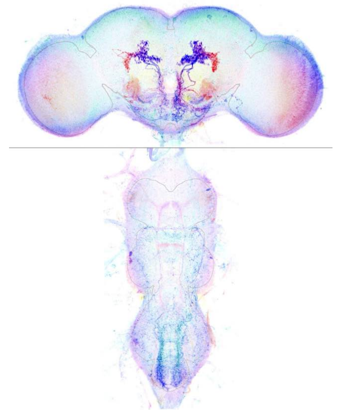
SS49915

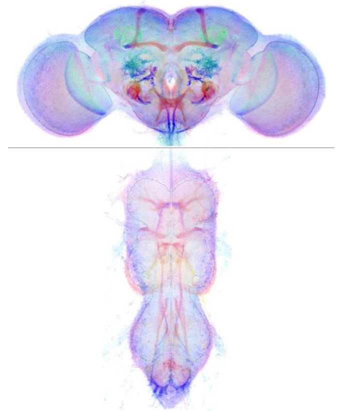
SS49923

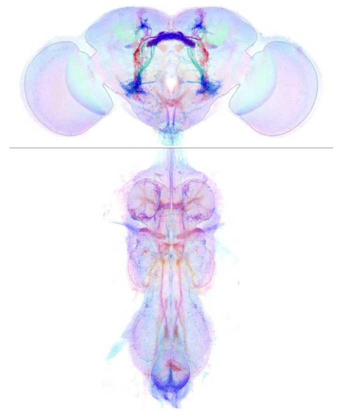
SS49931

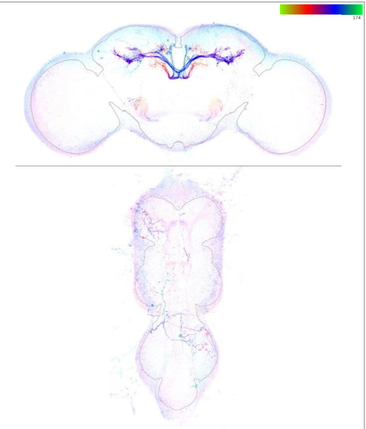

SS49951

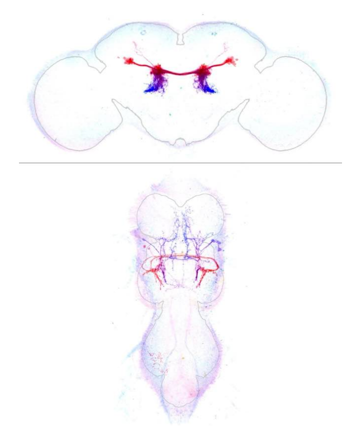

SS49952

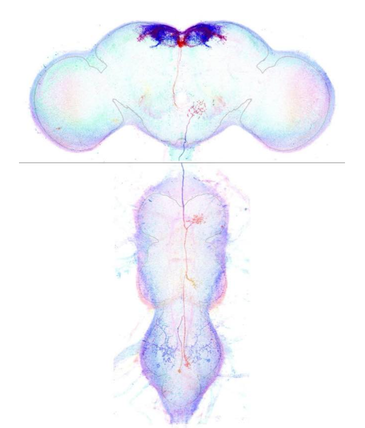

SS49970

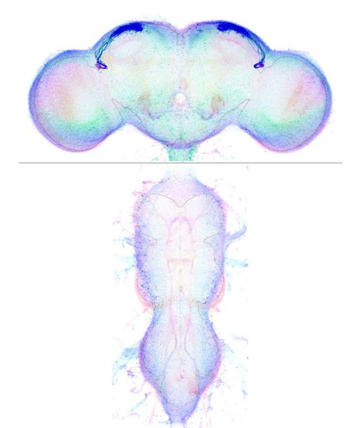

SS49971

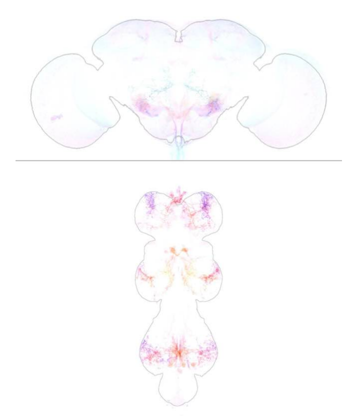

SS49978

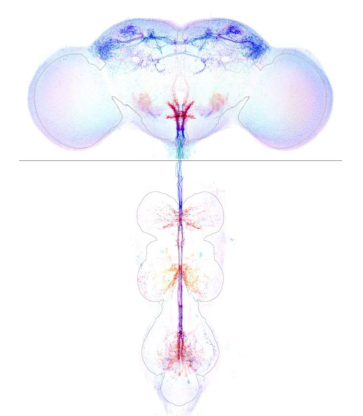

SS49982

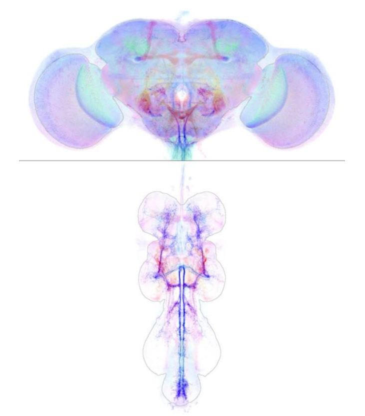

SS50012

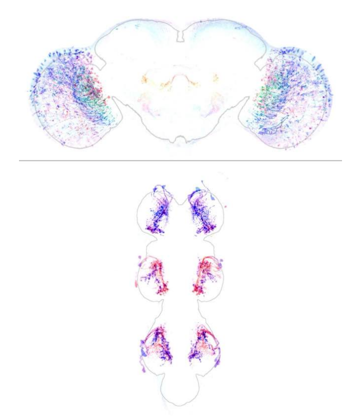

SS50022

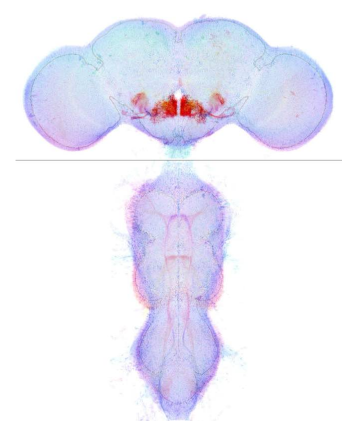

SS50143

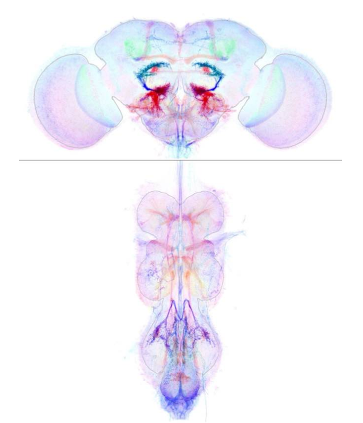

SS50173

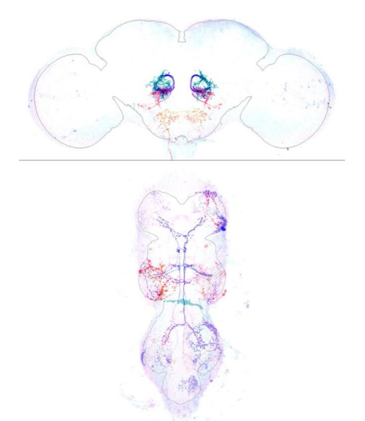

SS50176

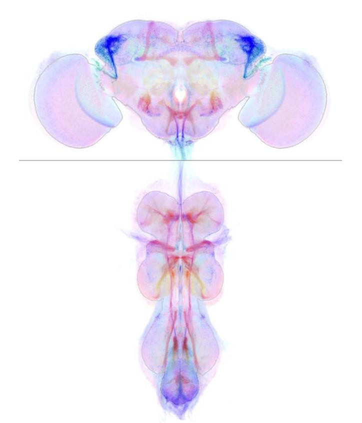

SS50188

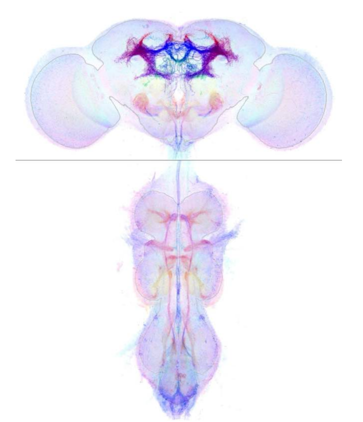

SS50191

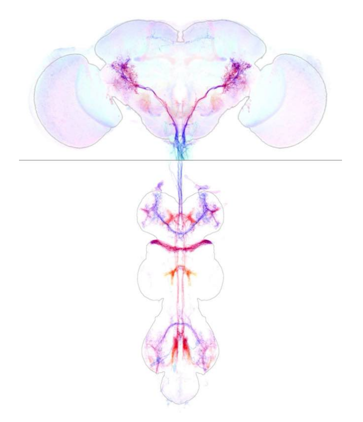

SS50195

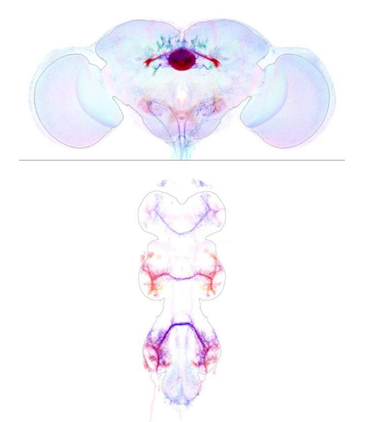

SS50197

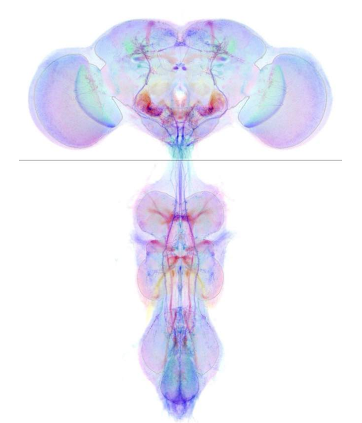

SS50200

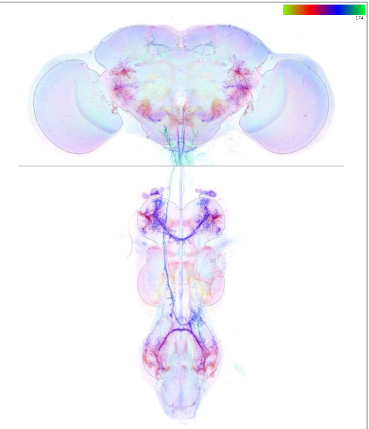

SS50201

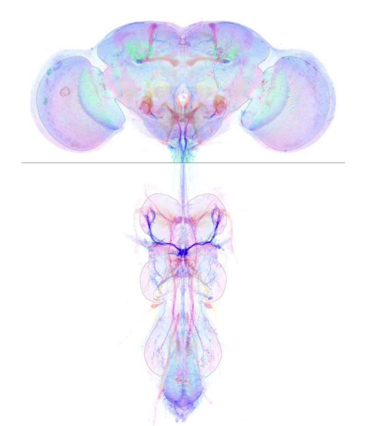

SS50207

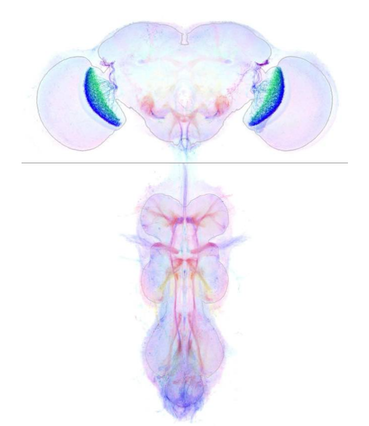

SS50215

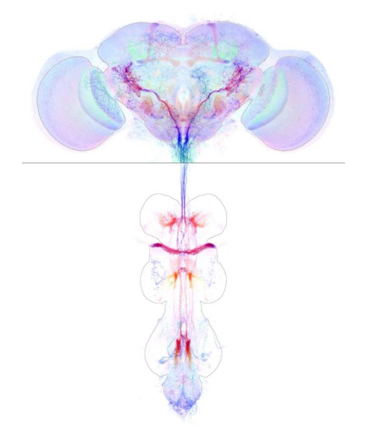

SS50218

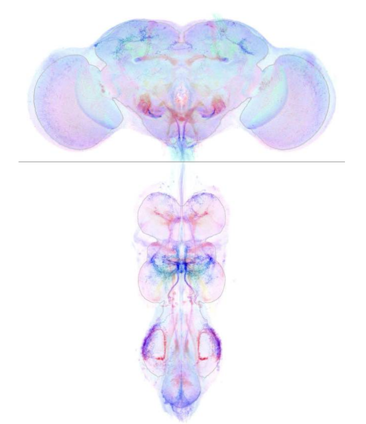

SS50245

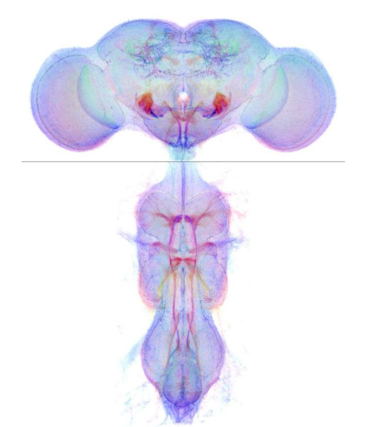

SS50258

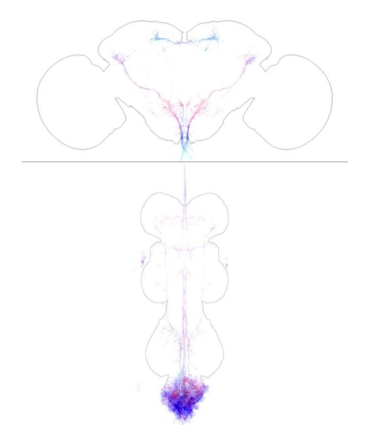

SS50268

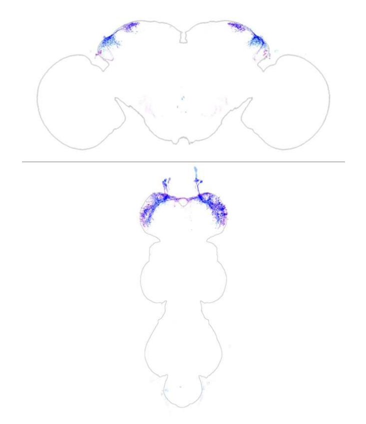

SS50281

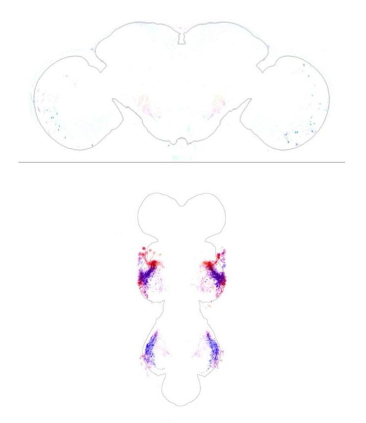

SS50313

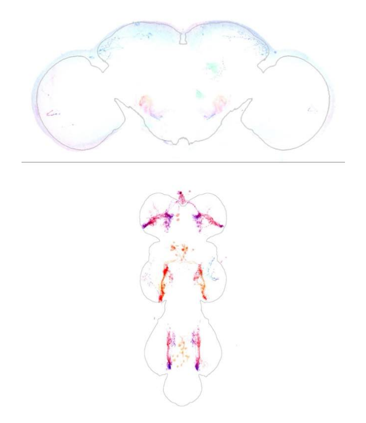

SS50329

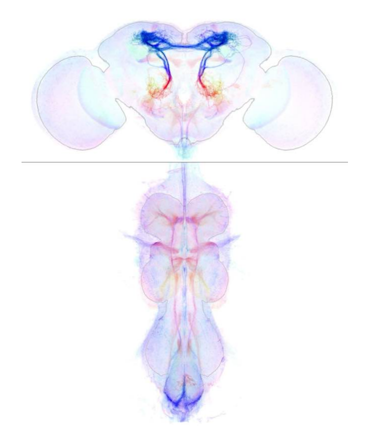

SS50333

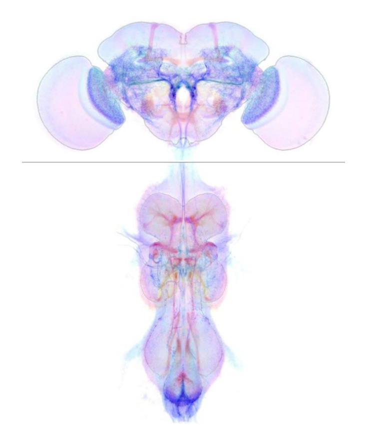

SS50336

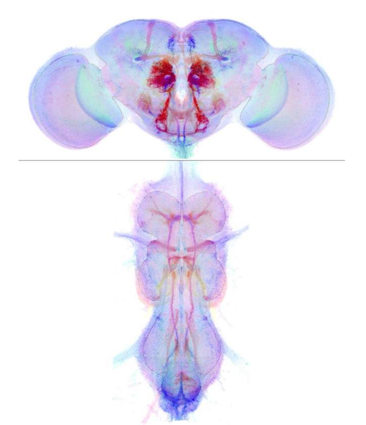

SS50337

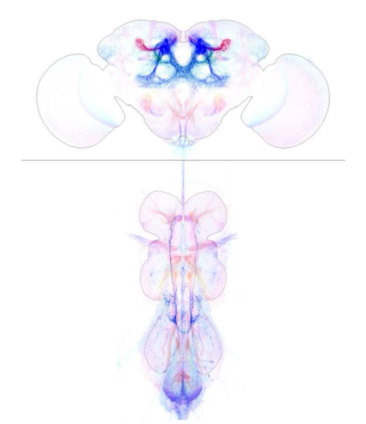

SS50351

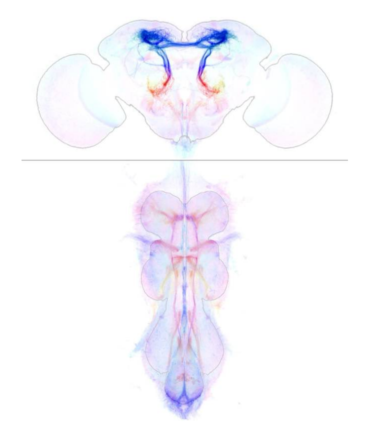

SS50353

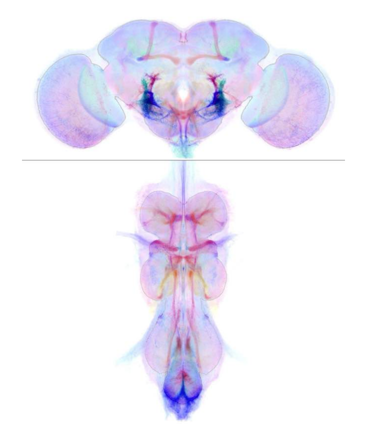

SS50362

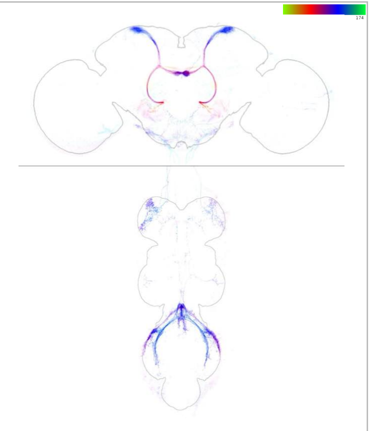
SS50413

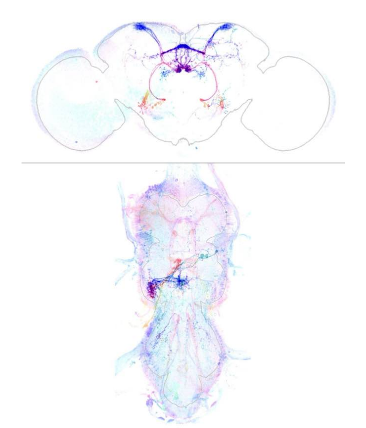
SS50419

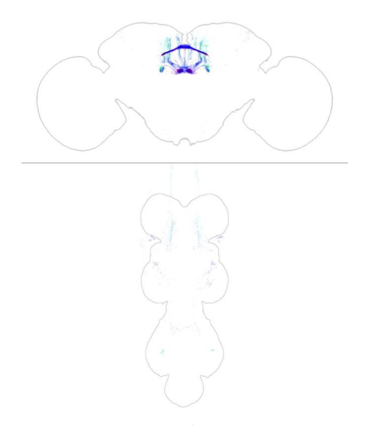
SS50447

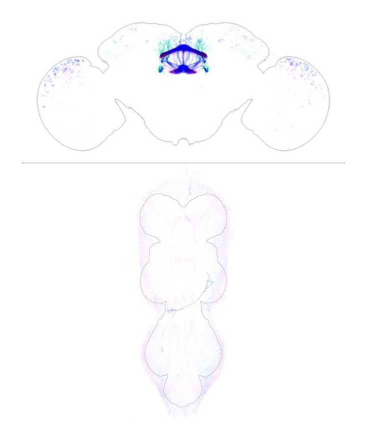
SS50485

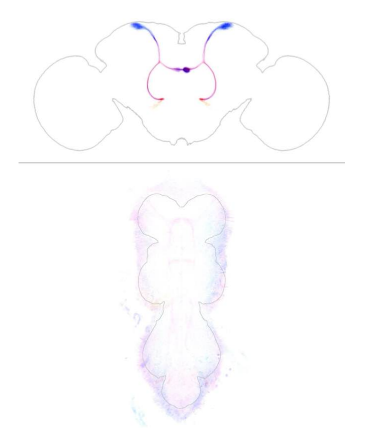
SS50498

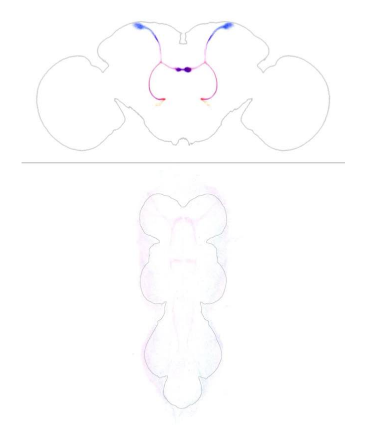
SS50502

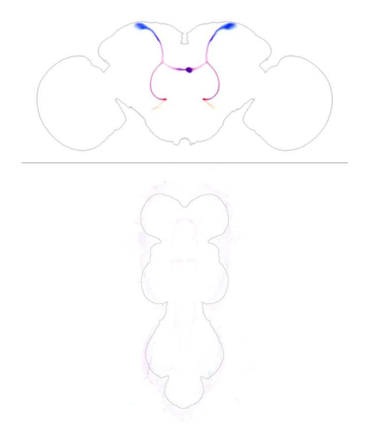
SS50510

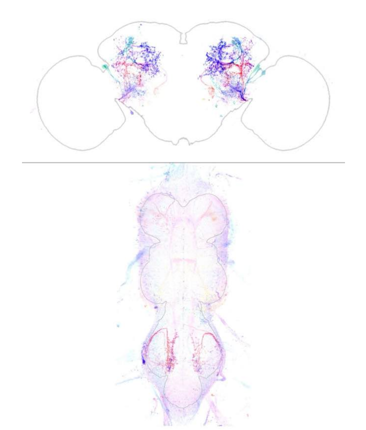
SS50567

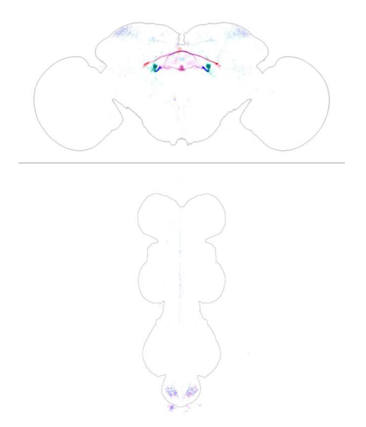
SS50576

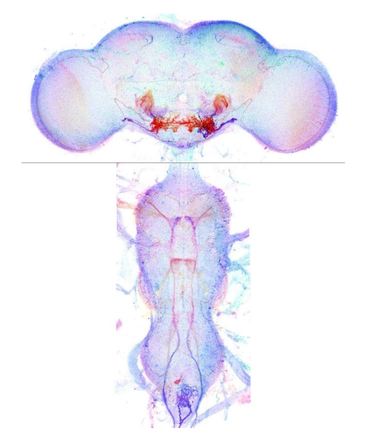
SS50701

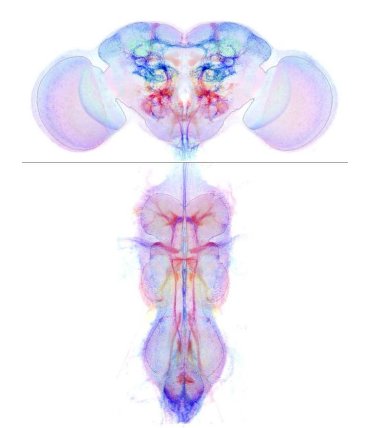
SS50738

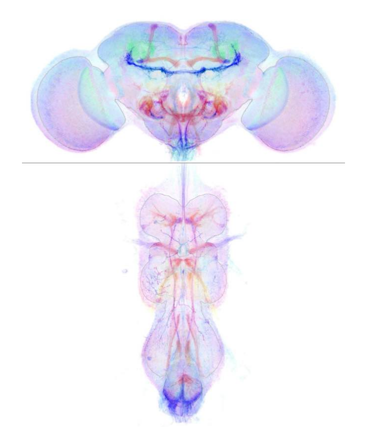
SS50747

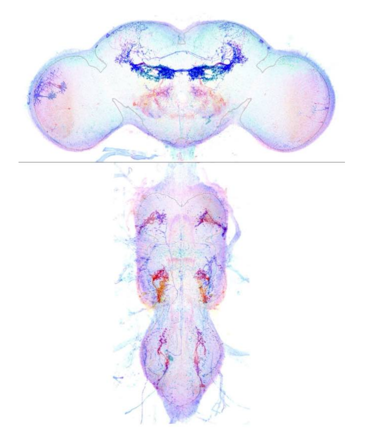
SS50750

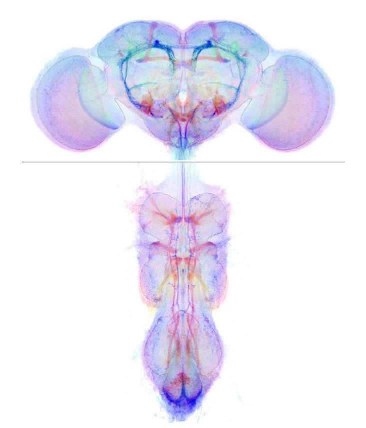
SS50756

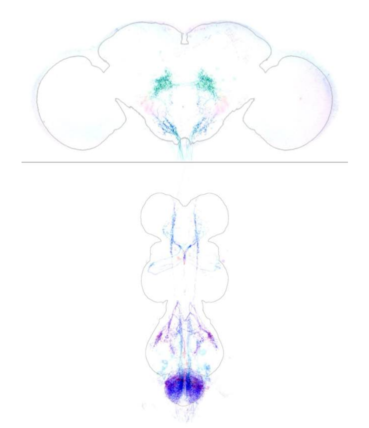
SS50766

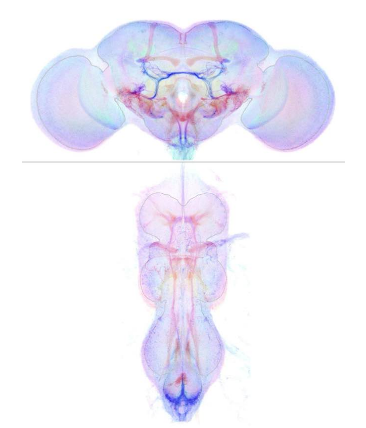
SS50767

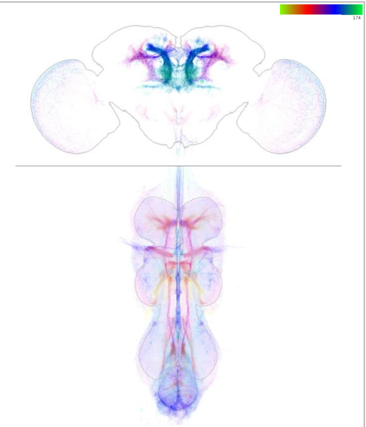
SS50785
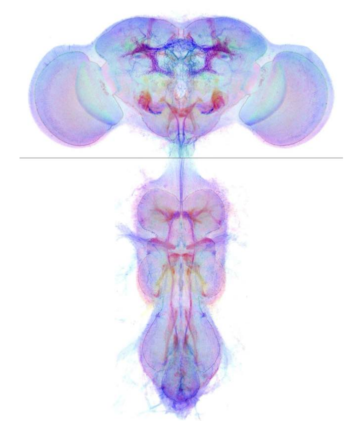
SS50786
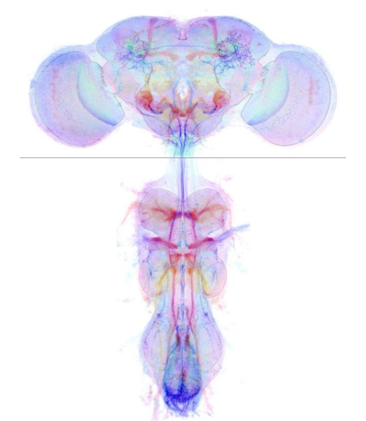
SS50795
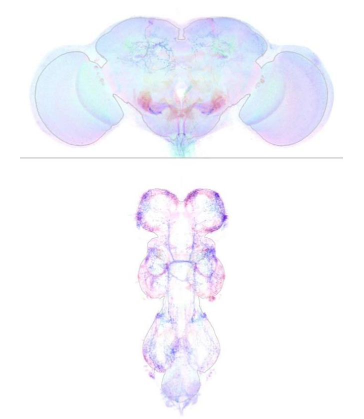
SS50802
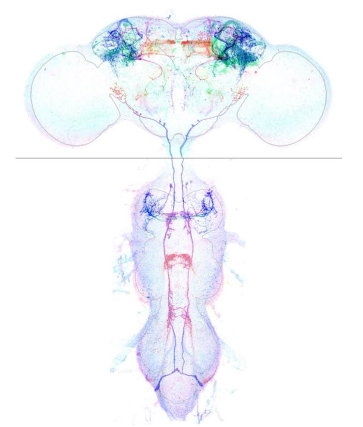
SS50814
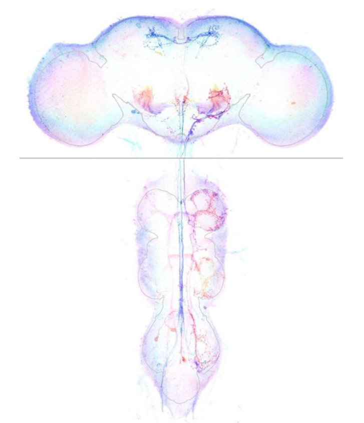
SS50829
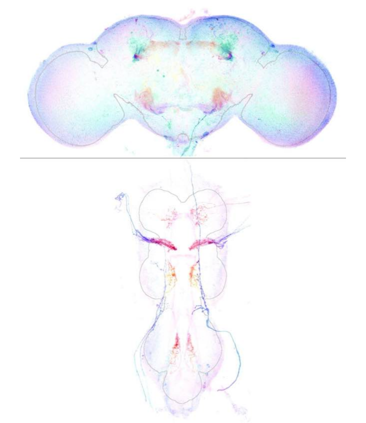
SS50841
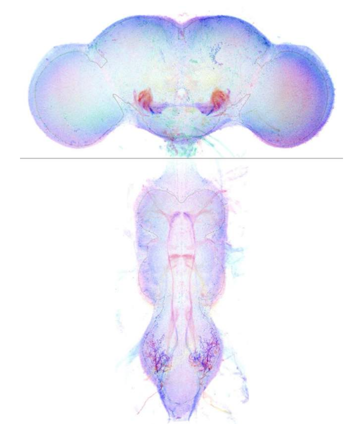
SS50865
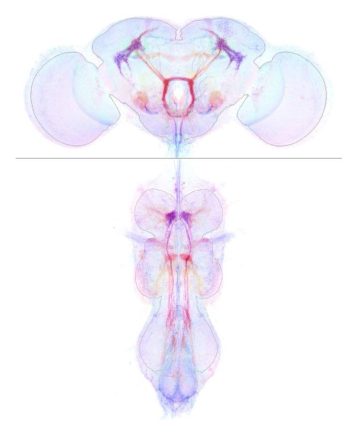
SS50889
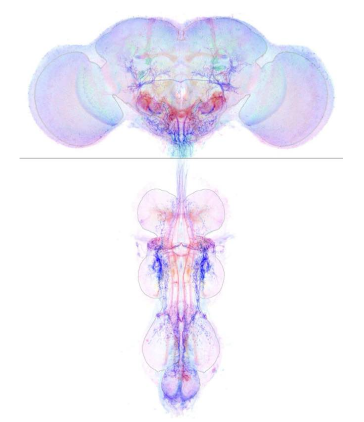
SS50896
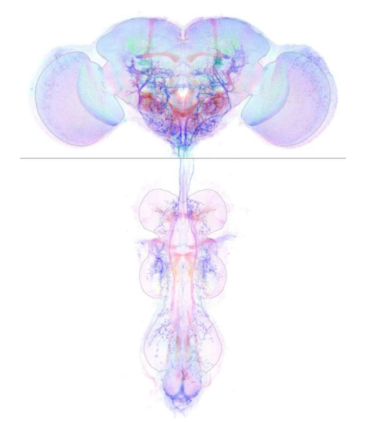
SS50897
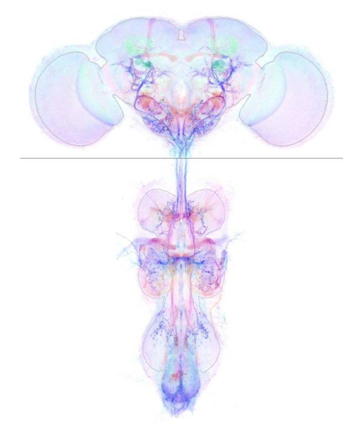
SS50898
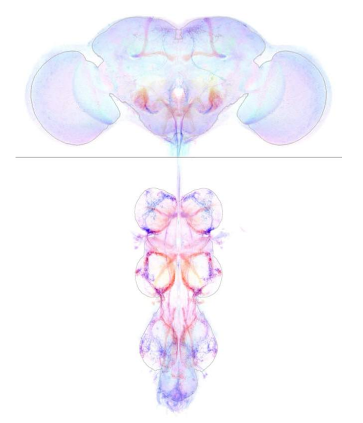
SS50907
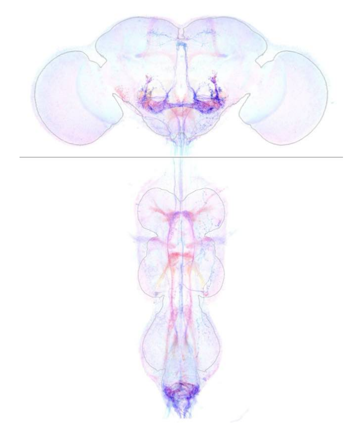
SS50910
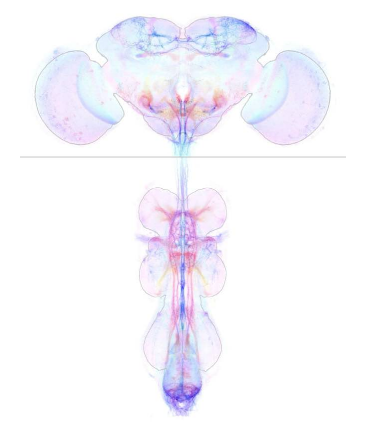
SS50911
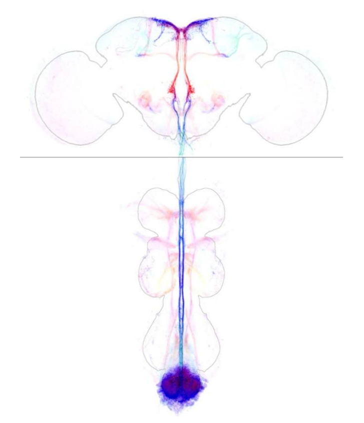
SS50914

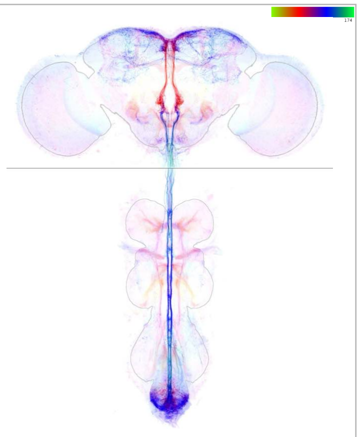
SS50915

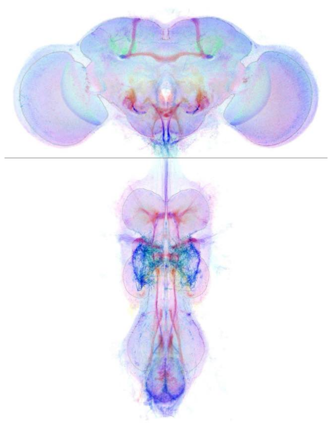
SS50916

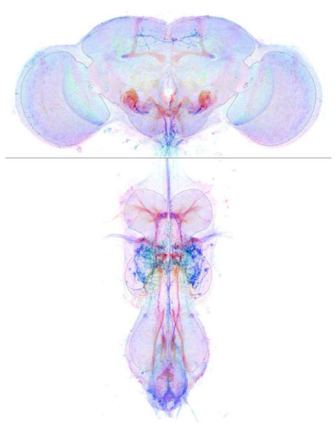
SS50919

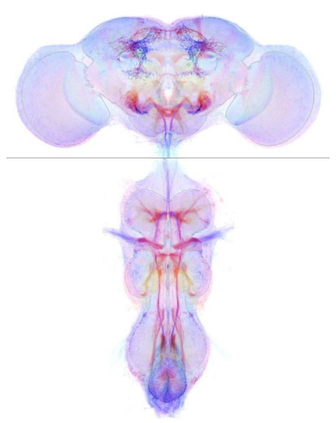
SS50921

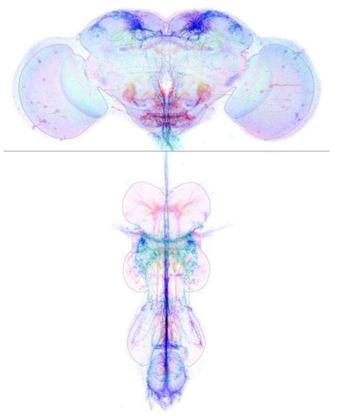
SS50928

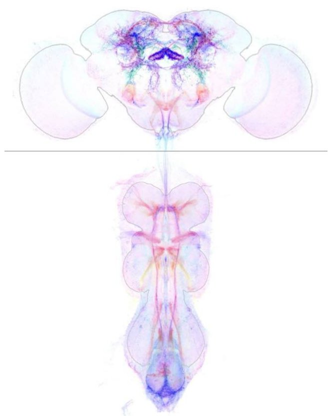
SS50931

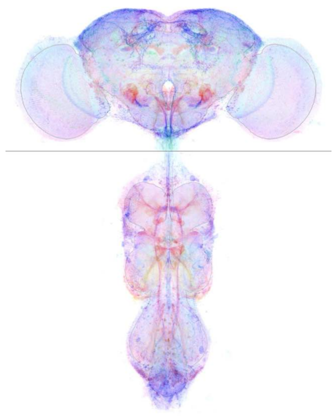
SS50937

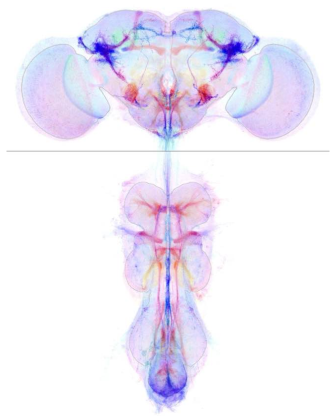
SS50938

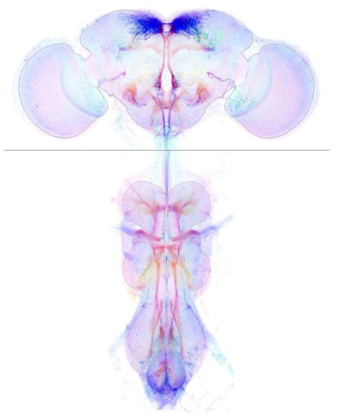
SS50940

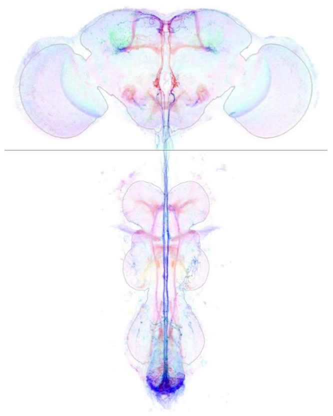
SS50945

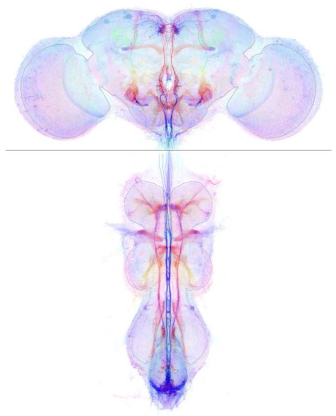
SS50946

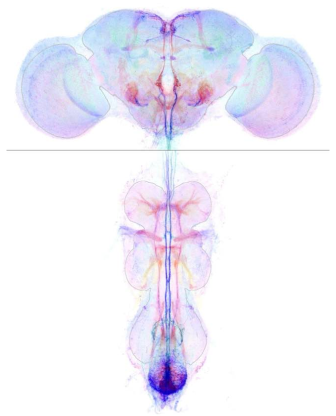
SS50947

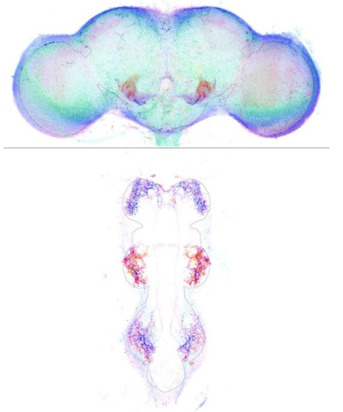
SS50965

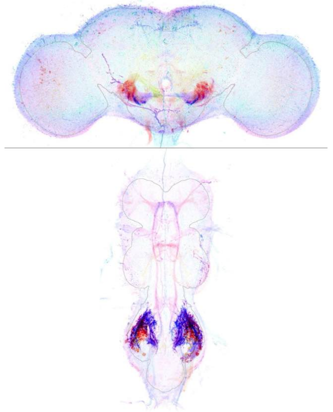
SS50974

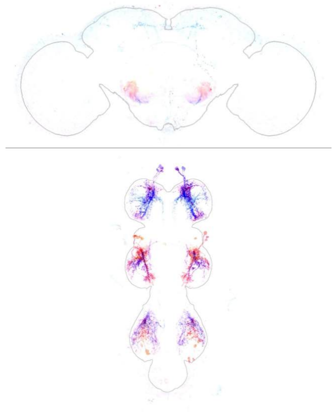
SS50975

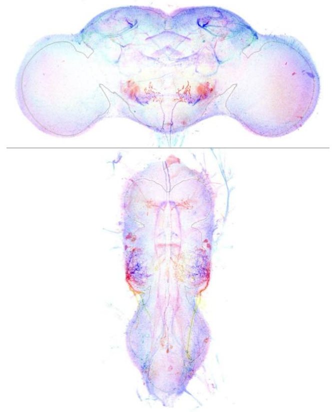
SS50991

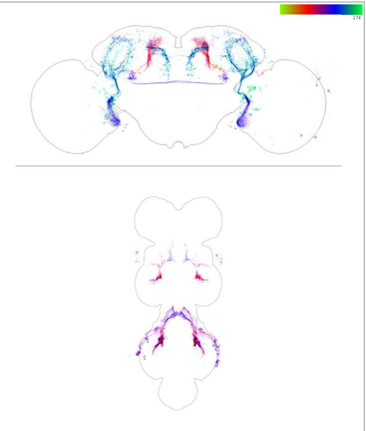
SS50995

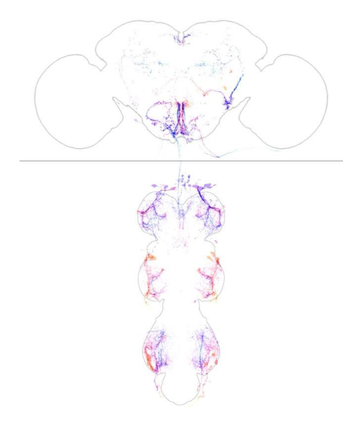
SS50996

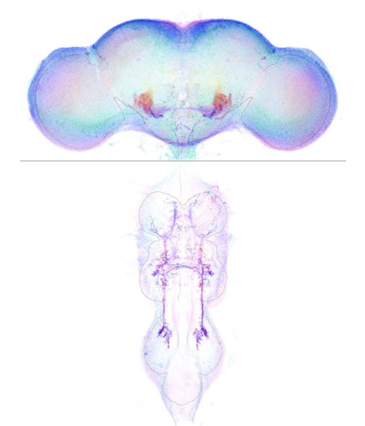
SS51006

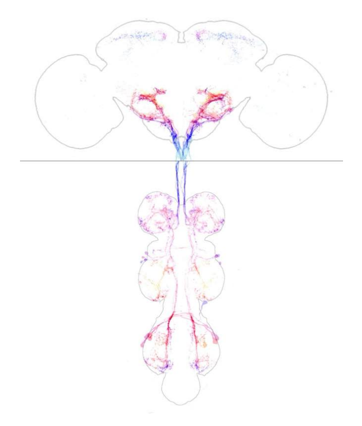
SS51017

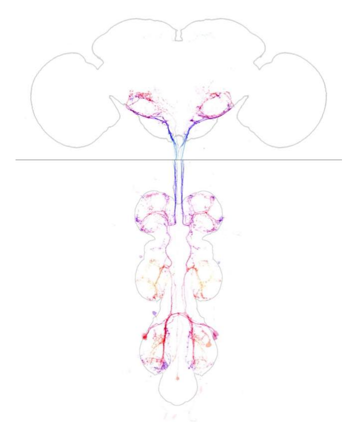
SS51021

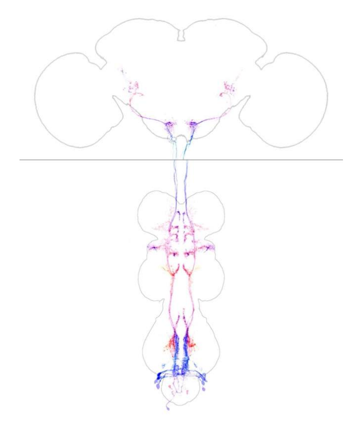
SS51029

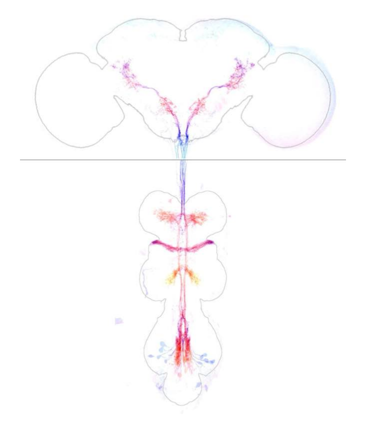
SS51038

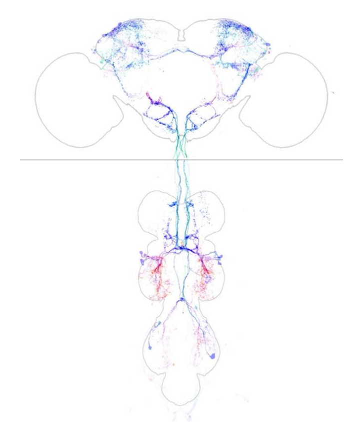
SS51046

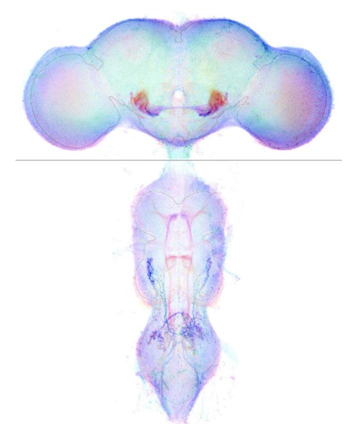
SS51105

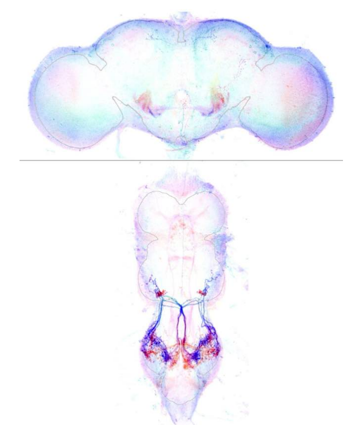
SS51112

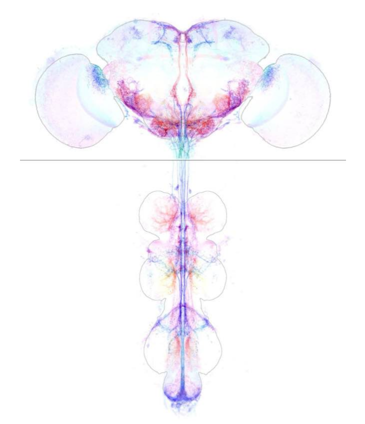
SS51118

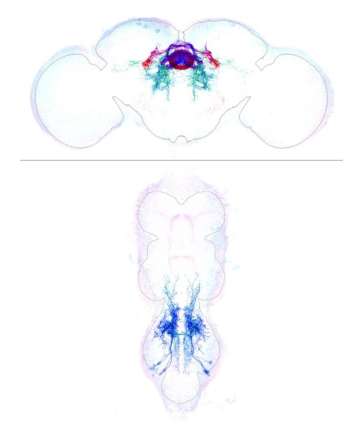
SS51128

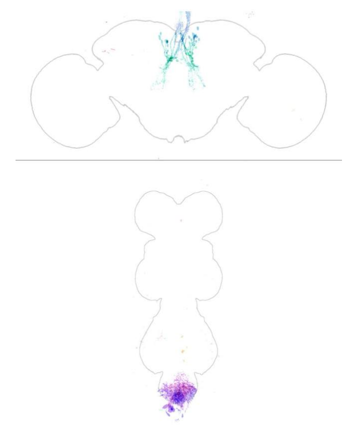
SS51143

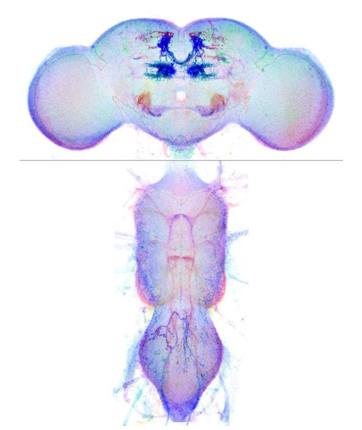
SS51152

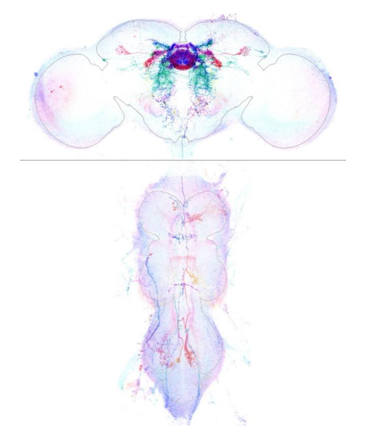
SS51169

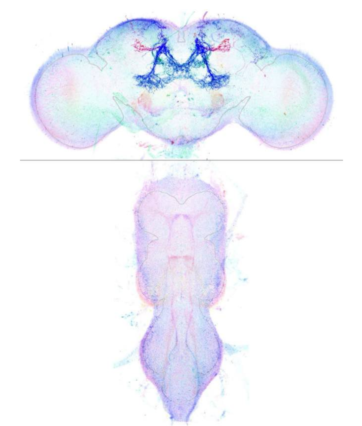
SS51173

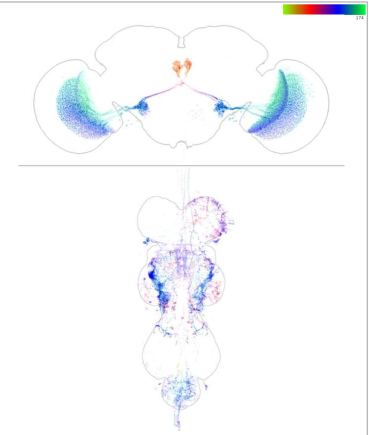
SS51209

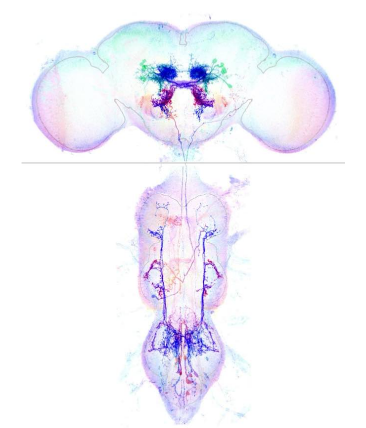
SS51247

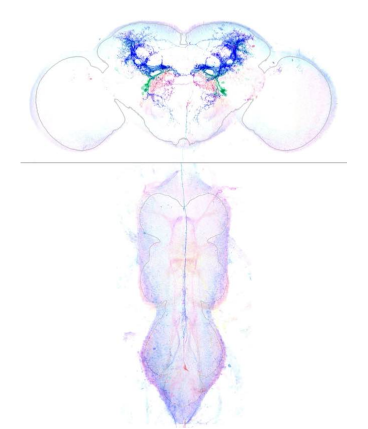
SS51264

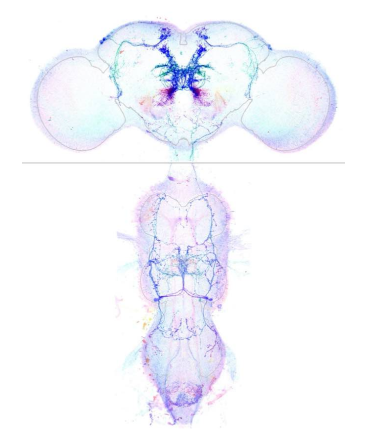
SS51274

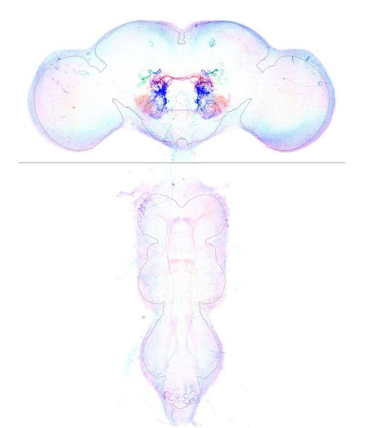
SS51277

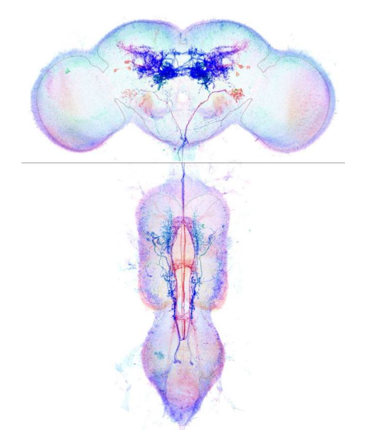
SS51305

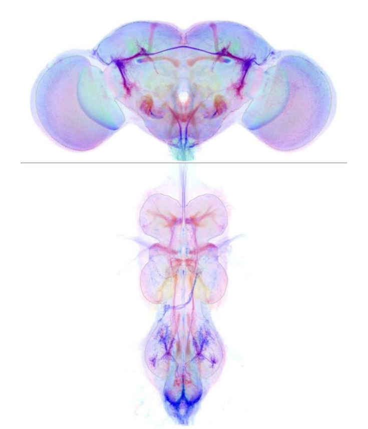
SS51359

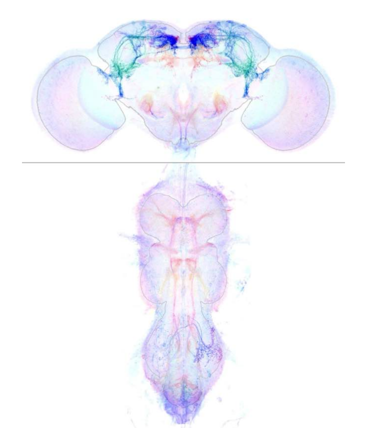
SS51399

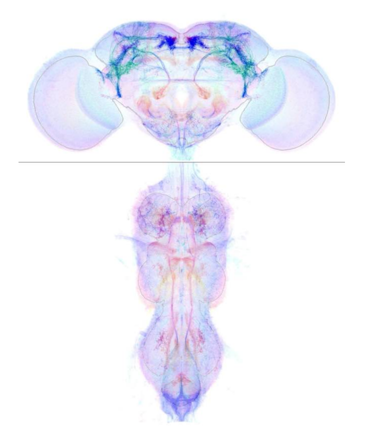
SS51407

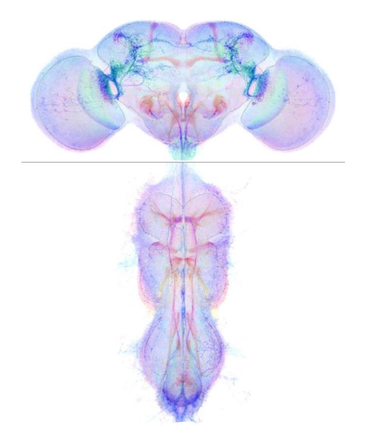
SS51421

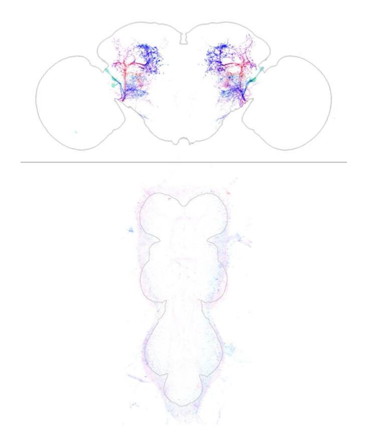
SS51474

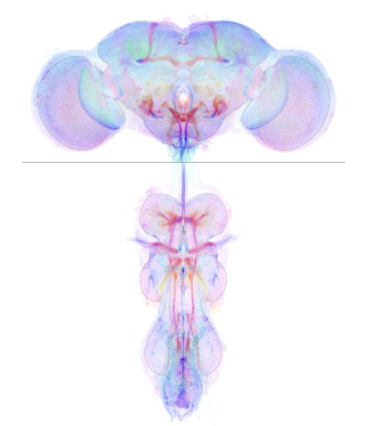
SS51508

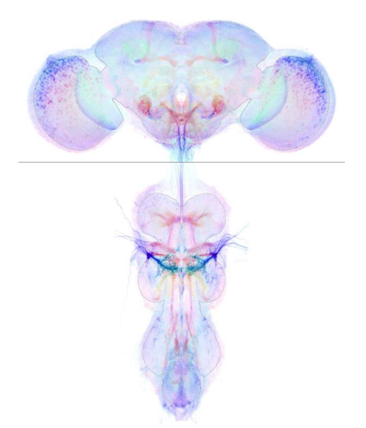
SS51519

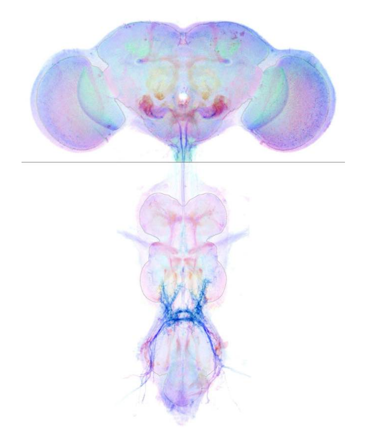
SS51524

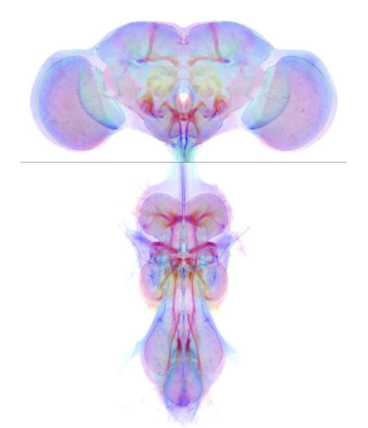
SS51528

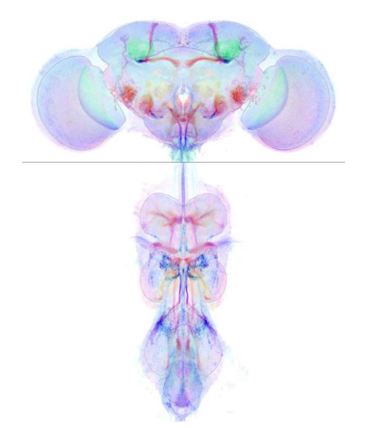
SS51531

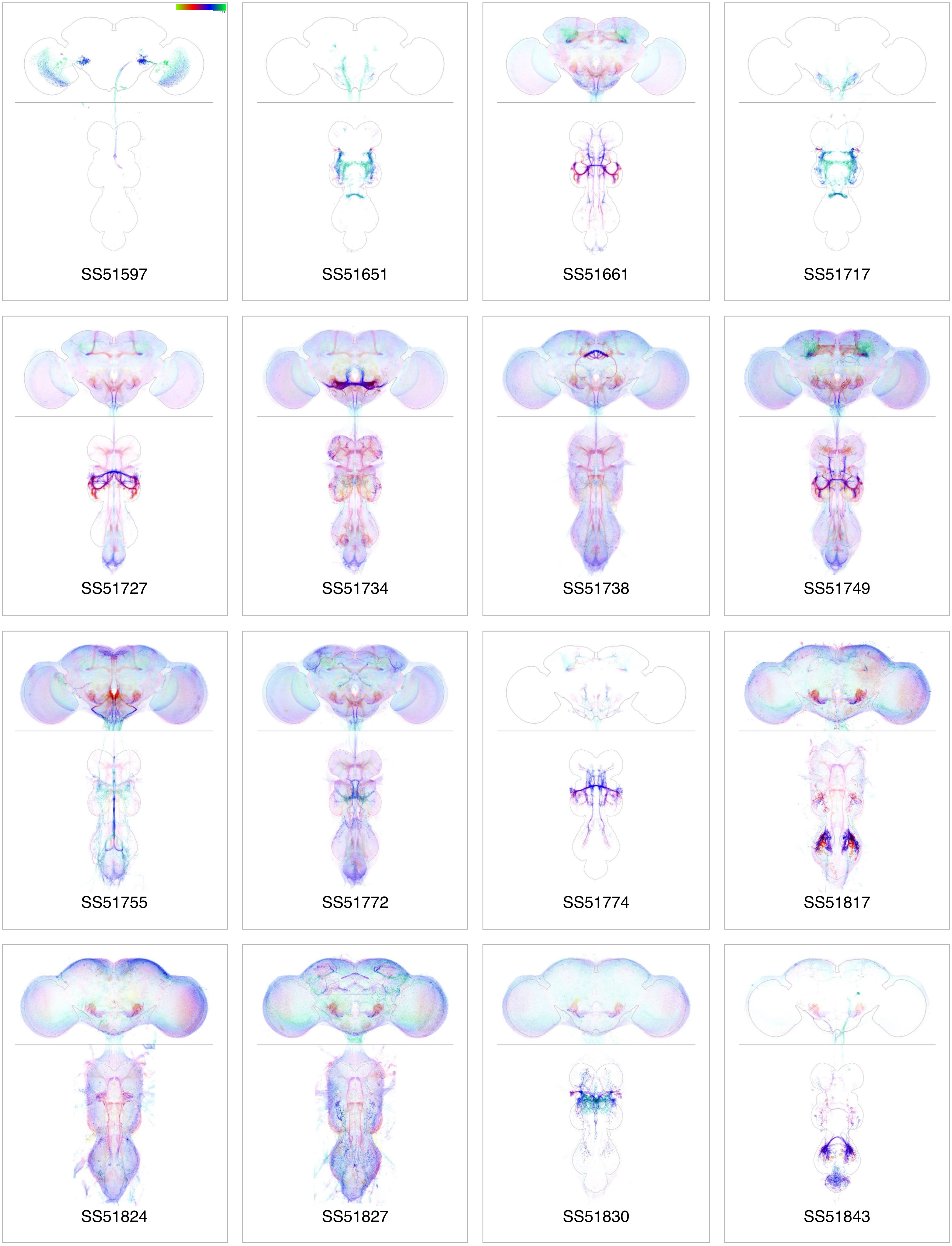
SS51597
SS51651
SS51661
SS51717
SS51727
SS51734
SS51738
SS51749
SS51755
SS51772
SS51774
SS51817
SS51824
SS51827
SS51830
SS51843

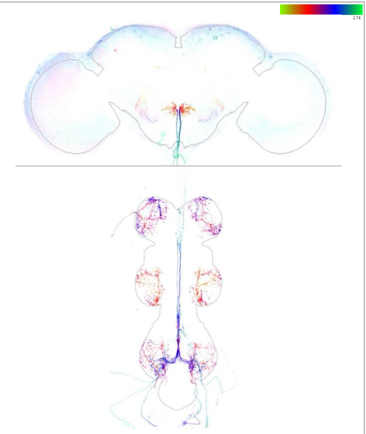
SS51867

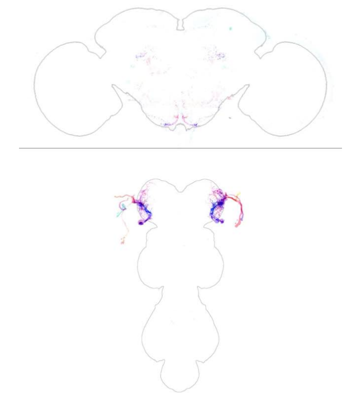
SS51874

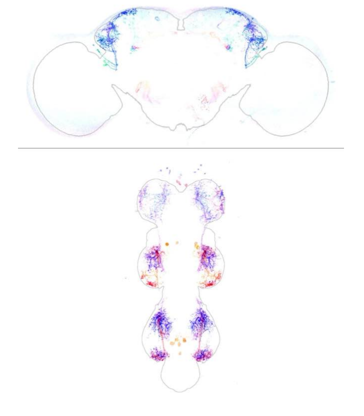
SS51877

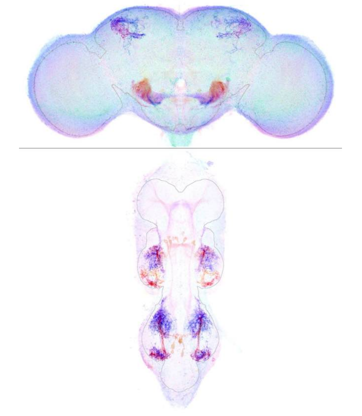
SS51891

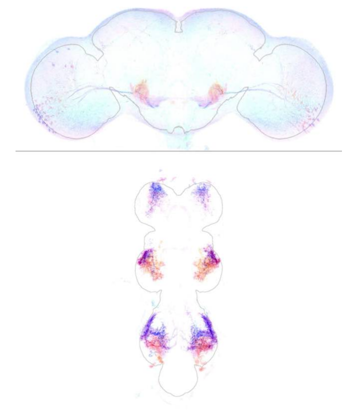
SS51893

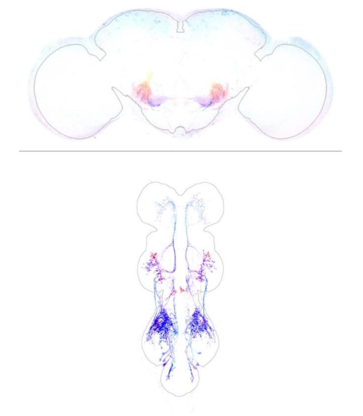
SS51895

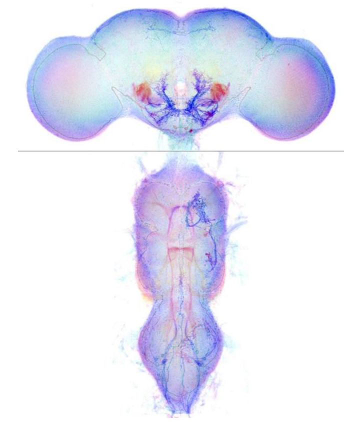
SS51896

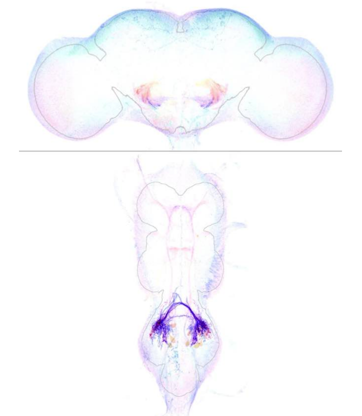
SS51907

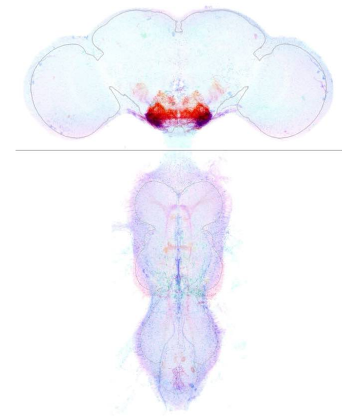
SS51930

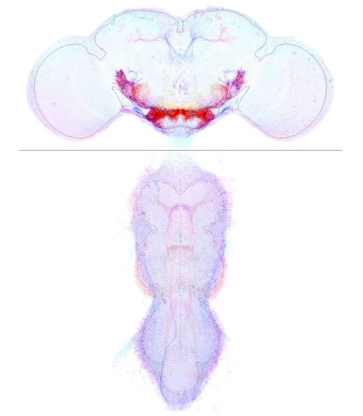
SS51933

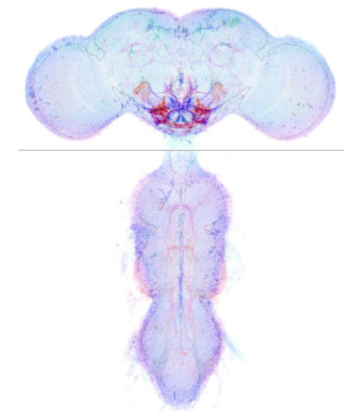
SS51950

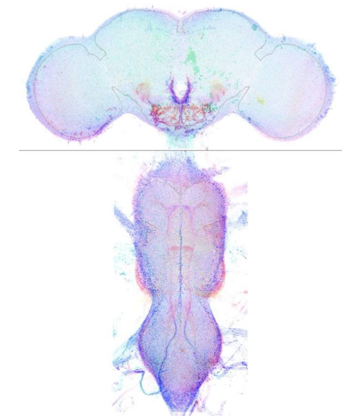
SS51988

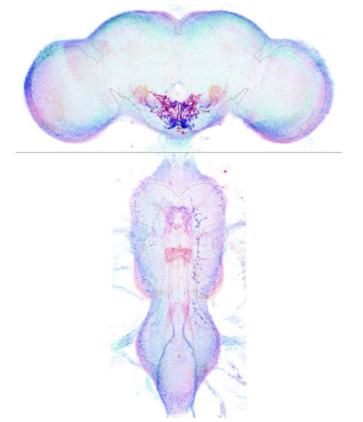
SS51996

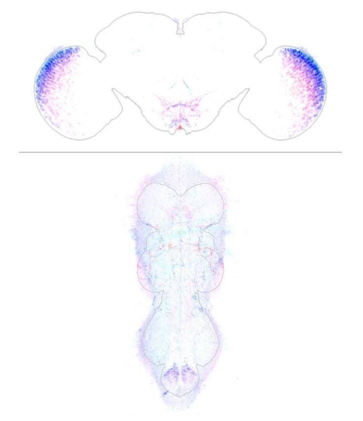
SS51997

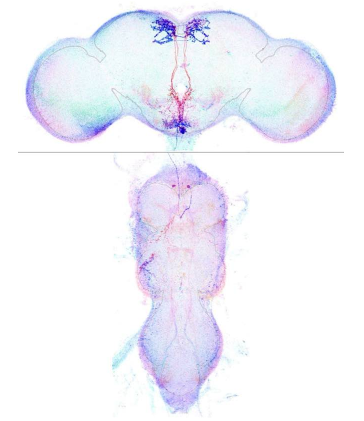
SS52034

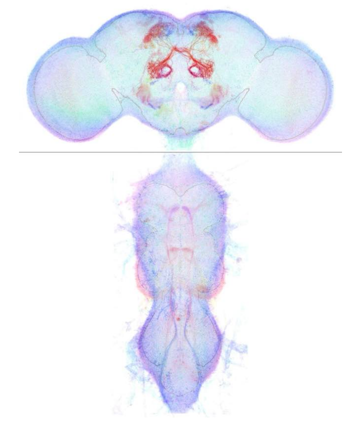
SS52050

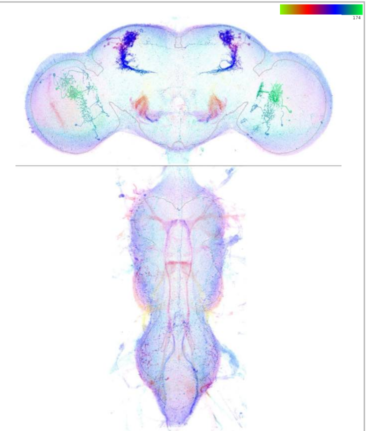
SS52124

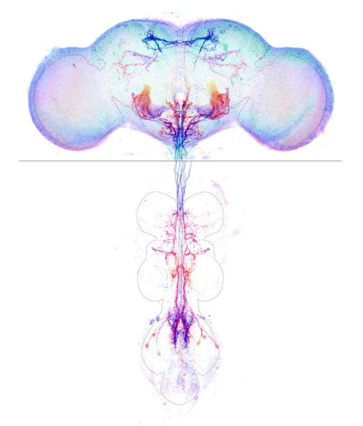
SS52147

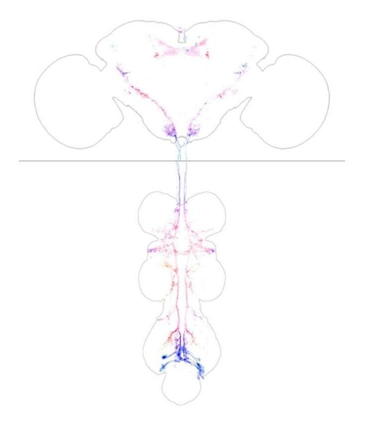
SS52172

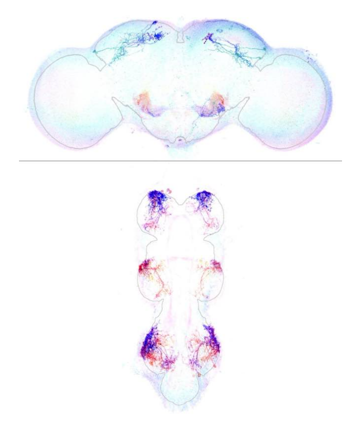
SS52179

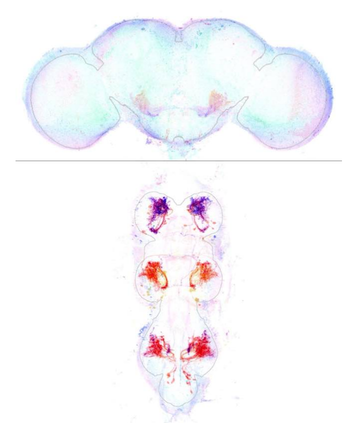
SS52196

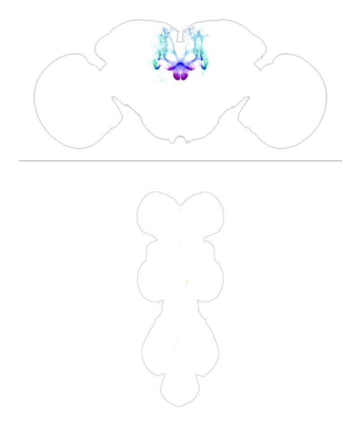
SS52244

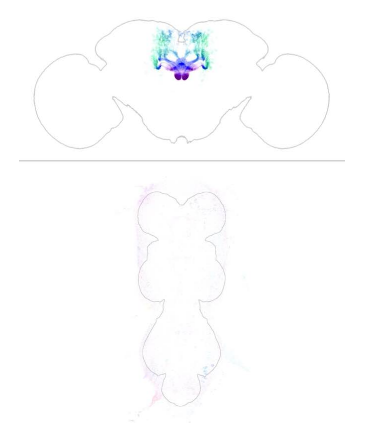
SS52245

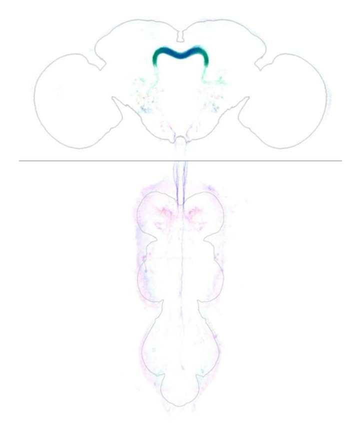
SS52257

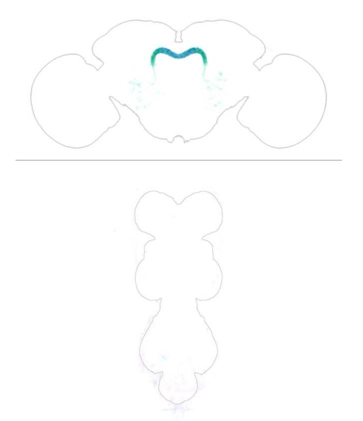
SS52313

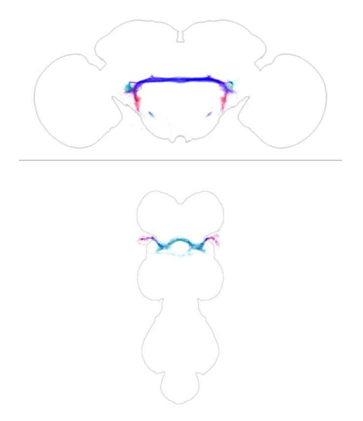
SS52389

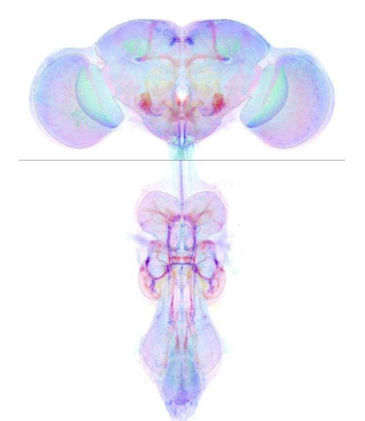
SS52392

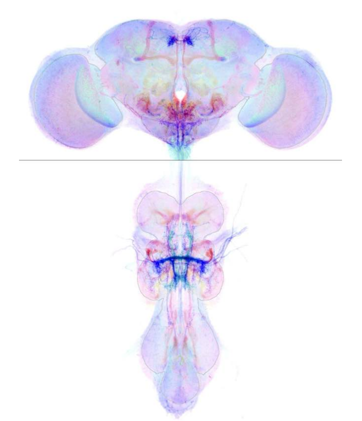
SS52395

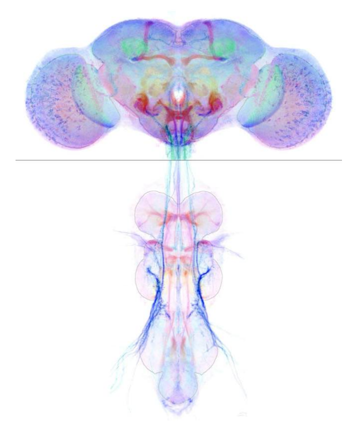
SS52396

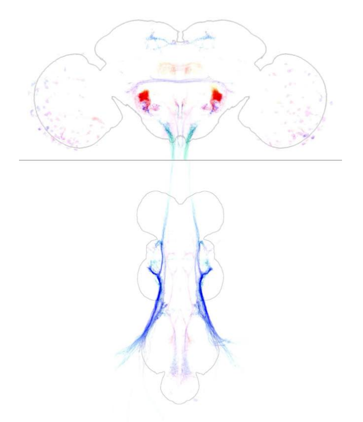
SS52400

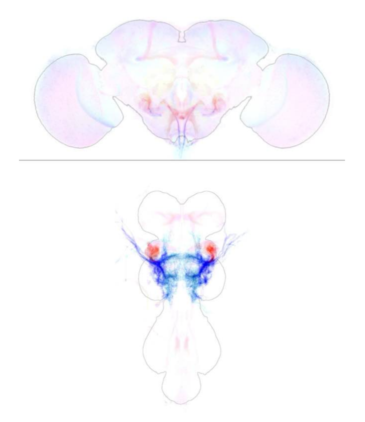
SS52404

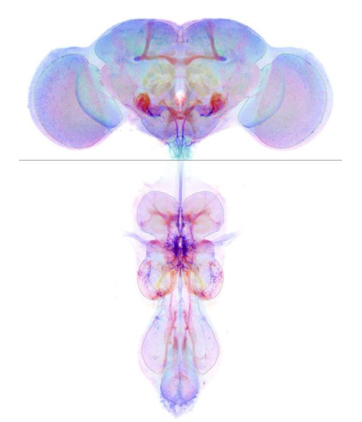
SS52458

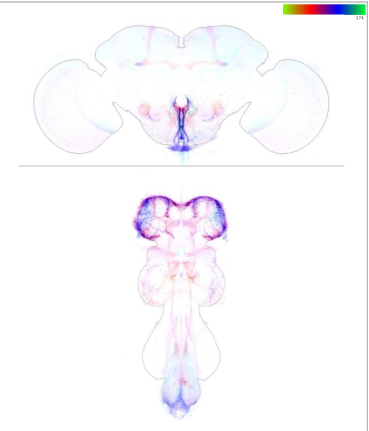
SS52470

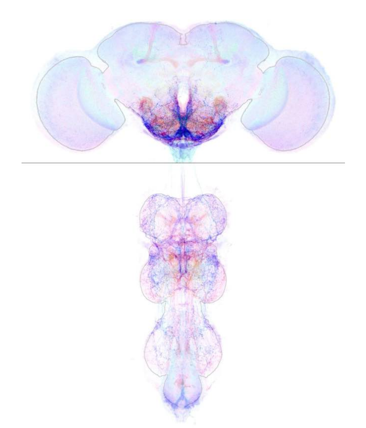
SS52476

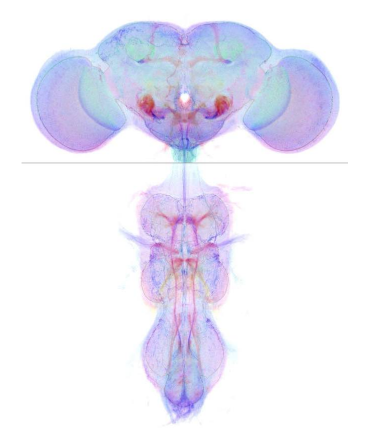
SS52482

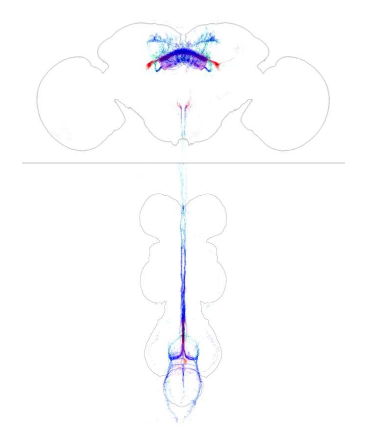
SS52547

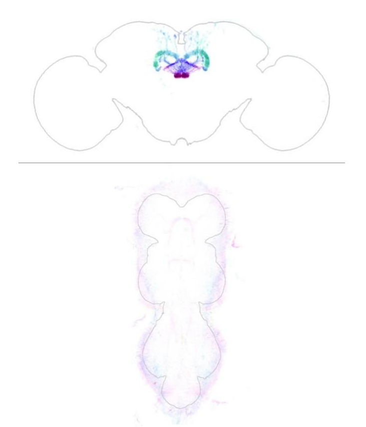
SS52577

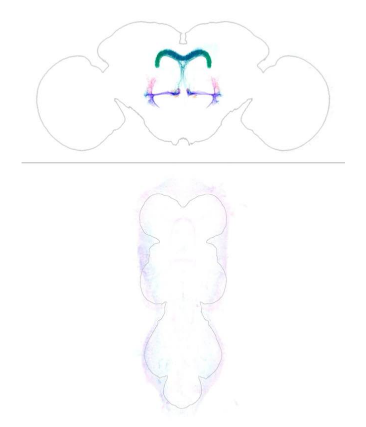
SS52578

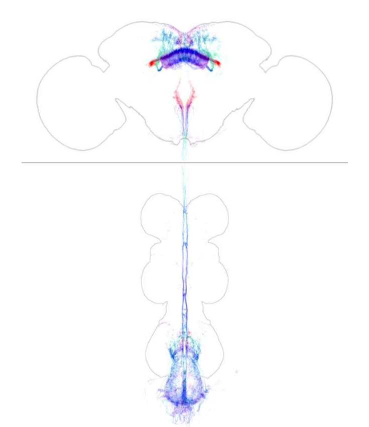
SS52590

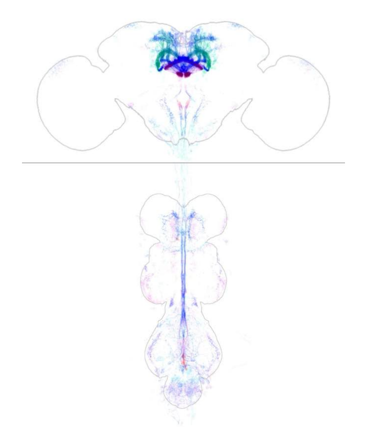
SS52631

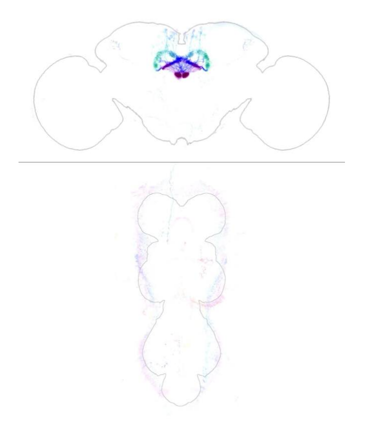
SS52669

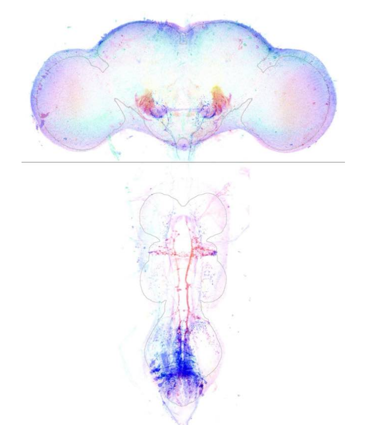
SS52806

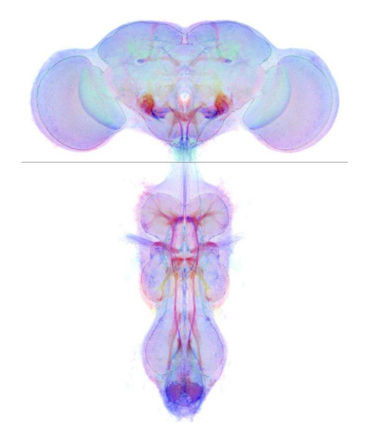
SS52815

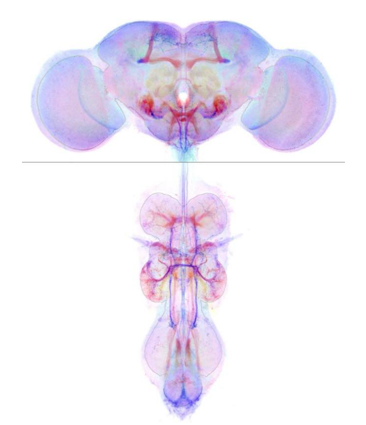
SS52827

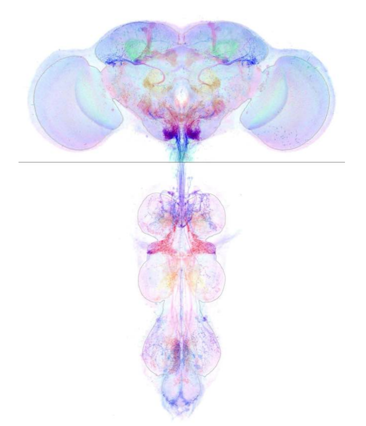
SS52839

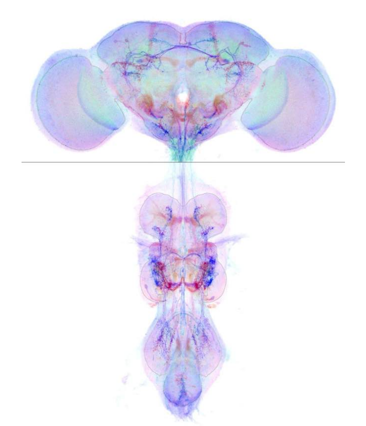
SS52848

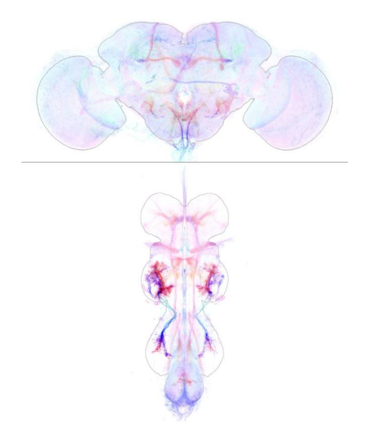
SS52849

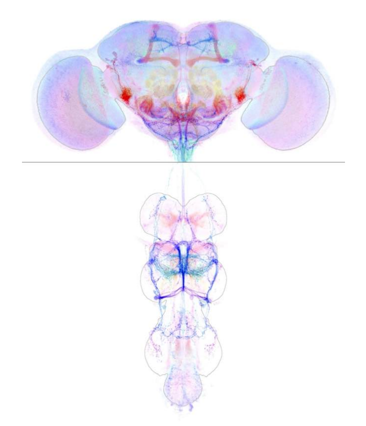
SS52867

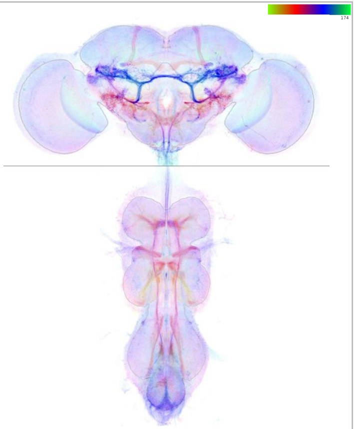
SS52884

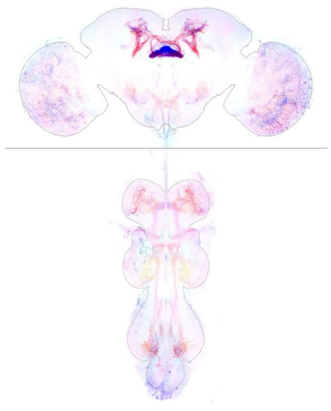
SS52894

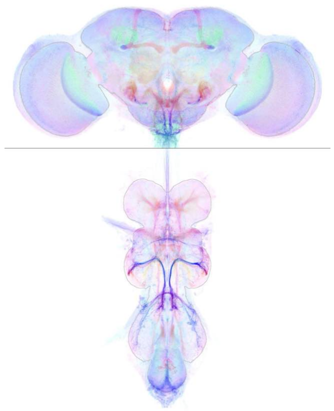
SS52905

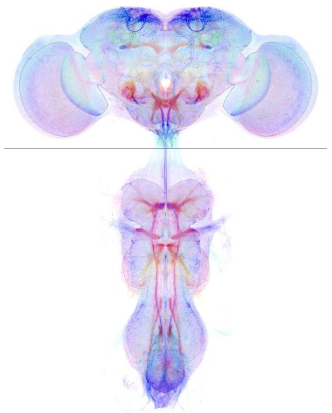
SS52928

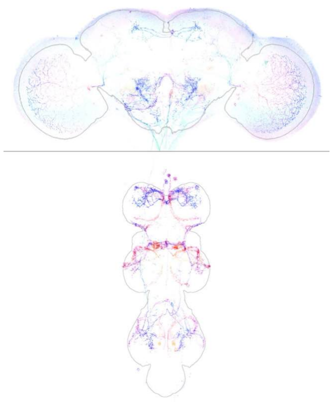
SS53029

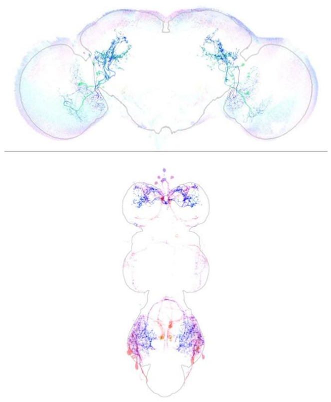
SS53030

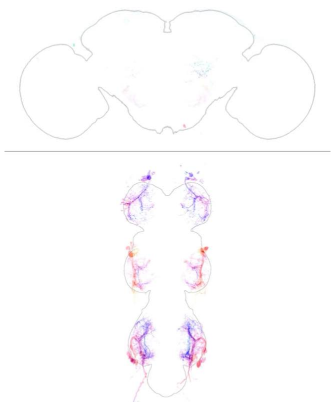
SS53050

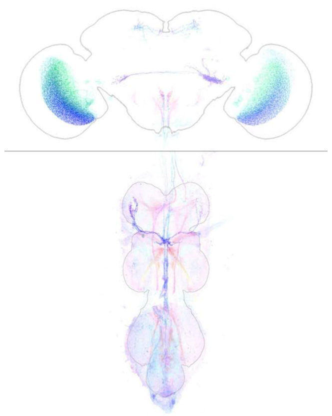
SS53141

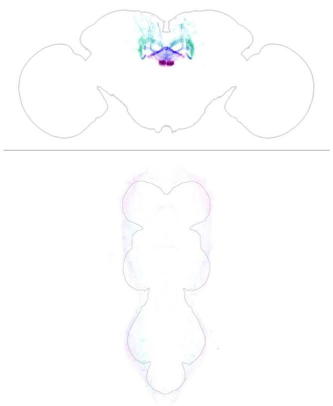
SS53161

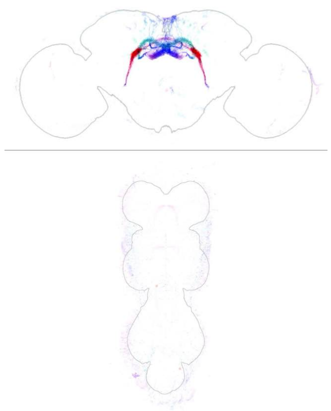
SS53185

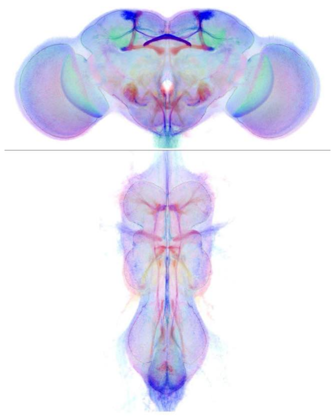
SS53326

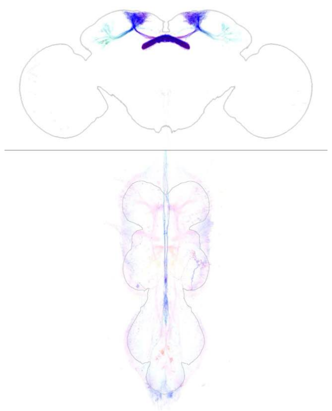
SS53333

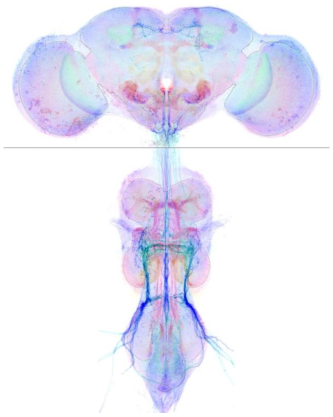
SS53403

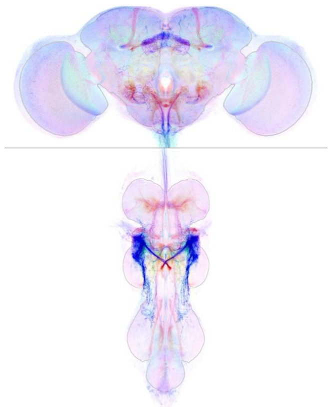
SS53421

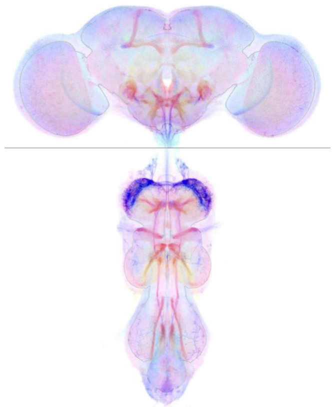
SS53435

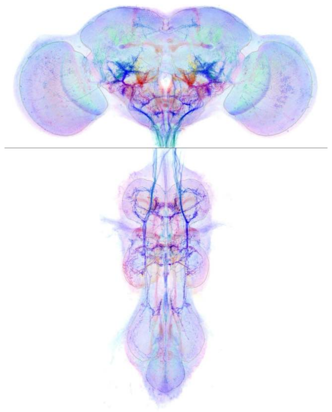
SS53436

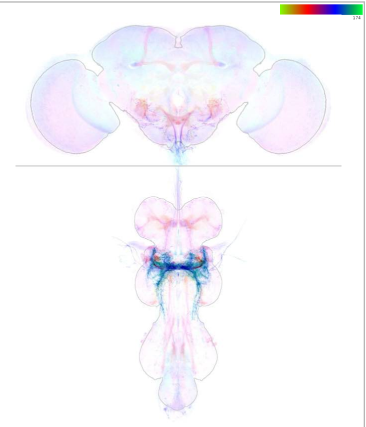

SS53438

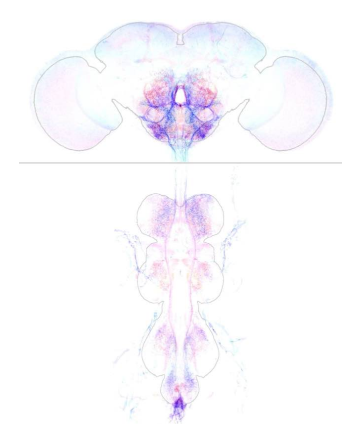

SS53439

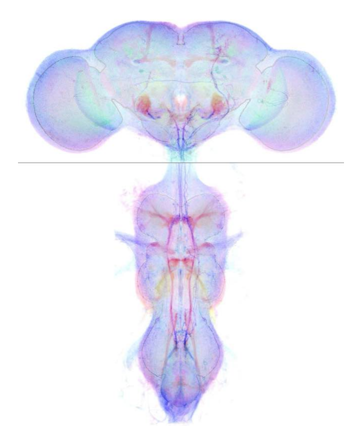

SS53449

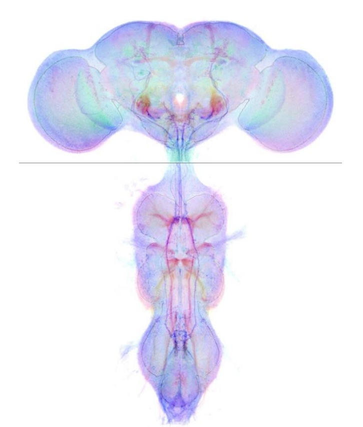

SS53450

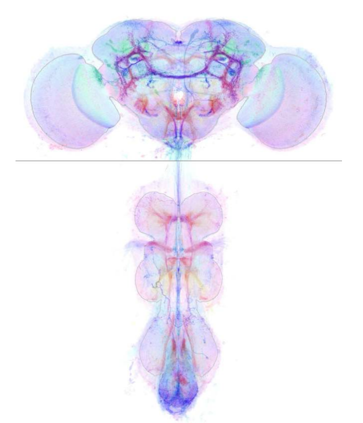

SS53456

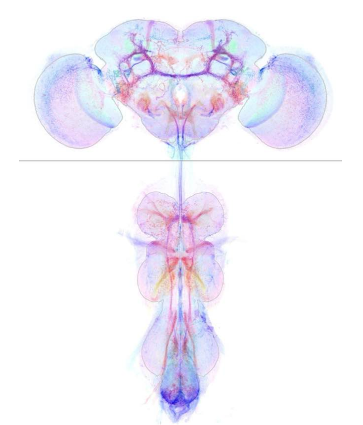

SS53459

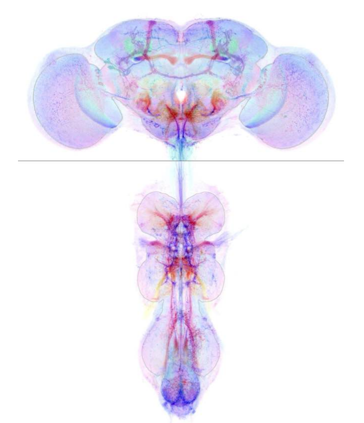

SS53463

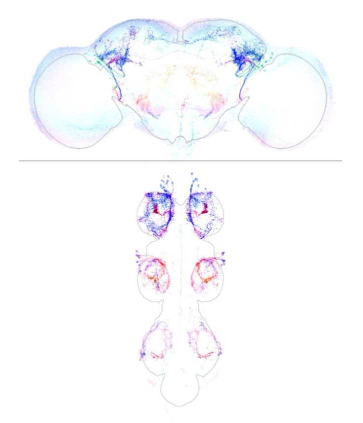

SS53493

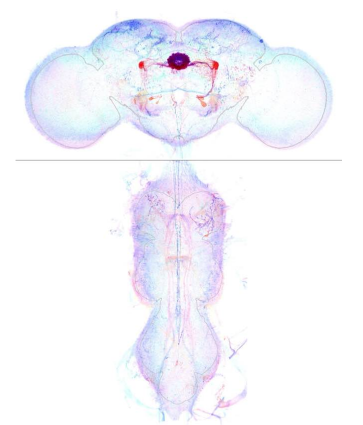

SS53617

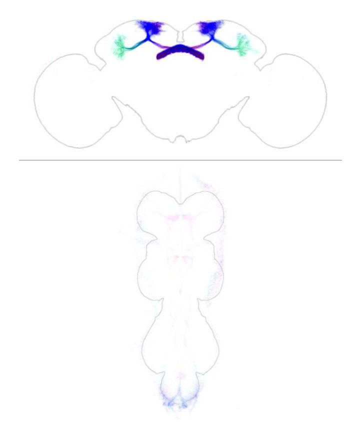

SS53655

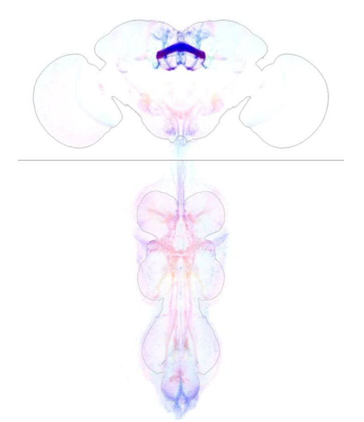

SS53683

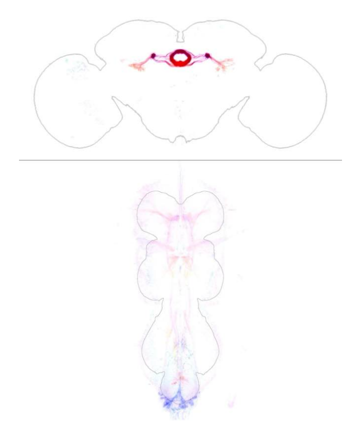

SS53692

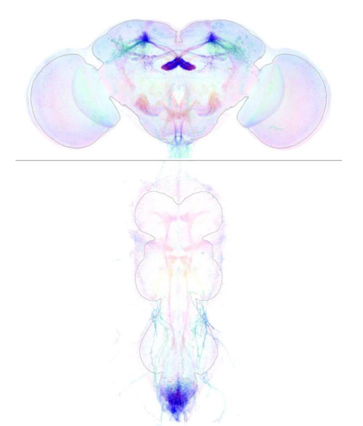

SS53702

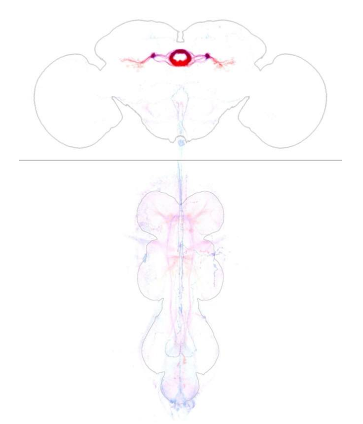

SS53754

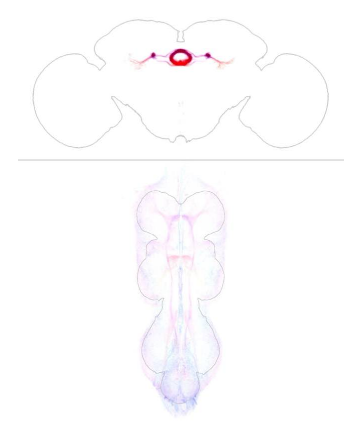

SS53771

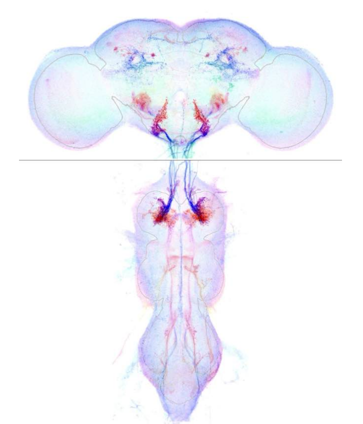

SS53822

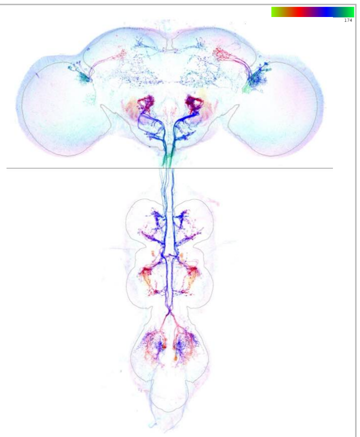
SS53890

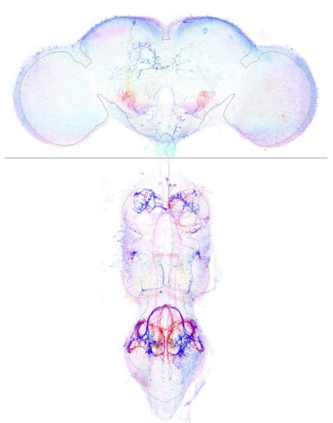
SS53903

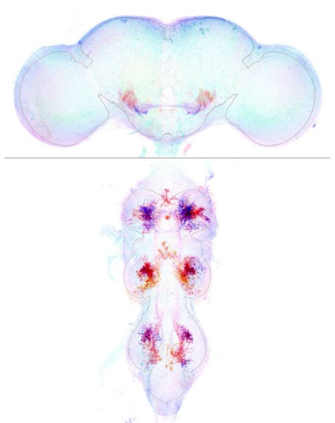
SS53940

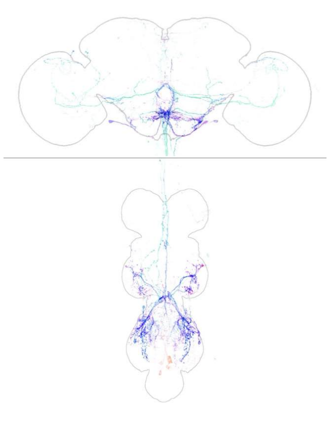
SS54002

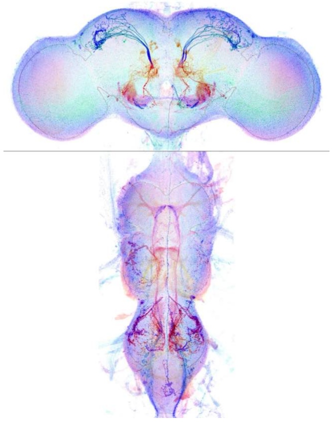
SS54009

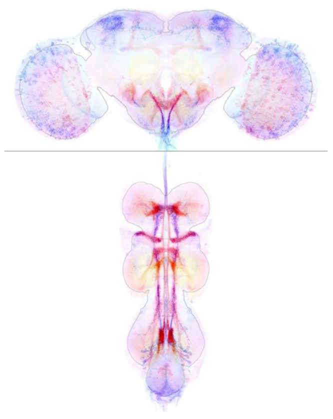
SS54096

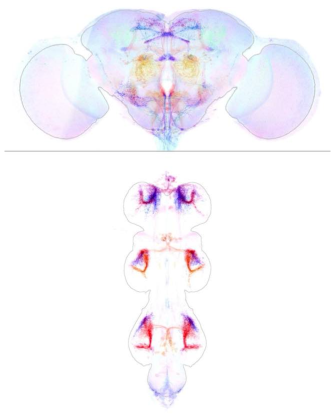
SS54102

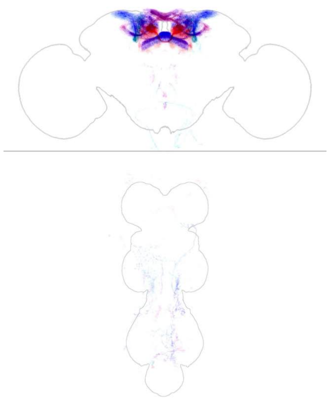
SS54126

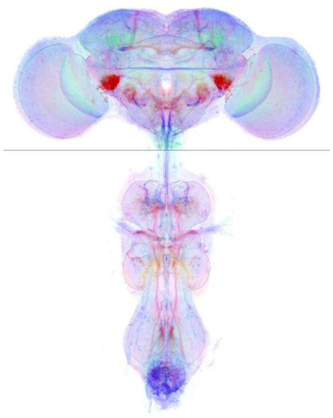
SS54140

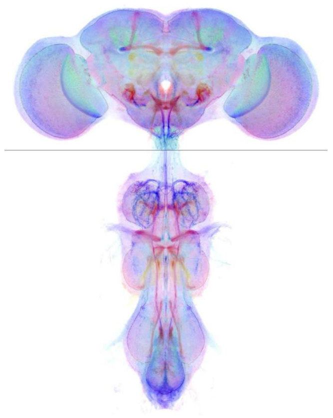
SS54144

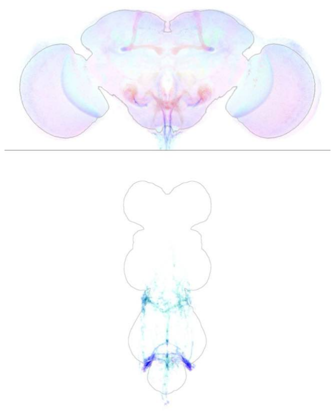
SS54152

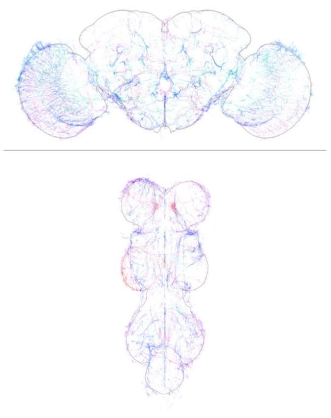
SS54164

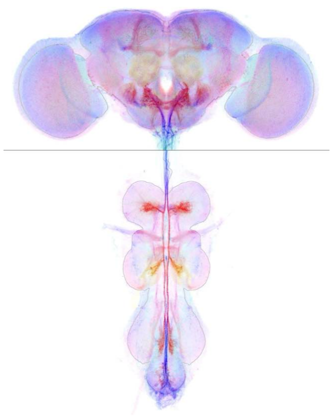
SS54169

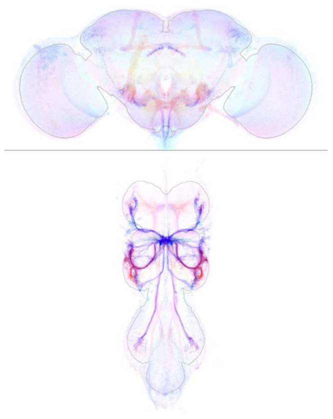
SS54172

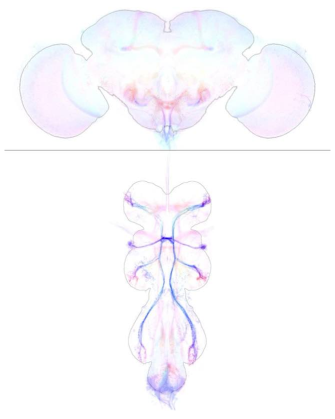
SS54173

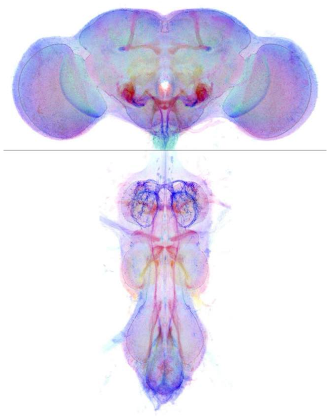
SS54183

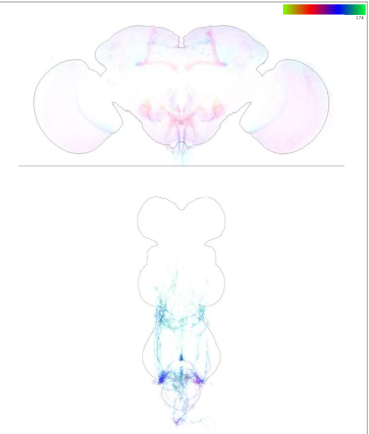
SS54184

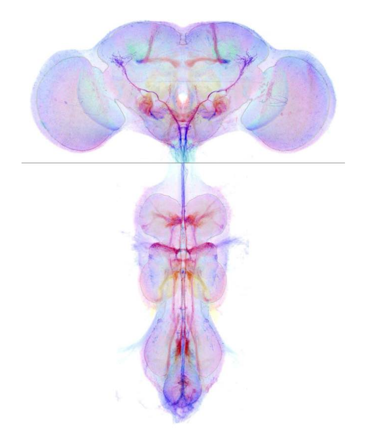
SS54200

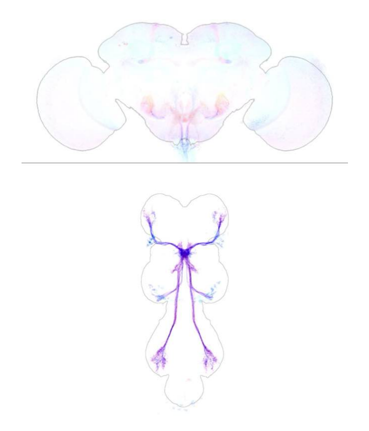
SS54203

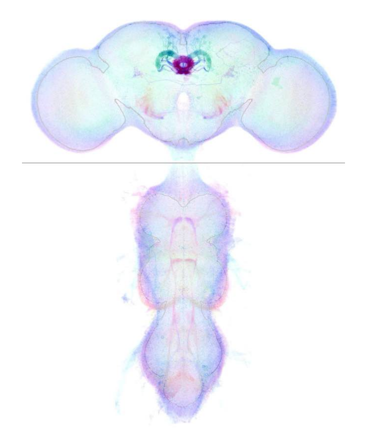
SS54295

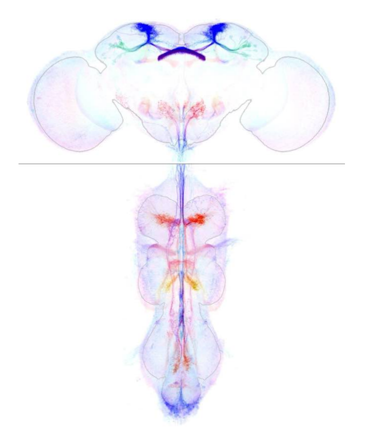
SS54308

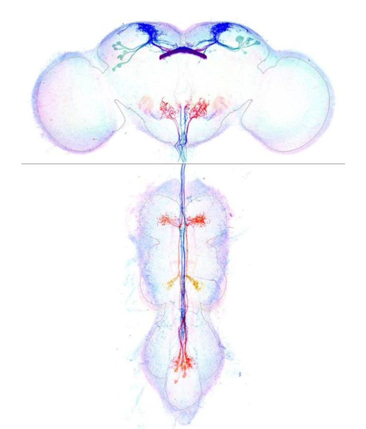
SS54316

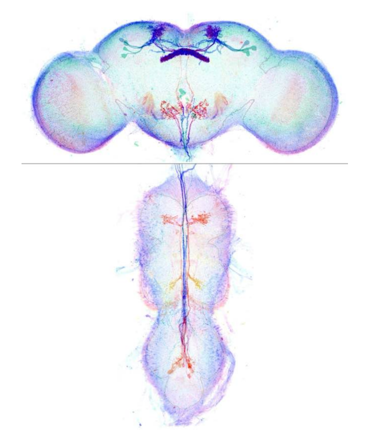
SS54317

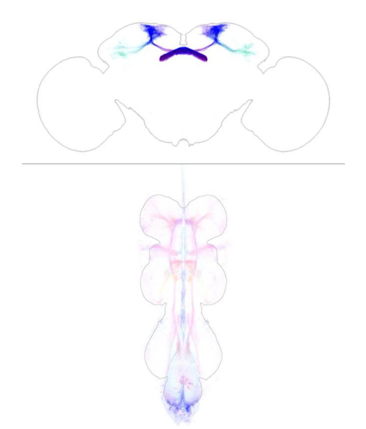
SS54320

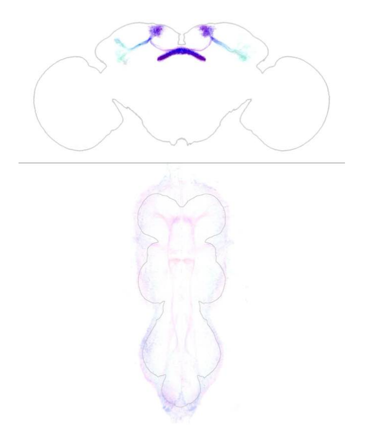
SS54343

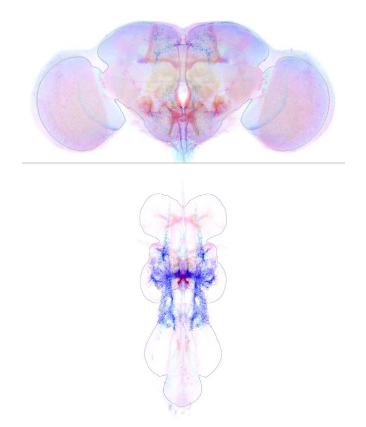
SS54445

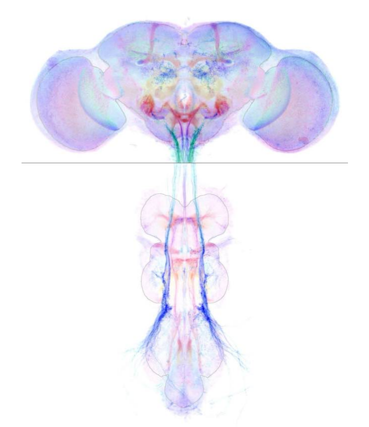
SS54468

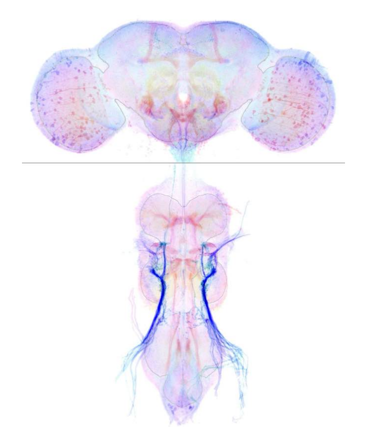
SS54476

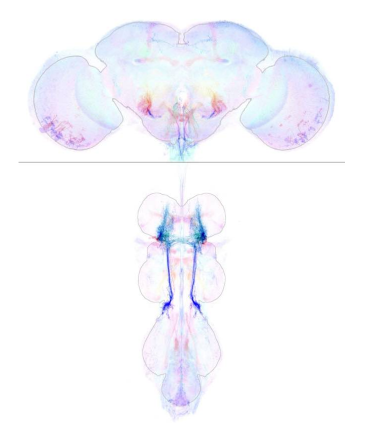
SS54480

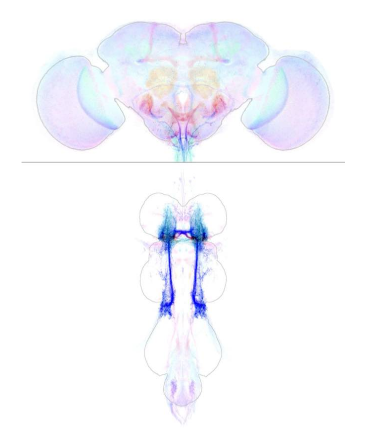
SS54495

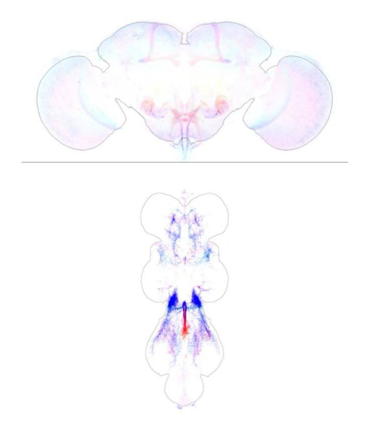
SS54506

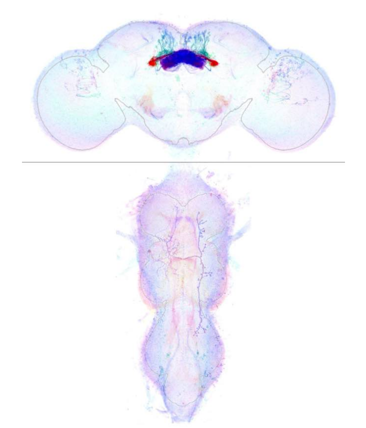
SS54549

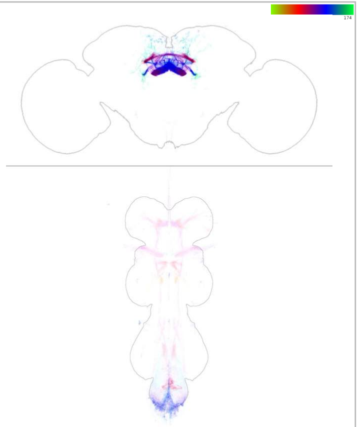

SS54552

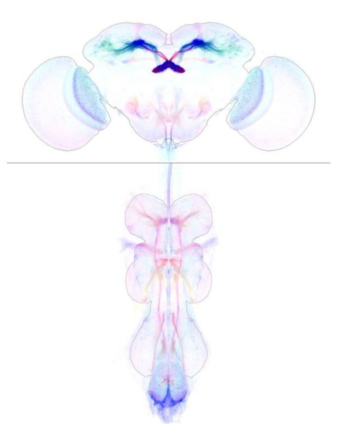

SS54610

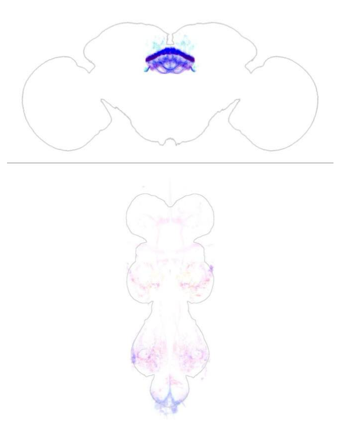

SS54676

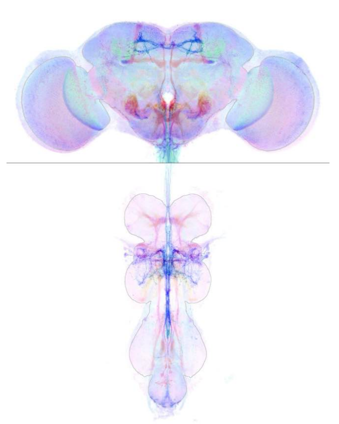

SS54706

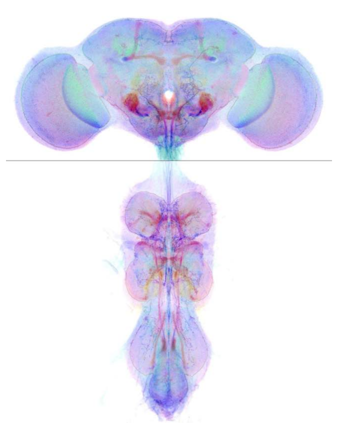

SS54709

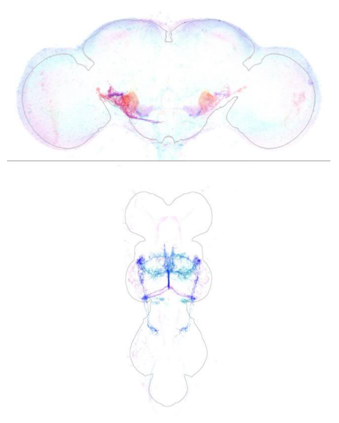

SS54712

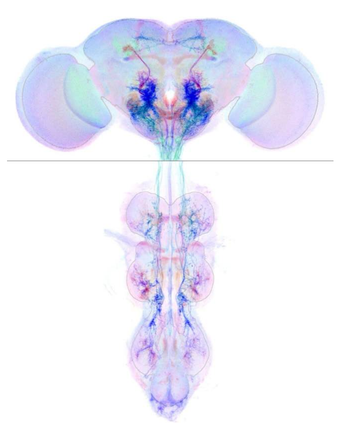

SS54723

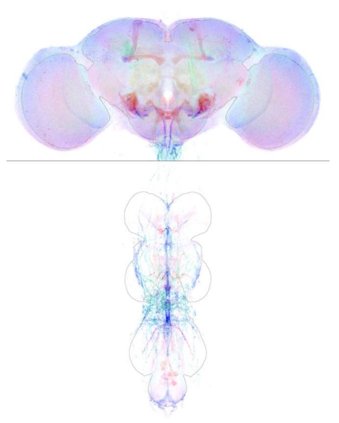

SS54724

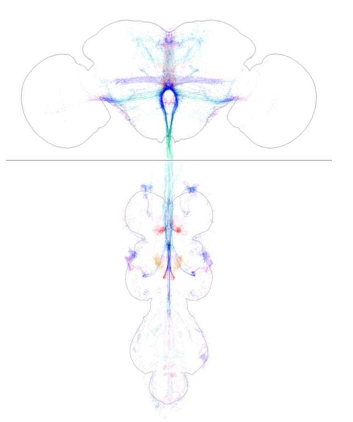

SS54725

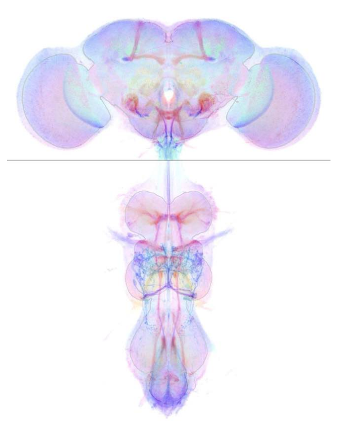

SS54728

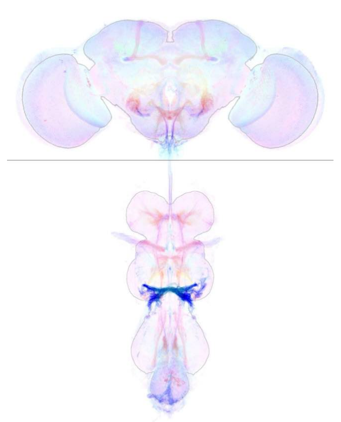

SS54729

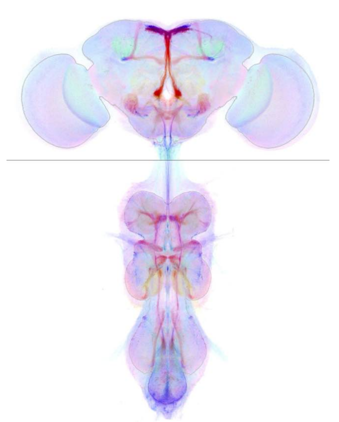

SS54751

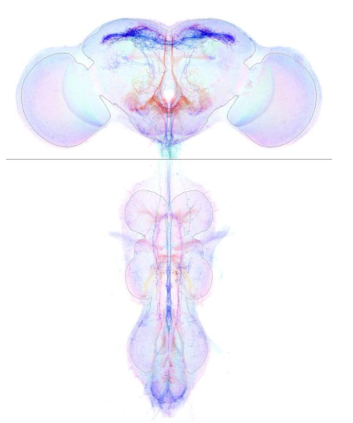

SS54758

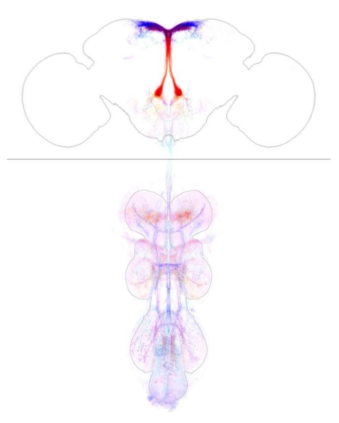

SS54759

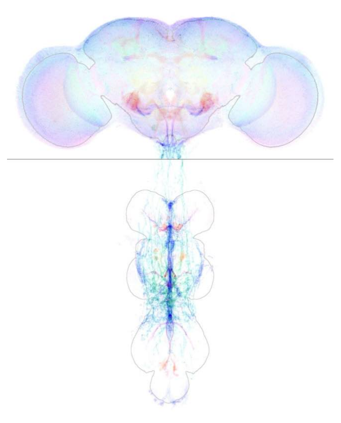

SS54760

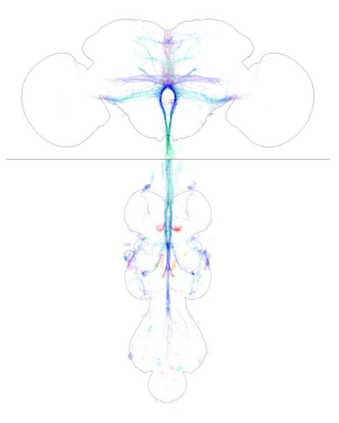

SS54765

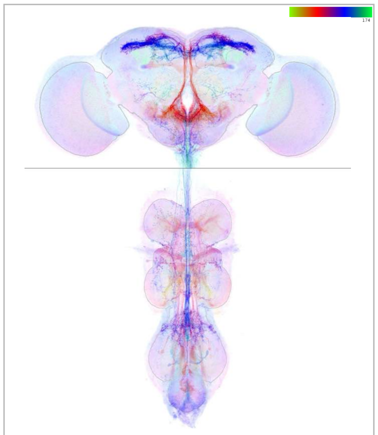
SS54774

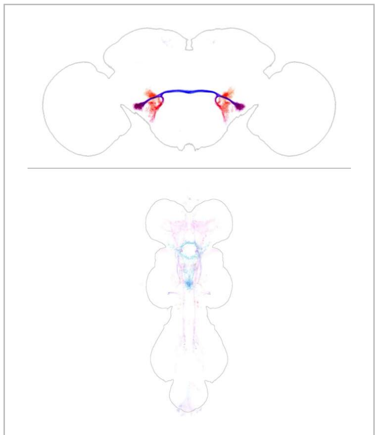
SS54803

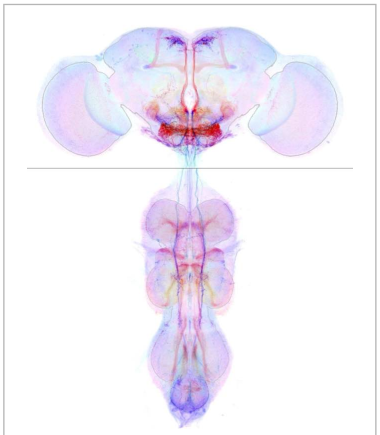
SS54804

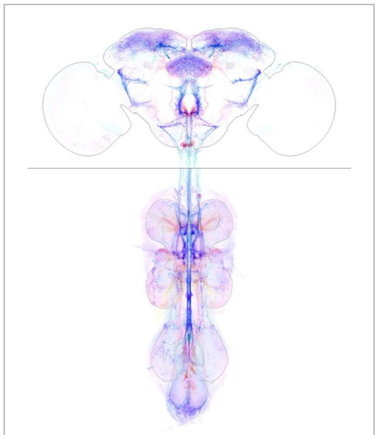
SS54806

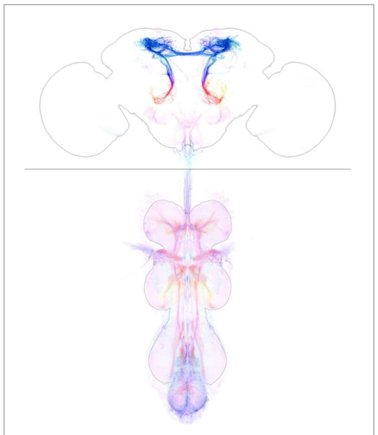
SS54815

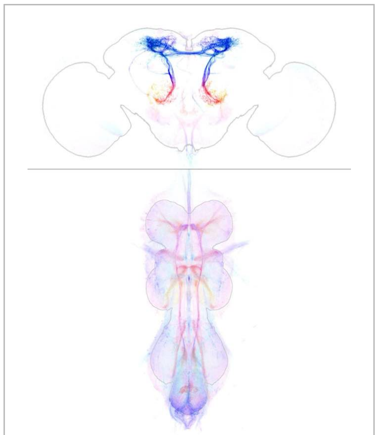
SS54816

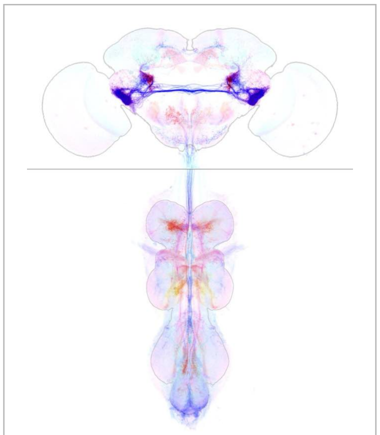
SS54838

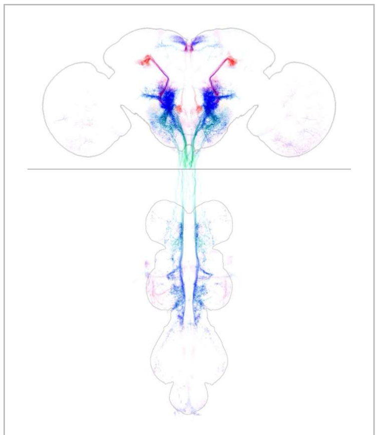
SS54839

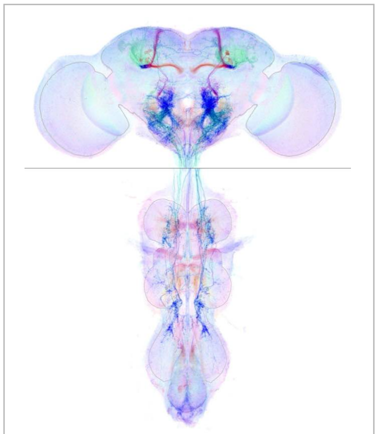
SS54840

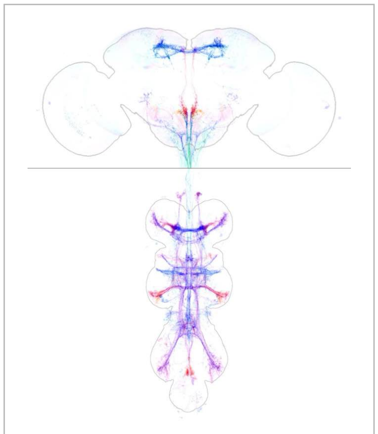
SS54841

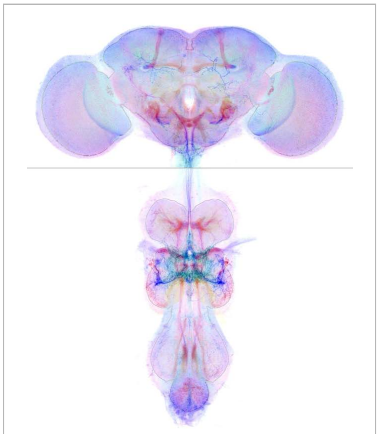
SS54847

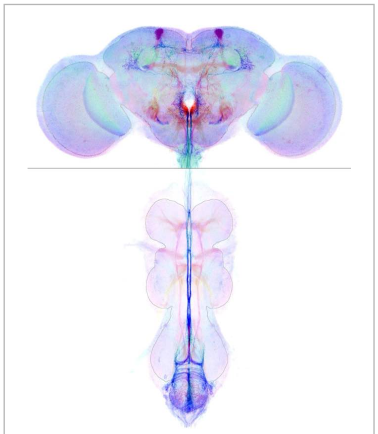
SS54864

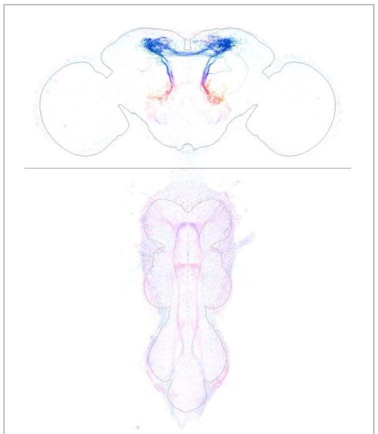
SS54868

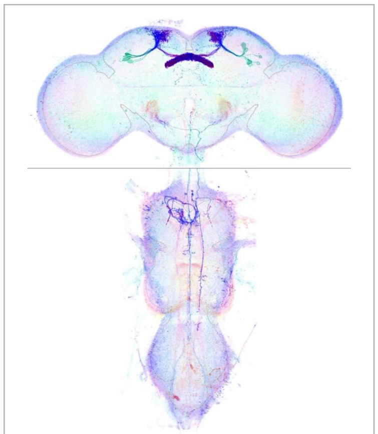
SS54882

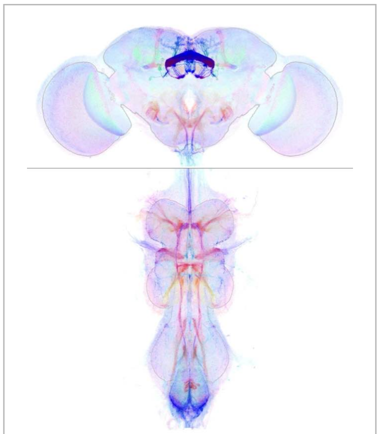
SS54903

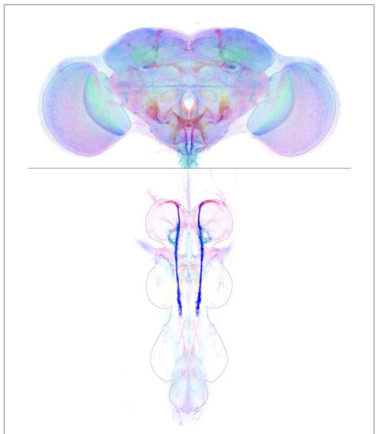
SS54966

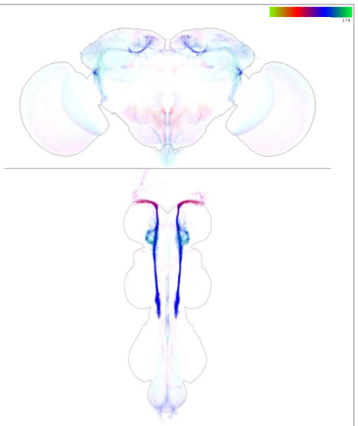
SS54967

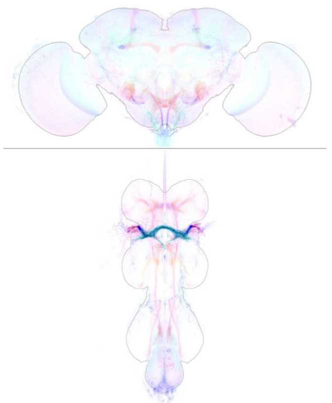
SS54979

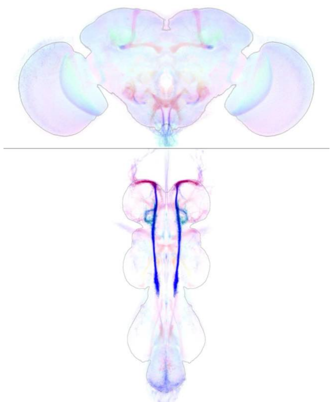
SS54995

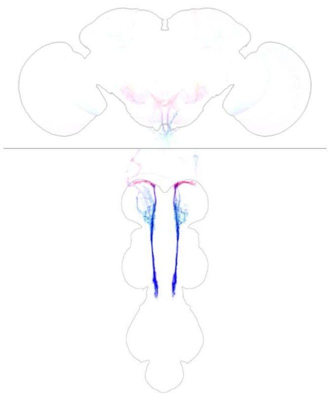
SS54997

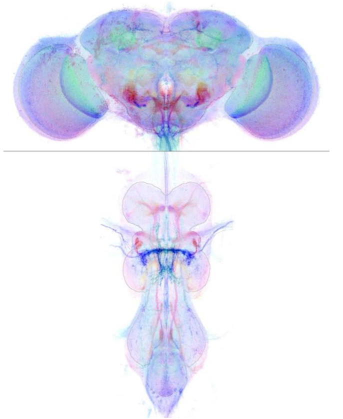
SS55000

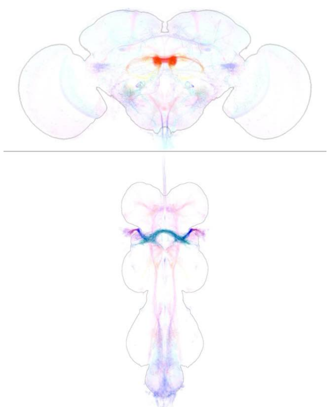
SS55002

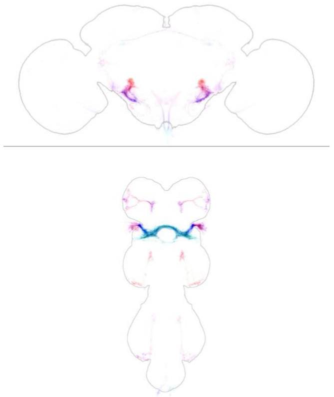
SS55005

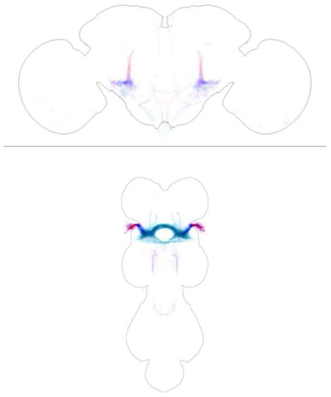
SS55009

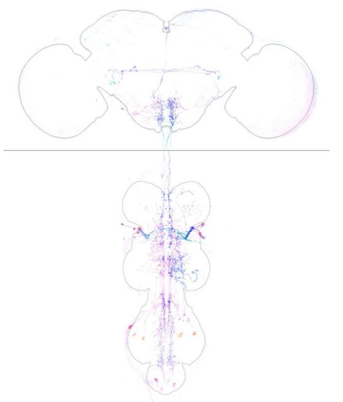
SS55017

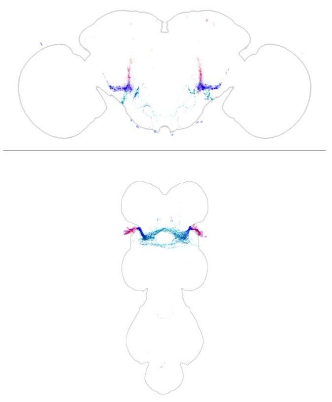
SS55018

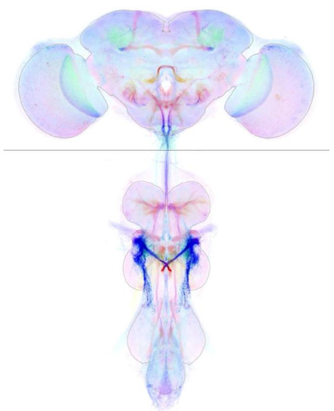
SS55026

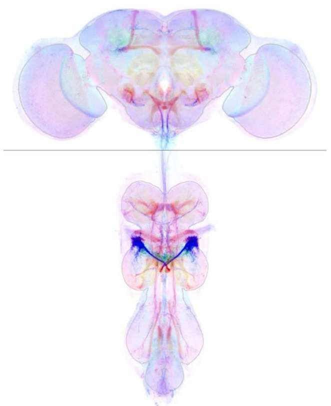
SS55029

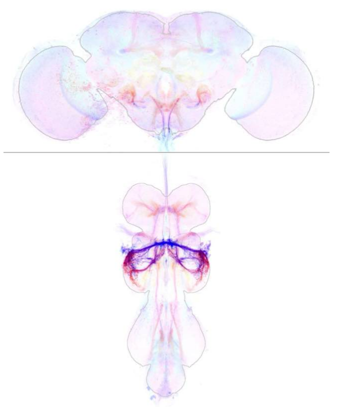
SS55037

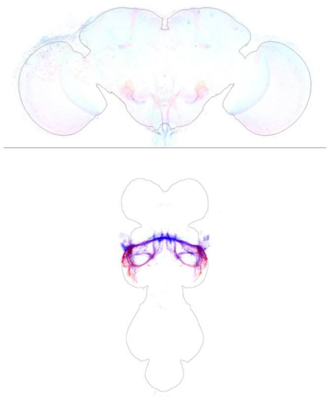
SS55042

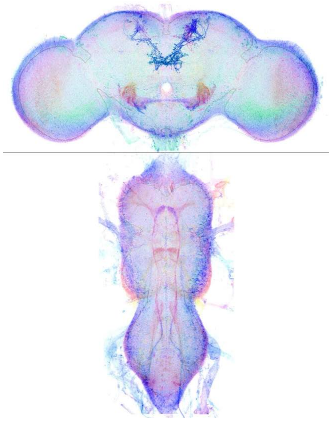
SS55043

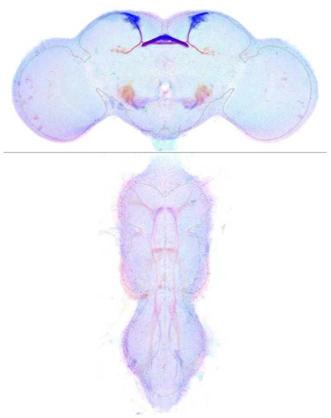
SS55134

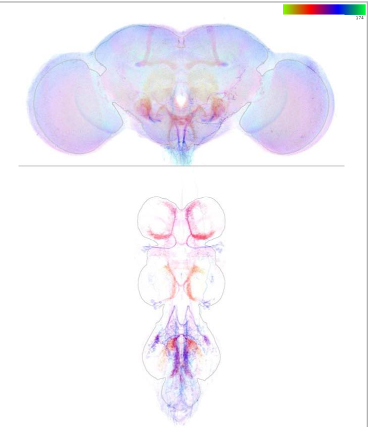
SS55145

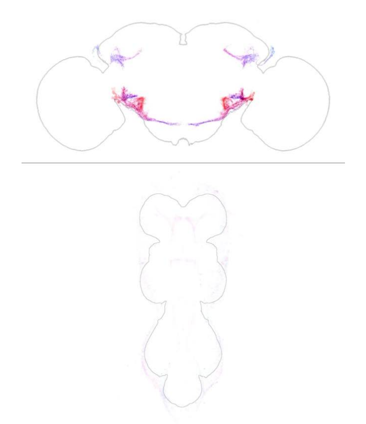
SS55231

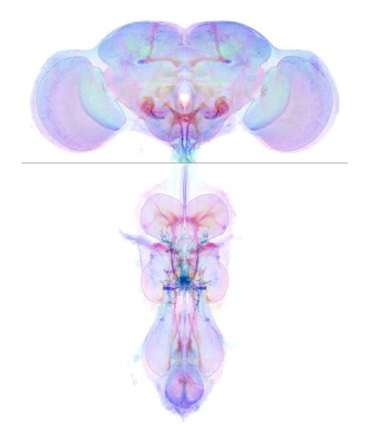
SS55245

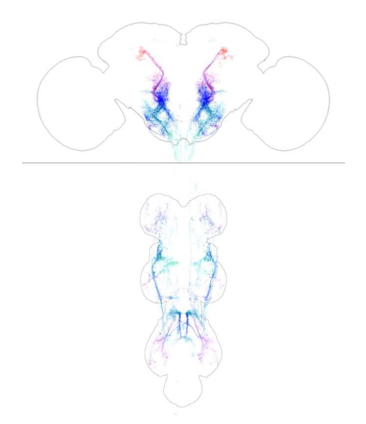
SS55246

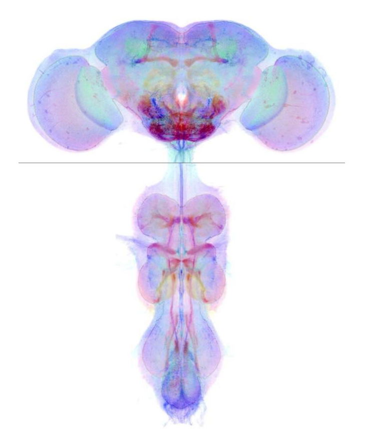
SS55250

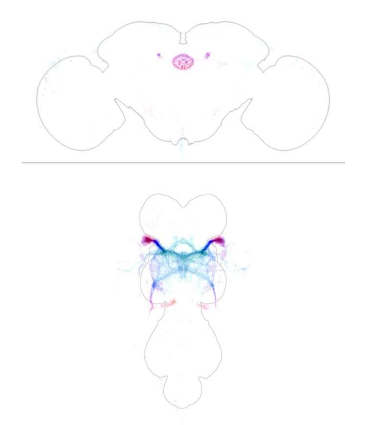
SS55257

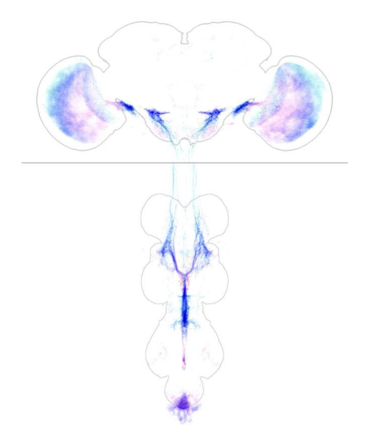
SS55270

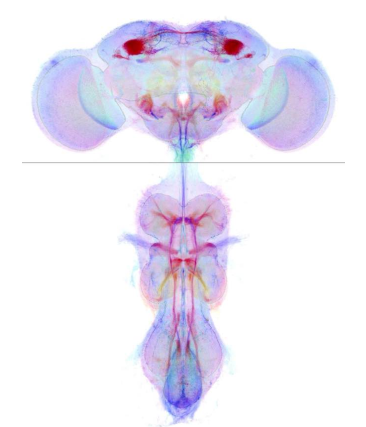
SS55276

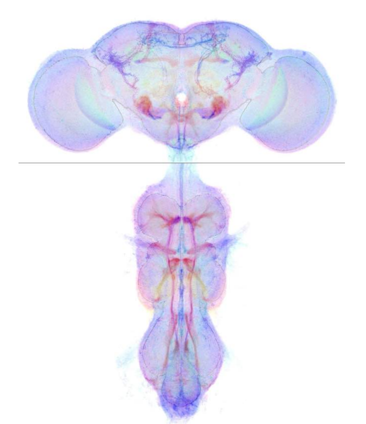
SS55299

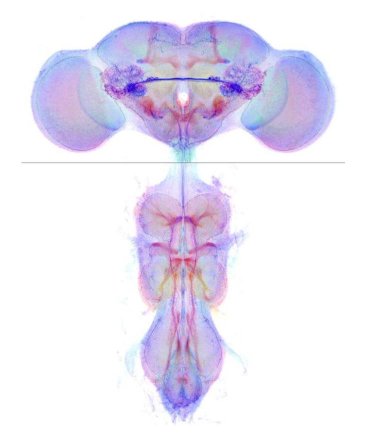
SS55316

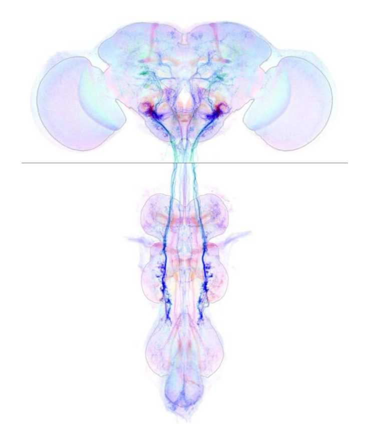
SS55349

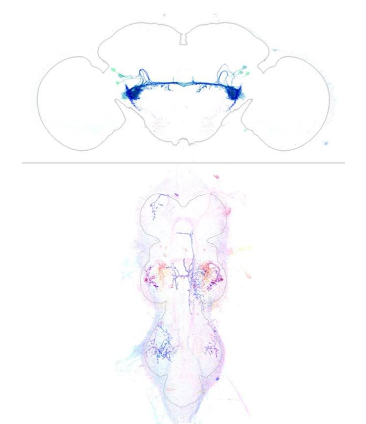
SS55394

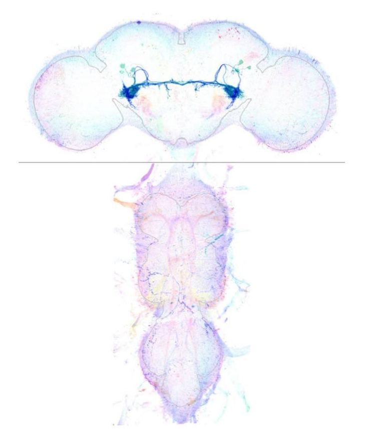
SS55433

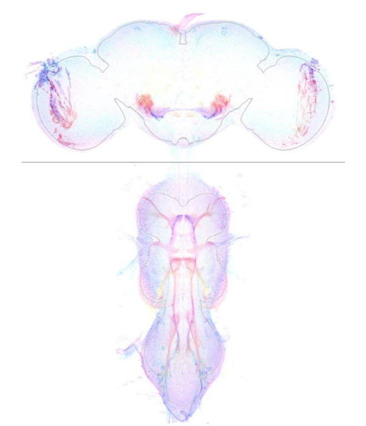
SS55437

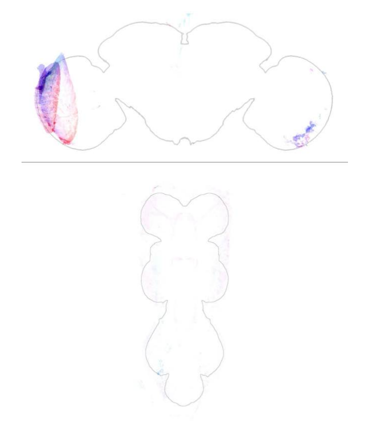
SS55438

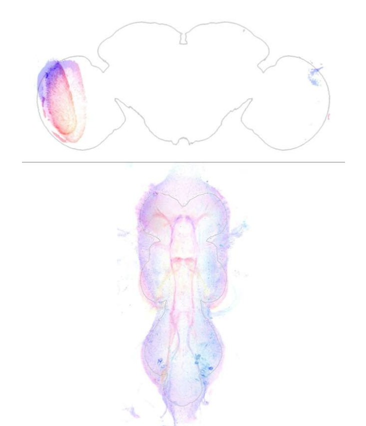
SS55439

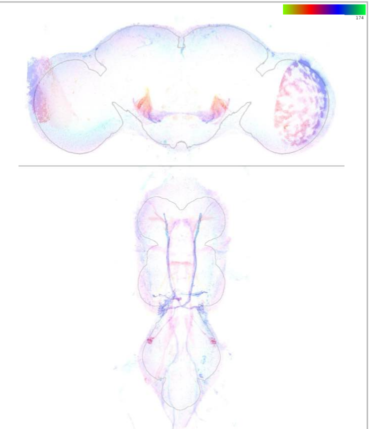
SS55440

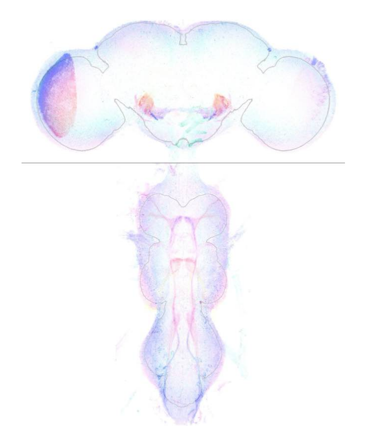
SS55441

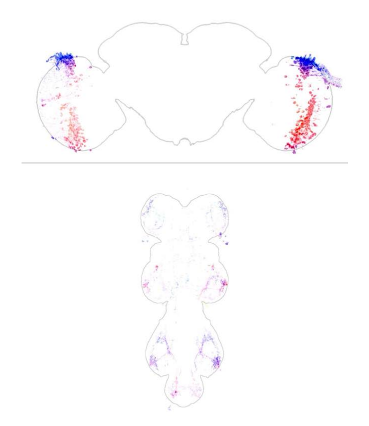
SS55442

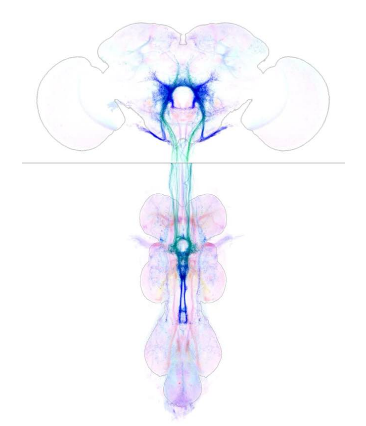
SS55460

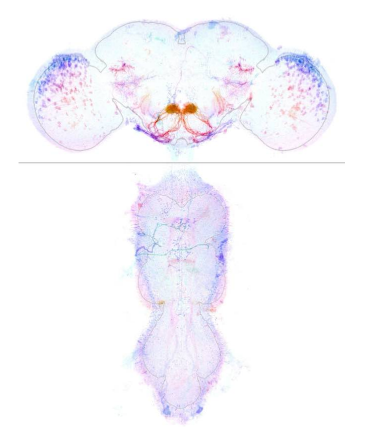
SS55570

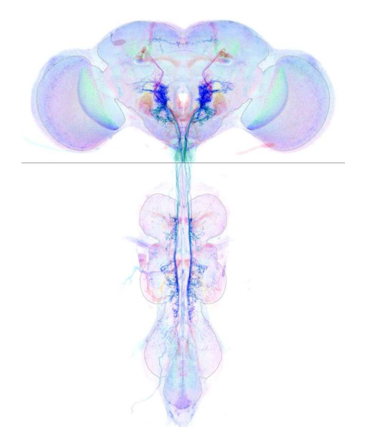
SS55587

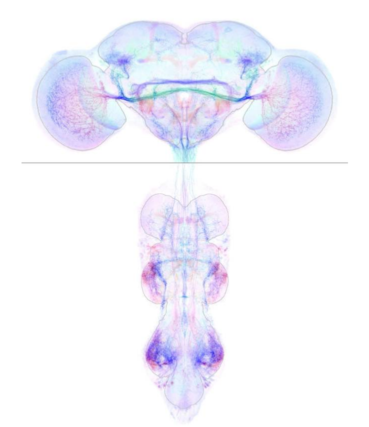
SS55726

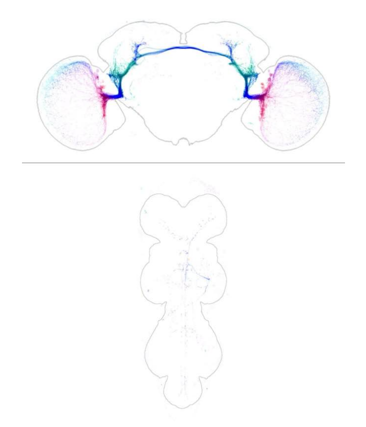
SS55729

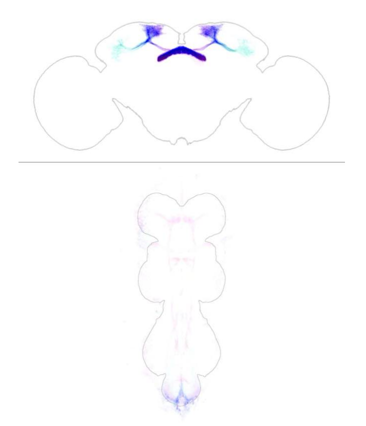
SS55832

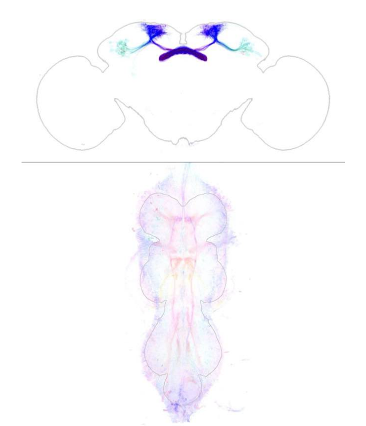
SS55859

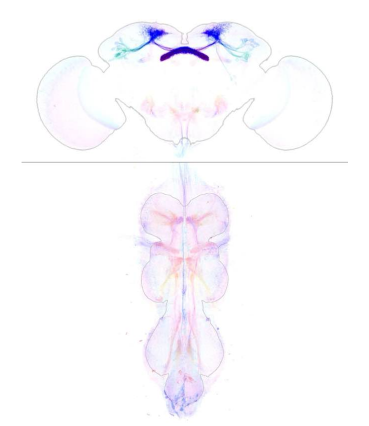
SS55868

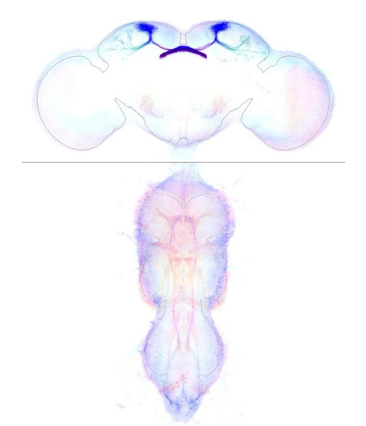
SS55876

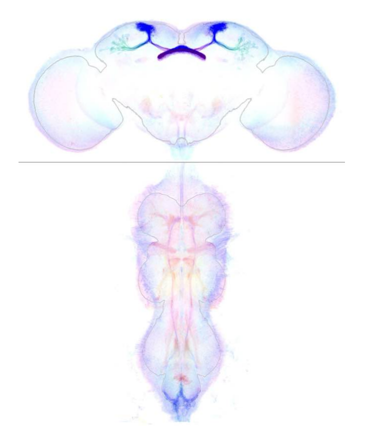
SS55882

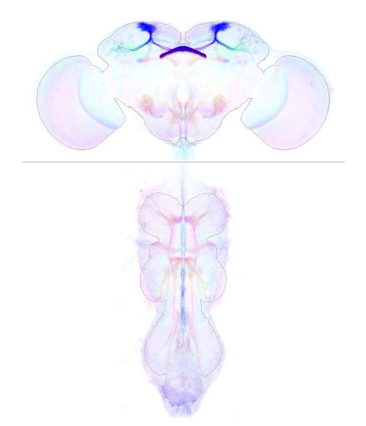
SS55888

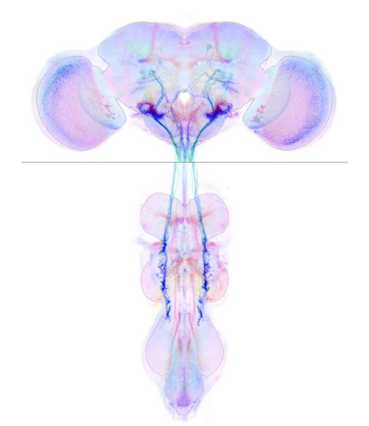
SS55960

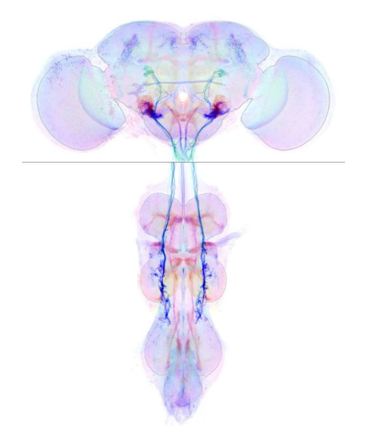
SS55961

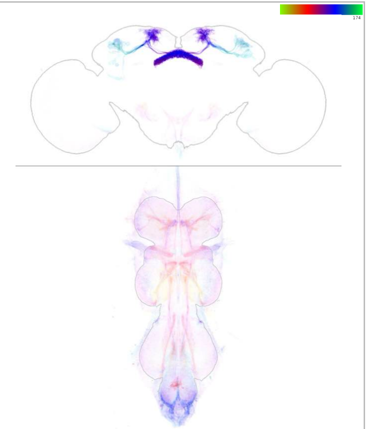
SS56056

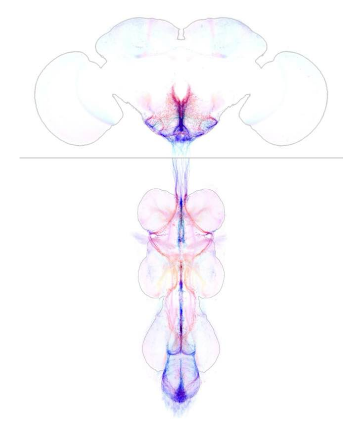
SS56145

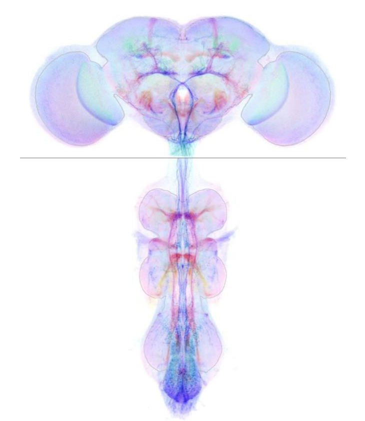
SS56181

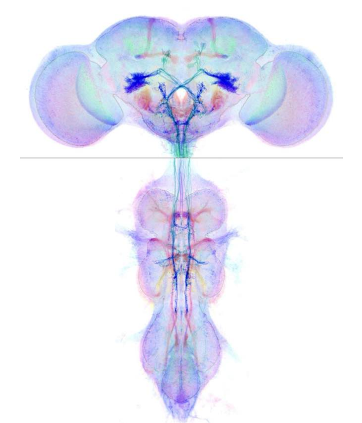
SS56230

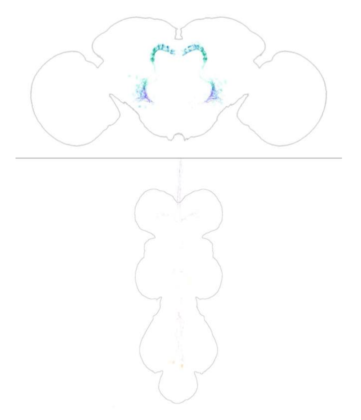
SS56247

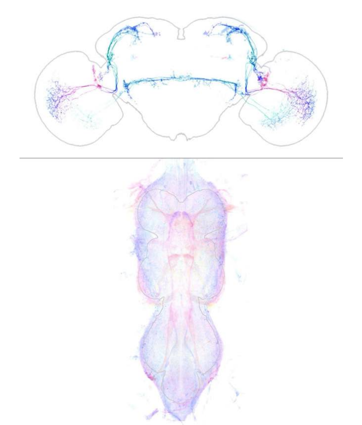
SS56293

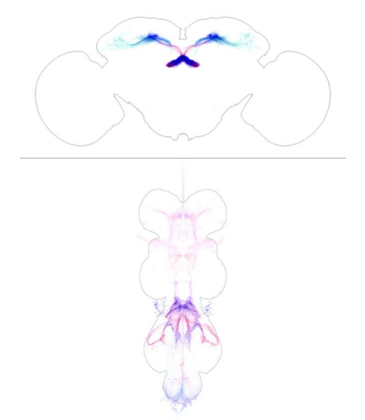
SS56319

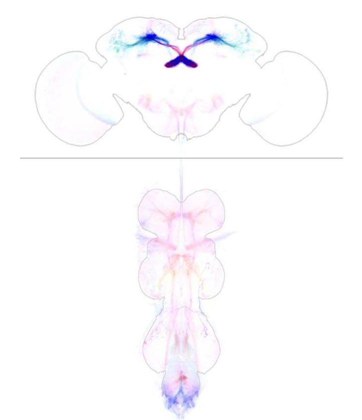
SS56320

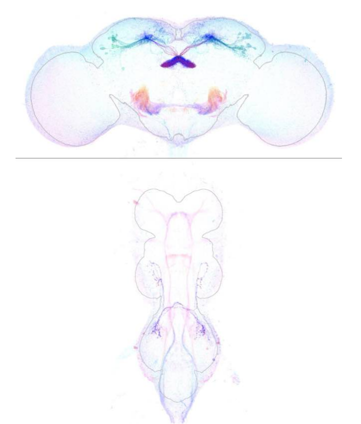
SS56322

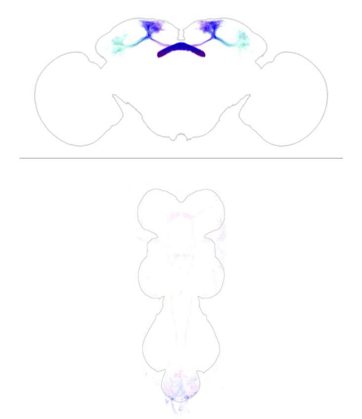
SS56338

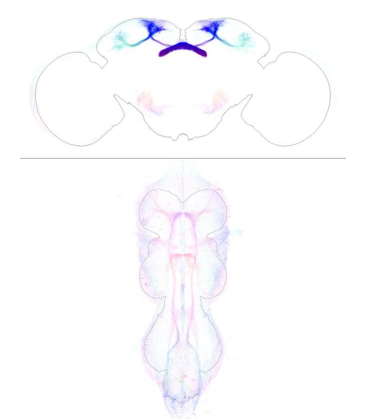
SS56343

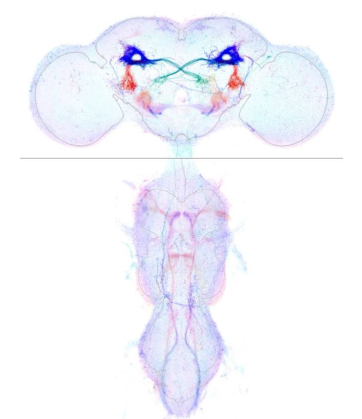
SS56423

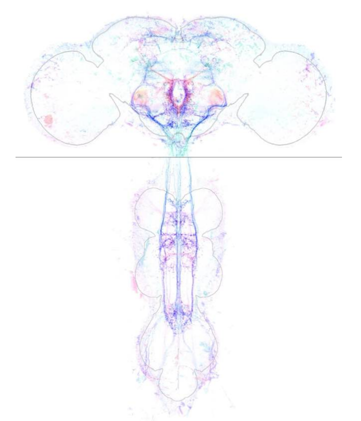
SS56430

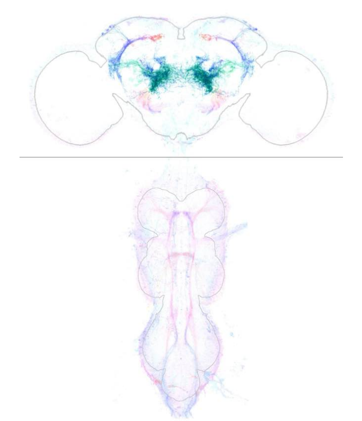
SS56436

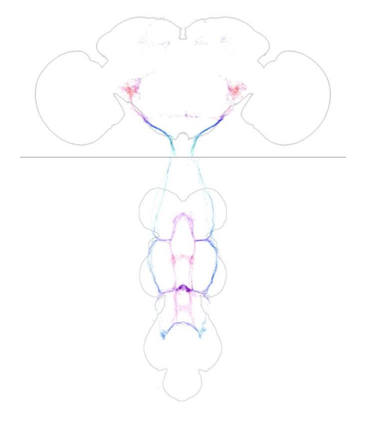
SS56460

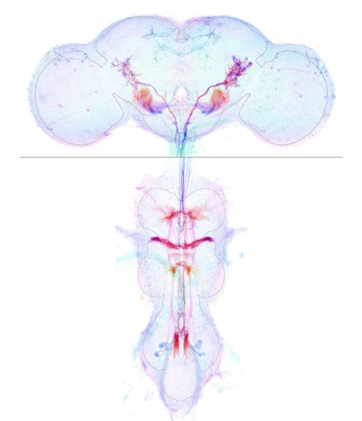
SS56508

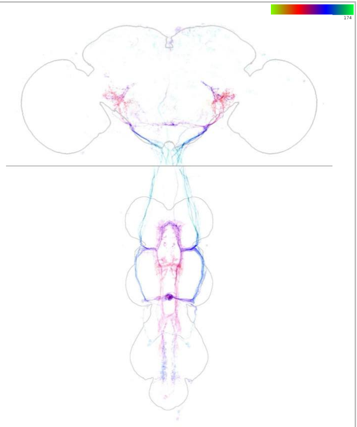
SS56532

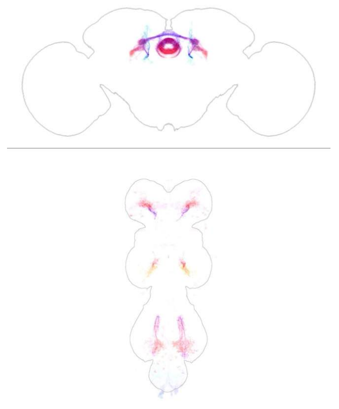
SS56684

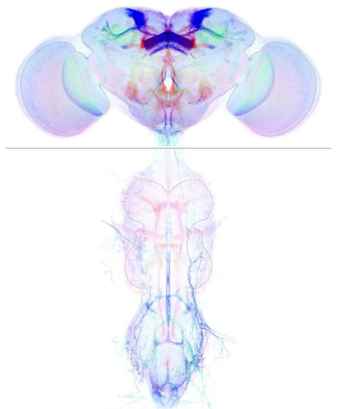
SS56699

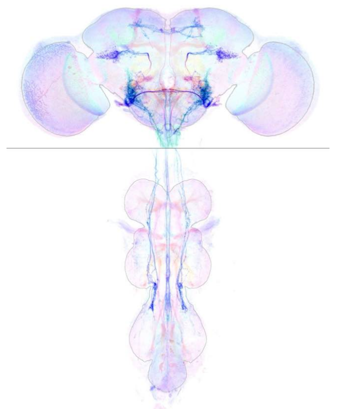
SS56718

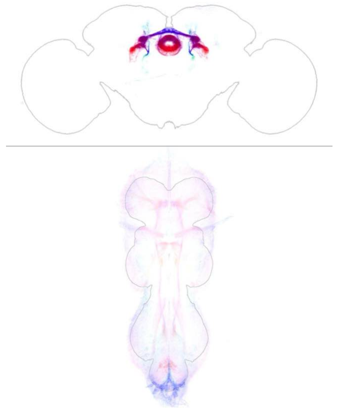
SS56803

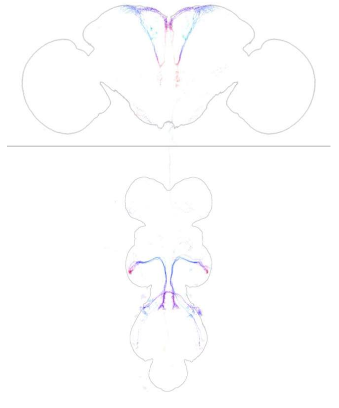
SS56867

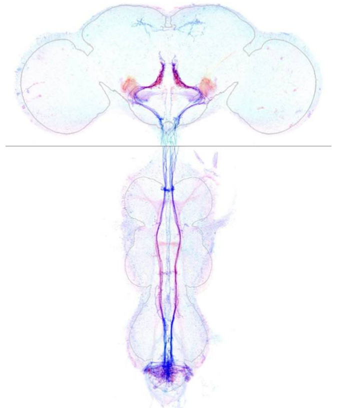
SS56886

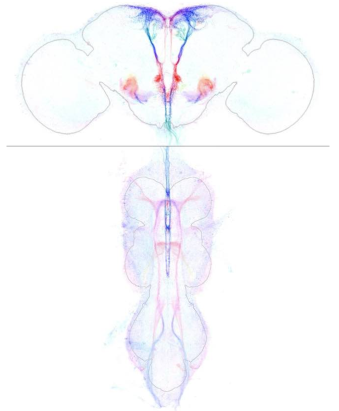
SS56902

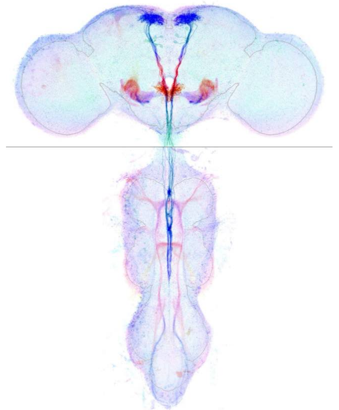
SS56907

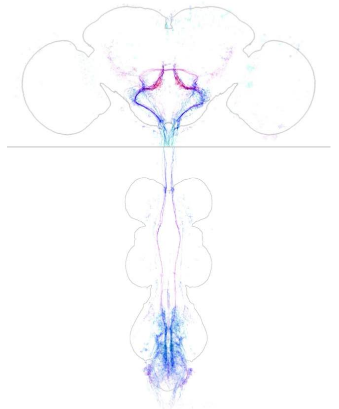
SS56910

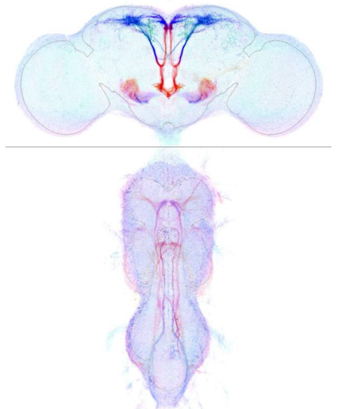
SS56922

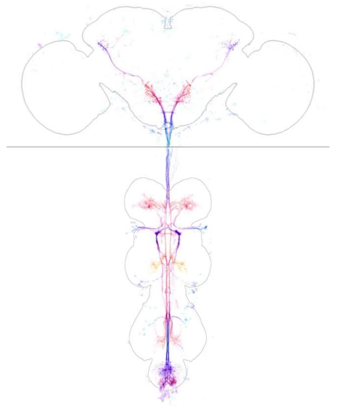
SS56947

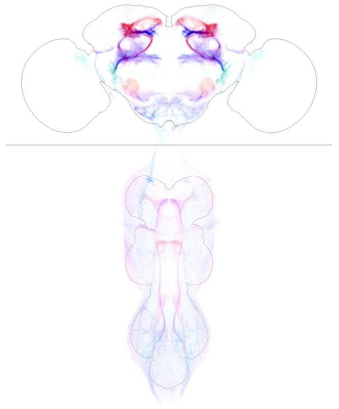
SS56964

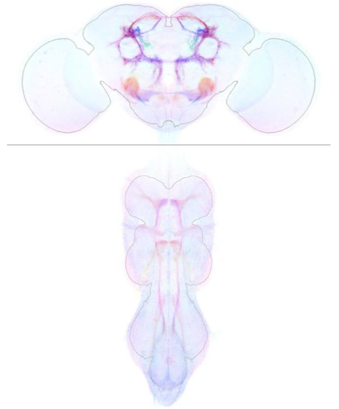
SS56987

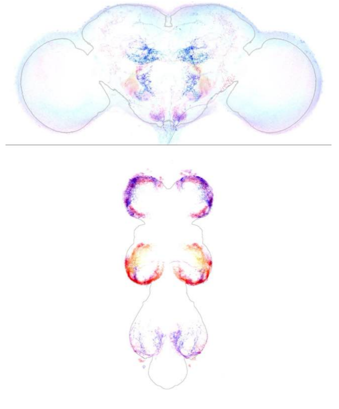
SS56994

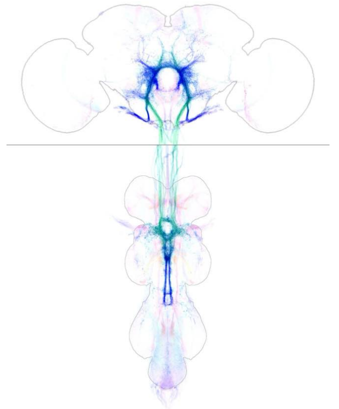
SS56995

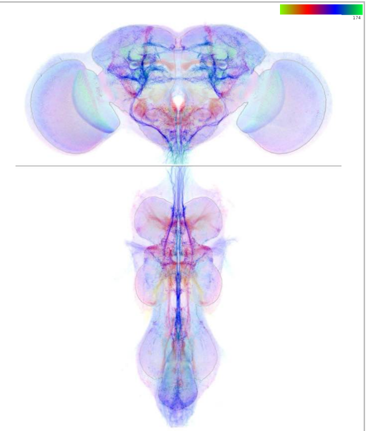
SS57049

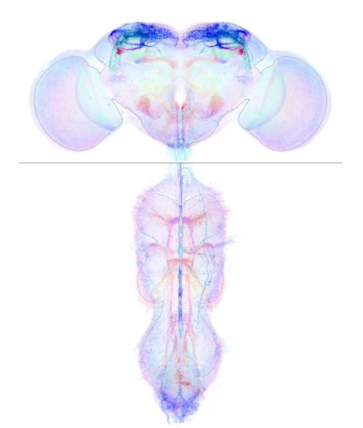
SS57167

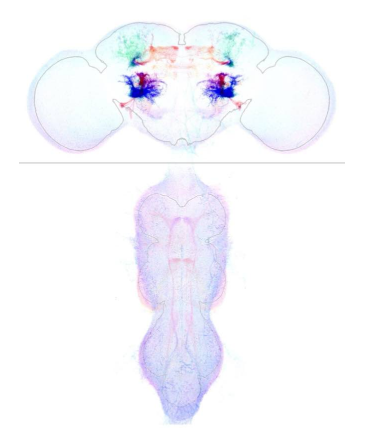
SS57179

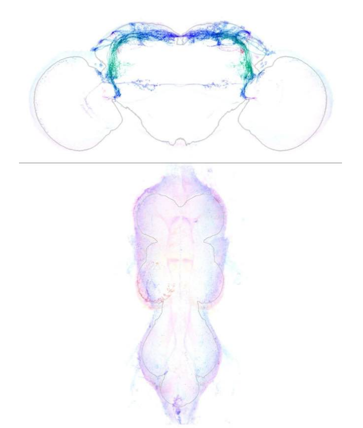
SS57193

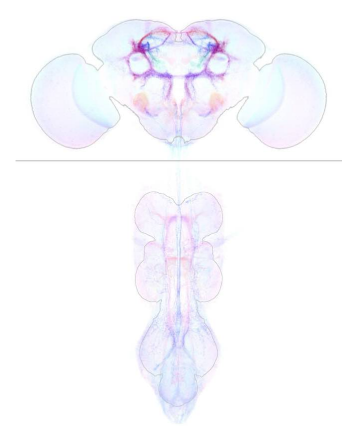
SS57598

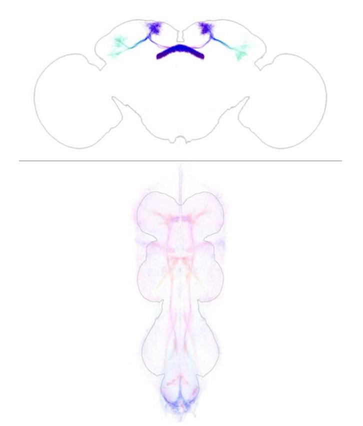
SS57656

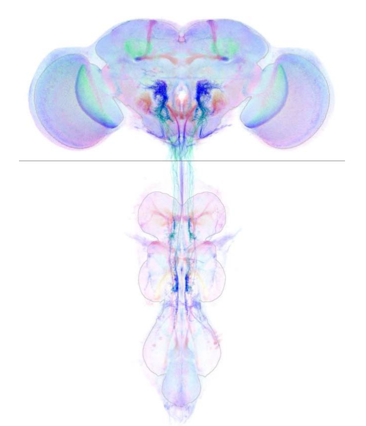
SS57798

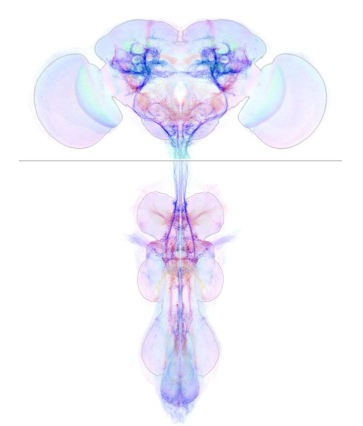
SS57807

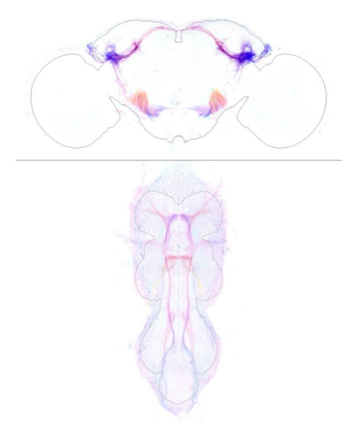
SS57871

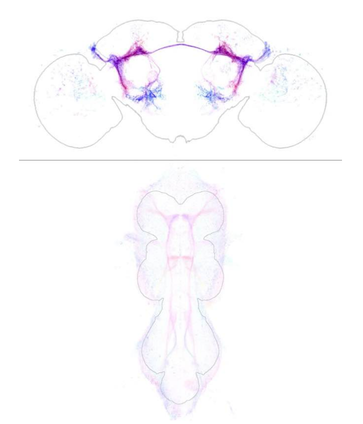
SS57873

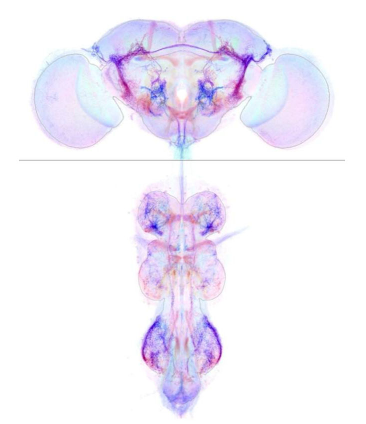
SS57877

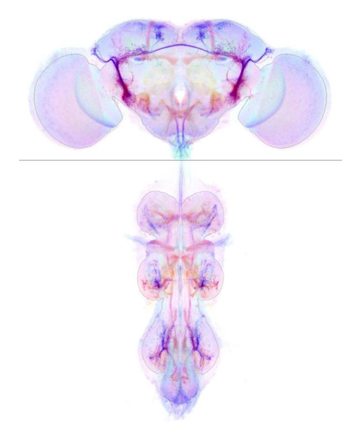
SS57878

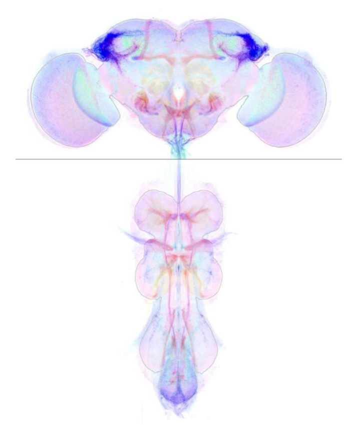
SS57880

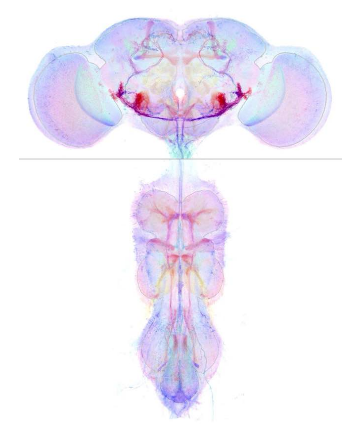
SS57881

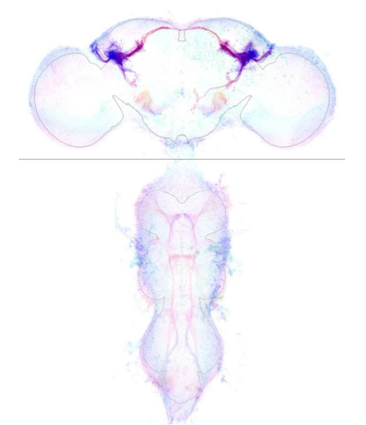
SS57882

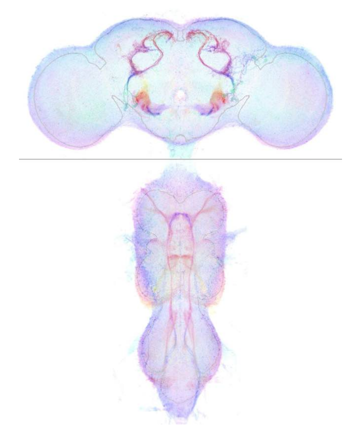
SS57883

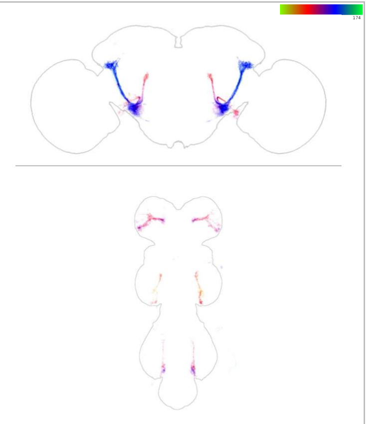
SS57886

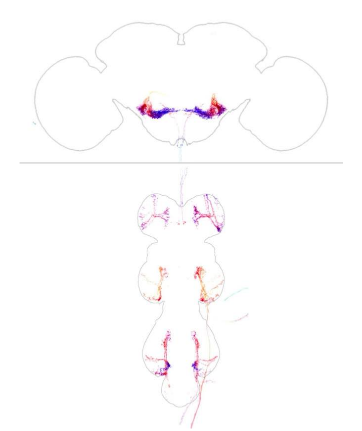
SS57933

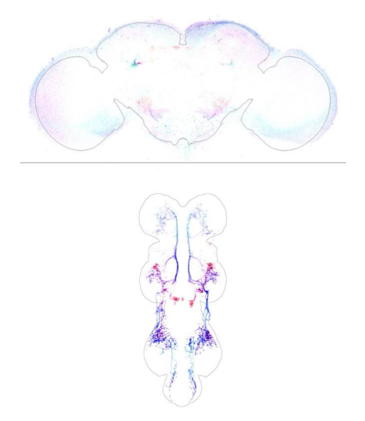
SS57939

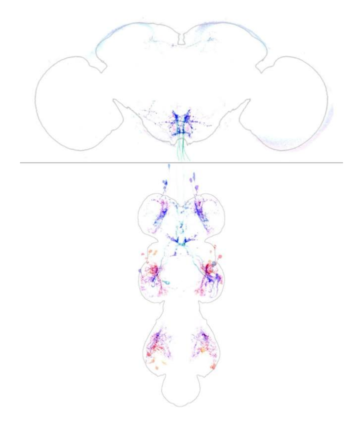
SS57941

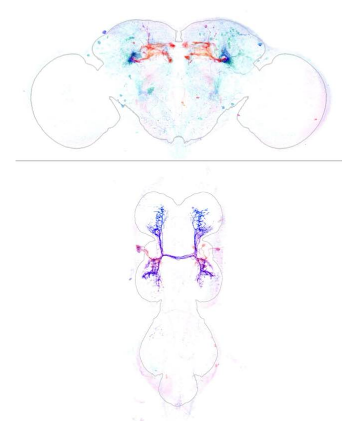
SS57948

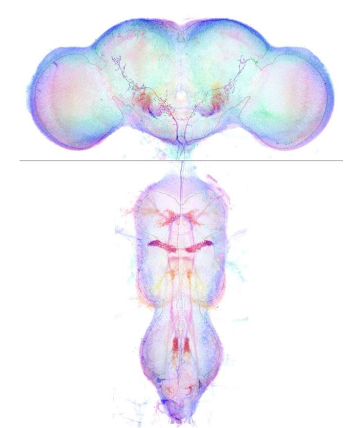
SS57949

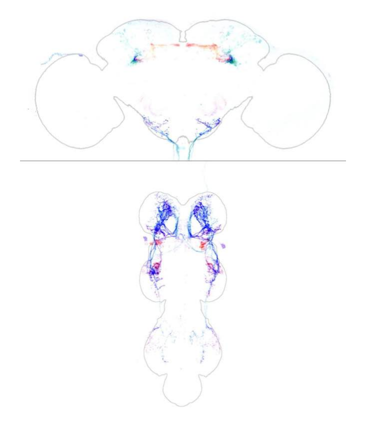
SS57954

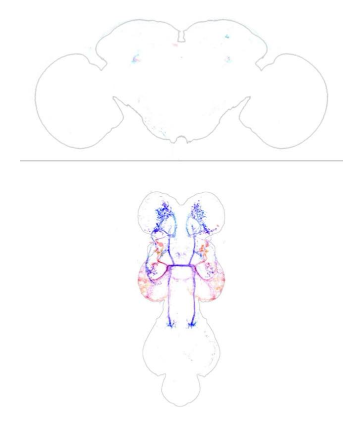
SS57955

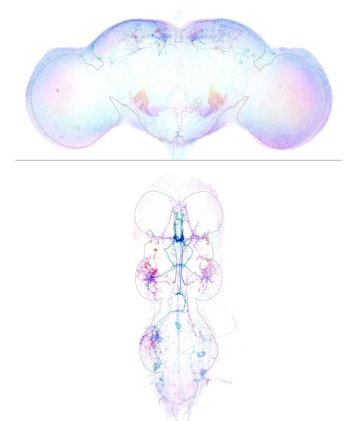
SS57956

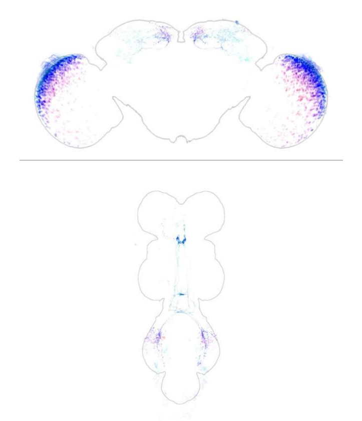
SS57966

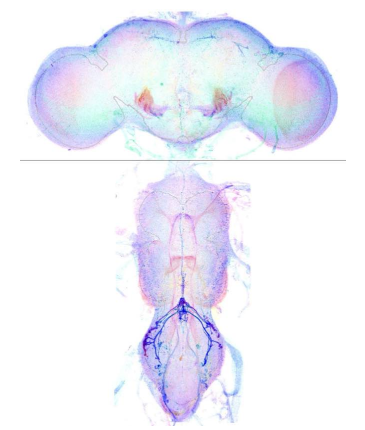
SS57975

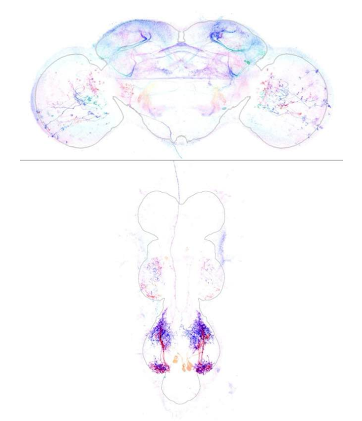
SS57977

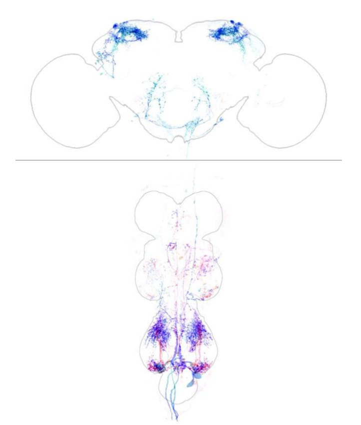
SS58000

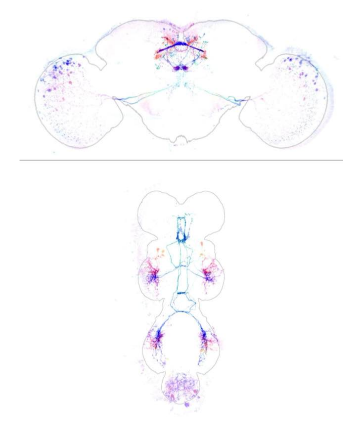
SS58001

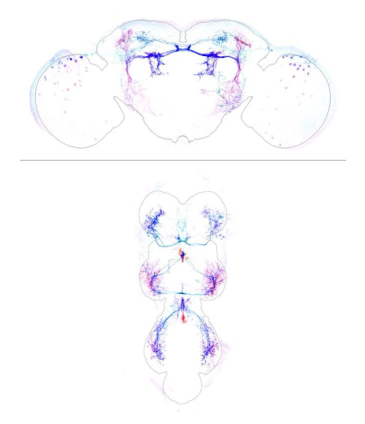
SS58002

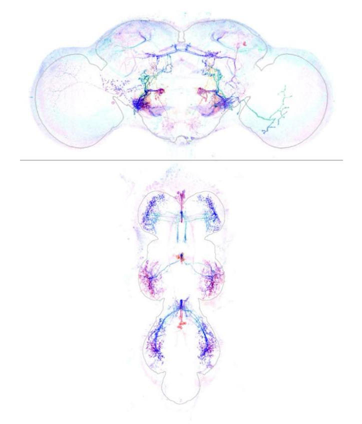
SS58005

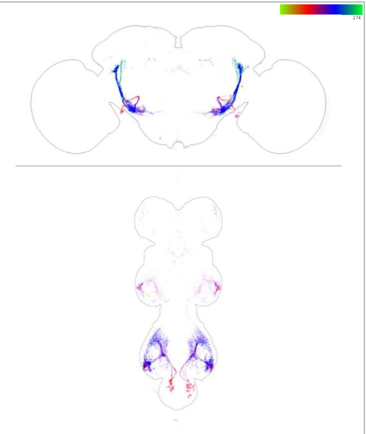
SS58006

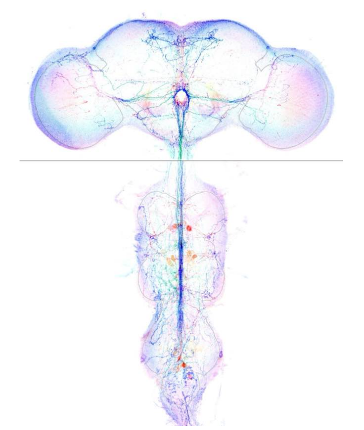
SS58011

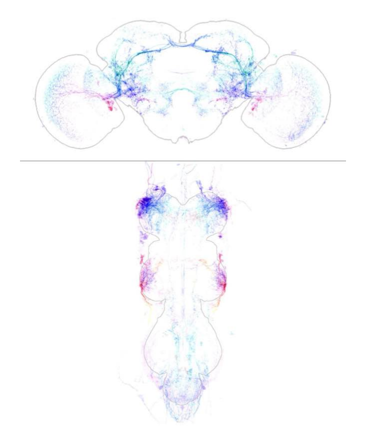
SS58142

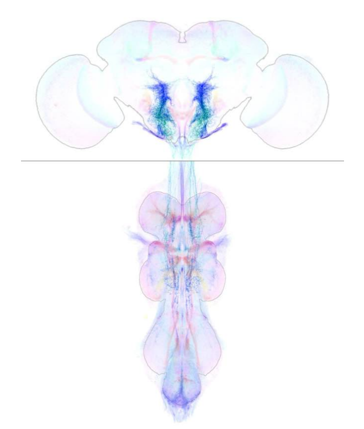
SS58304

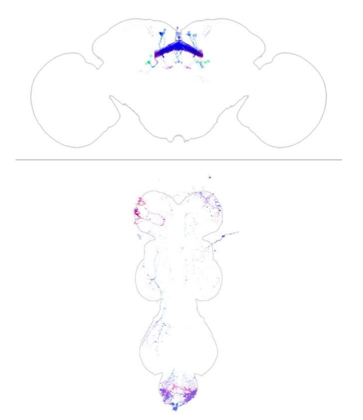
SS58449

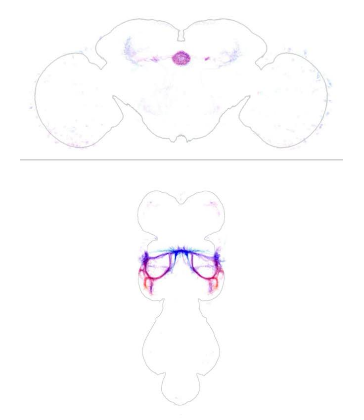
SS58605

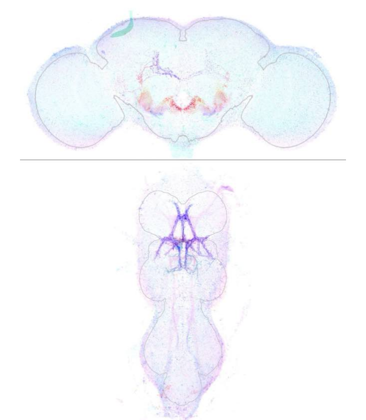
SS58626

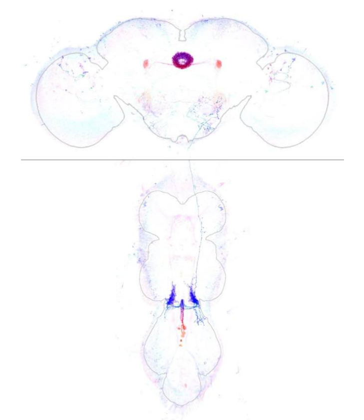
SS58784

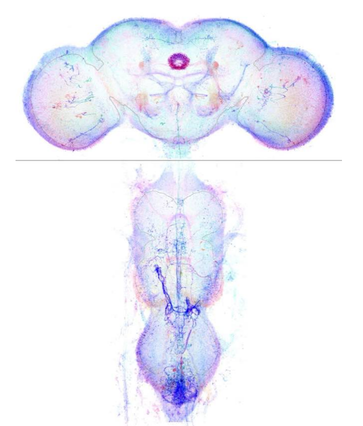
SS58788

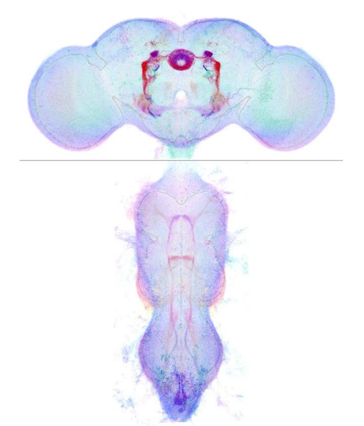
SS58826

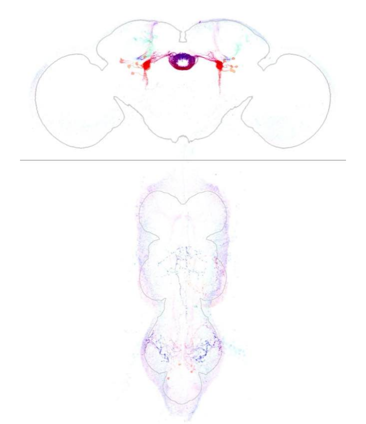
SS58833

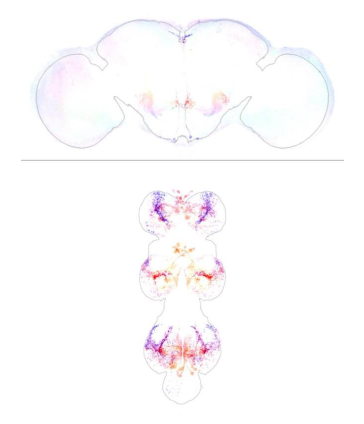
SS58839

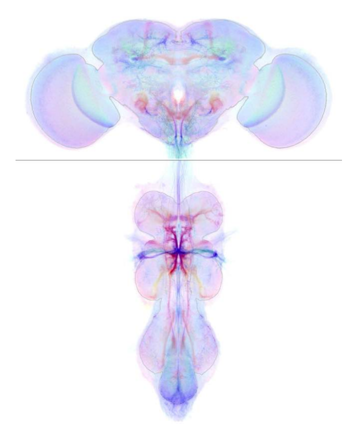
SS58881

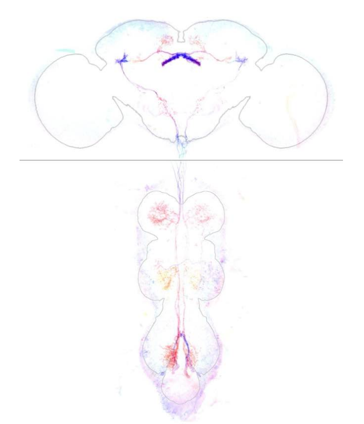
SS59090

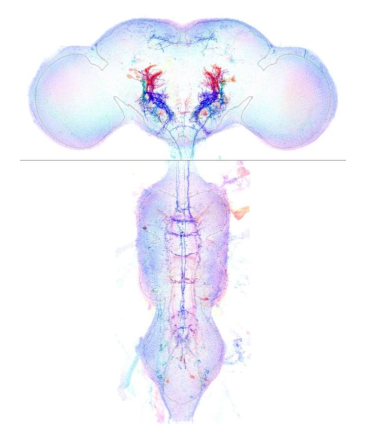
SS59149

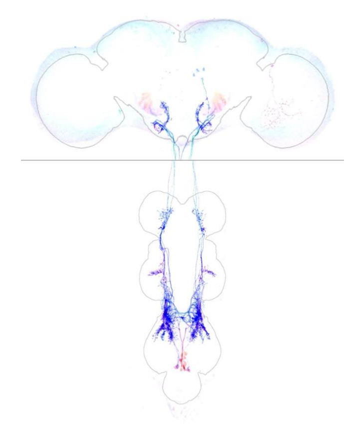
SS59163

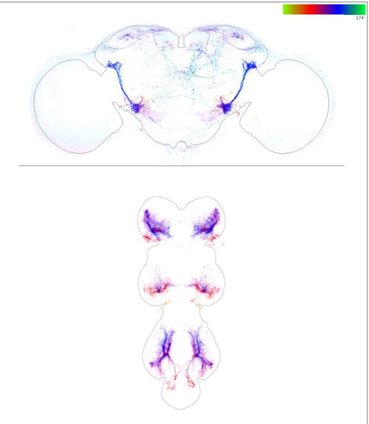
SS59173

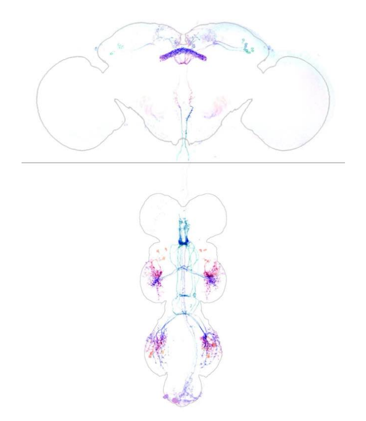
SS59205

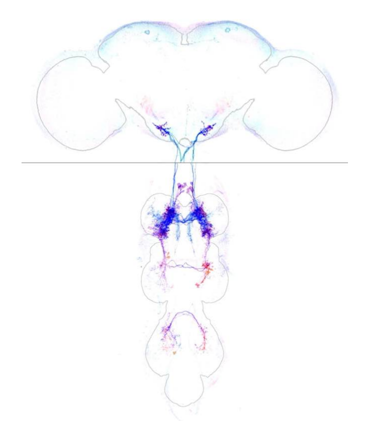
SS59209

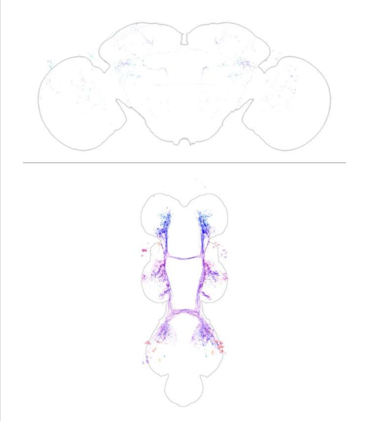
SS59225

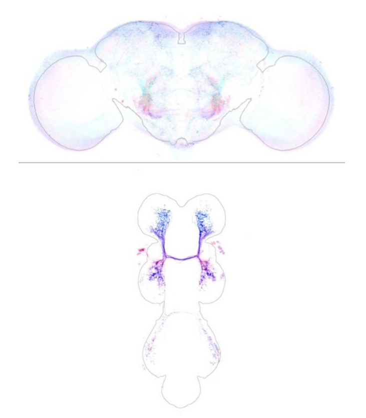
SS59226

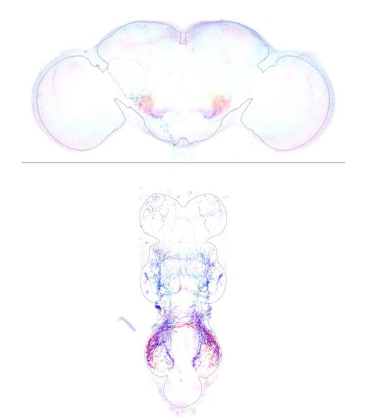
SS59229

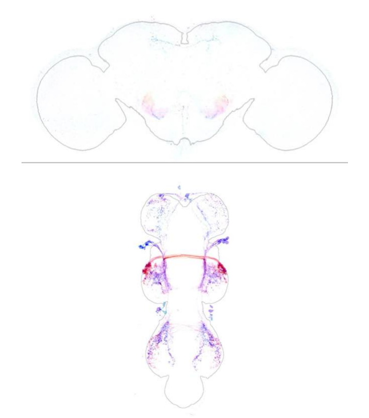
SS59230

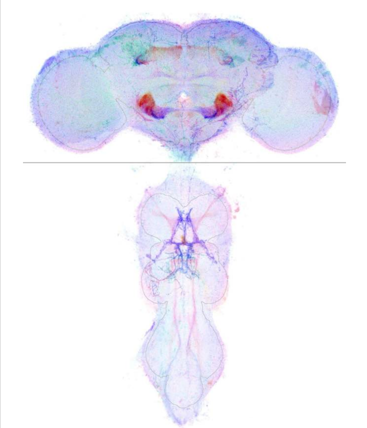
SS59290

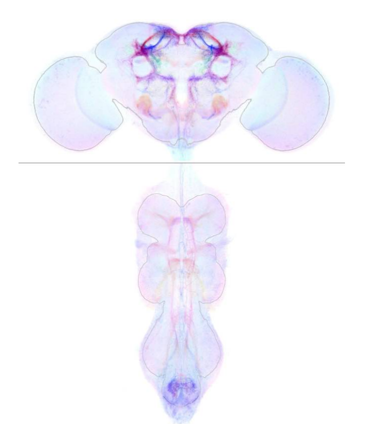
SS59331

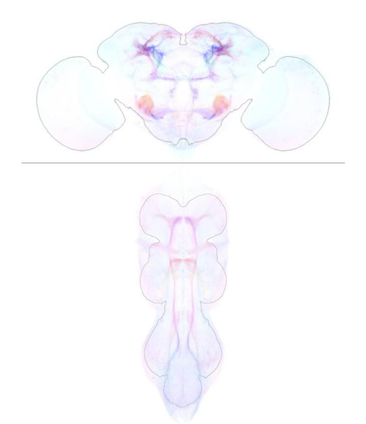
SS59336

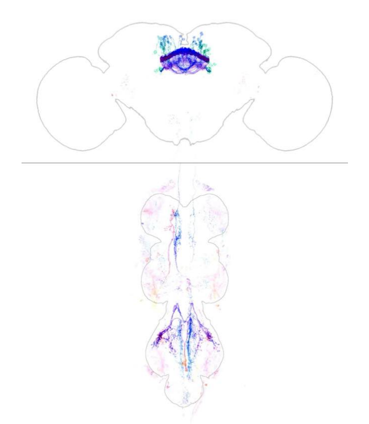
SS59408

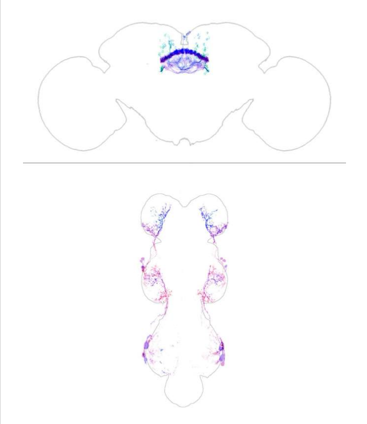
SS59410

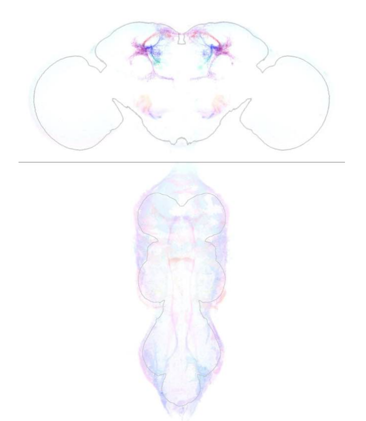
SS59433

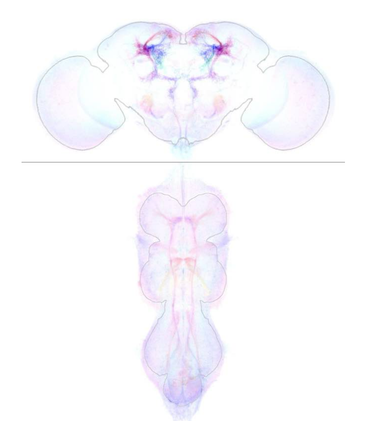
SS59453

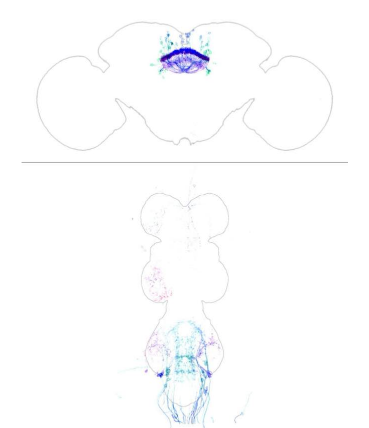
SS59455

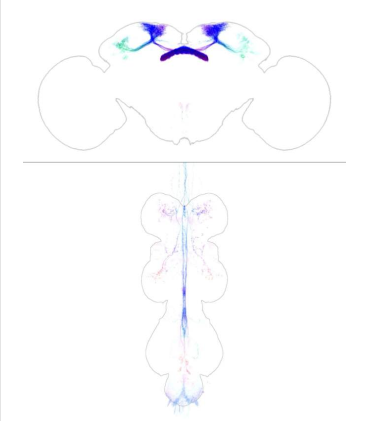
SS59468

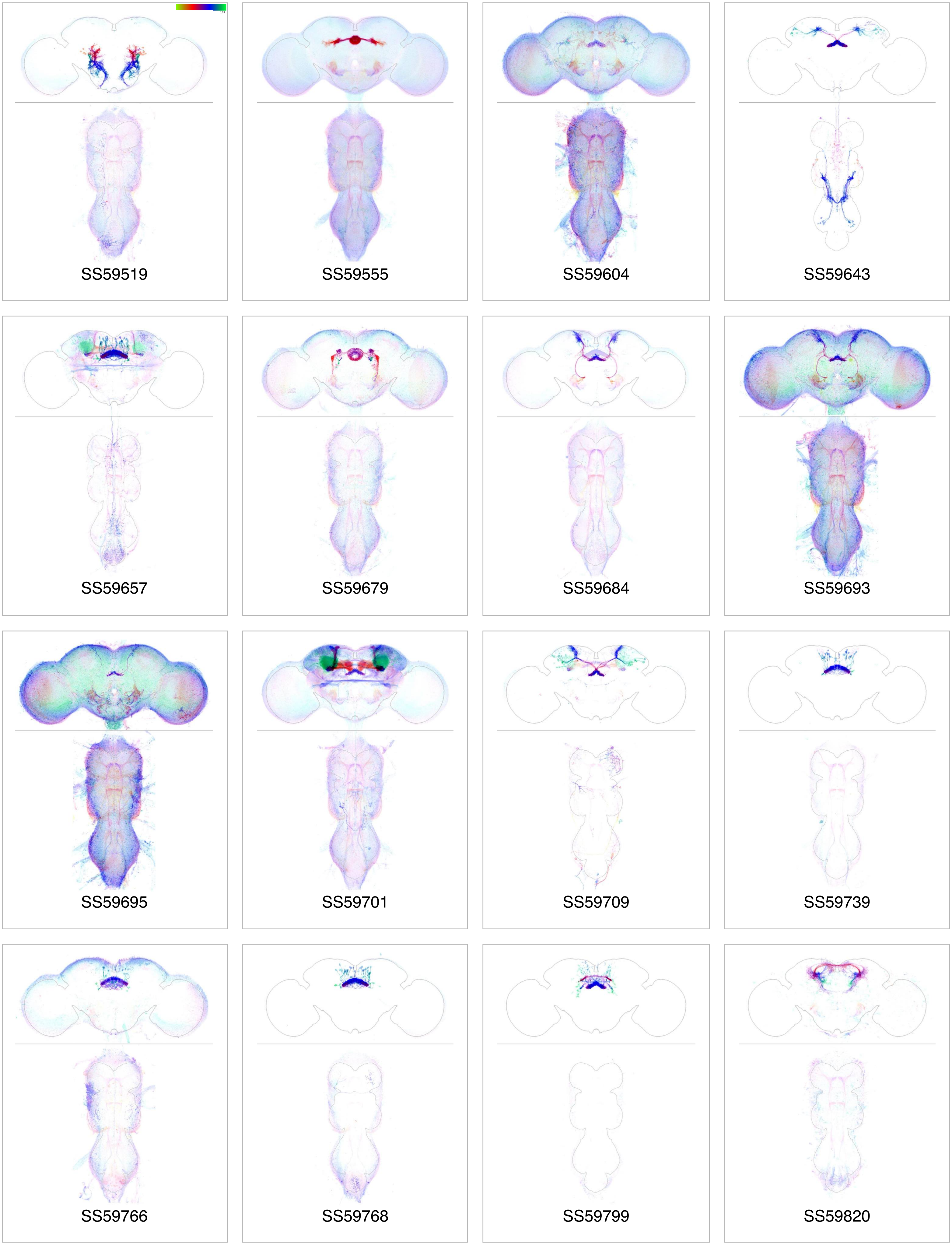
SS59519
SS59555
SS59604
SS59643
SS59657
SS59679
SS59684
SS59693
SS59695
SS59701
SS59709
SS59739
SS59766
SS59768
SS59799
SS59820

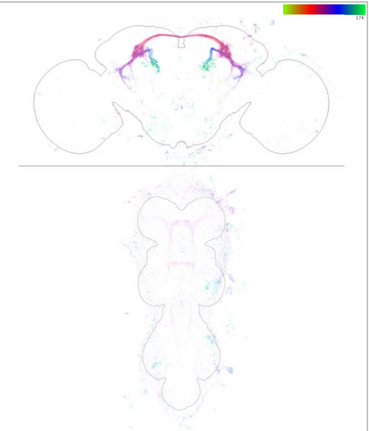
SS59821

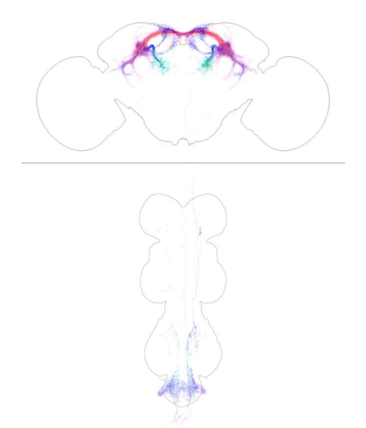
SS59823

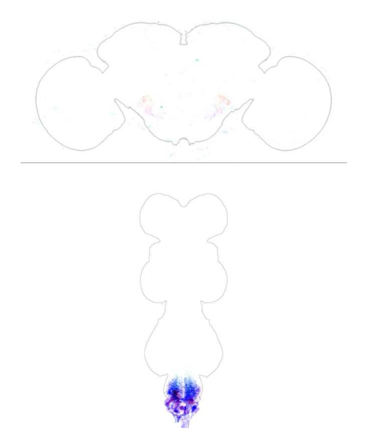
SS59824

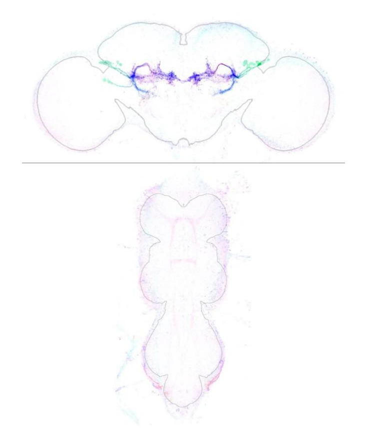
SS59825

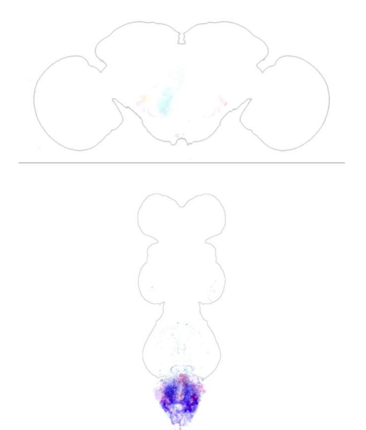
SS59827

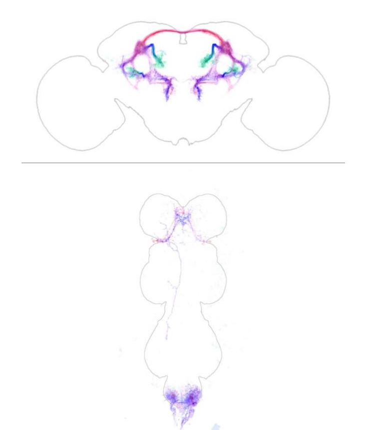
SS59829

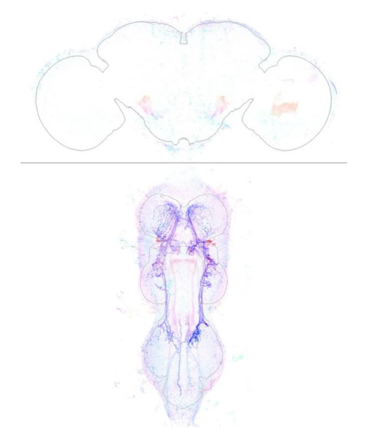
SS59830

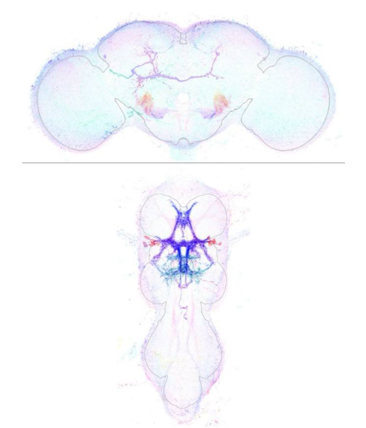
SS59832

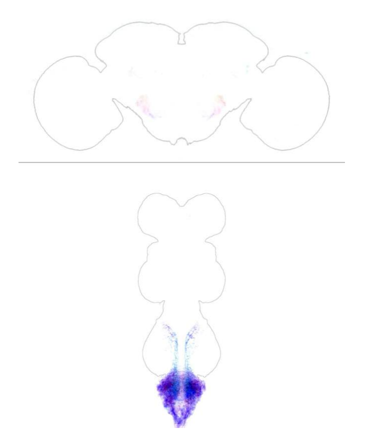
SS59833

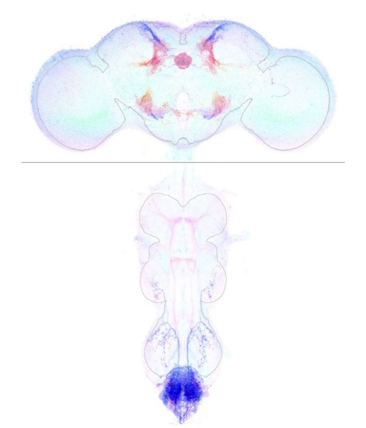
SS59835

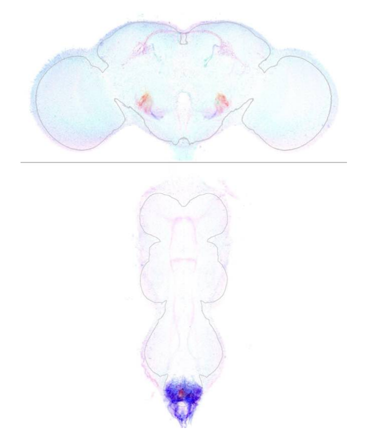
SS59836

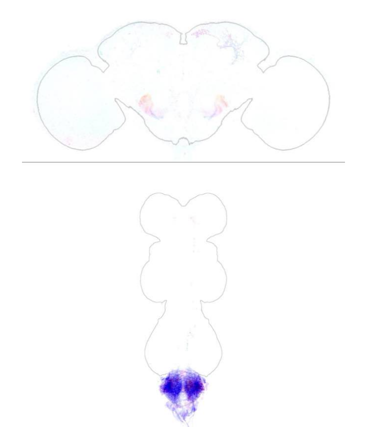
SS59841

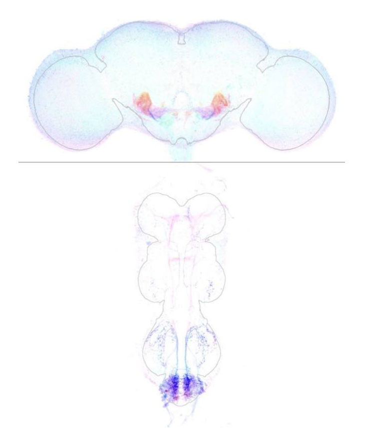
SS59842

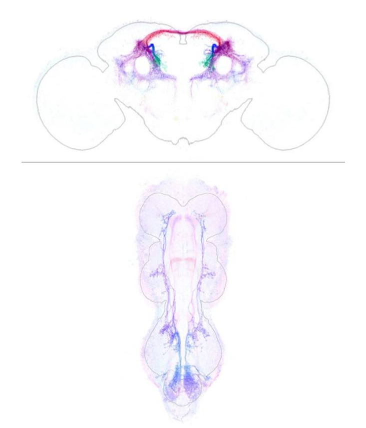
SS59843

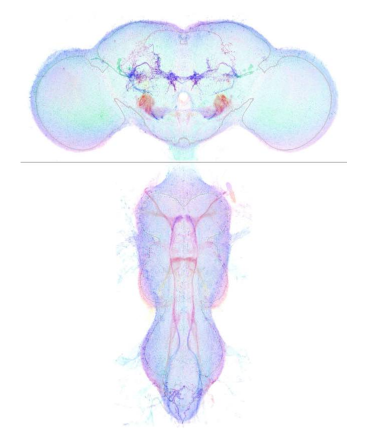
SS59844

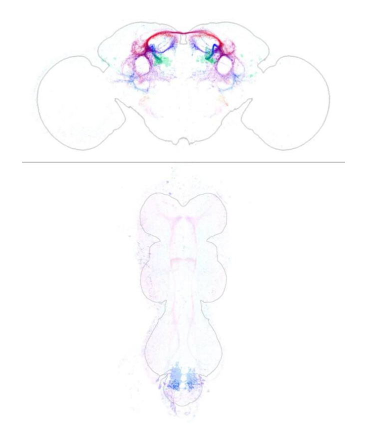
SS59845

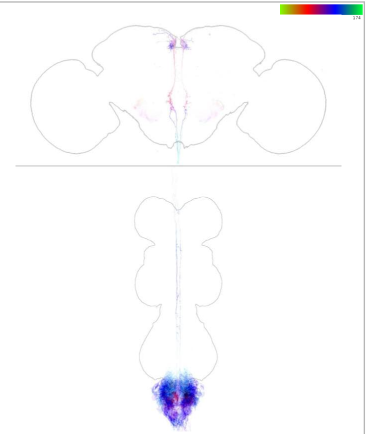
SS59847

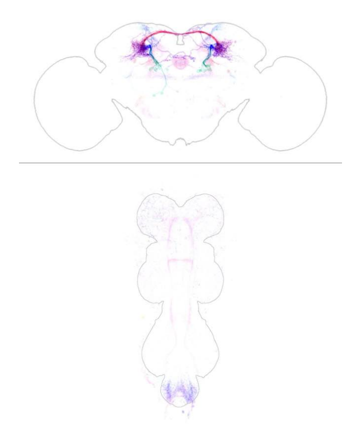
SS59850

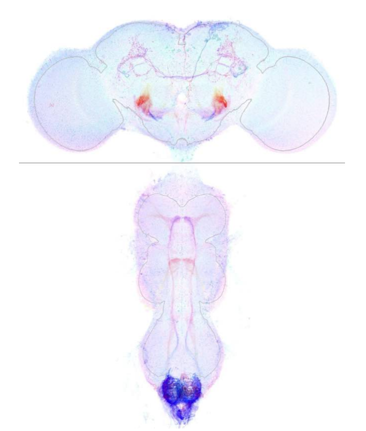
SS59852

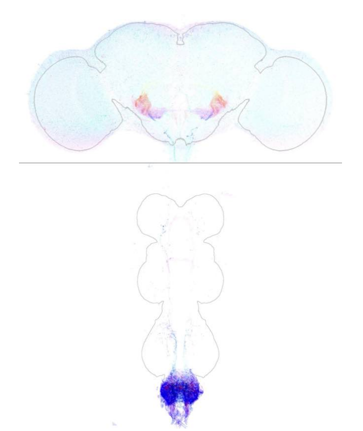
SS59862

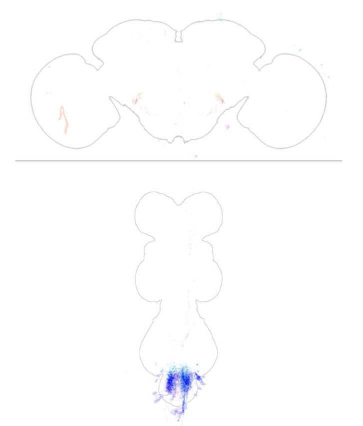
SS59869

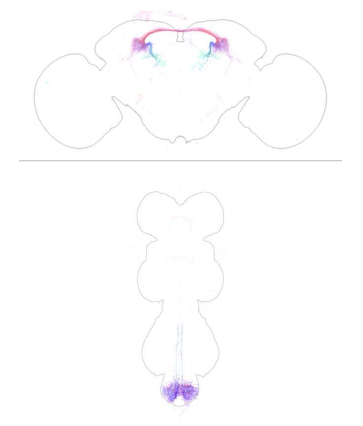
SS59871

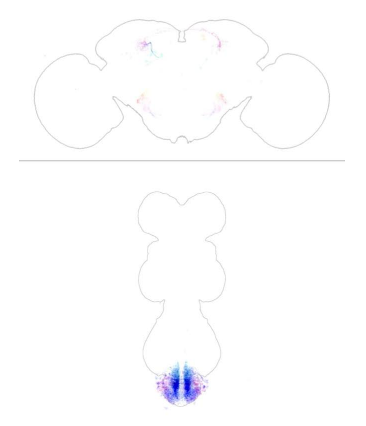
SS59872

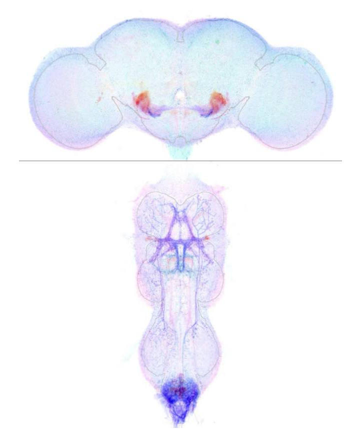
SS59874

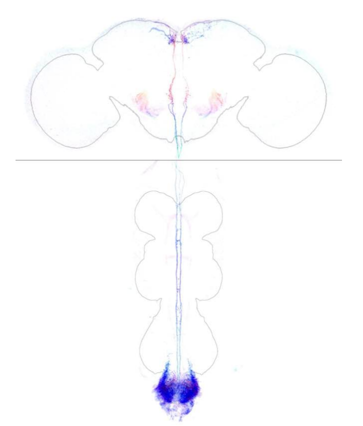
SS59876

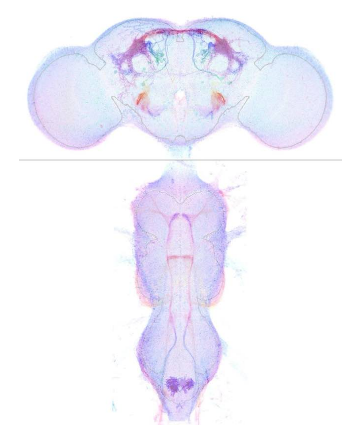
SS59879

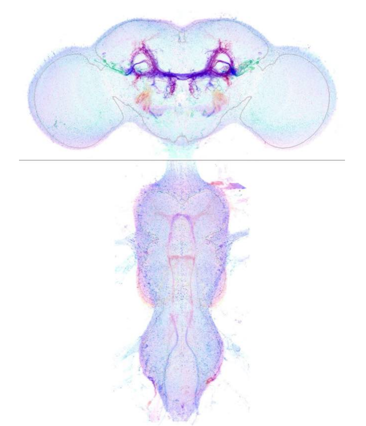
SS59880

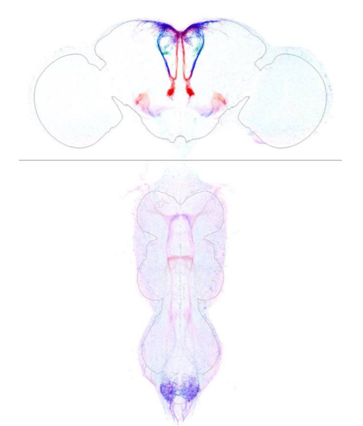
SS59881

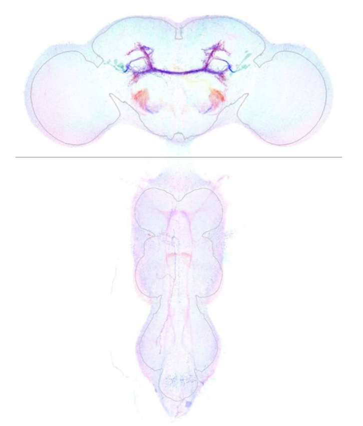
SS59882

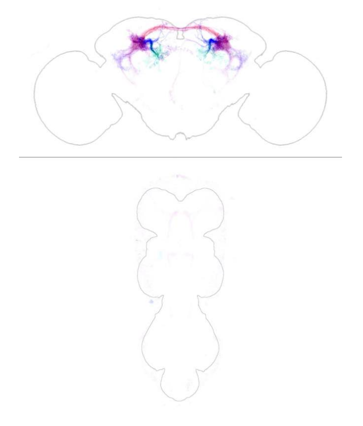
SS59887

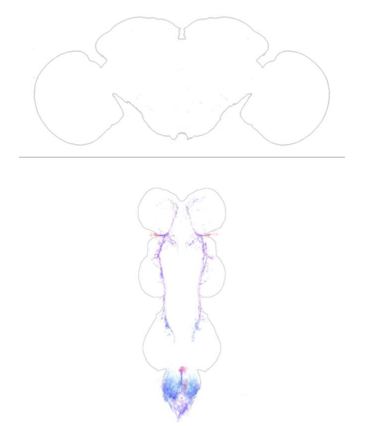
SS59889

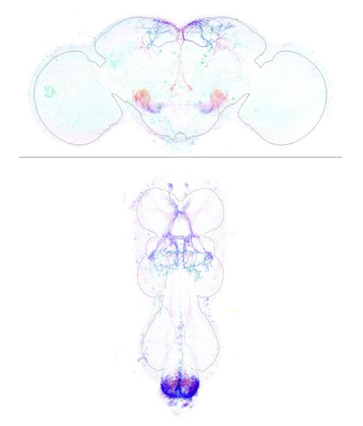
SS59903

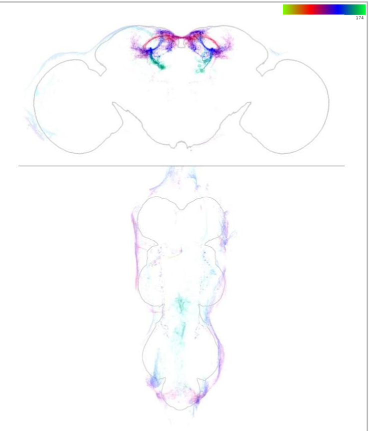
SS59911

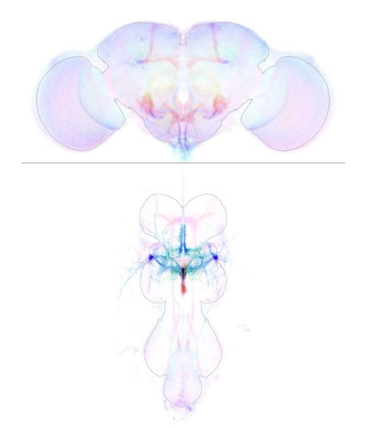
SS59970

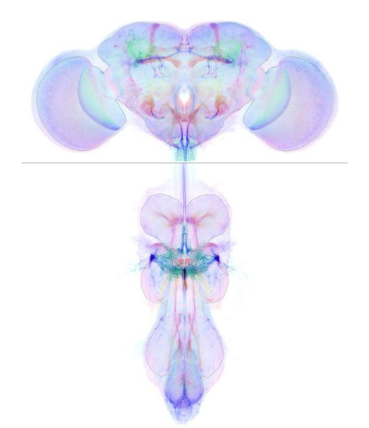
SS59972

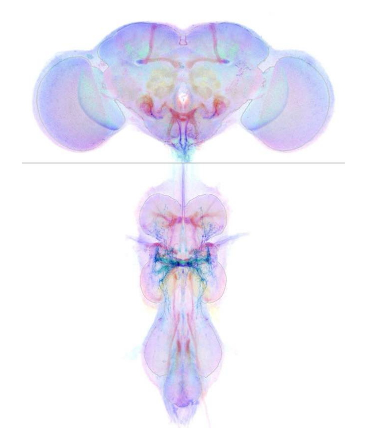
SS59978

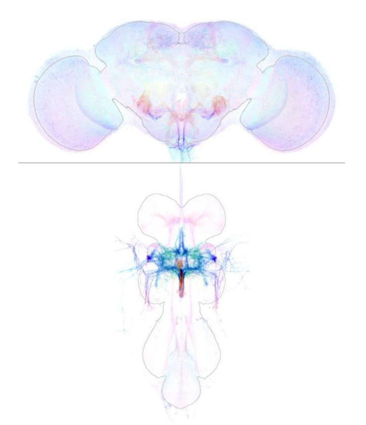
SS60041

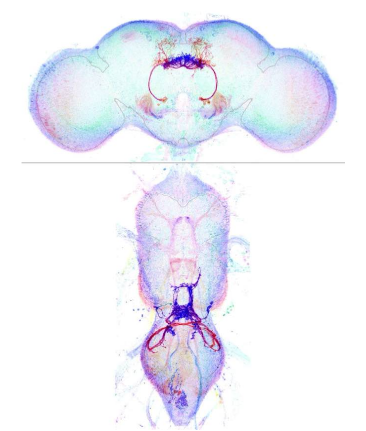
SS60051

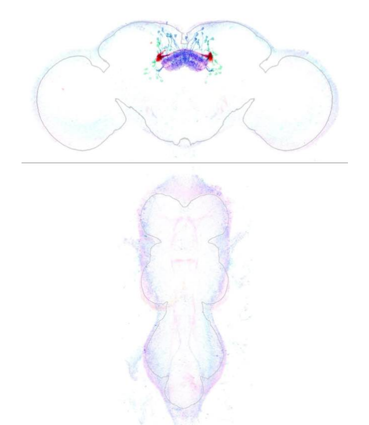
SS60081

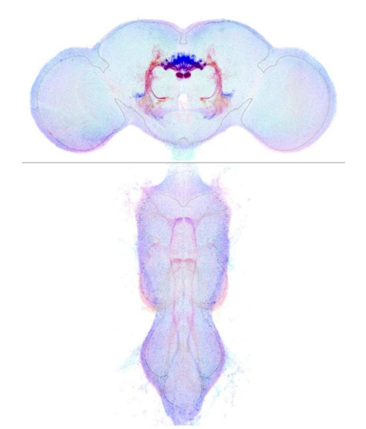
SS60112

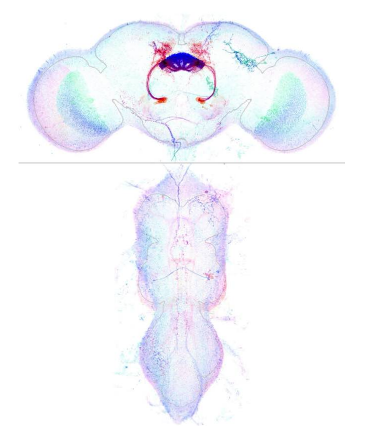
SS60118

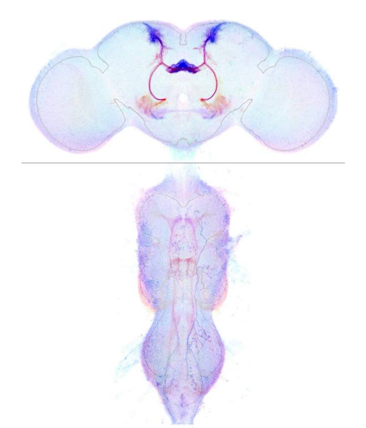
SS60125

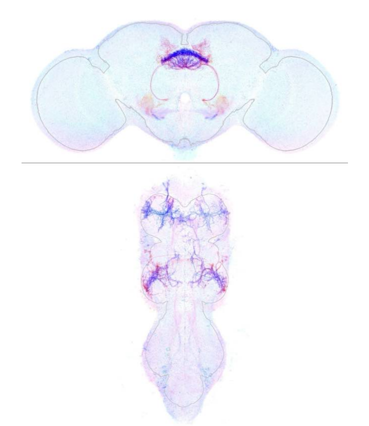
SS60140

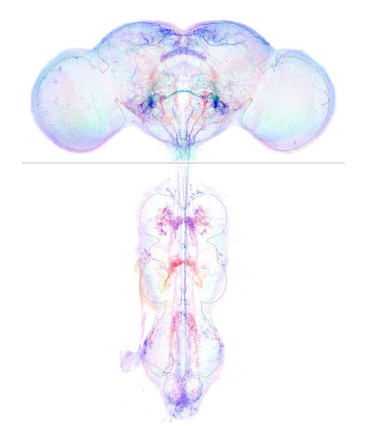
SS60184

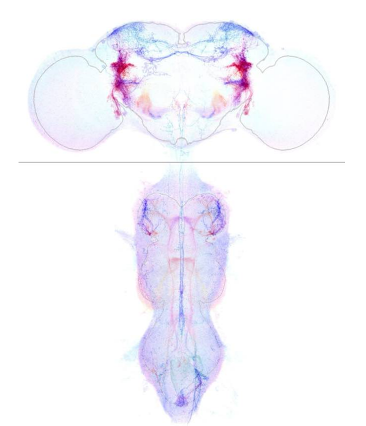
SS60195

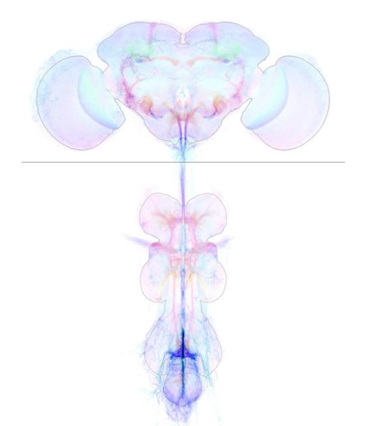
SS60199

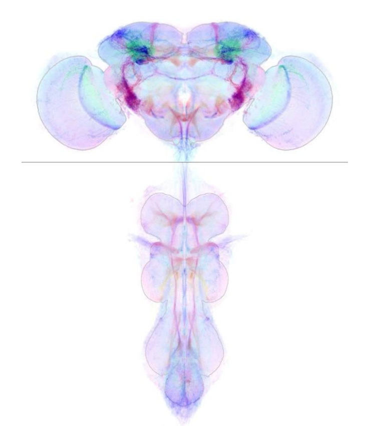
SS60200

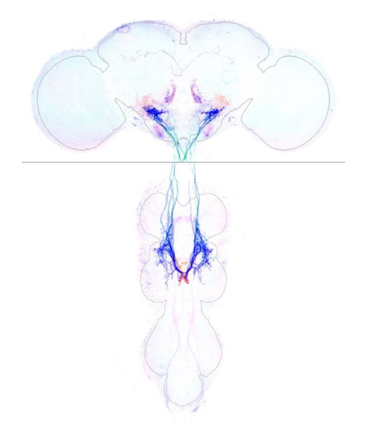
SS60203

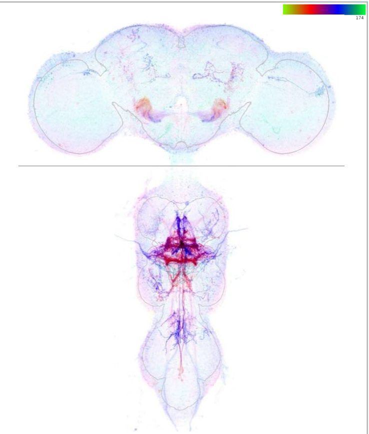
SS60209

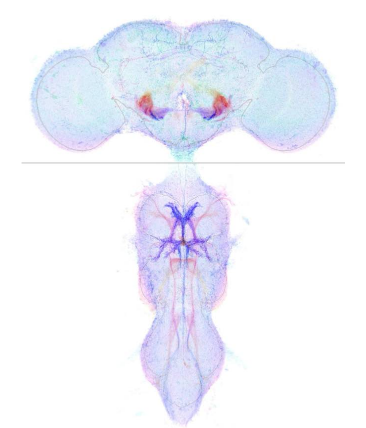
SS60214

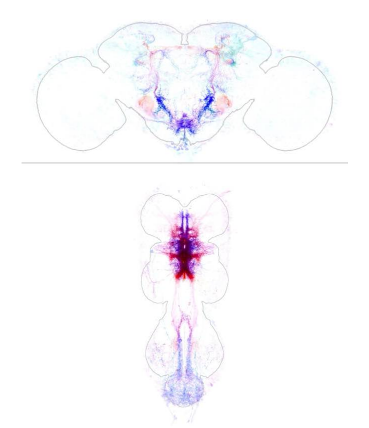
SS60215

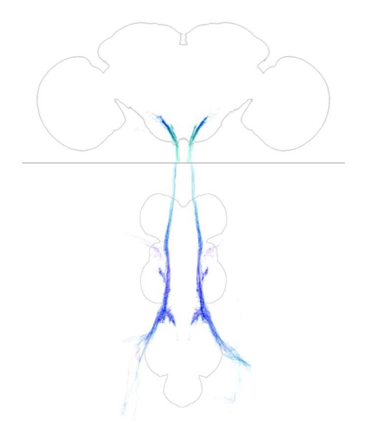
SS60216

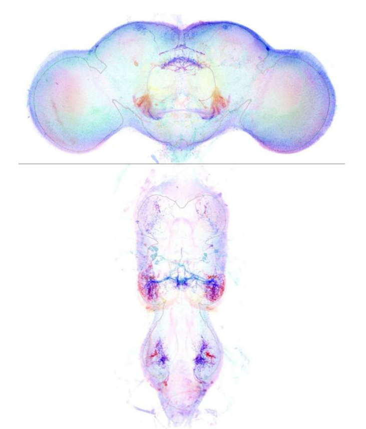
SS60227

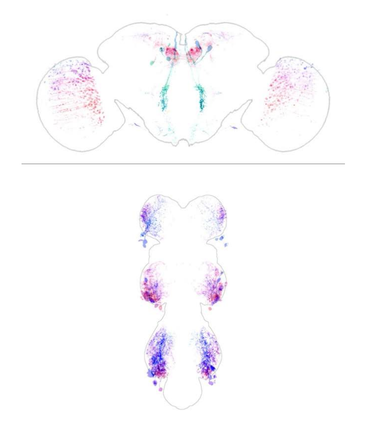
SS60231

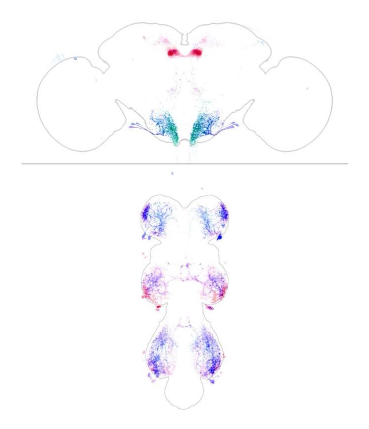
SS60232

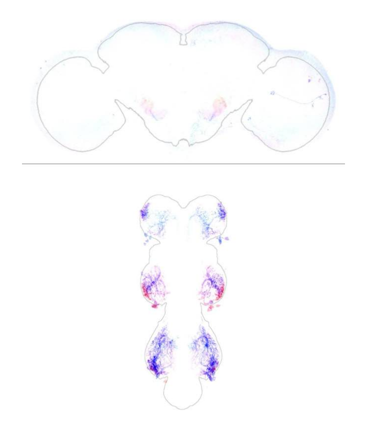
SS60234

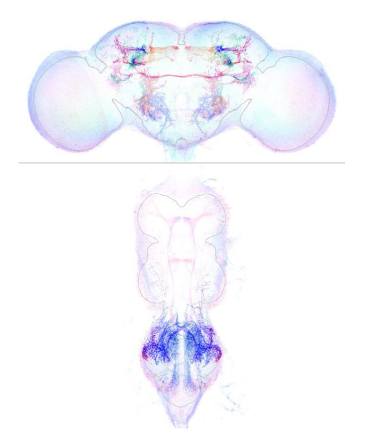
SS60237

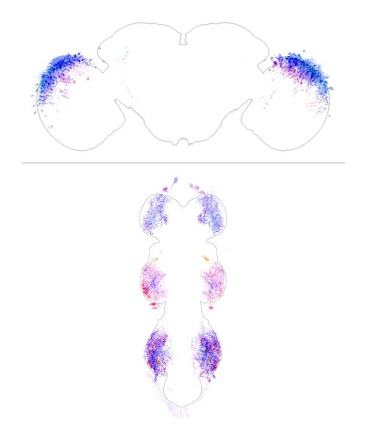
SS60240

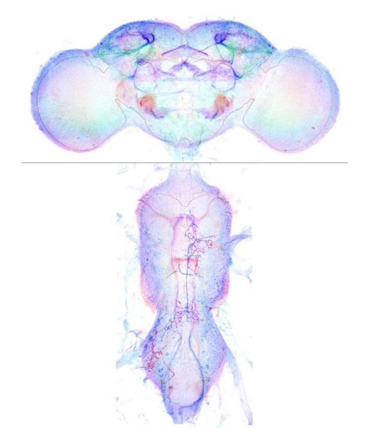
SS60258

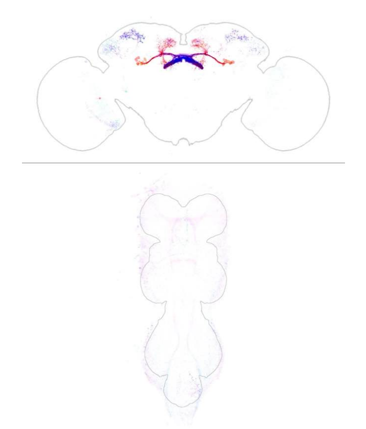
SS60305

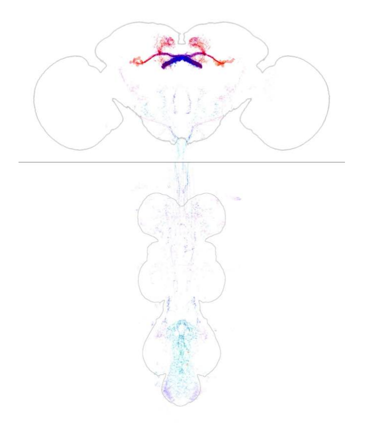
SS60306

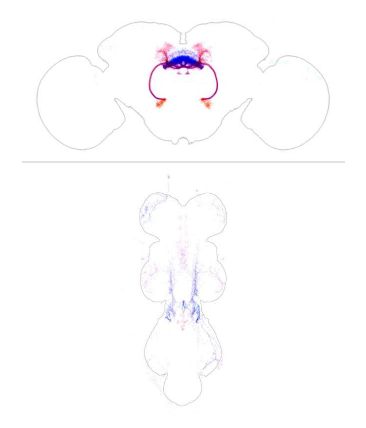
SS60355

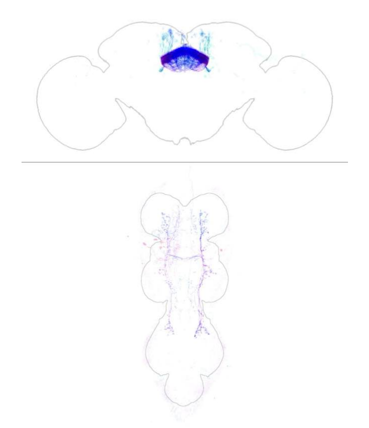
SS60363

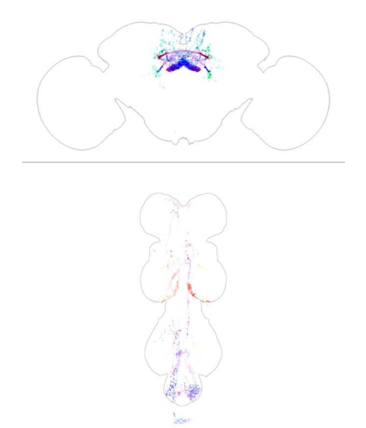
SS60366

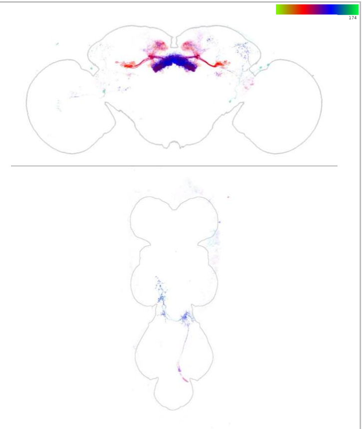
SS60378

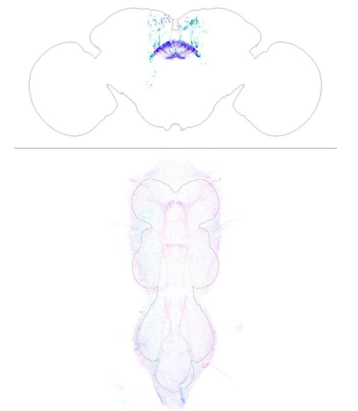
SS60427

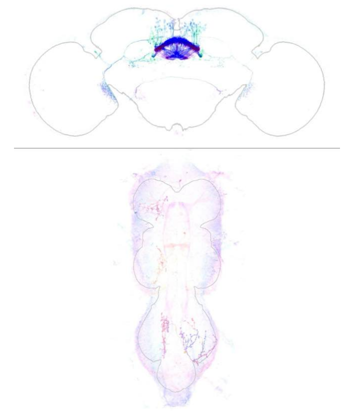
SS60428

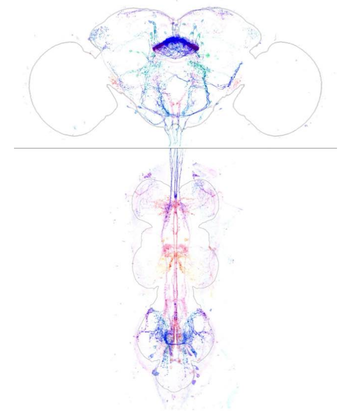
SS60474

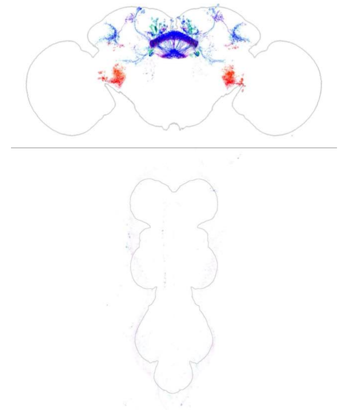
SS60482

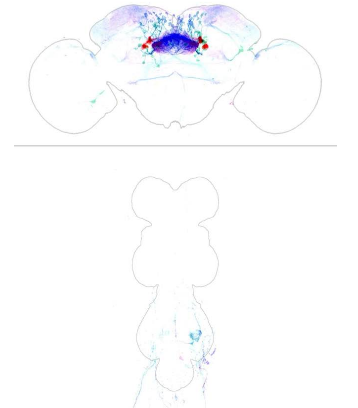
SS60483

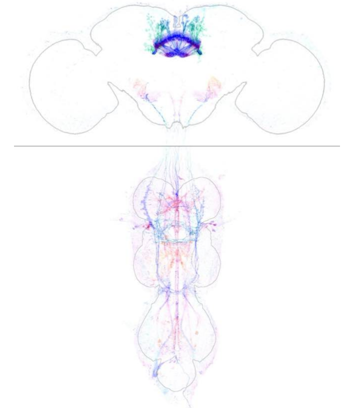
SS60493

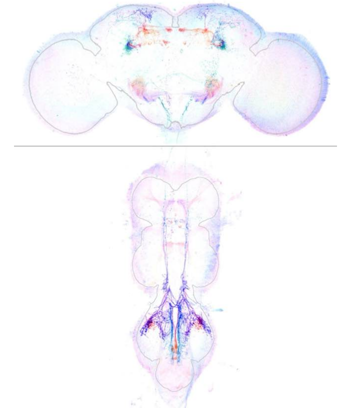
SS60585

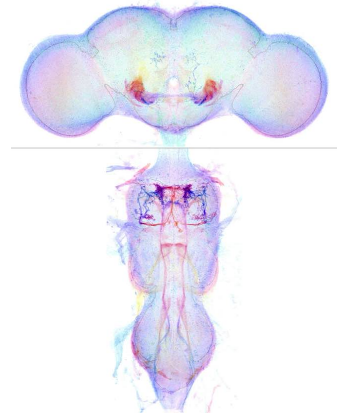
SS60590

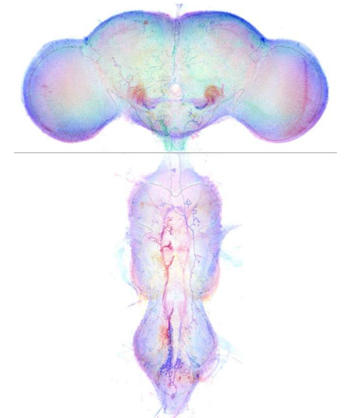
SS60600

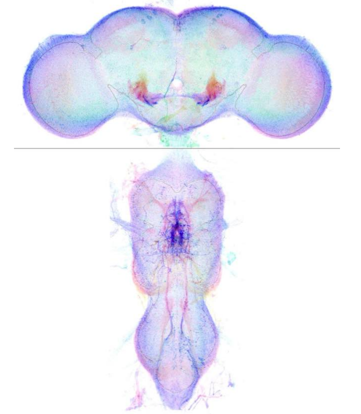
SS60603

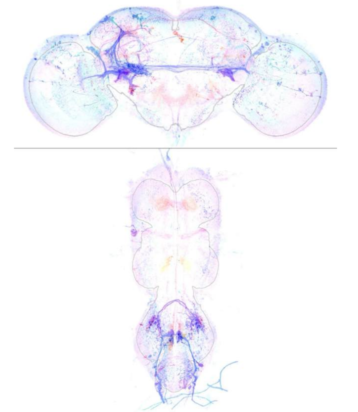
SS60605

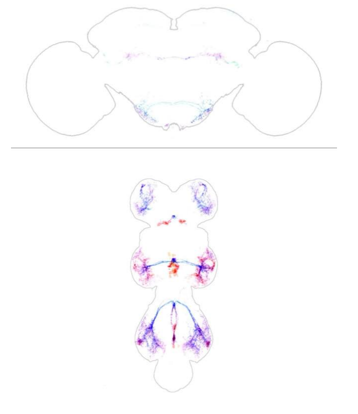
SS60607

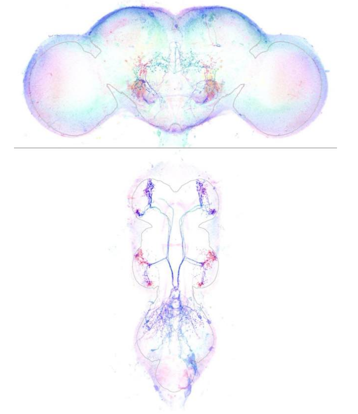
SS60618

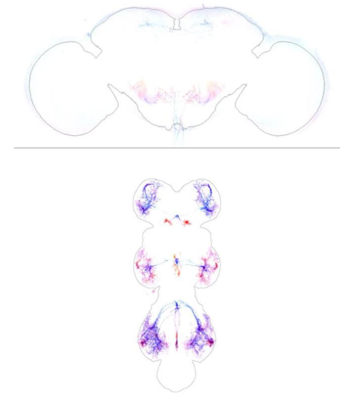
SS60624

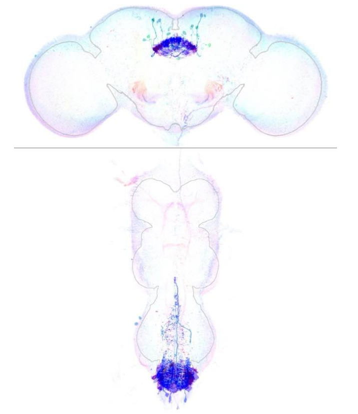
SS60702

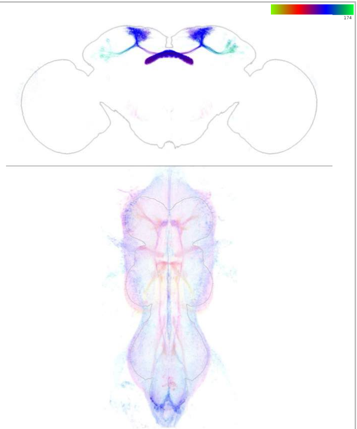
SS60711

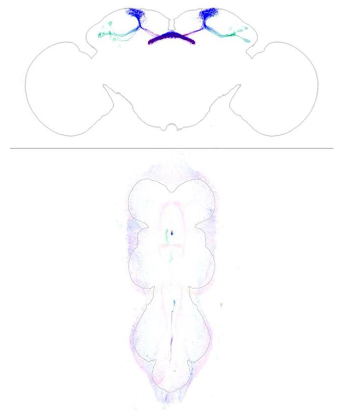
SS60754

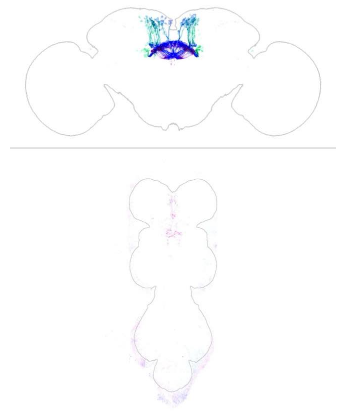
SS60755

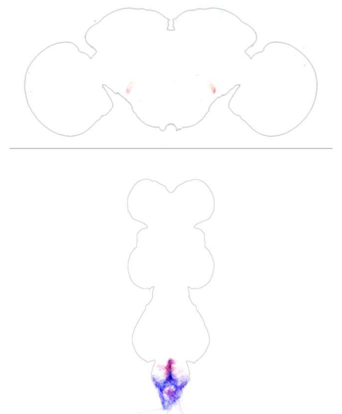
SS60797

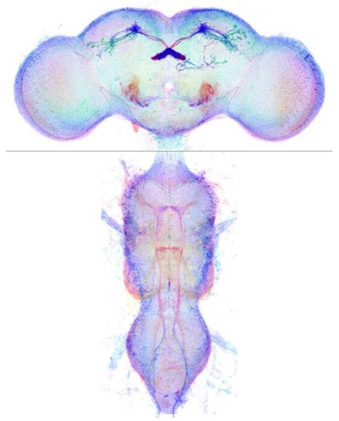
SS60877

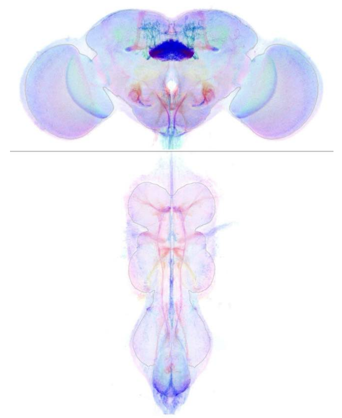
SS60919

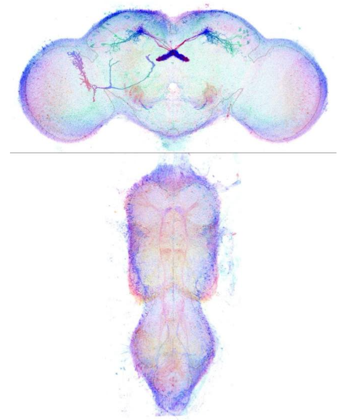
SS60954

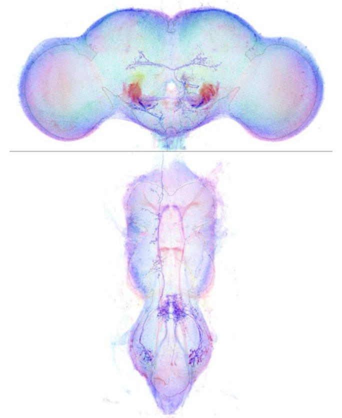
SS61022

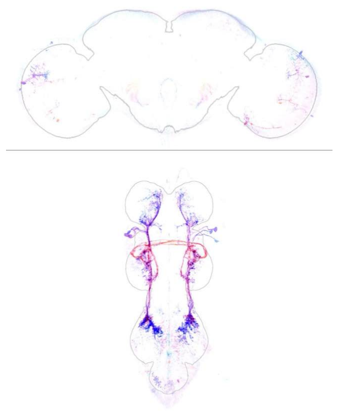
SS61036

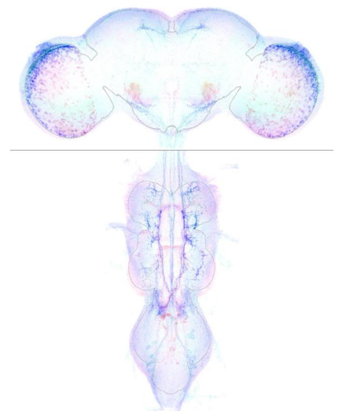
SS61045

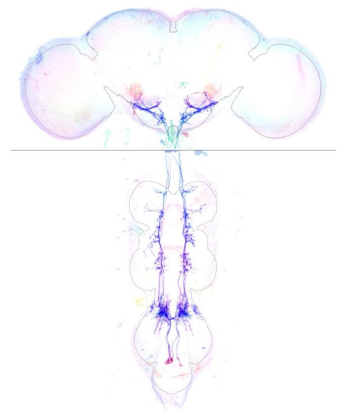
SS61051

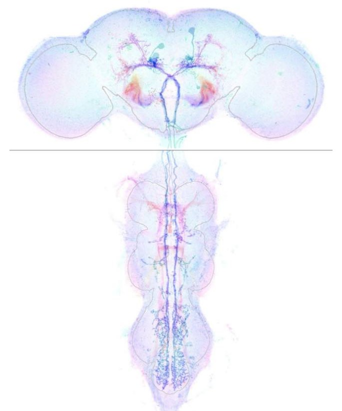
SS61089

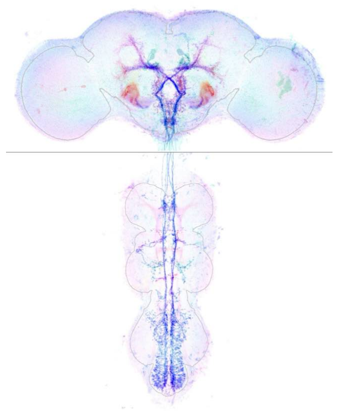
SS61090

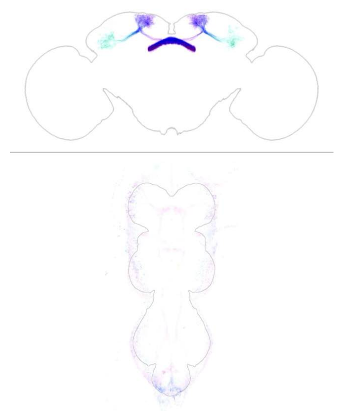
SS61099

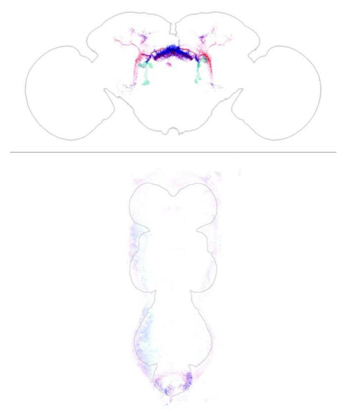
SS61133

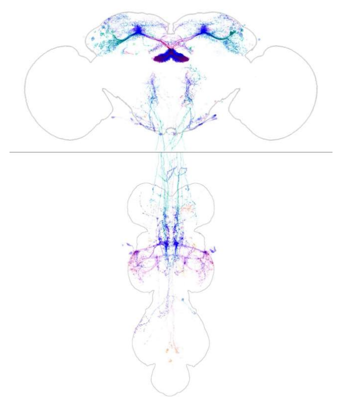
SS61140

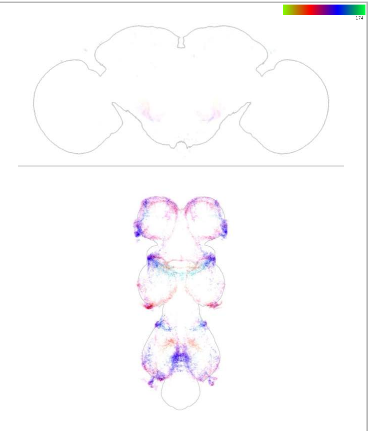
SS61325

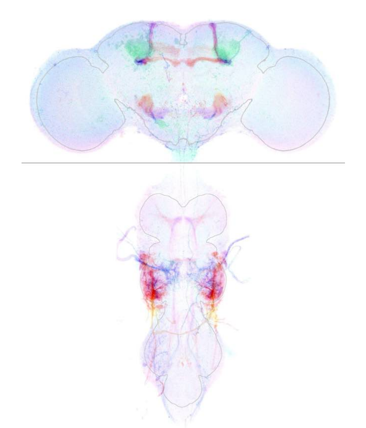
SS61345

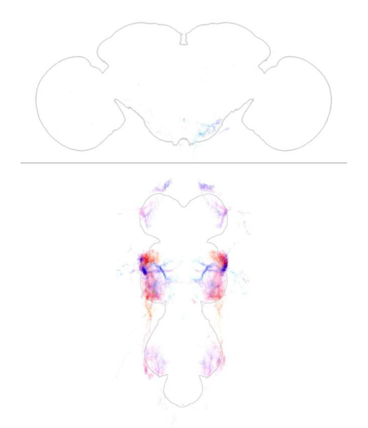
SS61350

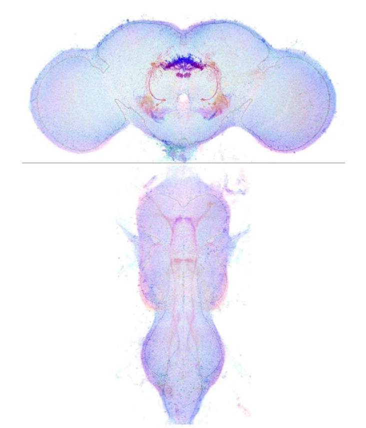
SS61391

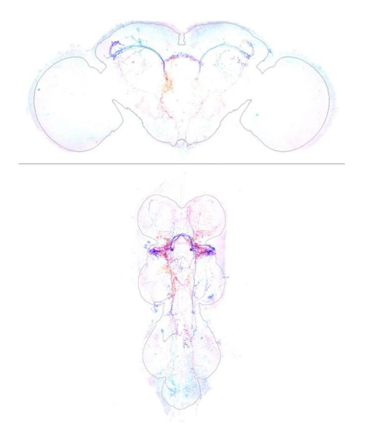
SS61439

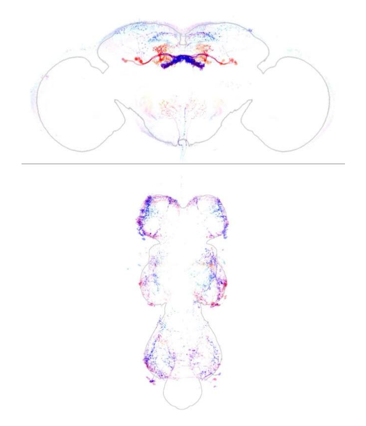
SS61448

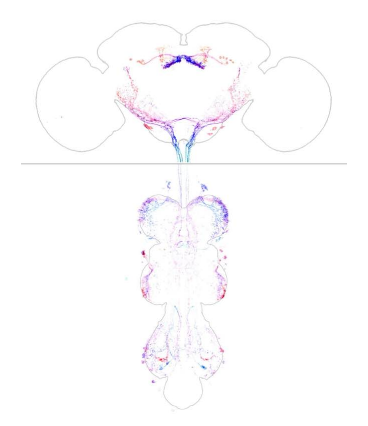
SS61450

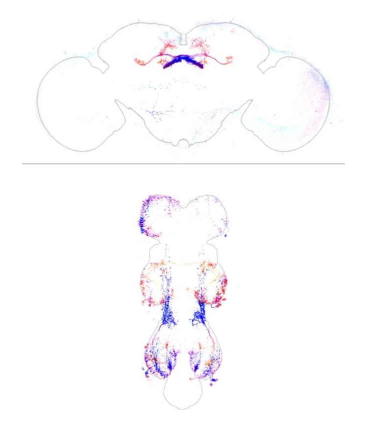
SS61475

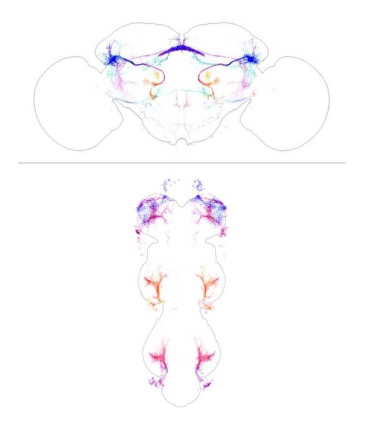
SS61503

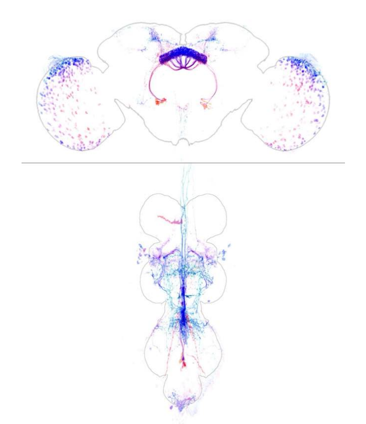
SS61514

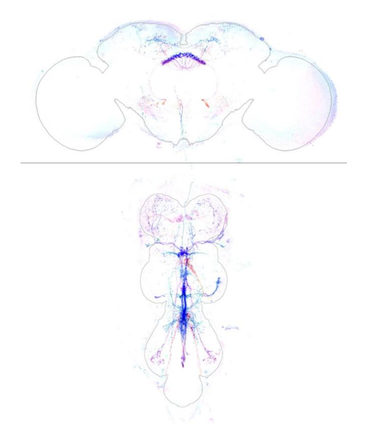
SS61528

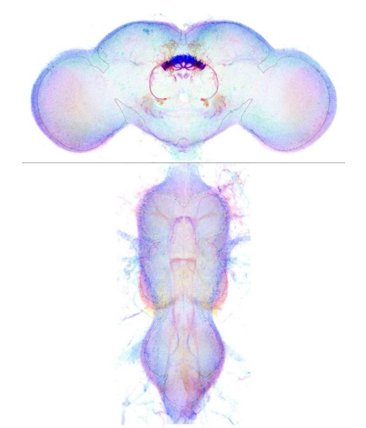
SS61532

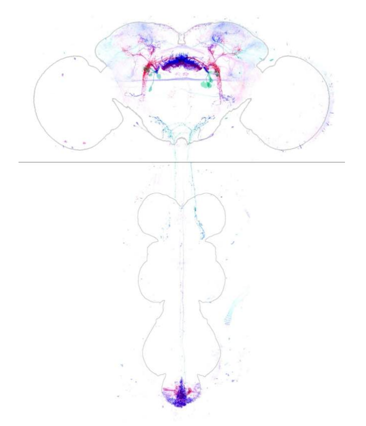
SS61593

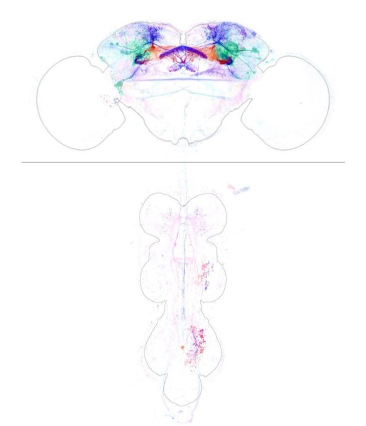
SS61676

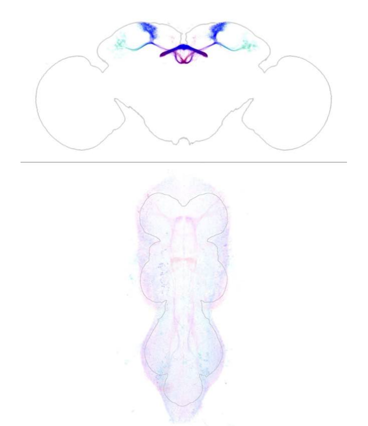
SS61677

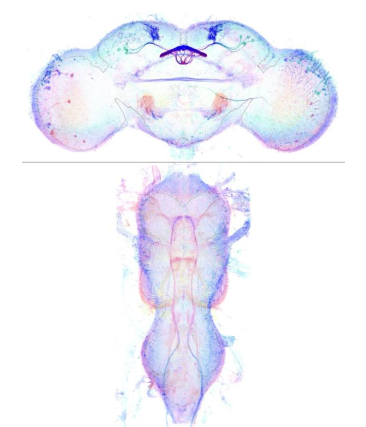
SS61682

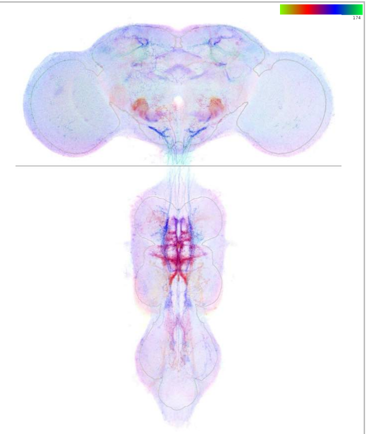
SS61739
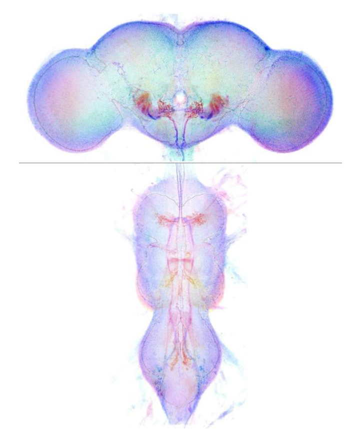
SS61786
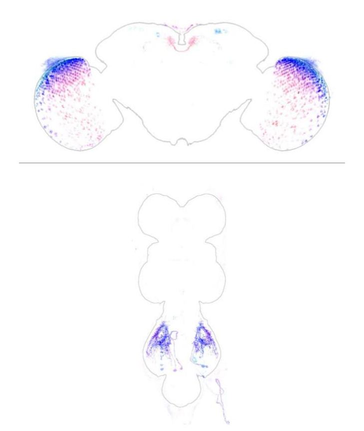
SS61790
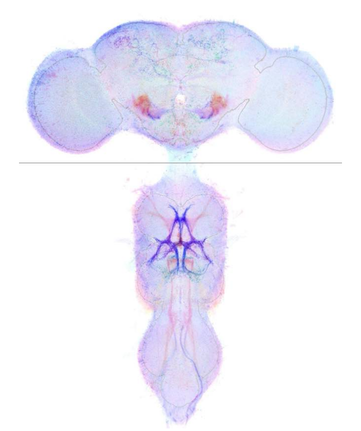
SS61806
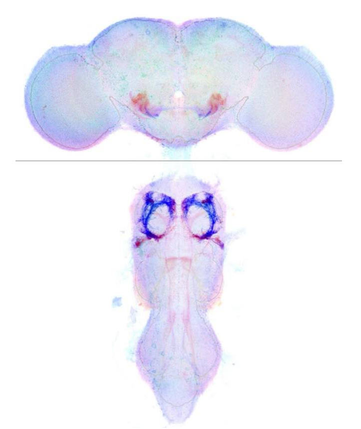
SS61810
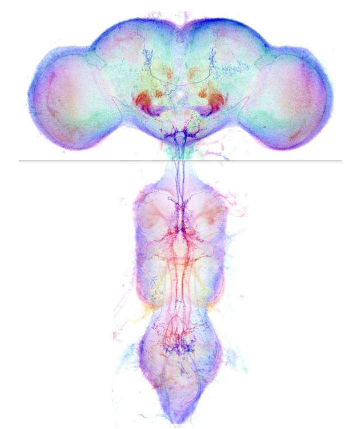
SS61813
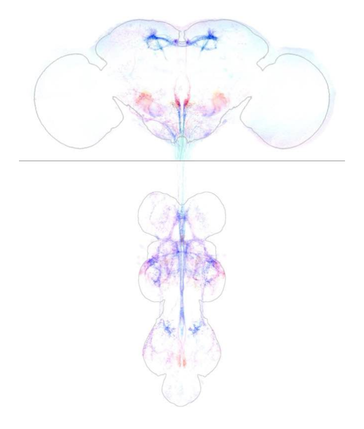
SS61820
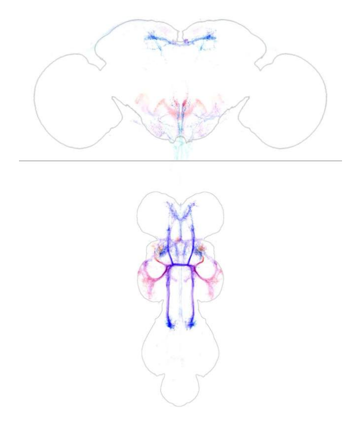
SS61821
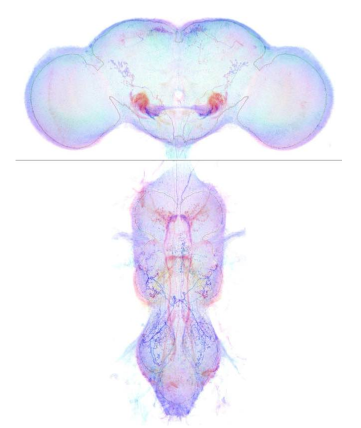
SS61829
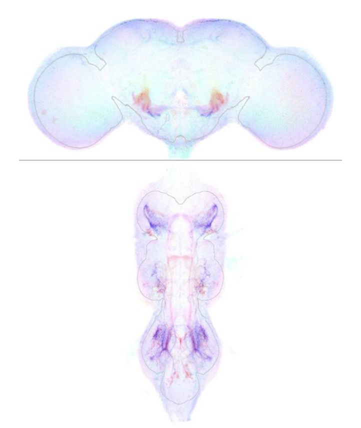
SS61836
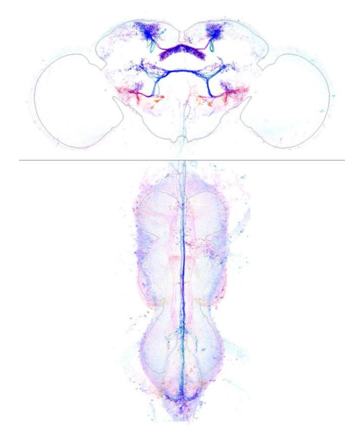
SS61862
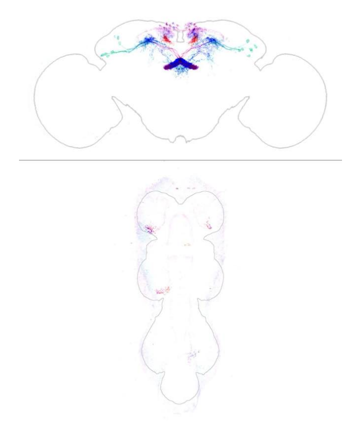
SS61885
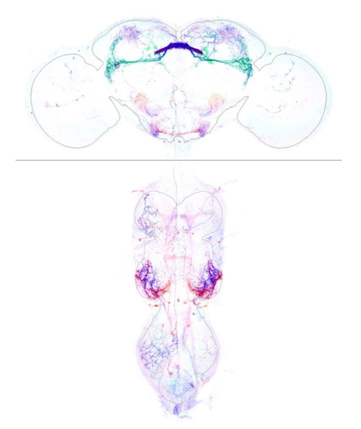
SS61934
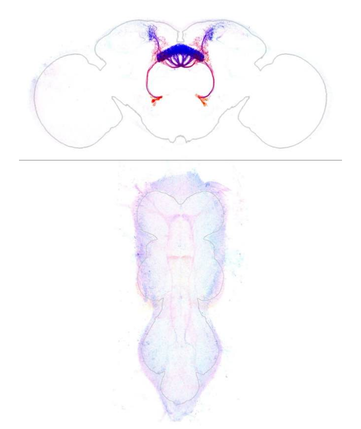
SS61953
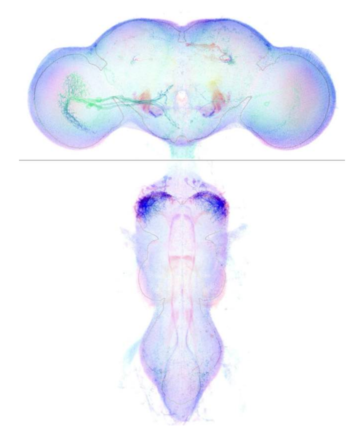
SS62120
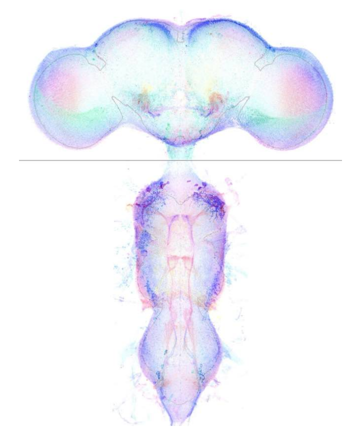
SS62137

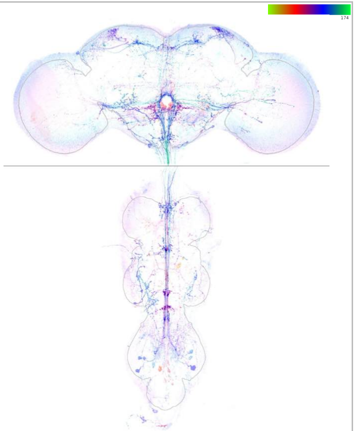

SS62148

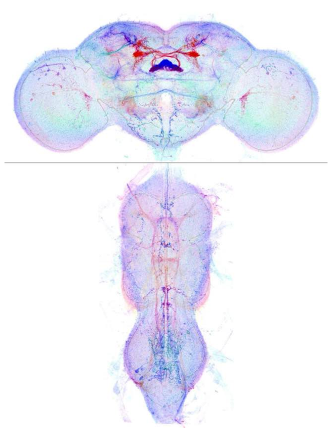

SS62182

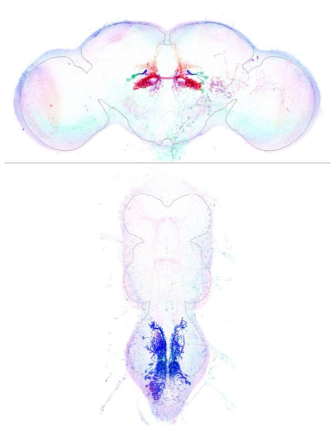

SS62404

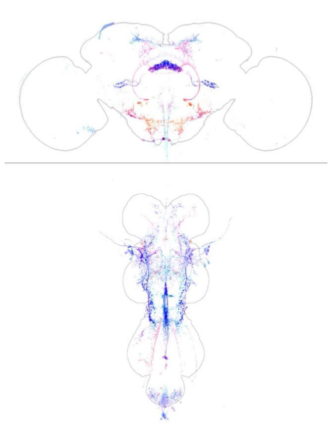

SS62473

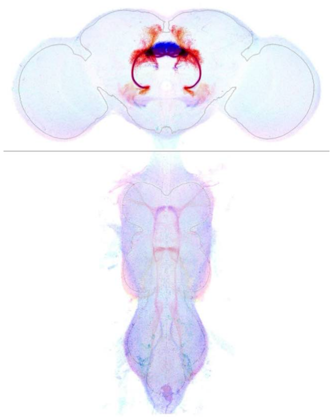

SS62506

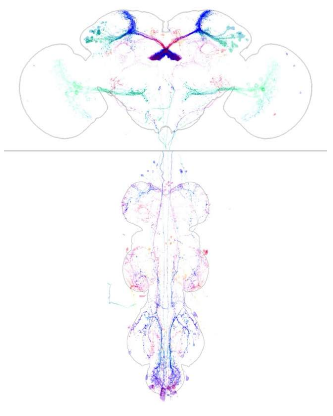

SS62558

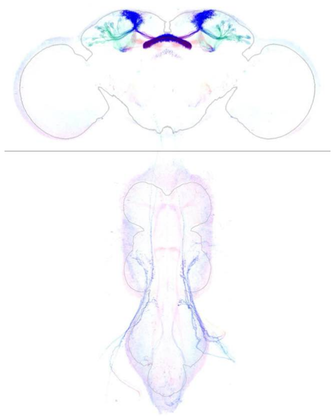

SS62566

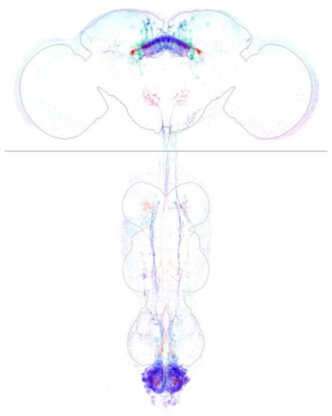

SS62596

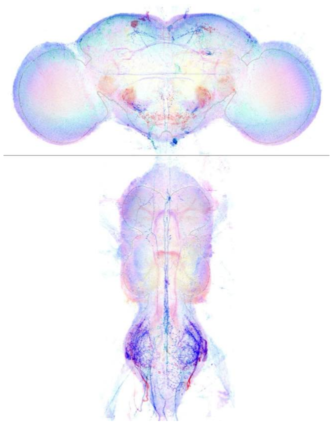

SS62678

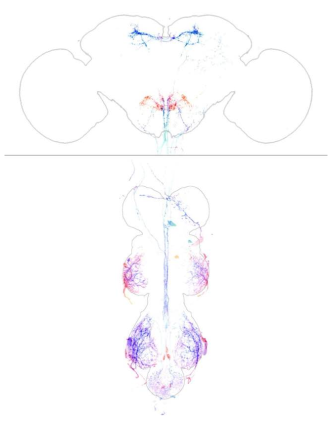

SS62687

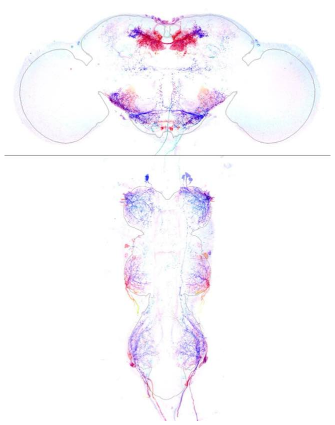

SS62706

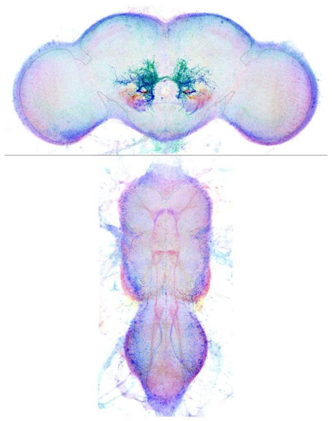

SS62736

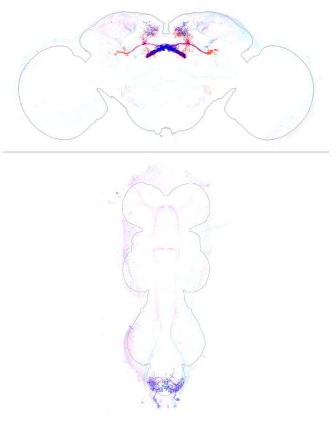

SS62768

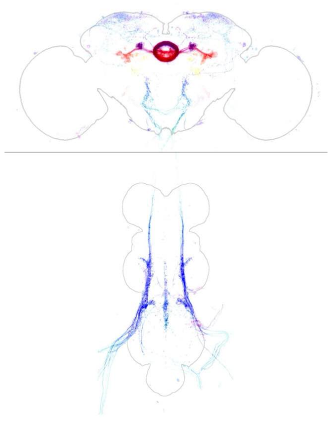

SS62792

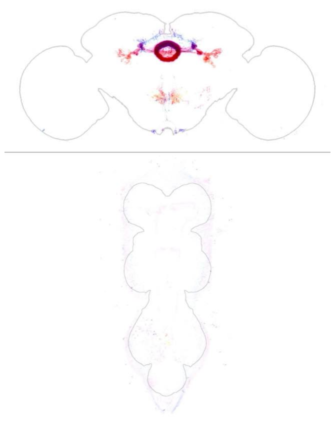

SS62810

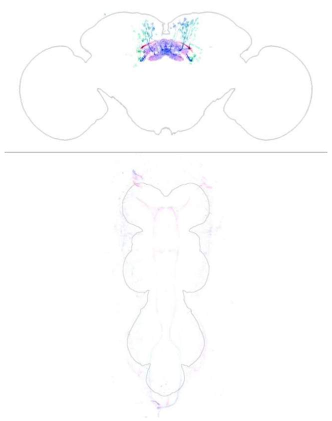

SS62856

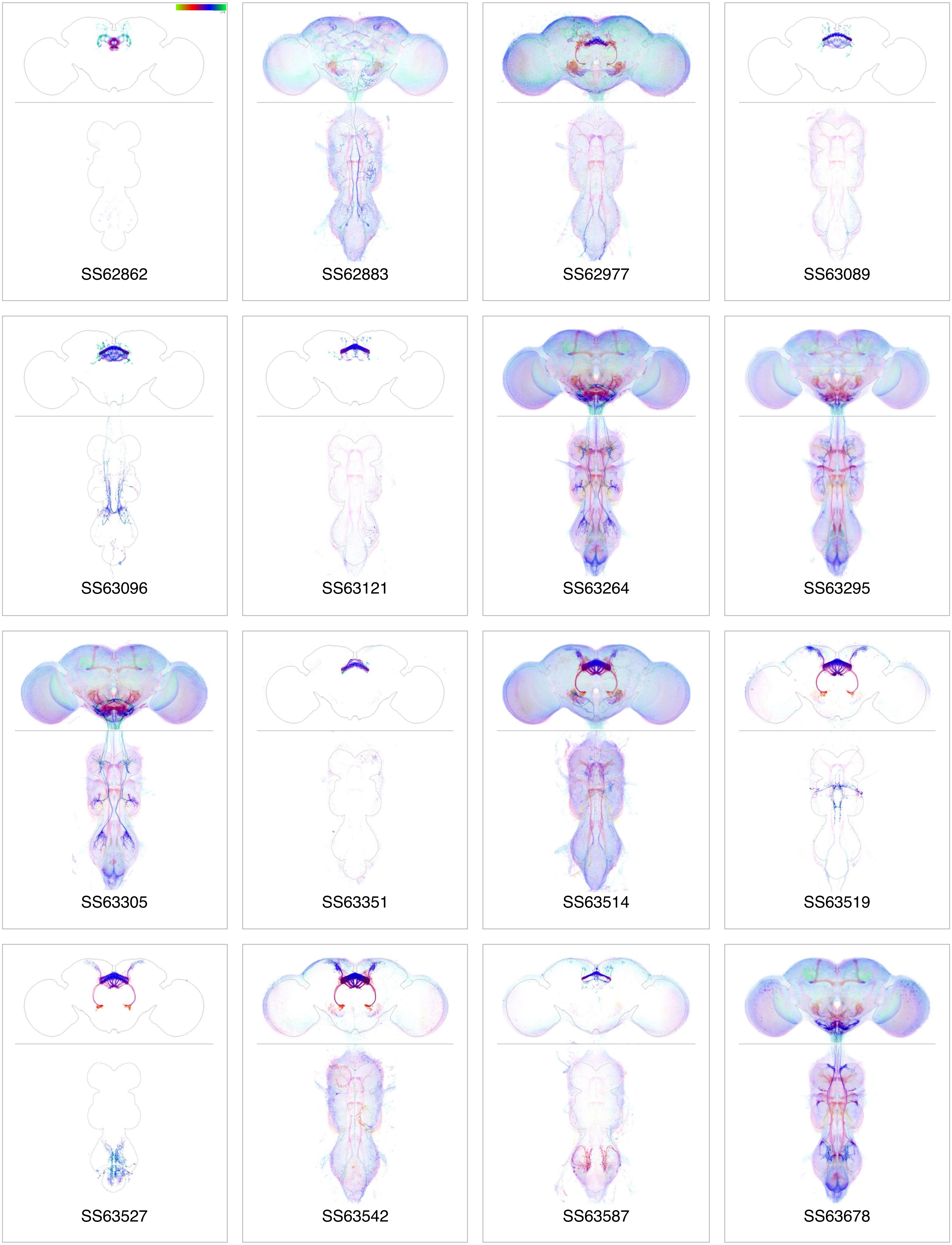
SS62862
SS62883
SS62977
SS63089
SS63096
SS63121
SS63264
SS63295
SS63305
SS63351
SS63514
SS63519
SS63527
SS63542
SS63587
SS63678

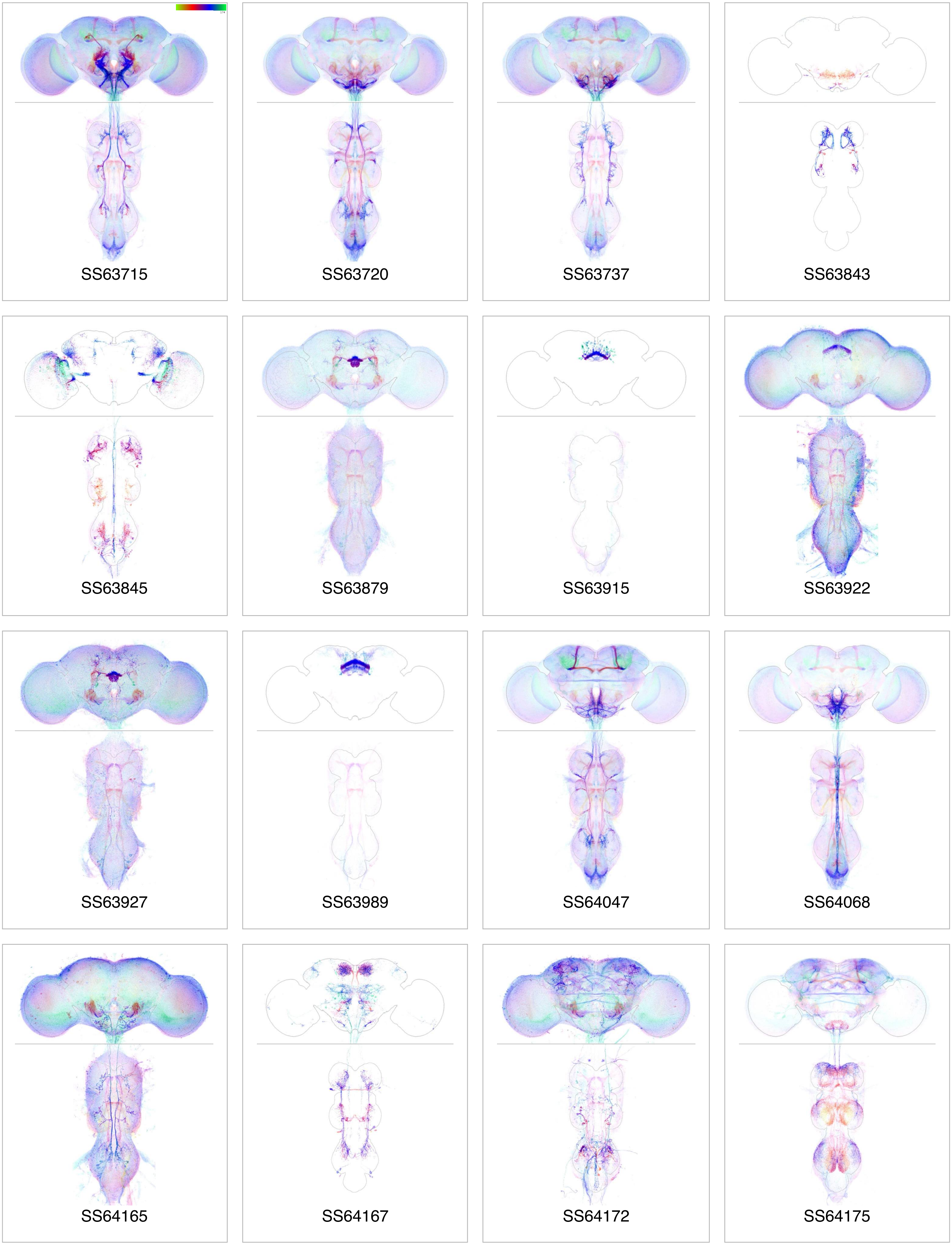
SS63715
SS63720
SS63737
SS63843
SS63845
SS63879
SS63915
SS63922
SS63927
SS63989
SS64047
SS64068
SS64165
SS64167
SS64172
SS64175

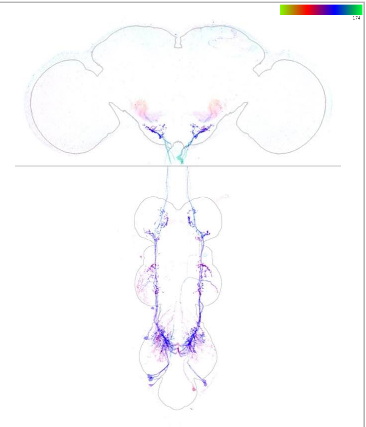
SS64179

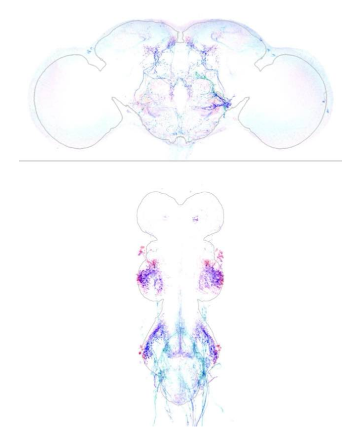
SS64184

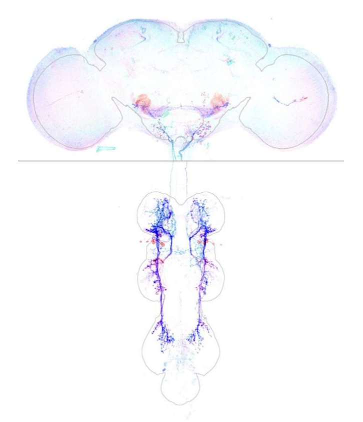
SS64188

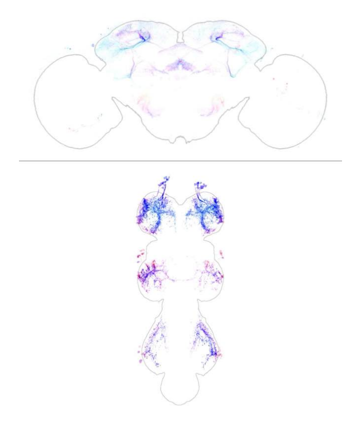
SS64190

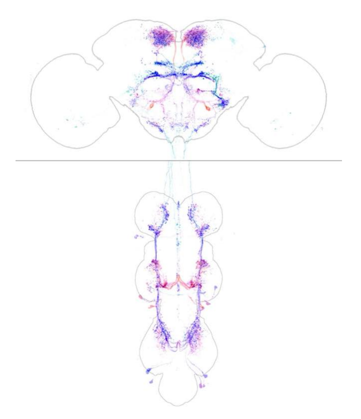
SS64214

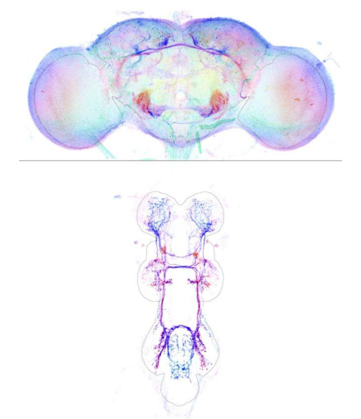
SS64218

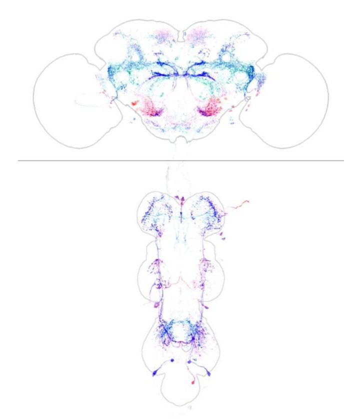
SS64223

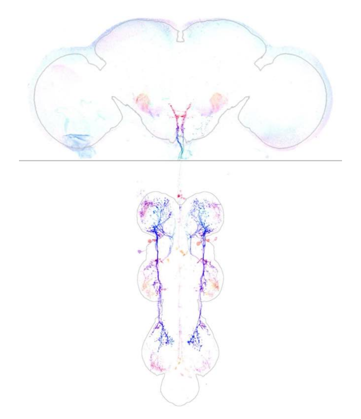
SS64225

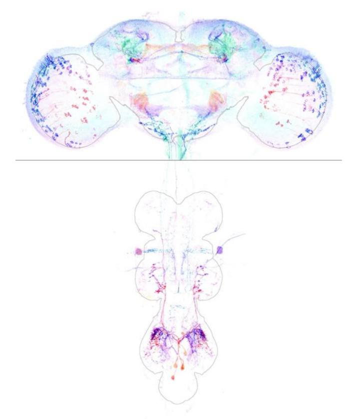
SS64234

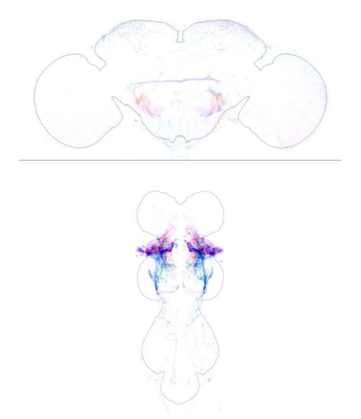
SS64282

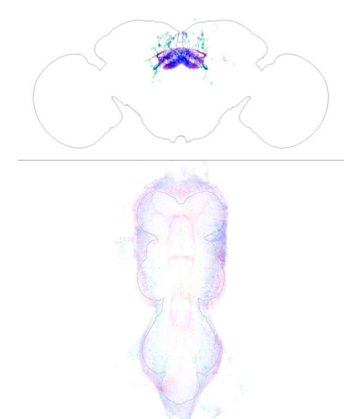
SS64305

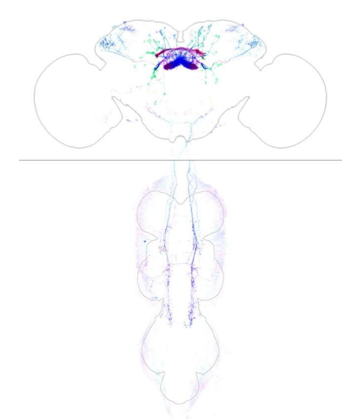
SS64320

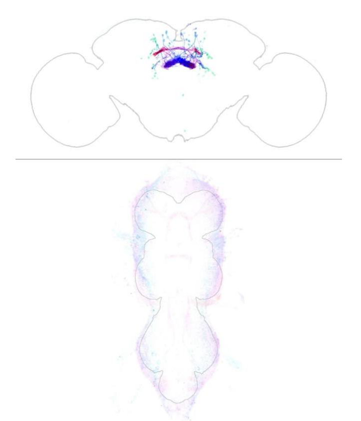
SS64334

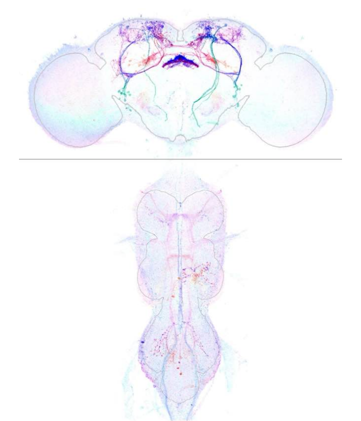
SS64389

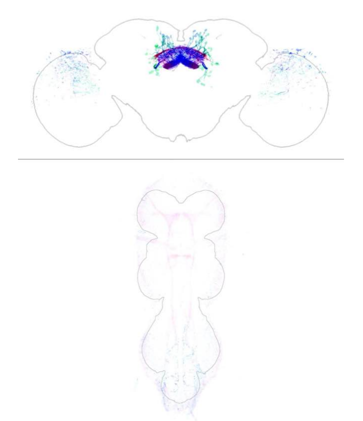
SS64396

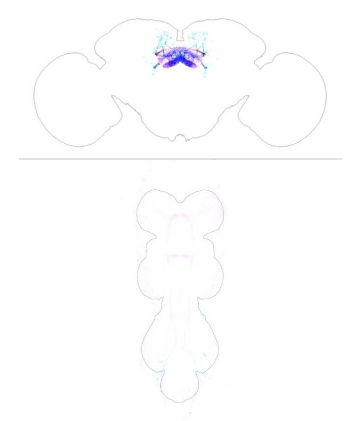
SS64404

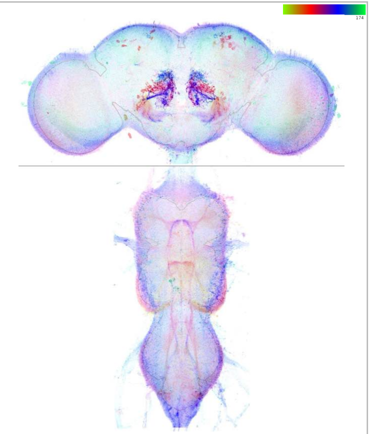

SS64414

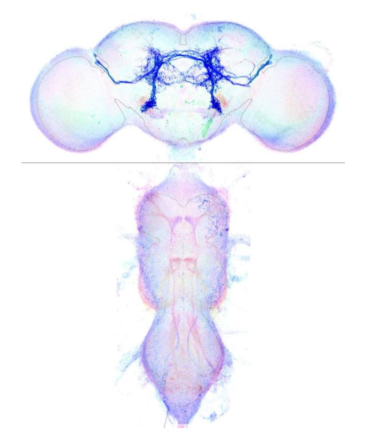

SS64427

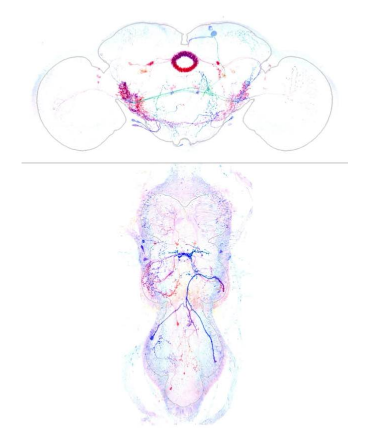

SS64455

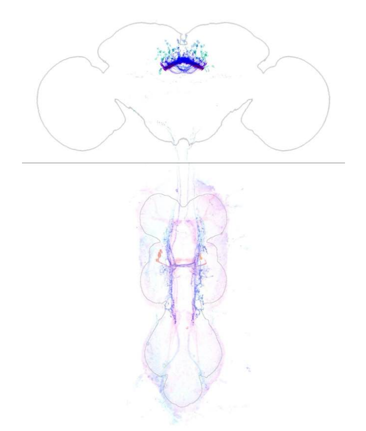

SS64467

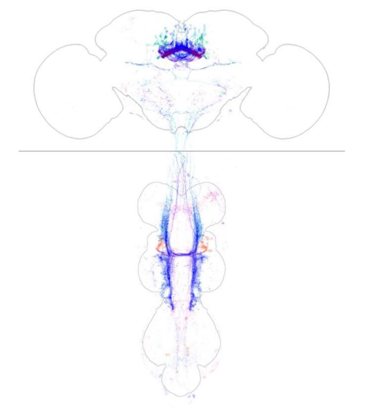

SS64503

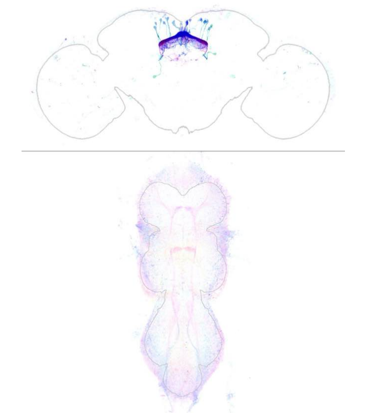

SS64505

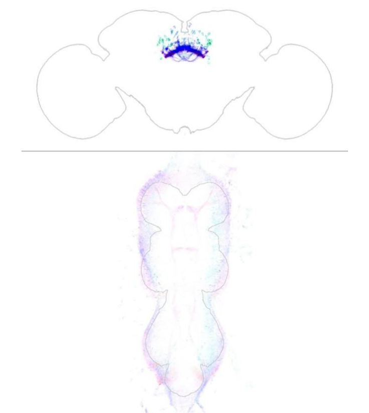

SS64527

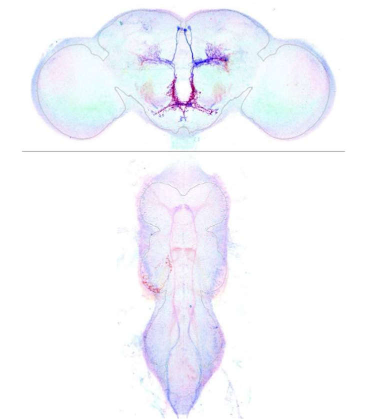

SS64548

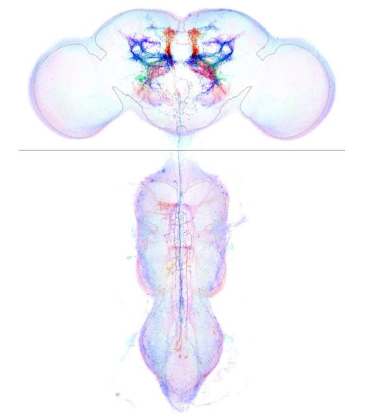

SS64550

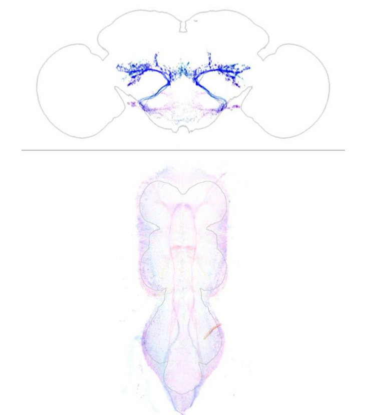

SS64560

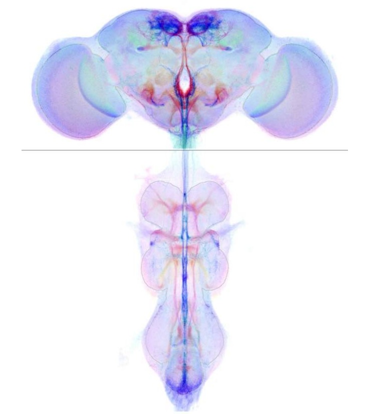

SS64601

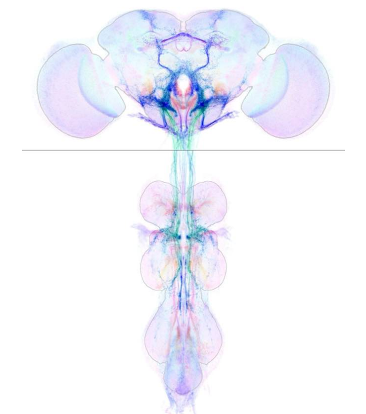

SS64679

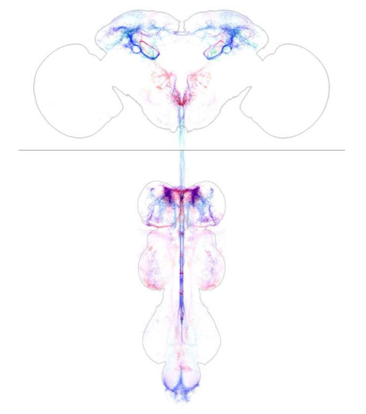

SS64688

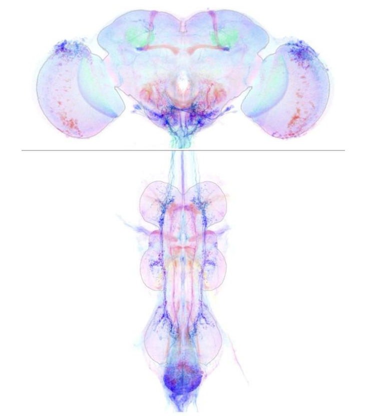

SS64882

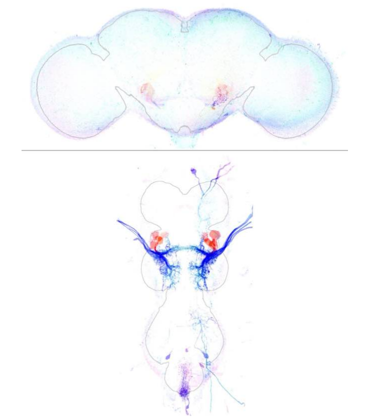

SS64893

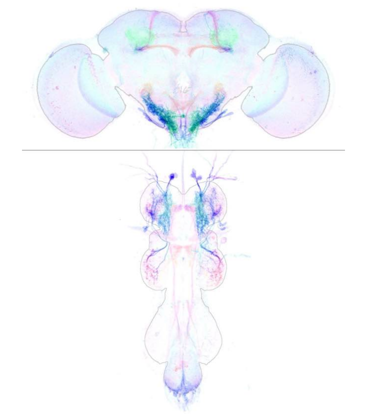

SS64901

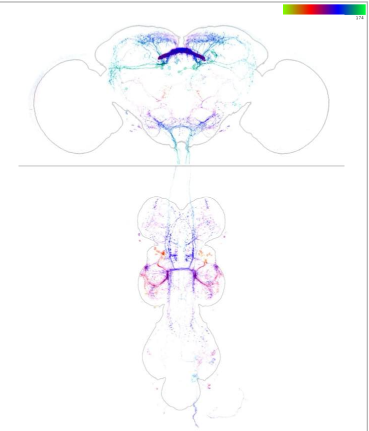

SS65066

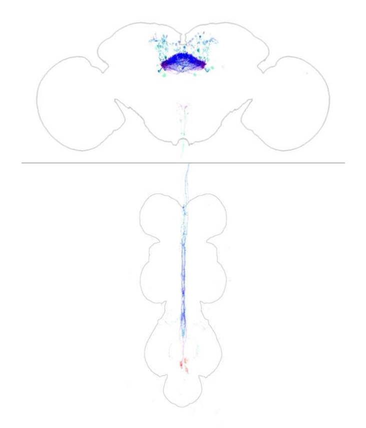

SS65156

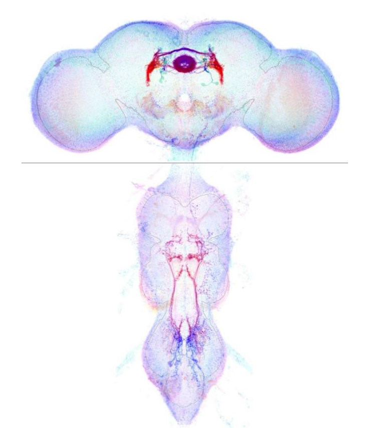

SS65157

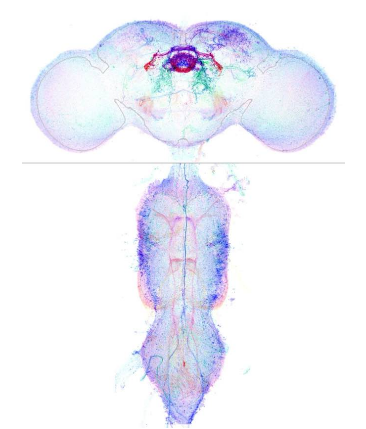

SS65188

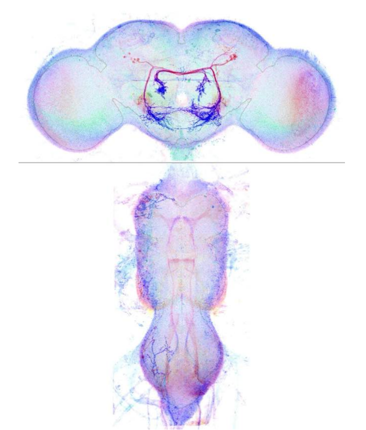

SS65207

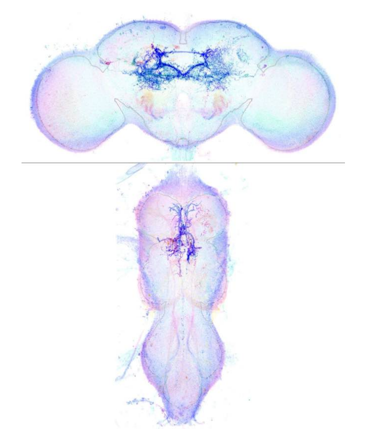

SS65209

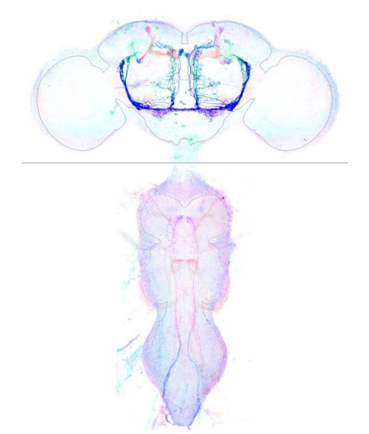

SS65212

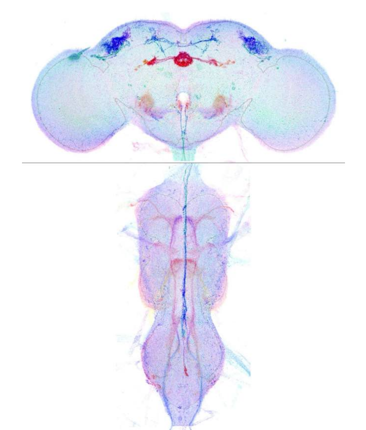

SS65260

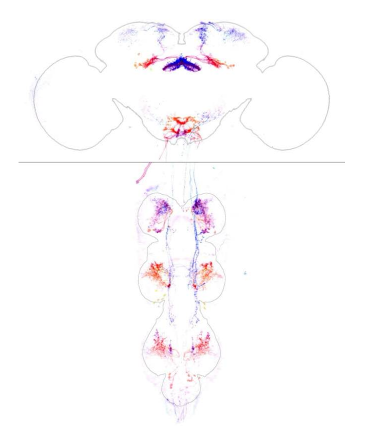

SS65285

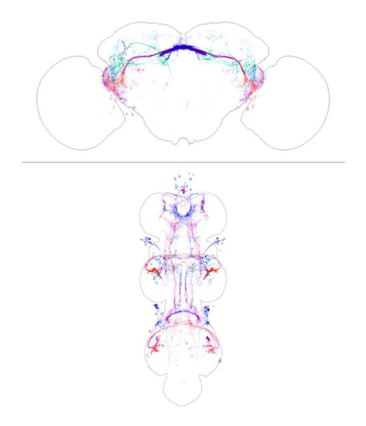

SS65331

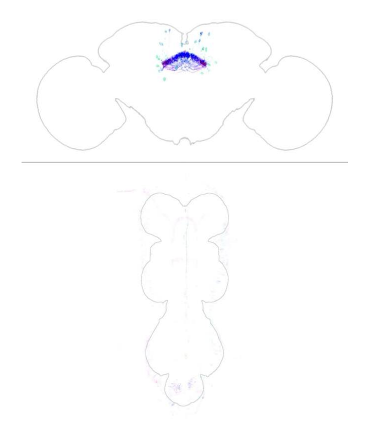

SS65340

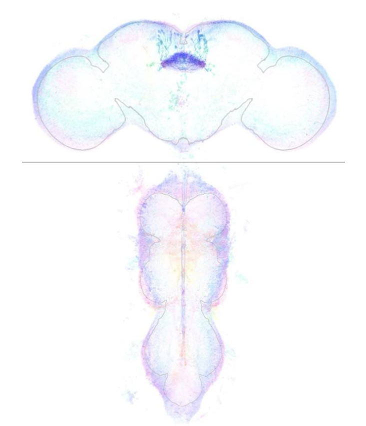

SS65342

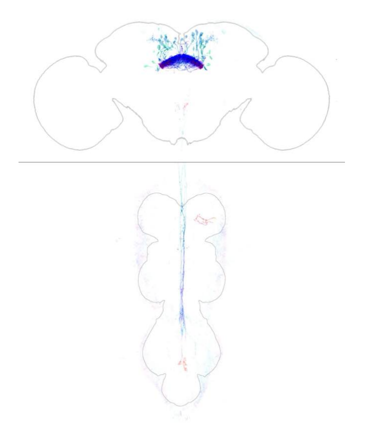

SS65351

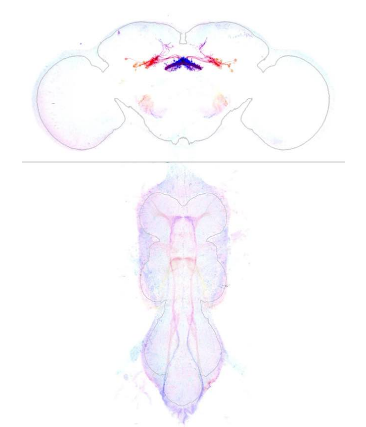

SS65383

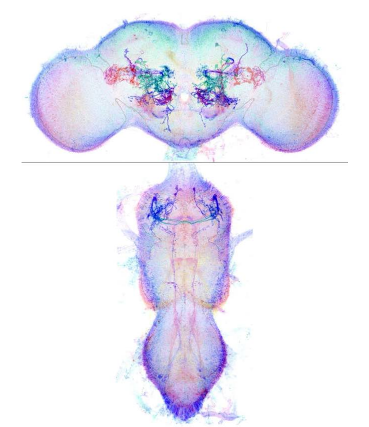

SS65397

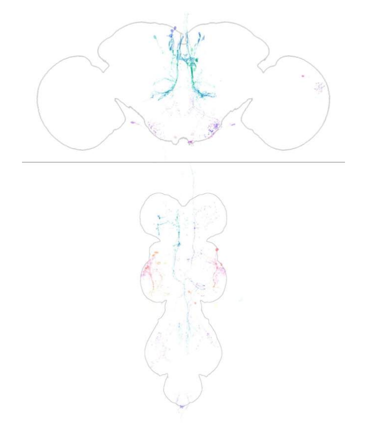

SS65402

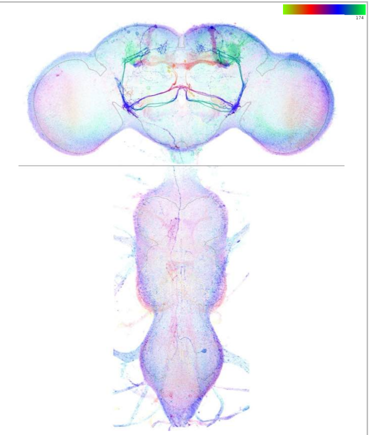
SS65417

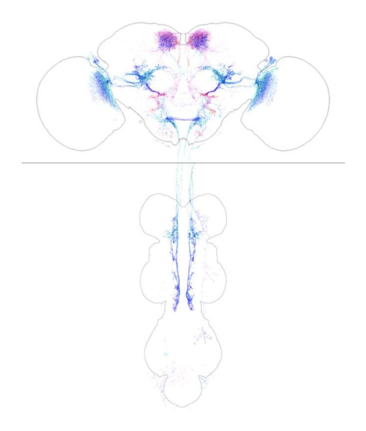
SS65422

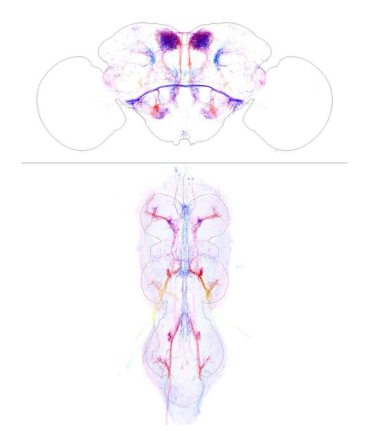
SS65423

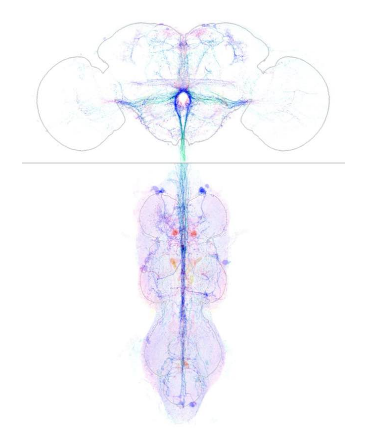
SS65426

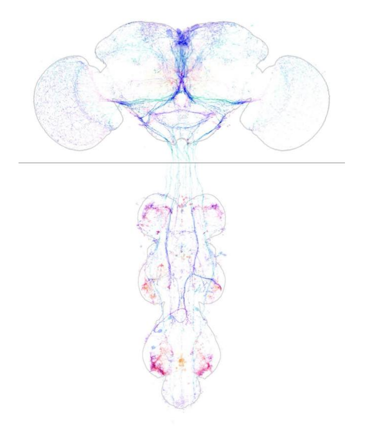
SS65433

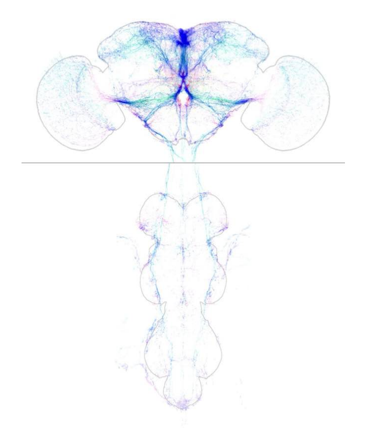
SS65434

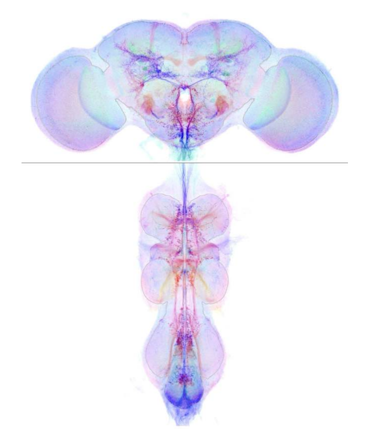
SS65441

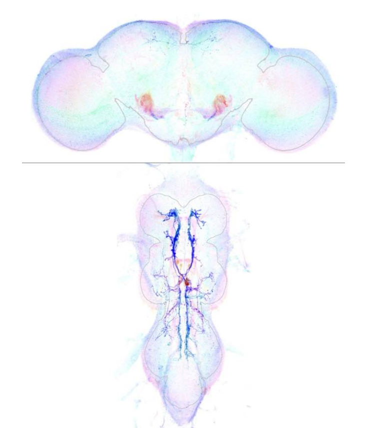
SS65449

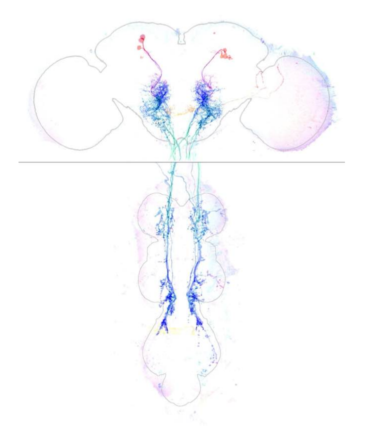
SS65483

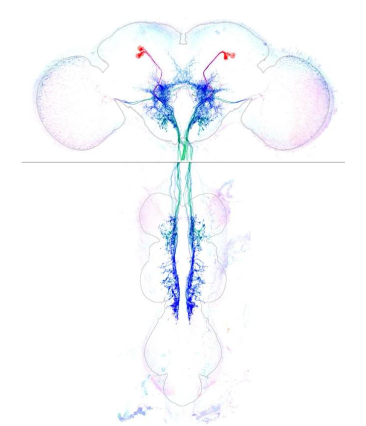
SS65486

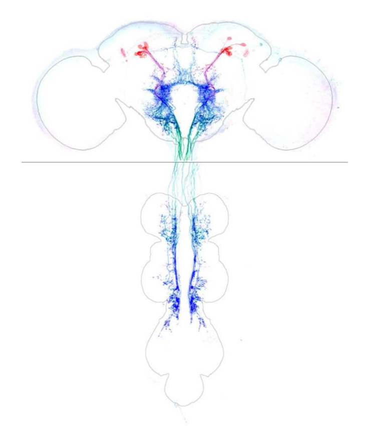
SS65487

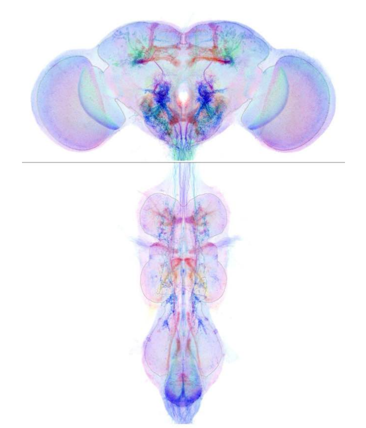
SS65544

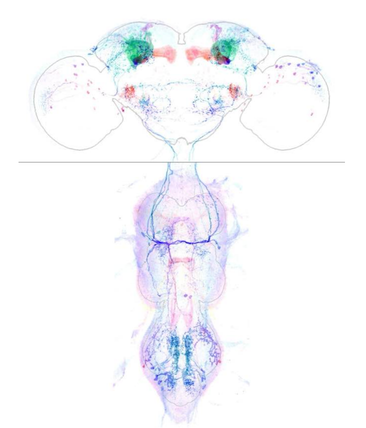
SS65564

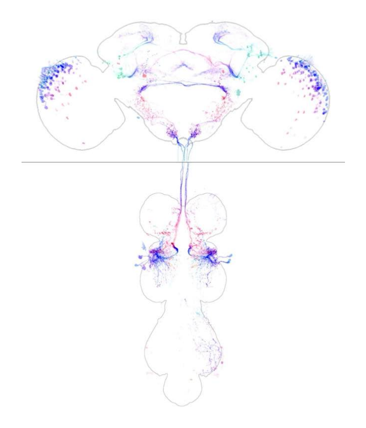
SS65590

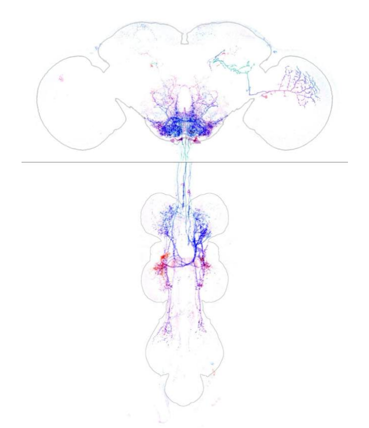
SS65687

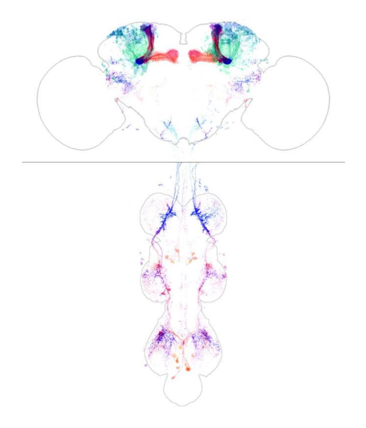
SS65692

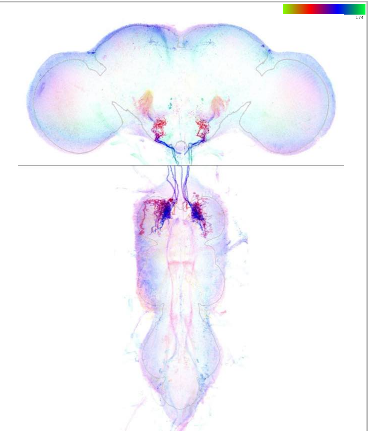
SS65693

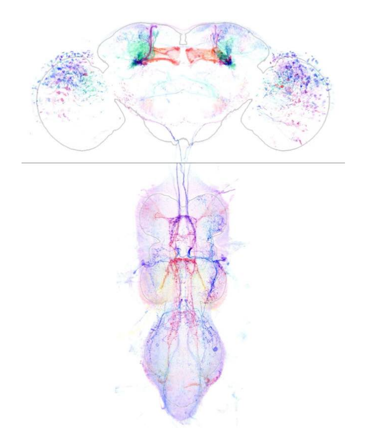
SS65700

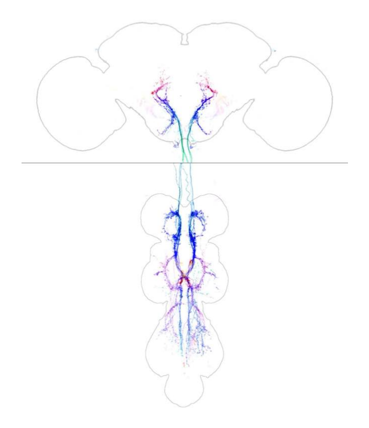
SS65706

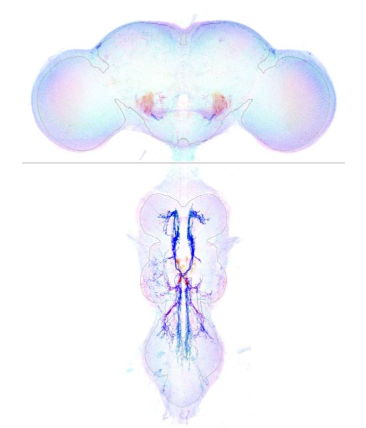
SS65710

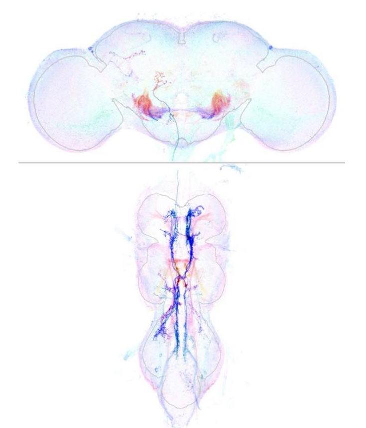
SS65713

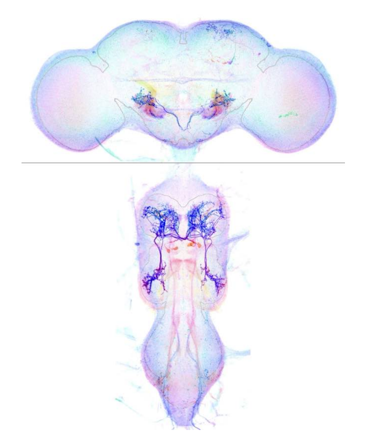
SS65765

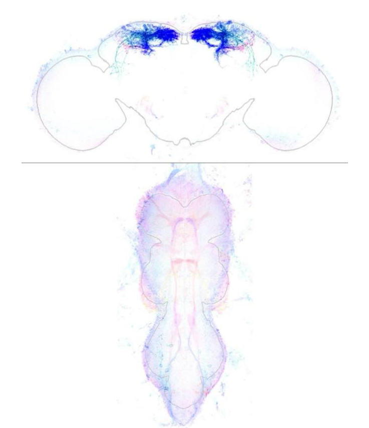
SS65818

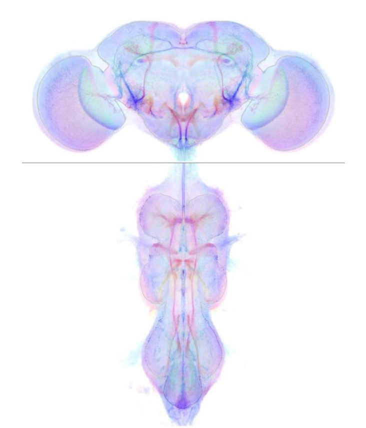
SS65837

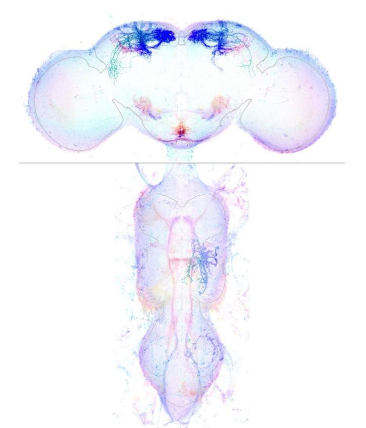
SS65842

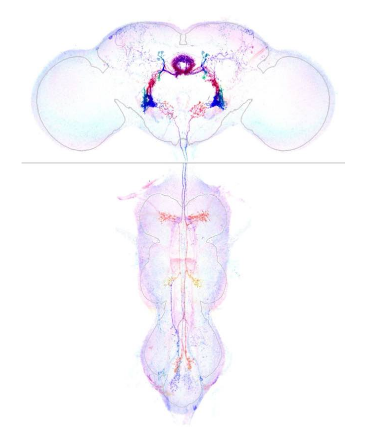
SS65849

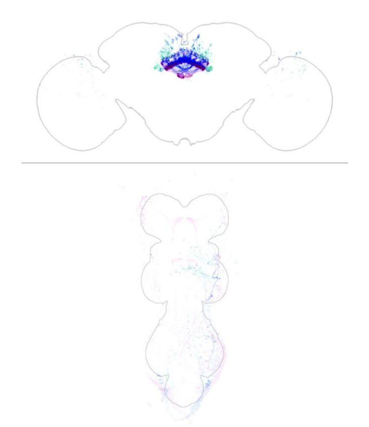
SS65855

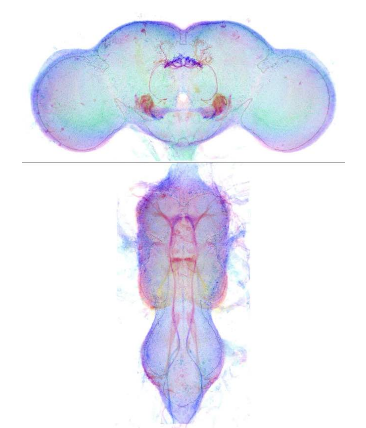
SS65885

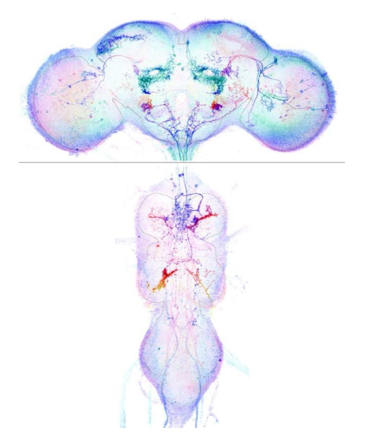
SS65925

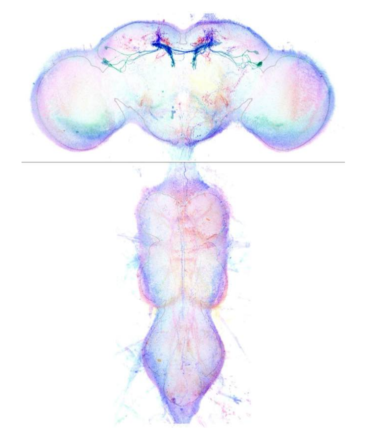
SS65936

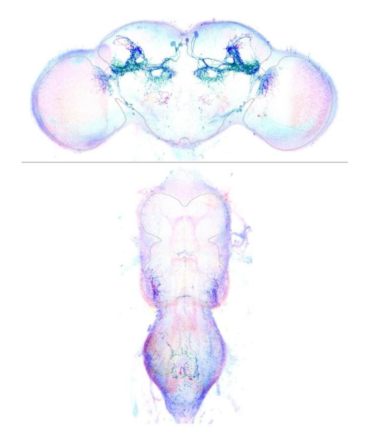
SS65940

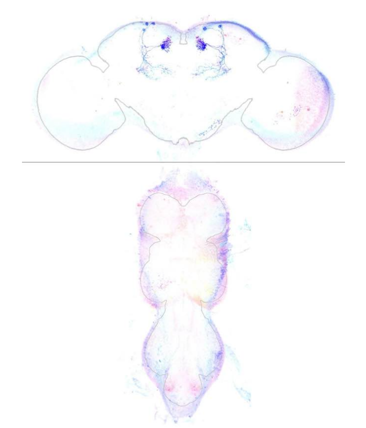
SS65942

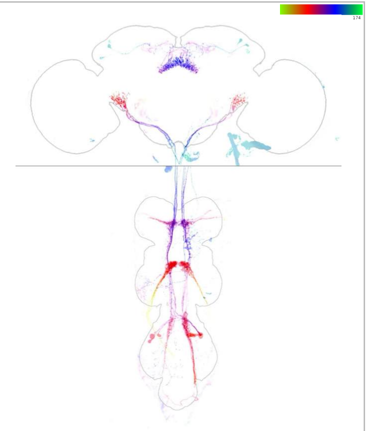
SS66004

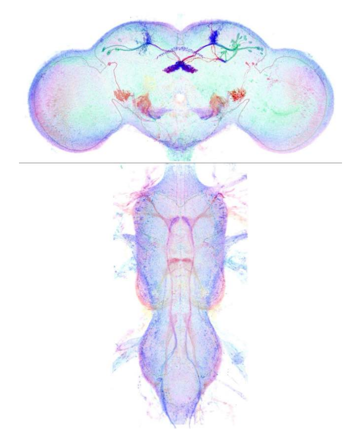
SS66112

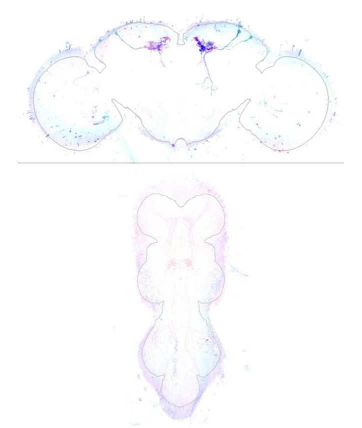
SS66127

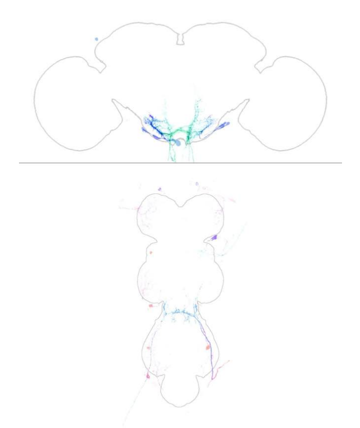
SS66144

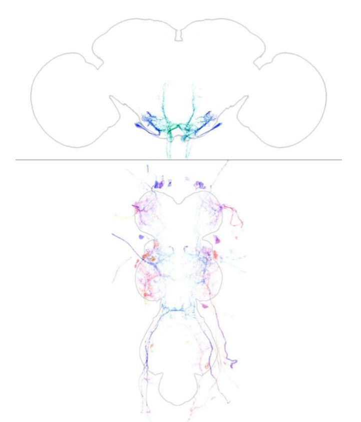
SS66145

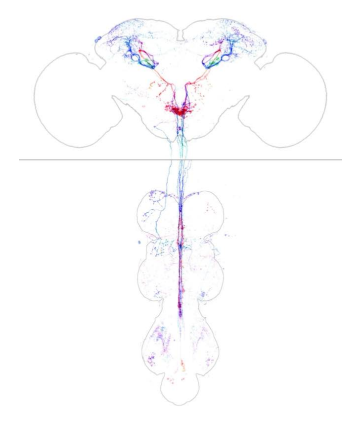
SS66153

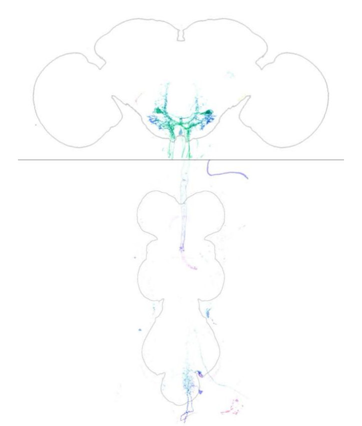
SS66162

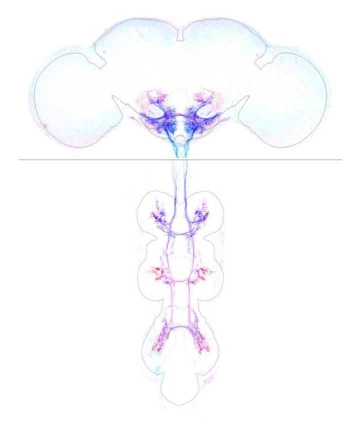
SS66182

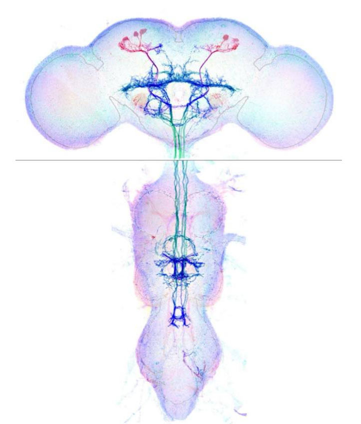
SS66183

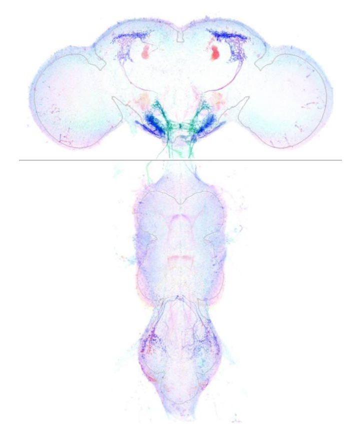
SS66192

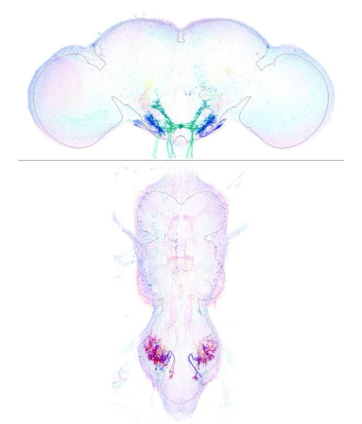
SS66201

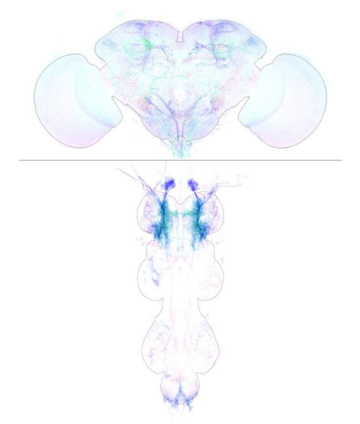
SS66208

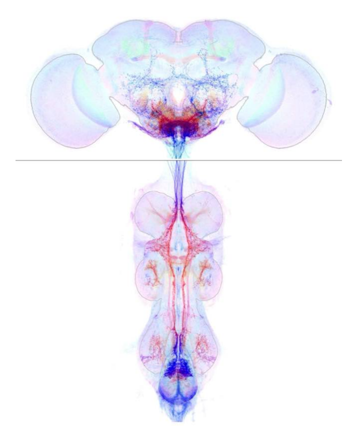
SS66270

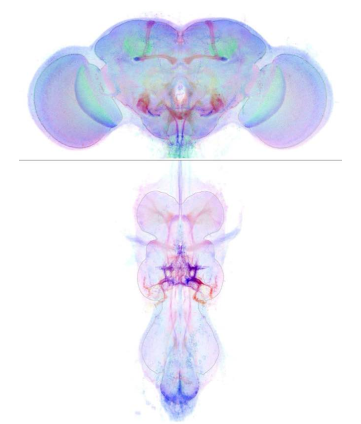
SS66402

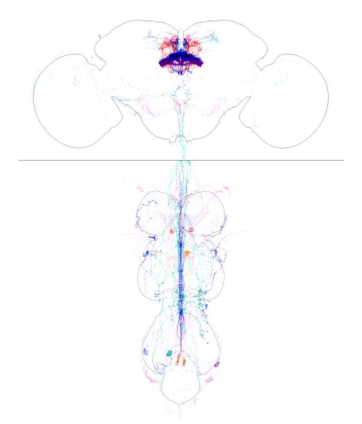
SS66508

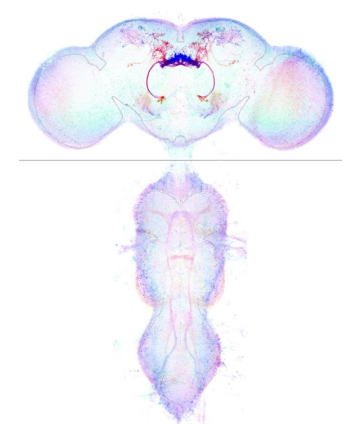
SS66526

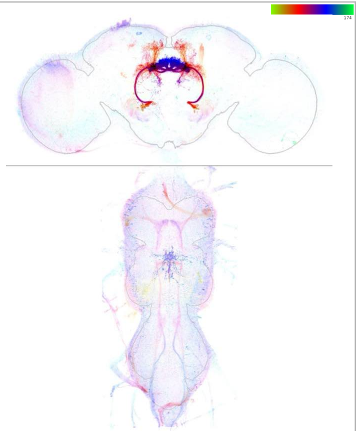
SS66550

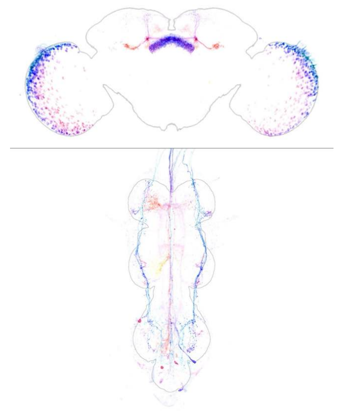
SS66643

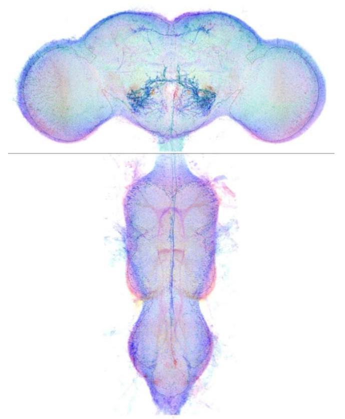
SS66732

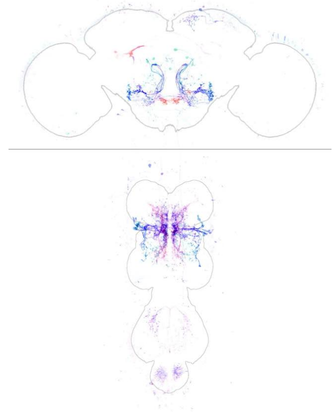
SS66736

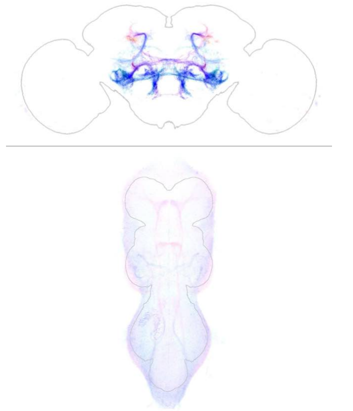
SS66737

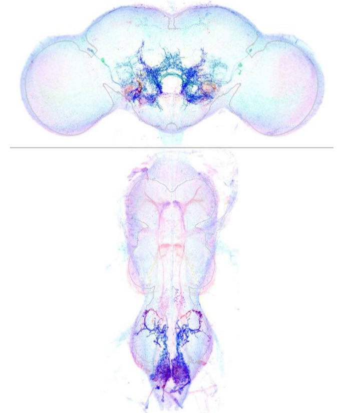
SS66744

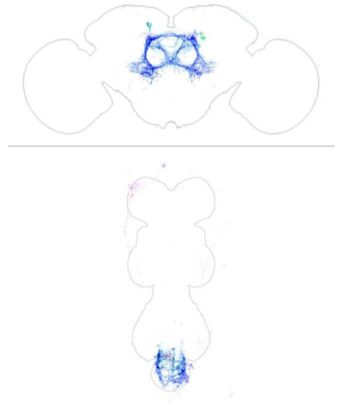
SS66754

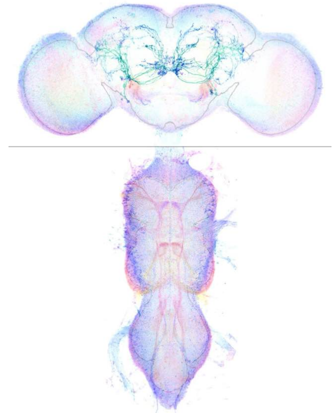
SS66758

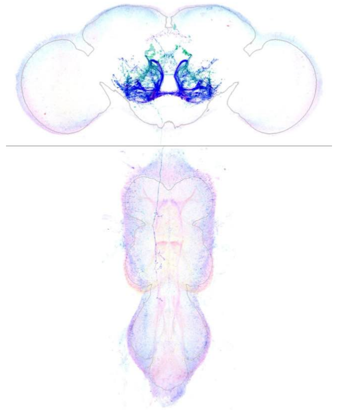
SS66766

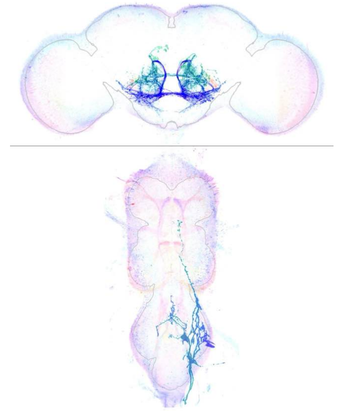
SS66768

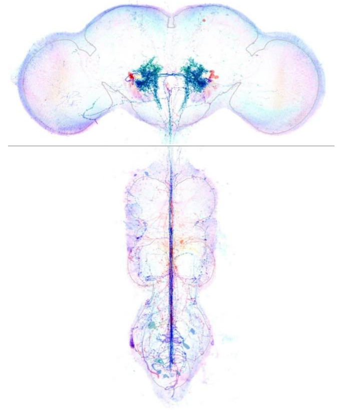
SS66769

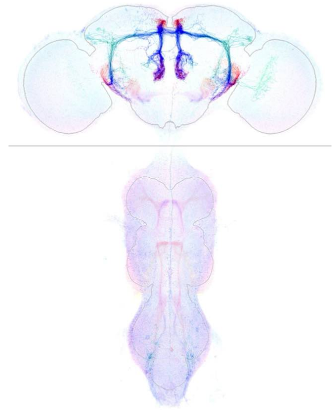
SS66859

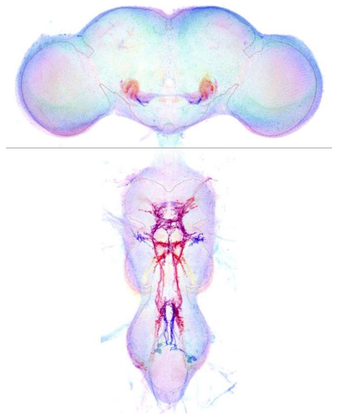
SS66941

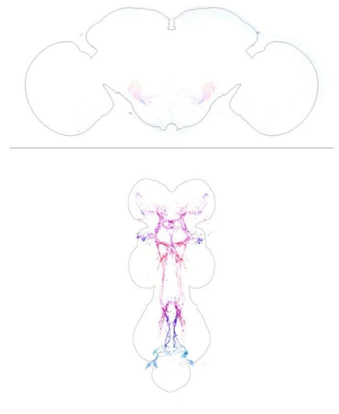
SS66968

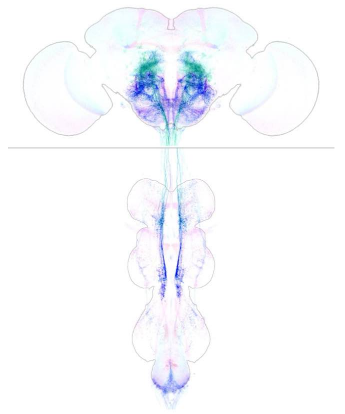
SS67050

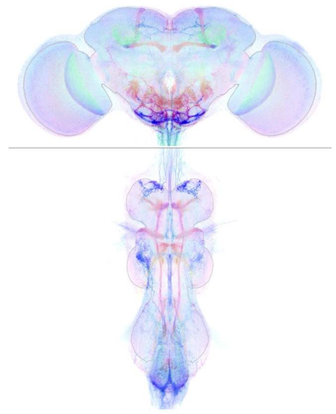
SS67102

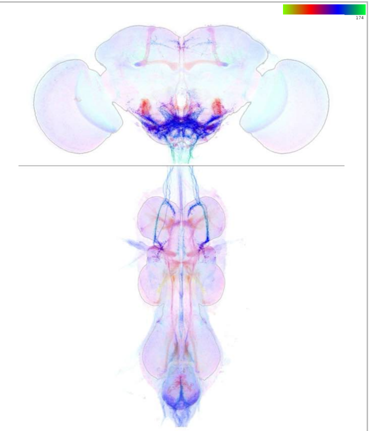
SS67109

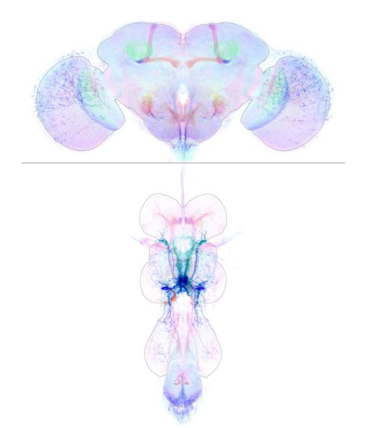
SS67116

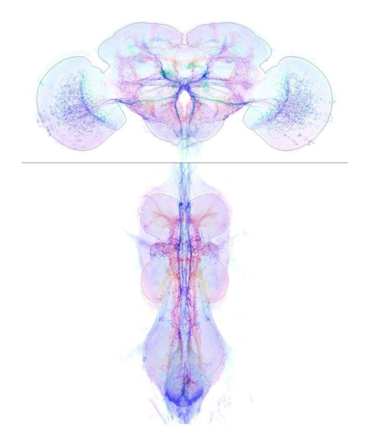
SS67122

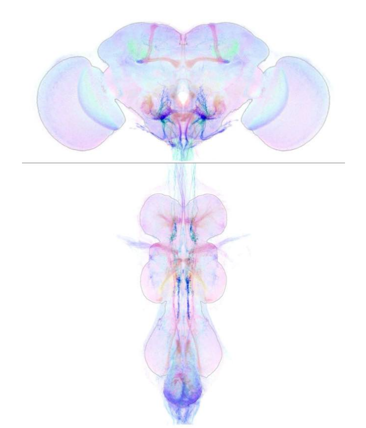
SS67124

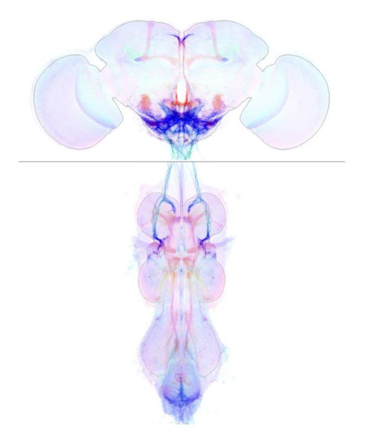
SS67133

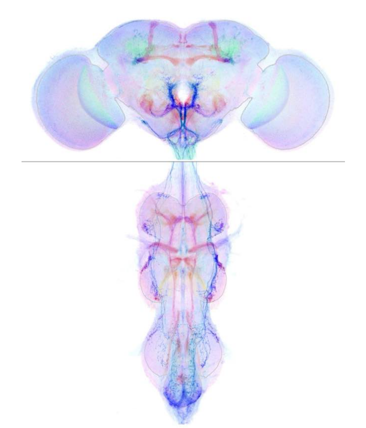
SS67148

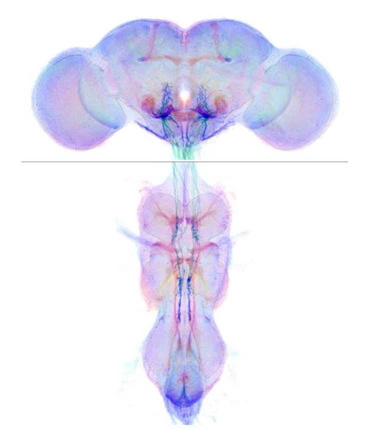
SS67162

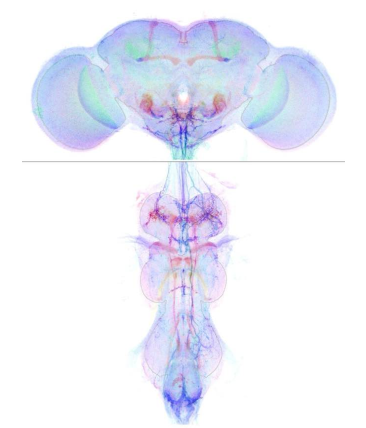
SS67175

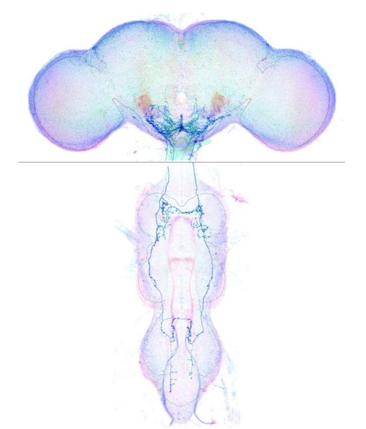
SS67217

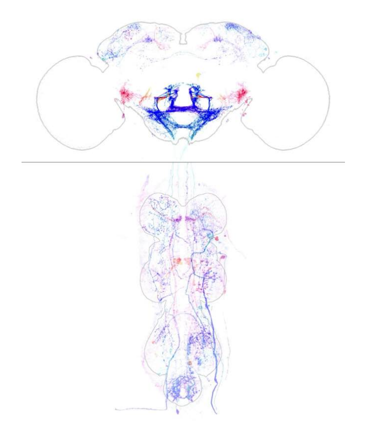
SS67219

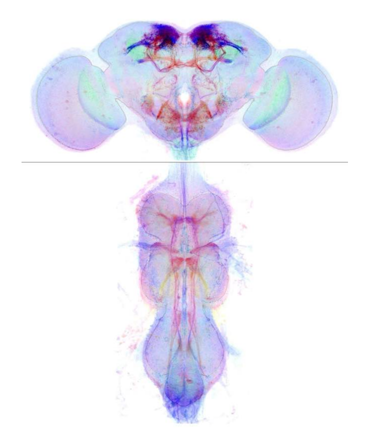
SS67221

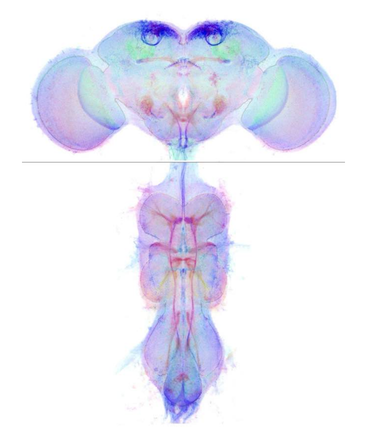
SS67238

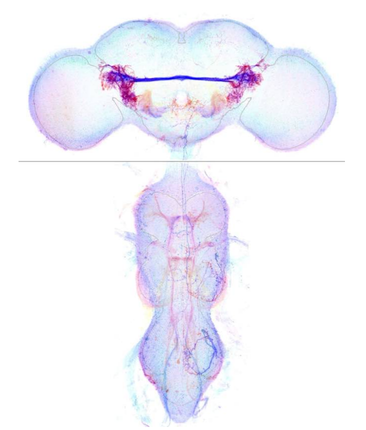
SS67241

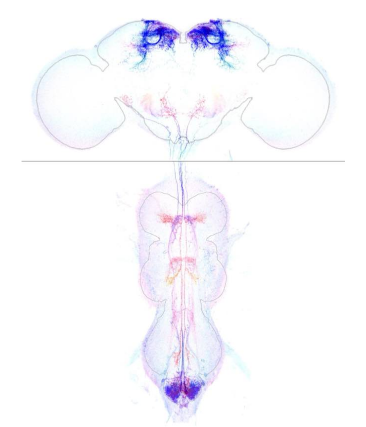
SS67248

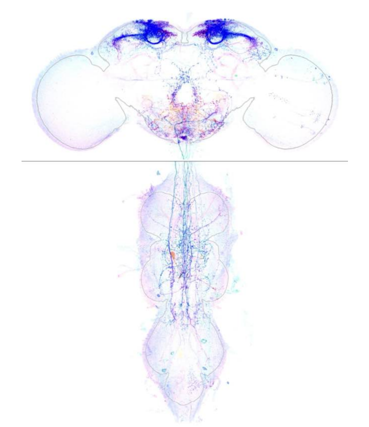
SS67254

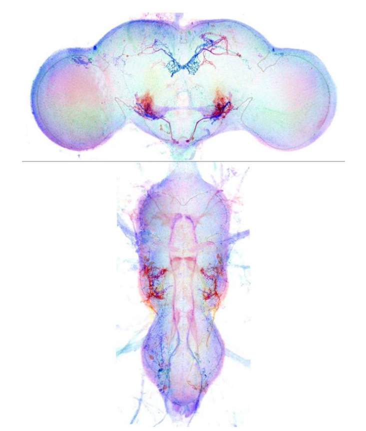
SS67311

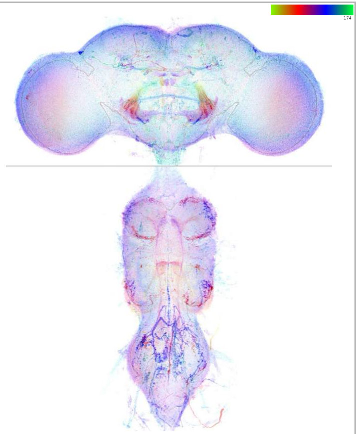
SS67322

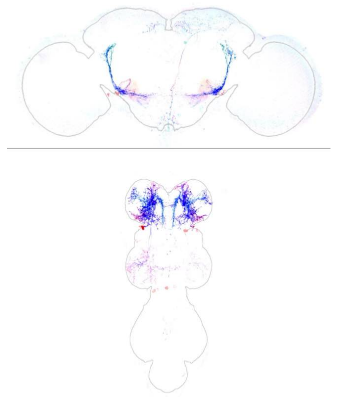
SS67323

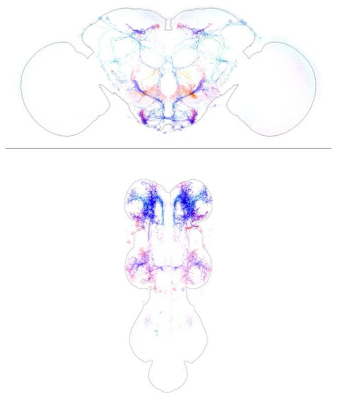
SS67324

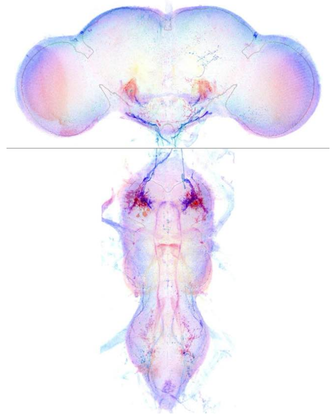
SS67325

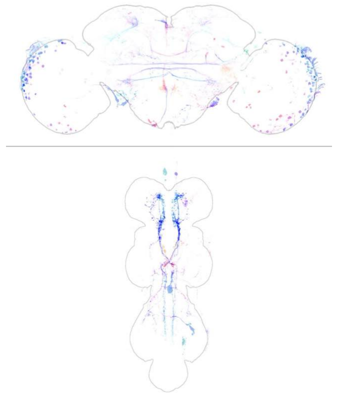
SS67354

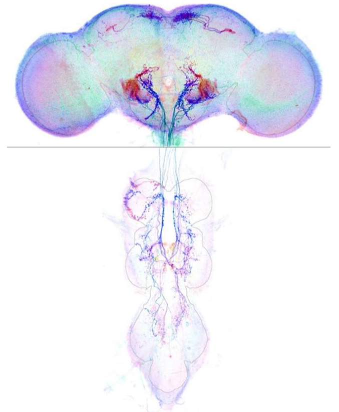
SS67361

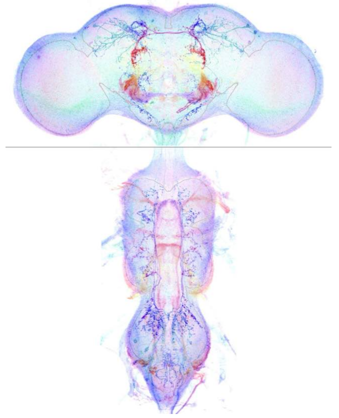
SS67416

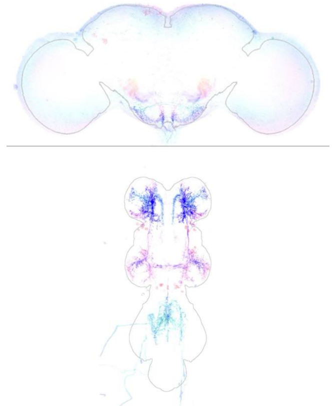
SS67423

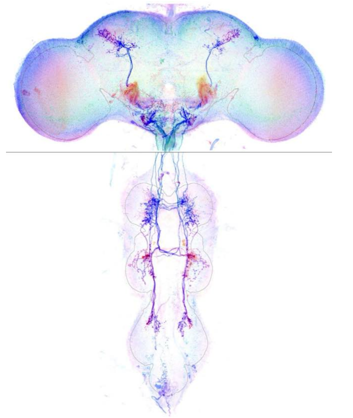
SS67451

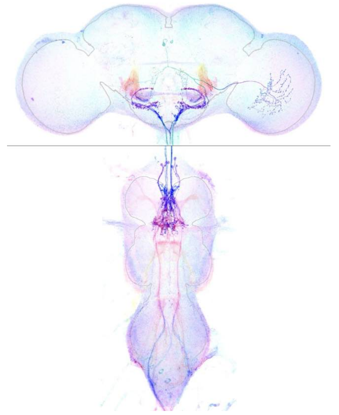
SS67459

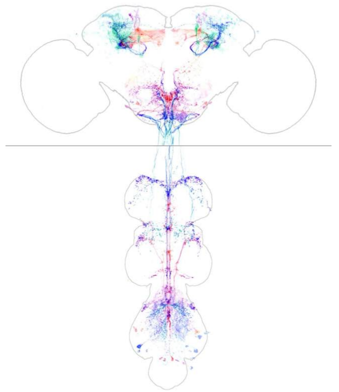
SS67463

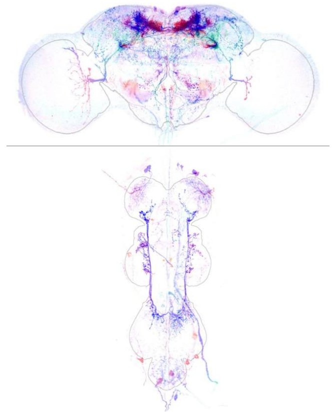
SS67466

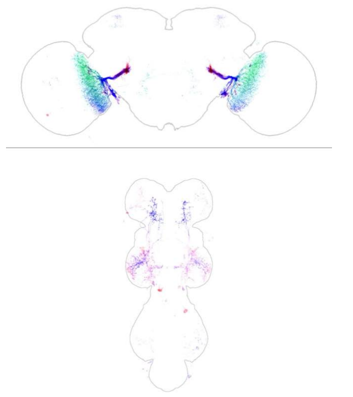
SS67472

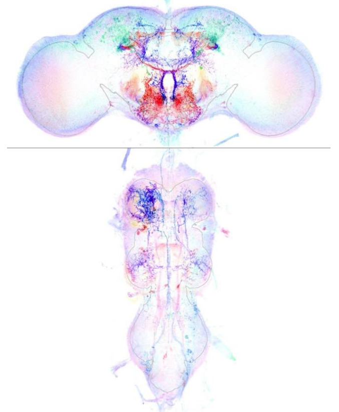
SS67473

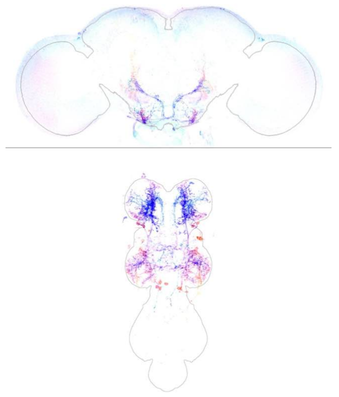
SS67476

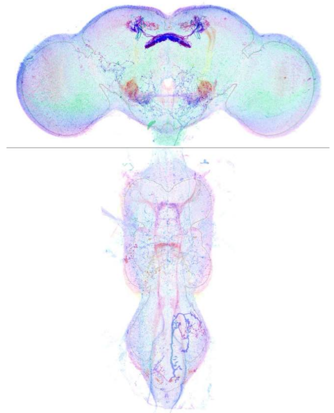
SS67490

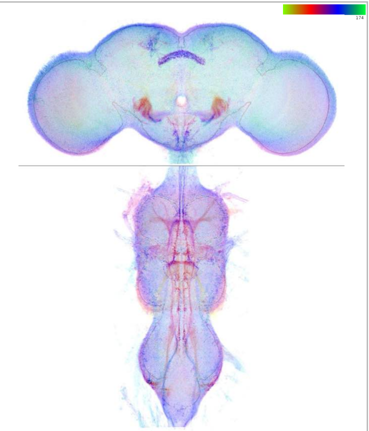
SS67498

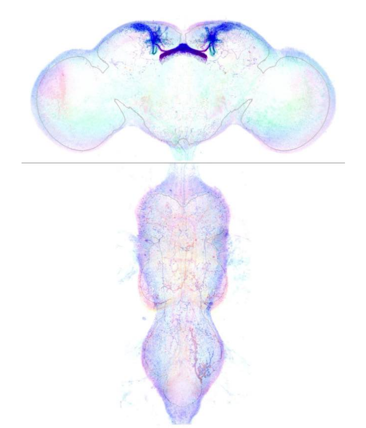
SS67500

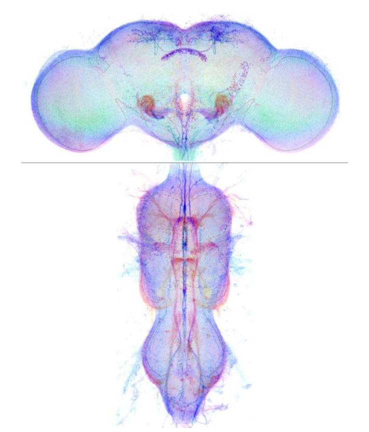
SS67563

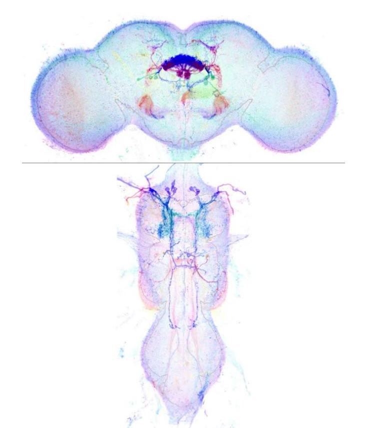
SS67580

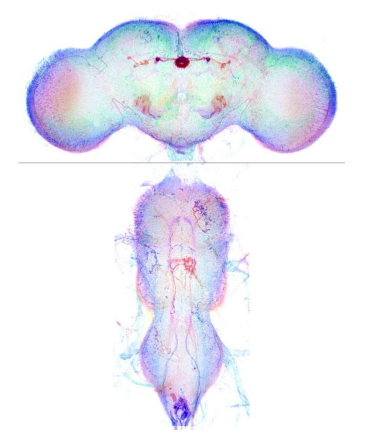
SS67611

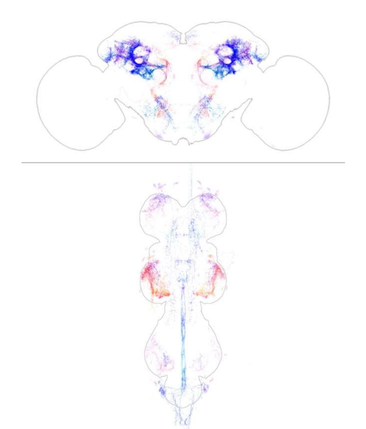
SS67641

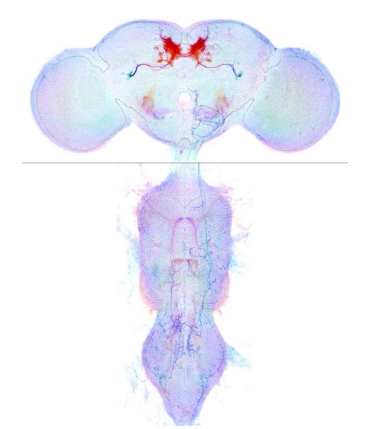
SS67662

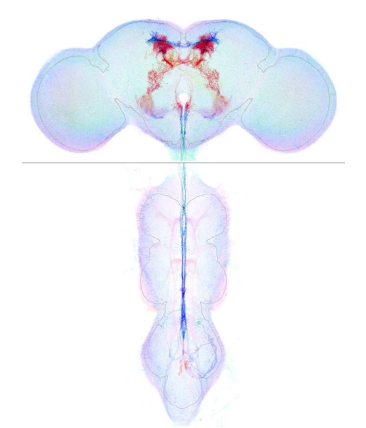
SS67663

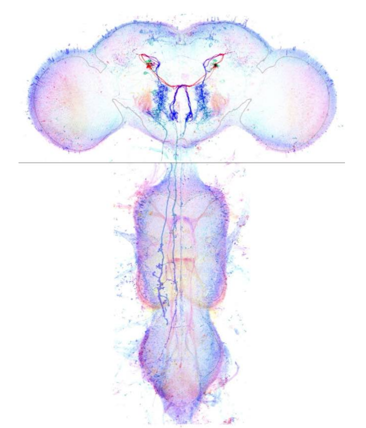
SS67674

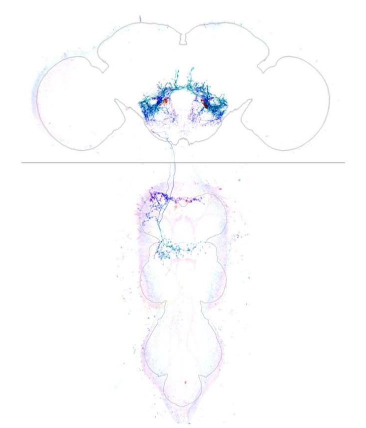
SS67678

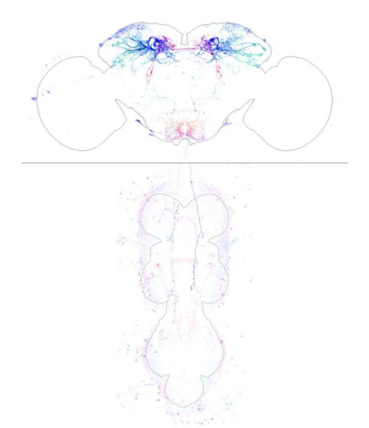
SS67683

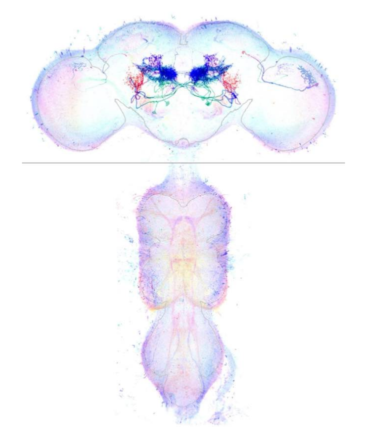
SS67692

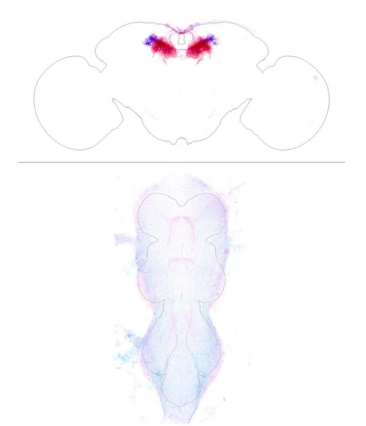
SS67728

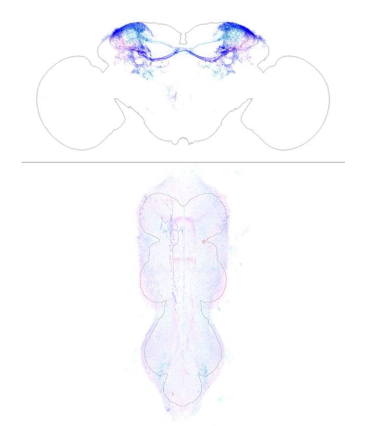
SS67739

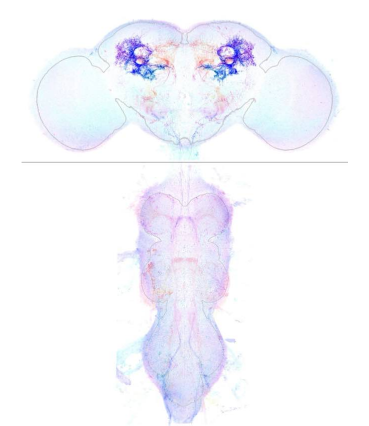
SS67741

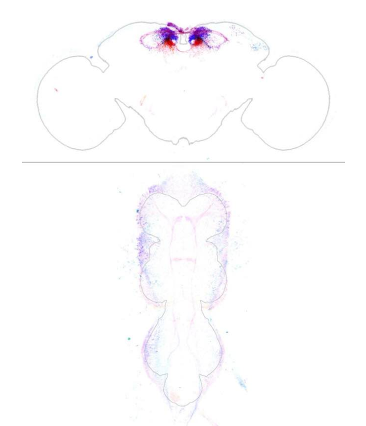
SS67754

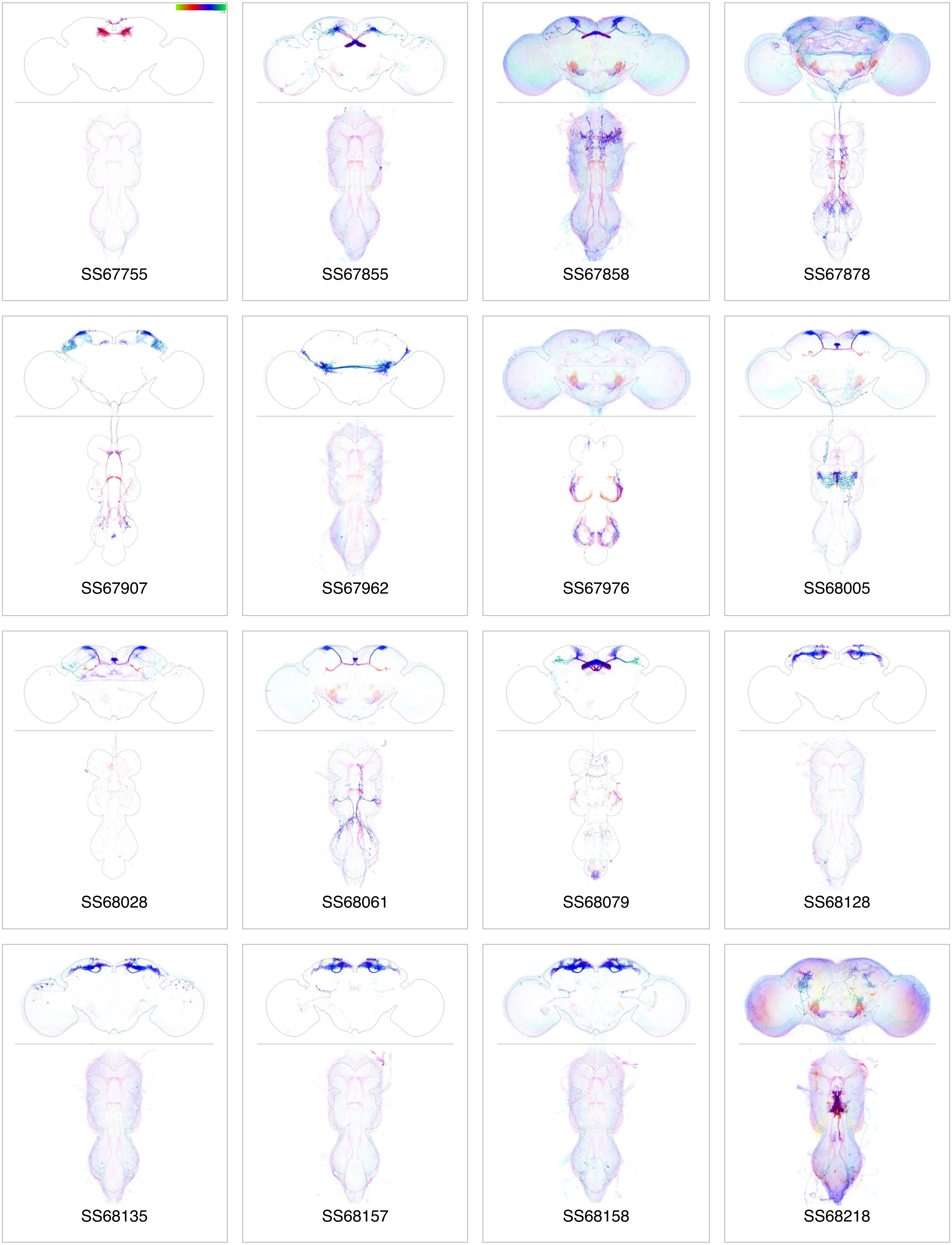
SS67755
SS67855
SS67858
SS67878
SS67907
SS67962
SS67976
SS68005
SS68028
SS68061
SS68079
SS68128
SS68135
SS68157
SS68158
SS68218

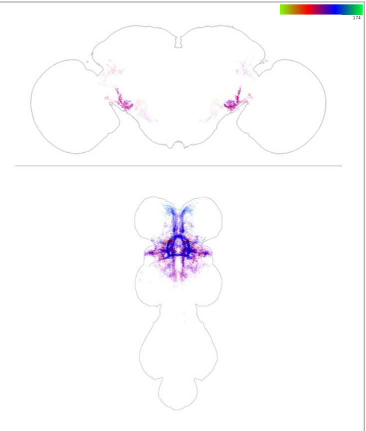

SS68219

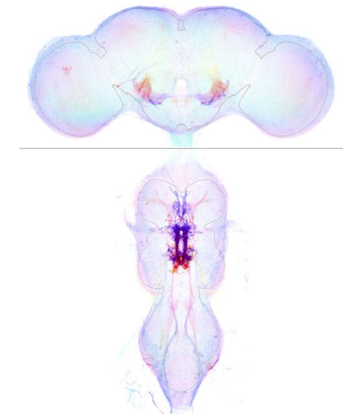

SS68220

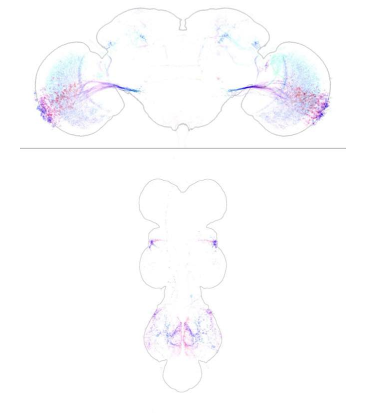

SS68223

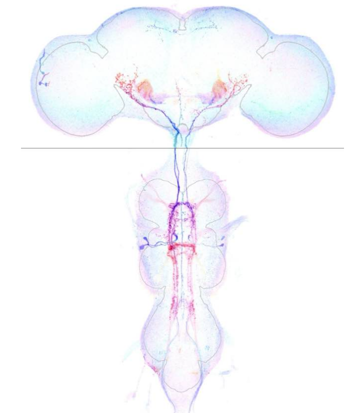

SS68237

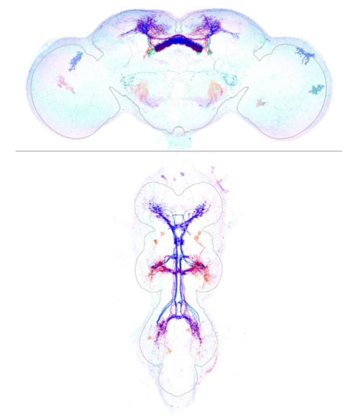

SS68285

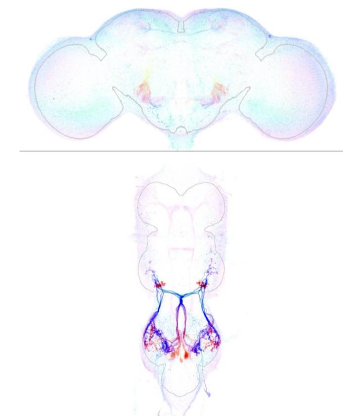

SS68298

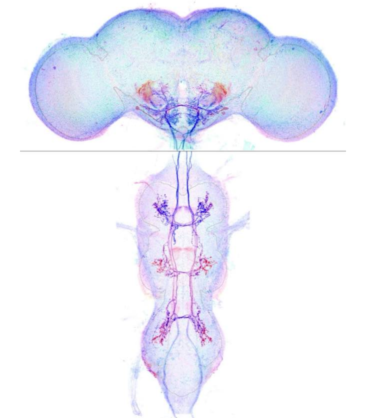

SS68371

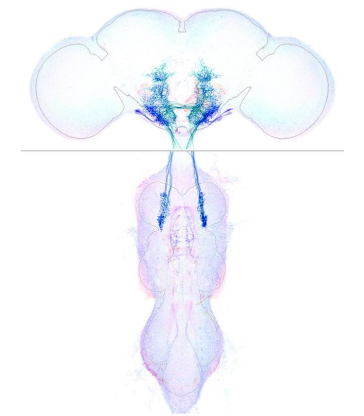

SS68377

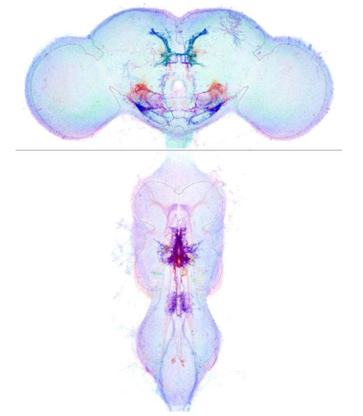

SS68428

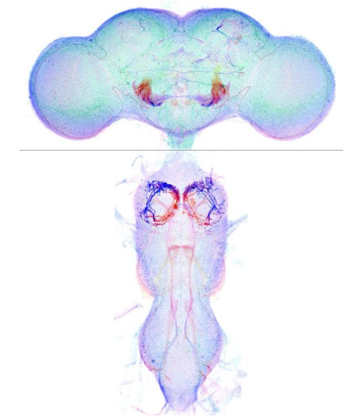

SS68443

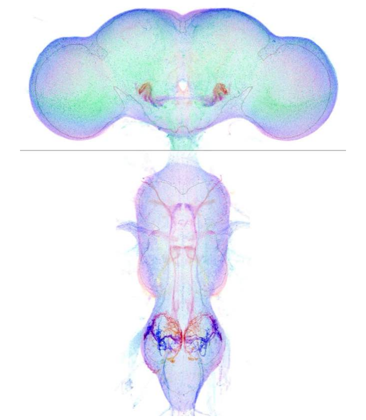

SS68466

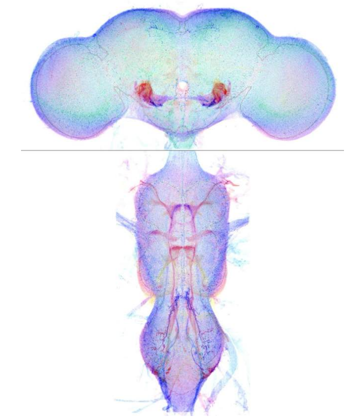

SS68475

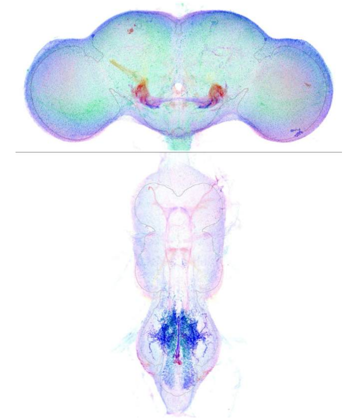

SS68512

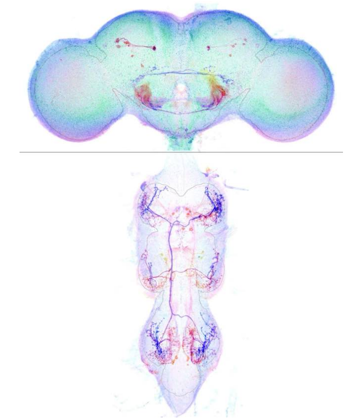

SS68544

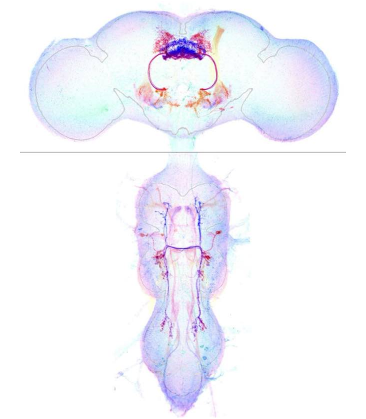

SS68610

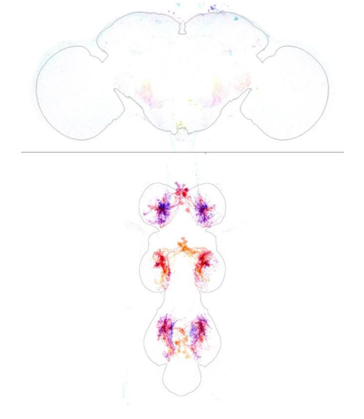

SS68719

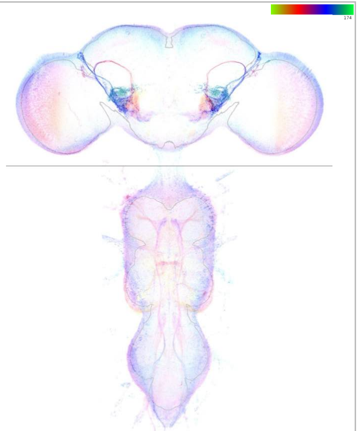
SS69011

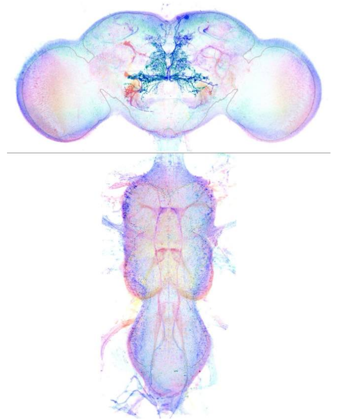
SS69020

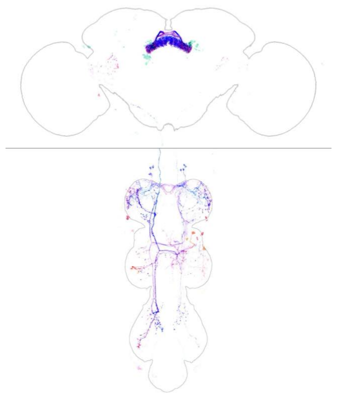
SS69089

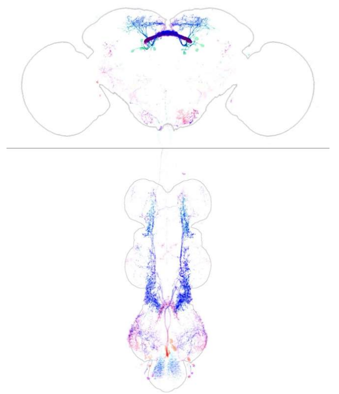
SS69117

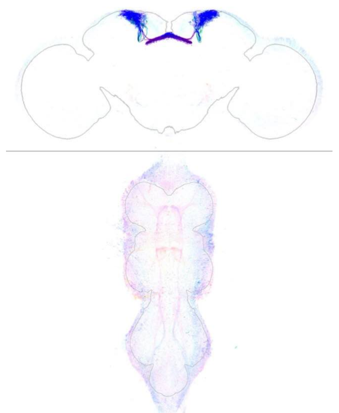
SS69168

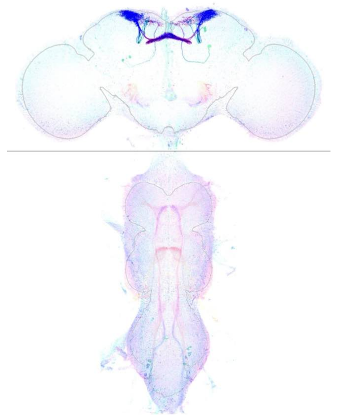
SS69170

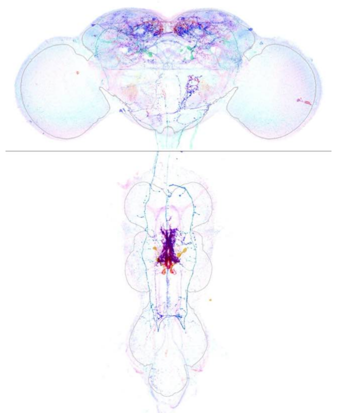
SS69296

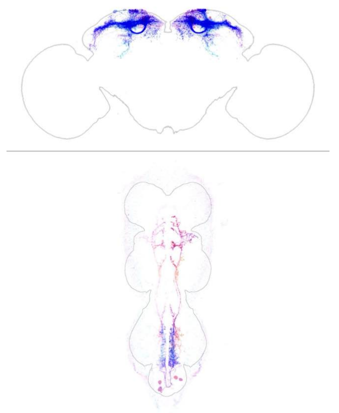
SS69402

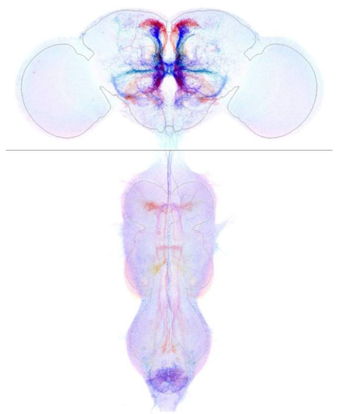
SS69505

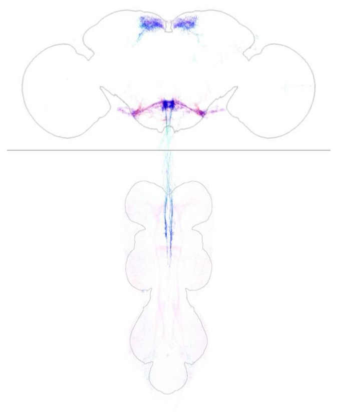
SS69506

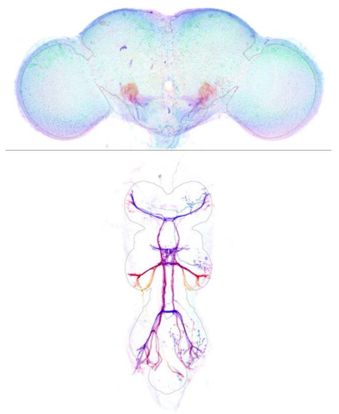
SS69586

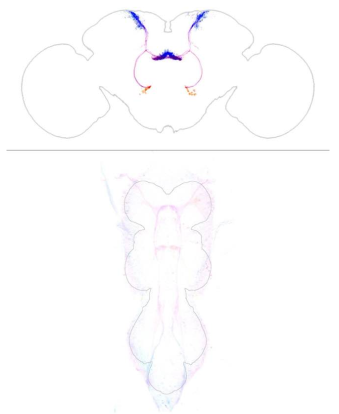
SS69677

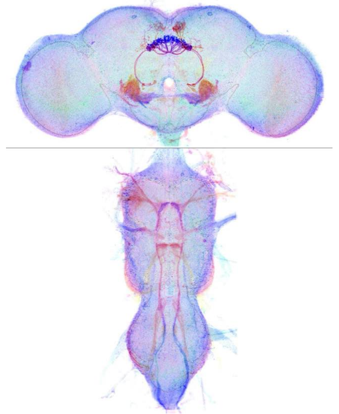
SS69713

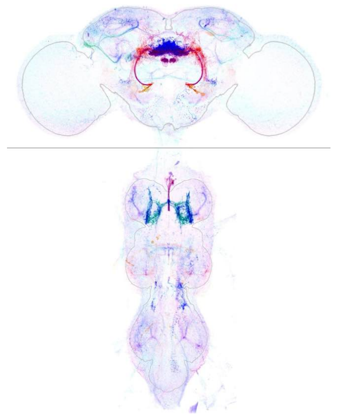
SS69904

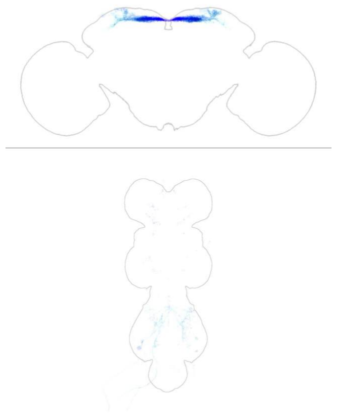
SS69909

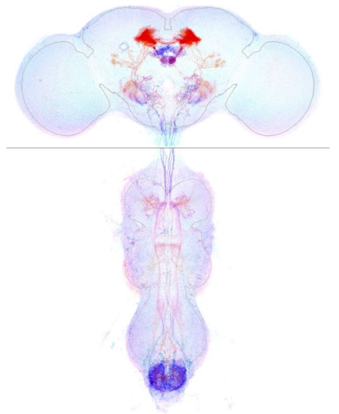
SS69910

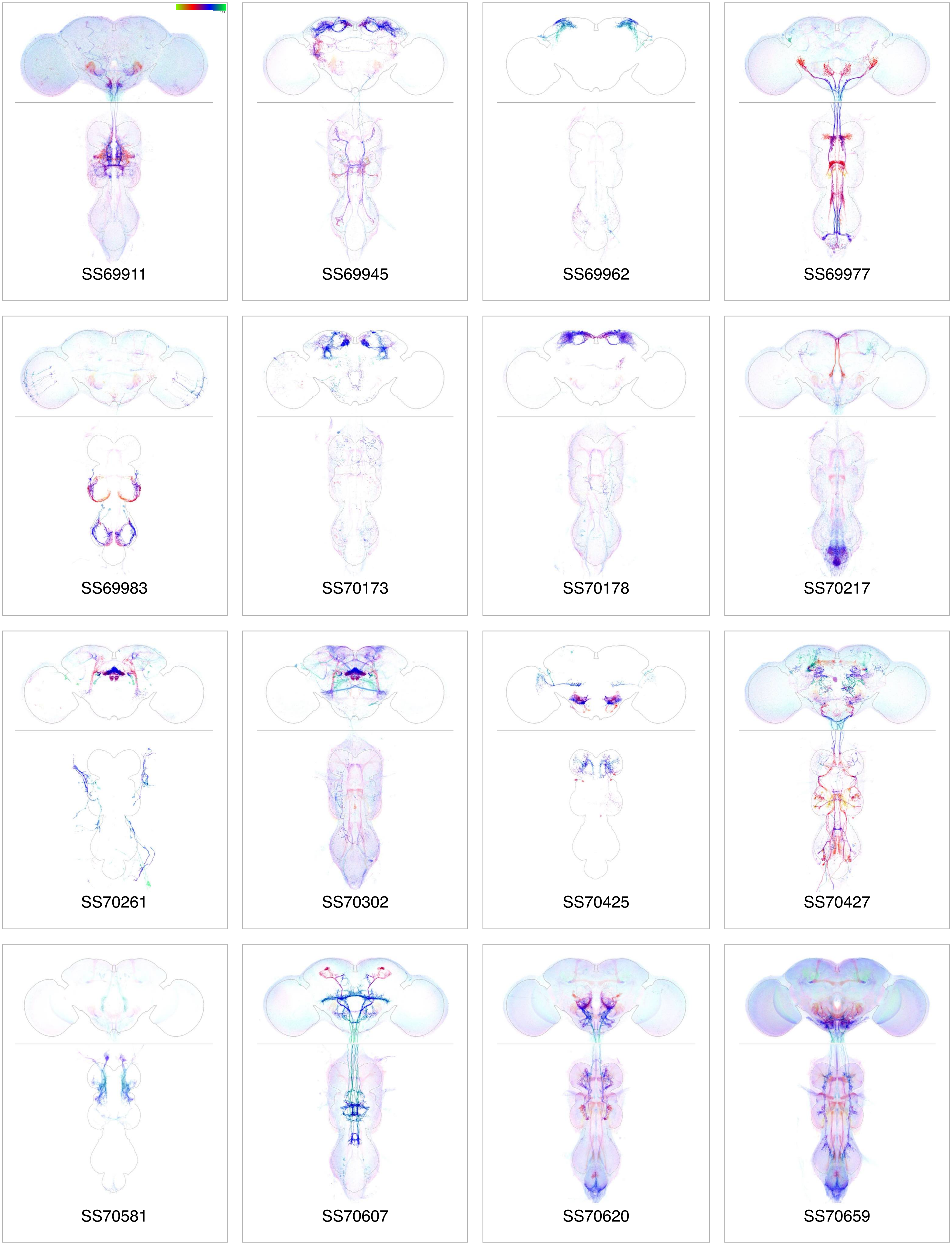
SS69911
SS69945
SS69962
SS69977
SS69983
SS70173
SS70178
SS70217
SS70261
SS70302
SS70425
SS70427
SS70581
SS70607
SS70620
SS70659

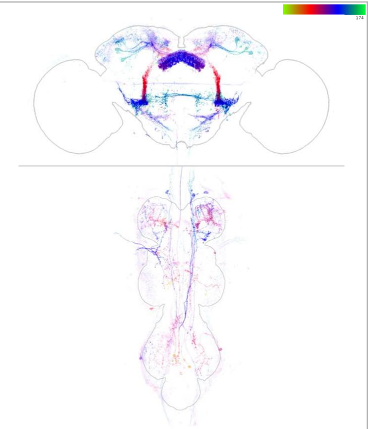
SS70711

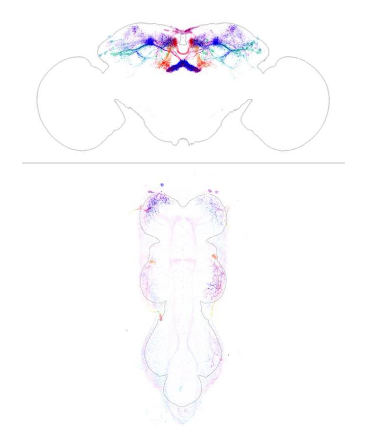
SS70902

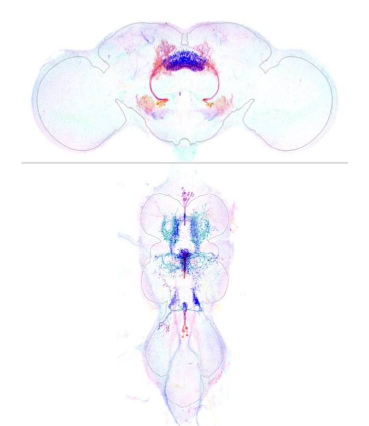
SS70921

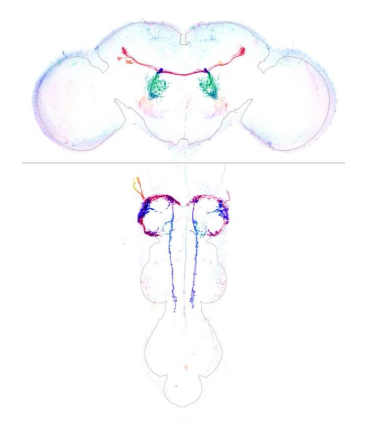
SS70962

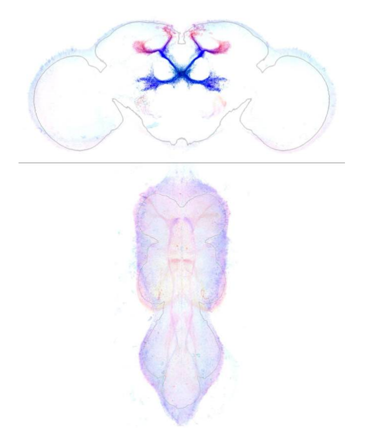
SS70971

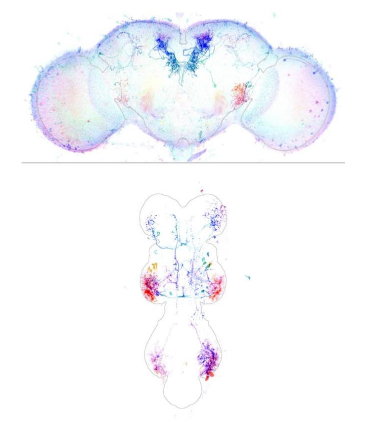
SS70977

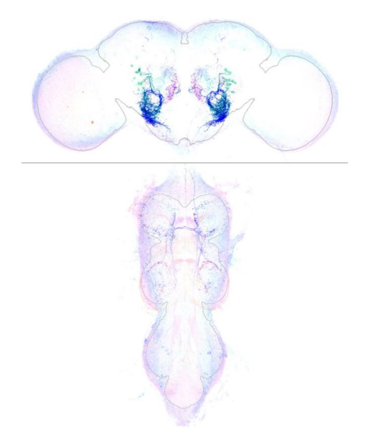
SS71277

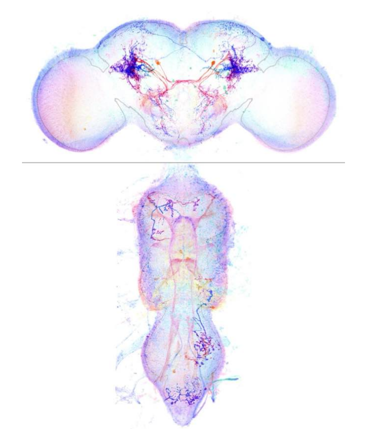
SS71285

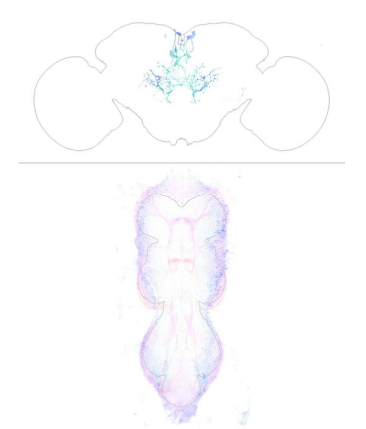
SS71290

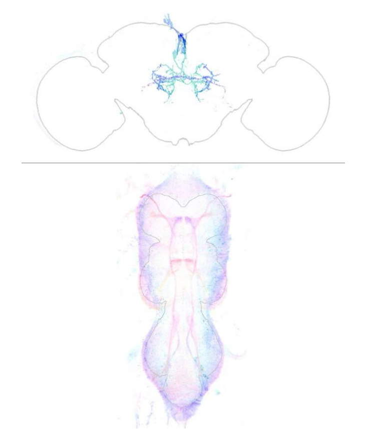
SS71299

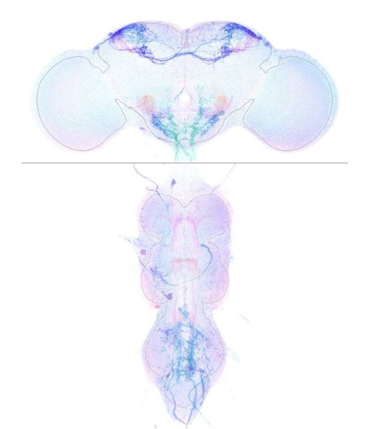
SS71326

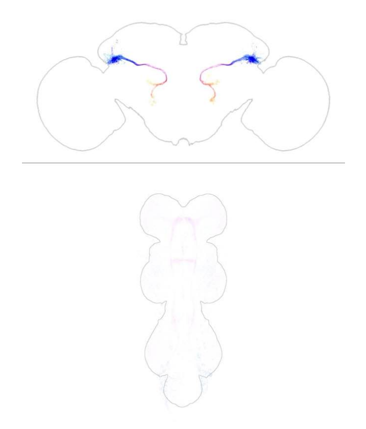
SS71340

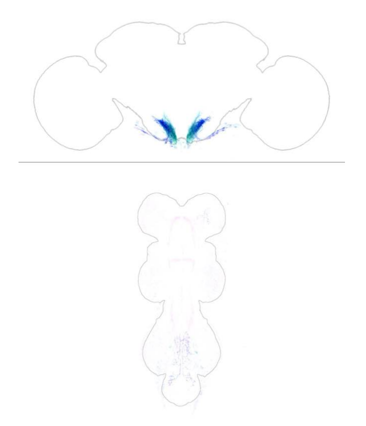
SS71348

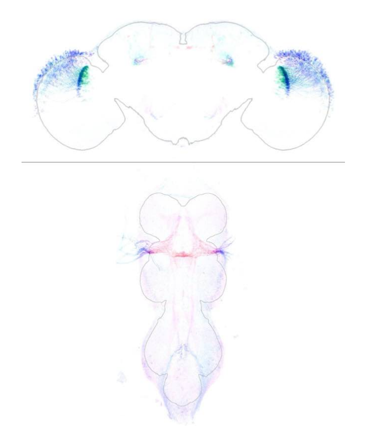
SS71373

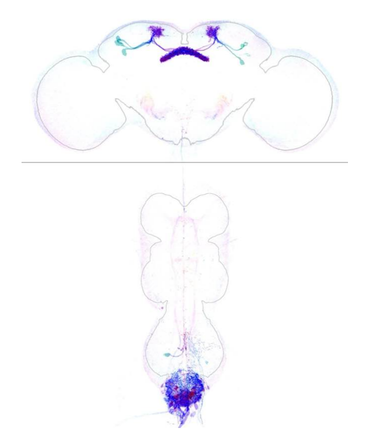
SS71433

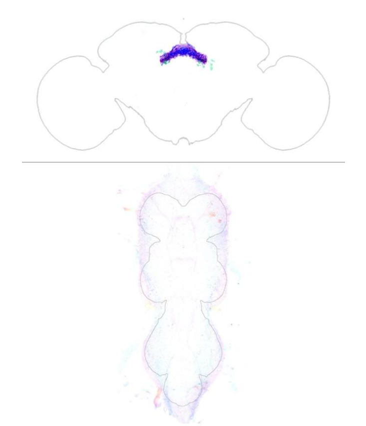
SS71436

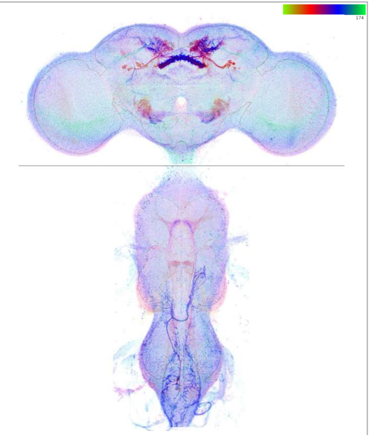

SS71790

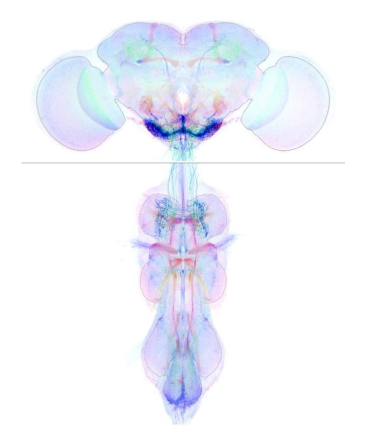

SS72084

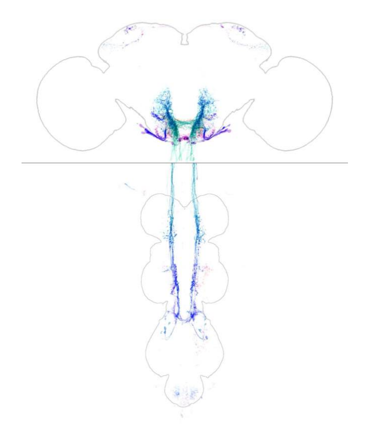

SS72104

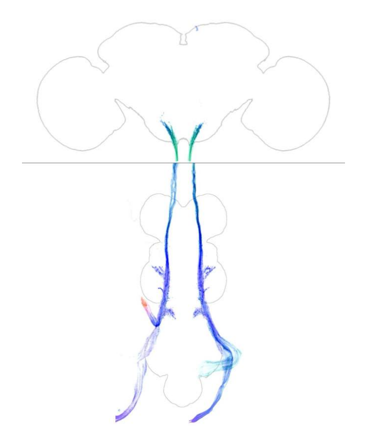

SS72201

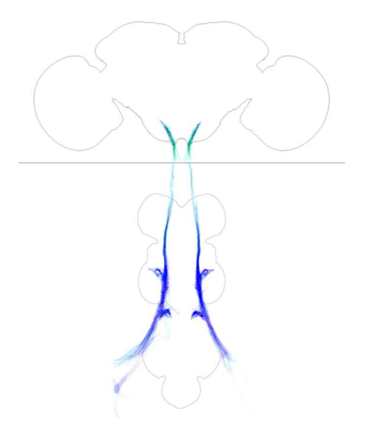

SS72203

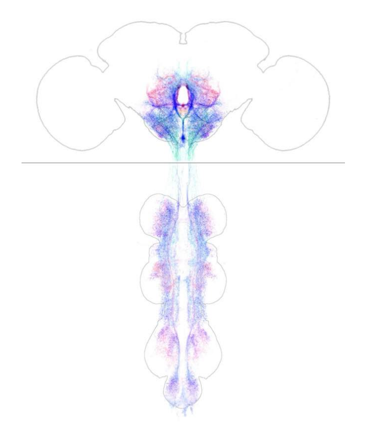

SS72207

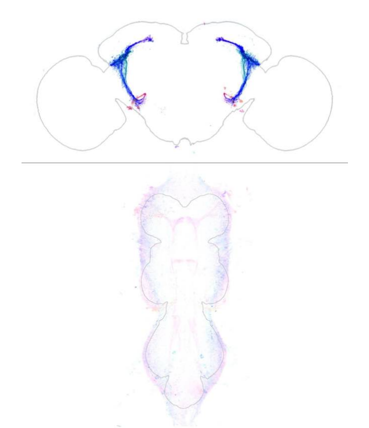

SS72272

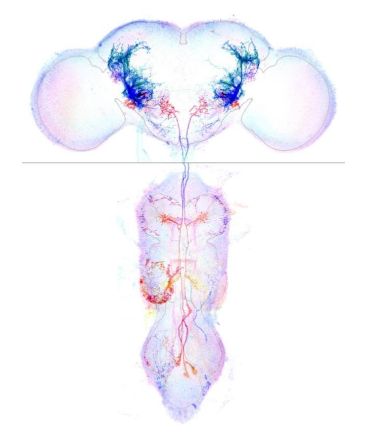

SS72288

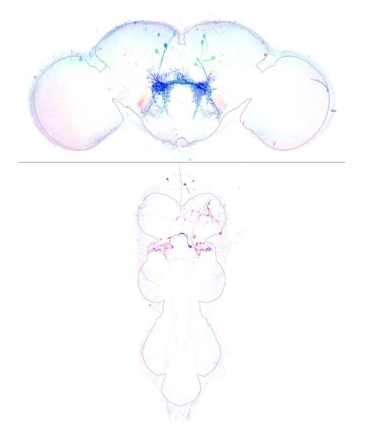

SS72289

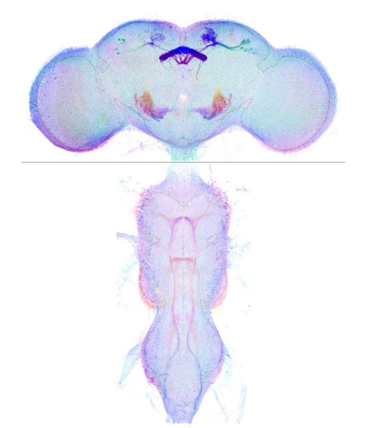

SS72415

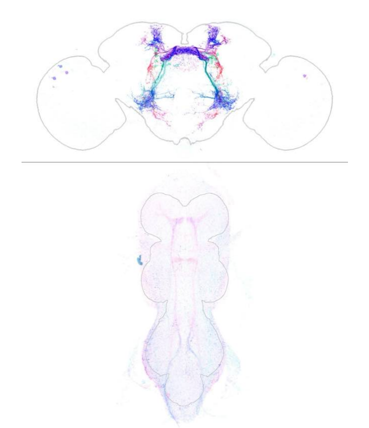

SS72731

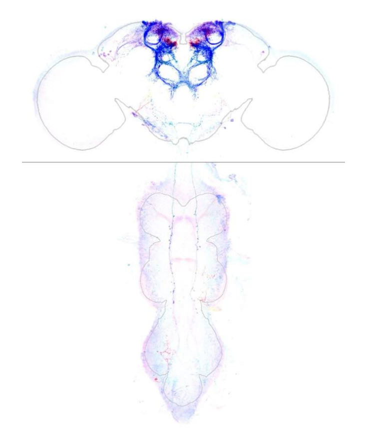

SS72874

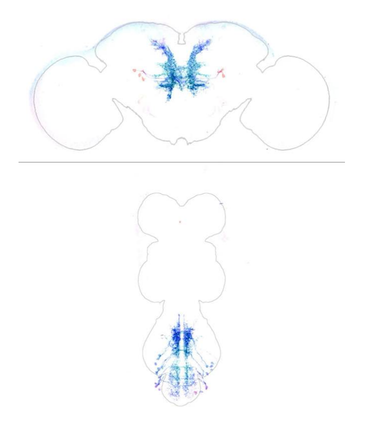

SS72883

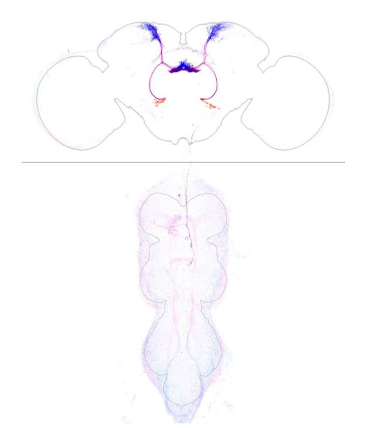

SS72913

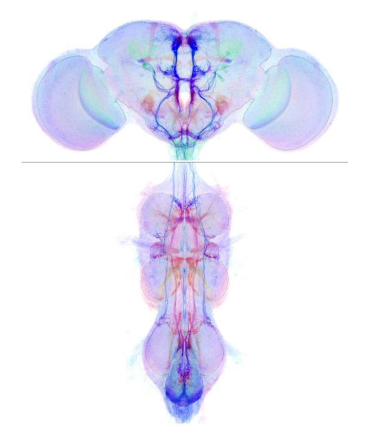

SS72989

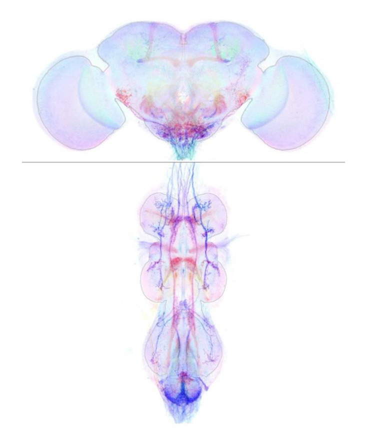

SS73035

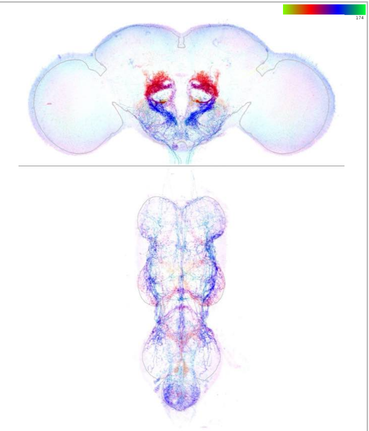

SS73369

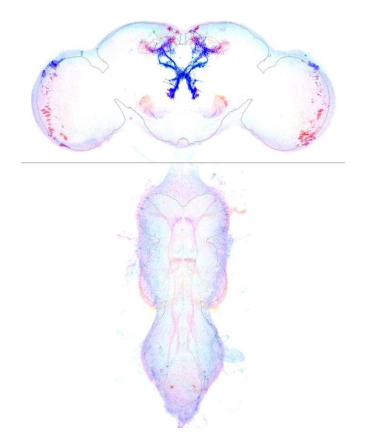

SS73377

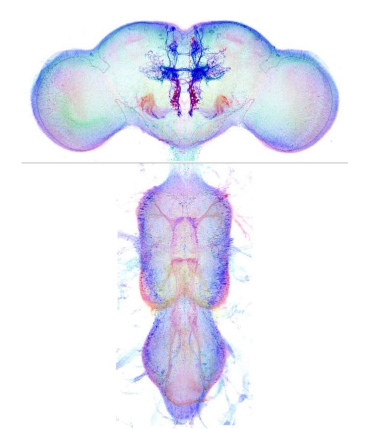

SS73384

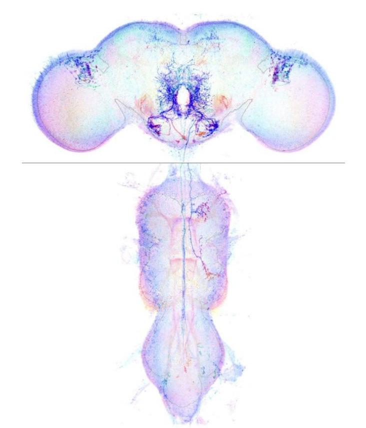

SS73388

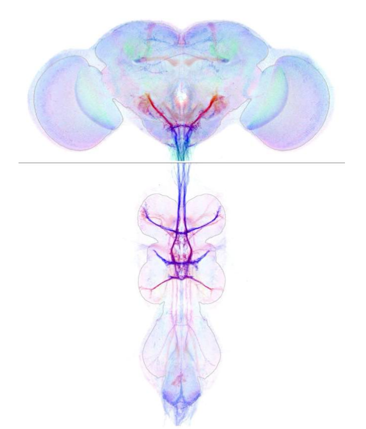

SS73430

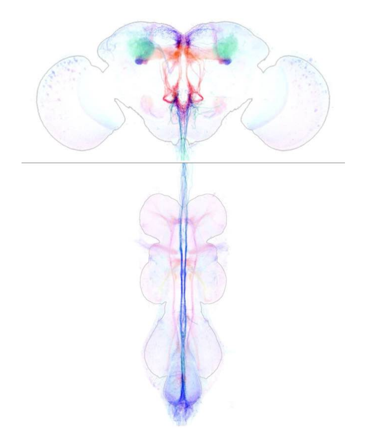

SS73441

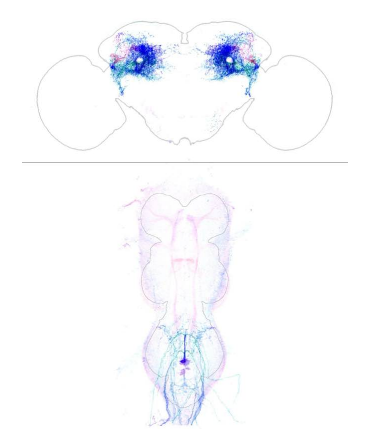

SS73495

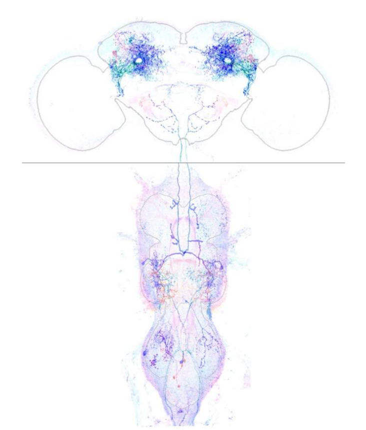

SS73509

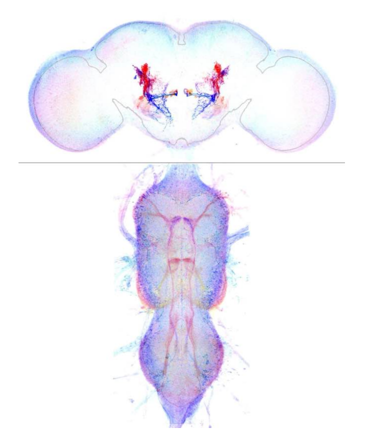

SS73654

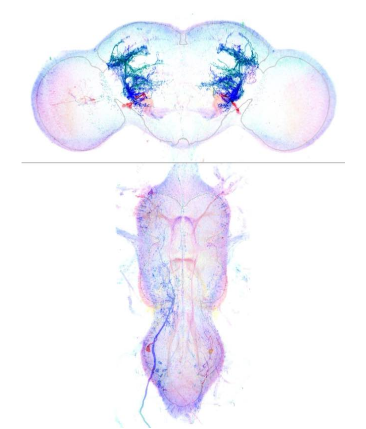

SS73660

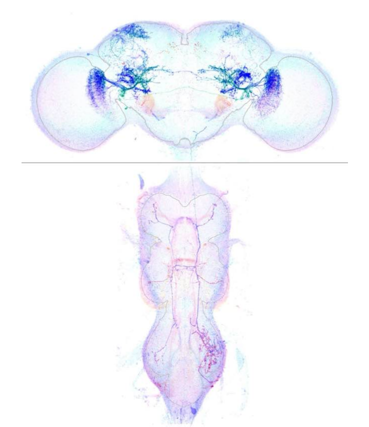

SS74070

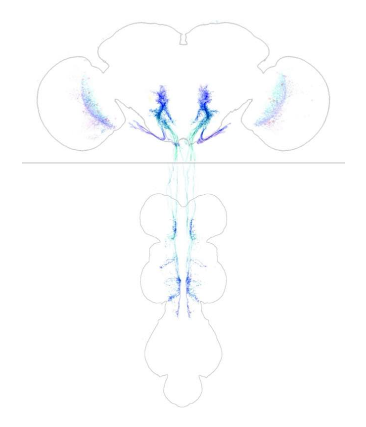

SS74390

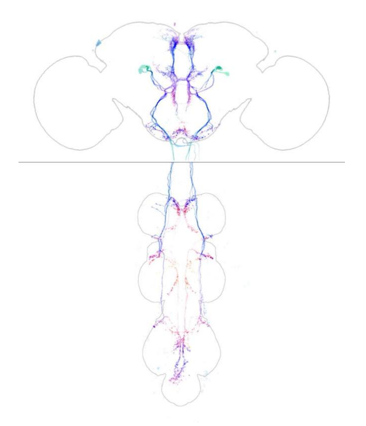

SS74396

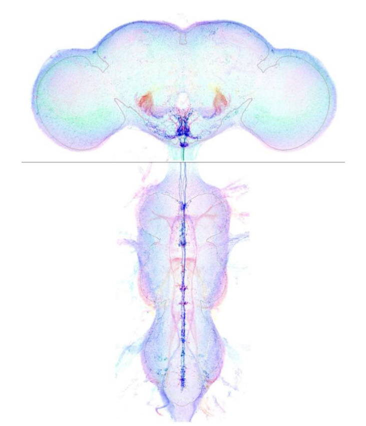

SS74442

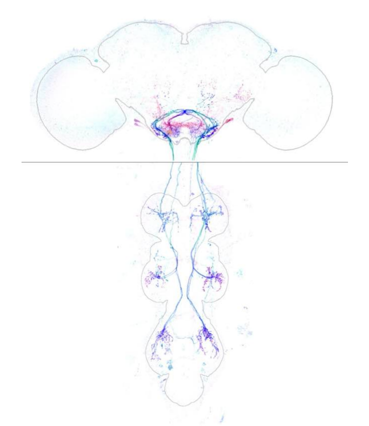

SS74458

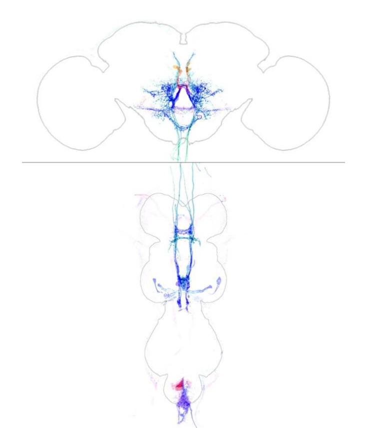

SS74475

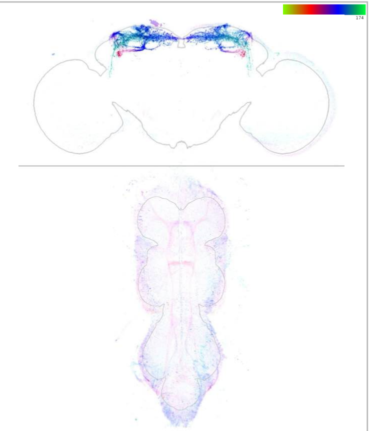
SS74572

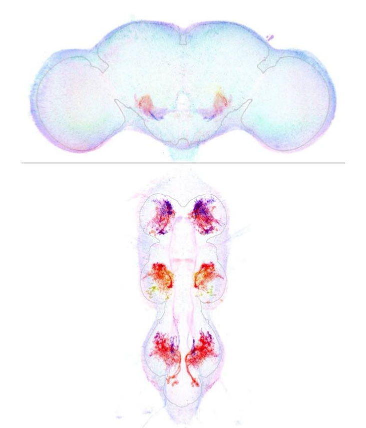
SS74639

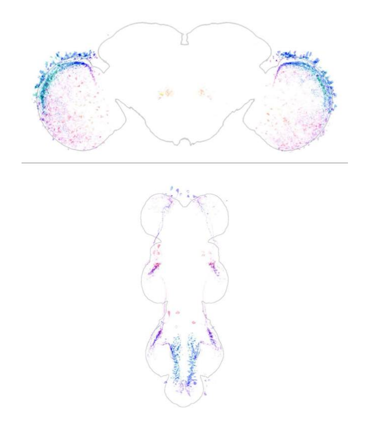
SS74667

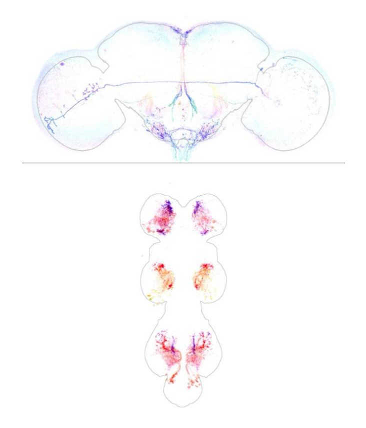
SS74713

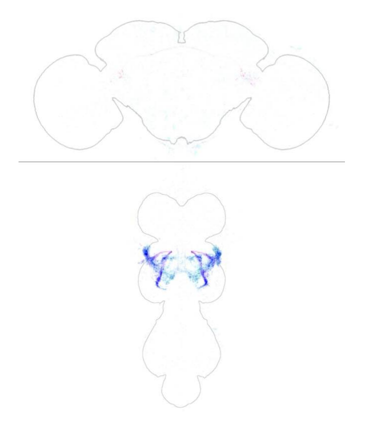
SS74735

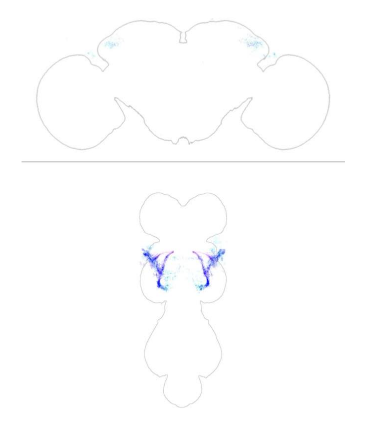
SS74741

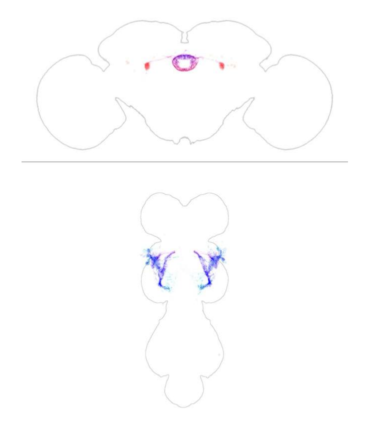
SS74757

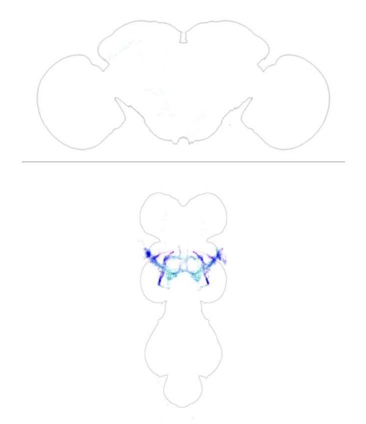
SS74760

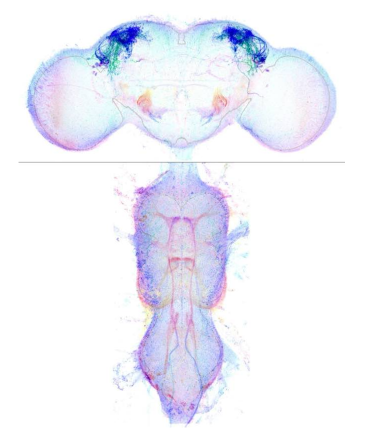
SS74831

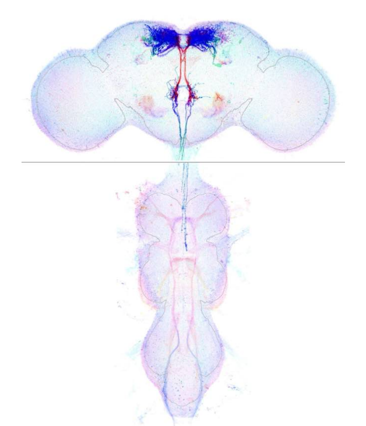
SS74884

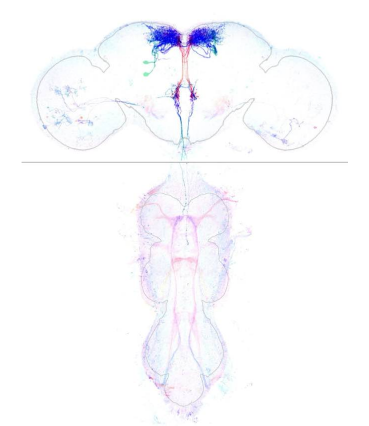
SS74917

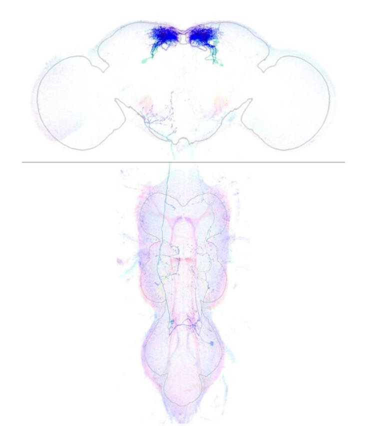
SS74918

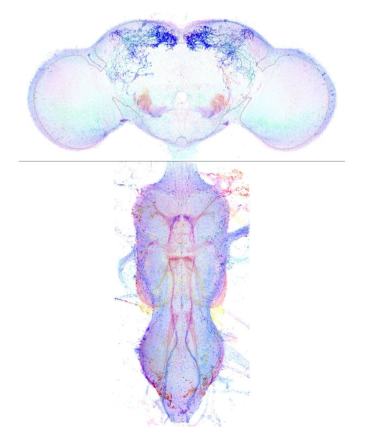
SS74926

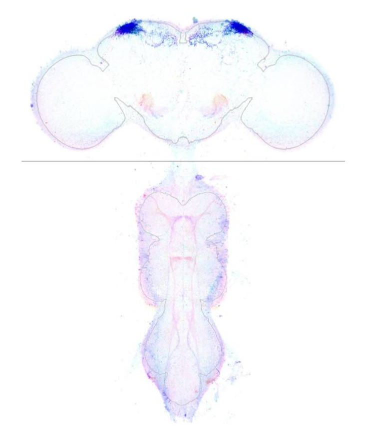
SS74930

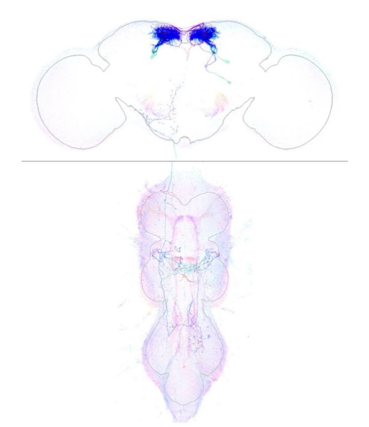
SS74944

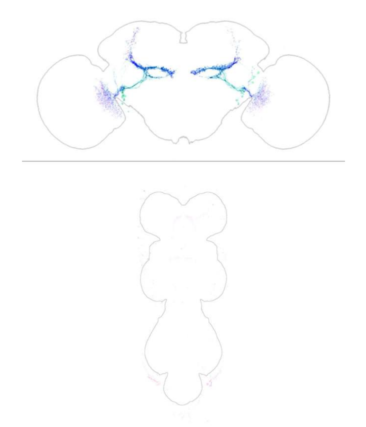
SS75046

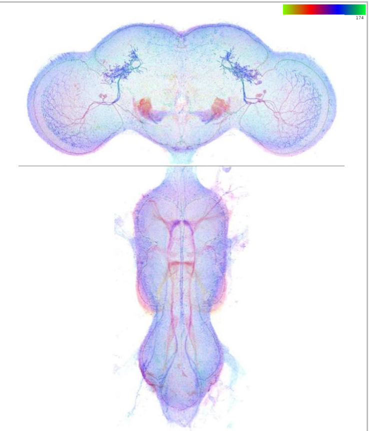
SS75074

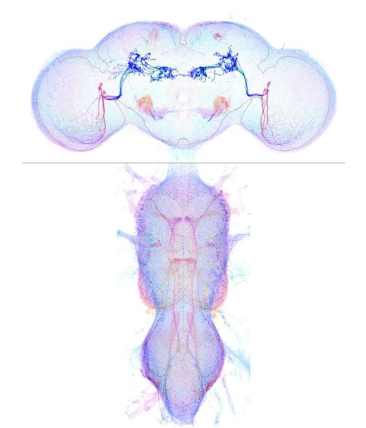
SS75092

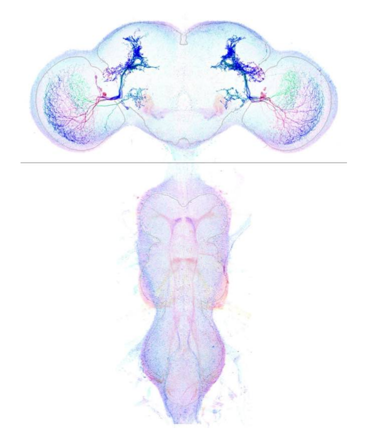
SS75146

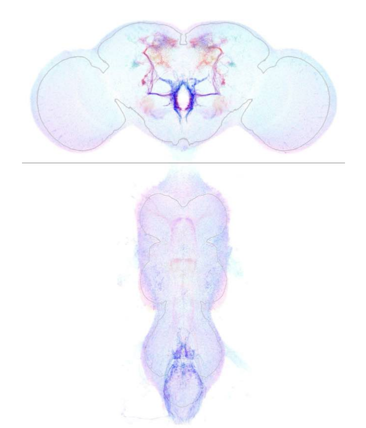
SS75199

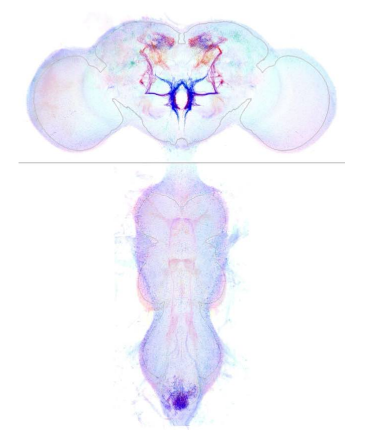
SS75200

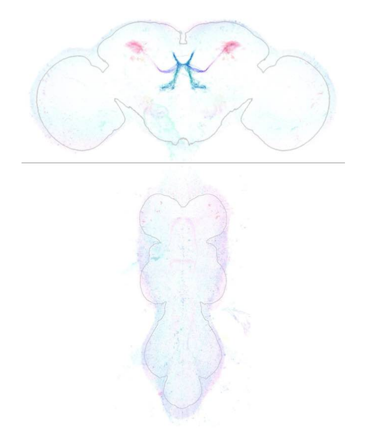
SS75253

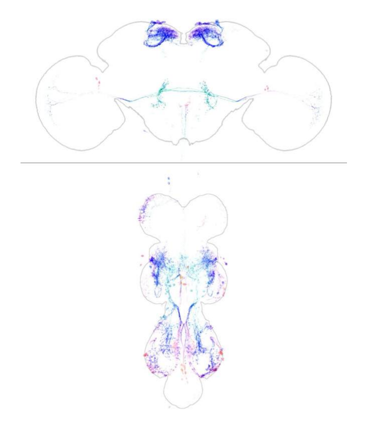
SS75439

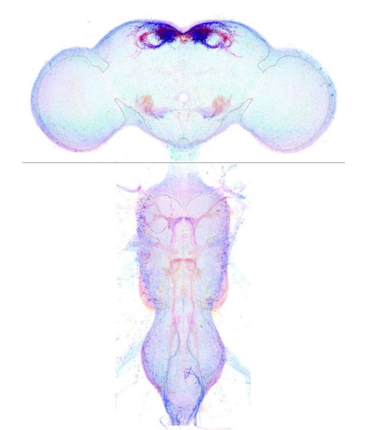
SS75456

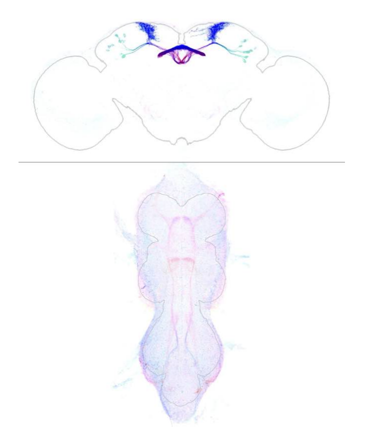
SS75512

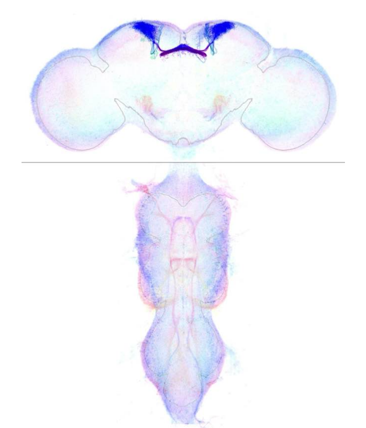
SS75530

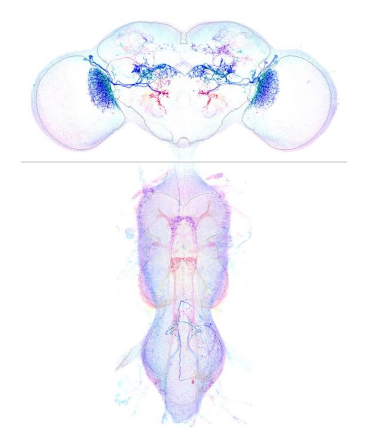
SS75546

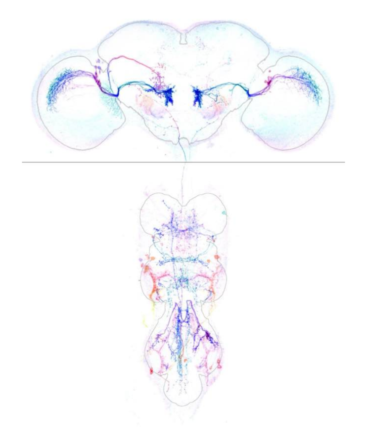
SS75571

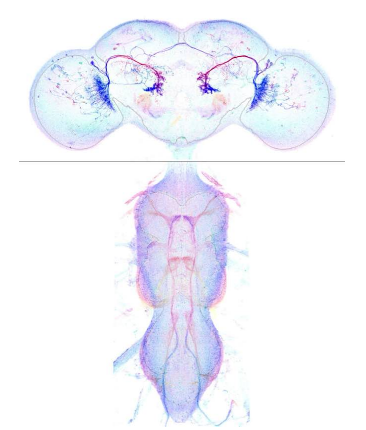
SS75634

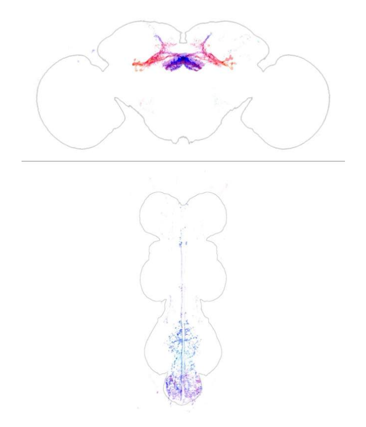
SS75731

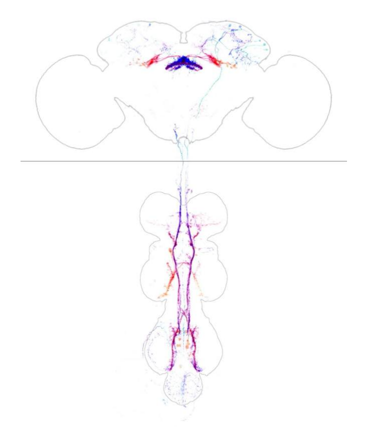
SS75735

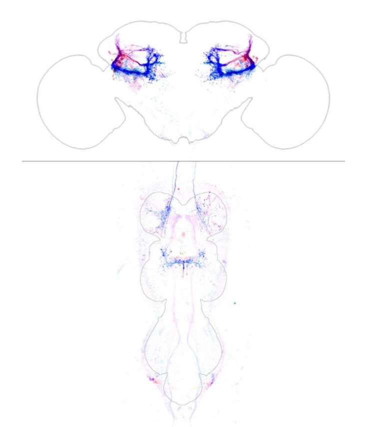
SS75751

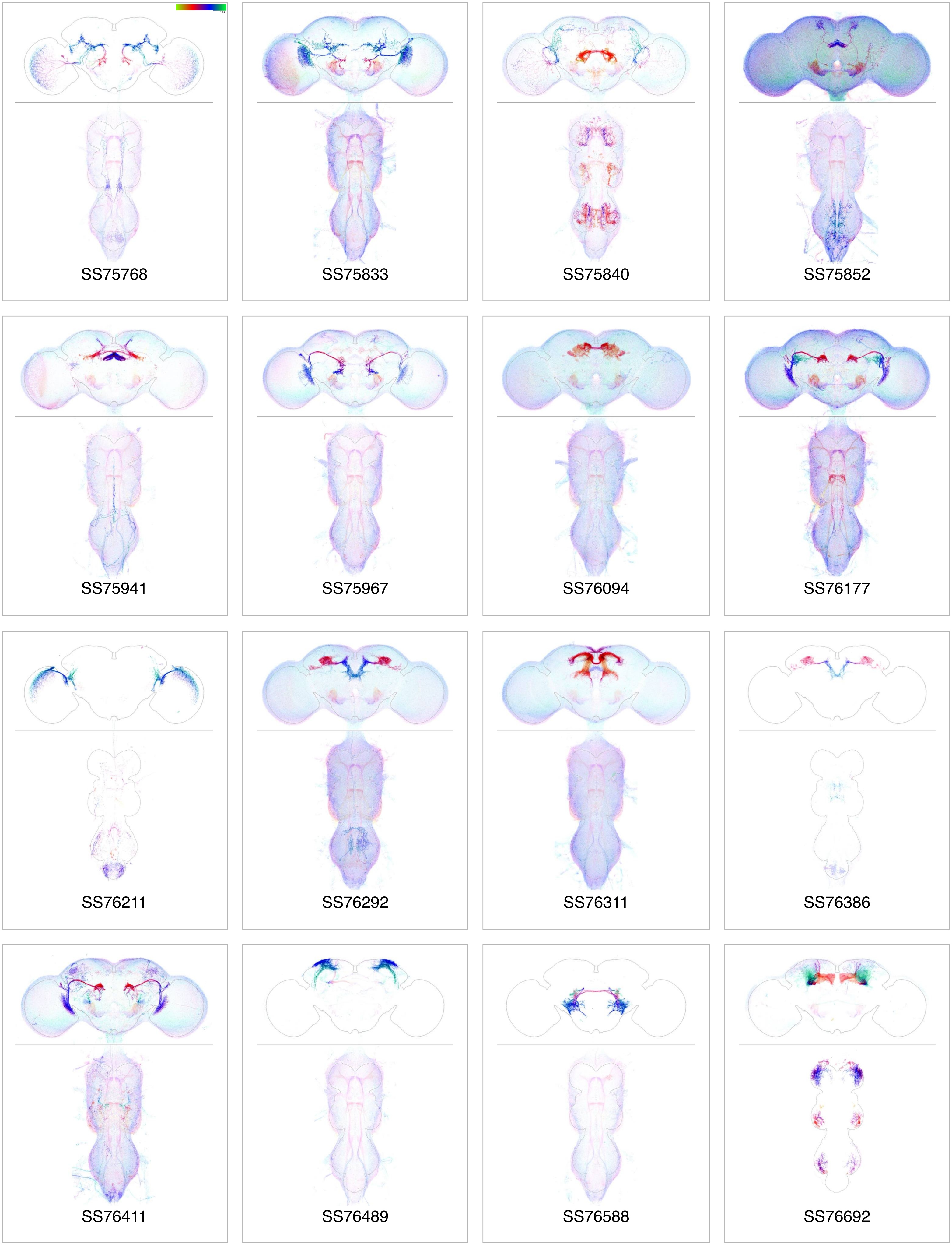
SS75768
SS75833
SS75840
SS75852
SS75941
SS75967
SS76094
SS76177
SS76211
SS76292
SS76311
SS76386
SS76411
SS76489
SS76588
SS76692

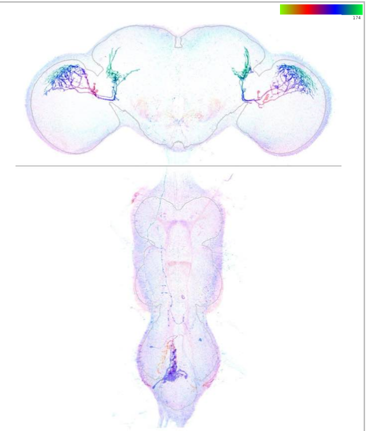
SS76835

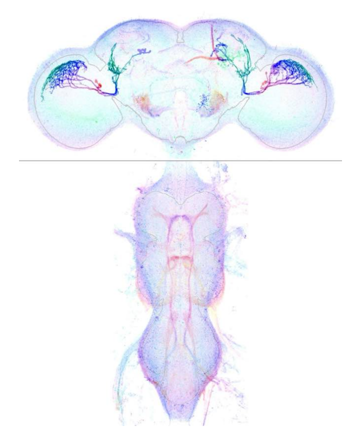
SS76839

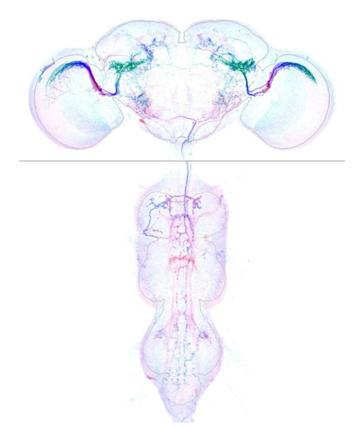
SS76873

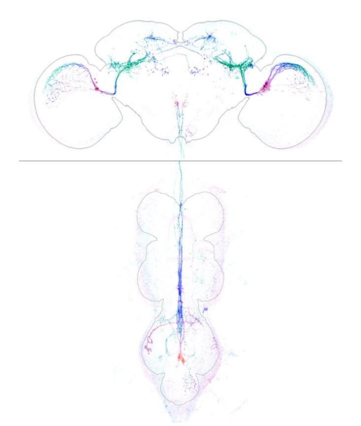
SS76874

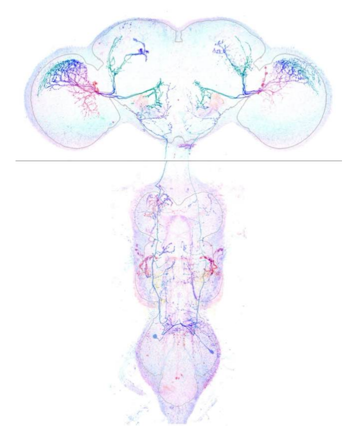
SS76877

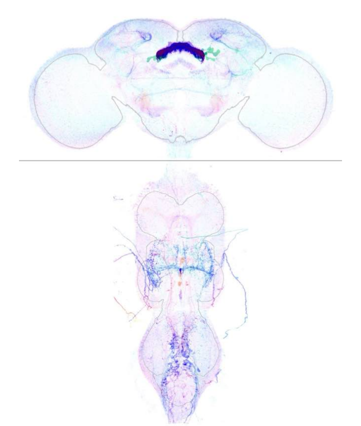
SS77099

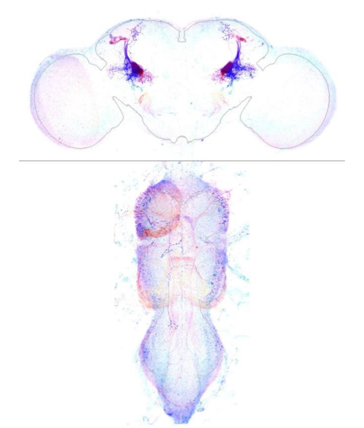
SS77132

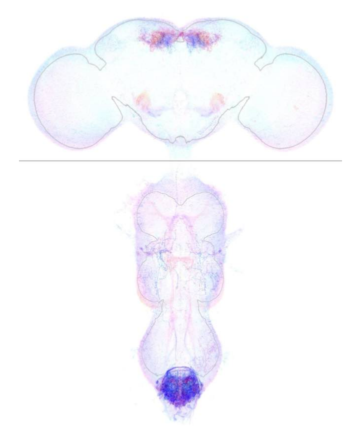
SS77191

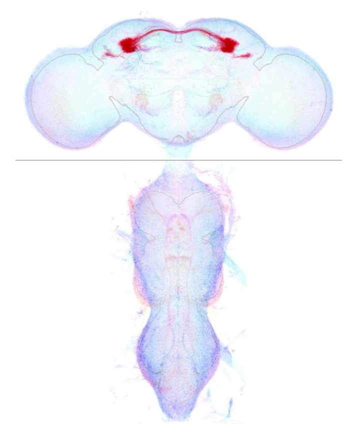
SS77402

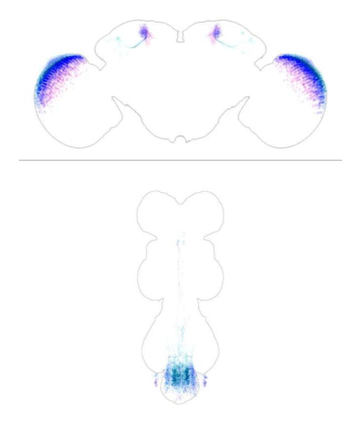
SS77424

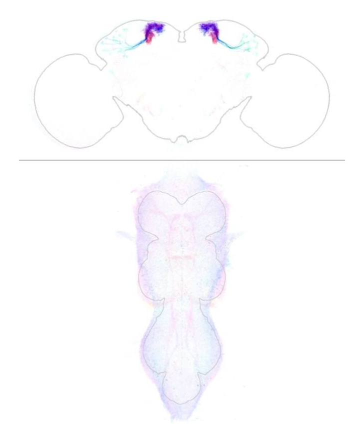
SS77442

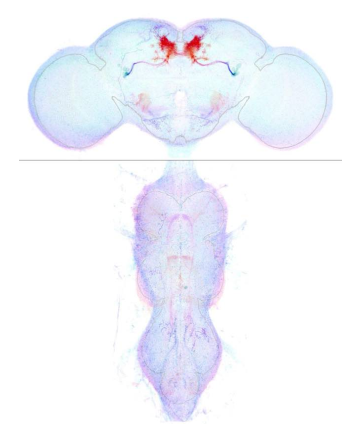
SS77450

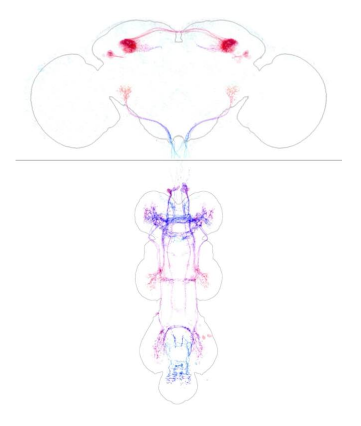
SS77462

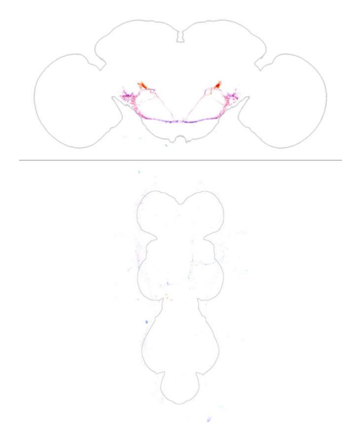
SS77498

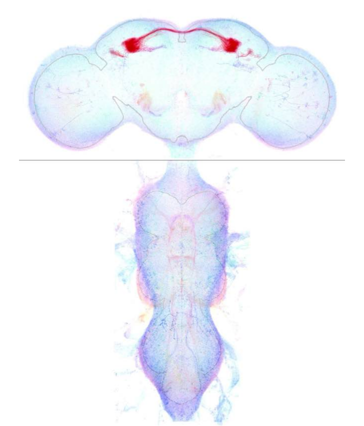
SS77545

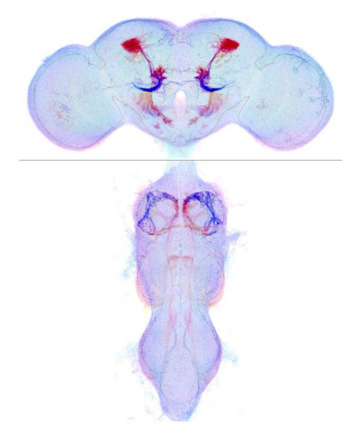
SS77569

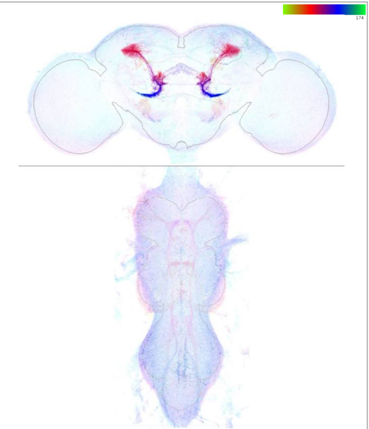
SS77582
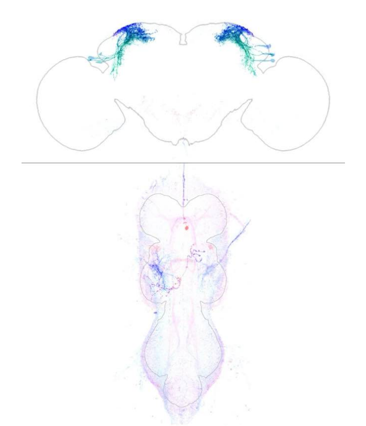
SS77940
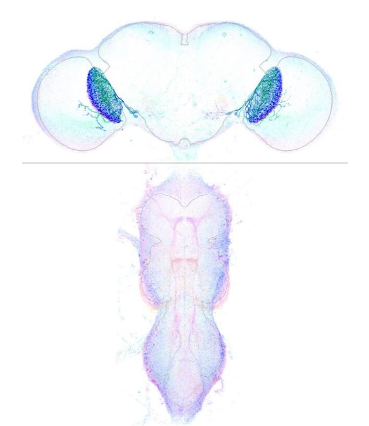
SS77942
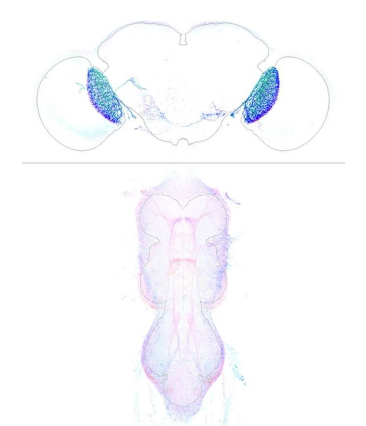
SS77964
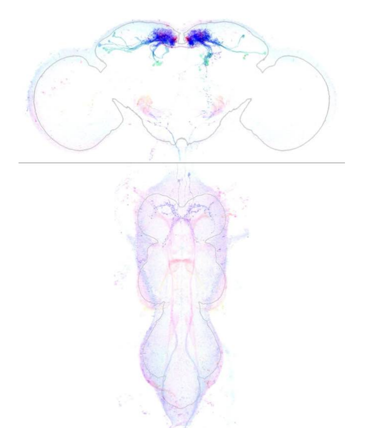
SS77971
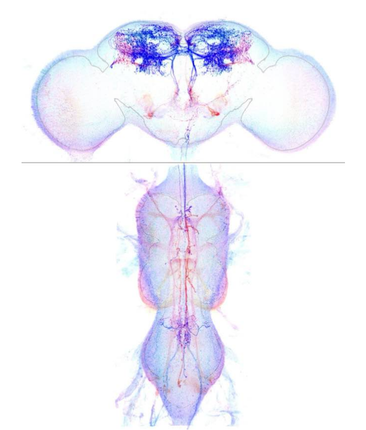
SS78219
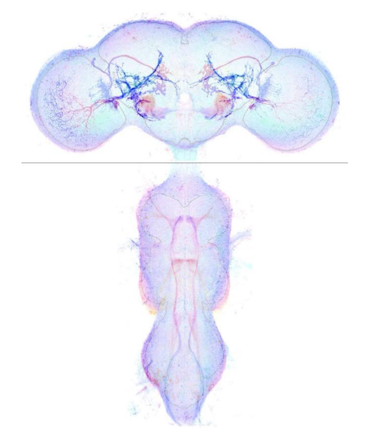
SS78239
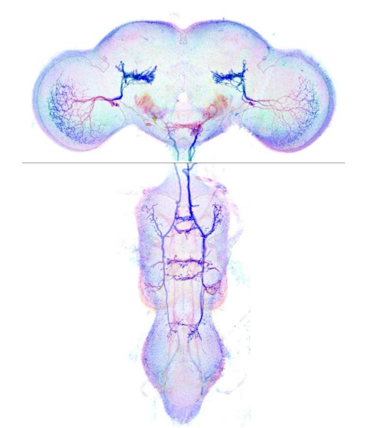
SS78248
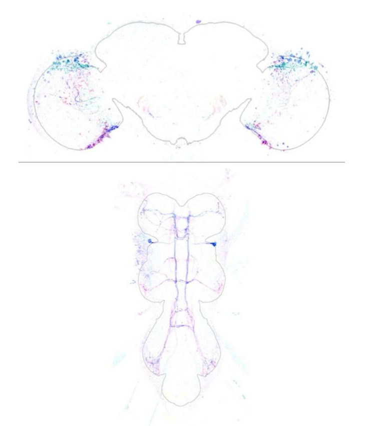
SS78380
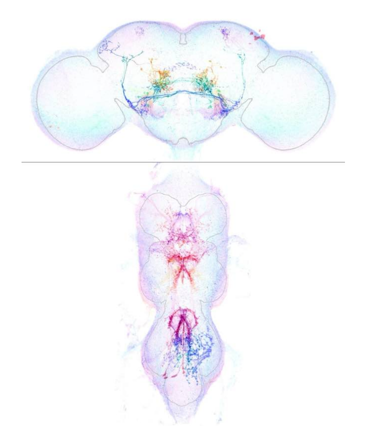
SS78420
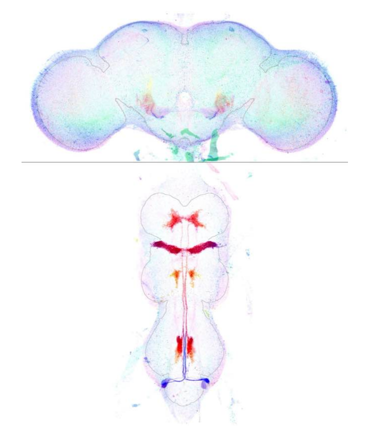
SS78430
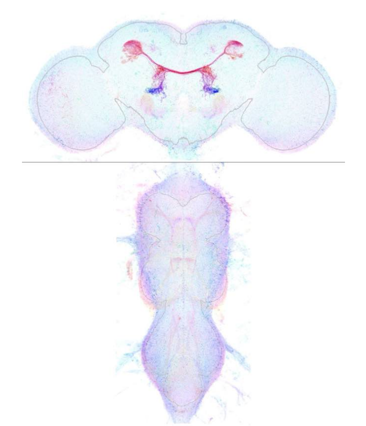
SS78469
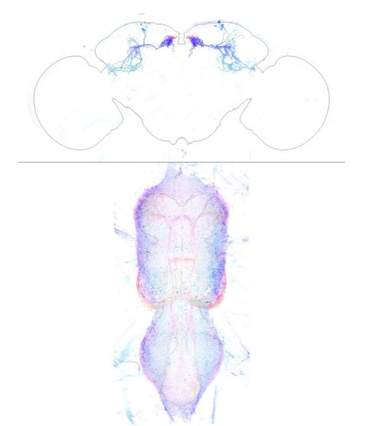
SS78556
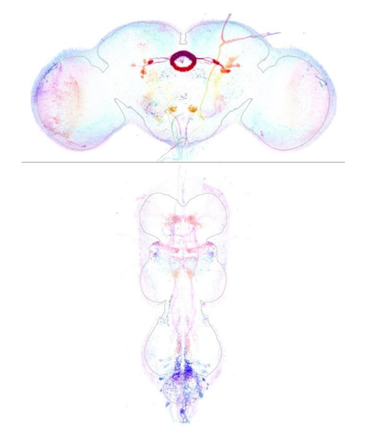
SS78687
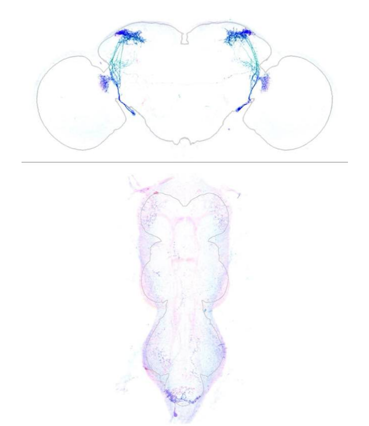
SS78841
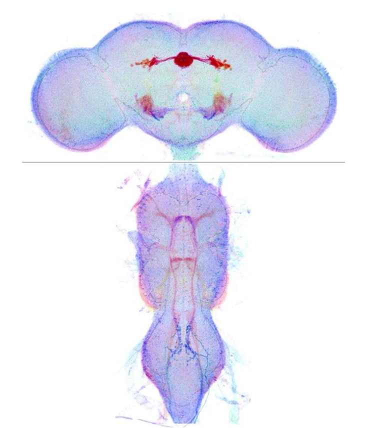
SS79053

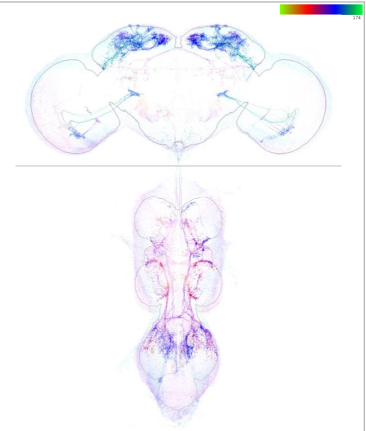
SS79089

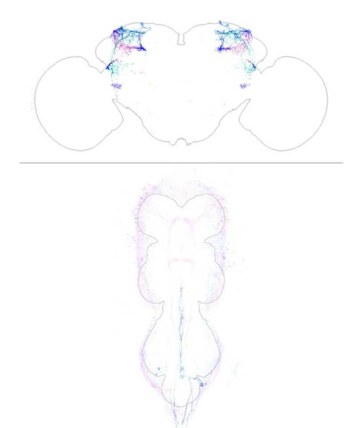
SS79124

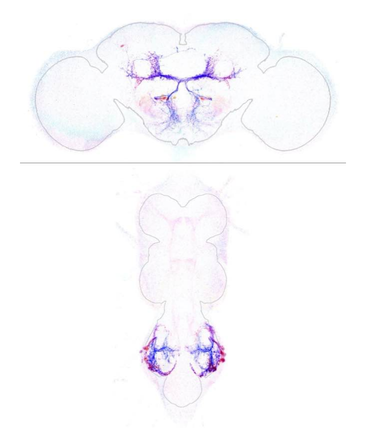
SS79251

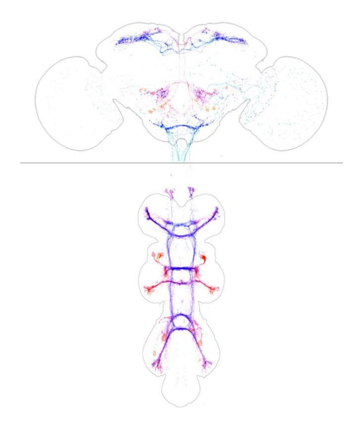
SS79279

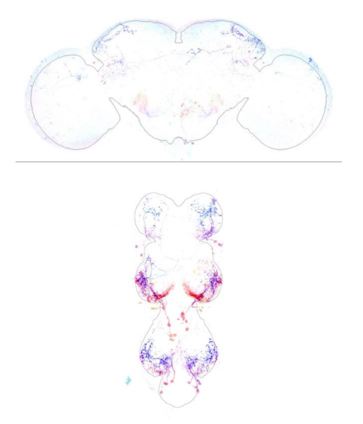
SS79321

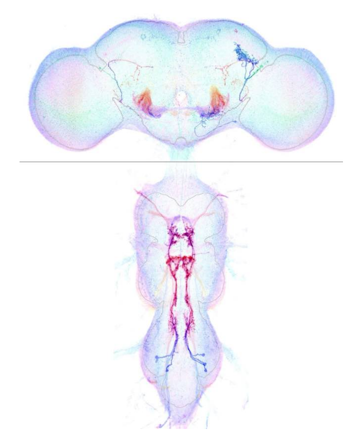
SS79361

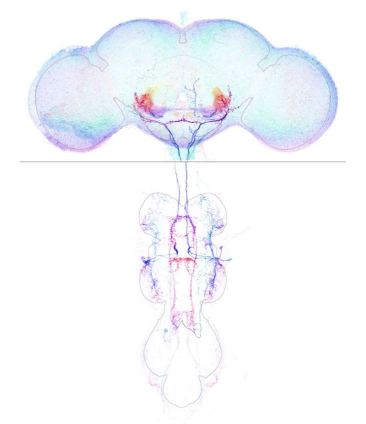
SS79392

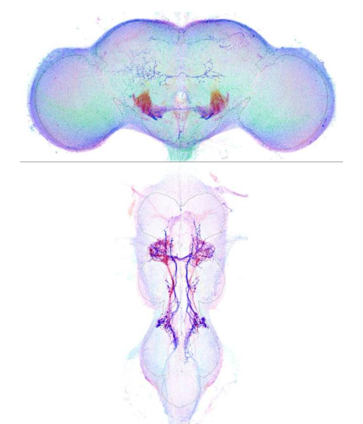
SS79449

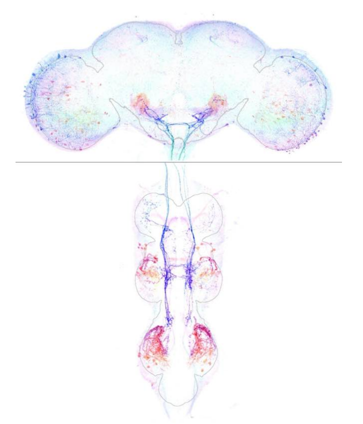
SS79454

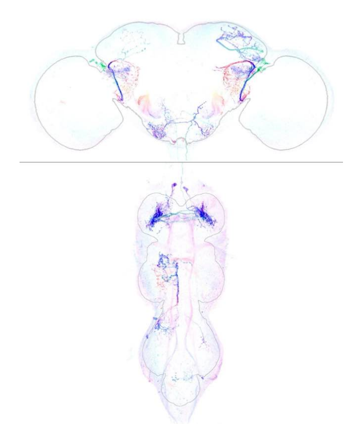
SS79461

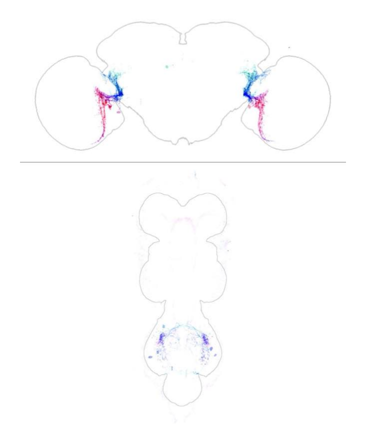
SS79481

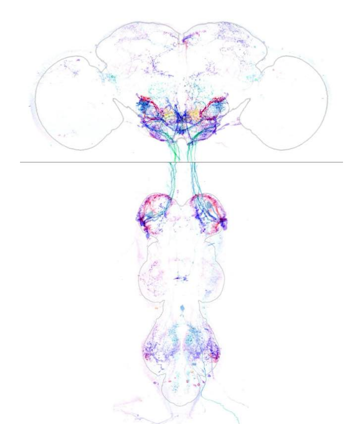
SS79515

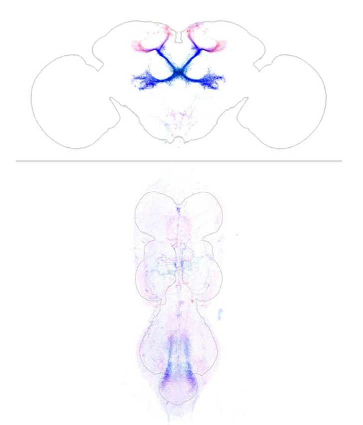
SS79521

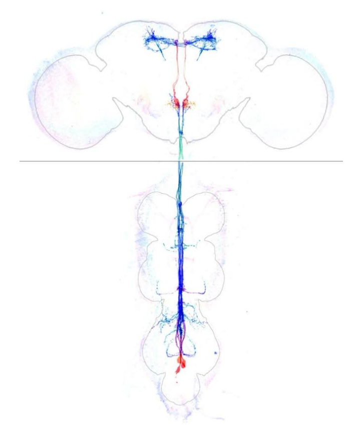
SS79551

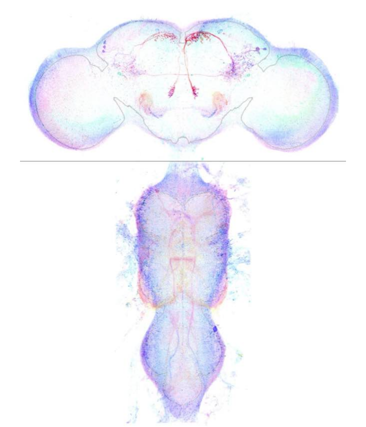
SS79598

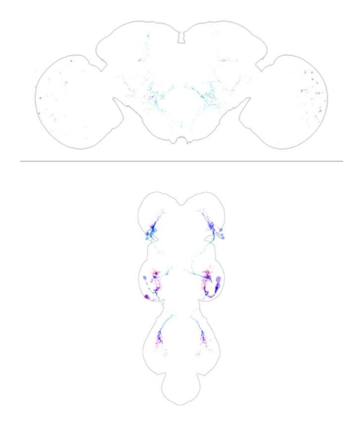
SS80021

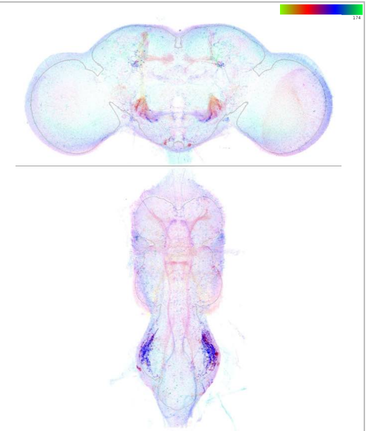

SS80088

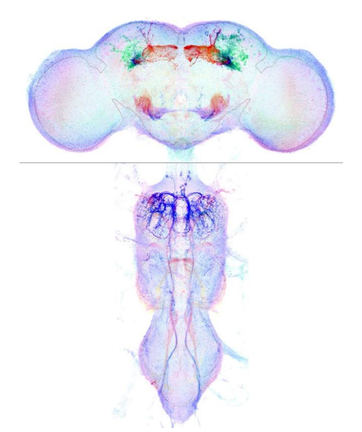

SS80089

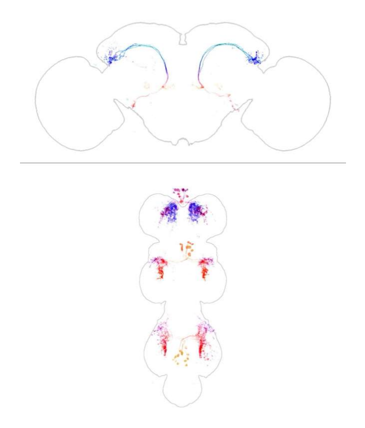

SS80093

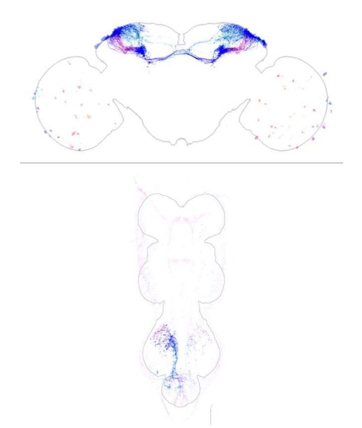

SS80180

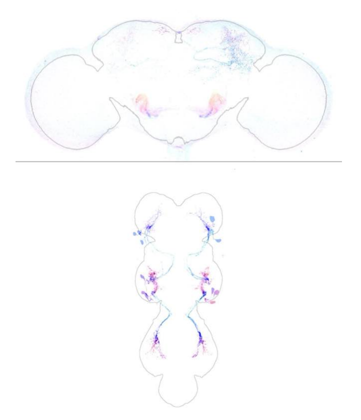

SS80251

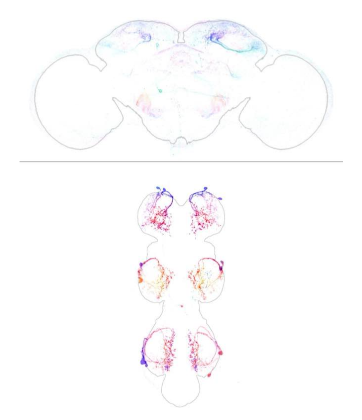

SS80260

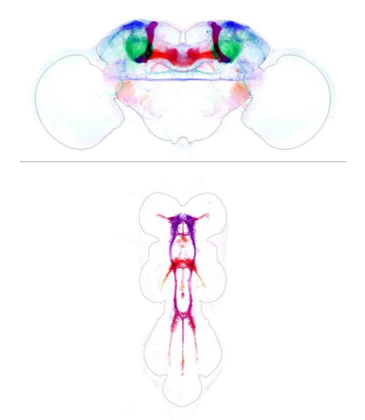

SS80292

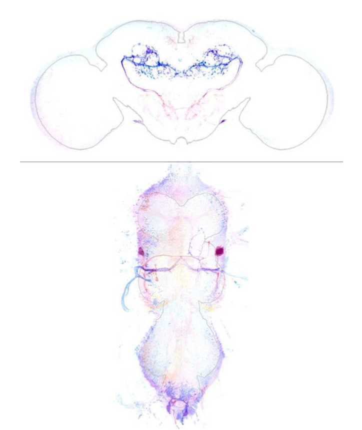

SS80314

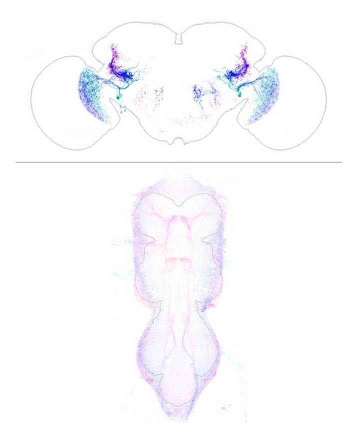

SS80326

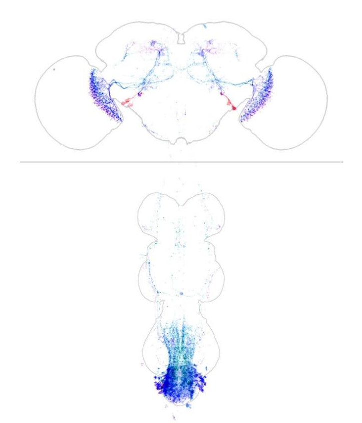

SS80350

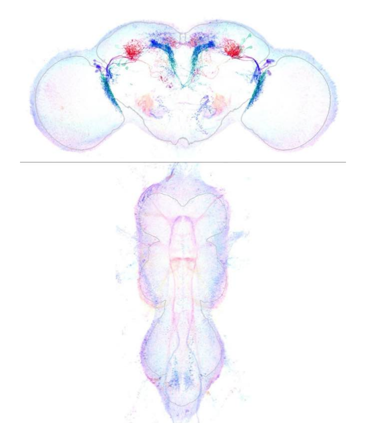

SS80374

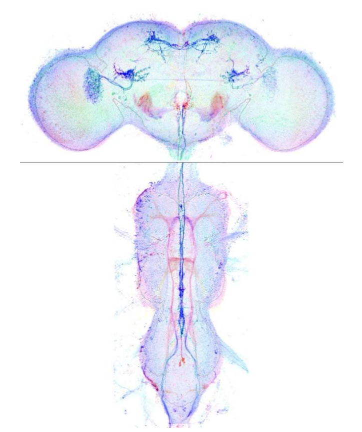

SS80378

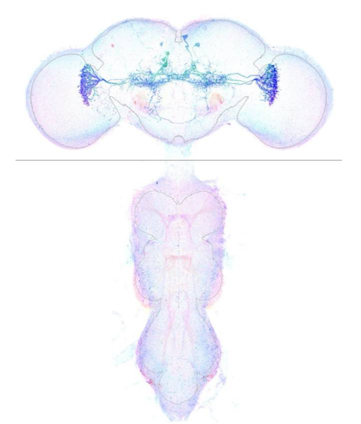

SS80435

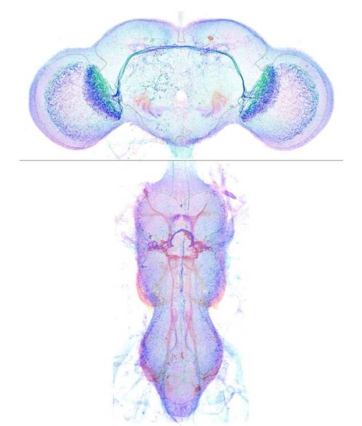

SS80443

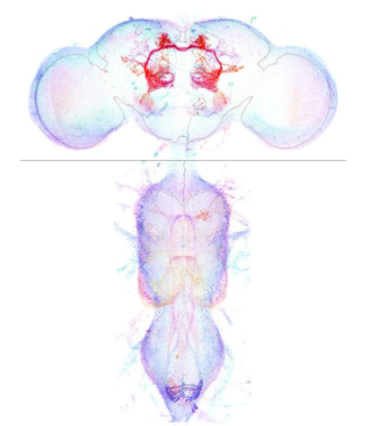

SS80756

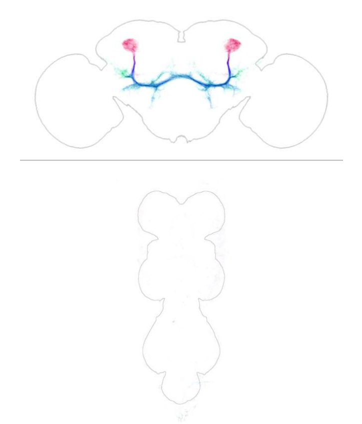

SS80761

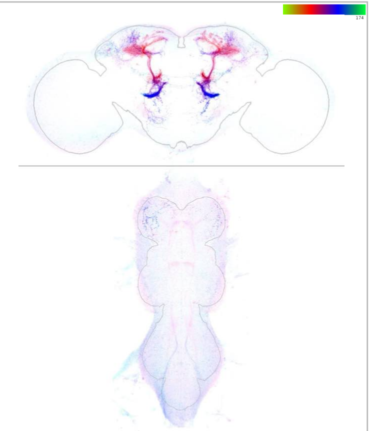
SS80822

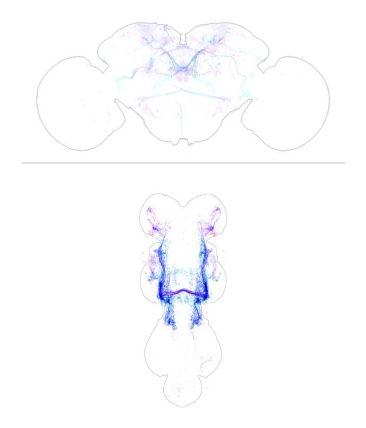
SS80845

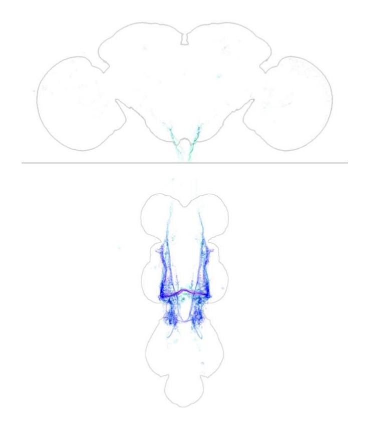
SS80846

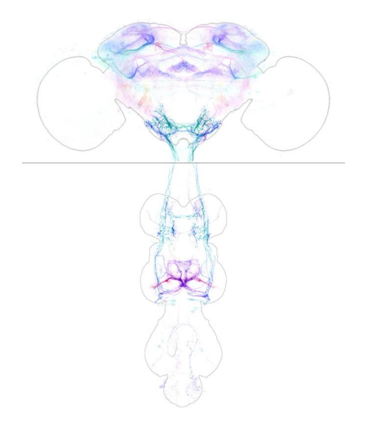
SS80847

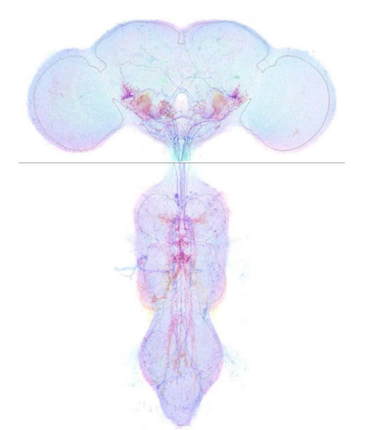
SS80854

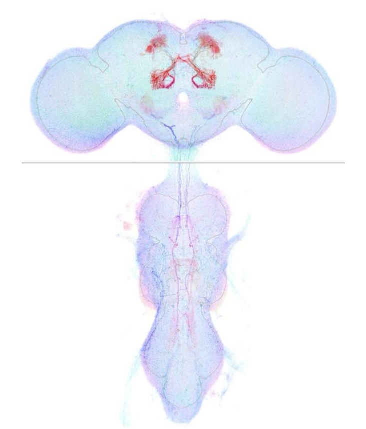
SS80896

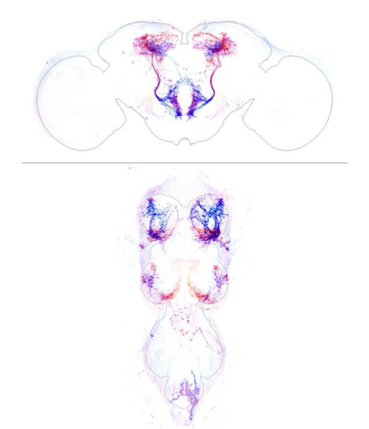
SS80940

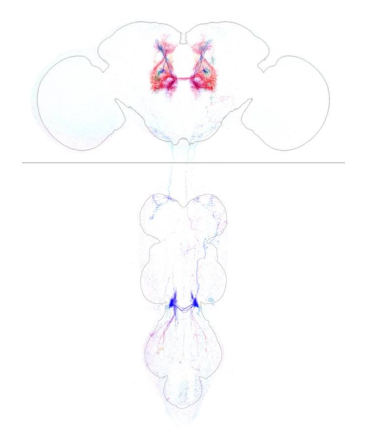
SS80958

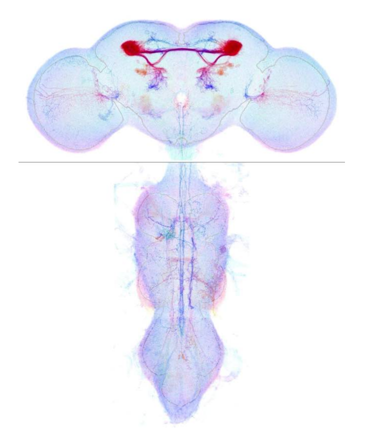
SS80967

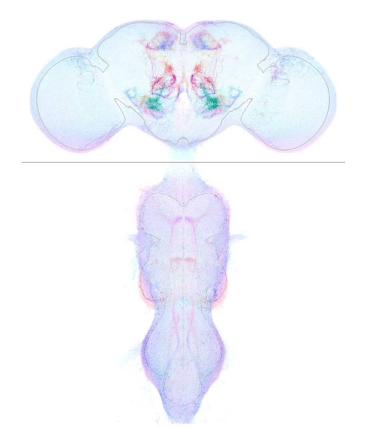
SS80974

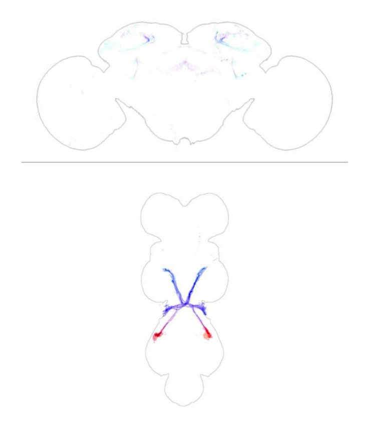
SS80982

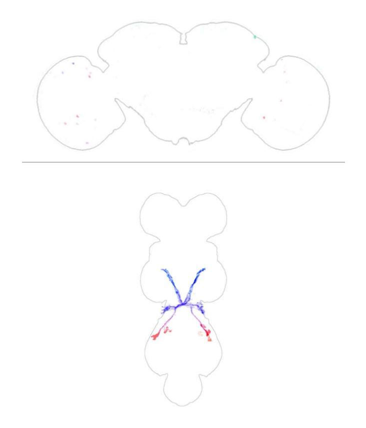
SS80984

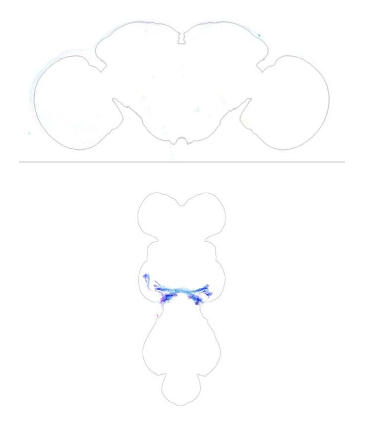
SS80991

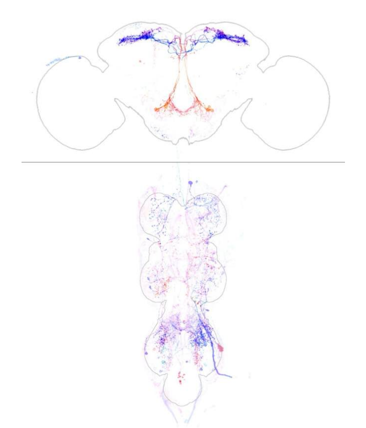
SS81024

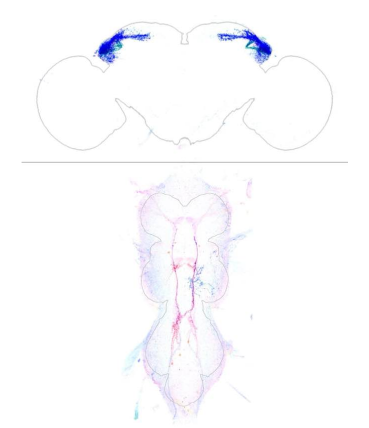
SS81027

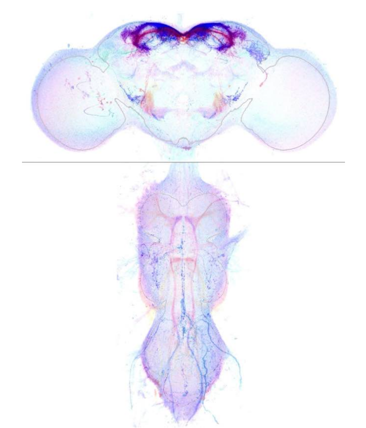
SS81054

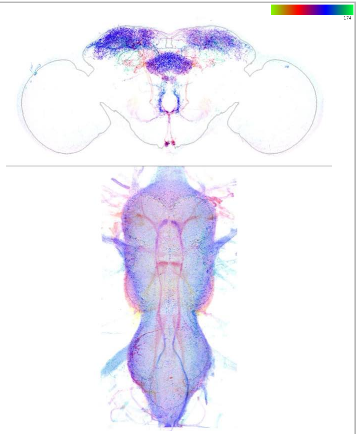
SS81068

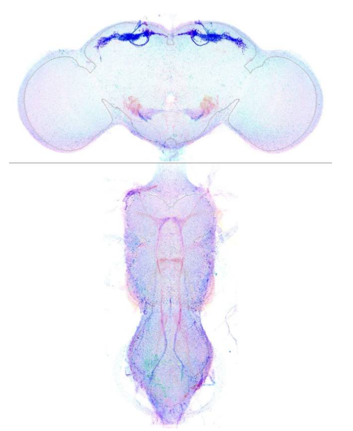
SS81072

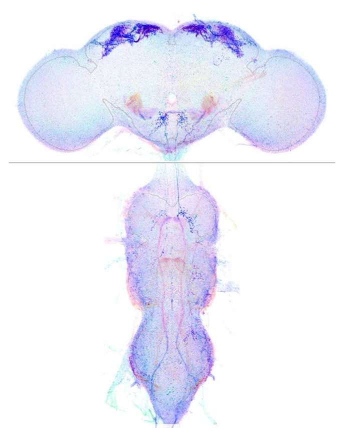
SS81122

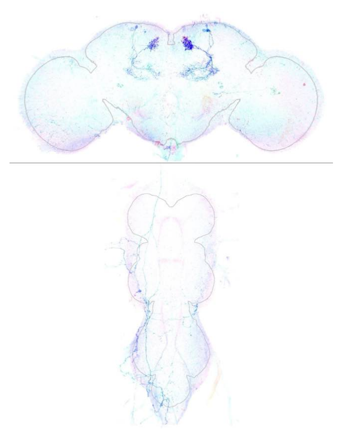
SS81138

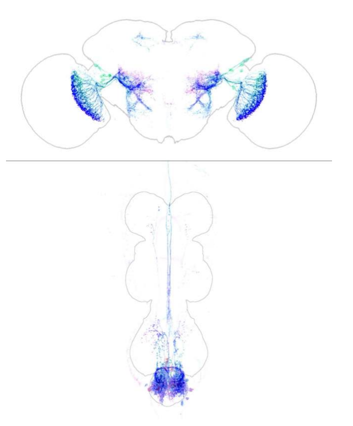
SS81174

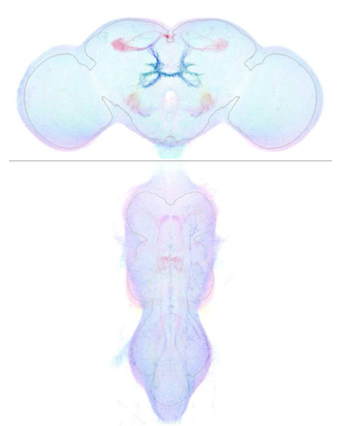
SS81305

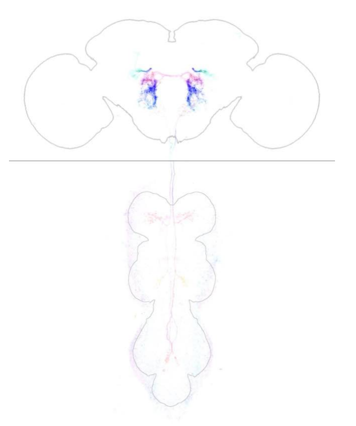
SS81341

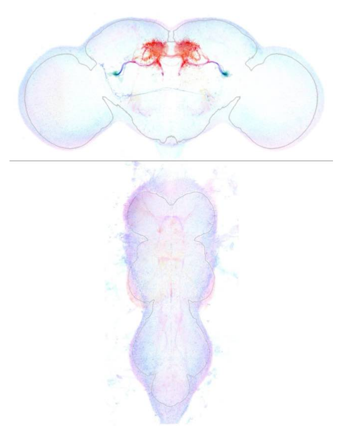
SS81353

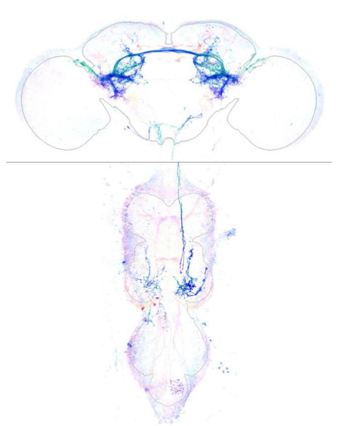
SS81408

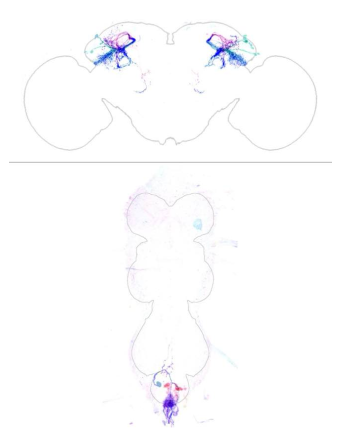
SS81475

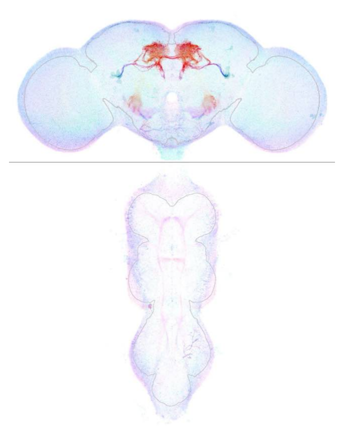
SS81521

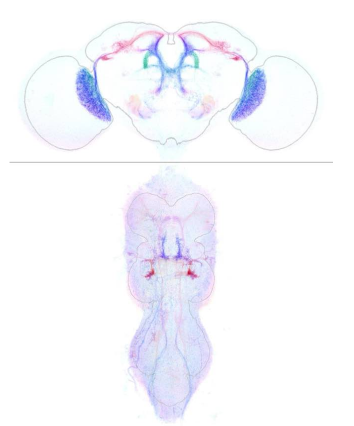
SS81529

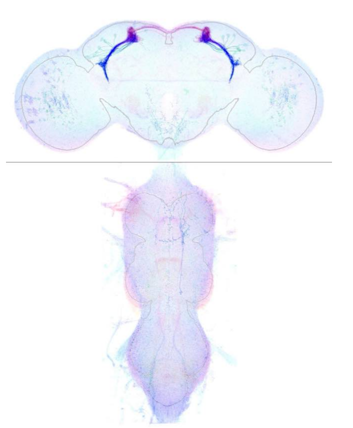
SS81557

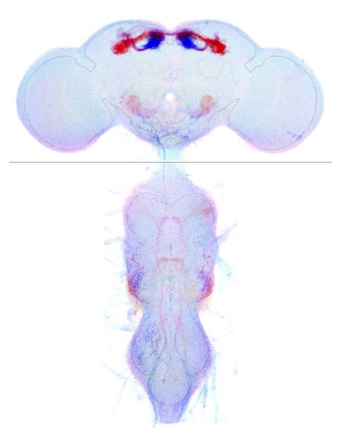
SS81561

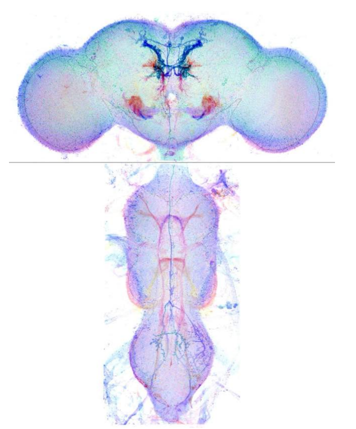
SS81567

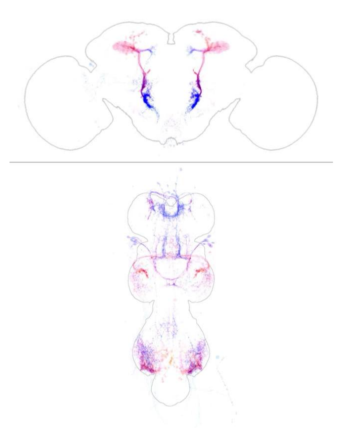
SS81667

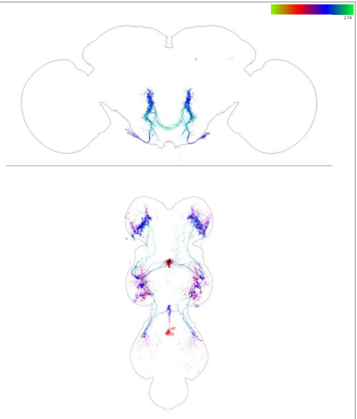

SS81722

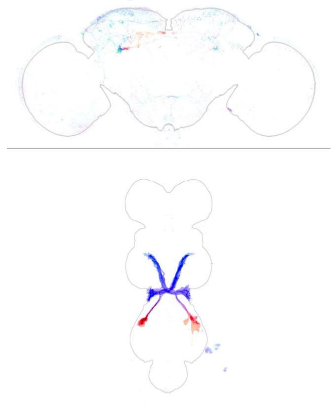

SS81814

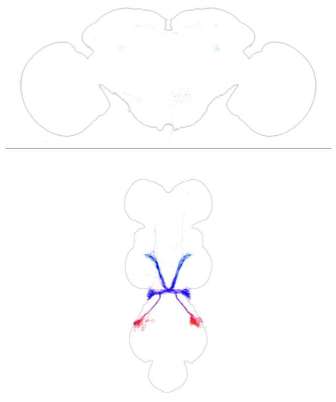

SS81815

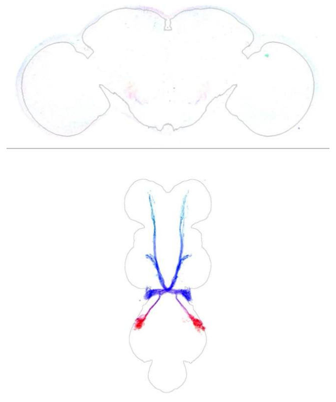

SS81816

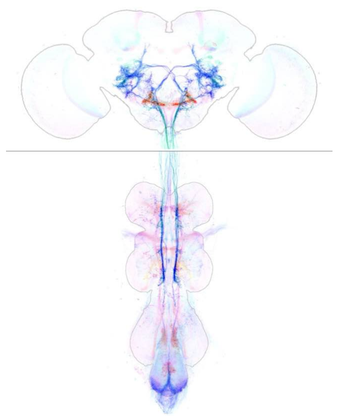

SS81818

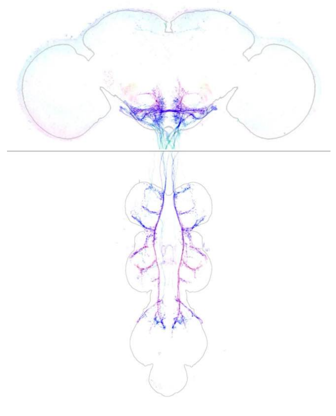

SS81829

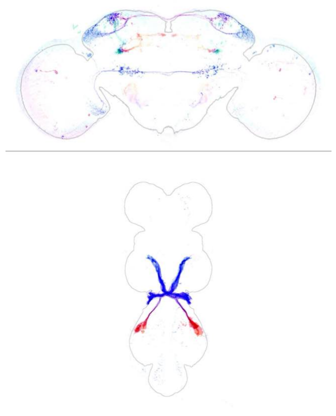

SS81837

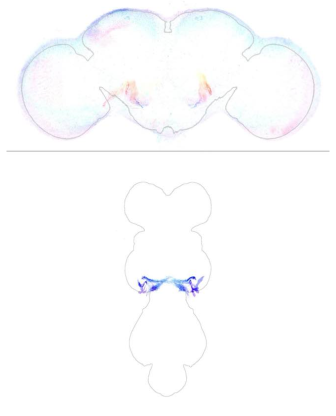

SS81860

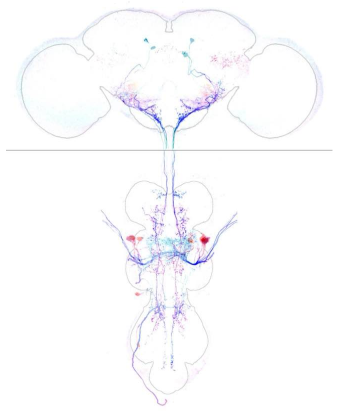

SS81905

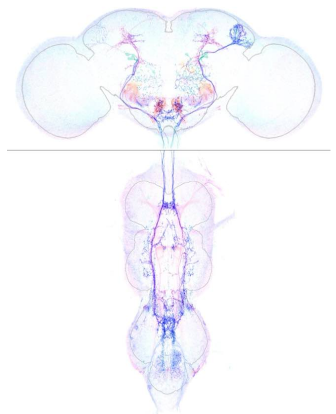

SS81912

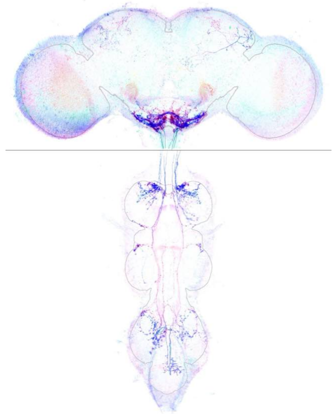

SS81919

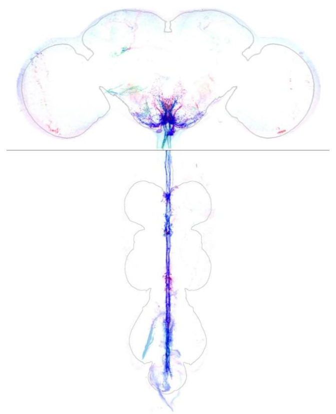

SS81923

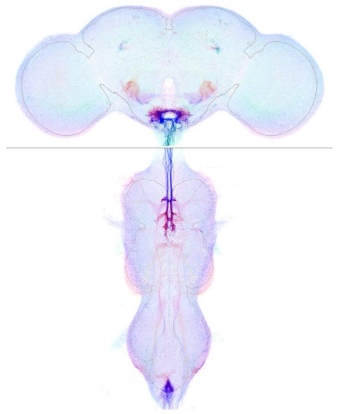

SS81934

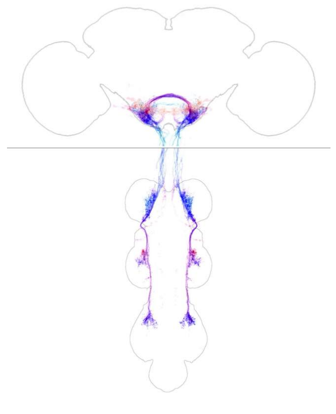

SS81943

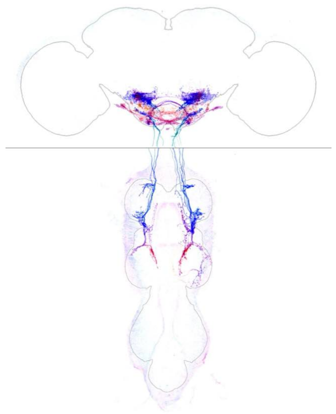

SS81946

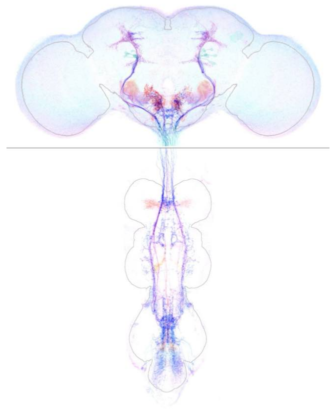

SS81962

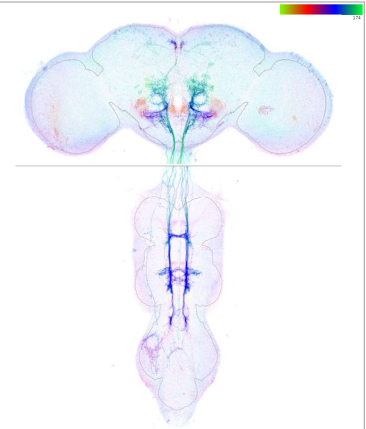

SS81965

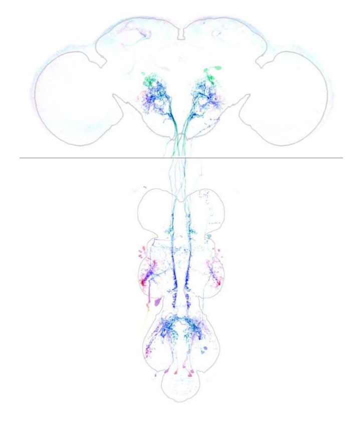

SS81969

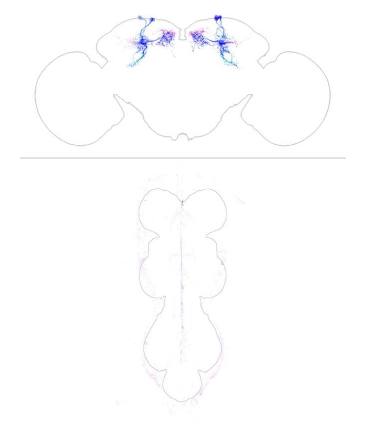

SS82088

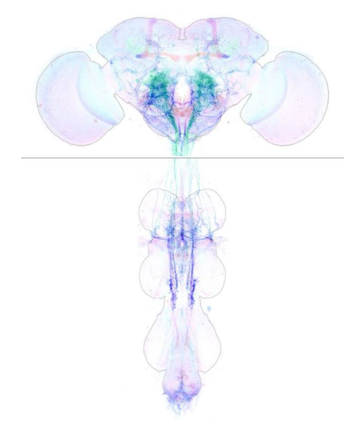

SS82242

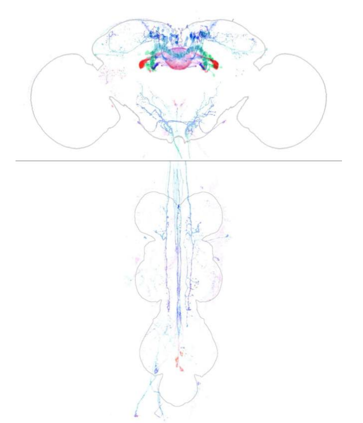

SS82331

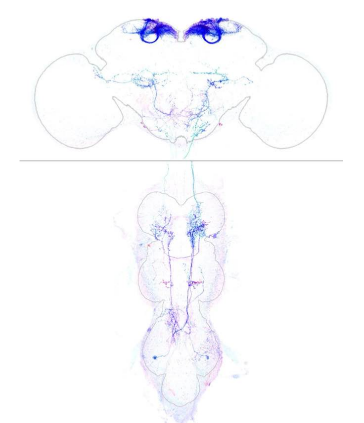

SS82405

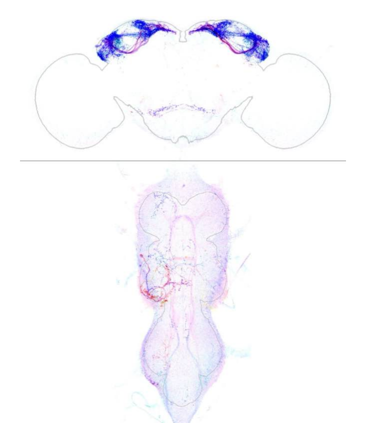

SS82503

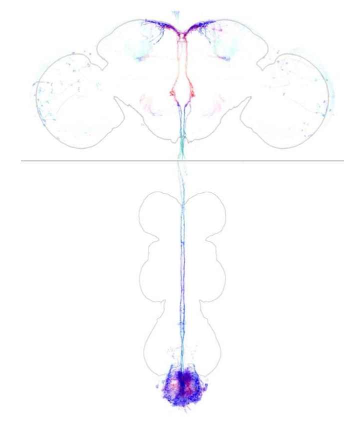

SS82529

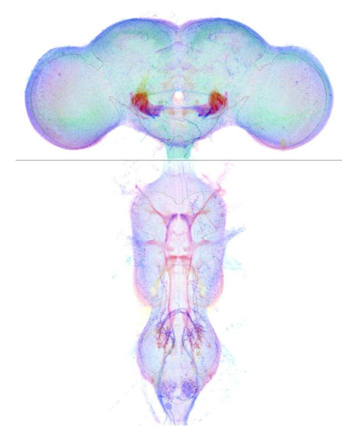

SS82614

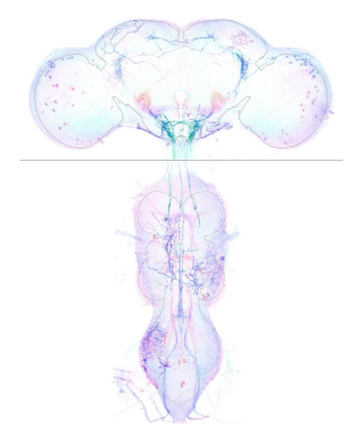

SS82954

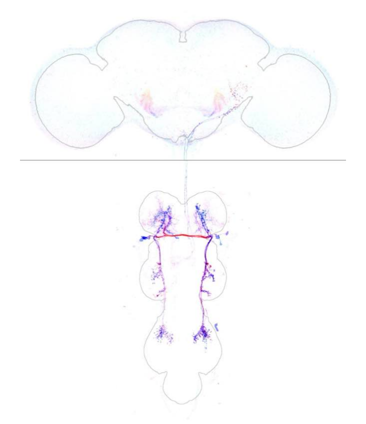

SS82974

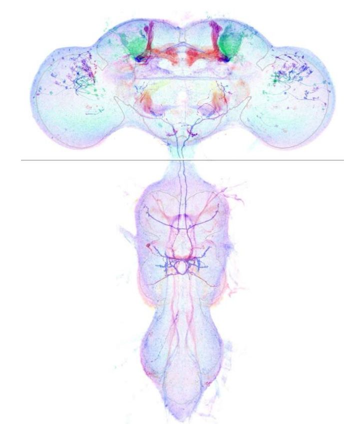

SS83003

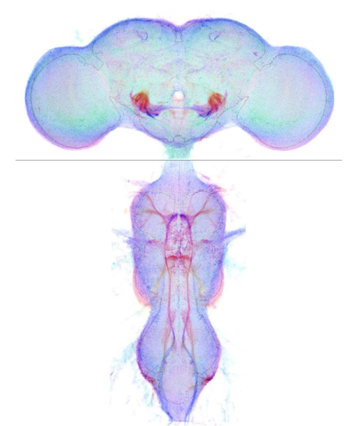

SS83018

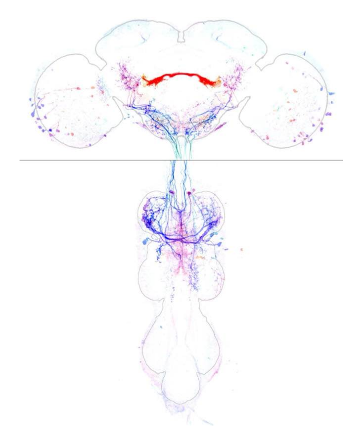

SS83077

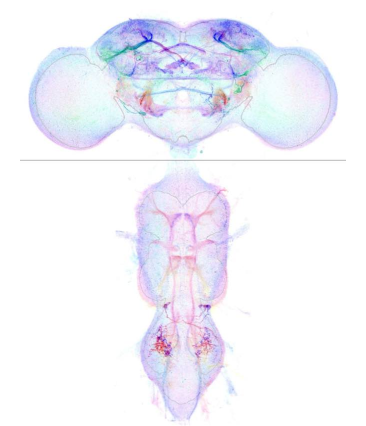

SS83118

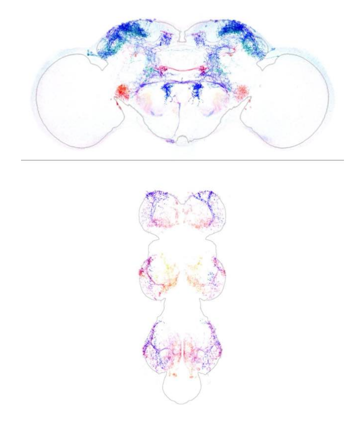

SS83354

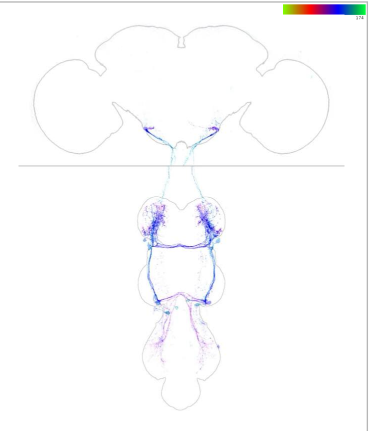

SS83382

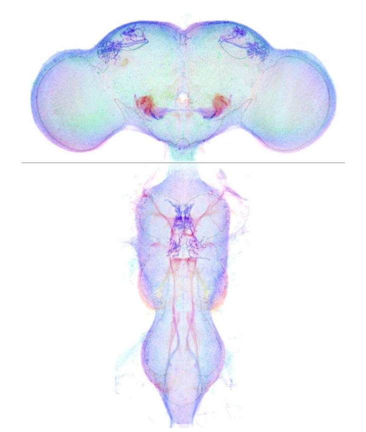

SS83414

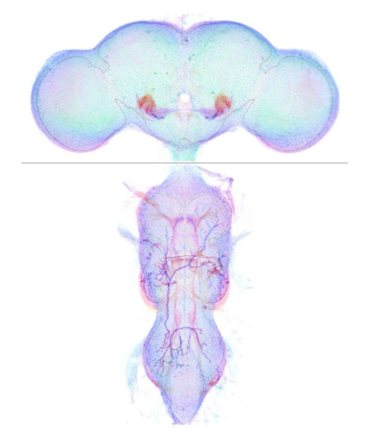

SS83440

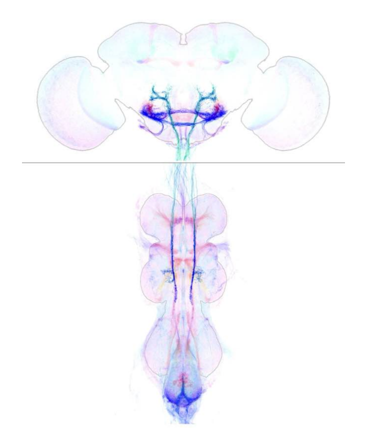

SS83497

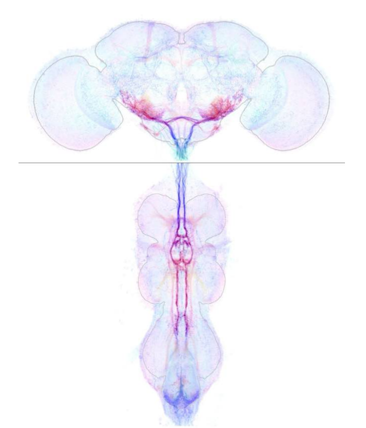

SS83514

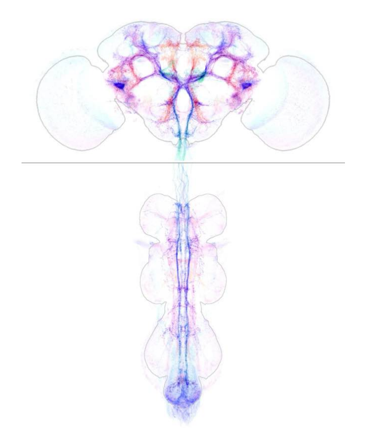

SS83545

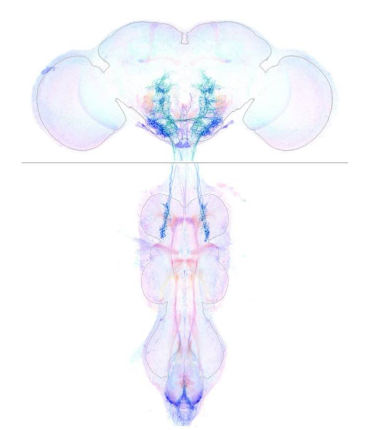

SS83573

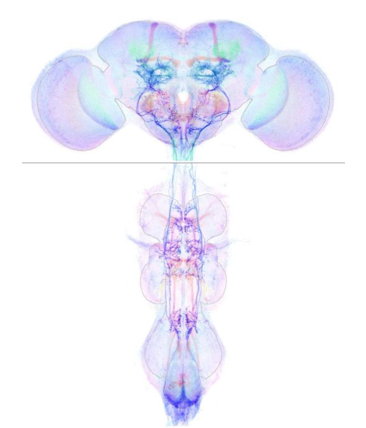

SS83614

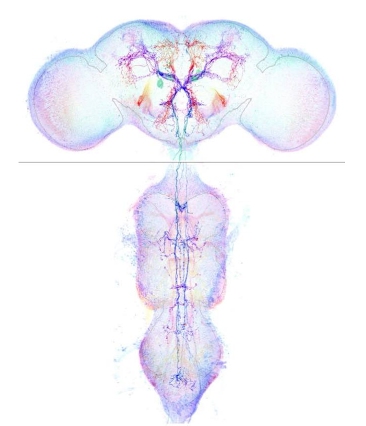

SS83615

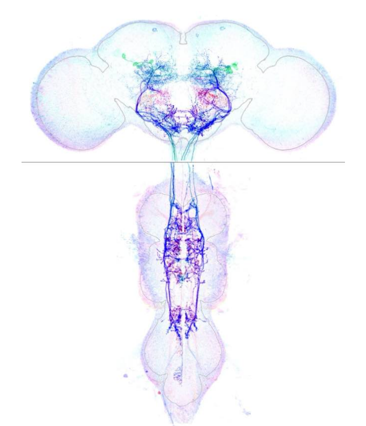

SS83628

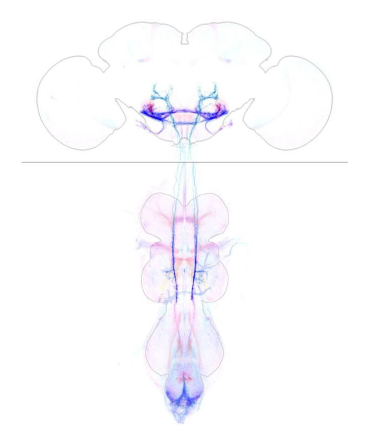

SS83634

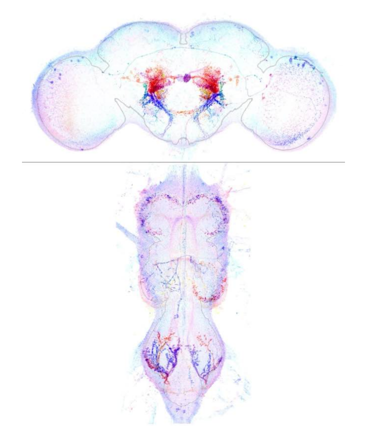

SS83676

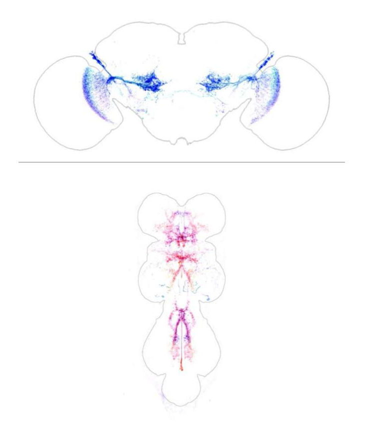

SS83694

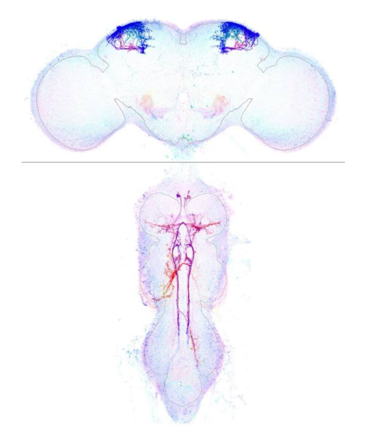

SS83695

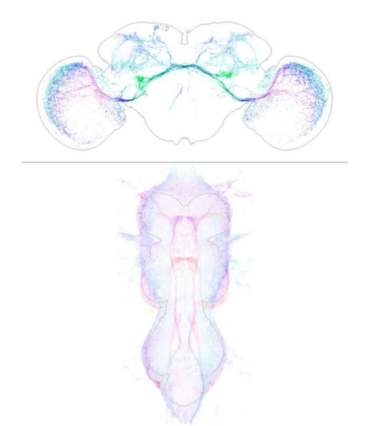

SS83741

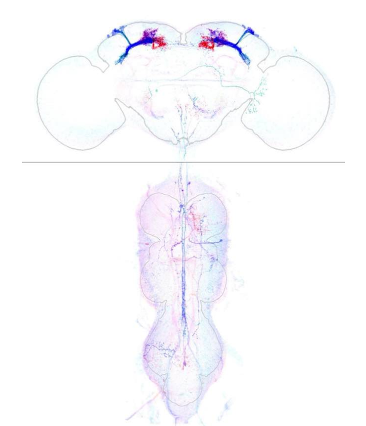

SS83861

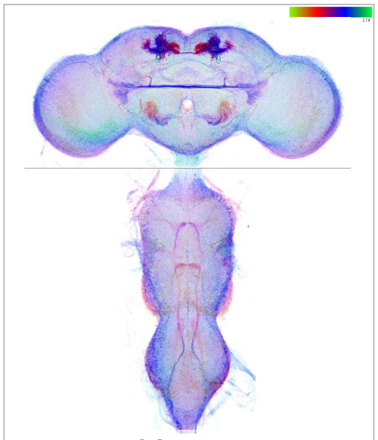
SS83894

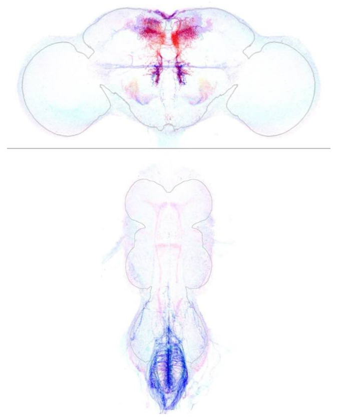
SS83956

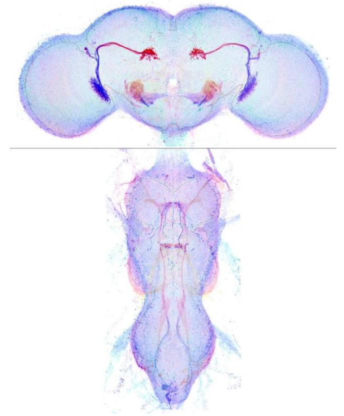
SS84010

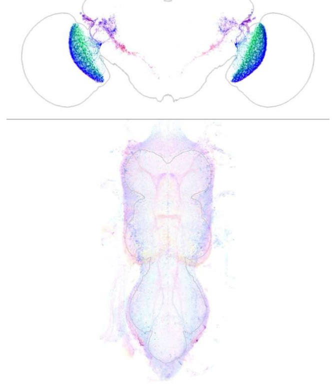
SS84053

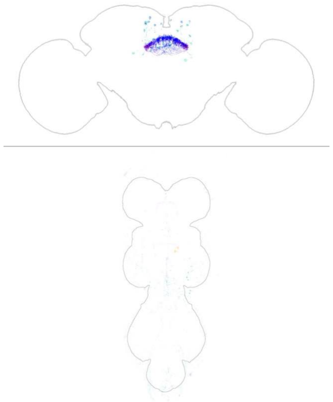
SS84364

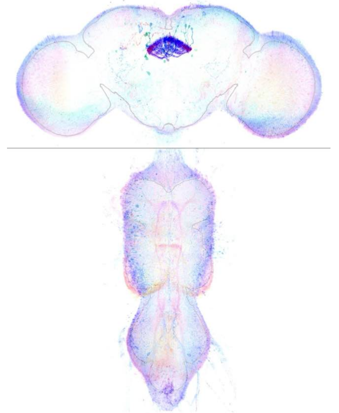
SS84383

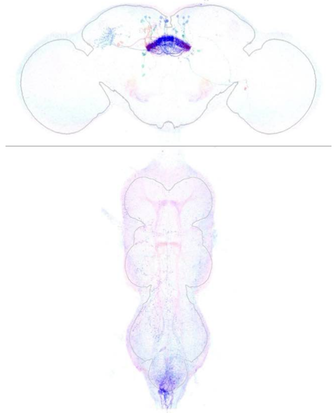
SS84385

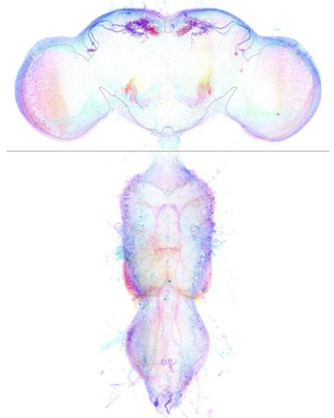
SS84406

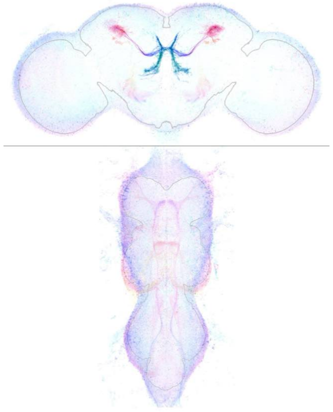
SS84473

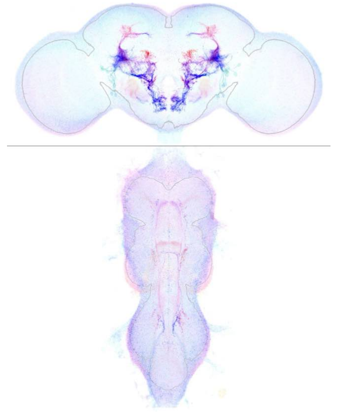
SS84487

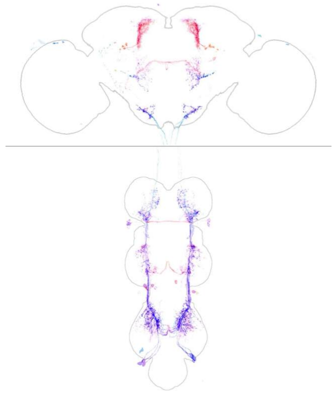
SS84628

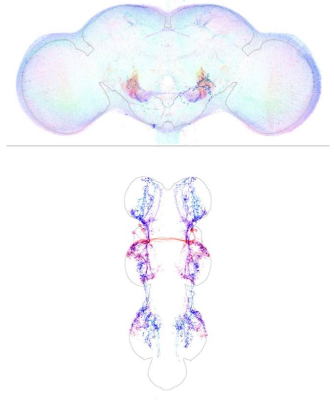
SS84697

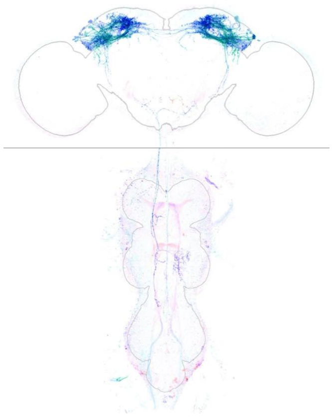
SS84841

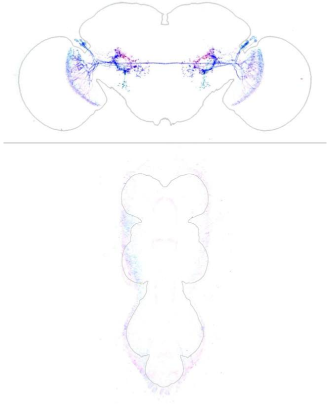
SS84843

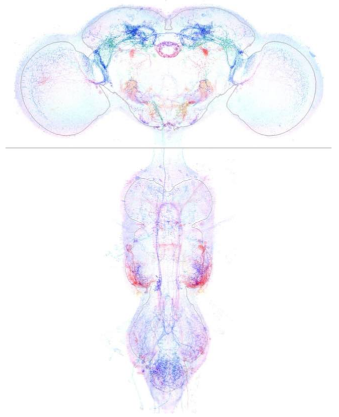
SS84857

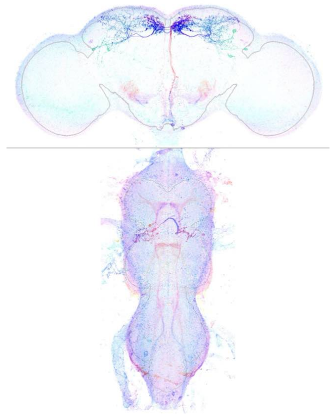
SS84880

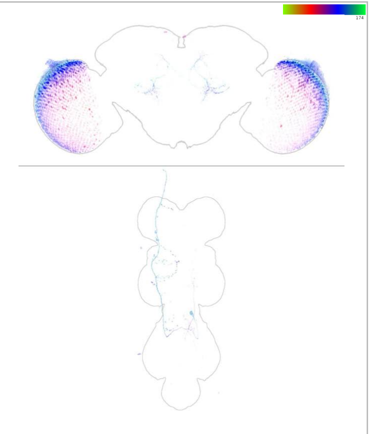
SS84898

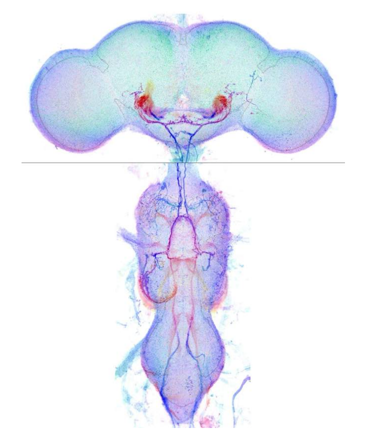
SS85151

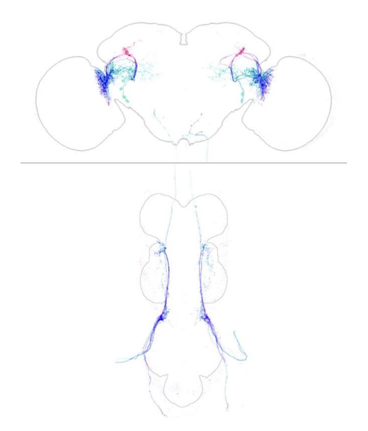
SS85183

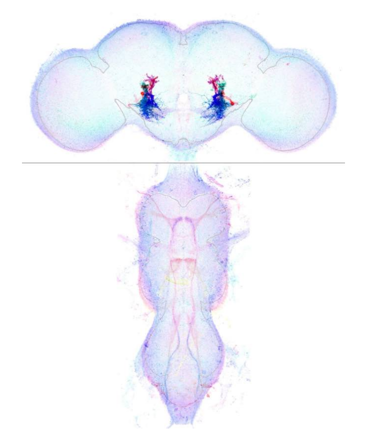
SS85191

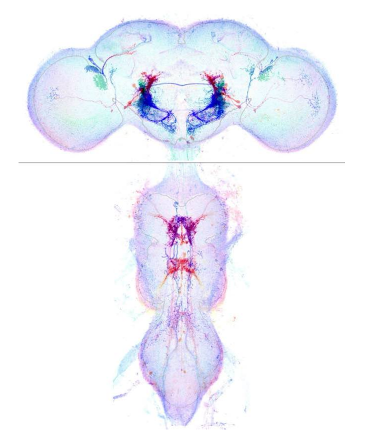
SS85196

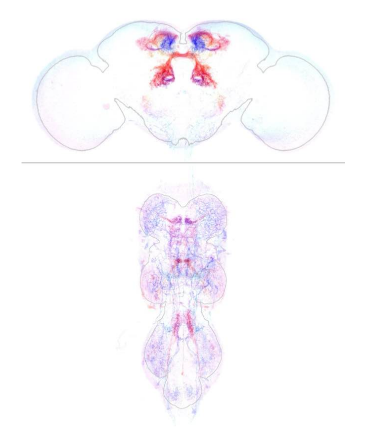
SS85243

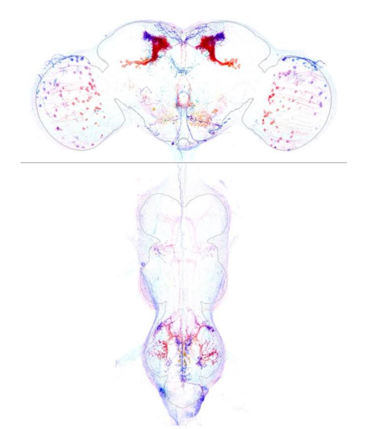
SS85348

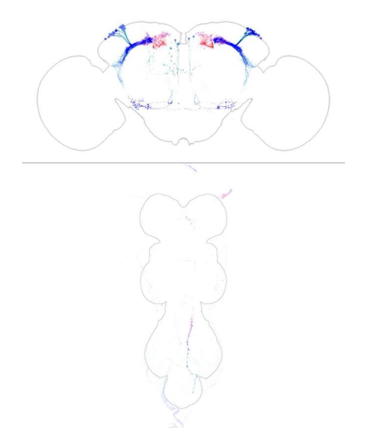
SS85535

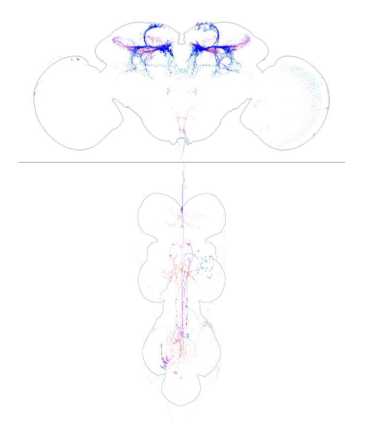
SS85568

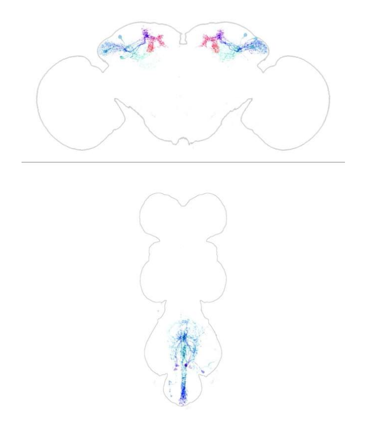
SS85572

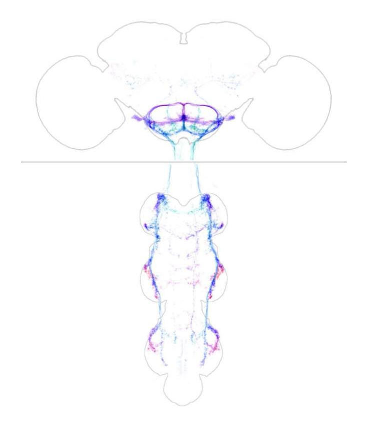
SS85855

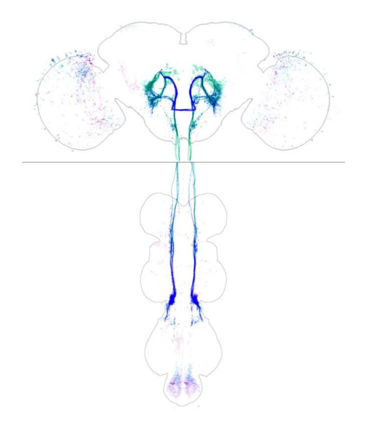
SS85873

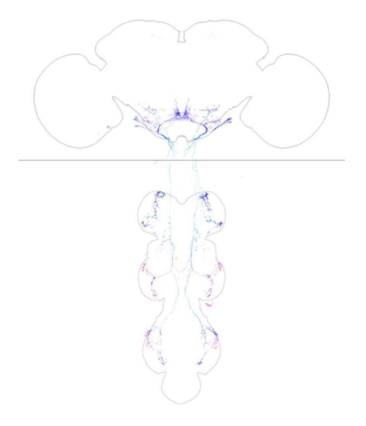
SS85917

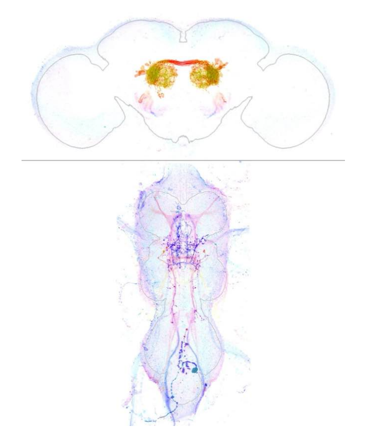
SS85927

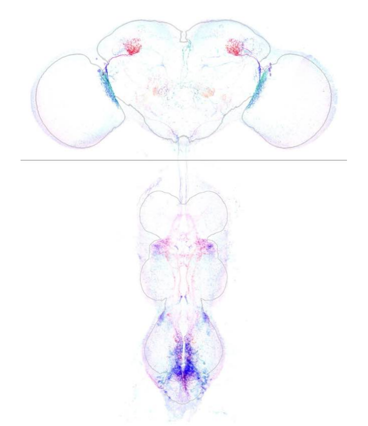
SS85942

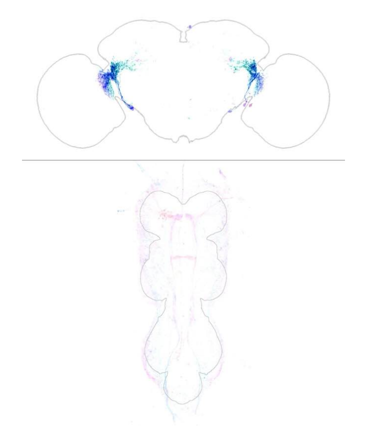
SS85949

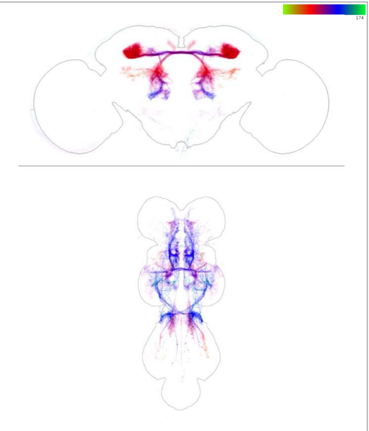
SS85999

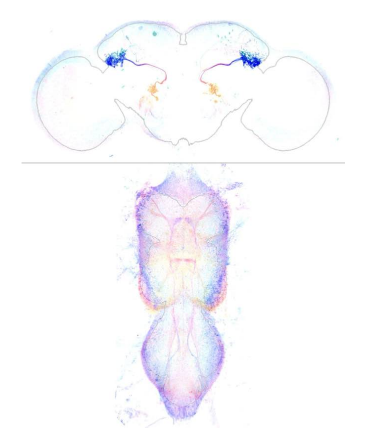
SS86025

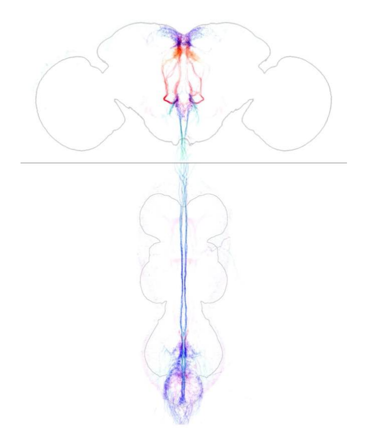
SS86098

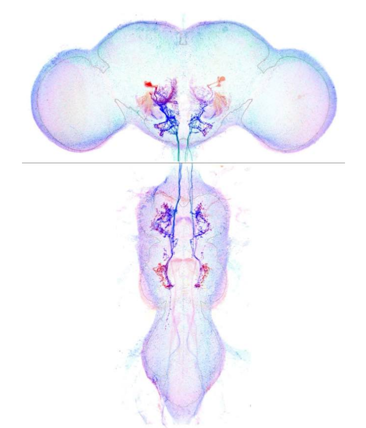
SS86129

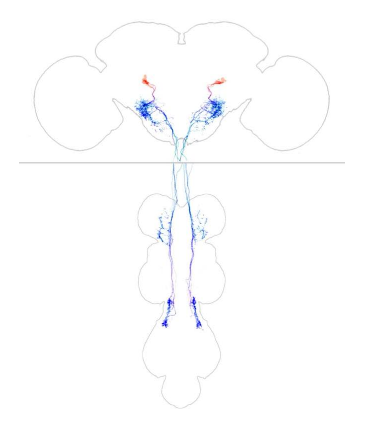
SS86130

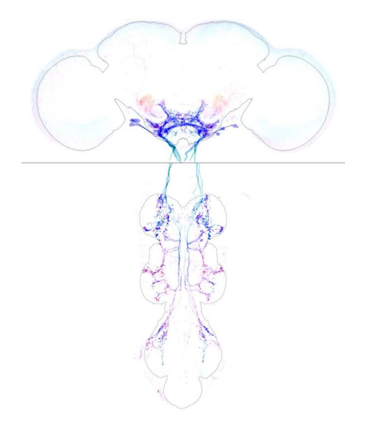
SS86205

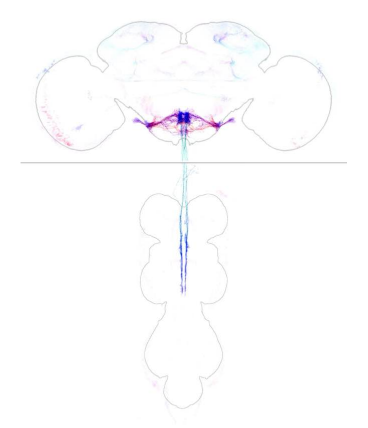
SS86249

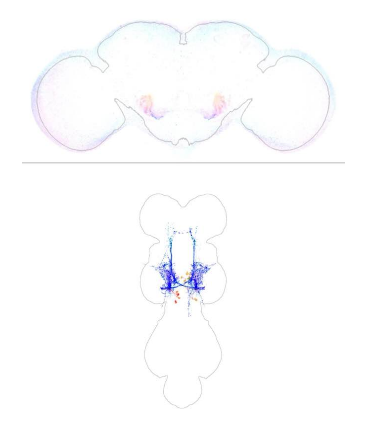
SS86265

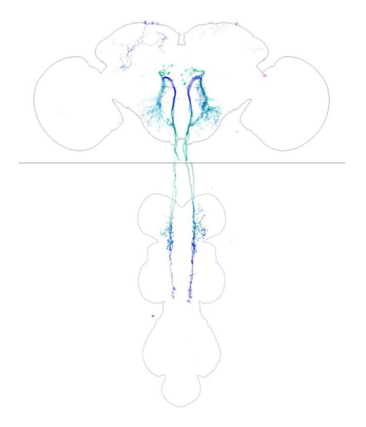
SS86302

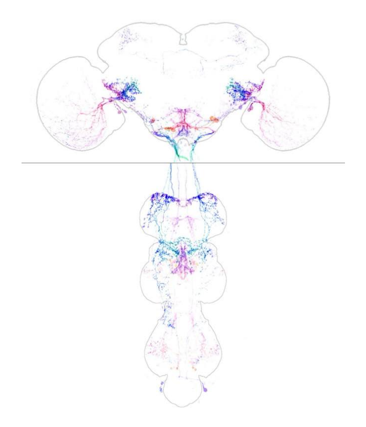
SS86366

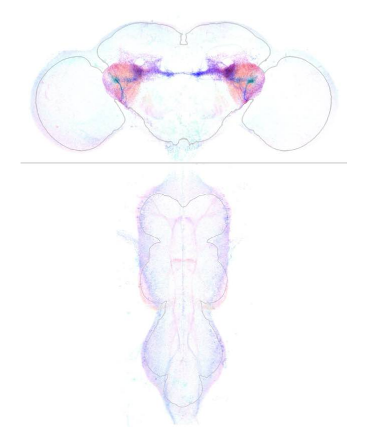
SS86464

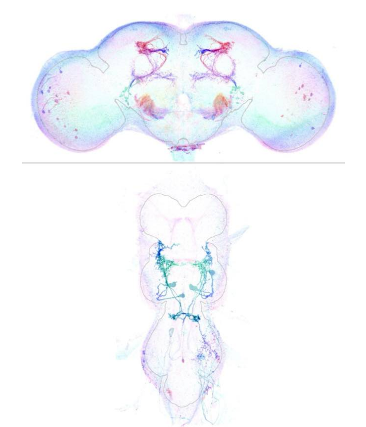
SS86766

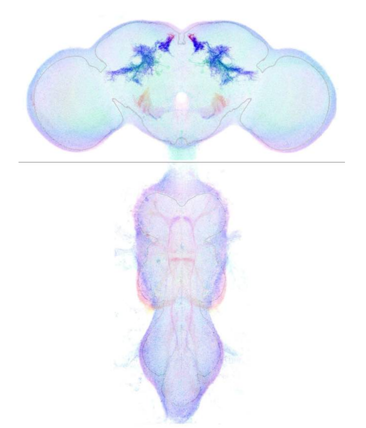
SS86882

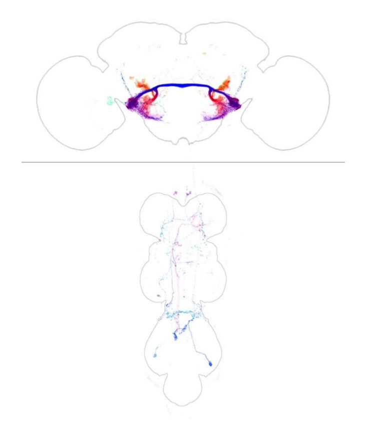
SS86904

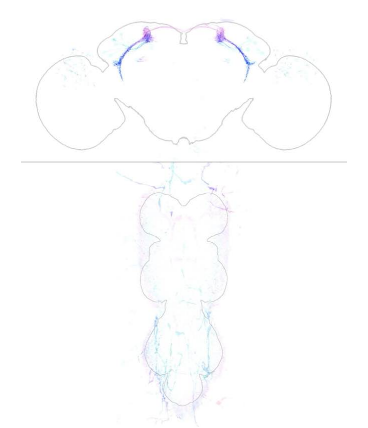
SS86947

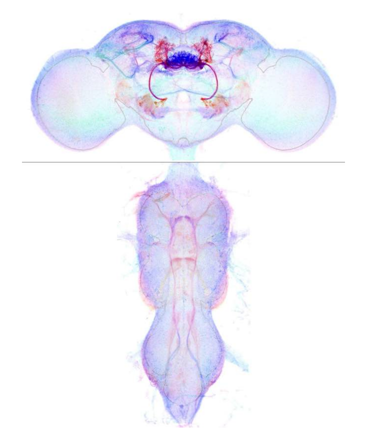
SS87118

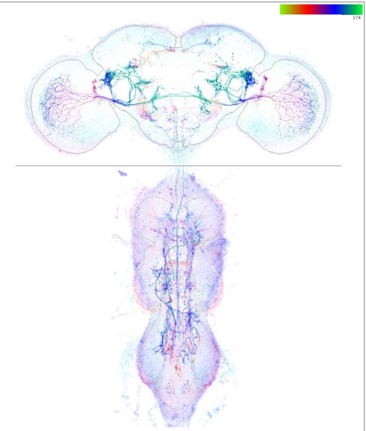
SS87189

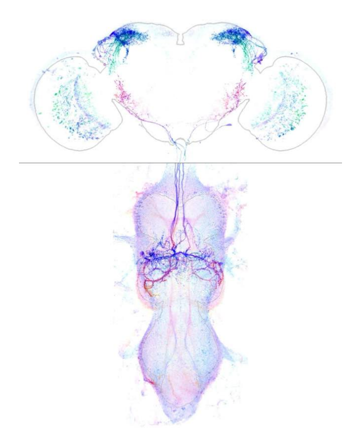
SS87195

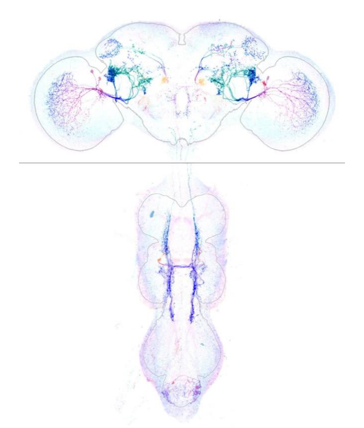
SS87220

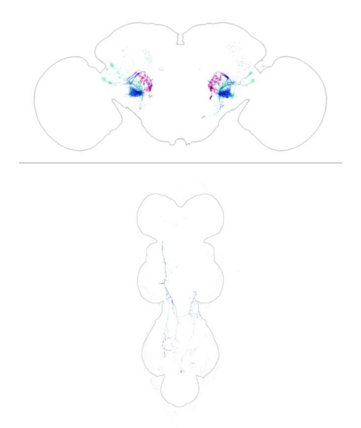
SS87239

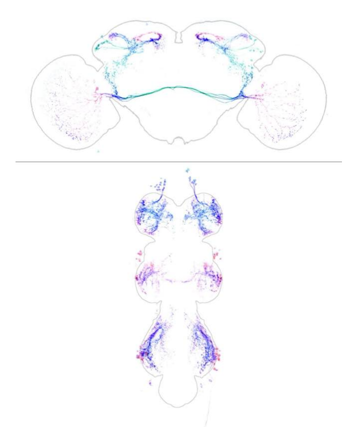
SS87242

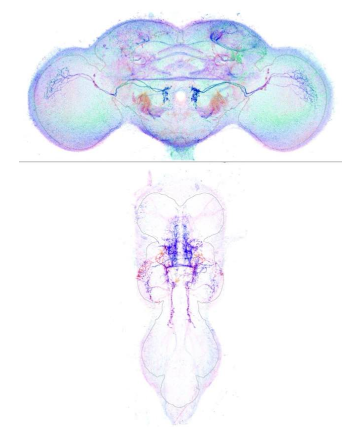
SS87246

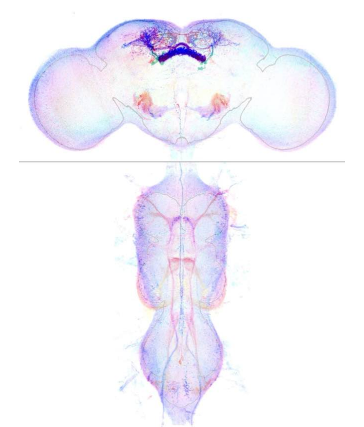
SS87368

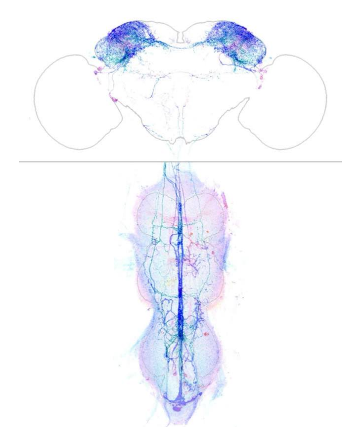
SS87416

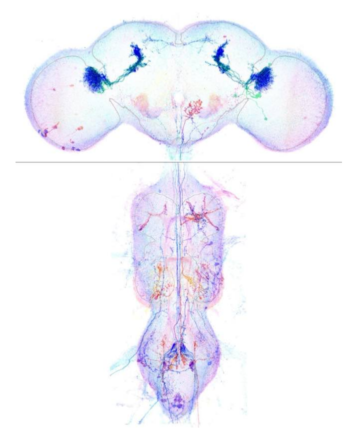
SS87547

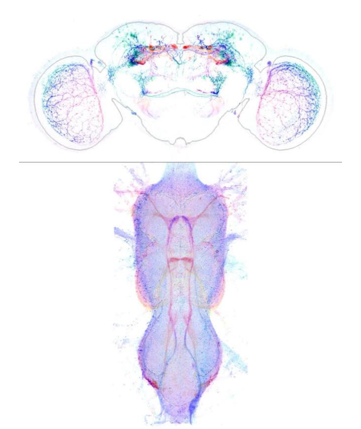
SS87587

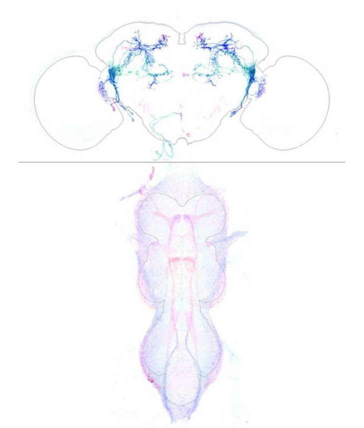
SS87589

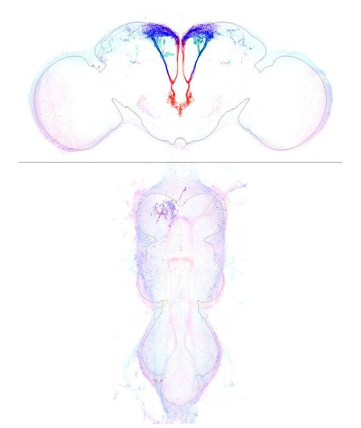
SS87605

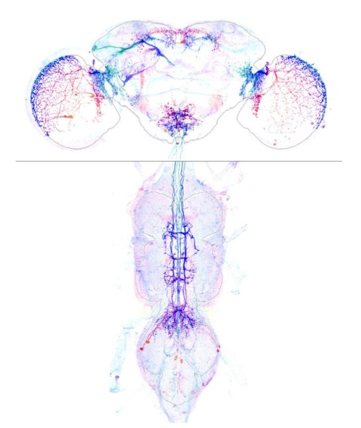
SS87616

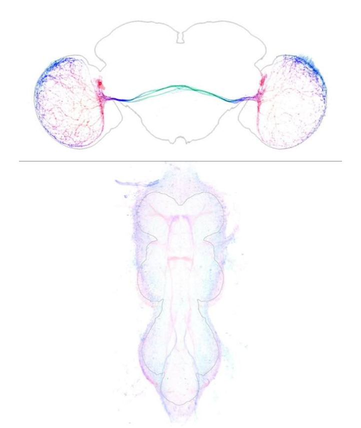
SS87651

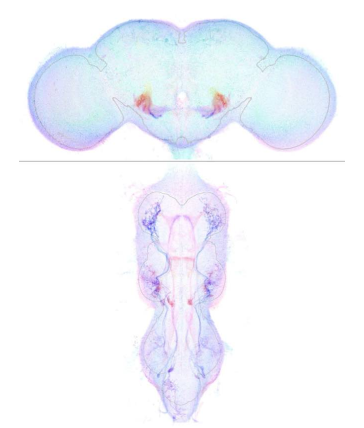
SS87668

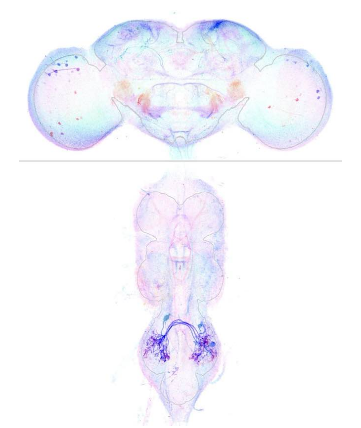
SS87705

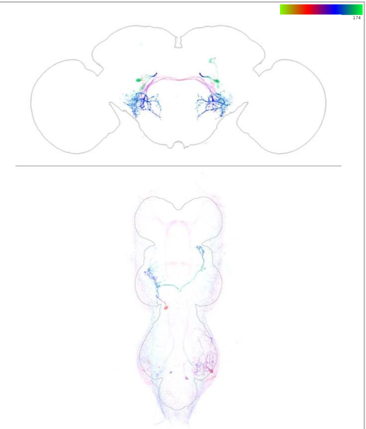
SS87906

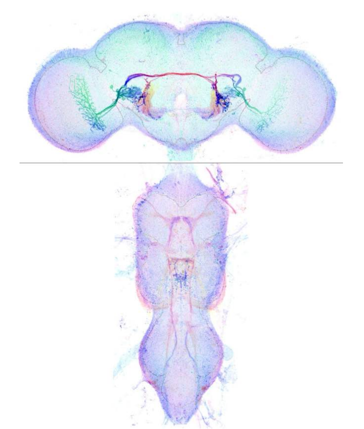
SS87930

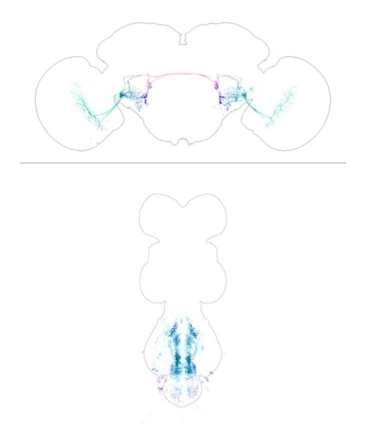
SS87944

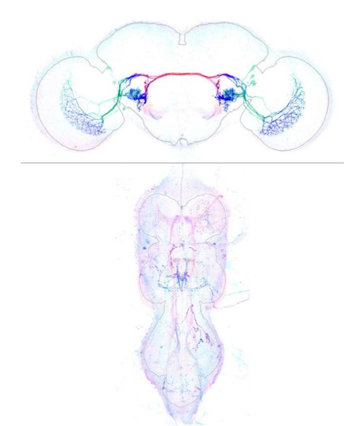
SS87981

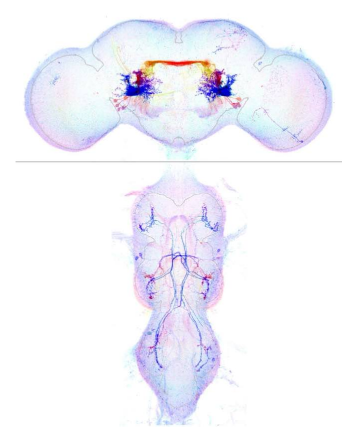
SS87995

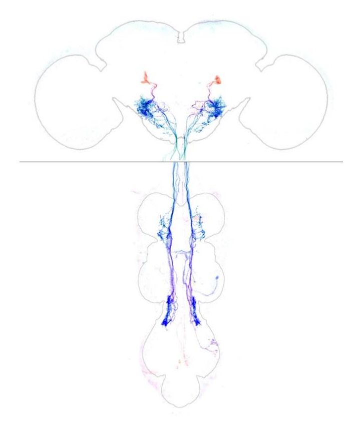
SS88143

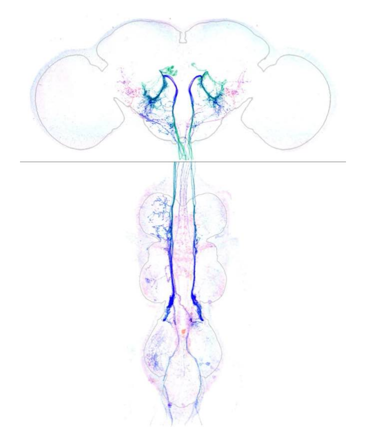
SS88197

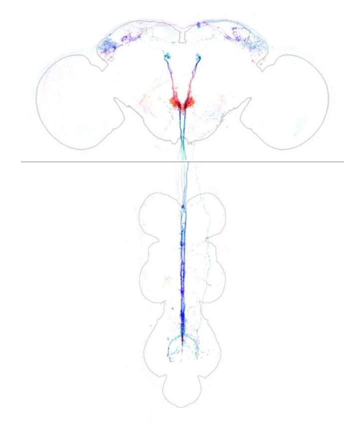
SS88242

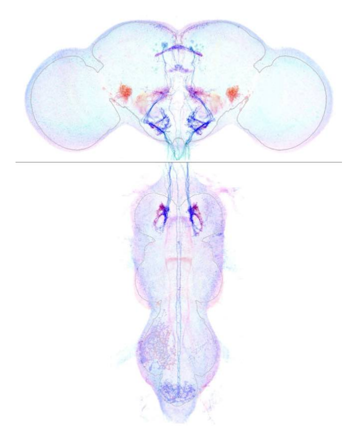
SS88245

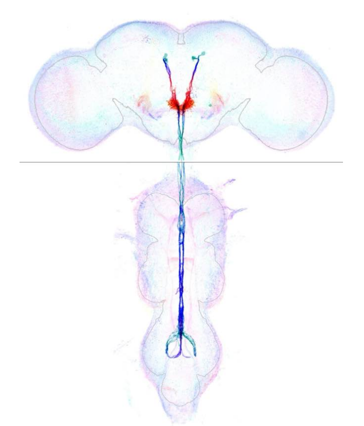
SS88246

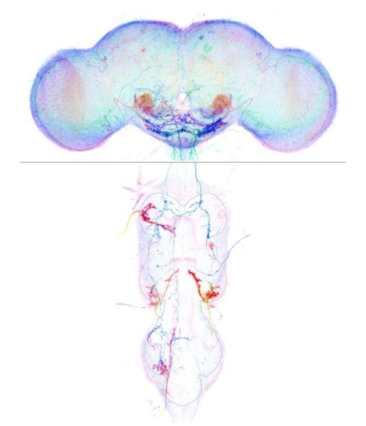
SS88264

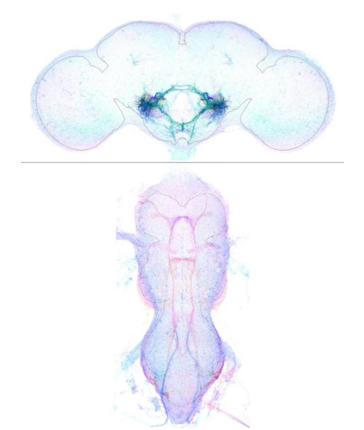
SS88313

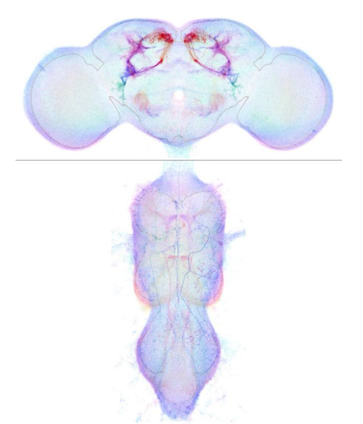
SS88322

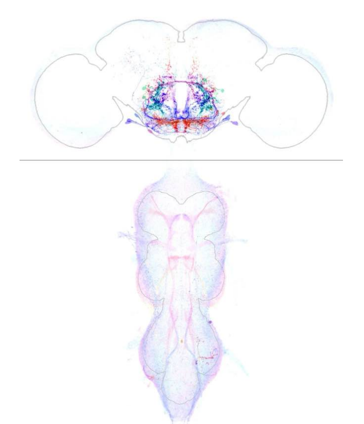
SS88441

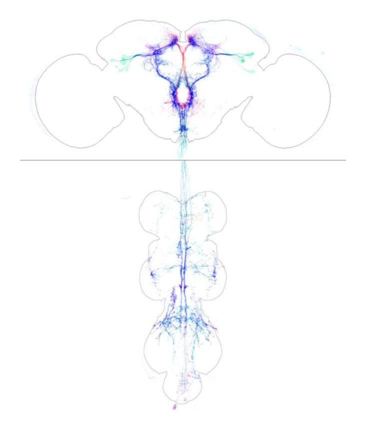
SS88586

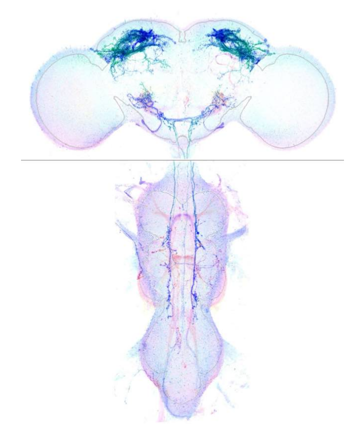
SS88672

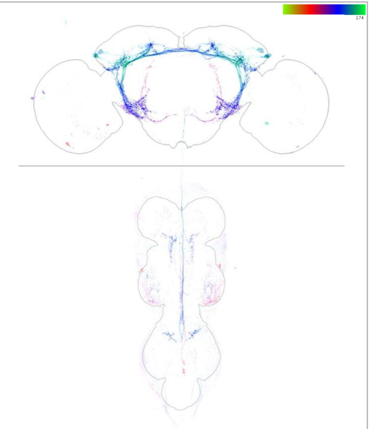
SS88901

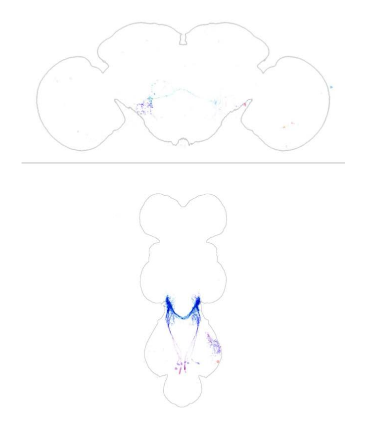
SS88938

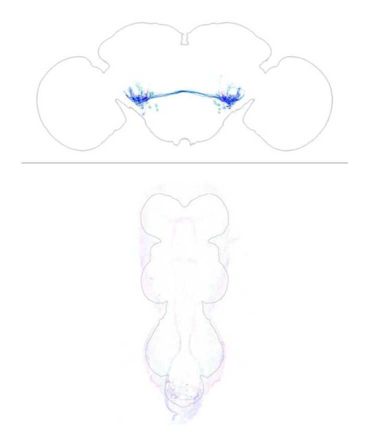
SS89005

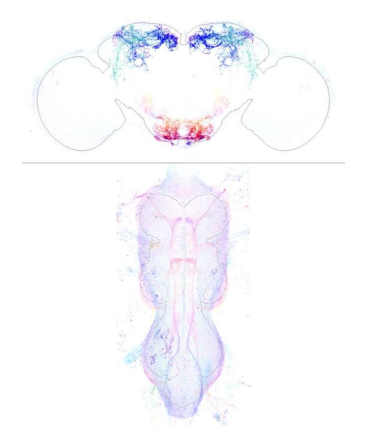
SS89024

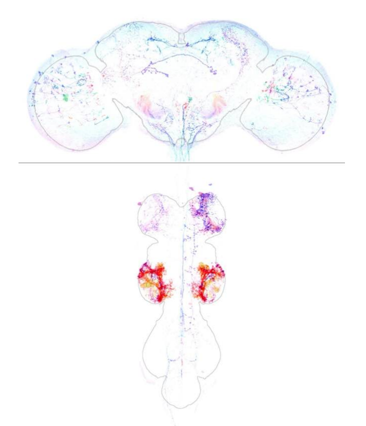
SS89292

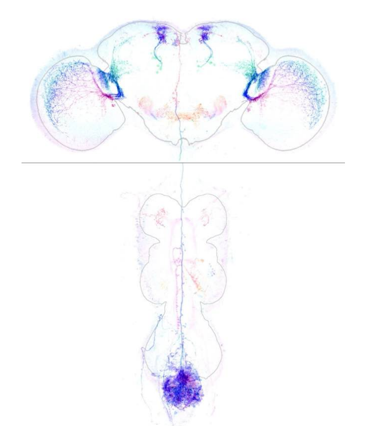
SS89446

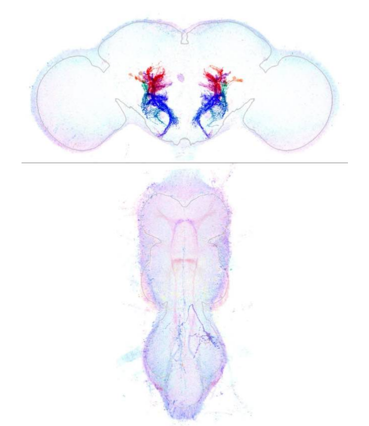
SS89447

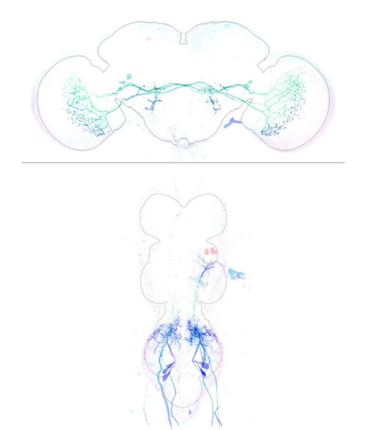
SS89455

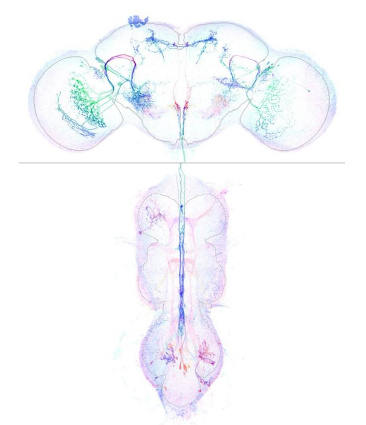
SS89516

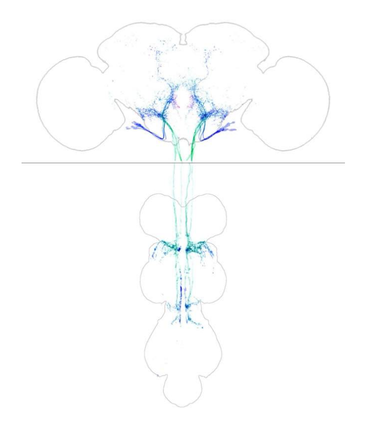
SS89603

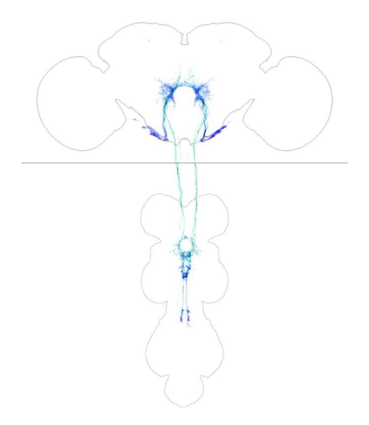
SS89637

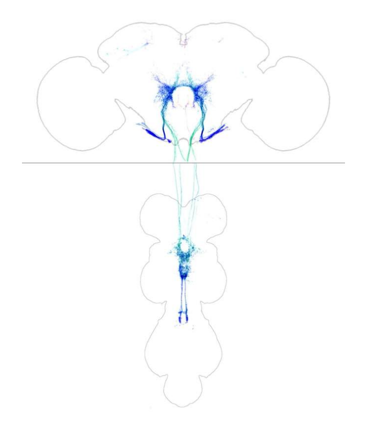
SS89712

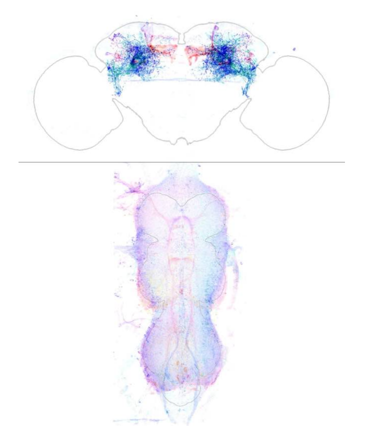
SS89869

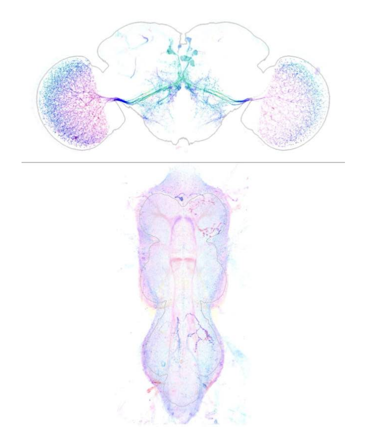
SS90112

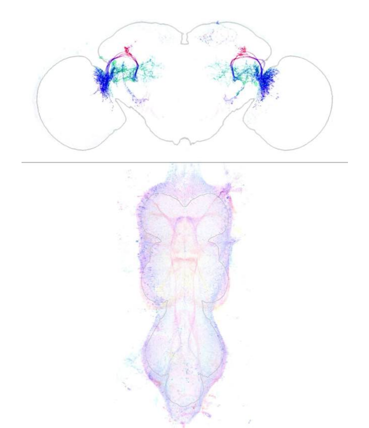
SS90154

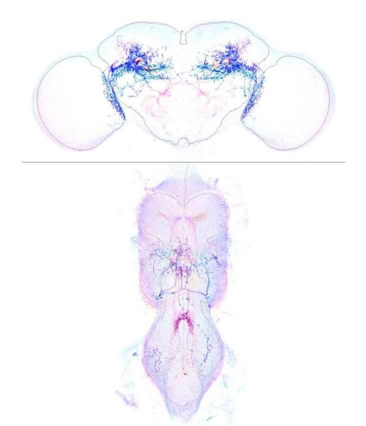
SS90162

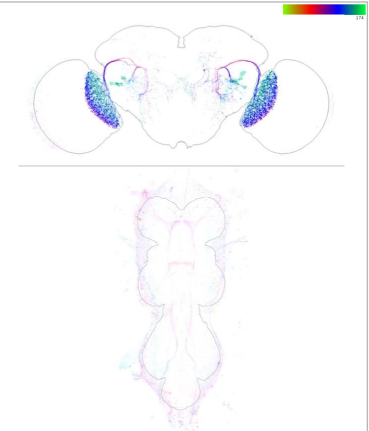

SS90166

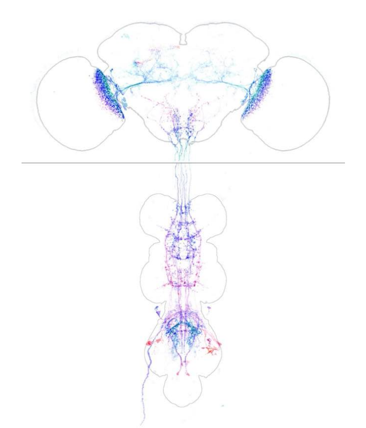

SS90245

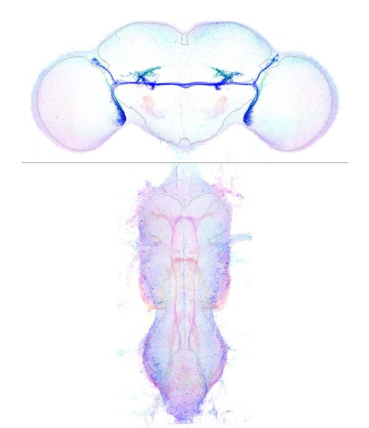

SS90275

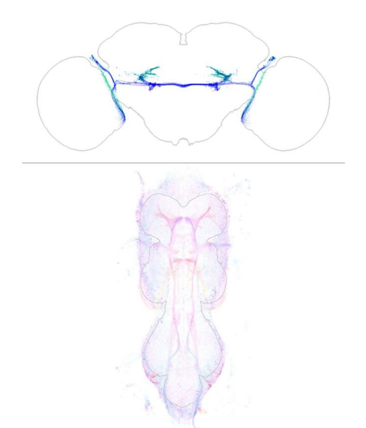

SS90303

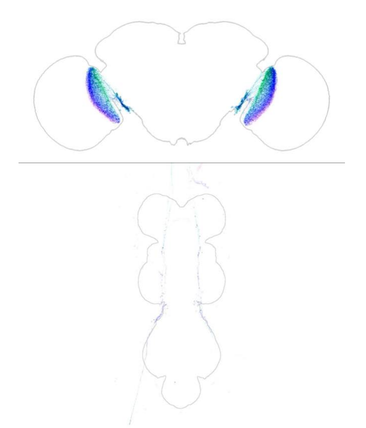

SS90337

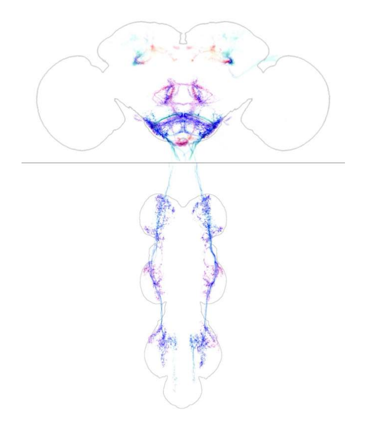

SS90782

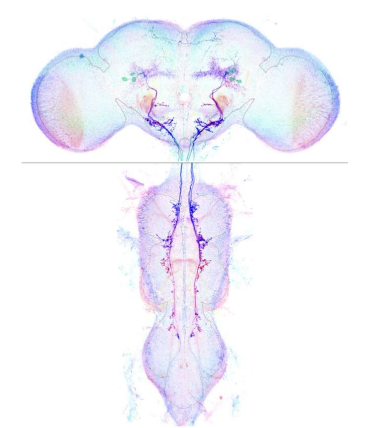

SS90829

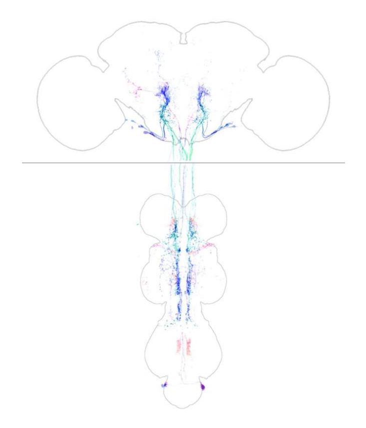

SS90856

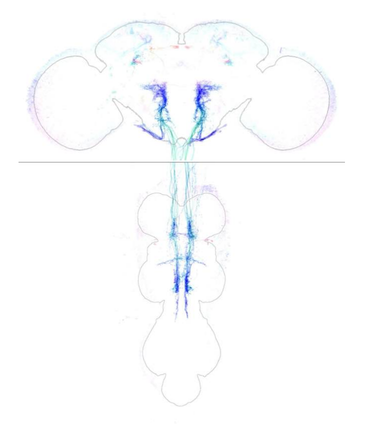

SS90868

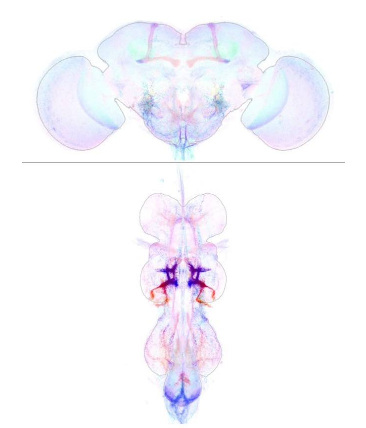

SS90876

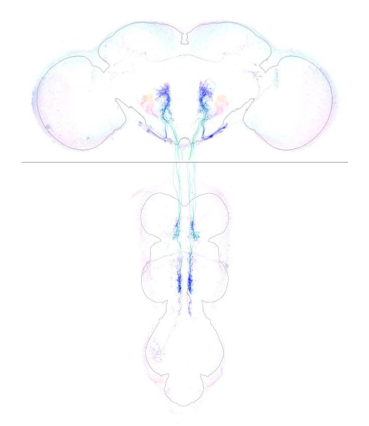

SS90918

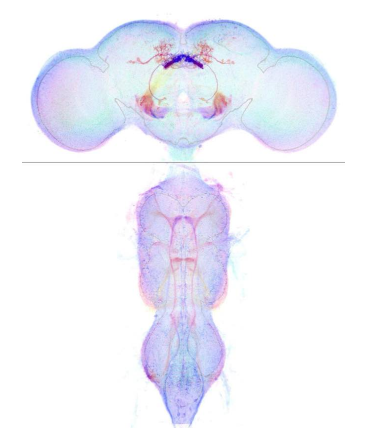

SS91010

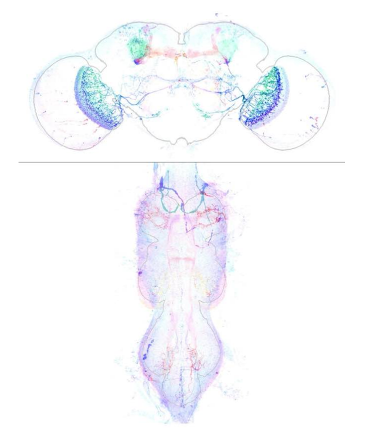

SS91076

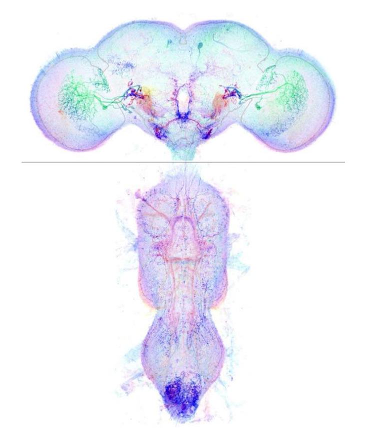

SS91414

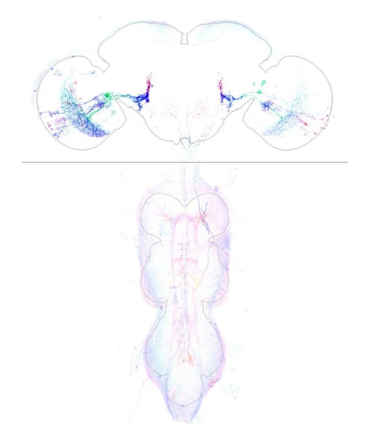

SS91415

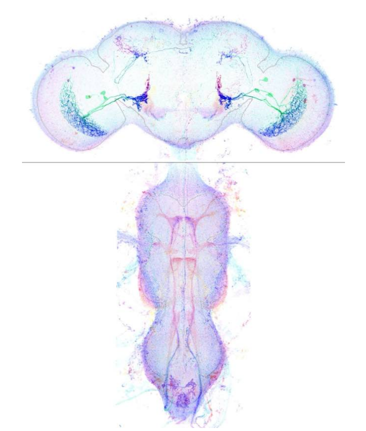

SS91494

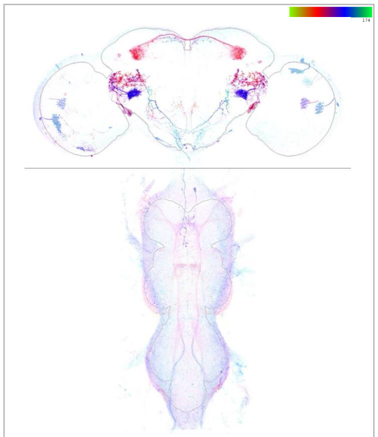

SS91727

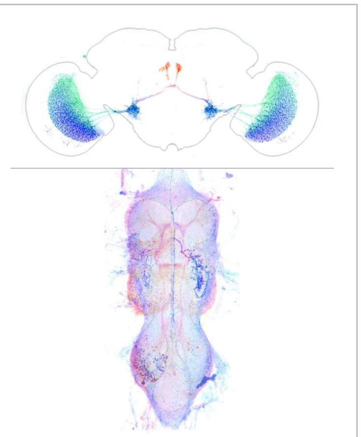

SS91799

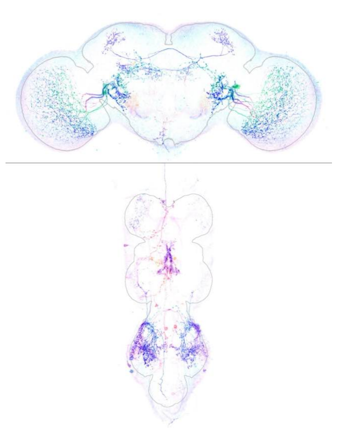

SS91814

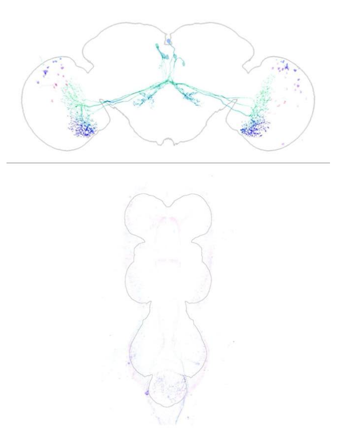

SS91829

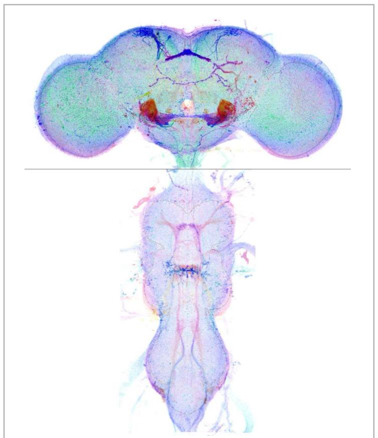

SS92555

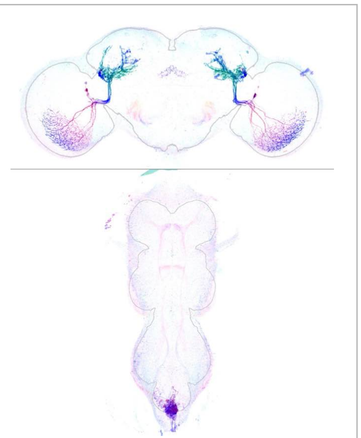

SS92560

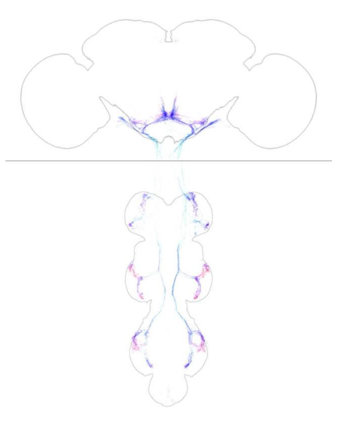

SS92658

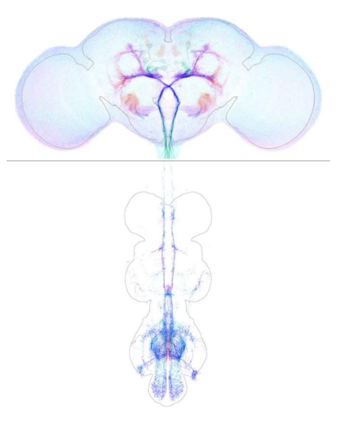

SS92713

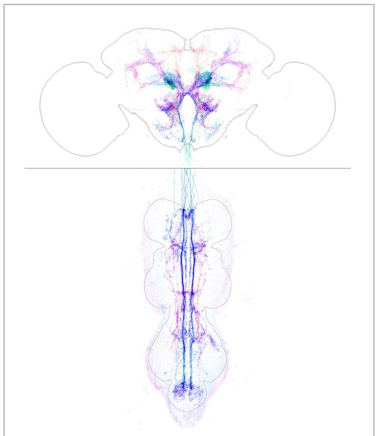

SS92748

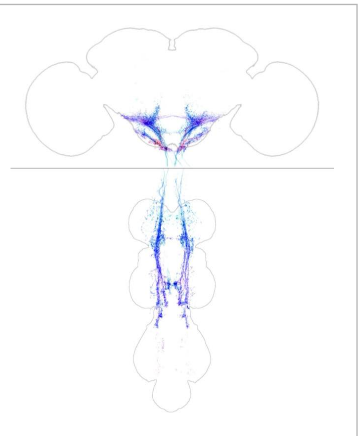

SS92759

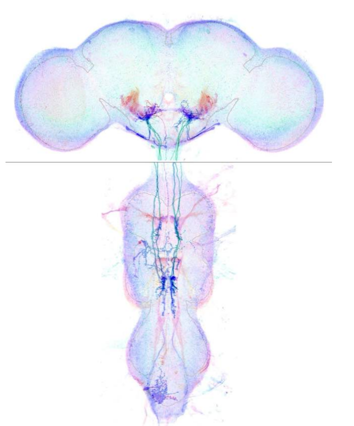

SS92768

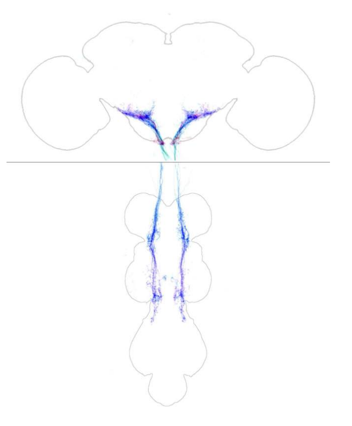

SS92805

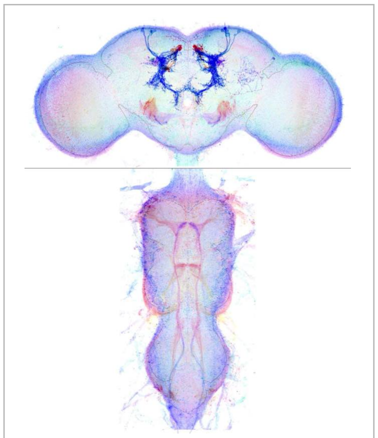

SS92939

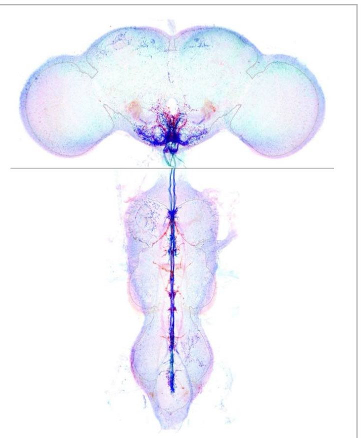

SS93166

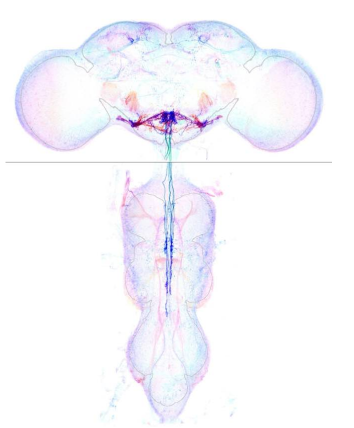

SS93646

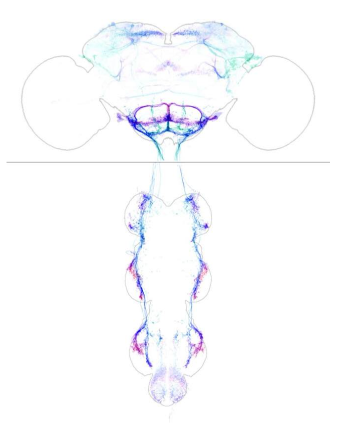

SS93763

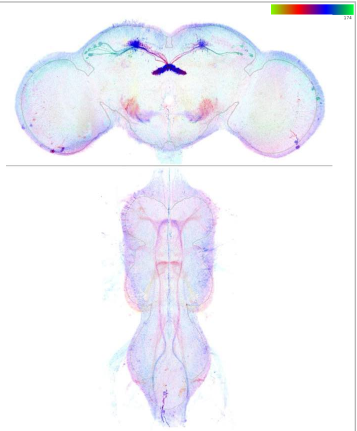
SS93973

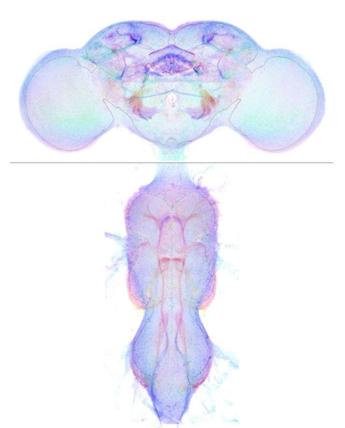
SS93987

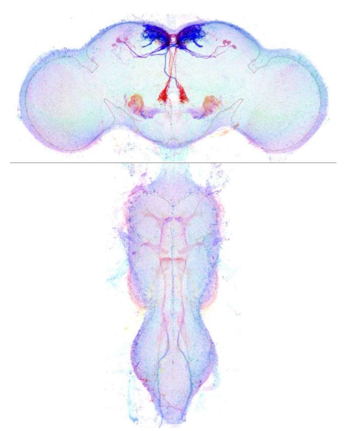
SS94386

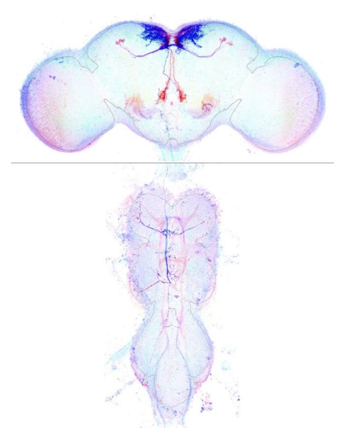
SS94388

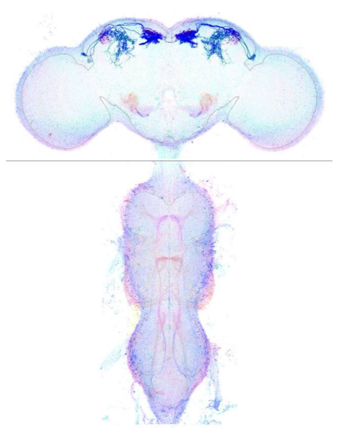
SS94414

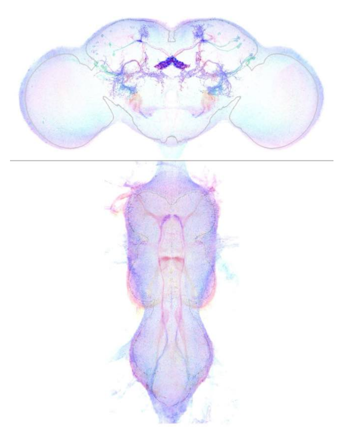
SS94479

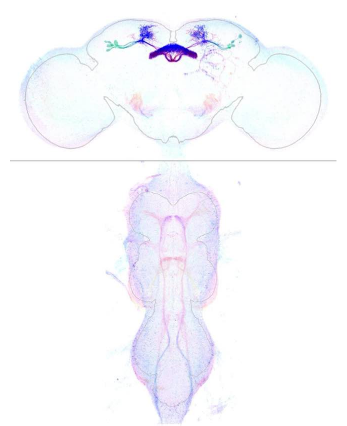
SS94480

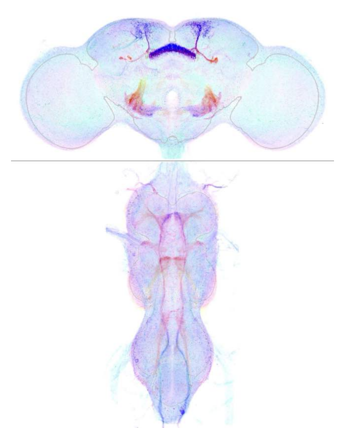
SS94527

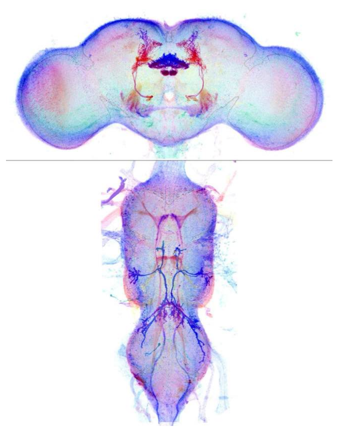
SS94615

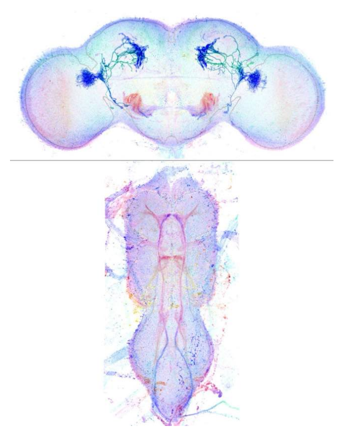
SS94653

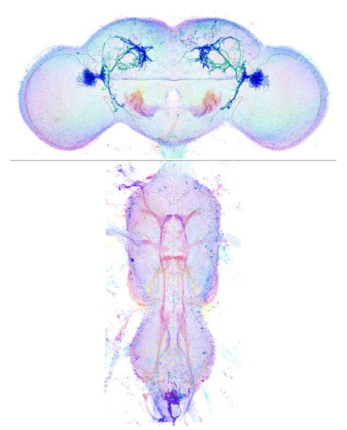
SS94671

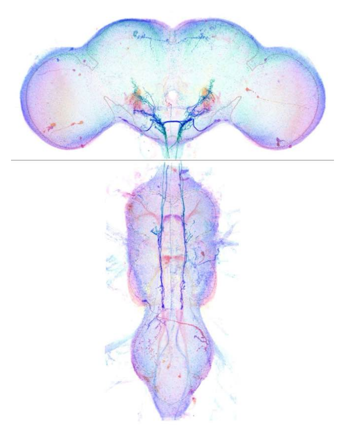
SS94744

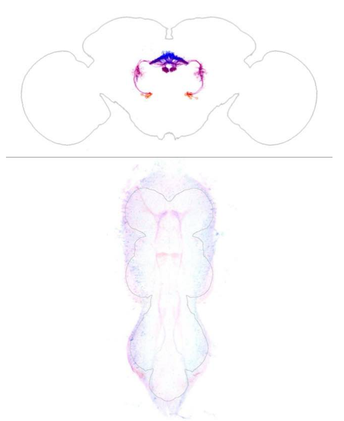
SS94879

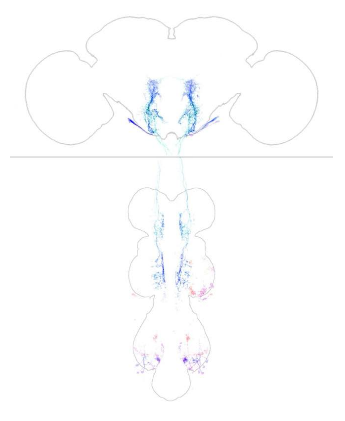
SS94997

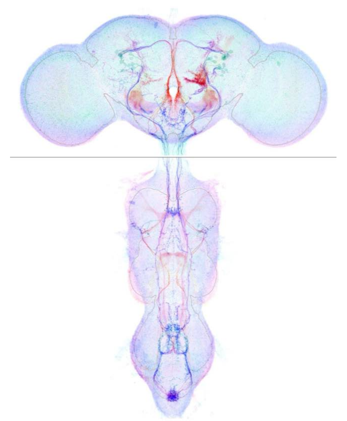
SS95004

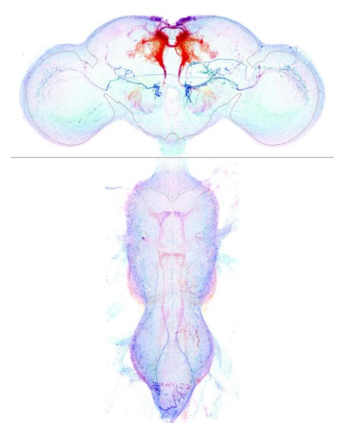
SS95107

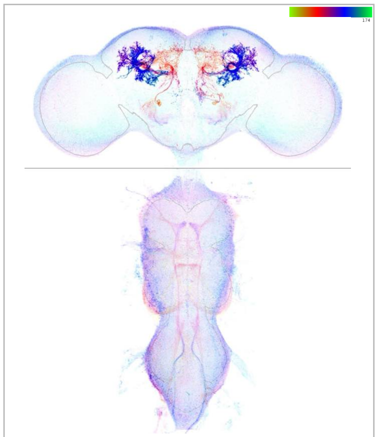
SS95118

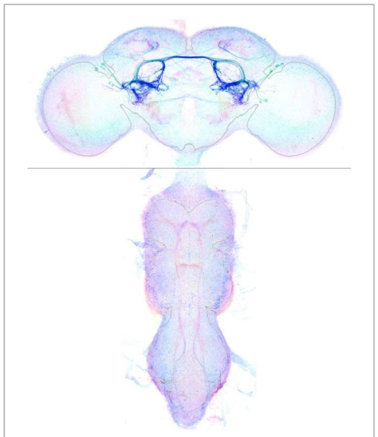
SS95125

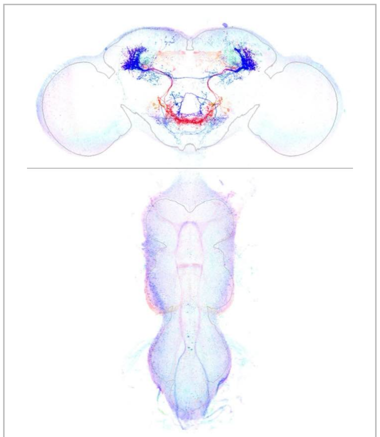
SS95158

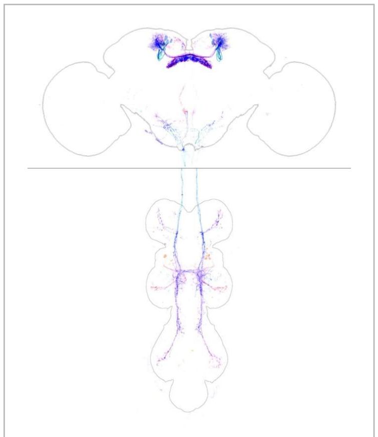
SS95274

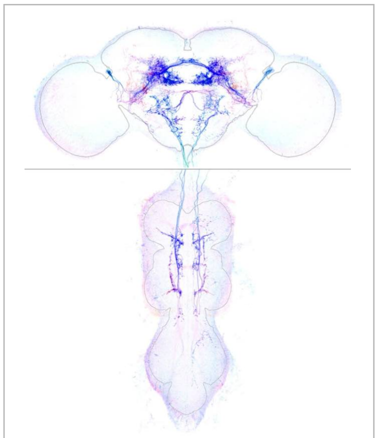
SS95330

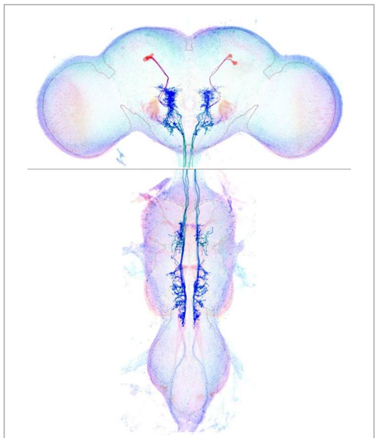
SS95335

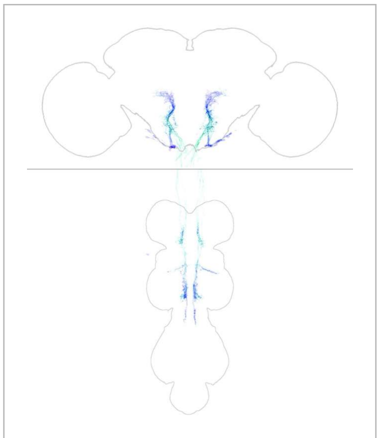
SS95356

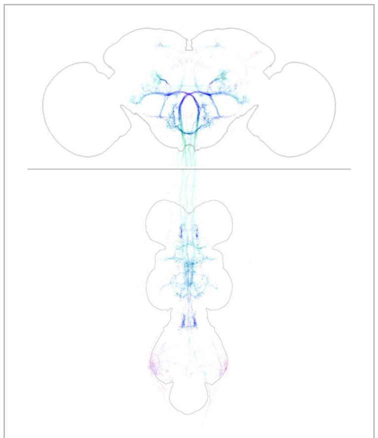
SS95362

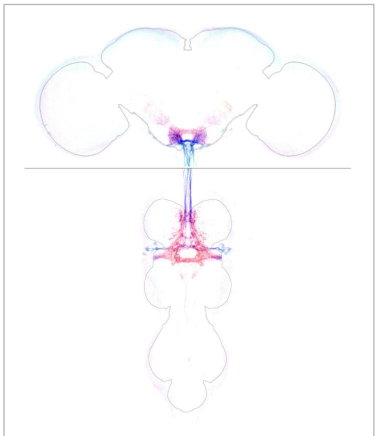
SS95406

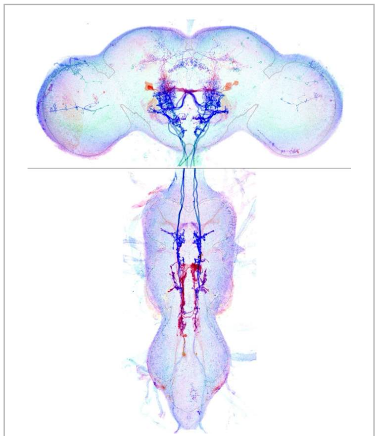
SS95408

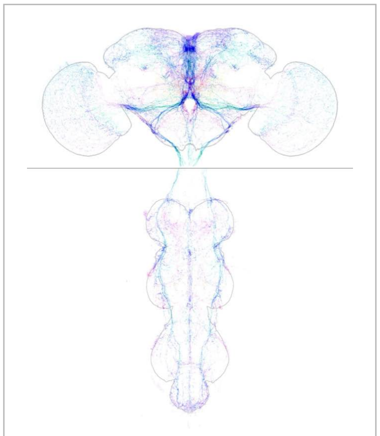
SS95441

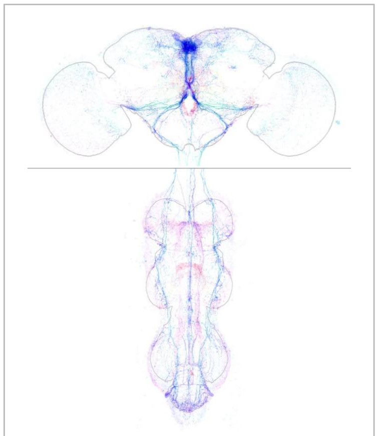
SS95458

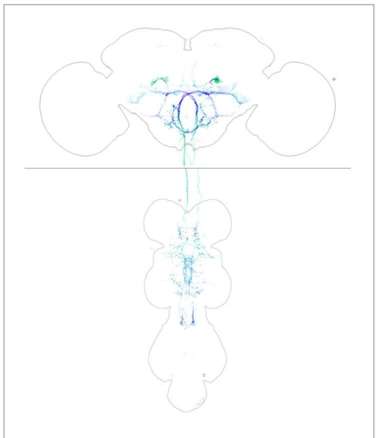
SS95510

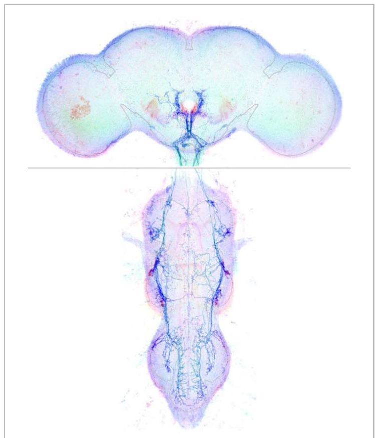
SS95555

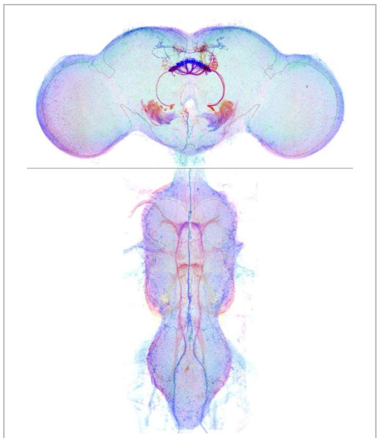
SS95617

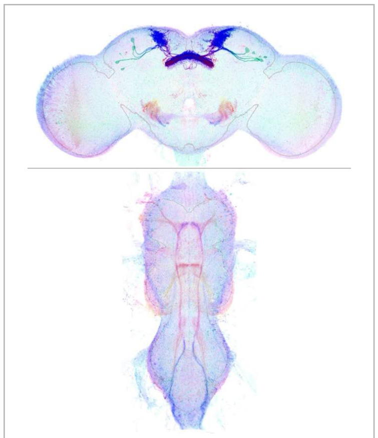
SS95649

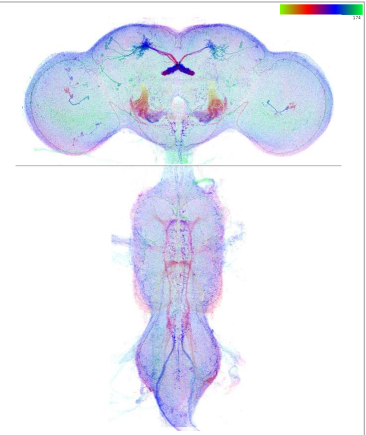

SS95726

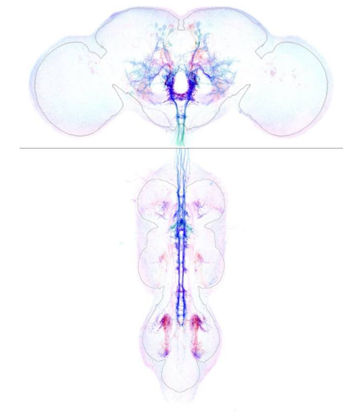

SS96091

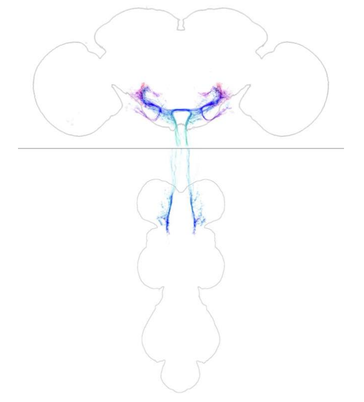

SS96100

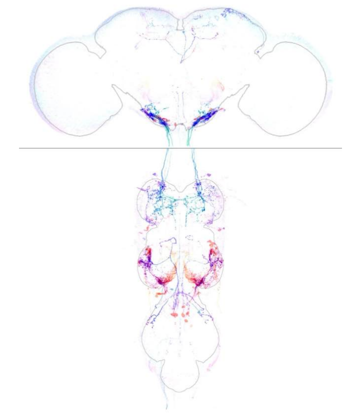

SS96120
