# Supplementary material for: A split-GAL4 driver line resource for Drosophila neuron types
Source: eLife. 2025 Jan 24;13:RP98405. doi: 10.7554/eLife.98405 (PMC11759409; doi:10.7554/eLife.98405)

Guide to image data on FlyLight websites

For: splitgal4.janelia.org & gen1mcfo.janelia.org

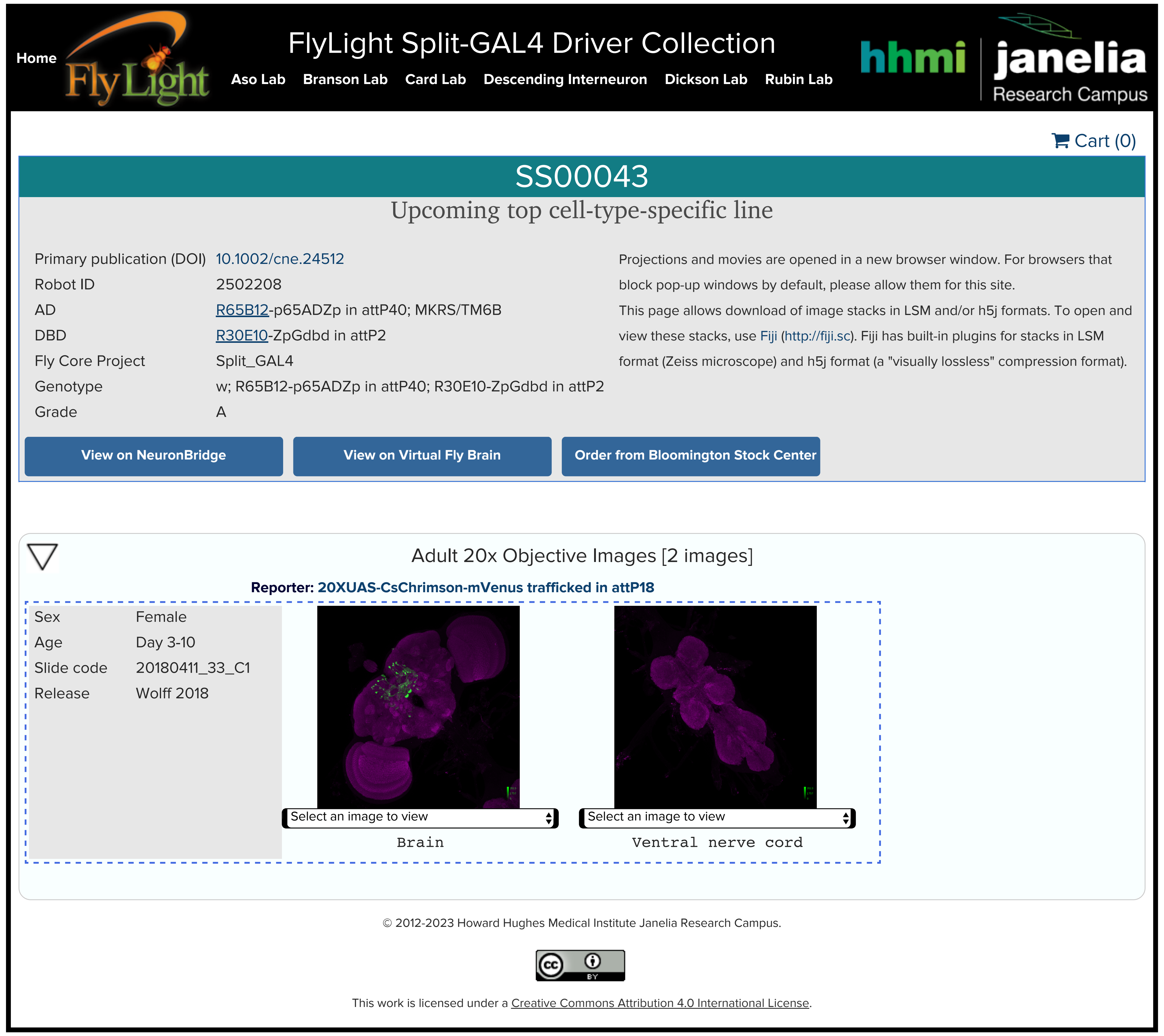

Contents:

1. Image formats
2. FlyLight sample labeling methods
3. 63x and 40x image tile names

Organization

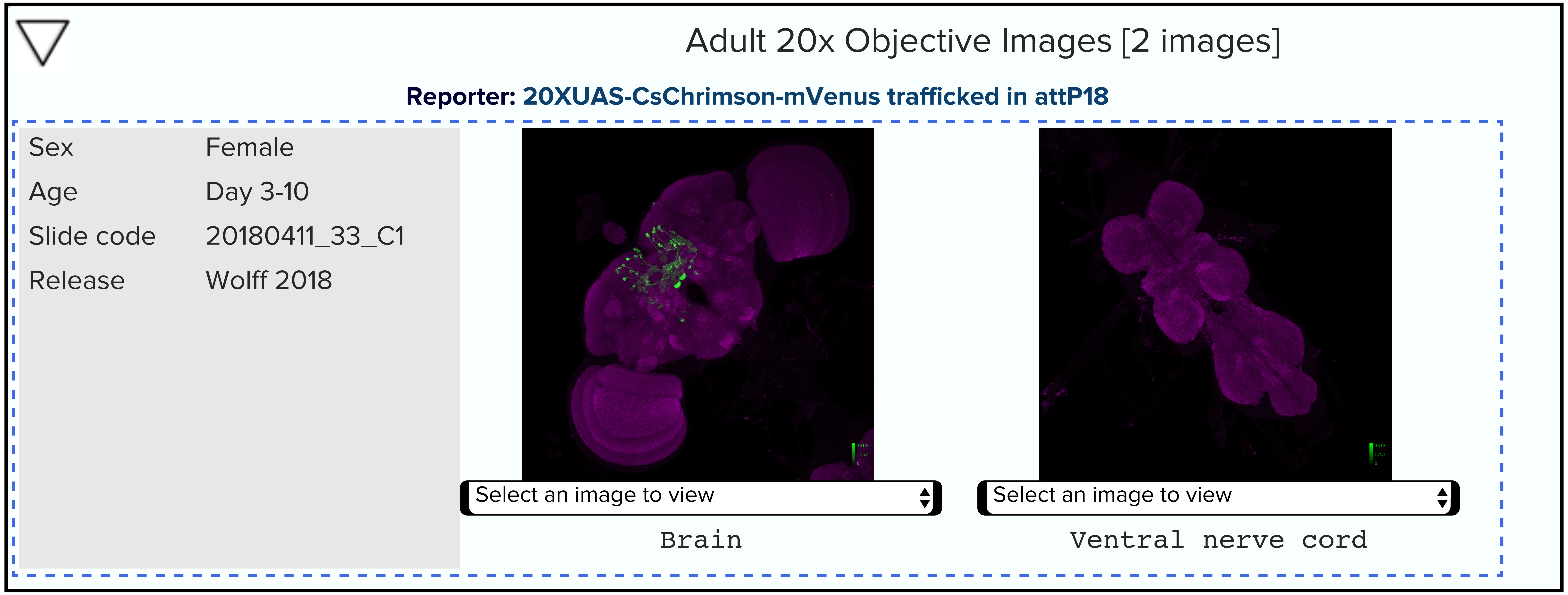

- Images for each line are grouped by objective, reporter, and sample (fly), with image tiles shown as thumbnails
- Select the menu beneath a thumbnail to view or download a variety of 2D image, movie, or microscope format 3D stacks, detailed on next page

Image formats

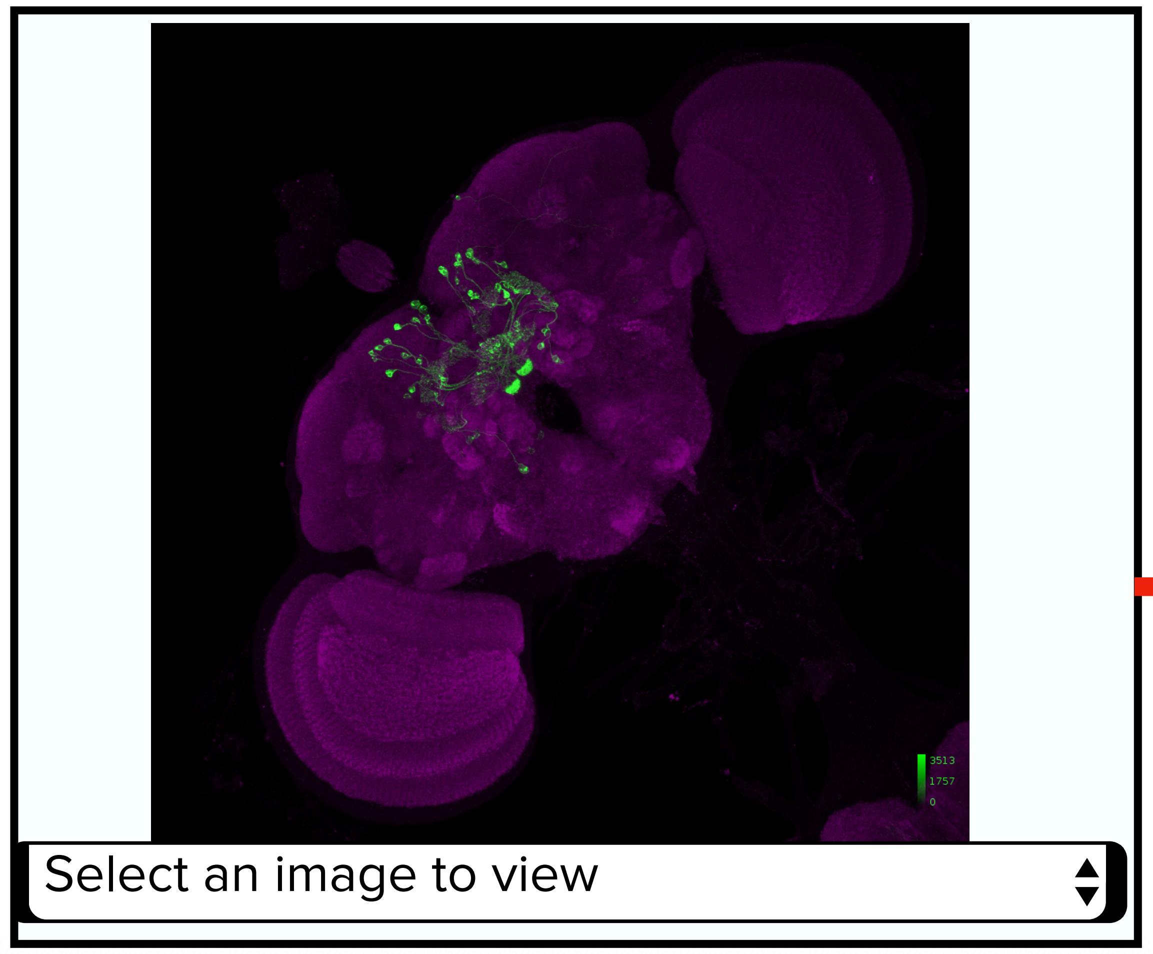

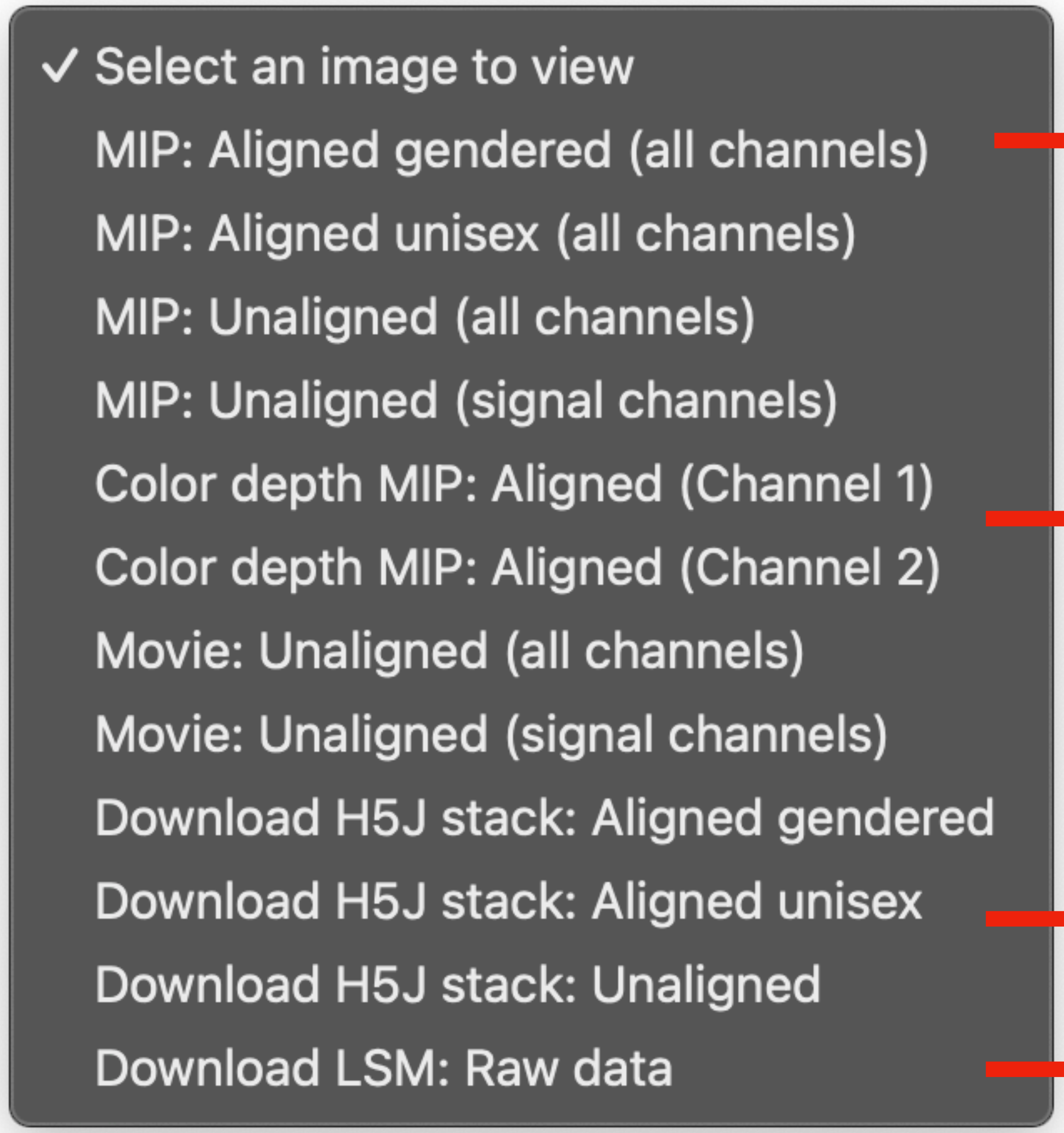

- Thumbnails show unaligned neuropil reference and neuron signal channels

- Maximum Intensity Projections (MIPs) give 2D views of data with original colors
- Images can be unaligned (as captured) or aligned to JRC2018 template (https://doi.org/10.1371/journal.pone.0236495)

- Color Depth MIPs (CDM) use color to indicate depth in z dimension; blue is close and red is far (http://dx.doi.org/10.1101/318006)

- Most stacks are H5J compressed (http://data.janelia.org/h5j)
- Original data capture format is a minimally-compressed LSM stack

Image processing details

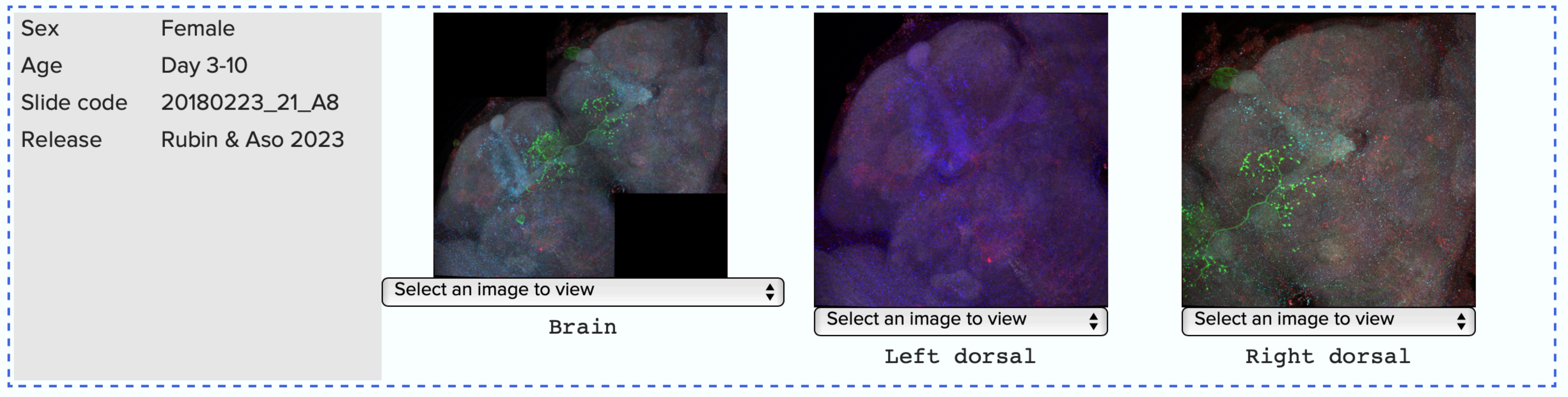

- The number of samples per line varies
- Slide code indicates date of slide creation, pipeline code, and location of individual sample on slide
- Images are individually optimized for brightness
- Some types of image outputs may show saturation; raw data (LSM stacks) have minimal saturation
- Brain and VNC images may be stitched from multiple tiles. Tiles for each sample/region are captured with consistent imaging parameters but are shown scaled independently.

FlyLight pipelines characterizing genetic lines

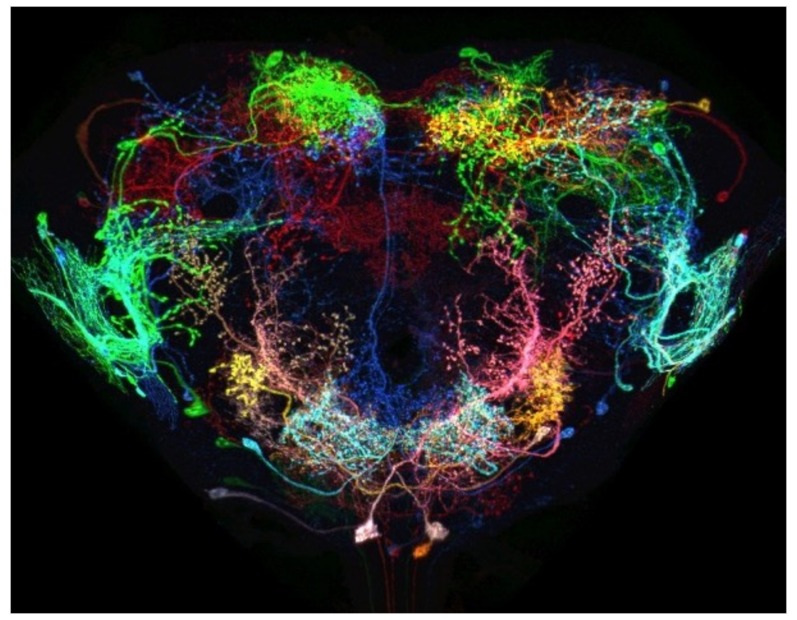

Generation 1 MCFO:
Identifying potential Gen1 GAL4 lines

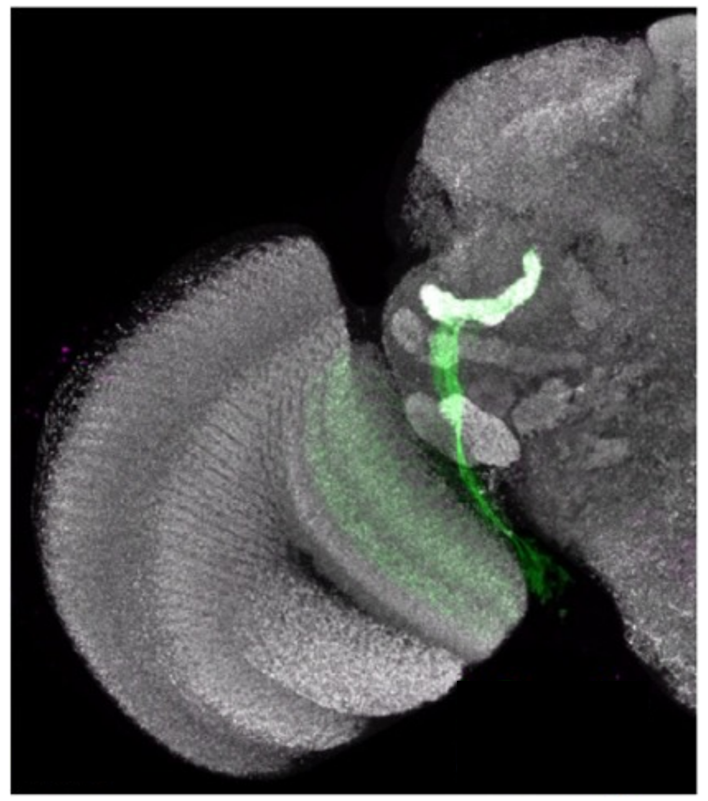

Wu, Nern, et al., 2016

Screen:
Identifying the best split-GAL4 combinations

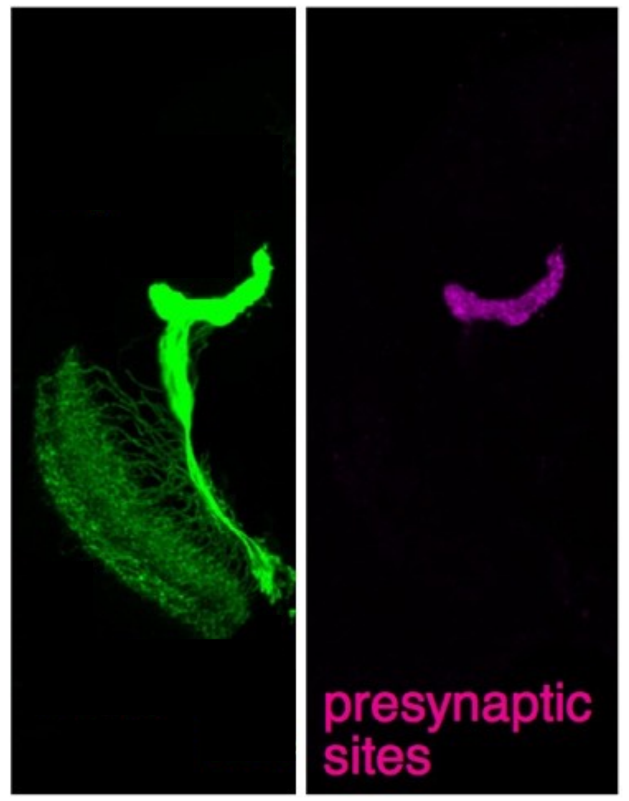

Polarity:
Determining neuronal input and output

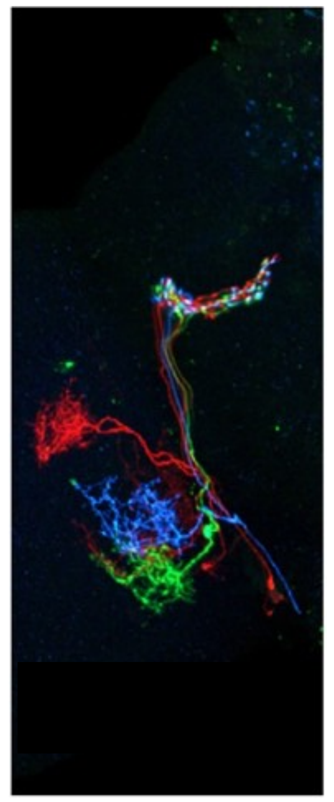

Multicolor Flp-out (MCFO):
Understanding cells within a line

Screen pipeline

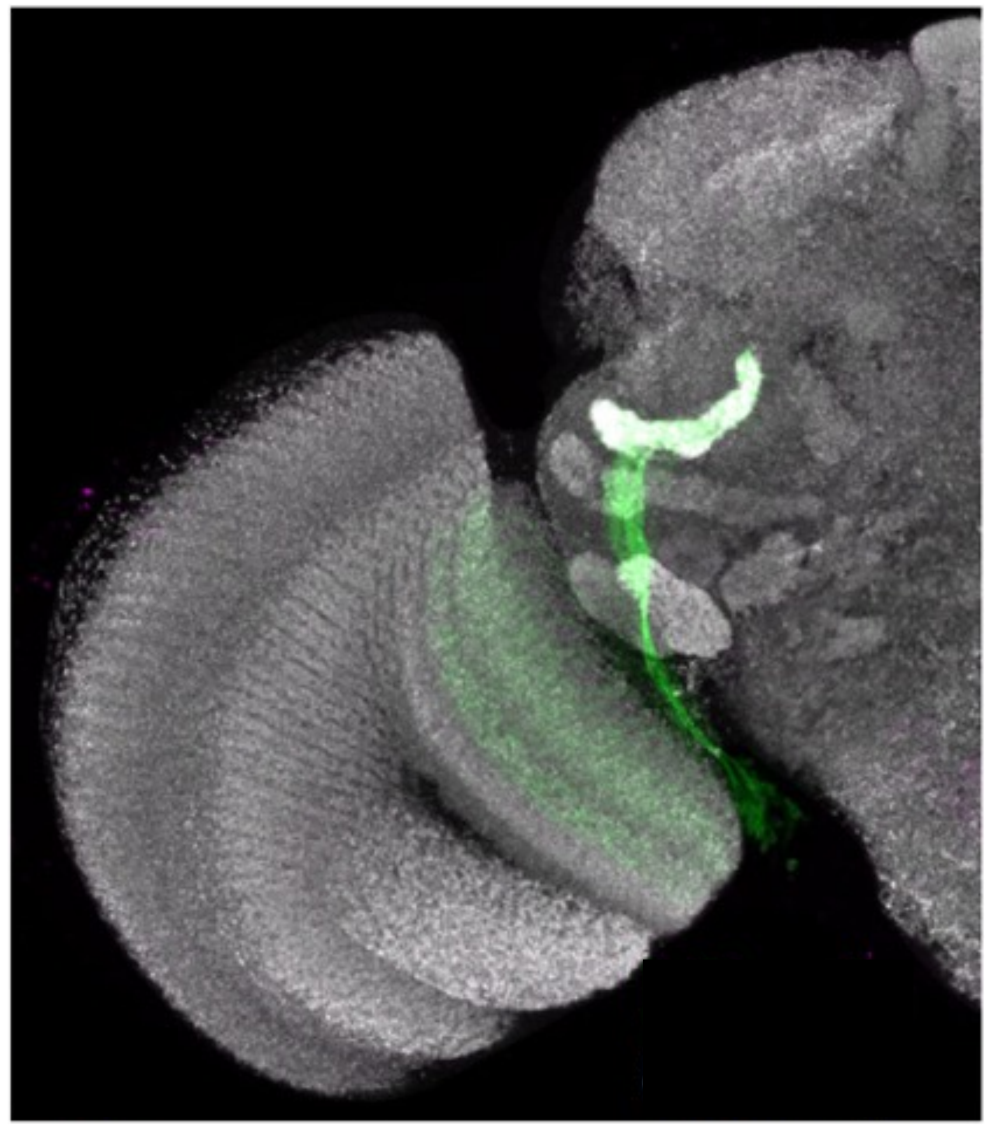

UAS reporter: 20XUAS-Cs-Chrimson-mVenus trafficked in attP18

	Screen	
Target	Reference neuropil	neuron
Genetic marker	endogenous	UAS-GFP
Primary	mouse nc82	rabbit anti-GFP
Secondary	568 anti-mouse	488 anti-rabbit

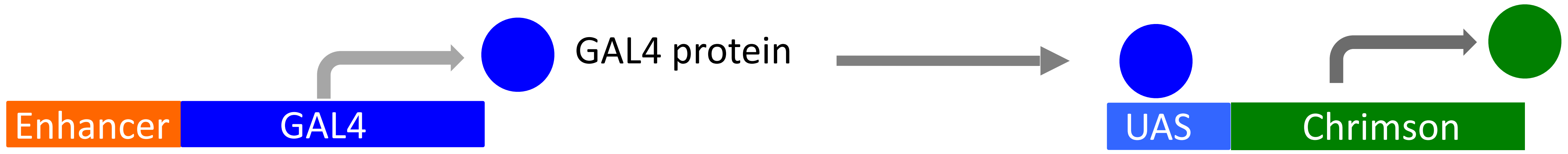

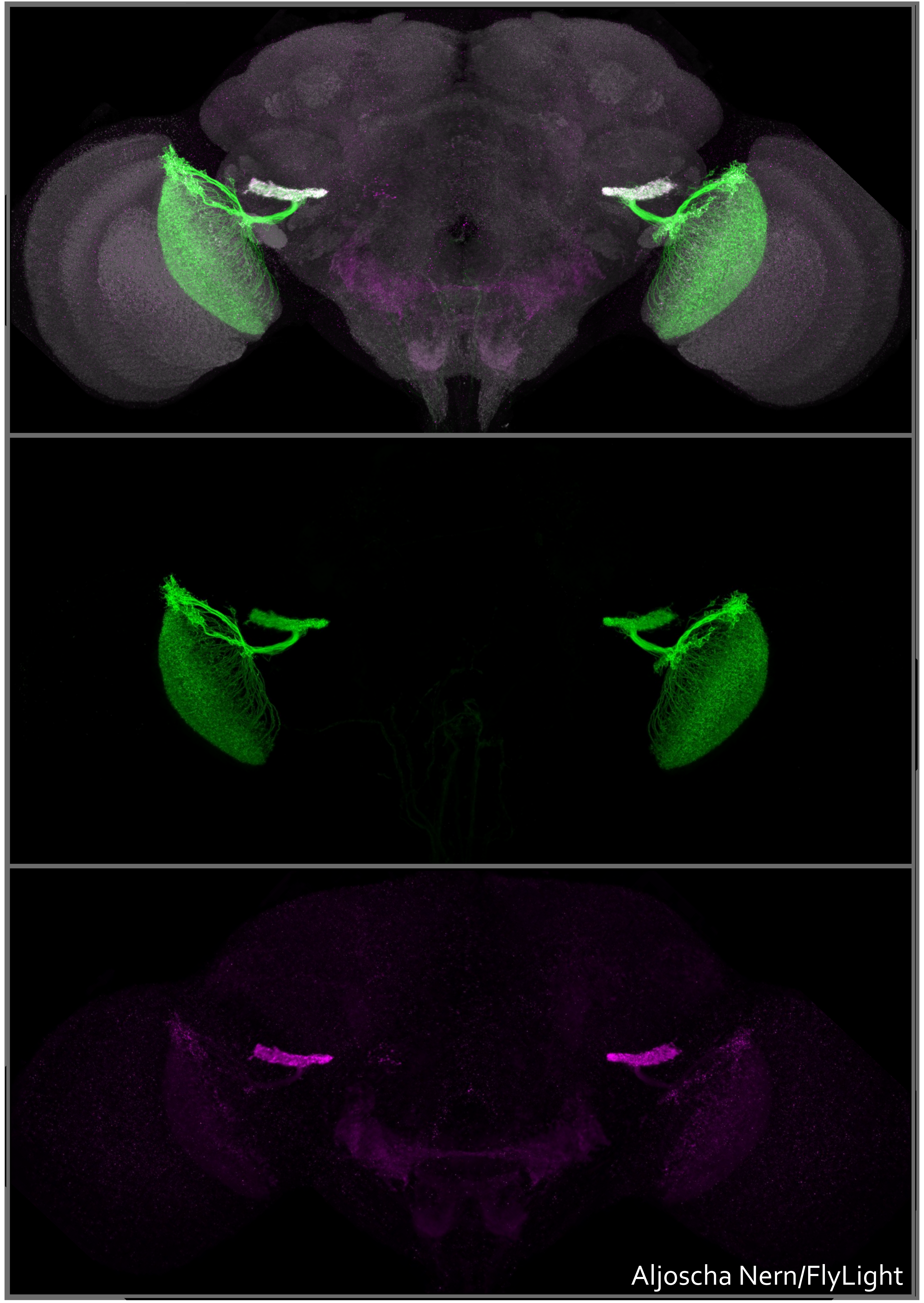

Polarity pipeline

Standard approach is to label:

- GAL4-specific neuronal membrane
- GAL4-specific presynaptic terminal
- nc82 neuropil reference

Two main purposes:

- Determine polarity (inputs and outputs) of neurons
- High-quality GAL4 membrane labeling

Polarity Cases (variations on labeling methods)

	Case 3		Case 4		Case 5	
	**Primary Ab**	**Secondary Ab**	**Primary Ab**	**Secondary Ab**	**Primary Ab**	**Secondary Ab**
**nc82 (neuropil)**	mouse nc82	Cy2 anti-mouse	mouse nc82	AF568 anti-mouse	mouse nc82	AF568 anti-mouse
**UAS-Syt-HA (synapse)**	Rabbit anti-HA	Cy3 anti-rabbit	-	-	Rat anti-HA	ATTO647N anti-rat
**UAS-myr-FLAG (membrane)**	Rat anti-FLAG	ATTO647N anti-rat	-	-	-	-
**UAS-Chrimson (membrane)**	-	-	Rabbit anti-GFP	AF488 anti-rabbit	Rabbit anti-GFP	AF488 anti-rabbit

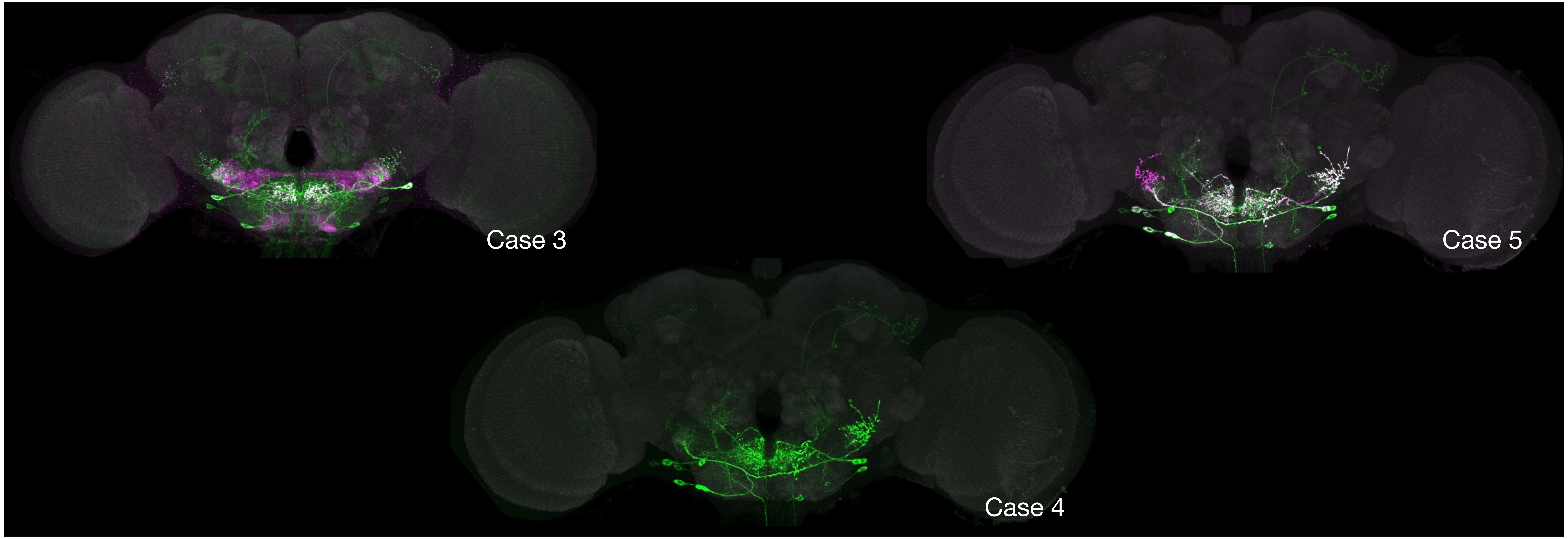

Polarity Case 3

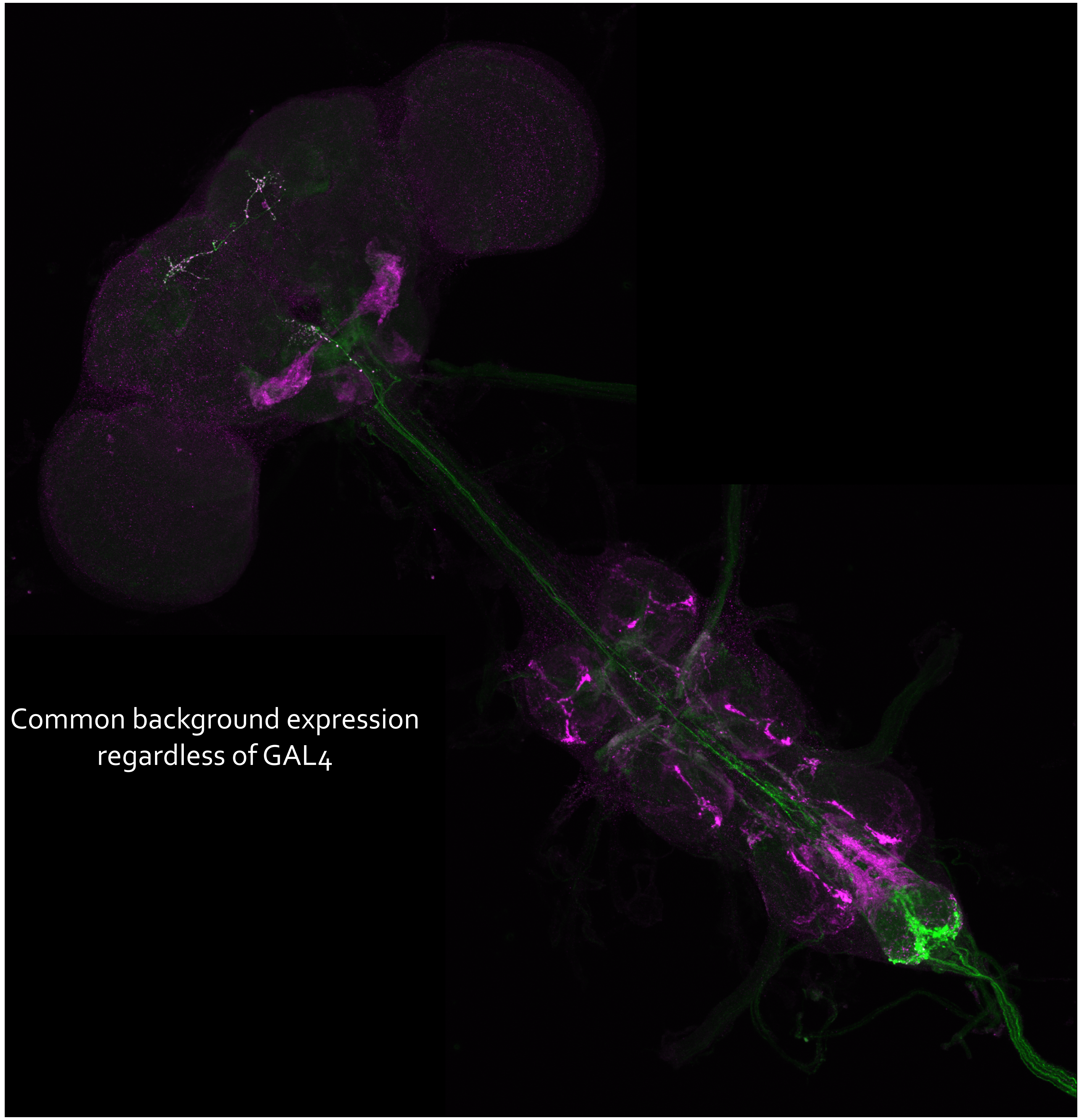

- UAS Reporters:
- Membrane: pJFRC225-5XUAS-IVS-myr::smGFP-FLAG in VK00005
- Synapse: pJFRC51-3XUAS-IVS-Syt::smGFP-HA in su(Hw)attP1

- Excellent intracellular synaptic labeling specificity
- Membrane label reliably fills GAL4 patterns
- But high background expression of membrane and synaptic reporters

- UAS-myr-FLAG reporter instead of Chrimson-mVenus
  - Different reporter from Screen & behavioral studies
    - Different attP insertion site
  - Harder to correlate Polarity pattern with other studies

Polarity Case 4

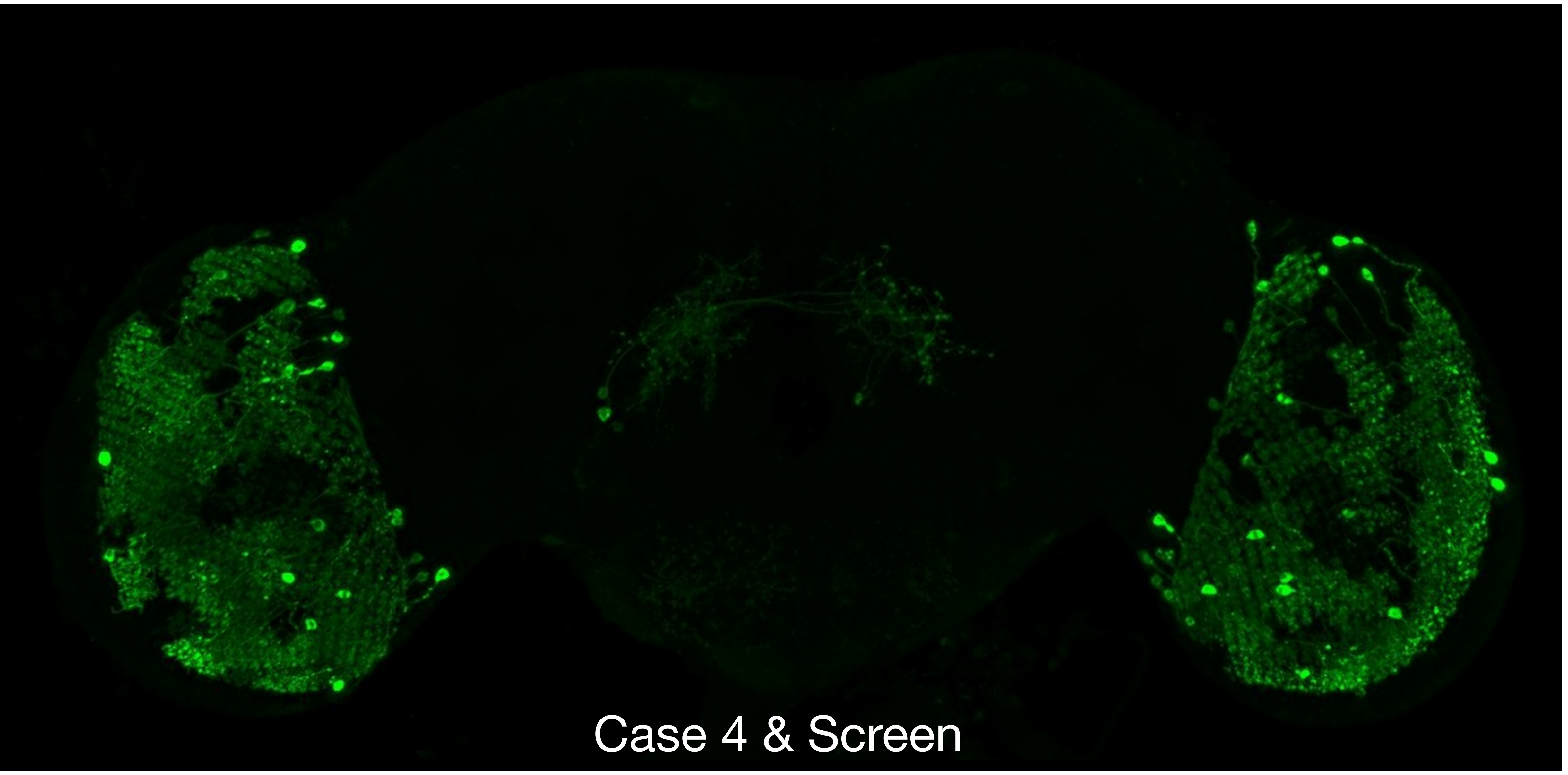

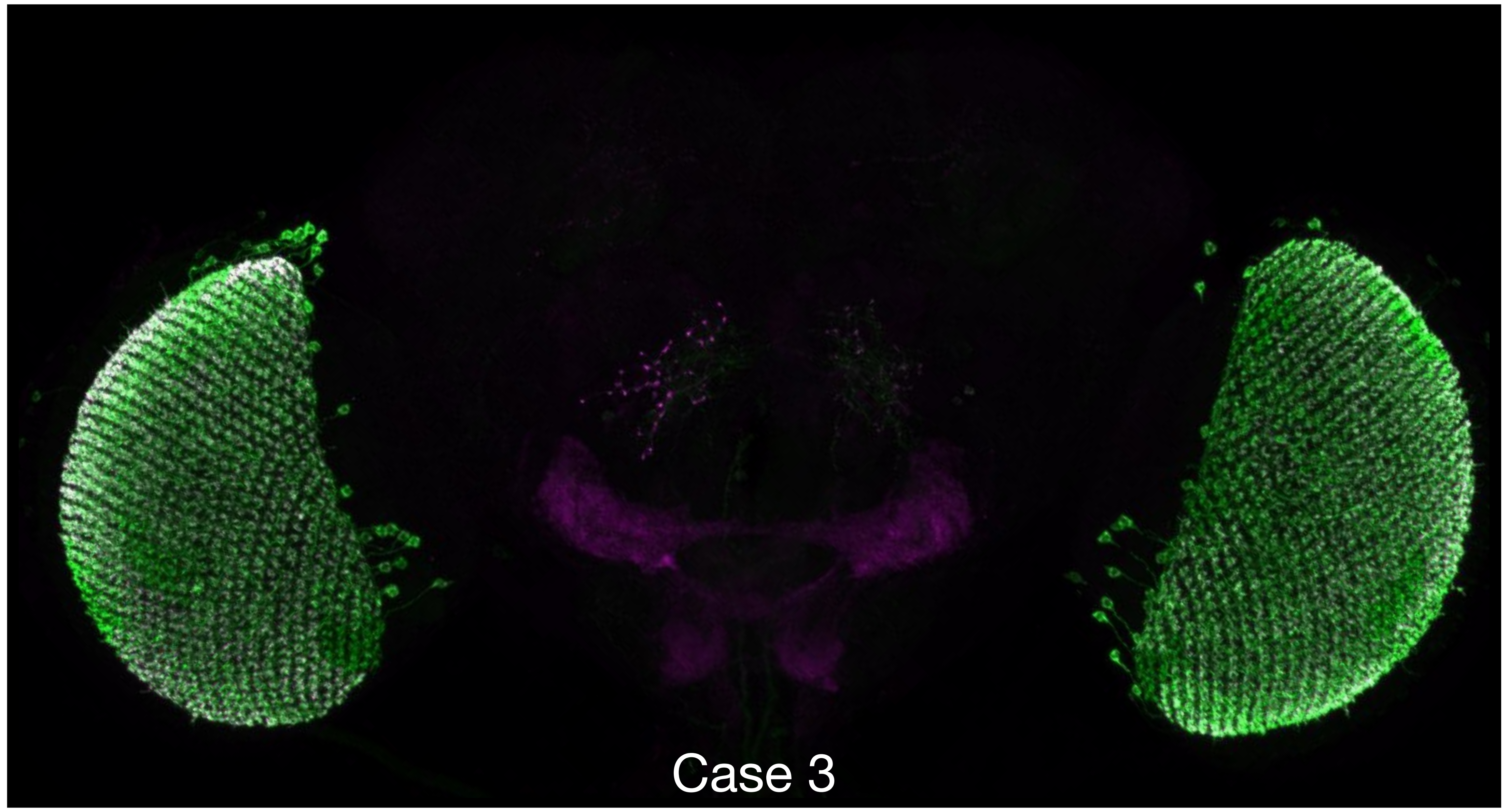

- UAS reporter: 20XUAS-Cs-Chrimson-mVenus trafficked in attP18

- Polarity Case 4 and Screen pipelines use the same UAS reporter and antibodies
- UAS-Chrimson-mVenus reporter allows direct comparison to physiology
- No synaptic label, despite Polarity name
- Very low background
- Less uniform labeling of GAL4 pattern than UAS-myr-FLAG
  - Greater variation between cell body and projection intensities
  - Doesn't always label all neurons in GAL4 pattern

Polarity Case 5

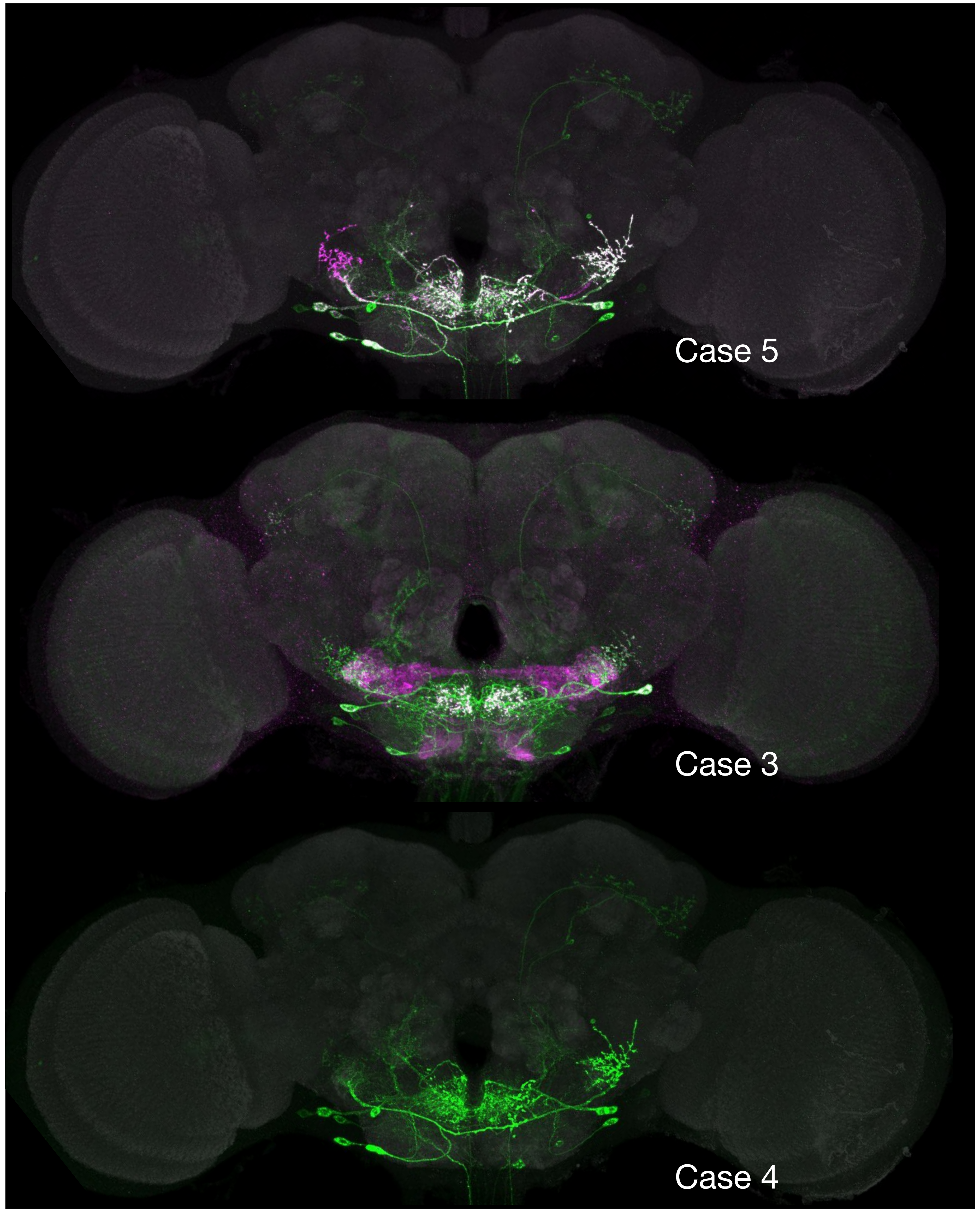

- UAS Reporters:
- Membrane: 20XUAS-Cs-Chrimson-mVenus trafficked in attP18
- Synapse: UAS-Syt-HA (different from Case 3)

- Chrimson-mVenus instead of myr-FLAG
    - Maximizes consistency with screening & physiology results
- Labels presynaptic terminals, but less accurate intracellular localization than Case 3
- Very low background in most cases
- Common contaminants can grow during IHC and be labeled by anti-GFP antibody, requiring sodium azide suppression

MultiColor FlpOut (MCFO) subdivides GAL4 & split-GAL4 lines

Full GAL4 pattern with UAS-Chrimson-mVenus reporter

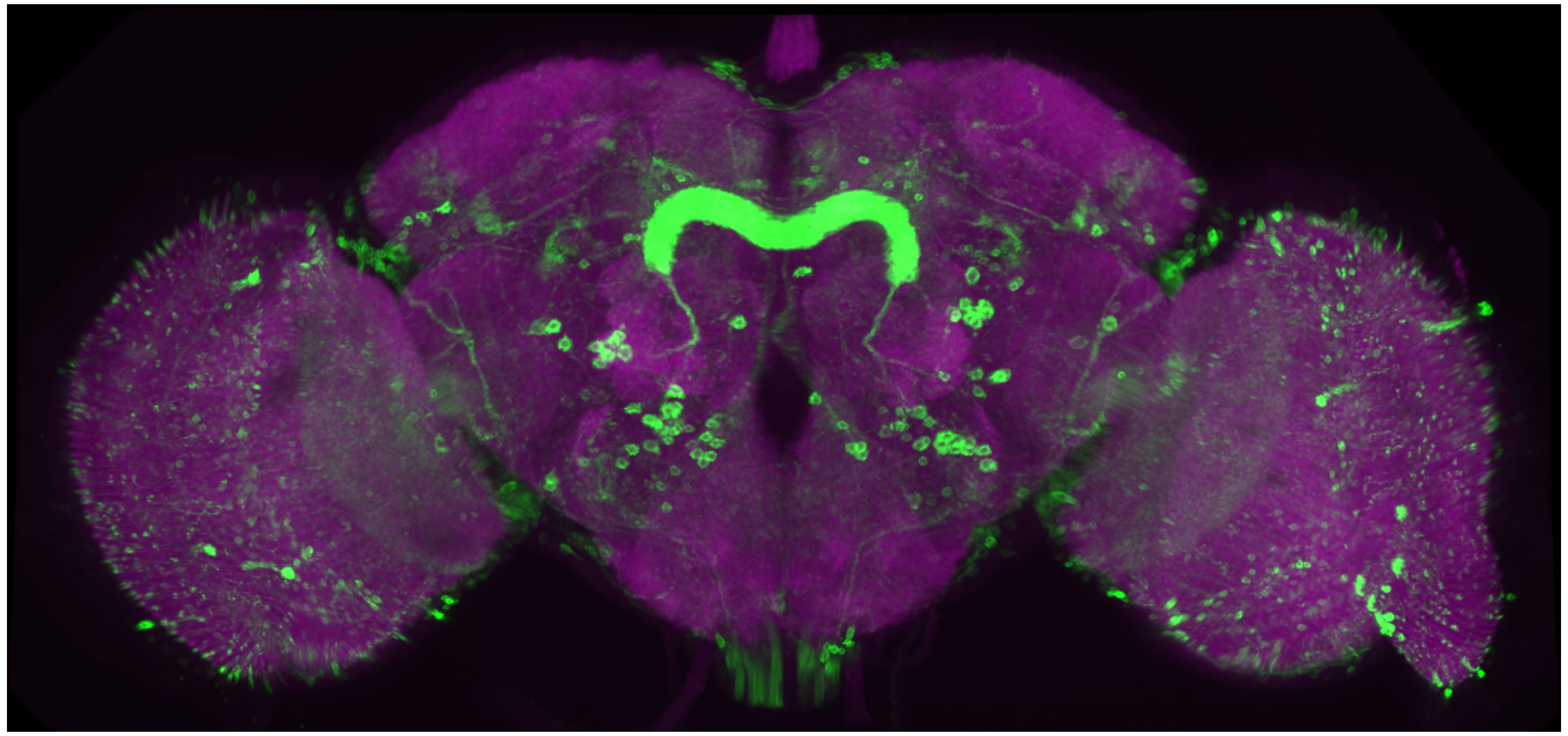

Subsets of neurons labeled in different colors by MultiColor FlpOut (MCFO)

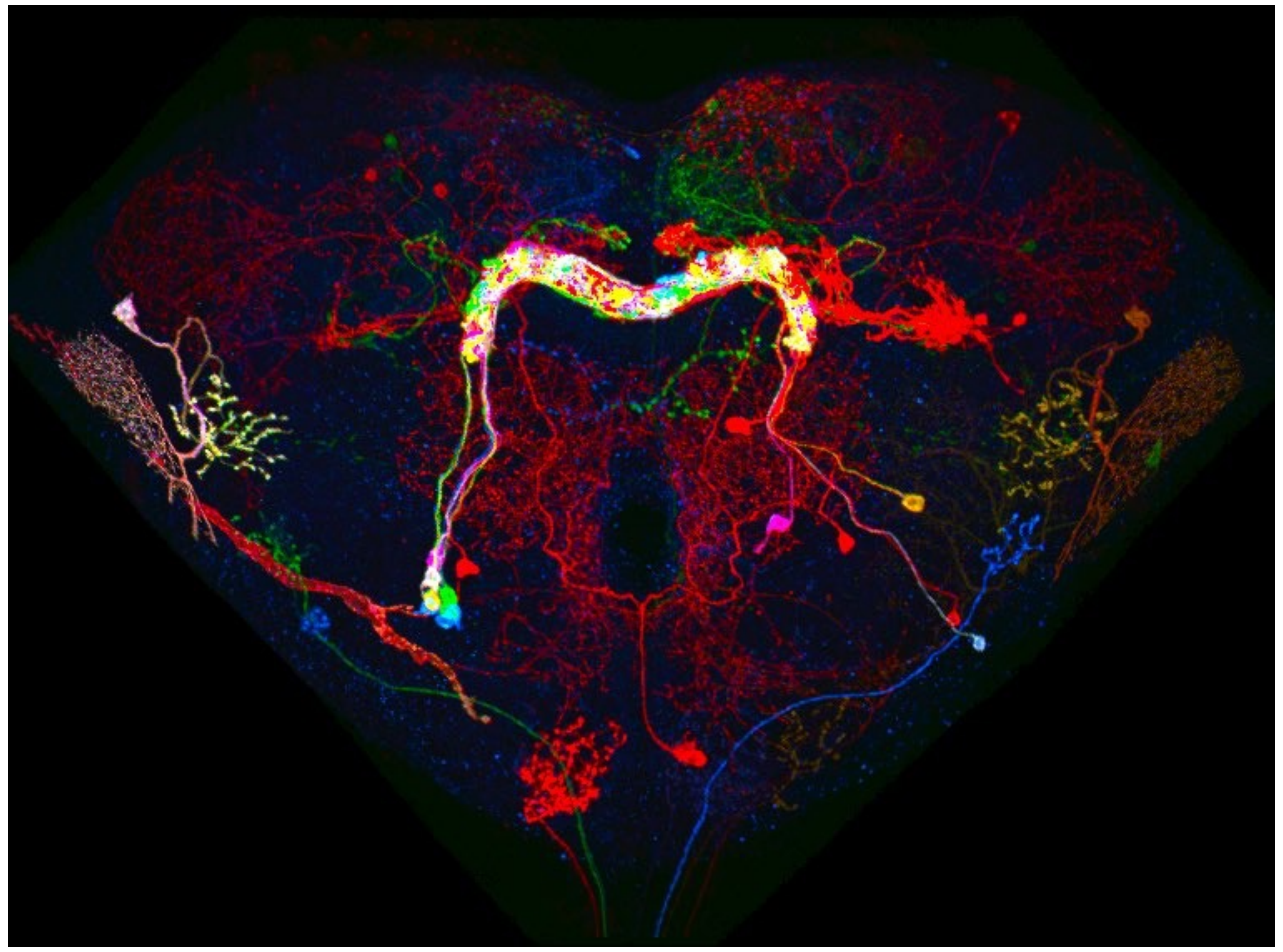

Multicolor FlpOut (MCFO)

MCFO-1 UAS Reporter:
pBPhsFlp2::PEST in attP3;
pJFRC201-10XUAS>STOP>myr::smGFP-HA in VK0005,
pJFRC240-10XUAS>STOP>myr::smGFP-V5-THS-10XUAS>STOP>myr::smGFP-FLAG in su(Hw)attP1

	Multicolor Flp-out			
Target	Reference neuropil	neuronal membrane 1	neuronal membrane 2	neuronal membrane 3
Genetic marker	endogenous	UAS>>HA	UAS>>V5	UAS>>FLAG
Primary/Direct Ab	mouse nc82	rabbit anti-HA	DL550 anti-V5	rat anti-FLAG
Secondary Ab	AF488 anti-mouse	AF594 anti-rabbit		ATTO647N anti-rat

See Nern et al., 2015 for more details on MCFO variations

https://doi.org/10.1073/pnas.1506763112

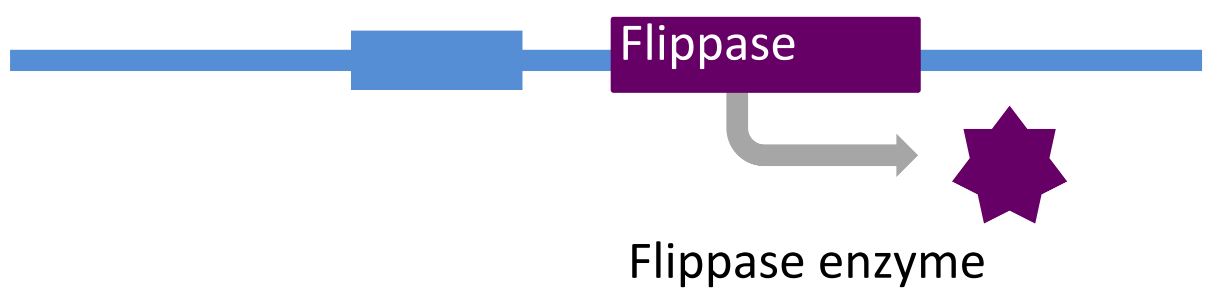

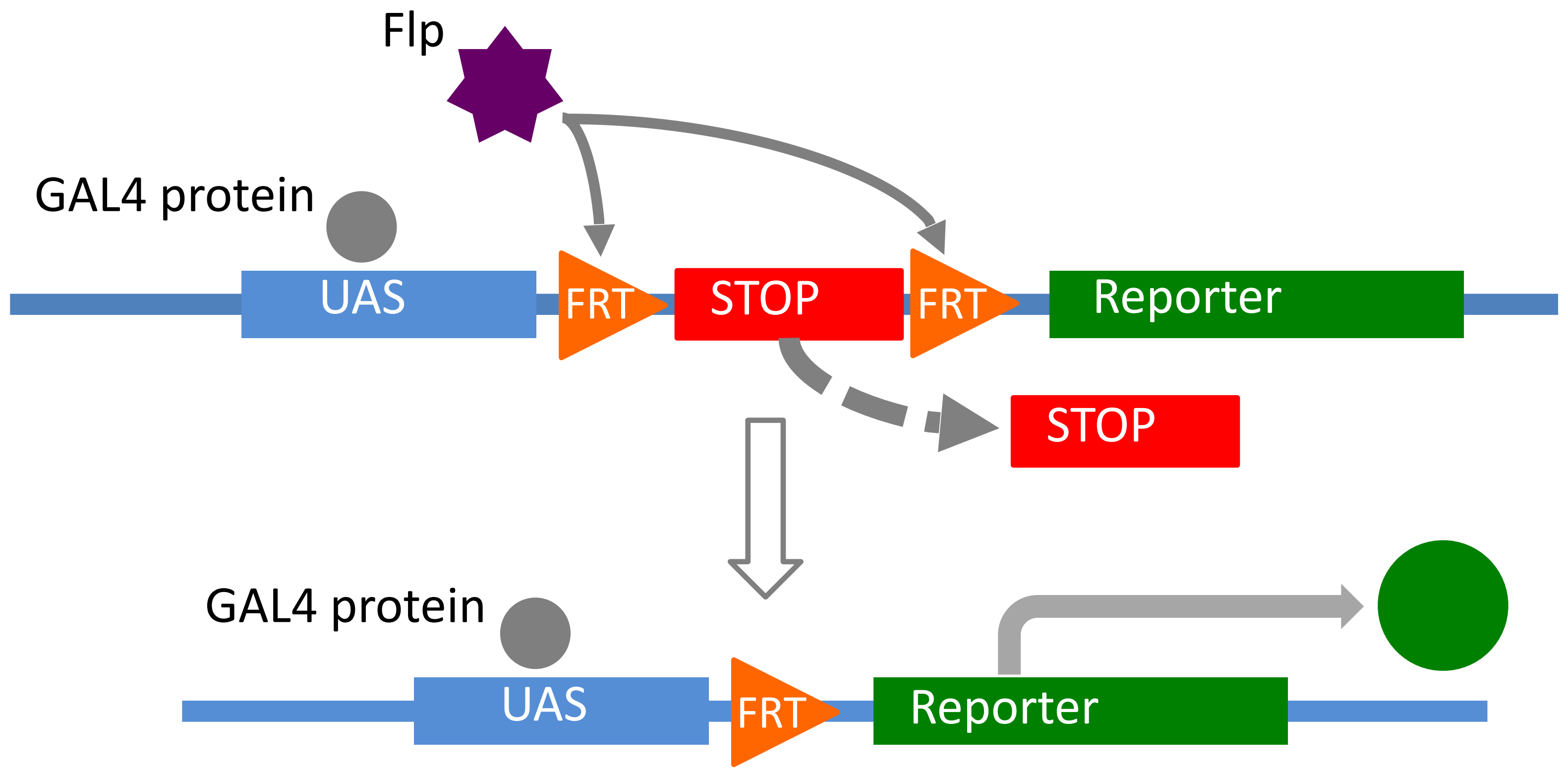

63X Adult Tiles - Brain

L – Left Optic Lobe

V – Ventral

LD – Left Dorsal

RD – Right Dorsal

R – Right Optic Lobe

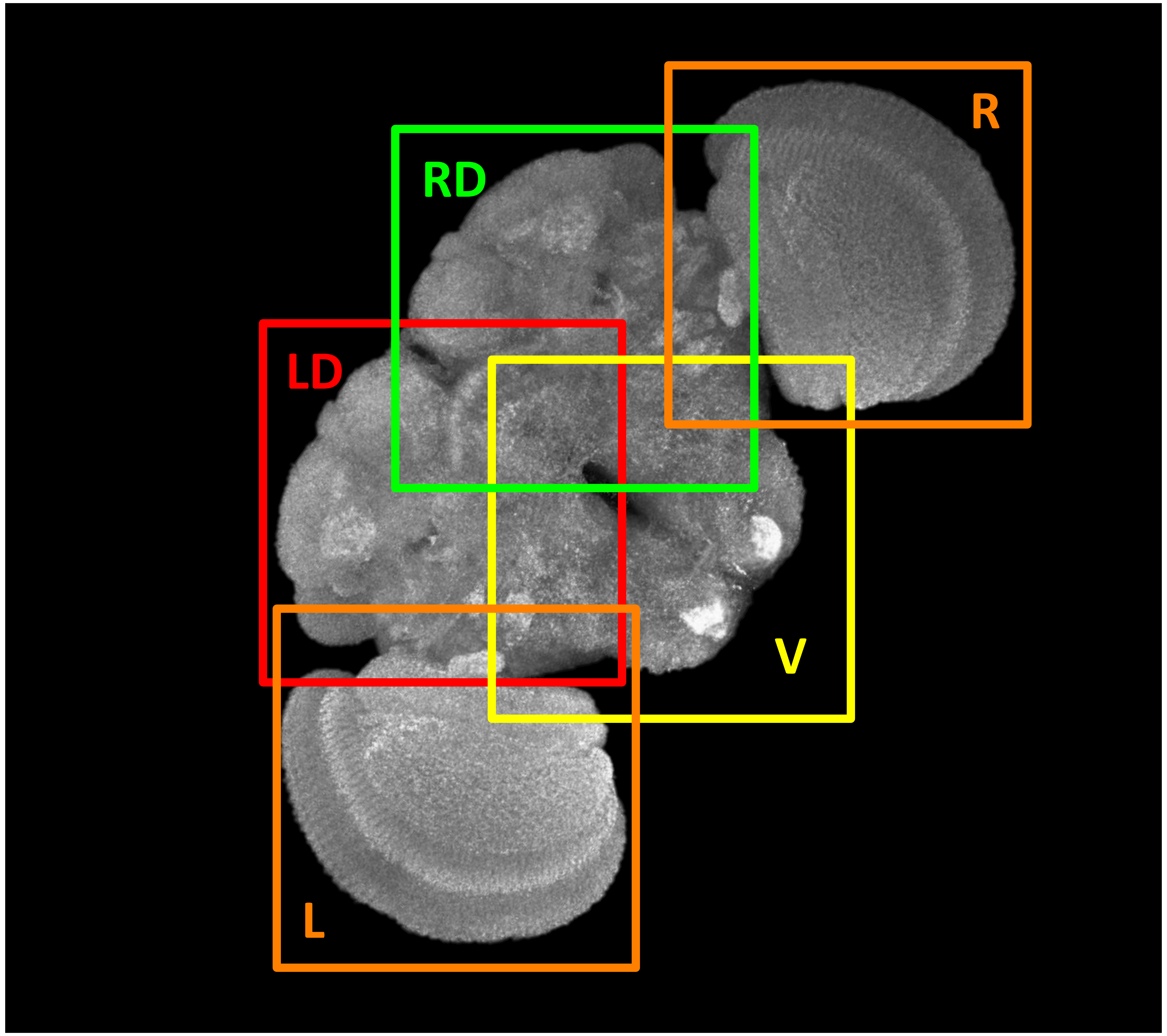

63X Adult Tiles – Additional Brain Tiles

DM – Dorsal Medial

CEN – Central

LC – Left Central

RC – Right Central

LV – Left Ventral

RV – Right Ventral

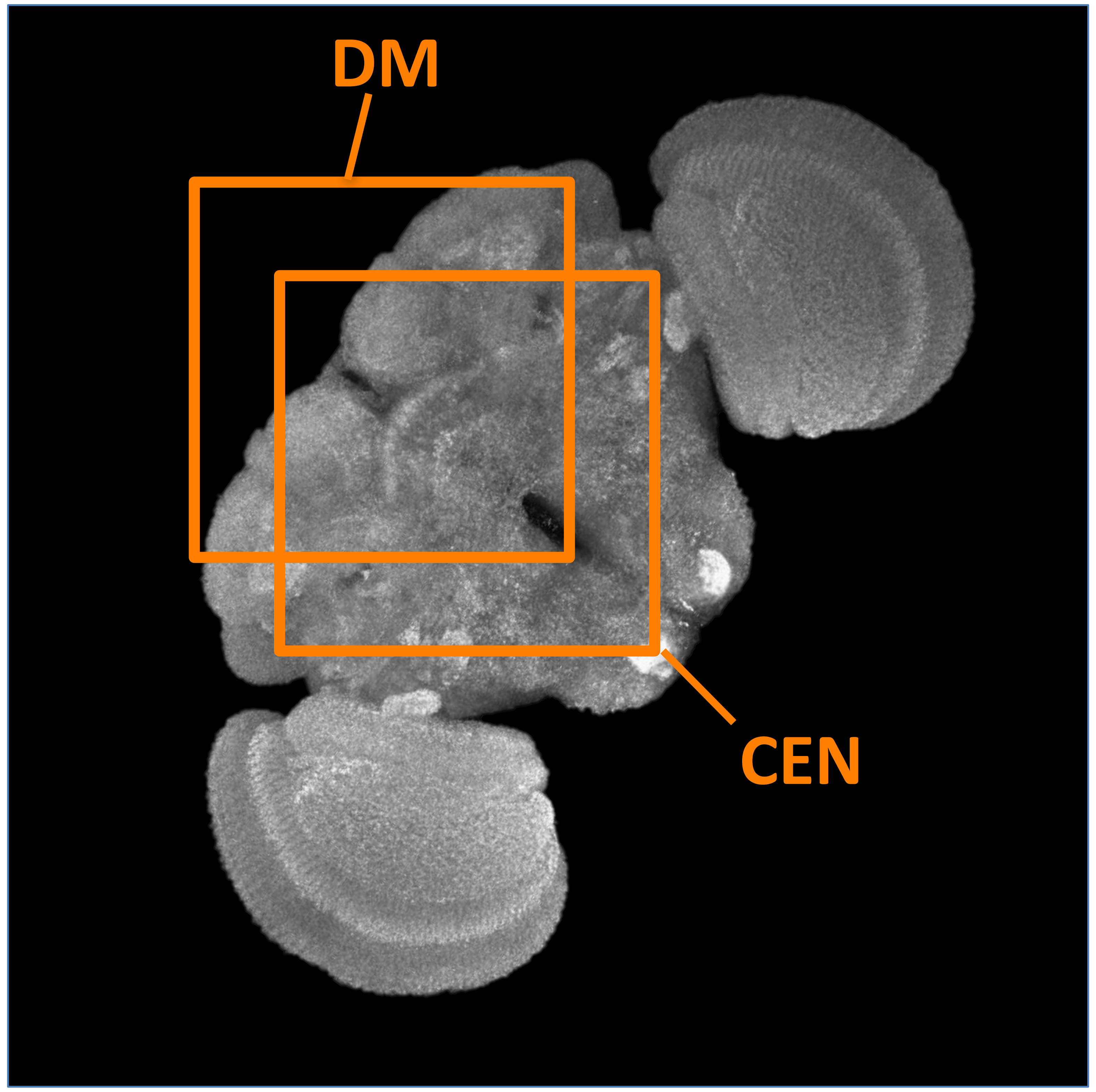

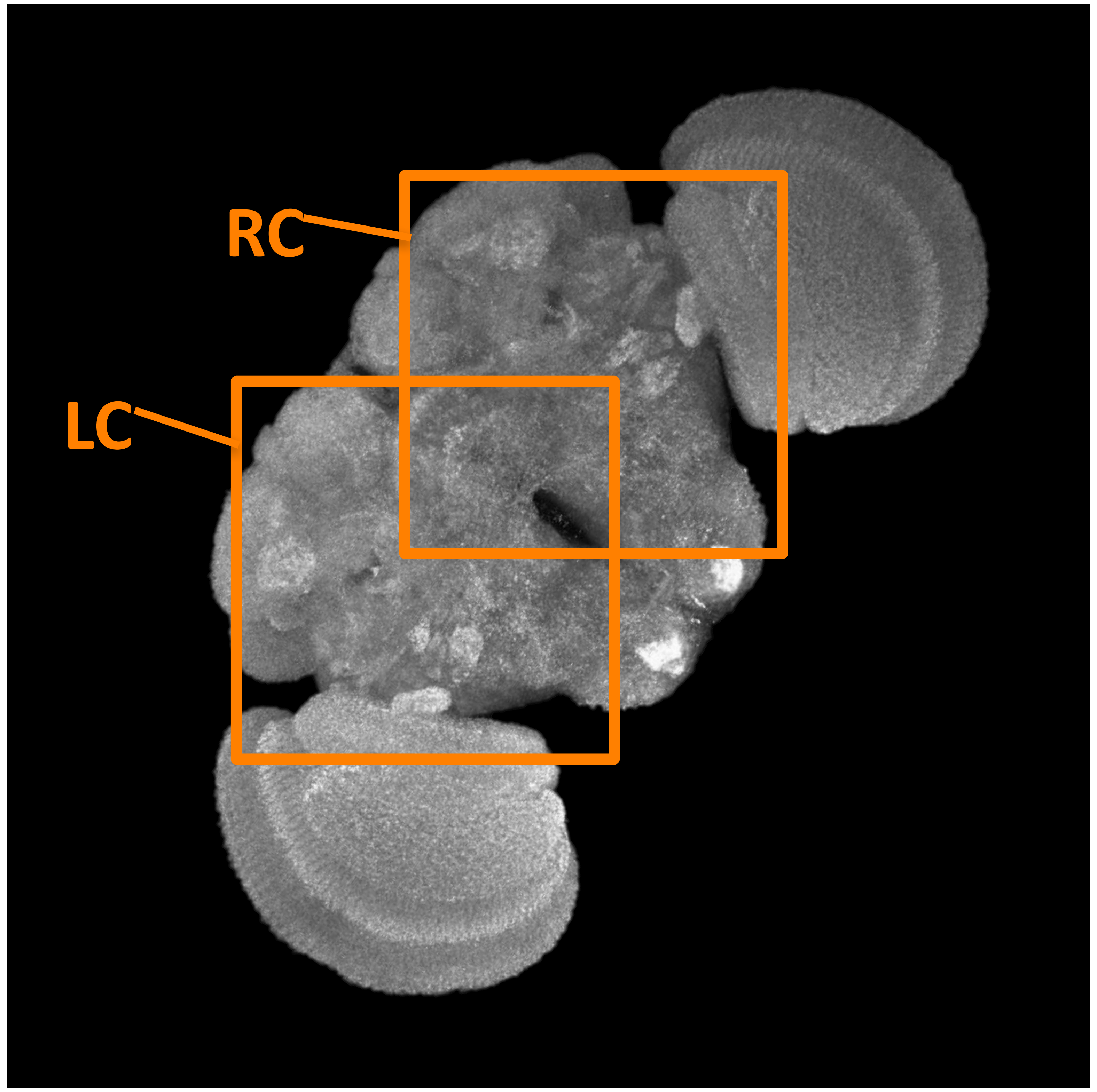

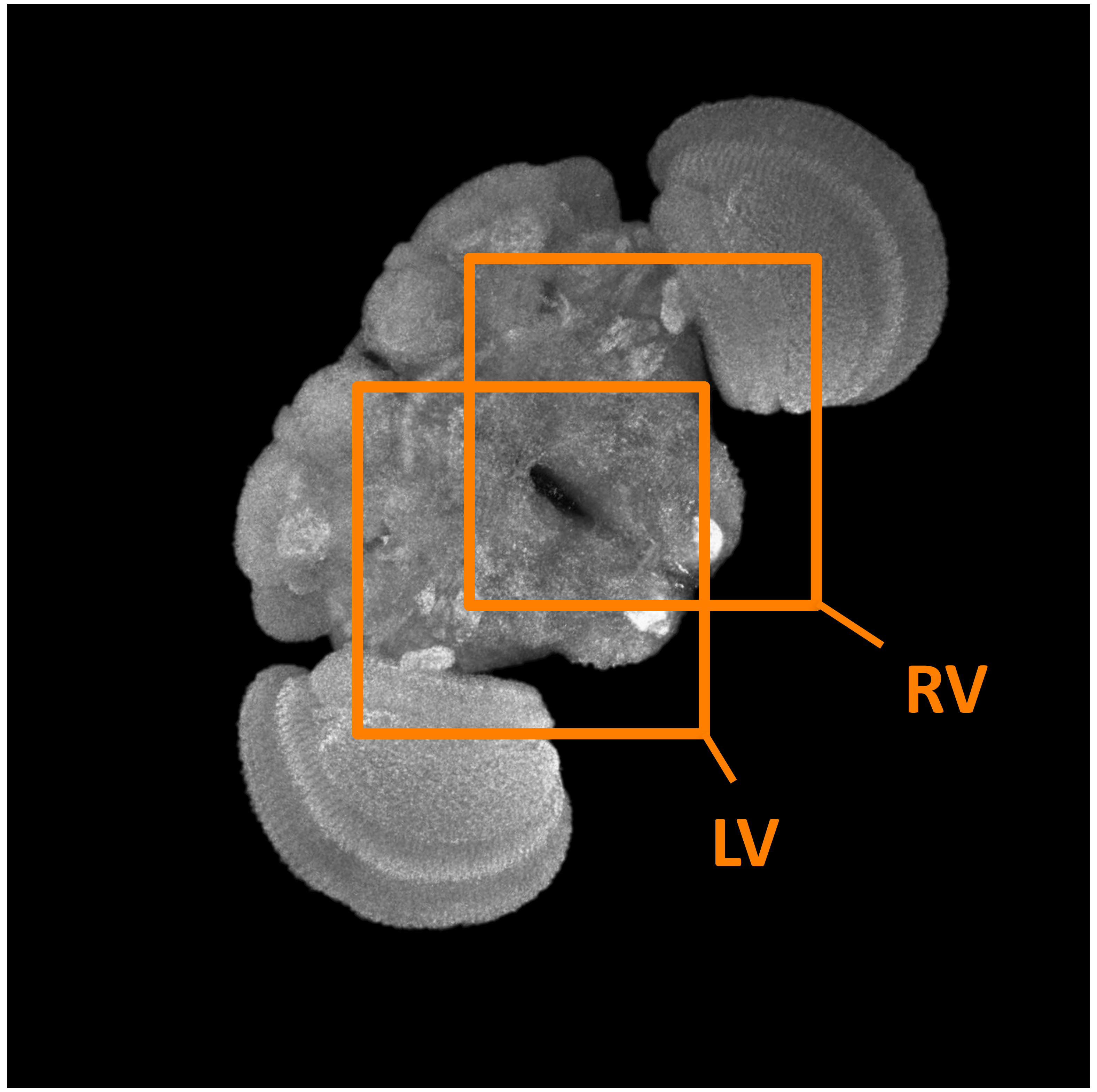

63X Adult Tiles - VNC

PRO – **Prothoracic**

MES – **Mesothoracic**

META– **Metathoracic**
(sometimes covers Abd)

ABD – **Abdominal**

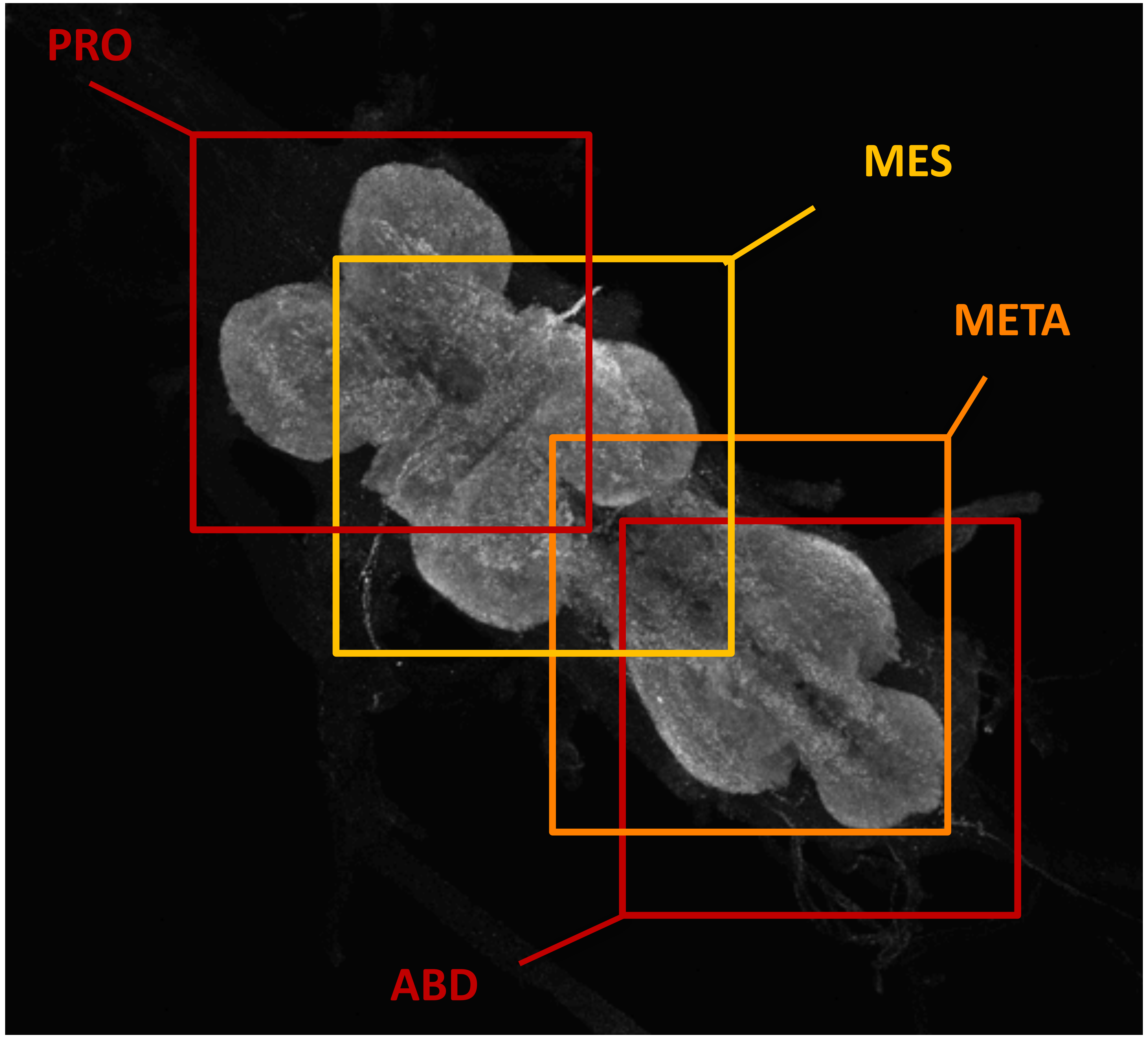

63X Adult Tiles - Neck

NEC-Ant – **Anterior Neck**

NEC-Pos – **Posterior Neck**

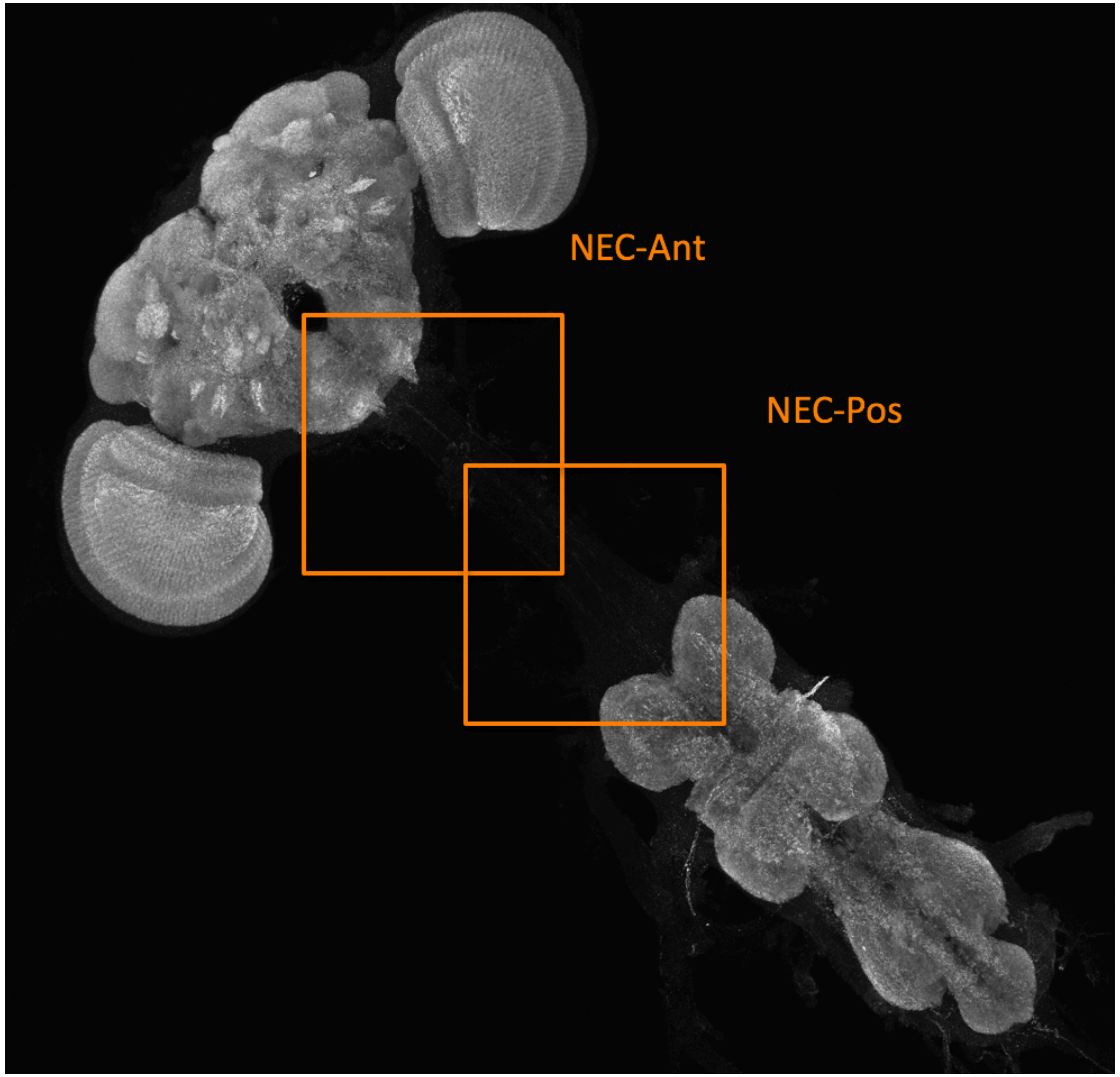

40X Adult Tiles - Brain & VNC

BRAIN – 1 tile:

CEN - Central brain

VNC – 2 tiles:

PRO - Prothoracic (anterior VNC)

META - Metathoracic (posterior VNC)

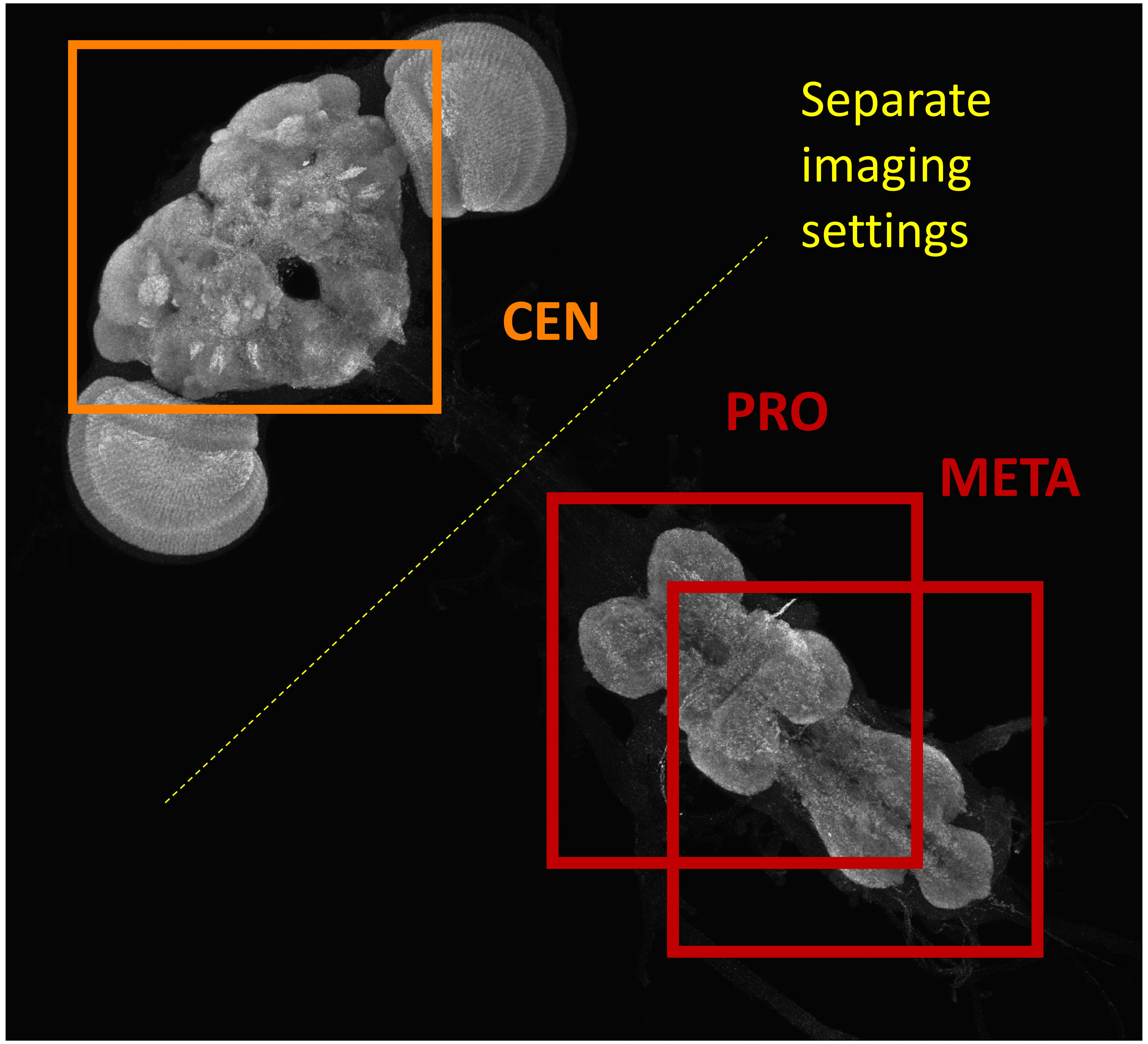

Supplement: Supplementary file 2. — A guide to interpreting the images at https://gen1mcfo.janelia.org and https://splitgal4.janelia.org further describes data organization, labeling, and imaging methods. Images at https://www.janelia.org/gal4-gen1 are processed as described in Jenett et al., 2012. [file elife-98405-supp2.pdf]
